# Luminescent Liquid-Crystalline J‑Aggregate Based on a Columnar Axial Coassembly

**DOI:** 10.1021/jacs.5c03166

**Published:** 2025-05-28

**Authors:** Llorenç Rubert, Clémence Marre, Pedro Ximenis, Rosa M. Gomila, Antonio Frontera, Bartolome Soberats

**Affiliations:** Department of Chemistry, 16745 Universitat de les Illes Balears, Cra. Valldemossa Km 7.5, 07122 Palma de Mallorca, Spain

## Abstract

Controlling the self-assembly of dyes is essential for designing functional materials with tailored optical, electronic, and mechanical properties. However, achieving precise structures from two distinct chromophores remains a major challenge in the field, requiring sophisticated strategies to direct their organization at the molecular level. In the present work, we report a novel approach to engineer complex liquid-crystalline (LC) columnar nanostructures through the precise coassembly of two bis-dendronized chromophores: a tris­(*p*-phenyleneethynylene) (TPE) dicarboxylic acid (**1**) and a tris­(*p*-phenylenevinylene) (TPV) bis­(pyridine) (**2**). TPE **1** forms an unconventional four-stranded orthogonal columnar LC phase via hydrogen bonding between carboxylic acid groups, while TPV **2** adopts a lamellar soft-crystalline phase. Remarkably, their equimolar mixture (**1·2**) gives rise to an unprecedented two-component columnar liquid crystal. This coassembly is grounded on the complementary hydrogen bonding between pyridine and carboxylic acid groups that leads to the formation of 1D strands composed of alternating molecules of **1** and **2**. These strands hierarchically organize by π–π interactions into eight-stranded columnar structures in which the **1**/**2** molecules are oriented with the transition dipole moments parallel to the columnar axis. This configuration promotes slipped π–π interactions and J-type coupling of the TPE and TPV components, resulting in a fluorescent LC material. This work paves the way for the design of precision multicomponent assemblies, opening exciting avenues for advanced optoelectronic and photonic materials.

## Graphical abstract

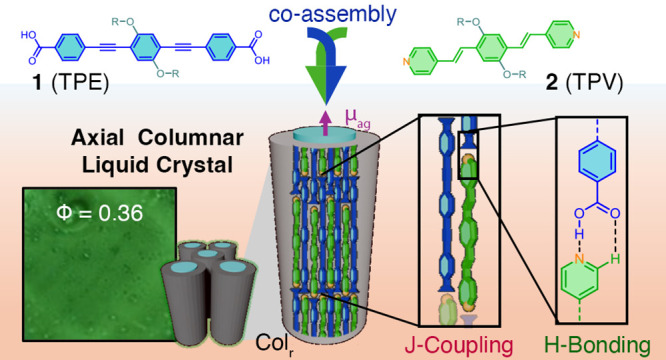

## Introduction

Columnar liquid crystals have attracted significant attention for their unique structural and anisotropic properties, positioning them as a versatile class of materials of interest for both fundamental research and advanced technological applications.^1–20^ Among the columnar liquid-crystalline (LC) materials, those consisting of π-conjugated molecules (discotic) are of special interest due to their electronic and optical properties that enable applications in electronics, photonics, and sensing, among others.^21–29^ These columnar phases are commonly formed via the stacking of the π-conjugated cores, which organize into columns that, in turn, arrange into different lattices (hexagonal, rectangular, etc.).^1–29^ The stacking of the π-conjugated molecules occurs in a cofacial manner resulting in the dyes lying perpendicular to the columnar axis (Figure 1a), which typically induces H-type couplings.^30–32^ Recently, a new type of assembly mode in dye-based columnar phases has been unveiled and is characterized by the orientation of the molecules with their π-conjugated cores parallel to the columnar axis (Figure 1b, left).^33–39^ This unconventional assembly mode provides not only new anisotropic features but also new exciton coupling possibilities, opening the door to the development of novel photonic systems.^40–42^

**Figure 1:**
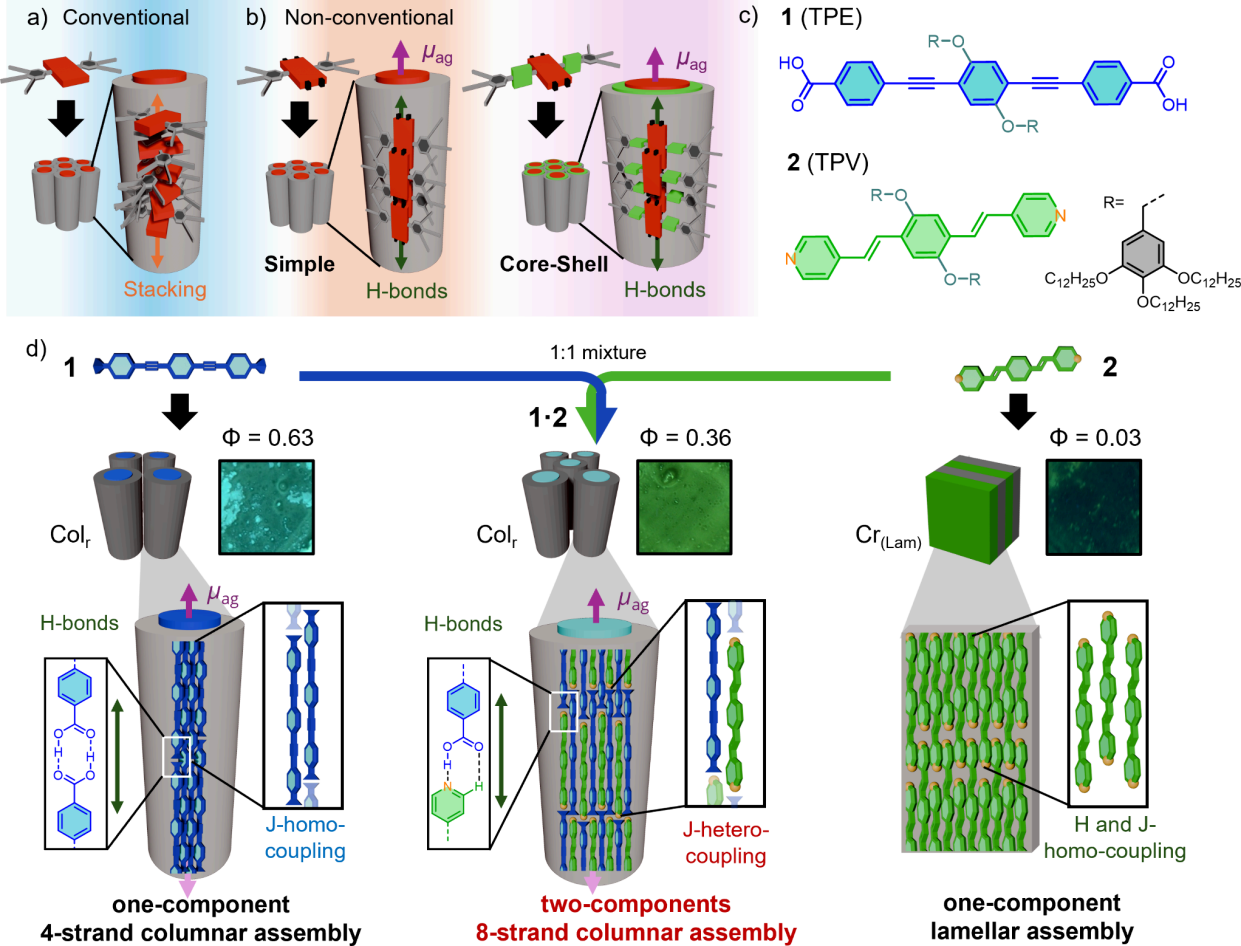
(a) Schematic representation of a conventional columnar LC assembly based on a disc-like liquid crystal. (b) Schematic representation of nonconventional simple (left) and core–shell (right) columnar LC assemblies with the chromophores oriented parallel to the columnar axis. The orientation of the transition dipole moments (μ_ag_) is illustrated with purple arrows, and the direction of the H-bonds is illustrated with green arrows. (c) Molecular structures of TPE **1** and TPV **2**. (d) Illustration of the molecular assembly of **1** (left), **2** (right), and **1·2** (middle) into columnar liquid crystals (**1** and **1·2**) and a crystalline lamellar phase (**2**). The columnar assemblies of **1** and **1·2** consists of orthogonal orientation of the molecules with μ_ag_ (purple arrows) parallel to the columnar axis, based on four (**1**) and eight (**1·2**) strands, respectively. H-bonding and slipped π–π interactions of the assemblies are shown as magnifications. Insets show pictures of the thin films of each compound under 360 nm UV irradiation and the emission quantum yield (ϕ).

To date, axial columnar assemblies based on LC dyes have been only reported for dendronized perylene diimides (PBIs),^33–35^ diketopyrrolopyrroles,^36,37^ naphthalene diimides (NDIs),^38^ and aryldipyrrolidones.^39^ The key design strategy for achieving such assemblies lies in incorporating donor–acceptor hydrogen-bonding (H-bonding) groups integrated in the dye scaffolds, facilitating multitopic H-bonds along the plane of the aromatic cores (Figure 1b left). As a result, the chromophores assemble into one-dimensional H-bonded strands, which in turn associate through secondary π–π interactions forming multistranded columnar structures. For example, a series of PBIs, with free NH at the imide positions and four dendrons at the bay positions, that self-assembles into H-bonded columnar J-aggregates was reported.^33–35^ Interestingly, by modulating the substitution pattern of the dendrons, it was possible to control the number of strands in the columnar structure from one to four, which was shown to modulate the absorption properties of the materials.^34^ A similar assembly mode has also been reported for a LC NDI that also forms a J-aggregate,^38^ which confirms that this assembly mode can favor the J-type coupling between the dyes,^30–32,43–45^ which is difficult to achieve in conventional discotics.^2^

This assembly approach has also been investigated using molecules containing two covalently linked chromophores, leading to the formation of core–shell columnar liquid crystals (Figure 1b, right).^46,47^ Würthner and co-workers reported a PBI functionalized with four dendronized oligothiophene moieties that self-assembles into a complex columnar structure comprising two distinct domains: PBI J-aggregates at the column cores, surrounded by oligothiophene units.^46,47^ Notably, the thiophene groups are oriented perpendicular to the columnar axis, while the PBI molecules adopt an unconventional alignment along the axis. This coassembly of two dye components exhibits intriguing Förster resonance energy transfer and photoconductive properties, facilitated by the spatial separation of the two dye domains. Inspired by these investigations, we hypothesized that such nonconventional assemblies could serve as a versatile platform for constructing precision supramolecular coassemblies based on various chromophores.^48–51^ Fine-tuning the self-assembly of binary π-conjugated molecules is key to achieving exciton heterocouplings and unlocking novel light-harvesting and charge-carrier systems with potential for photonic and optoelectronic applications.^21–29^

Herein, we report on the precise coassembly into a complex LC structure of two newly designed fluorescent chromophores, the tris­(*p*-phenyleneethynylene) (TPE) dicarboxylic acid (**1**) and the tris­(*p*-phenylenevinylene) (TPV) bis­(pyridine) (**2**) (Figure 1c), both functionalized with two dendrons in the central phenyl ring. TPE **1** forms through H-bonds a nonconventional four-stranded orthogonal LC columnar phase (Figure 1d, left), while TPV **2** forms a lamellar crystalline phase (Figure 1d, right). Interestingly, the equimolar mixture of **1** and **2** (**1·2**) leads to the formation of an unprecedented two-component columnar liquid crystal constituted by the precise coassembly of the two chromophores by complementary H-bonds between the pyridine and the carboxyl groups (Figure 1d, middle). The resulting columnar coassembly is composed by the stack of eight strands, each of them alternating H-bonded **1** and **2** along the columnar axis and with the transition dipole moments (*μ*_ag_) oriented parallel to the columnar axis (Figure 1d, middle). This is a completely new type of fluorescent columnar liquid crystal that precisely integrates two different chromophores promoting in turn slipped π–π interactions and J-type couplings. This research establishes new approaches to create precision multicomponent assemblies,^48–51^ opening new avenues to develop optoelectronic and photonic materials.

## Results and Discussion

### Molecular Design of Compounds **1** and **2**

In the previous examples, the one-component columnar LC assemblies with the dyes parallel to the columnar axis were achieved exploiting self-complementary H-bonds of imide or lactam units.^33–39^ For the present work, we relied on complementary H-bonding between carboxylic acids and pyridines to build two-component assemblies. H-bonding between carboxylic acids and pyridine was shown to be a robust platform for developing new materials as for example supramolecular liquid crystals and polymeric materials.^52–56^ These H-bonding moieties were incorporated in oligo-*p*-phenyleneethynylene (OPE) and oligo-*p*-phenylenevinylene (OPV) scaffolds, which are very relevant scaffolds in self-assembly and materials science,^57–69^ as for example in optoelectronics. The newly designed TPE **1** and TPV **2** display a bis-3,4,5-trisdodecyloxybenzyl phenyl central core, while they were distinctly functionalized with ethynylene-benzoic acid and vinylene-pyridine scaffolds, respectively (Figure 1c). The structural similarity of TPE **1** and TPV **2** was intentionally designed to increase their compatibility and facilitate their precise coassembly. Both compounds were prepared via well-established synthetic protocols and were obtained as waxy yellowish solids (Supporting Information).

The absorption and emission properties of both compounds were initially studied in CHCl_3_ and THF. Compound **1** presents two intense bands at 321 and 382 nm corresponding to the TPE core (Figure S5). Similarly, TPV **2** presents two absorption bands with maxima at 325 and 388 nm (Figure S6). Compounds **1** and **2** were found to be luminescent in CHCl_3_ or THF (Figures S5–S7 and Table S1) with quantum yields (ϕ) of 0.72 and 0.66, respectively.

### Liquid-Crystalline Behavior

The LC properties of compounds **1** and **2** were initially assessed by polarizing optical microscopy (POM), differential scanning calorimetry (DSC), and X-ray scattering (Figure 2 and Figures S8–S27). It was observed that TPE **1** undergoes gradual decomposition when heated above 197 °C when the sample melts. The thermal behavior of compound **1** is shown in Table 1. According to DSC and POM observations (Figure 2a,b), TPE **1** exhibits on cooling two LC phases from 197 to 163 °C and from 163 to 64 °C. However, the DSC first cooling cycle for this compound was measured starting from 190 °C to avoid decomposition (Figure 2b). The structural features of LC TPE **1** were studied by X-ray scattering at different temperatures. At 100 °C, TPE **1** showed an X-ray pattern consisting of an intense signal at 32.7 Å and six weaker signals along the middle angle region (Figure 2c). This pattern was assigned to a simple columnar rectangular (Col_r_) phase with *a* = 32.7 Å and *b* = 17.4 Å (Col_r2_).^70,71^ TPE **1** shows a second Col_r_ phase (Col_r1_) between 197 and 163 °C (Figures S13 and S14 and Table S2) and a crystalline lamellar (Cr_(Lam)_) phase at room temperature (Figures S17 and S18 and Table S4). Curiously, the Col_r2_ phase exhibits very similar parameters than the Col_r1_ phase, which suggest that the differences between the two phases may be caused by small changes in the molecular arrangements.

**Figure 2:**
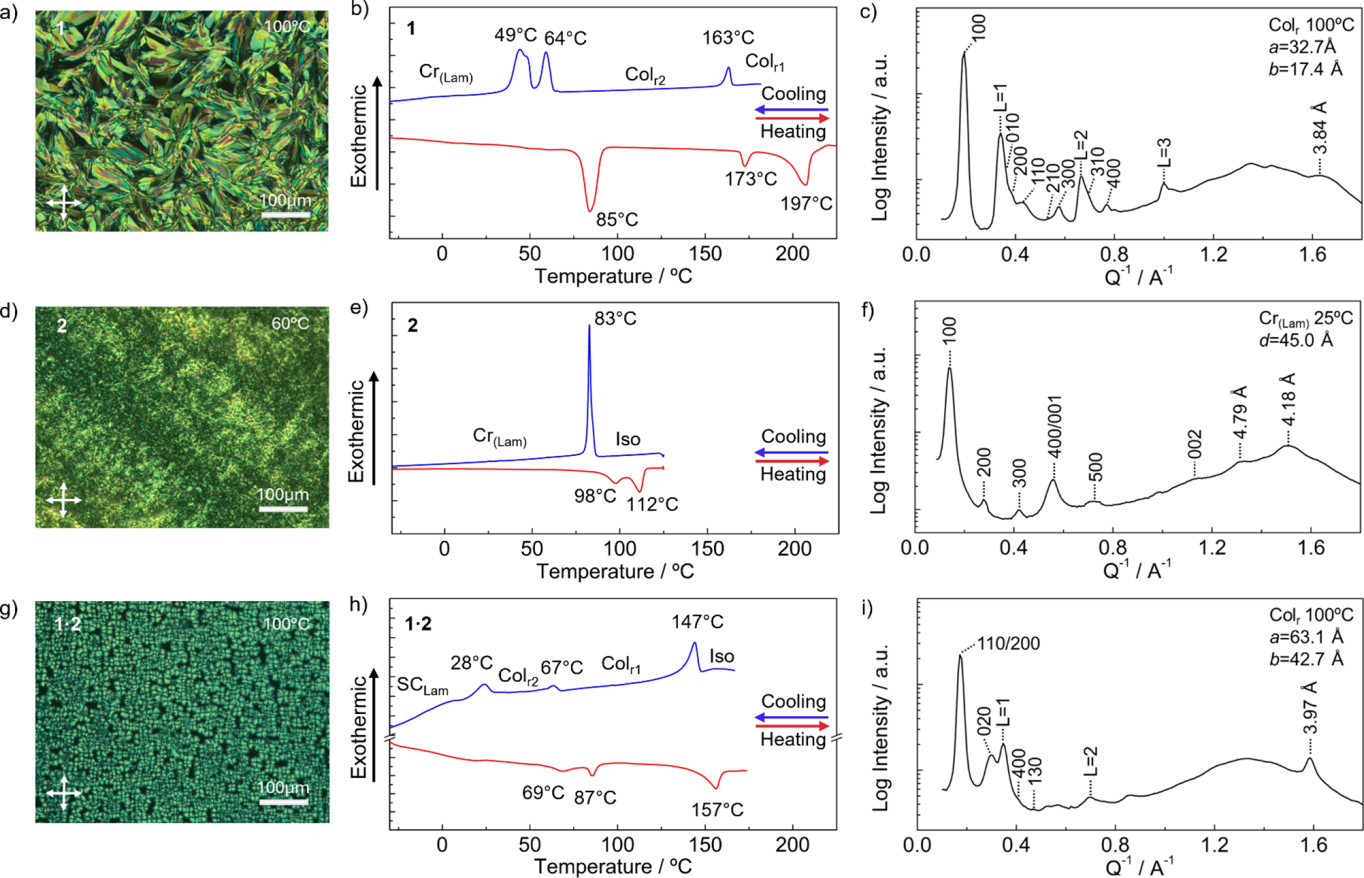
POM images of compounds (a) **1**, (d) **2**, and (g) **1·2** at 100, 60, and 100 °C, respectively. DSC first cooling (blue) and second heating (red) curves for (b) **1**, (e) **2**, and (h) **1·2**. Heating/cooling rate 10 °C min^–1^. X-ray patterns of (c) **1**, (f) **2**, and (i) **1·2** at 100, 25, and 100 °C, respectively. Miller indices, layer lines (L), phases, and lattice parameters are indicated in the inset.

**Table 1:** Thermal Properties of Compounds **1** and **2** and the **1·2** Mixture

compound	phase transition behavior^a^						
**1**	Iso	197	Col_r1_	163	Col_r2_	64/49	Cr_(Lam)_
**2**	Iso	83	Cr_(Lam)_				
**1·2**	Iso	147	Col_r1_	67	Col_r2_	28	SC_Lam_

a Phase transition temperatures (°C) determined by DSC (first cooling, scan rate: 10 °C min^–1^) and supported by polarizing optical microscope observation. Iso: isotropic; Col_r1_, Col_r2_: columnar rectangular; SC_Lam_: soft-crystalline lamellar; Cr: crystal. Compound **1** decomposes gradually when isotropic.

In contrast to TPE **1**, TPV **2** showed only a crystalline phase from −20 to 112 °C when the sample melts (Figure 2d,e). POM images of this phase showed birefringence, but the sample was not fluid. X-ray analysis of the sample at 25 °C confirmed the presence of a crystalline phase with a lamellar organization and an interlayer distance of 45 Å (Figure 2f).

After studying the structure of the single components, mixtures of **1** and **2** were prepared by dissolving 1 equiv of the TPE and 1 equiv of the TPV in CHCl_3_ and evaporating the solvent to obtain a waxy yellowish solid (**1·2**). Pleasingly, temperature-dependent POM observations confirmed the LC characteristics of the sample that showed small focal-conic domains under the crossed polarizers, which is consistent with the formation of a columnar phase (Figure 2g). Remarkably, the DSC of the **1·2** blend showed completely different transitions compared to the single components (Figure 2h). The blend showed three first-order transitions at 28, 67, and 147 °C on the cooling cycle (Table 1). The X-ray pattern of **1·2** at 100 °C showed a broad and strong signal at around 35 Å, which was assigned to the superposition of the 110 and 200 peaks in the small angle region and other weaker peaks at intermediate angles corresponding to a centered Col_r_ (Col_r1_) phase (*a* = 63.1 Å; *b* = 42.7 Å).^70,71^ At lower temperatures, **1·2** exhibits a second Col_r_ phase (Col_r2_, Figures S24 and S25 and Table S7) and a soft-crystalline lamellar phase (20 °C, Figures S26 and S27 and Table S8).^72^ Thus, it is apparent that the equimolar mixture of TPE **1** and TPV **2** indeed forms a well-defined thermotropic liquid crystal with completely different behavior than the single components.

### Anisotropic Experiments

To gain deeper insights into the assembly modes of columnar liquid crystals **1** and **1·2**, a combination of anisotropic experiments using grazing incidence WAXS (GiWAXS), POM, and polarized UV/vis and FT-IR spectroscopy was employed. Mechanical shearing was applied to obtain aligned samples of the LC phases, which is known to result in the columns oriented along the applied force (Figure 3a,b).^1,33–39,71^ The successful homogeneous alignment of the LC samples after shearing was first confirmed by POM (Figure 3c and Figure S37). Figure 3c shows the POM image of a sheared sample of **1·2**, appearing uniformly bright when the polarizer/analyzer was at 45° to the shearing direction (Figure 3c left), but turned dark upon a 45° rotation of the sample holder (Figure 3c right). This behavior was also observed for compound **1** (Figure S37) and confirmed the proper alignment of these columnar phases with the columns oriented along the shearing direction (Figure 3a). Importantly, for both samples, the homogeneous alignment was maintained upon cooling in the crystalline phases and reheating to the columnar phases, which implies that the molecules maintain their orientation in the phase transition processes.

**Figure 3:**
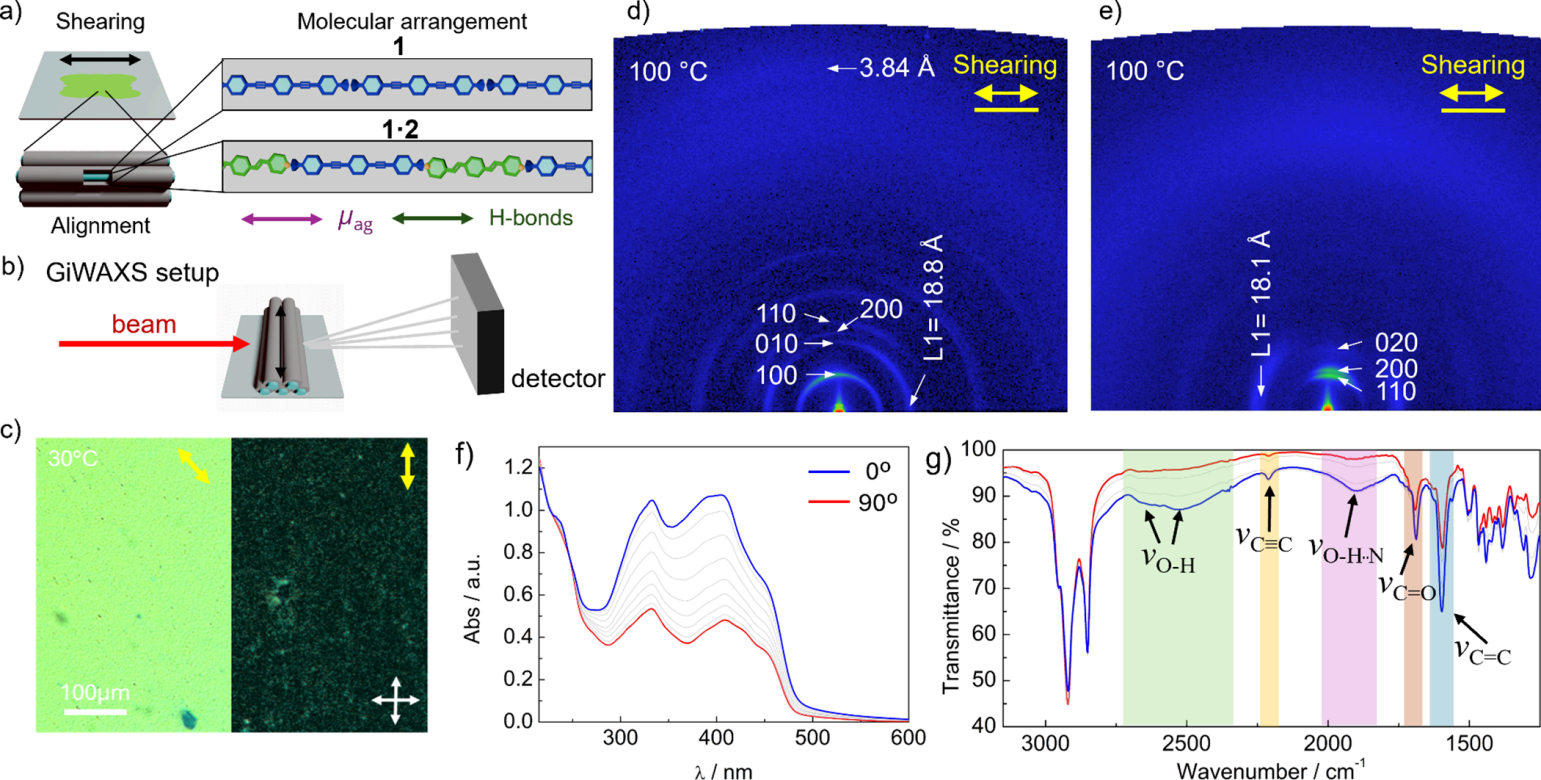
(a) Schematic representation of the shearing induced alignment of liquid crystals **1** and **1·2** and the proposed orientation of the molecules in the columnar assemblies. (b) Illustration of the setup and sample alignment for the GiWAXS experiments. (c) POM images at 30 °C of an aligned sample of **1·2** with the shearing direction (right) parallel to the analyzer and (left) rotated by 45° to the analyzer. The sample was aligned by mechanical shearing at 125 °C. GiWAXS patterns of aligned samples of (d) **1** (100 °C) and (e) **1·2** (100 °C) on silicon plates. The incidence of the X-ray beam and the relative alignment of the sample are shown in Figure 3b. (f) Polarized UV/vis absorption spectra of an aligned thin film of **1·2** on a quartz plate. The spectra were recorded with the polarizer parallel (0°, blue line) and perpendicular (90°, red line) to the shearing direction. (g) Polarized FT-IR spectra of an aligned sample of **1·2** on a KBr plate, with the polarizer parallel (0°, blue line) and perpendicular (90°, red line) to the direction of the alignment.

Figure 3d presents the GiWAXS pattern of a sheared sample of **1** (Figure 3b) on a silicon substrate at 100 °C. The pattern exhibits anisotropy, with distinct sets of signals observed along the vertical (Q_*z*_) and horizontal (Q_*y*_) directions. The reflections appearing along the Q_*z*_ axis matched well with the assignment of the simple Col_r_ phase (Figure 2c). Remarkably, an intense reflection appears along the Q_*y*_ direction at 18.8 Å that matched well with the length of the TPE scaffold and was assigned to layer line 1 (L1) (Figure 3d). Layer lines L2 and L3 were also identified. These observations are consistent with the formation of an unconventional columnar phase with the TPE units oriented along the columnar axis (Figure 1d).^33–39^ In the same line, the GiWAXS pattern of the sheared sample of **1·2** exhibited at 100 °C the 110 and 200 reflections of the Colr_1_ phase along the Q_*z*_ axis, and an intense signal appearing at 18.1 Å on the Q_*y*_ axis (Figure 3e). This distance was assigned to the L1, while the L2 and L3 also appear in the pattern. Again, these observations are indicative of the formation of axial columnar assemblies in **1** and **1·2** (Figure 1d), discarding any conventional packing based on π–π stacking.^1–20^

To confirm the formation of axial columnar phases and to determine the orientation of the molecules in the columnar phases of **1** and **1·2**, we performed polarized spectroscopic experiments. These experiments could only be performed at 25 °C, but the GiWAXS (Figures S39–S43) and POM (Figures S37 and 38) experiments demonstrated that the orientation of the molecules does not change with the temperature. The polarized UV/vis spectra of the sheared sample of **1·2** showed maximum absorption with the polarizer parallel to the shearing direction (Figure 3f), which indicated that the TPE/TPV molecules might be oriented with the long molecular axis (and the transition dipole moment, Figure S48) along the shearing (Figure 3a). The polarized UV/vis (Figure S46) experiments on **1** revealed an analogous behavior, which means that the TPE molecules orient along the shearing direction of the LC phase and subsequently along the columnar axis in the corresponding Colr phases (Figure 1d left).

With the FT-IR experiments, we confirmed the formation of H-bonds and their direction with respect to the shearing. LC **1** shows the two bands at 2673 and 2555 cm^–1^ that were assigned to the O–H stretching of the carboxyl groups and indicates that TPEs are establishing H-bonds between carboxyl pairs (Figure 1d),^73–76^ as previously reported for OPE liquid crystals.^77,78^ These bands appeared polarized along the shearing direction (Figure S44), indicating the parallel direction of the H-bonds to the shearing. A similar behavior was observed for the sheared **1·2** sample, in which the FT-IR pattern also showed the two O–H stretching bands appearing at 2638 and 2497 cm^–1^ (Figure S45). These two stretching bands appeared at different wavenumbers compared to the pure compounds, suggesting that a different H-bonding pattern may be stabilized in the mixture.^73–75^ This observation with the appearance of an additional broad band at 1896 cm^–1^ supported that the pyridines of TPV and the carboxyl groups of the TPE established complementary H-bonds in the **1·2** assembly, in line with previous reports.^73–75^ Polarized FT-IR experiments further revealed that the O–H stretching bands in the aligned **1·2** array are more intense when the polarizer is settled parallel to the shearing direction (Figure 3a,g). These results match well with the previous experiments and support the idea that **1** and **2** interact by complementary H-bonds forming well-defined LC phases and the cores axially oriented in the corresponding columnar phases.

### Absorption/Emission Experiments

For solid-state absorption/emission studies, CHCl_3_ solutions of **1**, **2**, and **1·2** were drop-casted onto quartz plates. The absorption experiments in film state revealed that all compounds exhibited a bathochromic shift in their absorption bands compared to the monomers in solution (Figure 4a,b and Figure S31). These shifts were attributed to a J-type coupling in the assemblies probably due to slipped π–π interactions in the columnar stacks,^43–45^ as supported in the theoretical calculations below (see Figure 5). The TPE **1** and the **1·2** mixture exhibited good and moderate luminescence quantum yields of 63 and 36%, respectively.^79^ In contrast, TPV **2** dropped the emission quantum yield from 66% in solution to 3% in solid state. The absorption/emission properties of all the compounds are compiled in Table S11. As a general trend, it is apparent that the samples containing the TPE **1** exhibited superior emission properties in the solid state.

**Figure 4:**
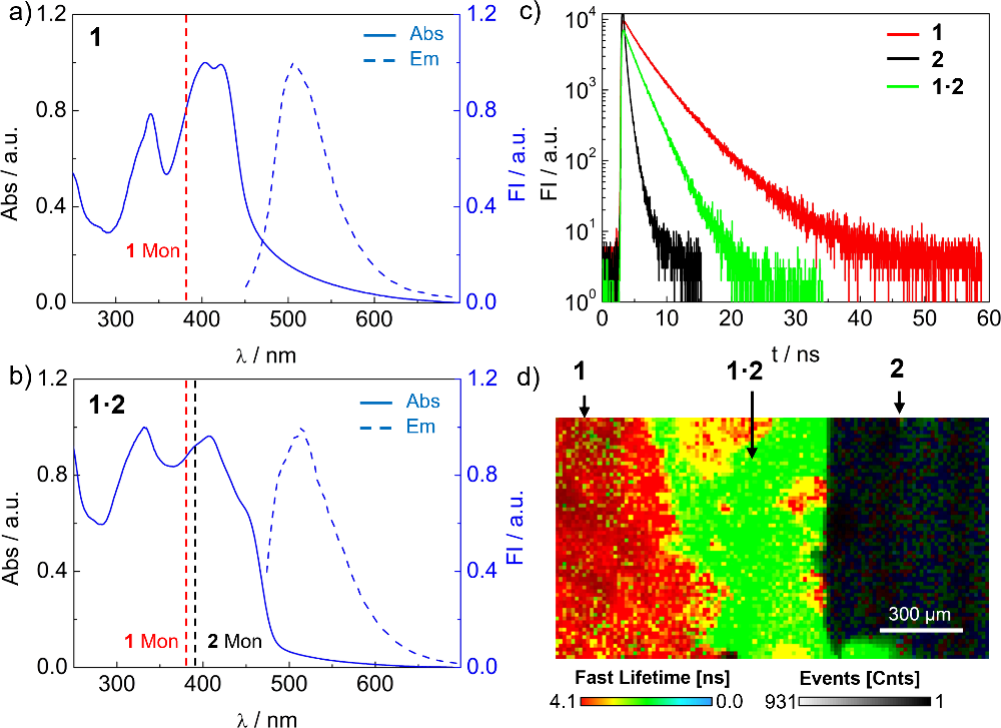
UV/vis (solid lines) and emission (dashed lines) spectra of (a) **1** and (b) **1·2** in solid-state films. Dashed vertical lines indicate the absorption maxima of the monomers of **1** (**1** Mon) and **2** (**2** Mon) in CHCl_3_. (c) Fluorescence decay curves for the solid-state films of **1** (red line), **2** (black line), and **1·2** (green line). Fittings are shown in the Supporting Information (Figures S28–S30). (d) FLIM contact angle experiments on annealed samples of **1** and **2** measured on a quartz plate at 25 °C.

**Figure 5:**
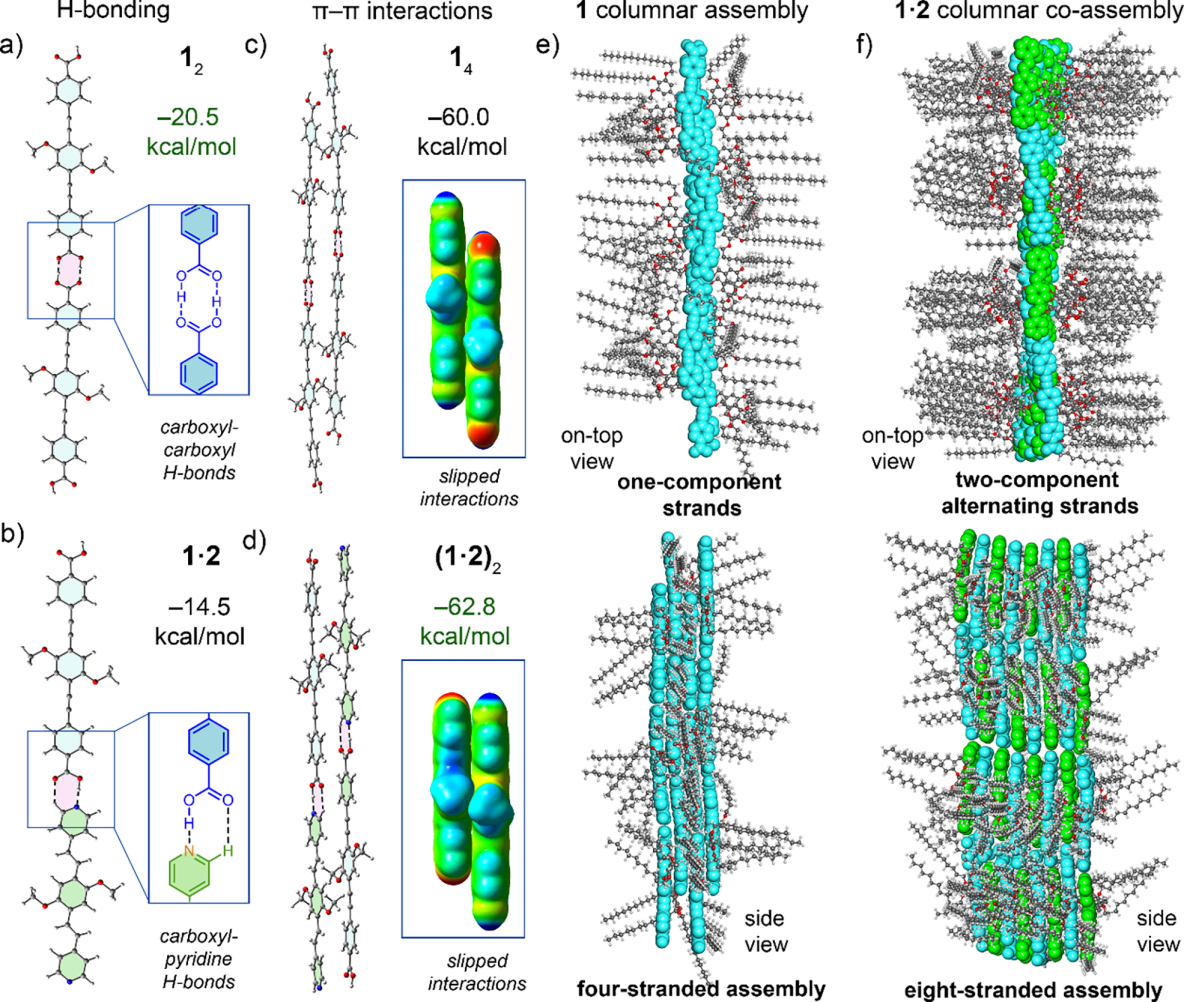
Optimized geometries (RI-BP86-D4/def2-TZVP) of (a) the H-bonded homodimer of **1** and (b) the H-bonded heterodimer of **1·2**. Magnifications show the H-boning patterns. (c) Optimized geometry of the H-bonded and π-stacked homotetramer of **1**. (d) Optimized geometry of the H-bonded and π-stacked heterotetramer of **1·2**. Magnifications show a single slipped π–π interaction for each assembly. (e) On-top and side views of the GFN2-xTB optimized geometry of the assembly model of **1** composed of 12 molecules (3504 atoms). (f) On-top and side views of the assembly model of **1·2** composed of 16 molecules of each coformer (9280 atoms). This was generated by replicating half of this model that was fully optimized at the GFN2-xTB semiempirical level.

Then, we analyzed the emission features of the solid-state samples. Figure 4c shows the emission decay plots for TPE **1**, TPV **2**, and the **1·2** mixture. Compounds **1** and **2** exhibited fluorescence lifetimes (τ) of 5 and >1 ns, respectively (Figures S28 and S29 and Table S11). The low photoluminescence quantum yields and short emission lifetimes for TPV **2** suggest an important contribution of the H-type couplings in the lamellar assembly,^30–32^ while J-coupling contributions are also present. On the other hand, the measurements for the solid-state **1·2**, prepared by evaporation of an equimolar solution of **1** and **2** in CHCl_3_, revealed a single decay process that was fitted to a lifetime of 2 ns (Figure S30 and Table S11). On the other hand, the fluorescence lifetime measurement of the physical mixing of the **1** and **2** powders revealed a decay consisting of two processes (Figure S32), which correspond well to the sum of the decay traces of the individual components. These results confirmed that the **1·2** sample, prepared from the evaporation of the CHCl_3_ solution, behaves like a well-defined system with a characteristic electronic signature, which is in turn consistent with the formation of a precise coassembly of the two components.

Contact angle experiments were also performed by fluorescence lifetime imaging microscopy (FLIM). The experiment was prepared by placing **1** and **2** in their powdered states adjacent to each other on a quartz plate, followed by annealing at 180 °C for 5 min. Figure 4b presents the FLIM image of the resulting array, clearly distinguishing three regions corresponding to pure **1**, pure **2**, and the **1·2** blend in the interphase, with lifetimes matching independent measurements (Table S11). This experiment confirms the compatibility of **1** and **2**, demonstrating their ability to coassemble into the LC **1·2** phase through both solution processing and thermal treatment in bulk.

### Theoretical Modeling of the LC Assemblies

To gain deeper insights into the assembly modes of LC **1** and the **1·2** blend as well as the factors driving the formation of columnar assemblies, we performed theoretical studies. Initially, we modeled the monomers of compounds **1** and **2** (replacing long chains with methyl groups) to investigate and compare the H-bonding patterns in pure **1** and in the **1·2** mixture. The RI-BP86-D4/def2-TZVP-optimized dimer of compound **1** (Supporting Information) revealed that the typical formation of the *R*_2_^2^(8) motif, characterized by two short H-bonds (highlighted in fuchsia, Figure 5a), has an interaction energy of −20.5 kcal/mol and an OH···O distance of 1.53 Å. In the case of the heterodimer **1·2** (Figure 5b), the optimized geometry exhibited an *R*_2_^2^(7) motif with a short OH···N H-bond (1.59 Å) and a longer CH···O contact (2.33 Å).

The dimerization energy of **1·2** (−14.5 kcal/mol) was weaker than that of the homodimer (**1**)_2_. However, H-bonds are not the sole forces governing the assemblies discussed here, as π-stacking plays a critical role. To further investigate, we extended our DFT calculations to tetrameric assemblies combining H-bond and π-stacking interactions (Figure 5c,d). Interestingly, the formation of the heterotetramer (**1·2**)_2_ was found to be 2.8 kcal/mol more favorable than that of the homotetramer (**1**)_4_, indicating the higher stability of the **1·2** blend. In both cases (homo- and heterotetramer), the aromatic rings were arranged in a parallel-displaced fashion, consistent with the formation of J-aggregates.

To simulate the columnar assemblies and validate the experimental findings, we performed additional calculations on larger systems using the extended semiempirical tight-binding model GFN2-xTB.^80^ This model offers the advantage of handling systems with thousands of atoms and incorporates the atomic partial charge-dependent D4 London dispersion model,^81^ enabling accurate treatment of π-stacking interactions. To model the columnar assemblies, we utilized the information obtained from the X-ray experiments for the Col_r_ assemblies of **1** and **1·2**, respectively. In both cases, we know that TPE/TPV molecules are oriented parallel to the columnar axis and are likely forming H-bonds. From the columnar lattices, it was calculated that the Col_r1_ slice (*h* = 18.8 Å) of **1** contains four TPE molecules, while the **1·2** Col_r1_ slice (*h* = 18.1 Å) is composed of eight molecules of TPE/TPV (Supporting Information).

An optimized model of the columnar assembly of **1** is shown in Figure 5e (top and side views). The results reveal that the alkyl chains play a prominent role by limiting the π-stacking to four strands at high temperatures. Specifically, some alkyl chains wrap around the aromatic rings, forming CH···π interactions that hinder further stacking in the π-direction. This is crucial for the formation of the columnar phases at high temperatures where the alkyl chains are more mobile and occupy a larger space around the TPE cores. At room temperature, however, higher ordering of the chains becomes possible, facilitating the formation of a crystalline lamellar phase (Figure S49), as reported in the previously described nondendronized TPEs.^76,77^

In contrast, for **1·2** assembly, the 1D H-bonded alternating strands can aggregate up to eight strands by π-stacking without significant steric hindrance between the alkyl chains. Nevertheless, as eight-strand stack, several alkyl chains in the first and last layers lack sufficient space to remain coplanar with the aromatic cores and instead orient perpendicular to the aromatic systems (Figure 5f), preventing further aggregation in this direction and the formation of lamellar structures. The geometric features of the optimized assemblies (Figure 5) are in good agreement with the experimental data from X-ray diffraction, lending credibility to both the theoretical models and the experimental interpretations. These findings highlight the interplay of hydrogen bonding and π-stacking, dictating the assembly behavior of LC **1** and the energetically favored **1·2** blend. Furthermore, the bulky dendrons are key to prevent the 2D growing of the assemblies, and defining the columnar nanostructures.

Using the optimized structures of the columnar assemblies of **1** and **1·2** (Figure 5e,f), we evaluated their exciton coupling characteristics applying the Kasha’s exciton theory based on the point-dipole approximation excluding vibronic coupling (Supporting Information).^82^ In the four-stranded assembly of **1**, we identified up to eight different types of TPE–TPE couplings, with only one exhibiting an H-type character (Figure S50 and Table S14). The exciton coupling analysis became significantly more complex in the eight-stranded assembly of **1·2**, not only due to the increased number of strands but also because of the presence of two types of homocouplings (**1–1** and **2–2**) and heterocoupling (**1·2**). In total, we considered five **1**–**1**, five **2**–**2**, and four **1·2** couplings, with only two of them displaying H-type characteristics (Figure S51 and Table S15). Despite the complexity, the results clearly indicate a strong influence of J-type couplings in the system, aligning well with the UV–vis experiments. These findings highlight a predominant J-type contribution in the assembly, consistent with the UV–vis results of the solid-state samples (Figure 4a,b).

## Conclusions

In this study, we present two newly designed bis-dendronized chromophores: a tris­(*p*-phenyleneethynylene) dicarboxylic acid (**1**) and a tris­(*p*-phenylenevinylene) bis­(pyridine) (**2**). Individually, these compounds self-assemble in the solid state, forming columnar and lamellar nanostructures, respectively. Remarkably, an unprecedented level of control over self-assembly was achieved by mixing the two components. The equimolar mixture of **1** and **2** undergoes precise coassembly, driven by complementary hydrogen bonding between the carboxylic acid groups of **1** and the pyridine groups of **2**. This interaction leads to the formation of unique 1D strands in which the two chromophores alternate along the strand. Further hierarchical organization of eight of such strands results in the formation of a luminescent columnar liquid-crystalline phase in which the chromophores align with their long axes and transition dipole moments parallel to the columnar axis. This specific orientation facilitates slipped π–π interactions and crucially J-type coupling between the two chromophores. This innovative coassembly strategy marks a significant advancement in the design of multicomponent liquid crystals by seamlessly integrating structurally and functionally distinct chromophores into a unified framework. The results of this study open exciting new avenues for the development of advanced functional materials with precisely tailored optical and electronic properties, offering great potential for applications in optoelectronic and photonic technologies.

## Supplementary Material





## Data Availability

All data that support the findings of this study are included within the article and its Supporting Information and are also available from the authors upon reasonable request.

## Notes

Materials and methods, synthetic protocols, additional UV/vis, luminescence, X-ray, and FT-IR experiments (PDF)
Cartesian coordinates of DFT-optimized compounds (PDF)
