# Supplementary material for: Luminescent Liquid-Crystalline J‑Aggregate Based on a Columnar Axial Coassembly
Source: J Am Chem Soc. 2025 May 28;147(27):23556–65. doi: 10.1021/jacs.5c03166 (PMC12257545; doi:10.1021/jacs.5c03166)
Supplement: Supplementary file 1 [file ja5c03166_si_001.pdf]

Supporting Information
Luminescent Liquid-Crystalline J-Aggregate based on a Columnar Axial Co-Assembly

Llorenç Rubert, Clémence Marre, Pedro Ximenis, Rosa M. Gomila, Antonio Frontera, Bartolome Soberats*

Universitat de les Illes Balears
Department of Chemistry
Cra. Valldemossa, Km. 7.5,
07122, Palma de Mallorca, Spain

Table of contents

1. Materials and methods……………………………………………………………….2
2. Synthetic procedures…………………………………………………………………4
   -Synthesis of compound 4……………………………………………………………4
   -Synthesis of compound 5…………………………………………………………..5
   -Synthesis of compound 1…………………………………………………………..5
   -Synthesis of compound 6…………………………………………………………..8
   -Synthesis of compound 2…………………………………………………………..8
3. Photophysical properties in solution…………………………………………………..11
4. Polarizing optical microscope observations…………………………………………...13
5. Differential scanning calorimetry……………………………...…………………………...14
6. Wide-angle X-ray scattering experiments………………………………………………15
   -TPE 1…………………………………………………………………………………..15
   -TPV 2………………………………………………………………………………….20
   -Mixture 1·2…………………………………………………………………………….22
   -Calculation of the number of molecules per unit cell……………………………………26
7. Photophysical properties in bulk…………………………………………………………27
8. Infrared experiments………………………………………………...…………………...30
9. Anisotropic experiments………………………………………..………………………..31
   -POM of aligned samples…………………………………………………………………31
   - GiWAXS……………………………………………………………………………...32
   - Polarized FT-IR…………………………………………………………………………34
   -Polarized UV/vis…………………………………………………………………………35
10. Theoretical Calculations…………………………………………………………………..36
11. Calculation of exciton coupling…………………………………………………………..38
12. Supplementary references……………………………………………………………………40

1. Materials and methods

Chemicals and Reagents: All chemicals were purchased from Apollo (Tamworth, B77 4DS, UK), Alfa Aesar (Ward Hill, MA, USA), Thermo Fisher (Waltham, MA, USA) or Fluorochem (Hadfield, SK13 1QH, UK), with minimum analytical grade quality and used without further purification unless otherwise stated. All air/humidity-sensitive reactions were carried out under an inert nitrogen/argon atmosphere. The reaction flasks used were oven dried and flushed with nitrogen. Dry solvents were obtained by purification using PureSolv Solvent Purification System.

Nuclear Magnetic Resonance (NMR): ^1H and ^{13}C NMR spectra were recorded at 298 K on Bruker Avance III-HD 300 spectrometer or Bruker AVANCE 600 MHz. Chemical shifts are reported in ppm (δ) and were internally referenced with the solvent peak. Multiplicities for proton signals are abbreviated as s, d, t, q and m for singlet, doublet, triplet, quadruplet and multiplet, respectively.

High resolution mass spectrometry (HRMS): HRMS were recorded using a direct exposure probe (DEP) in electron ionization mode on a Waters QTOF-I quadrupole time-of-flight mass spectrometer.

Polarized optical microscopy (POM): POM images were obtained with an Axio Imager.A2 microscope by Zeiss equiped with Axiocam 208 color camera and a Linkam LTS420 heating stage.

Differential scanning calorimetry (DSC): DSC was performed on a TA Instruments Q2000 under nitrogen atmosphere at a ramp rate of 10°C/min.

X-ray scattering: Wide angle (WAXS) and grazing incidence wide-angle X-ray scattering (GiWAXS) were recorded on a XENOCS XEUSS 3.0. The instrument is equipped with a GeniX 3D Cu microfocus X-ray source ($\lambda = 1.54$ Å; flux = 2×10^8 ph s–1) and a DECTRIS Pilatus3 R 300K silicon pixel detector with 487×619 pixels of 172 ×172 μm in size. WAXS experiments were measured using an extruded fiber of the compound inside a capillary. The WAXS measurements were recorded using a Peltier sample holder at the sample-detector distance of 100 mm. GIWAXS experiments were performed using the dedicated stage equipped with a Peltier. The samples were deposited on a silicon wafer and aligned by shearing using a spatula. The sample to detector distance was set to 100 mm. The scattering patterns were measured with 2 x 300 s exposure times at the selected temperatures. Lattice distances were measured with XSACT and "DataSqueeze" using the powder diffraction line position tool.[1]

Fourier-transform infrared spectroscopy (FT-IR): Solid-state measurements were carried out using a Bruker Tensor 27 with the samples aligned or deposited on a NaBr pellet.

UV/Vis spectroscopy: UV/Vis absorption spectra were recorded on an Agilent Cary 300 Bio and a JASCO V-750 spectrophotometers. JASCO V-750 was equipped with a Peltier device temperature-control unit. The solution measurements were performed in quartz glass cuvettes (Hellma) using spectroscopic grade solvents (ACROS organics). Extinction coefficients were calculated from Lambert-Beer's law. The solid-state experiments were measured with Agilent Cary 5000 UVVis-NIR equipped with Diffuse Reflectance accessory (DRA-2500) using 1.25 mm quartz round plates (Hellma).

Photoluminescence spectroscopy: Emission spectra were recorded on a PicoQuant Fluo Time 300 spectrometer. The solution measurements were performed in quartz glass cuvettes (Hellma) using spectroscopic grade solvents (ACROS organics). The solid measurements were performed on quartz 1 cm x 1 cm x 2 mm plates (Hellma). The quantum yields were calculated by using the absolute method in dedicated integration spheres.[2] Lifetimes were recorded using a 375nm laser. The data was recorded using the Easy Tau software. FLIM measurements were recorded on the same spectrometer connected to an Olimpus microscope. The data was treated with Simpho Time software.

2. Synthetic procedures

The (3,4,5-tridodecyloxy) benzyl bromide **3** was following a previously described methodology.[3]

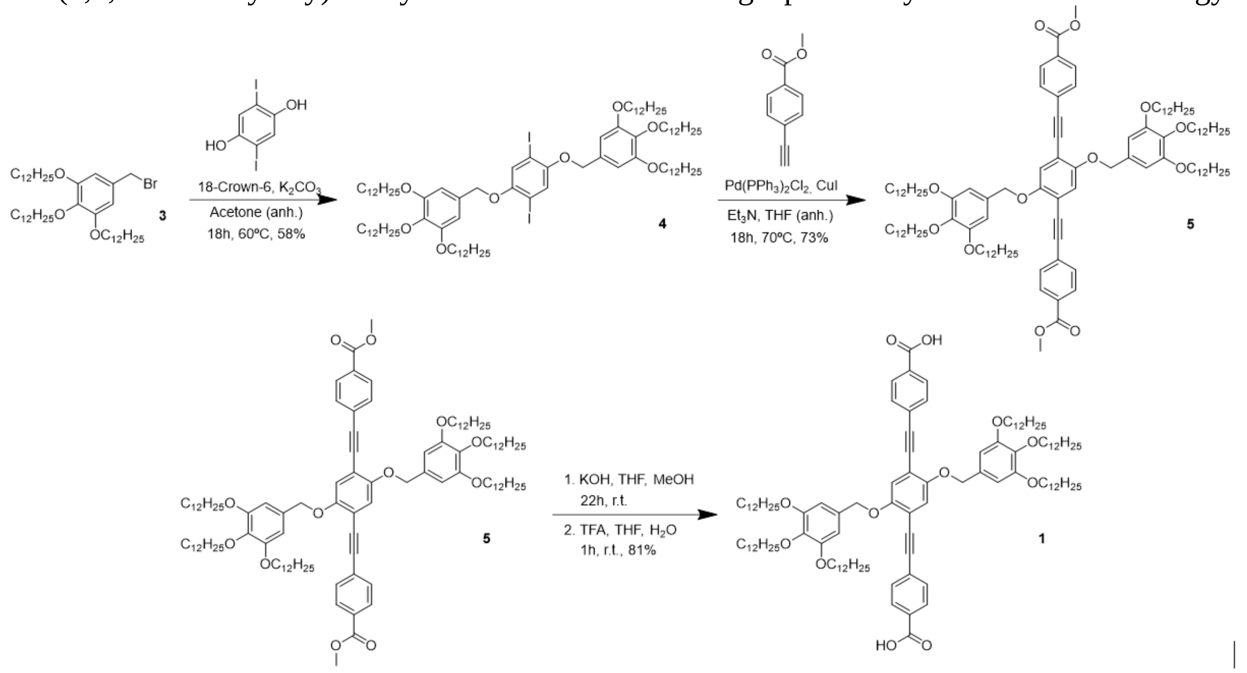

Scheme S1. Synthetic route for the preparation of compound of compound **1.**

*-Synthesis of compound **4***

Compound **3** (1.052 g, 1.450 mmol, 2.5eq), 2,5-diiodobenzene-1,4-diol (0.210 g, 0.580 mmol, 1 eq) and 18-crown-6 (0.038 g, 0.145mmol, 0.25eq) were dissolved in 200 mL of anhydrous acetone in a three-neck round-bottom flask. Then, potassium carbonate (0.481 g, 3.48 mmol, 6eq) was added and the mixture was degassed with three cycles of vacuum and argon. The mixture was stirred for 18 hours at 60°C. After this time, the hot mixture was filtered and the crude product was recrystallized in acetone obtaining a white solid (558 mg, 0.339 mmol, 58%).

^{1}H NMR (300 MHz, $CDCl_3$, rt) δ/ppm: 7.25 (s, 2H, Ar-H), 6.68 (s, 4H, Ar-H), 4.95 (s, 4H, O-CH_2-Ar), 3.99 (t, *J* = 6.46 Hz, 8H, -CH_2-O), 3.94 (t, *J* = 6.66 Hz, 4H, -CH_2-O), 1.84–1.69 (m, 12H, -CH_2-), 1.51-1.41 (m, 12H, -CH_2-), 1.36-1.19 (m, 96H, -$(CH_2)_8$-), 0.87 (t, *J* = 6.86 Hz, 18H, -CH_3)

^{13}C NMR (75 MHz, $CDCl_3$, rt) δ/ppm: 153.39, 152.91, 137.99, 131.25, 123.80, 105.79, 86.76, 73.58, 72.34, 69.30, 32.09, 30.49, 29.91, 29.86, 29.82, 29.54, 26.29, 22.85, 14.27

HRMS-ESI(+): [M+Na^+] calculated m/z for $C_{92}H_{160}I_2O_8Na$, 1670.0123; found, 1670.00948

-Synthesis of compound ***5***

Compound **4** (536 mg, 0.326 mmol, 1eq), methyl 4-ethynylbenzoate (208.2 mg, 1.304 mmol, 4 eq), copper iodide (9.3 mg, 0.099 mmol, 0.3eq) and of bis(triphenylphosphine)palladium(II) dichloride(22.9 mg, 0.033 mmol, 0.1eq) were added in a three-neck round-bottom flask. The mixture was degassed with three cycles of vacuum and argon. 2.5 mL of freshly distilled anhydrous THF and 2.5 mL of freshly distilled anhydrous triethylamine were added. The mixture was stirred for 18 hours under inert atmosphere and protected from light. After this time, the reaction mixture was cooled to room temperature and poured into water. The crude product was purified by silica-gel column chromatography, using as mobile phase a mixture of hexane and ethyl acetate (100:0 → 80:20). Compound **5** was obtained as a yellow, fluorescent powder (406 mg, 0.237 mmol, 73%).

^{1}H NMR (300 MHz, $CDCl_3$, rt) δ/ppm: 8.01 (d, *J* = 8.24 Hz, 4H, Ar-H), 7.55 (d, *J* = 8.91 Hz, 4H, Ar-H), 7.13 (s, 2H, Ar-H), 6.70 (s, 4H, Ar-H), 5.07 (s, 4H, O-CH_2-Ar), 3.97–3.87 (m, 18H, -CH_2-O and O-CH_3), 1.77–1.66 (m, 12H, -CH_2-), 1.50-1.44 (m, 12H, -CH_2-) 1.40-1.21 (m, 96H, -$(CH_2)_8$-), 0.87 (t, *J* = 6.98 Hz, 18H, -CH_3).

^{13}C NMR (75 MHz, $CDCl_3$) δ/ppm: 166.53, 153.91, 153.47, 138.10, 132.62, 131.83, 131.63, 129.72, 127.98, 118.10, 114.74, 105.85, 94.79, 88.81, 73.61, 71.96, 69.35, 52.37, 32.08, 30.51, 29.82, 29.58, 26.28, 22.84, 14.25.

HRMS-ESI(+): [M+Na^{+}] calculated m/z for $C_{114}H_{173}O_{12}Na$, 1734.2922; found, 1734.2928

-Synthesis of compound ***1***

Compound **5** (406 mg, 0.237 mmol, 1 eq) was dissolved in 15.8 mL of THF in a three-neck round-bottom flask. A solution of potassium hydroxide (3.364 mg, 59.961 mmol, 250 eq) in 7.9 mL of methanol was added to the reaction mixture. This mixture was stirred for 22 hours at room temperature. The reaction mixture was acidified with 120 mL of a 2M solution of trifluoroacetic acid in THF (18.36 mL of TFA in 101.64 mL of THF). After 1 hour of stirring at room temperature, the mixture was poured in water, the resulting precipitate was filtered and washed with water. The crude product was treated with boiling water and sonication to remove the undesired salts. The solid was dried and recrystallized in $CHCl_3$ obtaining a yellow fluorescent solid (322 mg, 0.191 mmol, 81%).

^{1}H NMR (300 MHz, $CDCl_3$) δ/ppm : 8.07 (d, *J* = 7.86 Hz, 4H, Ar-H), 7.59 (d, *J* = 7.86 Hz, 4H, Ar-H), 7.15 (s, 2H, Ar-H), 6.71 (s, 4H, Ar-H), 5.08 (s, 4H, O-CH_2-Ar), 3.95 (t, *J* = 6.60 Hz, 4H, -CH_2-O), 3.91 (t, *J* = 6.60 Hz, 8H, -CH_2-O), 1.77-1.67 (m, 12H, -CH_2-),1.41-1.19 (m, 108H, - $(CH_2)_8$-), 0.87 (t, *J* = 6.22 Hz, 18H, -CH_3).

^{13}C NMR (75 MHz, THF-d8) δ/ppm: 167.09, 154.98, 154.48, 139.31, 133.19, 132.28, 131.76, 130.77, 128.74, 118.57, 115.51, 106.74, 95.34, 89.70, 73.74, 72.29, 69.95, 33.07, 30.92, 30.83, 30.68, 30.61, 30.52, 27.33, 23.75, 14.61. The spectrum was recorded in THF due to the limited solubility in $CDCl_3$.

HRMS-ESI(-): [$M-2H^+$] calculated m/z for $C_{110}H_{168}O_{12}$, 840.62734; found, 840.62998

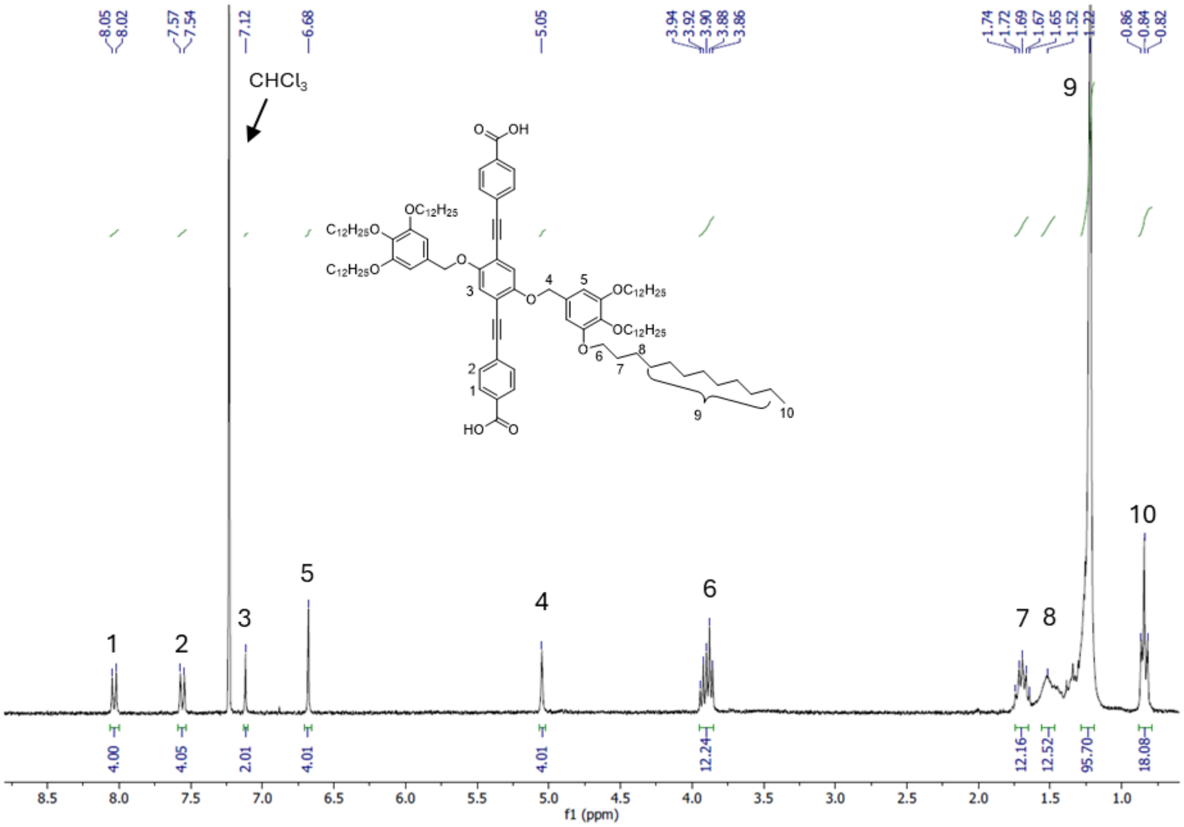

Figure S1. ^{1}H NMR (300 MHz, $CDCl_3$) of **1**.

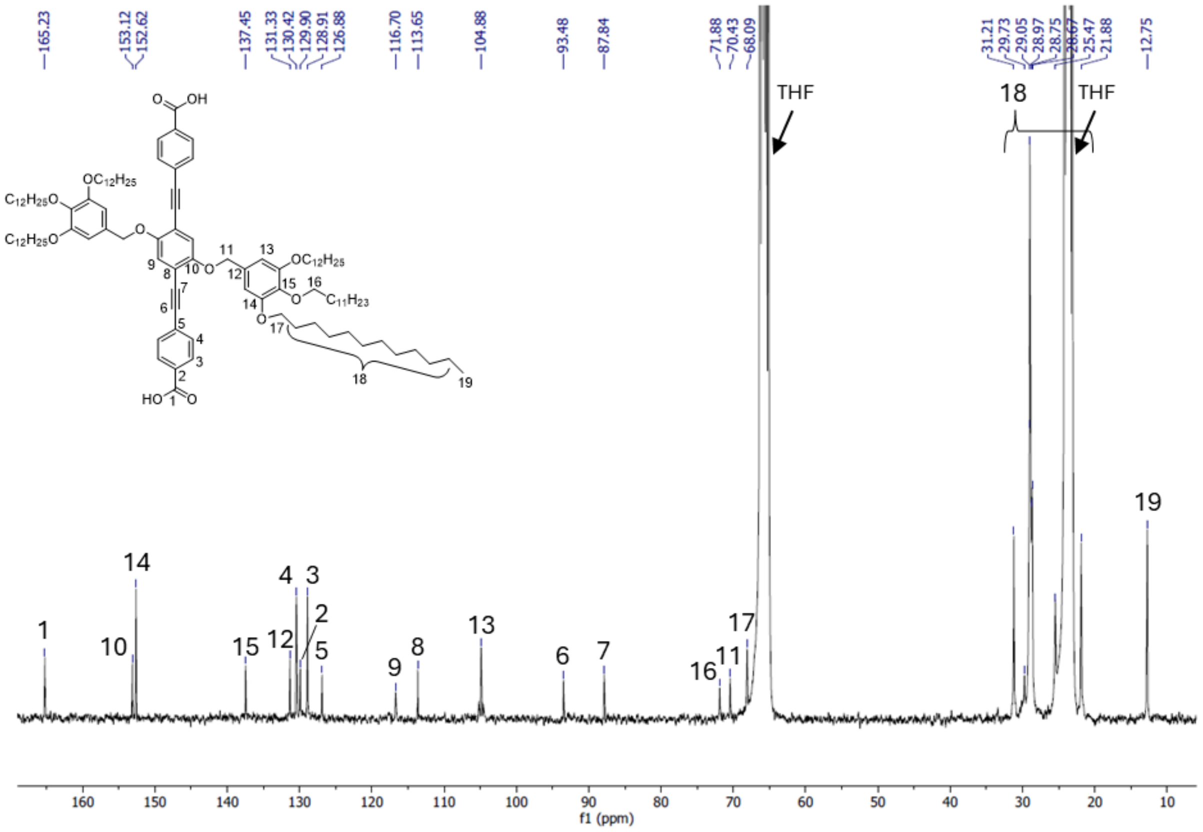

Figure S2. ^{13}C NMR (75 MHz, THF-d8) of **1**.

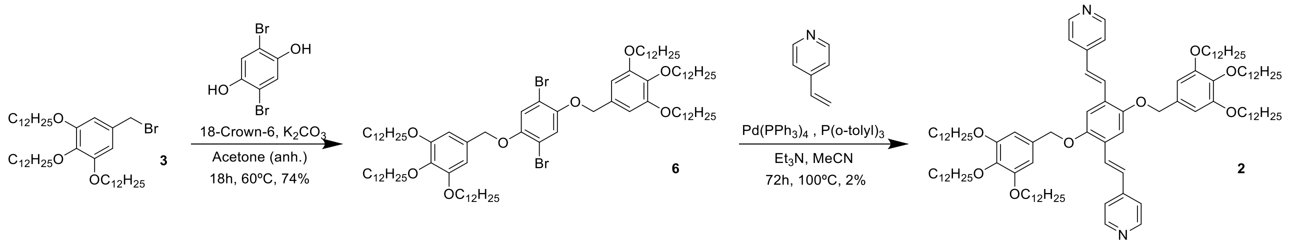

Scheme S2. Synthetic route for the preparation of compound of compound **2.**

*-Synthesis of compound **6***

Compound **3** (4.172 g, 5.80 mmol, 2.5eq), 2,5-dibromobenzene-1,4-diol (0.622 g, 2.32 mmol, 1eq) and 18-crown-6 (0.153 g, 0.58mmol, 0.25eq) were dissolved in 800 mL of anhydrous acetone in a three-neck round-bottom flask. Then, potassium carbonate (1.924 g, 13.92 mmol, 6eq) was added and the mixture was degassed with three cycles of vacuum and argon. The mixture was stirred for 18 hours at 60°C. After this time, the hot mixture was filtered and washed with hot acetone and the crude product was recrystallized in acetone obtaining a white solid (2.656 g, 1.709 mmol, 74%).

^{1}H NMR (300 MHz, $CDCl_3$, rt) δ/ppm: 7.18 (s, 2H, Ar-H), 6.66 (s, 4H, Ar-H), 4.98 (s, 4H, O-CH_2-Ar), 3.98 (t, *J* = 6.49 Hz, 8H, -CH_2-O), 3.96 (t, *J* = 6.62 Hz, 4H, -CH_2-O), 1.85–1.71 (m, 12H, -CH_2-), 1.53-1.43 (m, 12H, -CH_2-), 1.38-1.21 (m, 96H, -$(CH_2)_8$-), 0.89 (t, *J* = 6.91 Hz, 18H, -CH_3)

^{13}C NMR (300 MHz, $CDCl_3$, rt) δ/ppm: 153.41, 150.19, 138.10, 131.22, 119.54, 111.76, 105.85, 73.56, 72.40, 69.26, 32.08, 30.48, 29.90, 29.85, 29.81, 29.58, 29.53, 26.27, 22.84, 14.25

HRMS-ESI(+): [M+Na^+] calculated m/z for $C_{92}H_{160}O_8Br_2Na$, 1574.03722; found, 1574.0397

*-Synthesis of compound **2***

Compound **6** (2.626 g, 1.69 mmol, 1eq), tri-o-tolylphosphine (67 mg, 0.22 mmol, 13%) and tetrakis(triphenylphosphine)palladium(0) (115 mg, 0.995 mmol, 6%) were added in a three-neck round-bottom flask and the mixture was degassed with three cycles of vacuum and argon. In another round bottom flask, vinyl pyridine (1.82 mL, 16.9 mmol, 10eq), 2.5 mL of triethylamine and 17.5 mL of anhydrous acetonitrile were degassed and then added to the main round bottom flask. The reaction mixture was degassed with three cycles of vacuum and argon and was stirred for three days at 100°C. The crude product was purified by silica-gel column chromatography, using as mobile phase a mixture of hexane and ethyl acetate (100:0 → 50:50). The product was obtained as a yellow powder after recrystallization in acetone (45 mg, 0.278 mmol, 2%).

^{1}H NMR (600 MHz, $CDCl_3$) δ/ppm: 8.56 (d, *J* = 5.89 Hz, 4H, Pyr), 7.68 (d, *J* = 16.36 Hz, 2H, =CH-Ar), 7.32 (d, *J* = 5.89 Hz, 4H, Pyr), 7.21 (s, 2H, Ar), 7.04 (d, *J* = 16.58 Hz, 2H, =CH-Pyr), 6.67 (s, 4H, Ar), 5.07 (s, 4H, O-CH_2-Ar), 3.97 (t, *J* = 6.44 Hz, 4H, -CH_2-O), 3.96 (t, *J* = 6.44 Hz, 8H, -CH_2-O), 1.82-1.71 (m, 12H, -CH_2-), 1.46-1.39 (m, 12H, -CH_2-), 1.36-1.23 (m, 96H, -$(CH_2)_8$-), 0.87 (t, *J* = 6.77 Hz, 18H, -CH_3)

^{13}C NMR (75 MHz, $CDCl_3$) δ/ppm: 153.56, 151.62, 150.37, 144.96, 131.97, 127.79, 127.30, 120.95, 112.22, 106.14, 73.65, 72.33, 69.38, 32.08, 31.08, 30.53, 29.92, 29.86, 29.81, 29.58, 26.30, 22.85, 14.27

HRMS-ESI(+): [M+2H$^+$] calculated m/z for $C_{106}H_{174}O_8N_2$, 801.66296; found, 801.6628

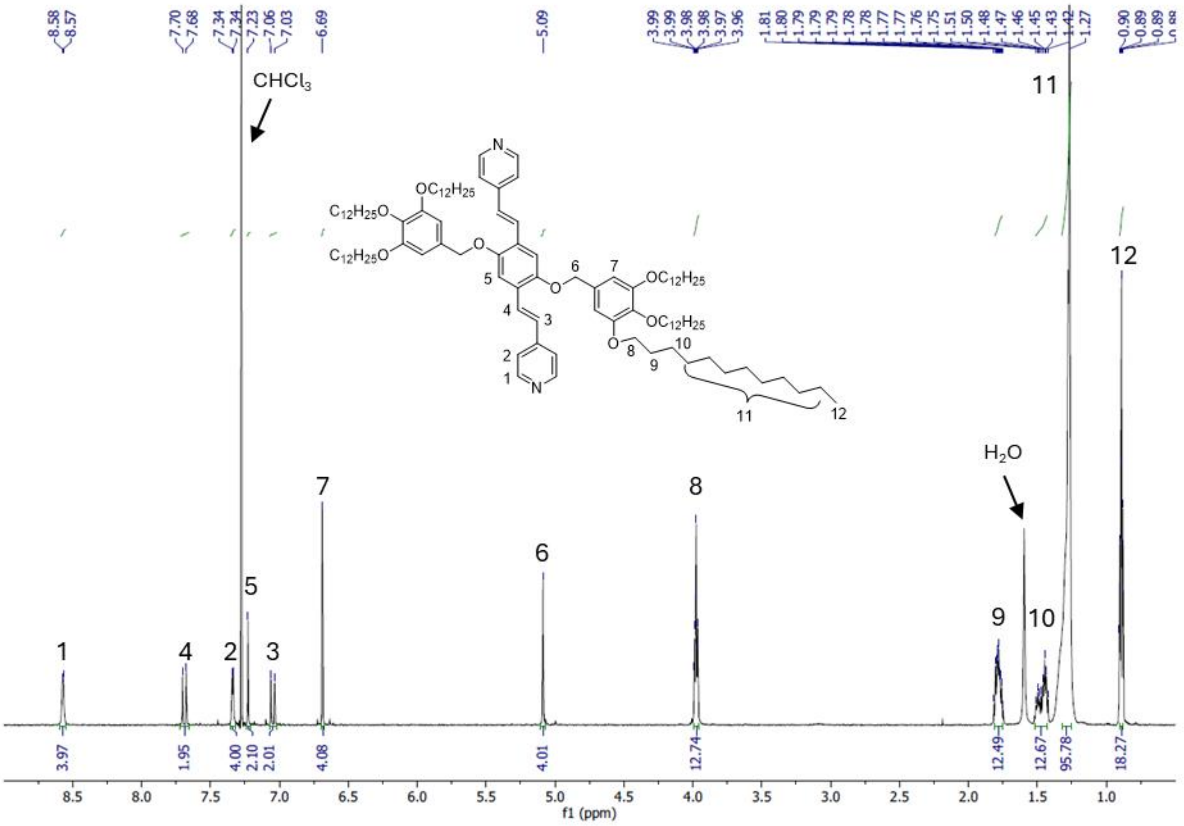

Figure S3. ^{1}H NMR (600 MHz, $CDCl_3$) of **2**.

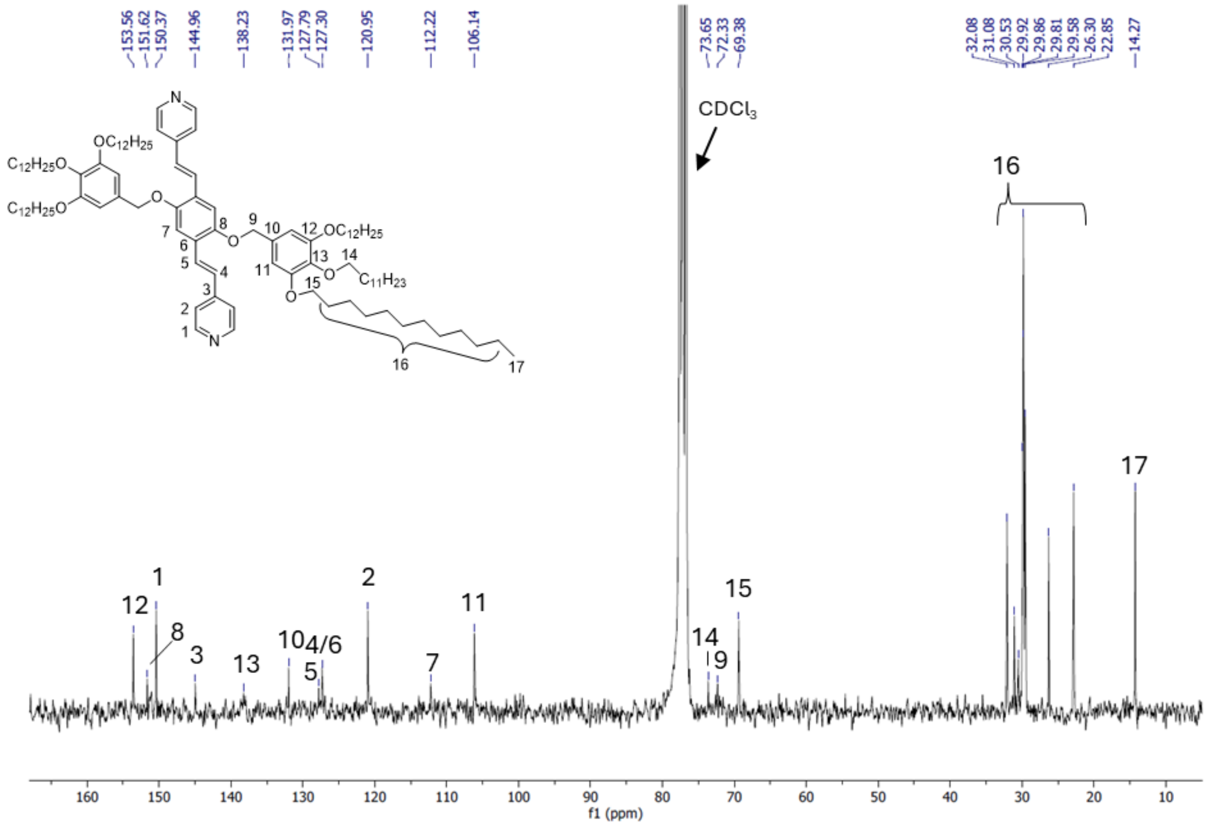

Figure S4. ^{13}C NMR (75 MHz, $CDCl_3$) of **2**.

3. Photophysical properties in solution

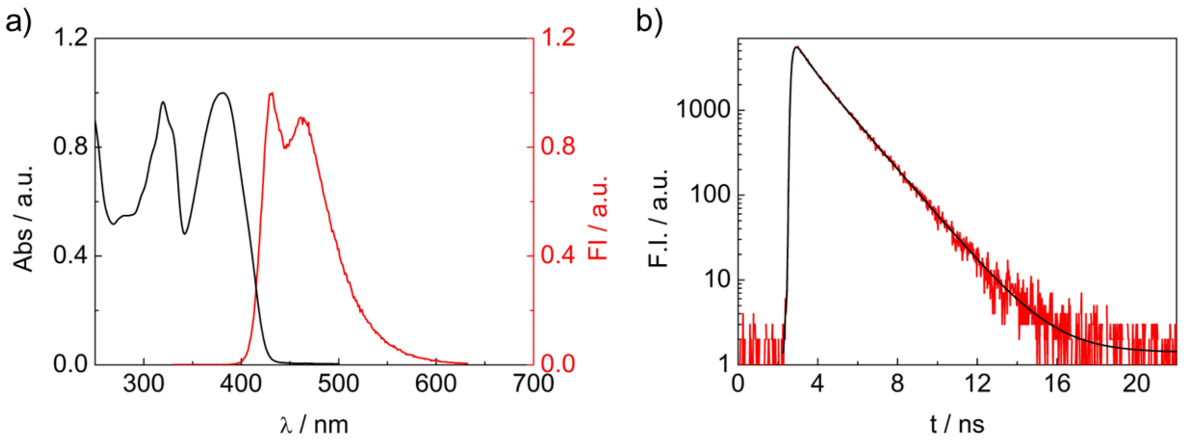

Figure S5. a) Absorption (1 × 10^{-5} M, $CHCl_3$, black line) and emission (1 × 10^{-6} M, $CHCl_3$, red line) (λ_{ex}=382 nm) spectra of compound **1**. b) The fluorescence decay curve (red) and fitting curve (black) of **1** (1 × 10^{-6} M, $CHCl_3$).

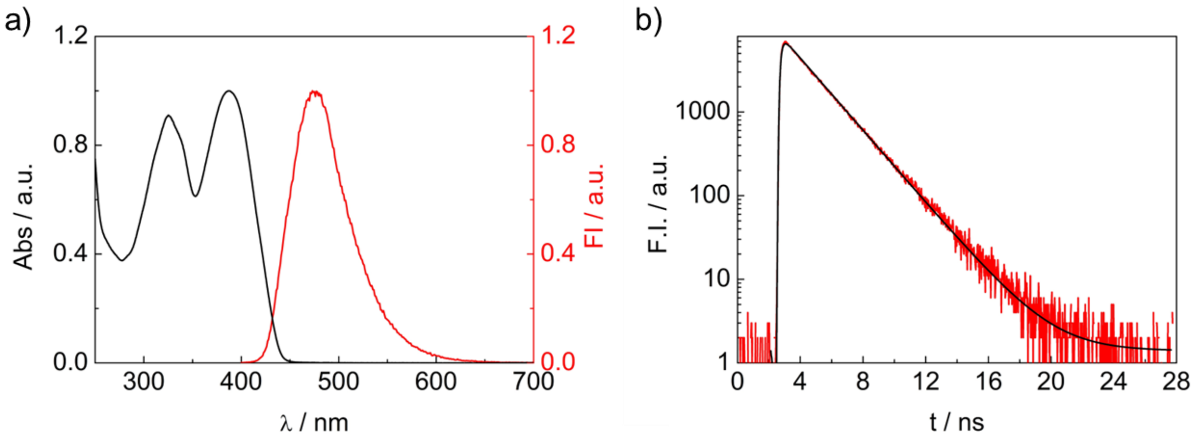

Figure S6. a) Absorption (1 × 10^{-5} M, THF, black line) and emission (1 × 10^{-6} M, THF, red line) (λ_{ex}=388 nm) spectra of compound **2**. b) The fluorescence decay curve (red) and fitting curve (black) of **2** (1 × 10^{-6} M, THF).

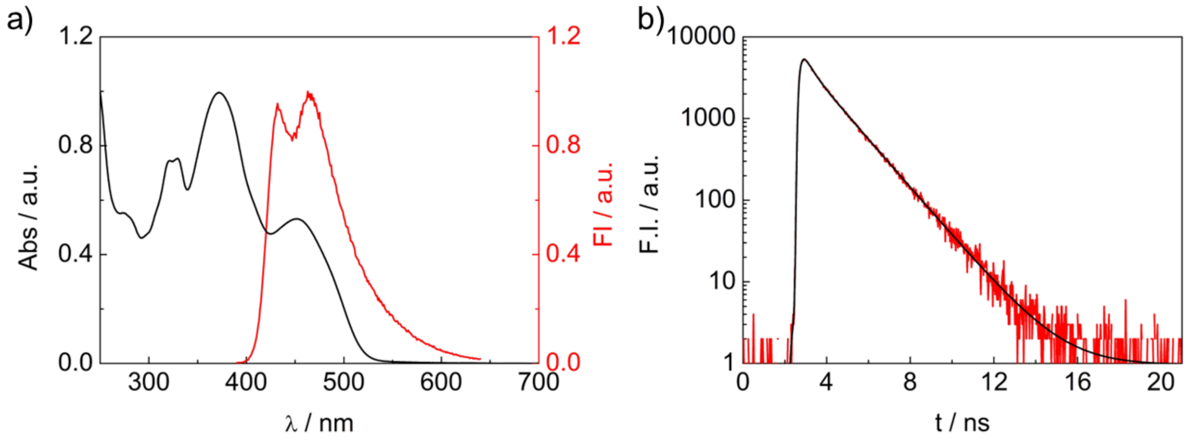

Figure S7. a) Absorption (1×10^{-5} M, $CHCl_3$, black line) and emission (1×10^{-6} M, $CHCl_3$, red line) (λ_{ex}=375 nm) spectra of compound **1·2**. b) The fluorescence decay curve (red) and fitting curve (black) of **1·2** (1×10^{-6} M, $CHCl_3$).

Note: The mixture of **1** and **2** in $CHCl_3$ does not lead to the simple monomeric species. The band appearing at 455 nm (Figure S7a) suggests that proton transfer process may take place.

Table S1. Absorption and emission maxima, quantum yields (calculated by the absolute method)[2] and fluorescence lifetimes for **1**, **2** and **1+2** in solution.

	Abs			Em				
**Sample**	**λ_1(nm)**	**λ_2(nm)**	**λ_3(nm)**	**λ_1(nm)**	**λ_2(nm)**	**ϕ**	**τ_1(ns)**	**τ_2(ns)**
**1** ($CHCl_3$)	321	382	-	431	464	0.72	1.585	0.390
**2** (THF)	325	388	-	476	-	0.66	2.013	-
**1+2** ($CHCl_3$)	331	375	455	432	465	0.31	1.471	0.327

4. Polarizing optical microscope observations

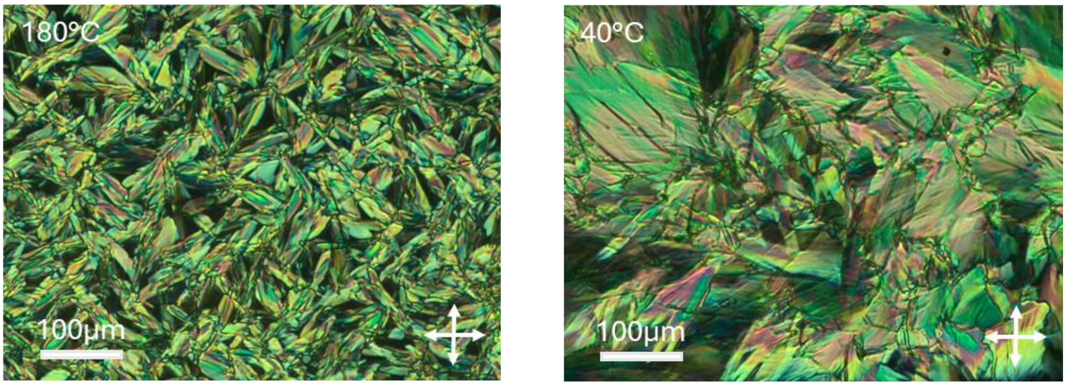

Figure S8. POM images of **1** at 180 ºC (left) and 40 ºC (right).

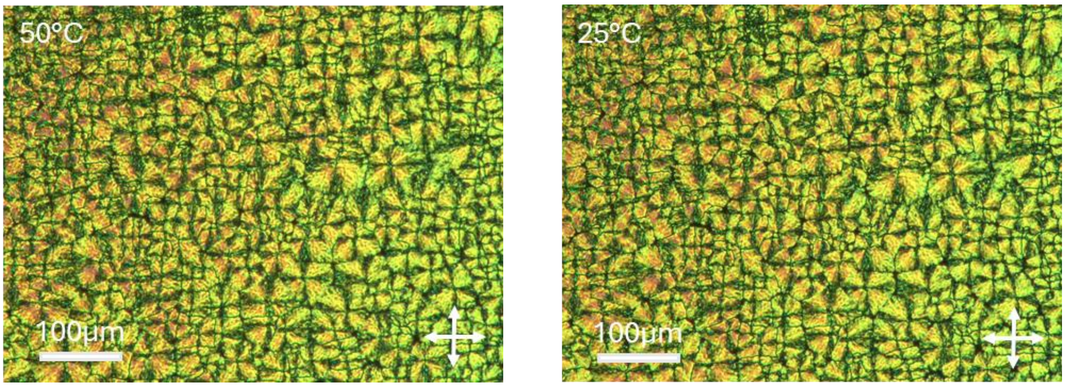

Figure S9. POM images of **1·2** at 50 ºC (left) and 25 ºC (right). The sample was obtained by evaporating and drying an equimolar solution of **1** and **2** in $CHCl_3$.

5. Differential scanning calorimetry

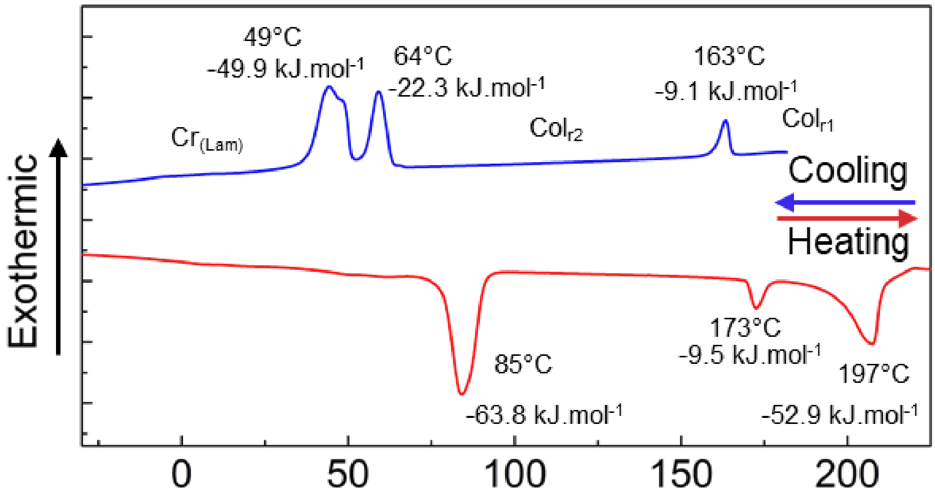

Figure S10. DSC first cooling (blue) and second heating (red) curves for **1**. Heating/ Cooling rate 10 °C/min.

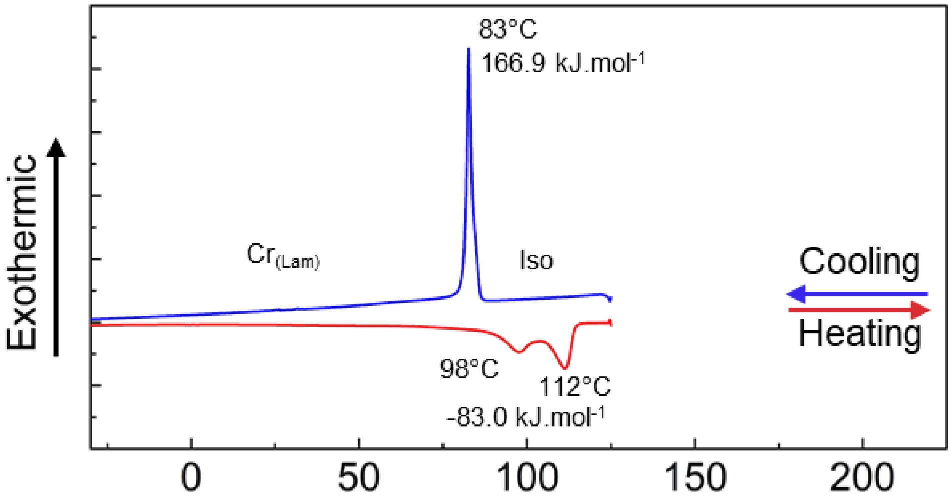

Figure S11. DSC first cooling (blue) and second heating (red) curves for **2**. Heating/ Cooling rate 10 °C/min.

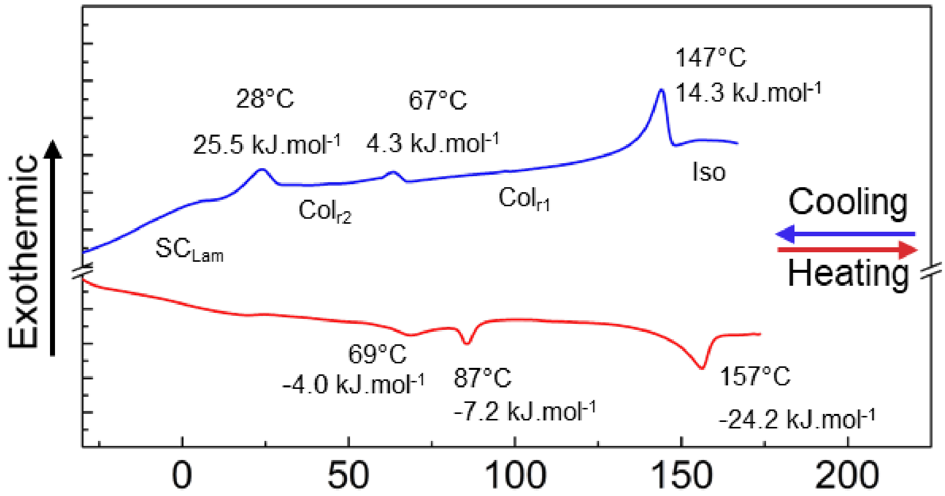

Figure S12. DSC first cooling (blue) and second heating (red) curves for **1·2**. Heating/ Cooling rate 10 °C/min.

6. Wide-angle X-ray scattering experiments

*-TPE **1***

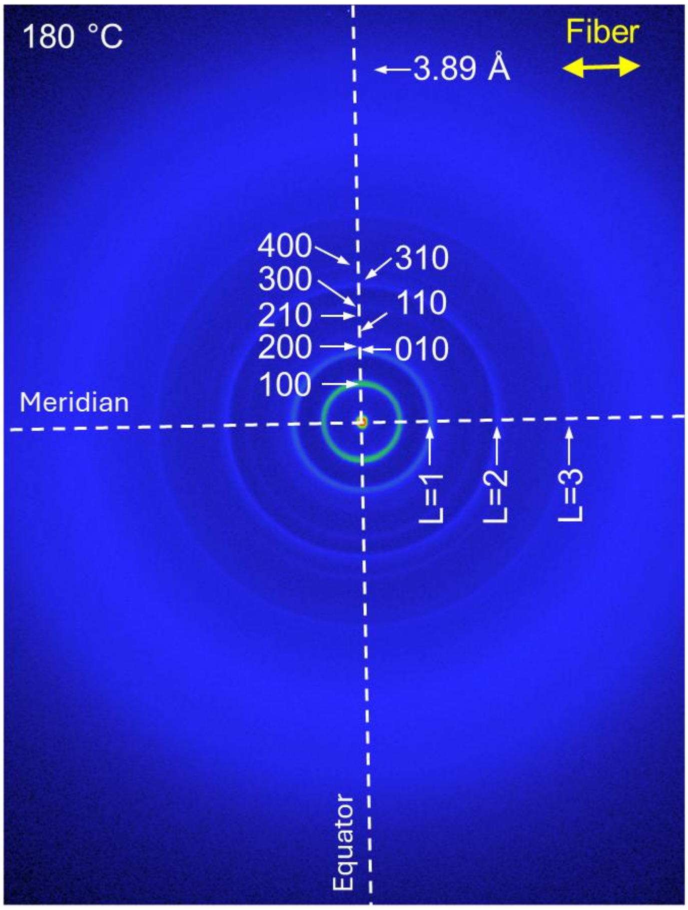

Figure S13. 2D WAXS pattern of a partially aligned fiber of **1** at 180 °C.

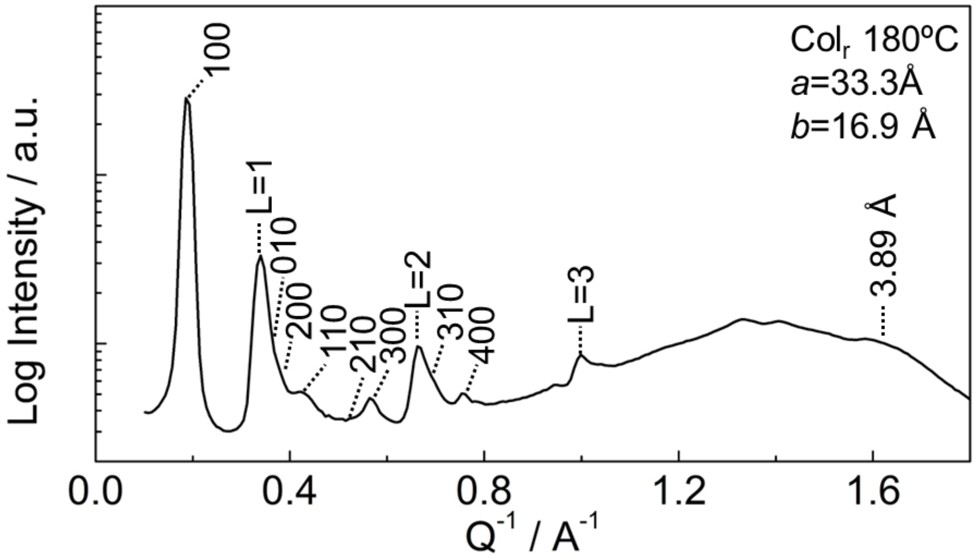

Figure S14. Integrated intensities of the WAXS pattern of **1** at 180 °C, showing the reflections indexed according to a simple columnar rectangular phase.

Table S2. Data of the WAXS pattern of **1** at 180 °C compared to the theoretical columnar rectangular geometry reflections.

Entry	*hkl*	d_{exp} / Å	d_{calc} / Å	Δ / Å
1	100	33.3	33.3	0.0
2	010	16.9	16.9	0.0
3	200	16.6	16.6	0.0
4	110	14.9	15.1	0.2
5	210	11.9	11.9	0.0
6	300	11.1	11.1	0.0
7	310	9.1	9.3	0.2
8	400	8.3	8.2	0.1
	L=1	18.8		
	L=2	9.4		
	L=3	6.3		

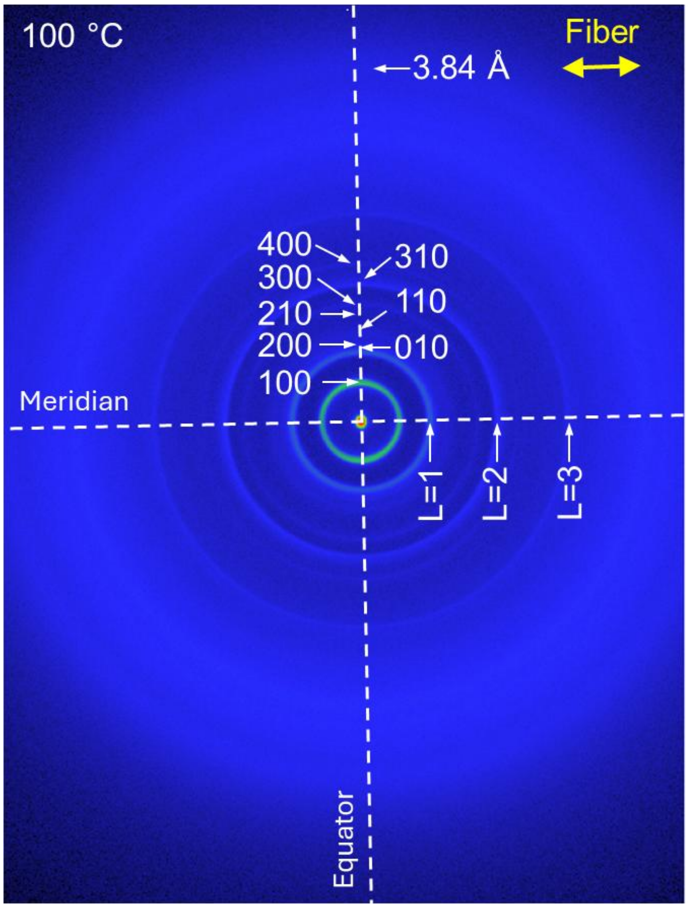

Figure S15. 2D WAXS pattern of a partially aligned fiber of **1** at 100 °C.

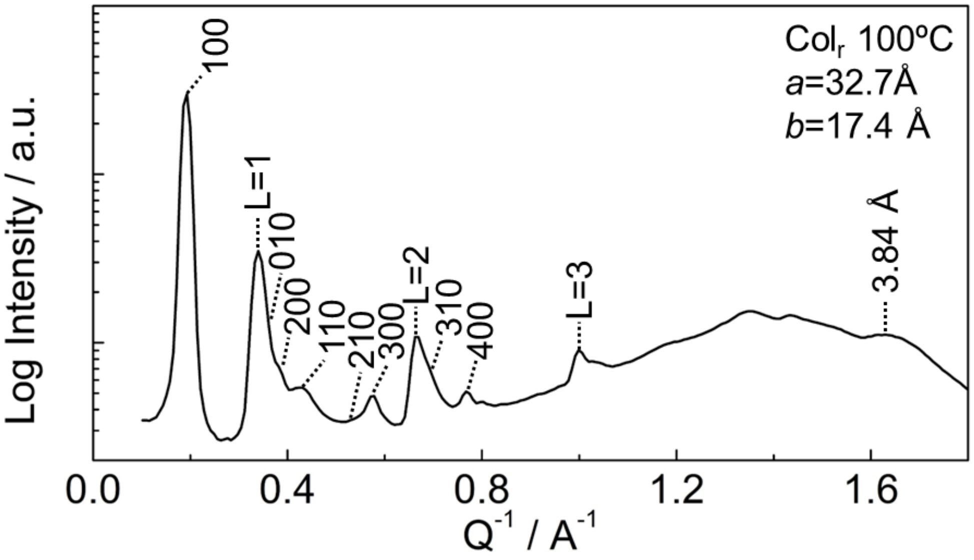

Figure S16. Integrated intensities of the WAXS pattern of **1** at 100 °C, showing the reflections indexed according to the simple columnar rectangular phase.

Table S3. Data of the WAXS pattern of **1** at 100 °C compared to the theoretical columnar rectangular geometry reflections.

Entry	hkl	d_{exp} / Å	d_{calc} / Å	Δ / Å
1	100	32.7	32.7	0.0
2	010	17.1	17.1	0.0
3	200	16.4	16.3	0.1
4	110	15.0	15.2	0.2
5	210	11.7	11.8	0.1
6	300	10.9	10.9	0.0
7	310	9.2	9.2	0.0
8	400	8.2	8.2	0.0
	L=1	18.8		
	L=2	9.4		
	L=3	6.3		

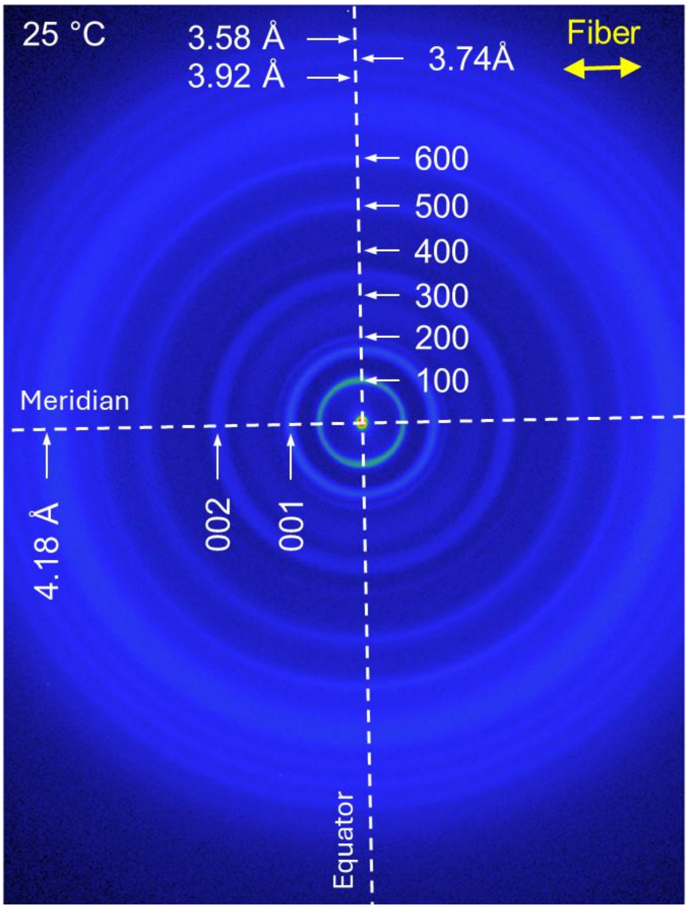

Figure S17. 2D WAXS pattern of a partially aligned fiber of **1** at 25 °C.

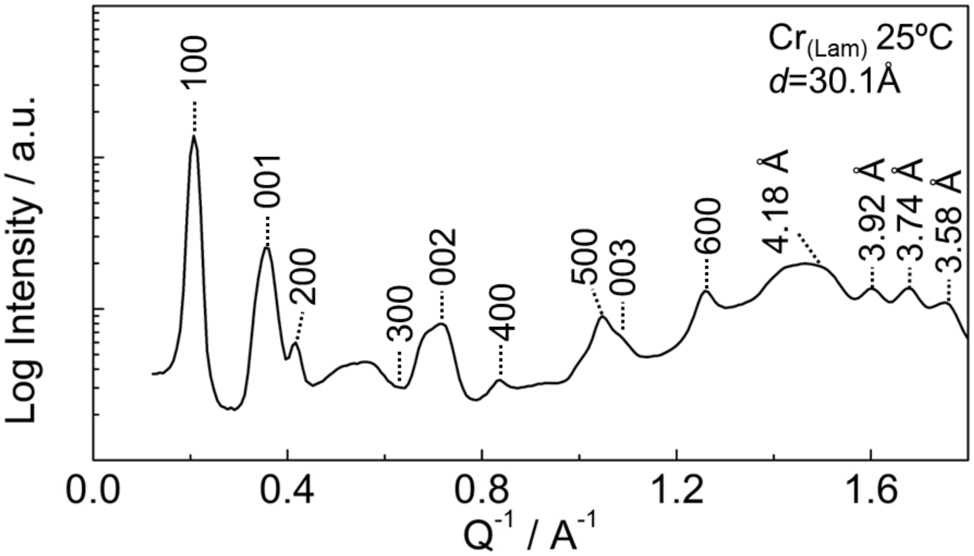

Figure S18. Integrated intensities of the WAXS pattern of **1** at 25°C, showing the reflections indexed according to the lamellar crystalline phase.

Table S4. Data of the WAXS pattern of **1** at 25 °C compared to the theoretical lamellar geometry reflections.

Entry	hkl	d_{exp} / Å	d_{calc} / Å	Δ / Å
1	100	30.1	30.1	0.0
2	200	15.1	15.1	0.0
3	300	10.0	10.0	0.0
4	400	7.5	7.5	0.0
5	500	6.0	6.0	0.0
6	600	5.0	5.0	0.0
7	001	17.4	17.4	0.0
8	002	8.7	8.7	0.0
9	003	5.8	5.8	0.0

-TPV **2**

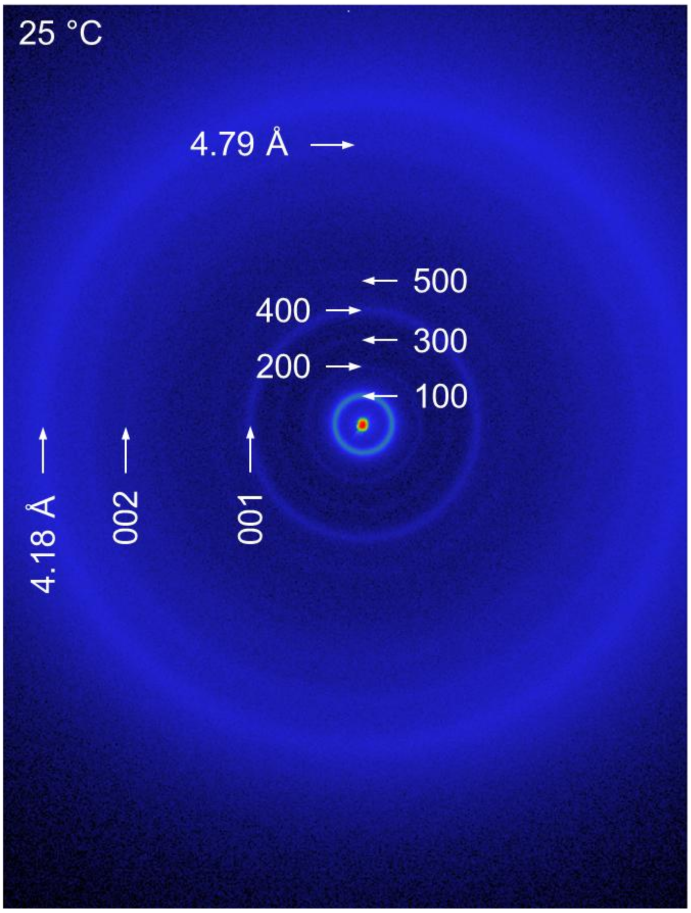

Figure S19. 2D WAXS pattern of bulk **2** at 25 °C.

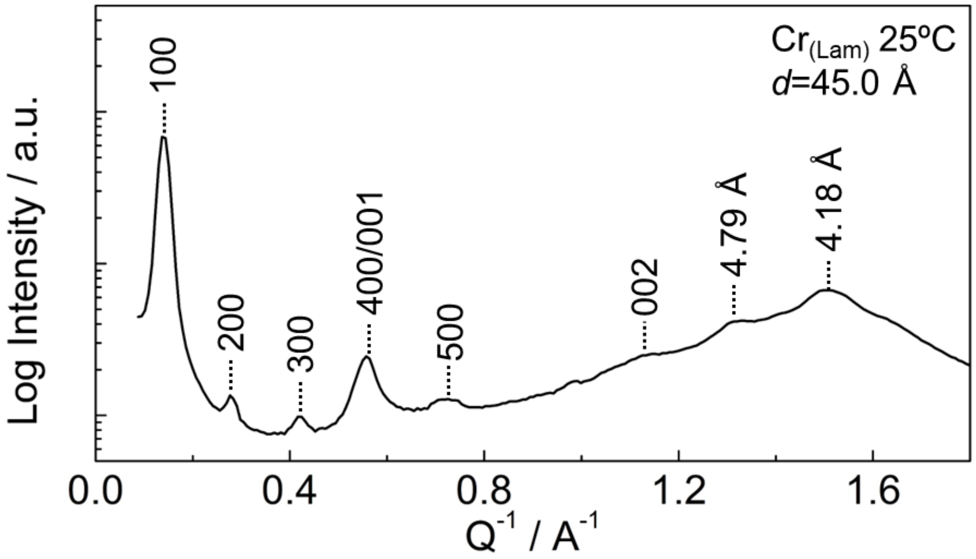

Figure S20. Integrated intensities of the WAXS pattern of **2** at 25 °C, showing the reflections indexed according to the lamellar crystalline phase.

Table S21. Data of the WAXS pattern of **2** at 25 °C compared to the theoretical lamellar geometry reflections.

Entry	hkl	d_{exp} / Å	d_{calc} / Å	Δ / Å
1	100	45.0	45.0	0.0
2	200	22.5	22.5	0.0
3	300	15.0	15.0	0.0
4	400	11.3	11.3	0.0
5	500	8.7	9.0	0.3
6	001	11.3	11.3	0.0
7	002	5.7	5.7	0.0

*-Mixture **1·2***

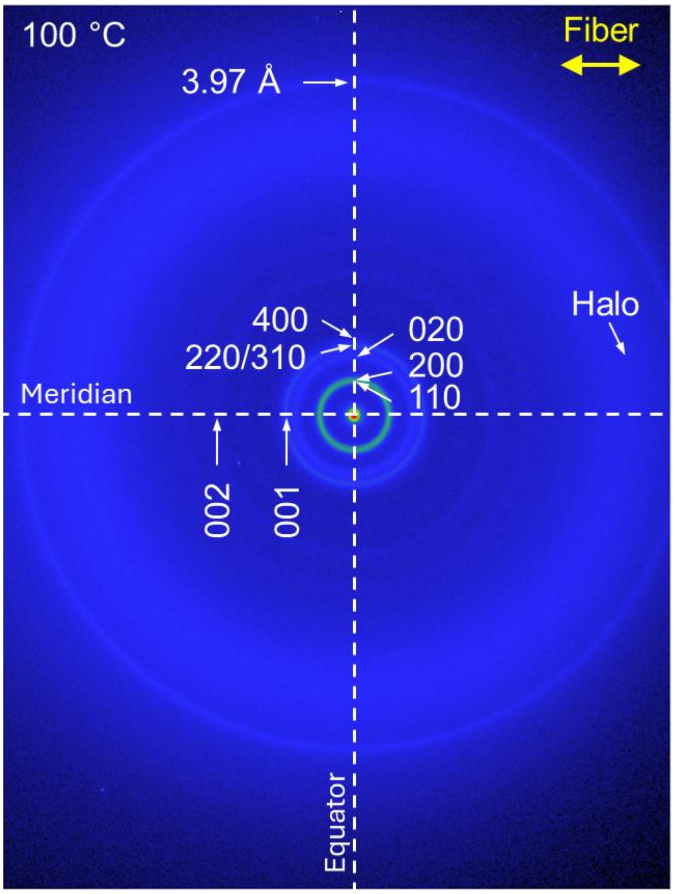

Figure S22. 2D WAXS pattern of a partially aligned fiber of **1·2** at 100 °C.

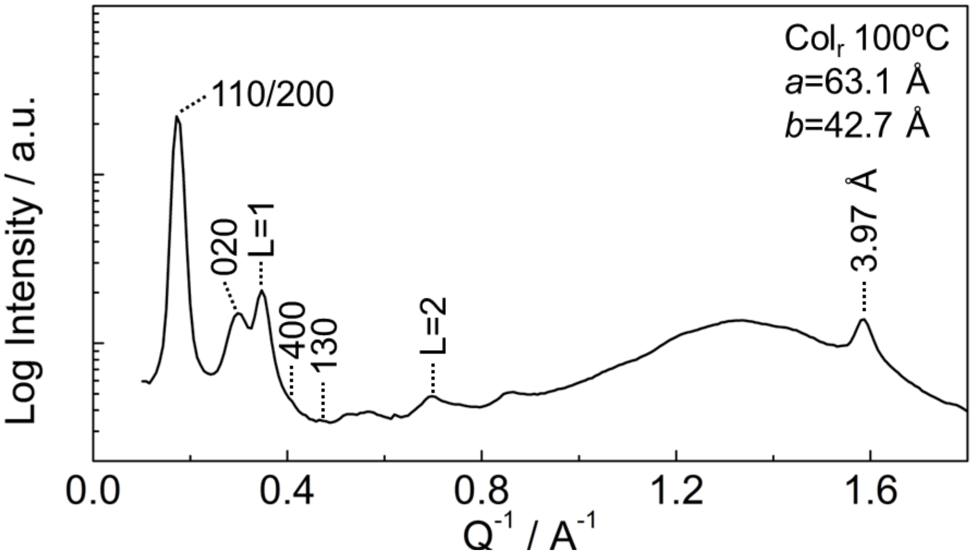

Figure S23. Integrated intensities of the WAXS pattern of **1·2** at 100 °C, showing the reflections indexed according to a centered columnar rectangular phase.

Table S6. Data of the WAXS pattern of **1·2** at 100 °C compared to the theoretical centered columnar rectangular geometry reflections.

Entry	hkl	d_{exp} / Å	d_{calc} / Å	Δ / Å
1	110*	70.9*	70.8	0.1*
2	200*	63.1*	63.1	0.0*
3	020	42.7	42.7	0.0
4	310*	37.2*	37.7	0.5*
5	220*	35.1*	35.4	0.3*
6	400	31.5	31.5	0.1
7	130	27.5	27.8	0.3
	L=1	18.1		
	L=2	9.1		

*****The presence of these reflections was confirmed from the GiWAXS patterns (see below figure S40 and S41).

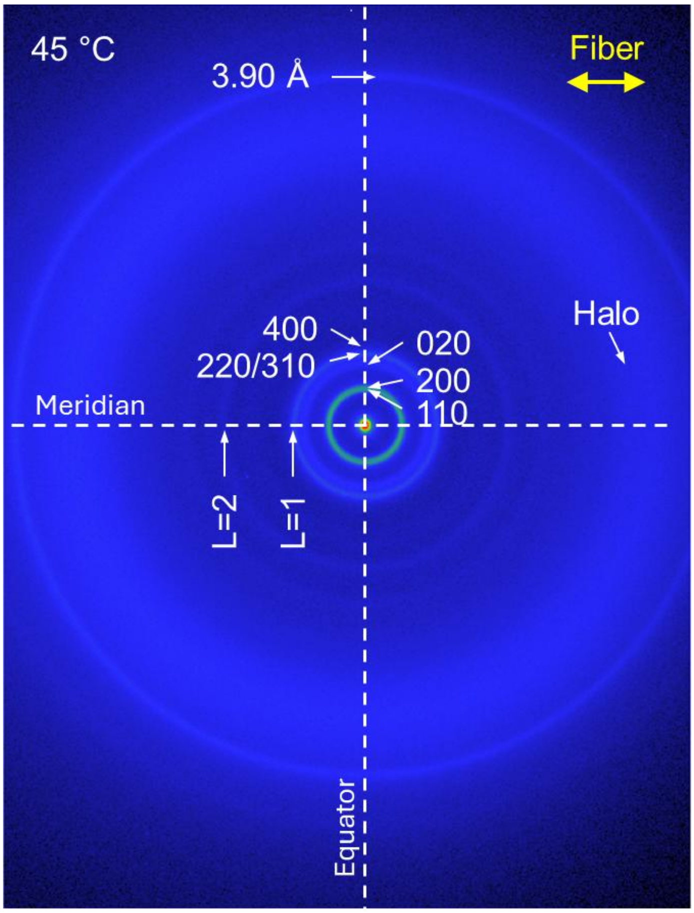

Figure S24. 2D WAXS pattern of a partially aligned fiber of **1·2** at 45 °C.

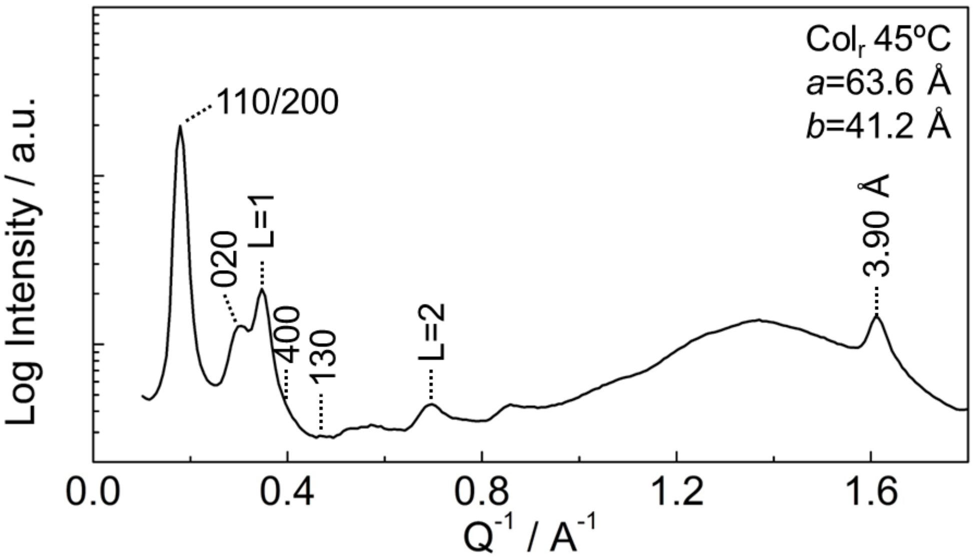

Figure S25. Integrated intensities of the WAXS pattern of **1·2** at 45 °C, showing the reflections indexed according to the centered columnar rectangular phase.

Table S7. Data of the WAXS pattern of **1·2** at 45 °C compared to the theoretical centered columnar rectangular geometry reflections.

Entry	hkl	d_{exp} / Å	d_{calc} / Å	Δ / Å
1	110*	69.2*	69.2	0.1*
2	200*	63.6*	63.6	0.0*
3	020	41.2	41.2	0.0
4	310*	37.8*	37.7	0.1*
5	220*	34.2*	34.6	0.4*
6	400	31.6	31.8	0.2
7	130	27.5	27.1	0.4
	L=1	18.1		
	L=2	9.1		

*****The presence of these reflections was deduced from the GiWAXS patterns (see below figure S40 and 41).

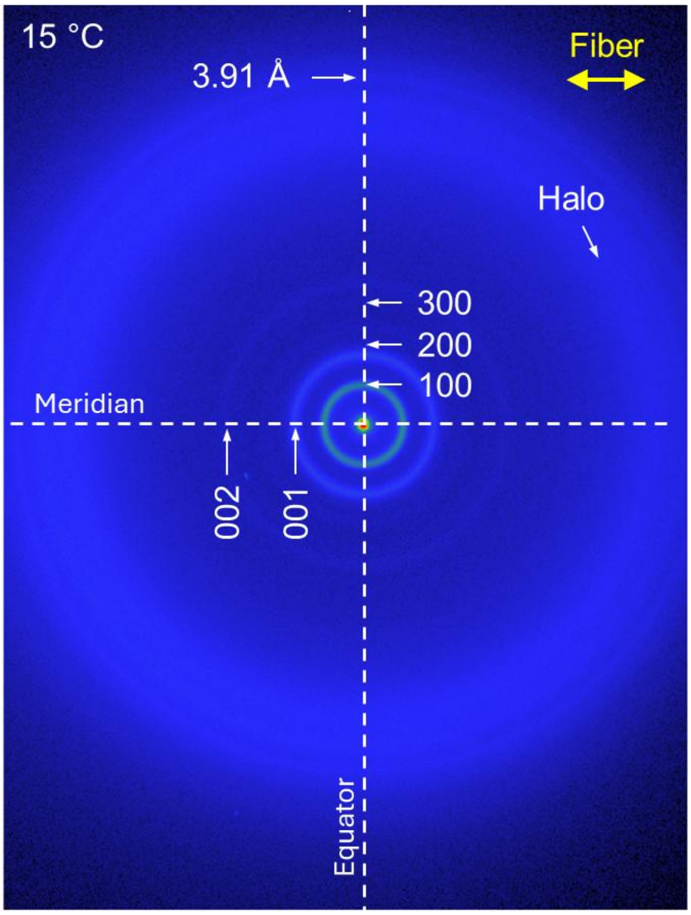

Figure S26. 2D WAXS pattern of a partially aligned fiber of **1·2** at 15 °C.

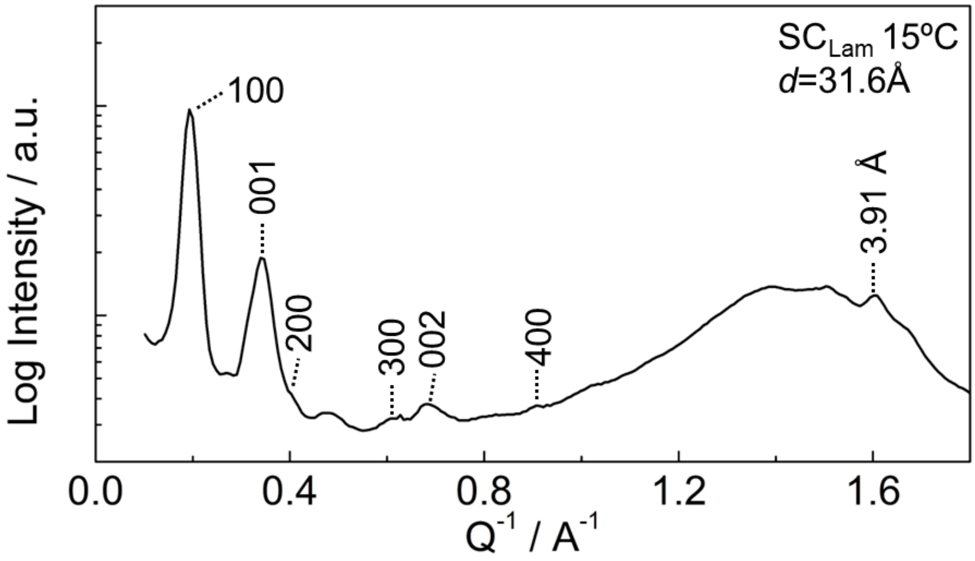

Figure S27. Integrated intensities of the WAXS pattern of **1·2** at 15 °C, showing the reflections indexed according to the lamellar soft-crystalline phase.

Table S8. Data of the WAXS pattern of **1·2** at 15 °C compared to the theoretical lamellar geometry reflections.

Entry	hkl	d_{exp} / Å	d_{calc} / Å	Δ / Å
1	100	31.6	31.6	0.0
2	200	15.8	15.8	0.0
3	300	10.3	10.5	0.2
4	400	7.9	7.9	0.0
5	001	18.3	18.3	0.0
6	002	9.2	9.2	0.0

-Calculation of the number of molecules per unit cell

The number of molecules per unit cell of the columnar phases of compounds **1** and **1·2** were calculated using the parameters obtained from the WAXS patterns using the following equations:

$$V_{unit\ cell} = a \times b \times c \times sen(ɣ)$$

$$Z = \frac{\delta \times N_a \times V_{unit\ cell}}{M}$$

a, b, c = unit cell parameter
ɣ = angle between the two directors of the unit cell
Z = number of molecules per unit cell
δ = density
M = molecular mass
N_A = Avogadro's Number
$V_{unit\ cell}$ = unit cell volume

Table S9. Parameters extracted from the WAXS pattern of **1** for the calculation of the number of molecules per unit cell.

Parameter	180 °C	100 °C
Phase	Col_r (simple)	Col_r (simple)
a	33.3 Å	32.7 Å
b	16.9 Å	17.1 Å
c	18.8 Å	18.8 Å
ɣ	90 º	90 º
M	1.68456 kg/mol	1.68456 kg/mol
δ	1000 kg/m^3 (supposed)	1000 kg/m^3 (supposed)
NA	$6.022 \cdot 10^{23}$ mol^{-1}	$6.022 \cdot 10^{23}$ mol^{-1}
$V_{unitcell}$	10580 Å^3 $1.0580 \cdot 10^{-26}$ m^3	10512 Å^3 $1.0512 \cdot 10^{-26}$ m^3
Z	3.78≈4 per column	3.75≈4 per column

Table S10. Parameters extracted from the WAXS pattern of **1·2** for the calculation of the number of molecules per unit cell.

Parameter	100 °C	45 °C
Phase	Col_r (centered)	Col_r (centered)
a	63.1 Å	63.6 Å
b	42.7 Å	41.2 Å
c	18.1 Å	18.1 Å
ɣ	90 º	90 º
M	1.64356 kg/mol	1.64356 kg/mol
δ	900 kg/m^3 (supposed)	900 kg/m^3 (supposed)
NA	$6.022 \cdot 10^{23}$ mol^{-1}	$6.022 \cdot 10^{23}$ mol^{-1}
$V_{unitcell}$	48768 Å^3 $4.8768 \cdot 10^{-26}$ m^3	47427 Å^3 $4.7427 \cdot 10^{-26}$ m^3
Z	16.1 (~8 per column)	15.6 (~8 per column)

7. Photophysical properties in bulk

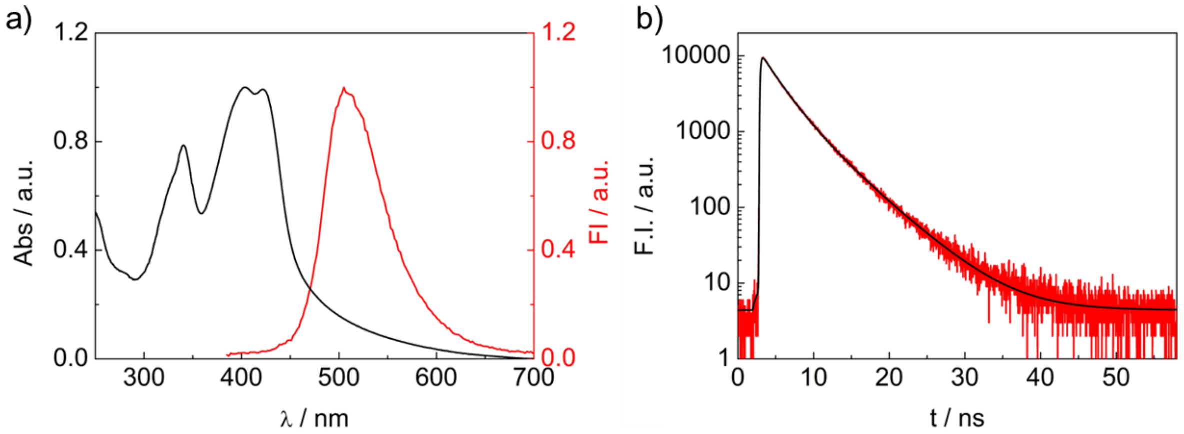

Figure S28. a) Absorption (bulk) and emission (bulk) (λ_{ex}=424 nm) spectra of compound **1** at 25 ºC. b) The fluorescence decay curve (red) and fitting curve (black) of **1** (bulk).

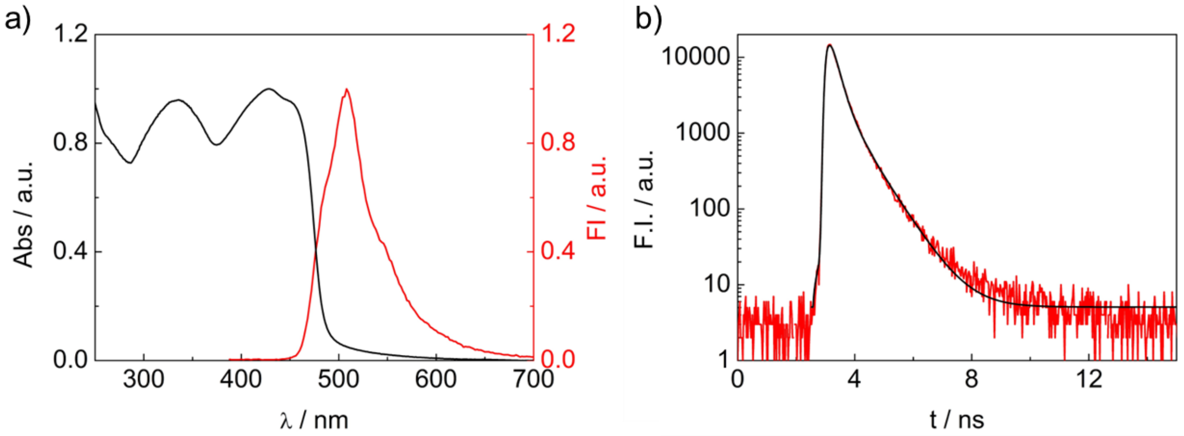

Figure S29. a) Absorption (bulk) and emission (bulk) (λ_{ex}=431 nm) spectra of compound **2** at 25 ºC. b) The fluorescence decay curve (red) and fitting curve (black) of **2** (bulk).

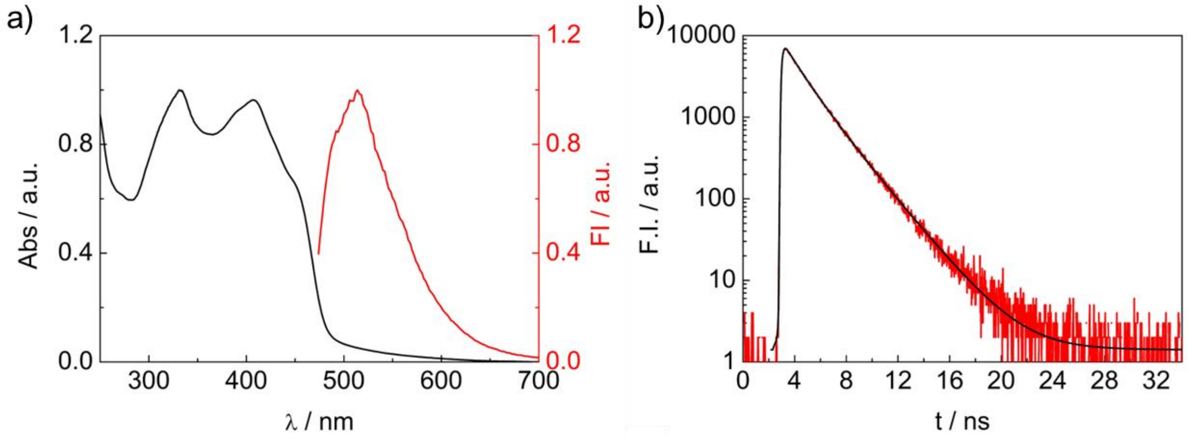

Figure S30. a) Absorption (bulk) and emission (bulk) (λ_{ex}=411 nm) spectra of compound **1·2** at 25 ºC. b) The fluorescence decay curve (red) and fitting curve (black) of **1·2** (bulk).

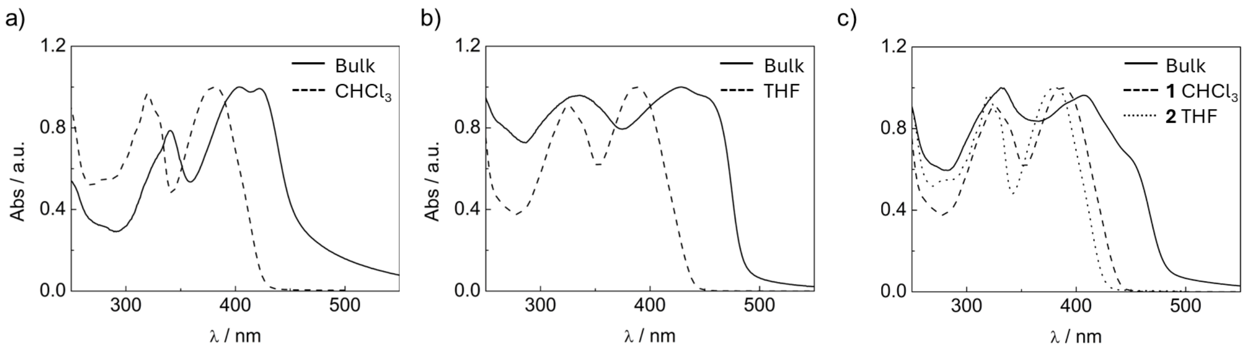

Figure S31. Comparison of the UV/vis absorption spectra in solution vs bulk for compounds a) **1**, b) **2** and c) **1** and **2** in solution and **1·2** bulk.

Table S11. Absorption and emission maxima, quantum yields (calculated by the absolute method)[2] and fluorescence lifetimes for **1**, **2** and **1·2** in solution and bulk.

	Abs			Em				
**Sample**	**λ_1(nm)**	**λ_2(nm)**	**λ_3(nm)**	**λ_1(nm)**	**λ_2(nm)**	**ϕ**	**τ_1(ns)**	**τ_2(ns)**
**1** ($CHCl_3$)	321	382	-	431	464	0.72	1.585	0.390
**2** (THF)	325	388	-	476	-	0.66	2.013	-
**1+2** ($CHCl_3$)	331	375	454	432	465	0.31	1.471	0.327
**1** (bulk)	342	407	424	507		0.63	4.962	2.335
**2** (bulk)	339	431	454	508	-	0.03	0.712	0.207
**1·2** (bulk)	335	411	451	513	-	0.36	2.232	1.121

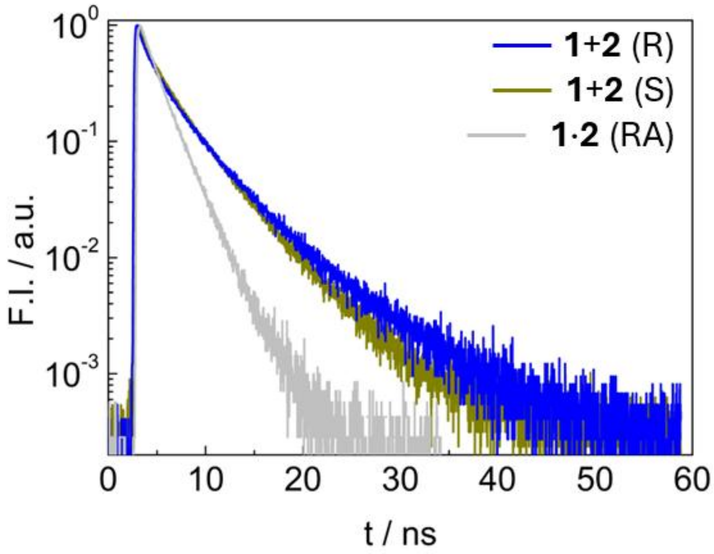

Figure S32. Fluorescence lifetimes and the corresponding decay curves of differently prepared mixtures of **1** and **2** at 25 ºC. **1+2** (R) was prepared by mechanical mixture of the two bulk compounds at room temperature; **1·2** (RA) was prepared by evaporation of an equimolar mixture of **1** and **2** in $CHCl_3$ and annealing the sample at 160ºC in bulk; and **1+2** (S) corresponds to the simulated decay of the sum of bulk **1** and bulk **2** measured independent experiments.

Table S12. Fluorescence lifetimes for **1·2** (mixture of **1** and **2** in solution, concentrated and annealed in bulk) and **1+2** (mechanical mixture of the two bulk compounds).

**Sample**	**τ_1(ns)**	**τ_2(ns)**	**τ_3(ns)**	**τ_4(ns)**
**1·2 (RA)**	2.232	1.121	-	-
**1+2 (R)**	3.80	0.27	1.19	8.33

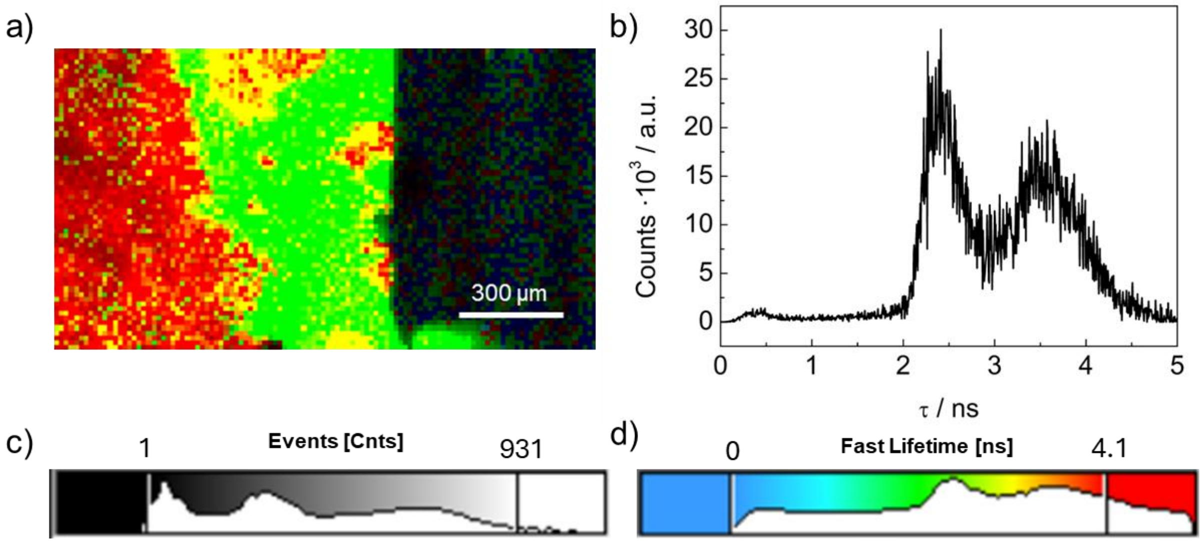

Figure S33. a) FLIM contact angle experiments on annealed samples of **1** (left) and **2** (right) measured on a quartz plate at 25 °C. b) Distribution of lifetimes. c) Distribution of pixels with certain counts and the limits defined for the image above. d) Distribution of pixels with certain lifetimes and the limits defined for the image above.

8. Infrared experiments

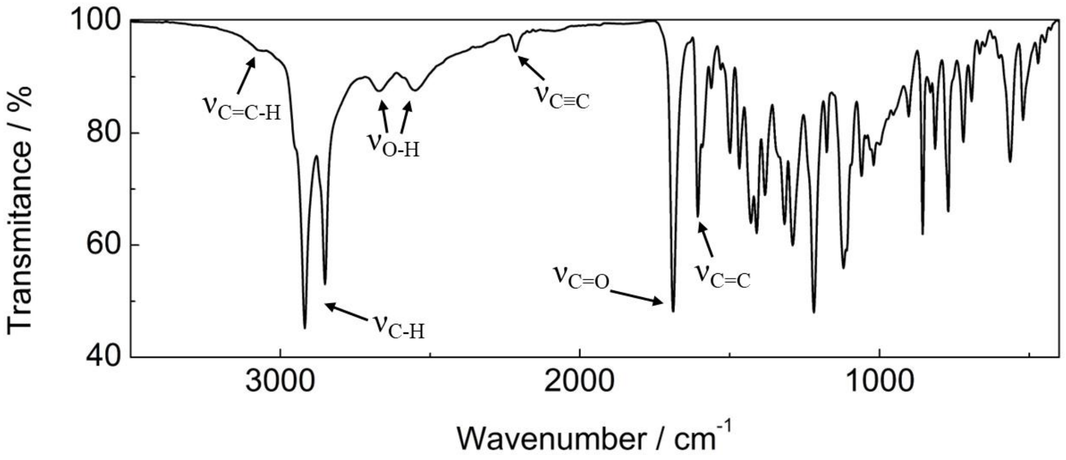

Figure S34. FT-IR of bulk **1** at 25 ºC.

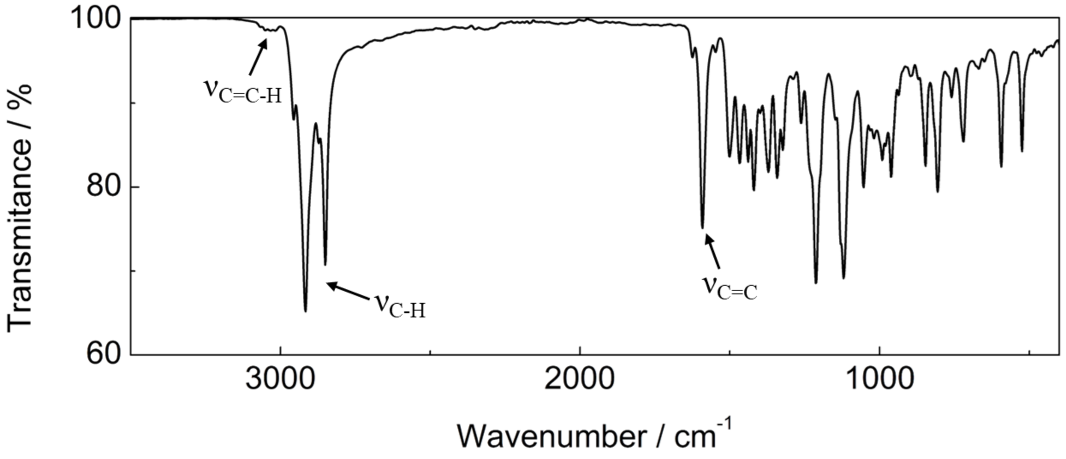

Figure S35. FT-IR of bulk **2** at 25 ºC.

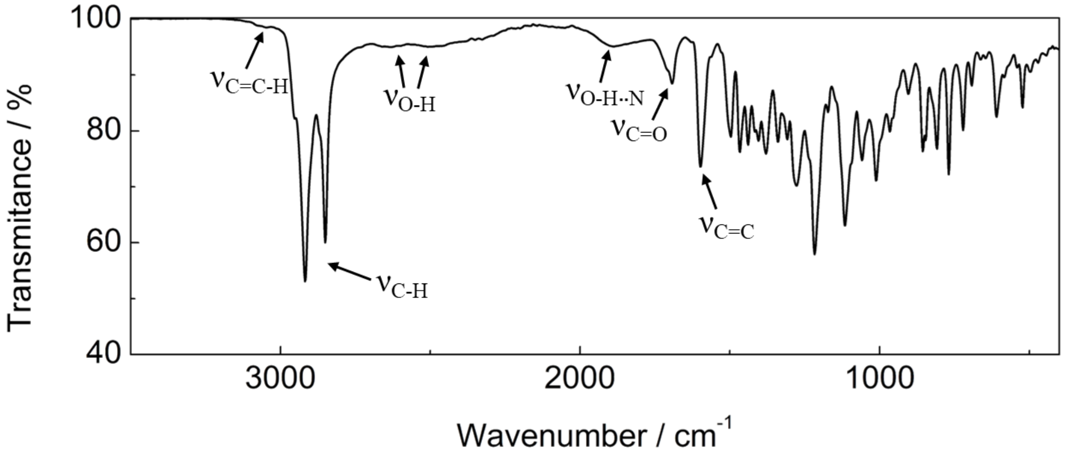

Figure S36. FT-IR of bulk **1·2** at 25 ºC.

Table S13. Wavenumbers of the most relevant FT-IR signals for the different samples of **1**, **2** and **1·2**.

	C=C-H / cm^{-1}	CH1 / cm^{-1}	CH2 / cm^{-1}	O-H1 / cm^{-1}	O-H2 / cm^{-1}	C=O / cm^{-1}	C=C / cm^{-1}
**1**	3060	2918	2850	2673	2555	1689	1606
**2**	3035	2916	2850	-	-	-	1591
**1·2**	3060	2918	2850	2638	2497	1693	1598

9. Anisotropic experiments

-POM of aligned samples

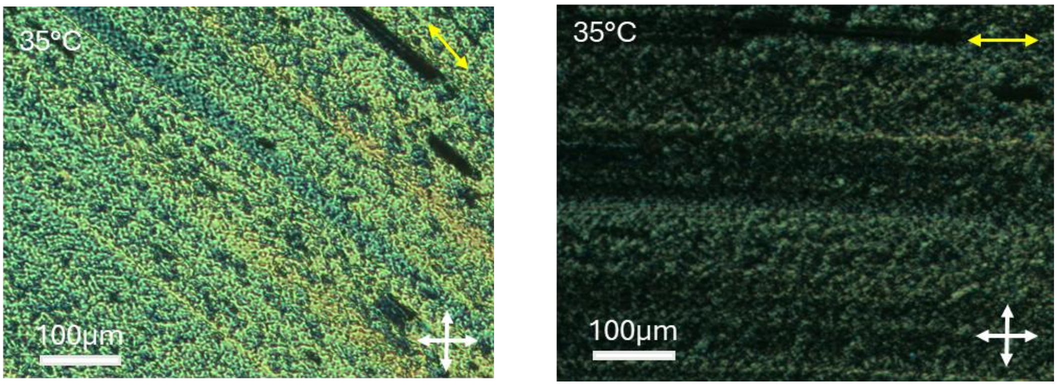

Figure S37. POM images of **1** after shearing the sample on a glass substrate. (right) Shearing direction parallel or perpendicular to the polarizer/analyzer, (left) after 45° sample holder rotation.

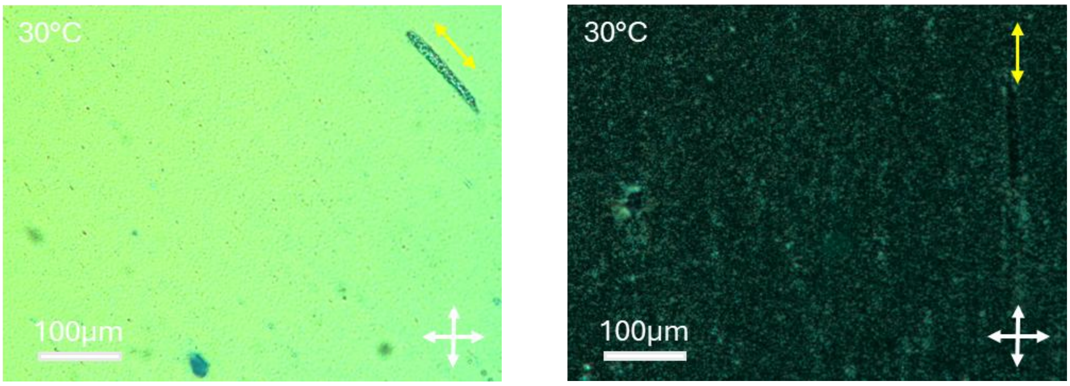

Figure S38. POM images of **1·2** after shearing the sample on a glass substrate. (right) Shearing direction parallel or perpendicular to the polarizer/analyzer, (left) after 45° sample holder rotation.

- *GiWAXS*

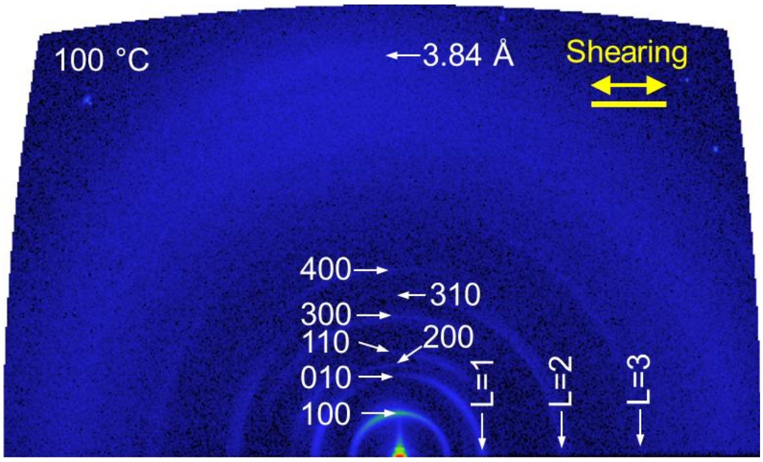

Figure S39. 2D GiWAXS pattern of a sheared sample of **1** at 100 °C.

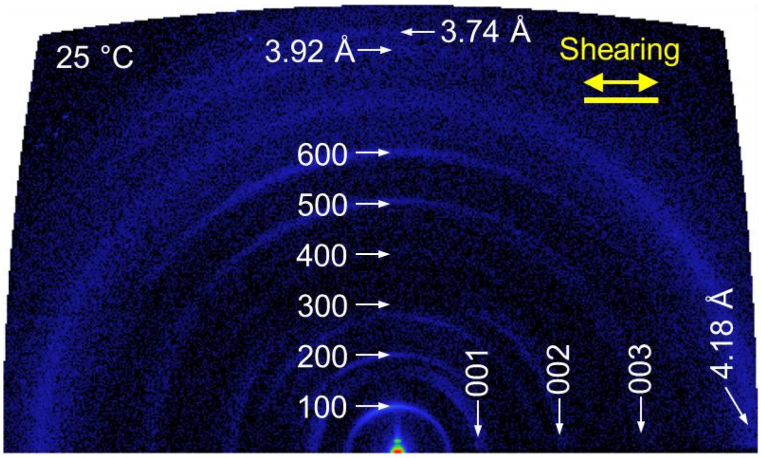

Figure S40. 2D GiWAXS pattern of a sheared sample of **1** at 25 °C.

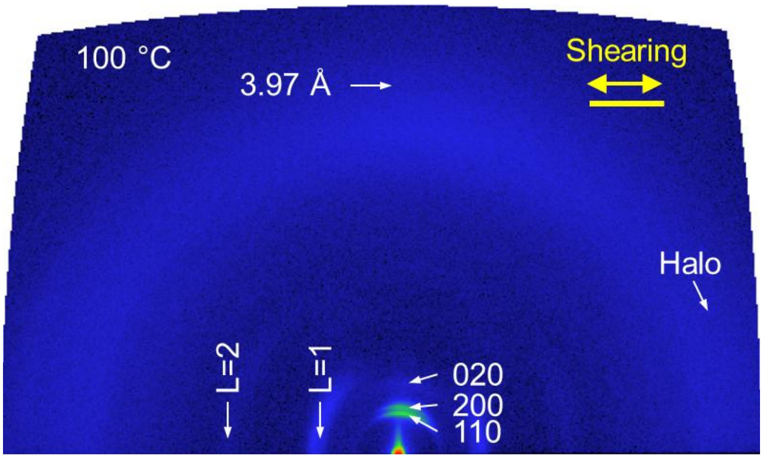

Figure S41. 2D GiWAXS pattern of a sheared sample of **1·2** at 100 °C.

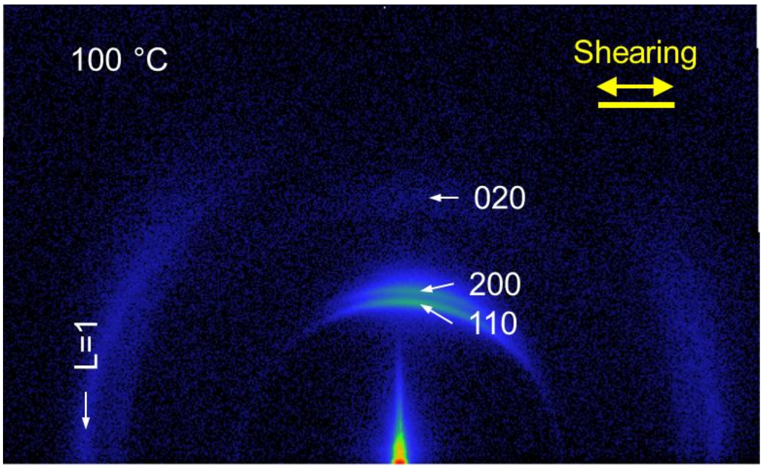

Figure S42. 2D GiMAXS pattern of a sheared sample of **1·2** at 100 °C.

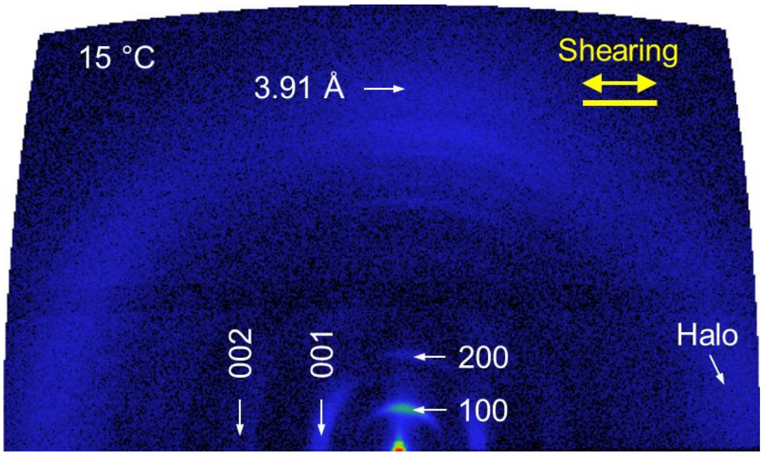

Figure S43. 2D GiWAXS pattern of a sheared sample of **1·2** at 15 °C.

- Polarized FT-IR

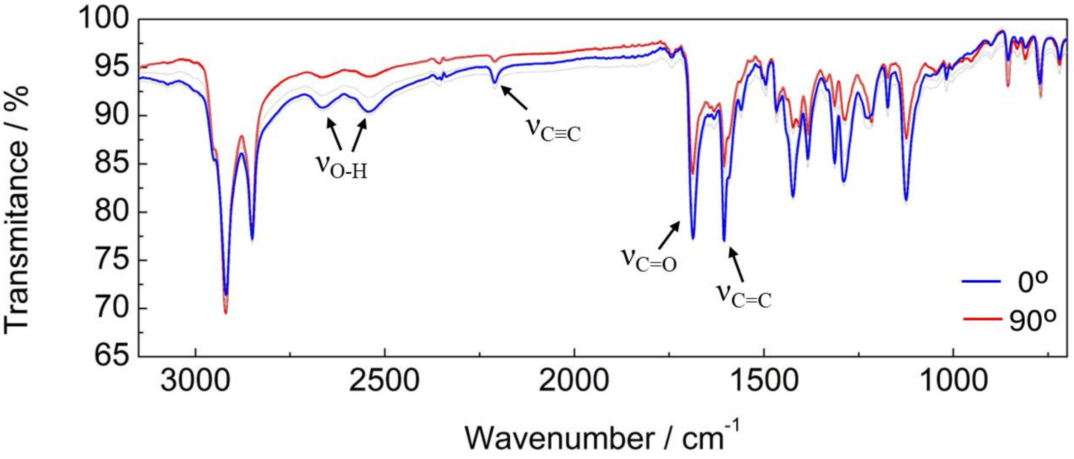

Figure S44. Polarized FT-IR of a sheared sample of **1** parallel to the polarized plane (blue) and perpendicular (red).

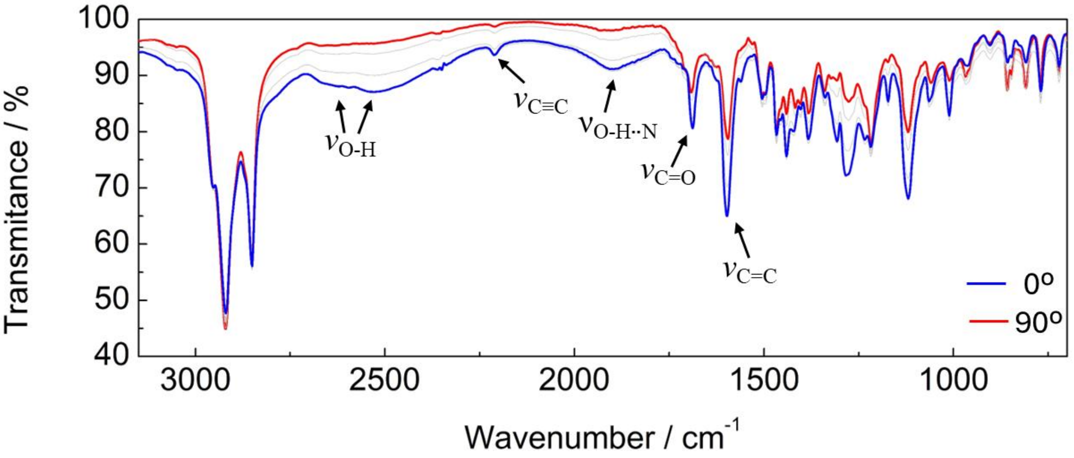

Figure S45. Polarized FT-IR of a sheared sample of **1·2** parallel to the polarized plane (blue) and perpendicular (red).

-Polarized UV/vis

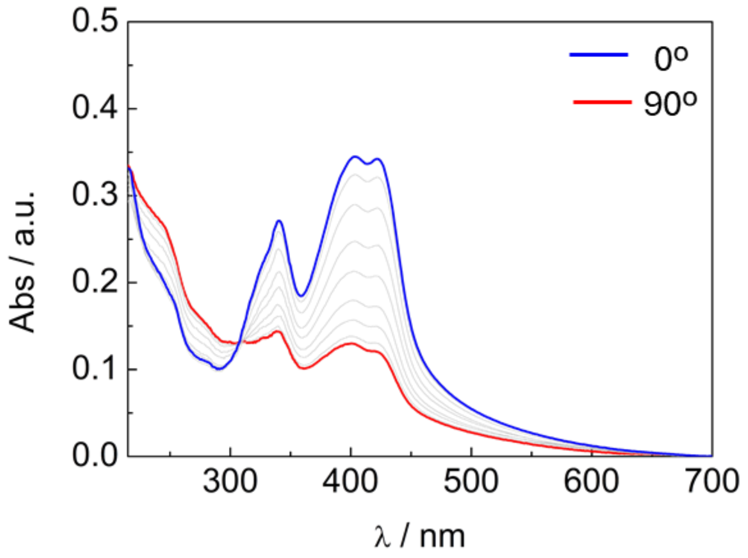

Figure S46. Polarized UV/vis of a sheared sample of **1** (annealed) parallel to the polarized plane blue and perpendicular red.

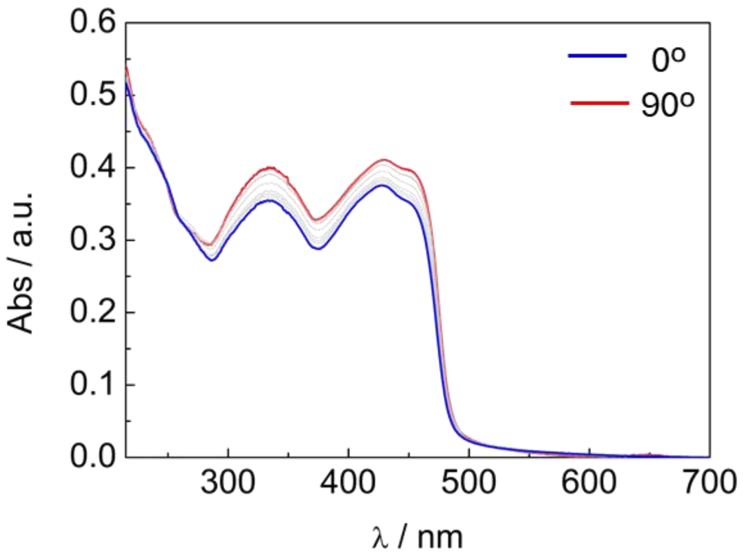

Figure S47. Polarized UV/vis of a sheared sample of **2** (annealed) parallel to the polarized plane blue and perpendicular red.

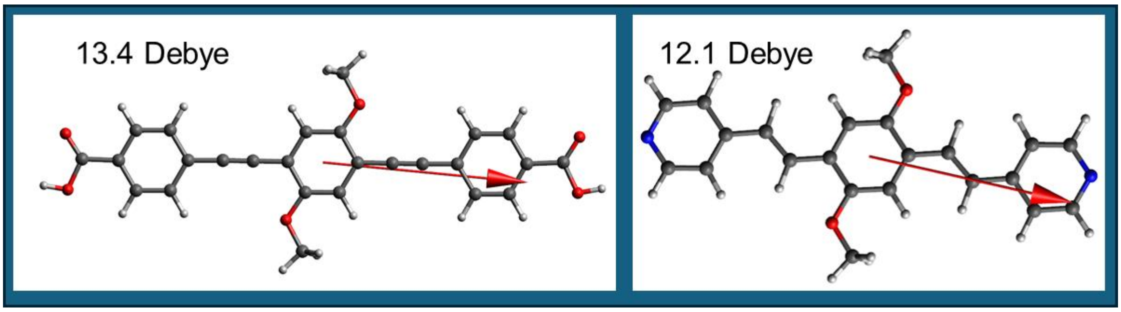

Figure S48. Representation of the transition dipole moment of **1** (left) and **2** (right). Transition dipole moments were computed using ORCA 5.0 at the RI-BP86-D4/def2-TZVP level of theory using fully optimized models.

10. Theoretical Calculations

For the calculations involving models where dodecyl groups were replaced by methyl groups, we employed density functional theory (DFT) at the RI-BP86-D4/def2-TZVP level of theory.[4-7] The calculations for monomers, dimers, and tetramers, as presented in the main text, were performed without symmetry constraints using the Turbomole 7.7 program.[8] Time-dependent DFT (TD-DFT) calculations and transition dipole moments were computed at the same level using the ORCA 5.0 program.[9]

The geometries of the supramolecular assemblies in this study were fully optimized using the extended semiempirical tight-binding model developed by Grimme.[10] This method is specifically designed for the efficient calculation of structures and noncovalent interaction energies in molecular systems containing approximately 1000 atoms. Notably, the assemblies reported here contain more than 4500 atoms. The XTB-GN2 program, which implements this model, is available free of charge at https://github.com/grimme-lab/xtb. The key innovation of this method is the inclusion of anisotropic second-order density fluctuation effects through short-range damped interactions of cumulative atomic multipole moments. Without a significant increase in computational cost, this approach provides a less empirical and more physically rigorous method that does not require classical halogen or hydrogen bonding corrections. Additionally, the D4 London dispersion model is incorporated self-consistently,[6] and fully analytical, numerically precise gradients (nuclear forces) are implemented.
For the modeling of columanar assemblies of **1** and **1·2**, formic acid units were included to cap the carboxylate ends. This approach is reasonable since these units mimic the next monomeric units in the real assembly, thereby facilitating self-assembly. In the representations of these assemblies in Figure 5, the formic acid units were omitted. Furthermore, while the four-stranded columnar assembly in Figure 5e was fully optimized (comprising 3544 atoms), the eight-stranded co-assembly in Figure 5f (four molecules per strand) was generated by assembling two eight-stranded units with two molecules per strand. This model already consists of 4680 atoms, reaching our computational limit. The complete assembly shown in Figure 5f, which contains twice as many atoms (9360 atoms), exceeds our computational capabilities. However, it is noteworthy that the optimized fragments composed of eight strands and two molecules per strand self-assemble seamlessly.

*-Room-temperature assembly of **1***

Figure S48 illustrates the one-dimensional columnar assembly of **1** observed at high temperature (left), consisting of four stacked strands, alongside the crystal lamellar phase observed at room temperature (right). The latter exhibits a higher degree of ordering in the dodecyl chains (see bottom-right in Figure S48). In the high-temperature four-strand assembly, some dodecyl chains are positioned over the π-surface of the molecules, preventing further stacking of the strands. At room temperature, the increased ordering of the strands facilitates infinite strand growth through π-stacking, ultimately leading to the formation of the crystalline phase.

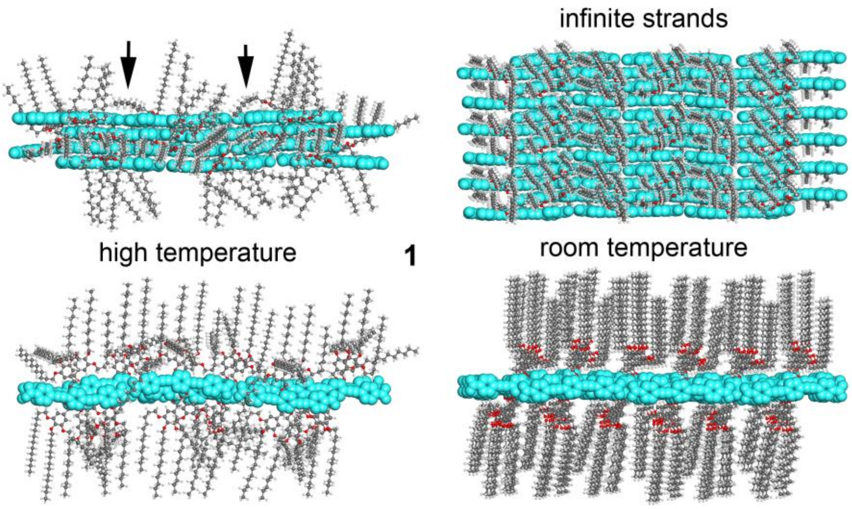

Figure S49. Left: Side and top views of the assembly model of **1**, consisting of 16 molecules fully optimized at the GFN2-xTB semiempirical level. Right: Crystal model of **1**, composed of 48 molecules (10,512 atoms), generated by replicating one-third of a fully optimized model at the same computational level.

Note: The coordinates for all the DFT models and the semiempirical models are provided in a separate supplementary file.

11. Calculation of exciton coupling

The exciton coupling of the columnar phases of compounds **1** were calculated using the measurements obtained from the theoretical calculations and the following equation:[11]

$$J_i = \frac{\mu_{eg}^2}{4\pi\varepsilon_0 r_{c-c}^3}(cos\alpha - 3\cos^2\theta)$$

J_i = exciton coupling
μ_{eg} = transition electric dipole moment
ε_0 = permittivity of the vacuum
r_{c-c} = distance between two coupling molecules
α = angle between the two transition electric dipole moments
θ = angle between the two coupling molecules

The sign (+/-) of the J_i indicate the type of exciton coupling contribution, where a negative exciton coupling indicates a J-type coupling and a positive exciton coupling indicates a H-type coupling.

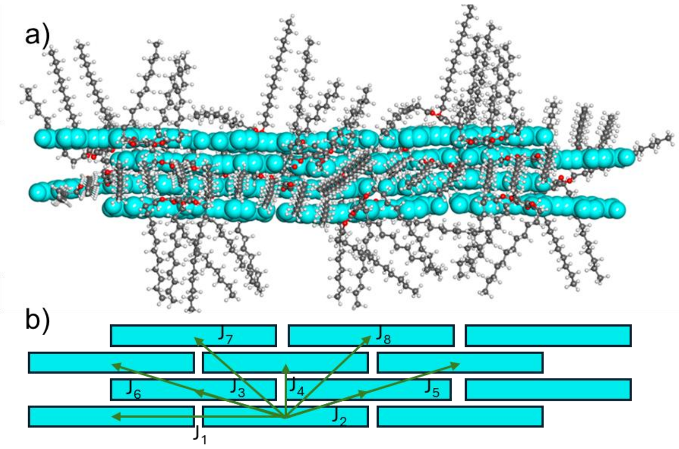

Figure S50. a) Optimized assembled structure of the columnar rectangular phase of TPE **1**. b) Representation of the different exciton couplings taking place in the columnar assembly of **1**.

Table S14. Calculated exciton coupling energies (J_i) for the TPE **1** columnar assembly; where μ_{eg} (transition electric dipole moment), $r_{c\text{-}c}$ (distance between two coupling molecules), θ (angle between two coupling molecules), α (angle between two transition dipole moments, all α in this assembly are equal to zero).

Entry	μ_{eg}/D	$r_{c\text{-}c}$ / Å	θ / °	J_i / cm^{-1}
J_1	13.4	11.27	15	-1136
J_2	13.4	22.95	5	-147
J_3	13.4	72.72	18	-752
J_4	13.4	6.16	84	3746
J_5	13.4	22.45	28	-108
J_6	13.4	25.11	25	-83
J_7	13.4	12.83	53	-38
J_8	13.4	16.63	36	-187

The exciton coupling of the columnar phases of compounds **1·2** were calculated using the measurements obtained from the theoretical calculation and the following equation:[11]

$$J_i = \frac{\mu_{eg\mathbf{1}}\mu_{eg\mathbf{2}}}{4\pi\varepsilon_0 r_{c-c}^3}(cos\alpha - 3\cos^2\theta)$$

J_i = exciton coupling
μ_{eg} = transition electric dipole moment of the corresponding molecules
ε_0 = permittivity of the vacuum
r_{c-c} = distance between two coupling molecules
α = angle between the two transition electric dipole moment
θ = angle between the two coupling molecules

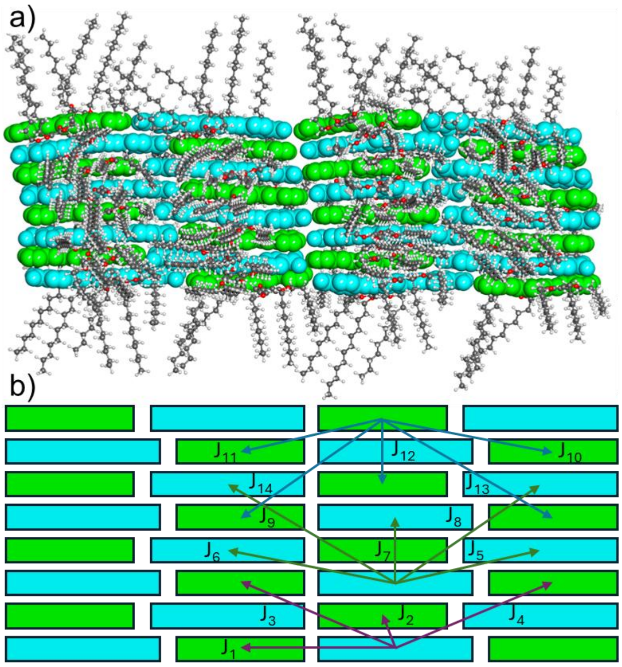

Figure S51. a) Results of the theoretical calculations. b) Representation of the different couplings taking place spread: hetero (purple), homo **1** (green) and homo **2** (blue)

Table S15. Calculated exciton coupling energies (J_i) for the **1·2** columnar assembly; where μ_{eg} (transition electric dipole moment), r_{c-c} (distance between two coupling molecules), θ (angle between two coupling molecules), α (angle between two transition dipole moments).

Entry	μ_{eg}/D	type	r_{c-c} / Å	θ / °	α / °	J_i / cm^{-1}
J_1	13.4/12.1	hetero	20.58	14	7	-171
J_2	13.4/12.1	hetero	4.40	53	7	-901
J_3	13.4/12.1	hetero	21.08	33	7	-97
J_4	13.4/12.1	hetero	23.71	15	7	-111
J_5	13.4	homo **1**	19.12	9	0	-250
J_6	13.4	homo **1**	25.45	7	0	-107
J_7	13.4	homo **1**	6.78	71	0	1978
J_8	13.4	homo **1**	20.35	34	0	-114
J_9	13.4	homo **1**	24.88	30	0	-73
J_{10}	12.1	homo **2**	22.31	9	0	-128
J_{11}	12.1	homo **2**	21.74	8	0	-140
J_{12}	12.1	homo **2**	6.67	72	0	1772
J_{13}	12.1	homo **2**	24.89	26	0	-68
J_{14}	12.1	homo **2**	20.34	25	0	-128

12. Supplementary references

1. Heiney, P. A. https://www.physics.upenn.edu/~heiney/datasqueeze/.

2. Würth, C.; Grabolle, M.; Pauli, J.; Spieles, M.; Resch-Genger, U. Relative and Absolute Determination of Fluorescence Quantum Yields of Transparent Samples. *Nat. Protoc.* **2013**, *8*, 1535–1550.

3. Ichikawa, T.; Yoshio, M.; Hamasaki, A.; Mukai, T.; Ohno, H.; Kato, T. Self-Organization of Room-Temperature Ionic Liquids Exhibiting Liquid-Crystalline Bicontinuous Cubic Phases: Formation of Nano-Ion Channel Networks. *J. Am. Chem. Soc.* **2007**, *129* (35), 10662–10663.

4. Becke, A. D. Density - Functional Exchange - Energy Approximation with Correct Asymptotic Behavior. *Phys. Rev. A* **1988**, *38* (6), 3098-3100.

5. Perdew, J. P. Density - Functional Approximation for the Correlation Energy of the Inhomogeneous Electron Gas. *Phys. Rev. B* **1986**, *33* (12), 8822-8824.

6. Caldeweyher, E.; Ehlert, S.; Hansen, A.; Neugebauer, H.; Spicher, S.; Bannwarth, C.; Grimme, S. A Generally Applicable Atomic-Charge Dependent London Dispersion Correction. *J. Chem. Phys*. **2019**, *150* (15), 154122.

7. Weigend, F.; Ahlrichs, R. Balanced Basis Sets of Split Valence, Triple Zeta Valence and Quadruple Zeta Valence Quality for H to Rn: Design and Assessment of Accuracy. *Phys. Chem. Chem. Phys*. **2005**, *7* (18), 3297–3305.

8. Balasubramani, S. G.; Chen, G. P.; Coriani, S.; Diedenhofen, M.; Frank, M. S.; Franzke, Y. J.; Furche, F.; Grotjahn, R.; Harding, M. E.; Hättig, C.; Hellweg, A.; Helmich-Paris, B.; Holzer, C.; Huniar, U.; Kaupp, M.; Marefat Khah, A.; Karbalaei Khani, S.; Müller, T.; Mack, F.; Nguyen, B. D.; Parker, S. M.; Perlt, E.; Rappoport, D.; Reiter, K.; Roy, S.; Rybkin, V. V.; Schäfer, A.; Scheele, M.; Schneider, M.; Sierka, M.; Tapavicza, E.; Tew, D. P.; van Wüllen, C.; Voora, V. K.; Weigend, F.; Wodyński, A.; Yu, V. W. TURBOMOLE: Modular Program Suite for Ab Initio Quantum-Chemical and Condensed-Matter Simulations. *J. Chem. Phys*. **2020**, *152* (18), 184107.

9. Neese, F. Software Update: The ORCA Program System—Version 5.0. *Wiley Interdiscip. Rev. Comput. Mol. Sci*. **2022**, *12* (1), e1606.

10. Bannwarth, C.; Ehlert, S.; Grimme, S. GFN2-xTB—An Accurate and Broadly Parametrized Self-Consistent Tight-Binding Quantum Chemical Method with Multipole Electrostatics and Density-Dependent Dispersion Contributions. *J. Chem. Theory Comput*. **2019**, *15* (3), 1652–1671.

11. Kasha, M.; Rawls, H. R.; Ashraf El-Bayoumi, M. The Exciton Model in Molecular Spectroscopy. *Pure Appl. Chem.* **1965**, *11* (3–4), 371–392.
