# Supplementary material for: Luminescent Liquid-Crystalline J‑Aggregate Based on a Columnar Axial Coassembly
Source: J Am Chem Soc. 2025 May 28;147(27):23556–65. doi: 10.1021/jacs.5c03166 (PMC12257545; doi:10.1021/jacs.5c03166)
Supplement: Supplementary file 2 [file ja5c03166_si_002.pdf]

Supplementary File

Luminescent Liquid-Crystalline J-Aggregate based on a Columnar Axial Co-Assembly

Llorenç Rubert, Clémence Marre, Pedro Ximenis, Rosa M. Gomila, Antonio Frontera, Bartolome Soberats*[a]

Universitat de les Illes Balears
Department of Chemistry
Cra. Valldemossa, Km. 7.5,
07122, Palma de Mallorca, Spain

Cartesian Coordinates of DFT optimized compounds

1

C 1.0740759 0.8939038 -0.3692206

C 1.4024111 -0.4043164 0.0088410

C 0.3728938 -1.3022010 0.4030886

C -0.9648169 -0.8542328 0.4074315

C -1.2927782 0.4447000 0.0321213

C -0.2638555 1.3411518 -0.3671287

C -0.5808145 2.6570804 -0.7570718

C 0.6867989 -2.6220851 0.7816761

O -2.5591631 0.9455586 0.0128490

O 2.6689603 -0.9038569 0.0323890

C -0.8590485 3.7969606 -1.0991551

C -1.2074743 5.1076670 -1.4937607

C -2.5527998 5.5380930 -1.4270553

C -2.9013841 6.8235335 -1.8166558

C -1.9198545 7.7134659 -2.2842389

C -0.5814898 7.2933768 -2.3532582

C -0.2259297 6.0112629 -1.9640948

C 0.9564347 -3.7687921 1.1073582

C 1.2931980 -5.0892740 1.4783288

C 0.2937352 -6.0130114 1.8605365

C	0.6330156	-7.3090344	2.2229631
C	1.9767477	-7.7181566	2.2145067
C	2.9756564	-6.8060678	1.8360167
C	2.6439868	-5.5105169	1.4723578
C	-2.2400797	9.0947734	-2.7149147
O	-1.4311933	9.9033208	-3.1368270
O	-3.5697557	9.3958078	-2.5943255
C	2.3919403	-9.0892202	2.5930915
O	3.5409302	-9.4961151	2.6071083
O	1.3357407	-9.8885791	2.9380856
H	1.8420374	1.5992723	-0.6772214
H	-1.7326864	-1.5603731	0.7139282
H	-3.3102449	4.8438330	-1.0647882
H	-3.9390476	7.1486893	-1.7625962
H	0.1657497	7.9972559	-2.7191568
H	0.8122155	5.6846367	-2.0176571
H	-0.7479650	-5.6932890	1.8663111
H	-0.1415037	-8.0156680	2.5164526
H	4.0125341	-7.1418015	1.8355368
H	3.4159621	-4.8005768	1.1774136
H	-3.6522004	10.3207175	-2.9050661
H	1.7327804	-10.7545006	3.1642025
C	-3.6171110	0.0769762	0.4138478
H	-3.6893614	-0.8001982	-0.2496126
H	-3.4858704	-0.2606987	1.4547958
H	-4.5334863	0.6710434	0.3352363
C	3.7285198	-0.0310880	-0.3560420
H	3.6070941	0.3080353	-1.3976636
H	3.7905271	0.8448301	0.3100168
H	4.6459690	-0.6222920	-0.2689802

2

C	-1.2328013	0.6521180	-0.0442127
C	-1.2015256	-0.7342638	0.0563314
C	0.0406728	-1.4260408	0.0968920
C	1.2164274	-0.6556551	0.0319697
C	1.1854215	0.7303415	-0.0682987
C	-0.0566373	1.4225254	-0.1084057
C	-0.0636625	2.8678813	-0.2123374
C	-1.1527375	3.6746896	-0.2325762
C	-1.1354900	5.1258408	-0.3390688
C	0.0514873	-2.8711327	0.2054632
C	1.1452309	-3.6717223	0.2226369
C	1.1404414	-5.1218540	0.3390675
C	-0.0252012	-5.9041072	0.4669566
C	0.0854603	-7.2861564	0.5711036
N	1.2514947	-7.9575892	0.5599123
C	2.3622061	-7.2145955	0.4386249
C	2.3598873	-5.8257086	0.3277536
C	0.0378588	5.8985337	-0.4546615
C	-0.0593928	7.2823006	-0.5478258
N	-1.2192505	7.9644253	-0.5368021
C	-2.3373122	7.2304820	-0.4282718
C	-2.3484345	5.8407163	-0.3291981
O	2.3103690	1.5083560	-0.1293351
O	-2.3253900	-1.5137841	0.1184058
H	-2.1899248	1.1670652	-0.0787016
H	2.1733475	-1.1705917	0.0675342
H	0.9250005	3.3230273	-0.2765128
H	-2.1521806	3.2359962	-0.1604334
H	-0.9350069	-3.3298350	0.2777310
H	2.1410736	-3.2267019	0.1406996
H	-1.0128813	-5.4431009	0.4863154

H	-0.8154808	-7.8984393	0.6701203
H	3.3086144	-7.7634115	0.4293480
H	3.3030354	-5.2837247	0.2312028
H	1.0213821	5.4287219	-0.4734121
H	0.8477419	7.8868410	-0.6376328
H	-3.2786546	7.7878628	-0.4201540
H	-3.2970800	5.3067025	-0.2431330
C	3.5745928	0.8523134	-0.0973626
H	3.7129095	0.2915907	0.8417573
H	3.6939052	0.1679768	-0.9533352
H	4.3246674	1.6479080	-0.1598080
C	-3.5912182	-0.8606310	0.0919933
H	-3.7085938	-0.1766488	0.9484641
H	-3.7351425	-0.3004646	-0.8465729
H	-4.3392291	-1.6580571	0.1577684

12

C	-3.6913545	10.3025227	-1.4426742
C	-4.0098695	11.6301494	-1.7107846
C	-3.0690808	12.4549961	-2.3862535
C	-1.8274875	11.9068546	-2.7713853
C	-1.5090087	10.5792219	-2.5033258
C	-2.4496602	9.7544745	-1.8276461
C	-2.1440149	8.4083080	-1.5476490
C	-3.3764661	13.8006082	-2.6672774
O	-0.3329824	9.9839222	-2.8459432
O	-5.1864703	12.2249362	-1.3699276
C	-1.8805722	7.2400259	-1.3035477
C	-1.5519220	5.8924256	-1.0374950
C	-0.2892273	5.3794783	-1.4157336
C	0.0369951	4.0555483	-1.1616356
C	-0.8842194	3.2068627	-0.5251071

C	-2.1391909	3.7088187	-0.1443143
C	-2.4718721	5.0316993	-0.3942235
C	-3.6454007	14.9675653	-2.9116682
C	-3.9821527	16.3129531	-3.1788832
C	-3.0898373	17.1621287	-3.8725378
C	-3.4294279	18.4833113	-4.1290457
C	-4.6664376	18.9931071	-3.7006082
C	-5.5583863	18.1554826	-3.0107959
C	-5.2264318	16.8349273	-2.7524224
C	-0.5583183	1.7873507	-0.2496631
O	-1.3768699	1.0301947	0.3081714
O	0.6450468	1.4108649	-0.6405198
C	-5.0771244	20.3953843	-3.9475824
O	-6.1342884	20.8894581	-3.5947208
O	-4.1337022	21.1139816	-4.6310188
H	-4.3929854	9.6503942	-0.9282835
H	-1.1258442	12.5588871	-3.2858506
H	0.4212384	6.0413236	-1.9098600
H	1.0089034	3.6613909	-1.4541847
H	-2.8428341	3.0385807	0.3484716
H	-3.4454212	5.4224509	-0.0997070
H	-2.1304732	16.7651745	-4.2031974
H	-2.7373579	19.1320145	-4.6635401
H	-6.5130923	18.5685992	-2.6857936
H	-5.9163001	16.1824038	-2.2183744
H	0.8038560	0.4082211	-0.4192448
H	-4.5165517	22.0095724	-4.7313387
C	0.6328295	10.7809409	-3.5288105
H	0.2420222	11.1460585	-4.4925398
H	0.9518412	11.6385959	-2.9140920
H	1.4884782	10.1216052	-3.7076558
C	-6.1593243	11.4233094	-0.7024801

H	-6.4695266	10.5686616	-1.3257821
H	-5.7797798	11.0536508	0.2639752
H	-7.0178644	12.0807277	-0.5306561
C	3.6407333	-10.2409787	1.4983672
C	4.0223340	-11.5570687	1.7400555
C	3.1112740	-12.4468917	2.3726731
C	1.8348708	-11.9733398	2.7435710
C	1.4535592	-10.6570500	2.5022733
C	2.3643016	-9.7675163	1.8694159
C	1.9986276	-8.4309719	1.6165522
C	3.4790658	-13.7832700	2.6241750
O	0.2407367	-10.1324652	2.8319627
O	5.2351248	-12.0812388	1.4100674
C	1.6928854	-7.2685396	1.3947603
C	1.3381130	-5.9237105	1.1492976
C	0.0706330	-5.4315723	1.5389973
C	-0.2729058	-4.1077061	1.3048386
C	0.6358107	-3.2397647	0.6767670
C	1.8949379	-3.7218753	0.2834984
C	2.2447198	-5.0432400	0.5136683
C	3.7945221	-14.9438382	2.8422444
C	4.1798819	-16.2818654	3.0798522
C	3.2860471	-17.1994839	3.6780377
C	3.6708361	-18.5139315	3.9038523
C	4.9566364	-18.9483723	3.5406238
C	5.8511332	-18.0420569	2.9476381
C	5.4733702	-16.7282072	2.7186521
C	0.3005668	-1.8196797	0.4180518
O	1.1181725	-1.0560450	-0.1320041
O	-0.9040531	-1.4487325	0.8110510
C	5.4170953	-20.3400914	3.7589206
O	6.5186769	-20.7694467	3.4614337

O	4.4651197	-21.1305029	4.3442190
H	4.3182074	-9.5404106	1.0164281
H	1.1572061	-12.6738115	3.2252594
H	-0.6296682	-6.1087100	2.0268670
H	-1.2484924	-3.7297861	1.6067657
H	2.5877010	-3.0363298	-0.2032822
H	3.2213469	-5.4182733	0.2091970
H	2.2893092	-16.8606369	3.9589553
H	2.9767307	-19.2155381	4.3637205
H	6.8445252	-18.3967399	2.6737377
H	6.1649431	-16.0230910	2.2588274
H	-1.0640455	-0.4454411	0.5959594
H	4.8829447	-22.0114667	4.4337508
C	-0.7007594	-10.9984922	3.4626049
H	-0.9542482	-11.8539873	2.8153788
H	-0.3200301	-11.3693374	4.4281255
H	-1.5958401	-10.3908307	3.6314966
C	6.1747375	-11.2163214	0.7747043
H	5.7910125	-10.8476916	-0.1904588
H	6.4302313	-10.3594236	1.4192484
H	7.0692821	-11.8243530	0.6043636

1·2

C	-3.3671944	11.3763133	-0.6177783
C	-3.3377776	9.9899882	-0.5184233
C	-2.0954611	9.2978500	-0.4770447
C	-0.9171996	10.0656752	-0.5411166
C	-0.9471877	11.4516197	-0.6404788
C	-2.1899971	12.1441019	-0.6805423
C	-2.1963111	13.5896891	-0.7839201
C	-3.2868916	14.3940101	-0.8040136
C	-3.2737889	15.8452921	-0.9100058

C	-2.0913146	7.8548296	-0.3688556
C	-1.0062092	7.0413888	-0.3463388
C	-1.0408780	5.5931616	-0.2317579
C	-2.2283122	4.8408593	-0.1019096
C	-2.1569125	3.4603909	-0.0007742
N	-0.9972197	2.7774960	-0.0189451
C	0.1633329	4.8617116	-0.2469823
C	-2.1025589	16.6205240	-1.0264341
C	-2.2040616	18.0041450	-1.1193683
N	-3.3662909	18.6819495	-1.1072876
C	-4.4823095	17.9454275	-0.9979892
C	-4.4891074	16.5555398	-0.8989299
O	0.1754147	12.2311941	-0.7013367
O	-4.4619256	9.2124139	-0.4582289
H	-4.3235772	11.8923760	-0.6522678
H	0.0391471	9.5492303	-0.5063626
H	-1.2080776	14.0454188	-0.8475859
H	-4.2853106	13.9530364	-0.7322177
H	-3.0820494	7.4047989	-0.3021269
H	-0.0033905	7.4704847	-0.4228809
H	-3.2034631	5.3259806	-0.0775105
H	-3.0644057	2.8610633	0.1007606
H	1.1172800	5.3825814	-0.3441979
H	-1.1178400	16.1533105	-1.0462869
H	-1.2991930	18.6117966	-1.2099294
H	-5.4250216	18.5003264	-0.9892262
H	-5.4358754	16.0184447	-0.8119821
C	1.4426579	11.5798513	-0.6705126
H	1.5844736	11.0210193	0.2690189
H	1.5638765	10.8959160	-1.5262875
H	2.1890415	12.3787026	-0.7346103
C	-5.7282610	9.8661843	-0.4858996

H	-5.8461897	10.5496100	0.3706114
H	-5.8709244	10.4260189	-1.4245808
H	-6.4760737	9.0687176	-0.4209107
C	3.0776204	-8.9890678	0.3742678
C	3.3181667	-10.3582186	0.4389541
C	2.2273961	-11.2566213	0.5917093
C	0.9179134	-10.7359210	0.6715341
C	0.6780288	-9.3672780	0.6064373
C	1.7694303	-8.4682278	0.4564237
C	1.5489121	-7.0778866	0.3912156
C	2.4532592	-12.6452374	0.6638728
O	-0.5574372	-8.7965230	0.6782325
O	4.5536768	-10.9274548	0.3650116
C	1.3673697	-5.8704489	0.3356287
C	1.1413286	-4.4760902	0.2721642
C	-0.1703170	-3.9583959	0.3678998
C	-0.3932435	-2.5891613	0.3030611
C	0.6809640	-1.6999413	0.1426781
C	1.9852274	-2.2076341	0.0489556
C	2.2180797	-3.5738738	0.1116485
C	2.6498945	-13.8502240	0.7276173
C	2.9020910	-15.2381745	0.7934785
C	1.8409538	-16.1652930	0.9099247
C	2.0972046	-17.5280441	0.9656621
C	3.4176390	-18.0046537	0.9083792
C	4.4778494	-17.0897327	0.7971872
C	4.2290433	-15.7276473	0.7399799
C	0.4810641	-0.2204262	0.0669819
O	1.4198356	0.5646629	-0.0617086
O	-0.7983842	0.1478808	0.1516091
C	3.7437148	-19.4485536	0.9573915
O	4.8683316	-19.9174822	0.9123851

O	2.6325263	-20.2426326	1.0594024
H	3.8945920	-8.2809428	0.2584728
H	0.0999494	-11.4429398	0.7878719
H	-1.0021696	-4.6512591	0.4927019
H	-1.4038568	-2.1903074	0.3757282
H	2.8057390	-1.5008886	-0.0746132
H	3.2316996	-3.9675041	0.0374307
H	0.8172617	-15.7945658	0.9530093
H	1.2745722	-18.2358808	1.0529381
H	5.4959295	-17.4766713	0.7560117
H	5.0492617	-15.0161157	0.6521212
H	-0.8637964	1.1908479	0.0860071
H	2.9748940	-21.1595906	1.0823065
C	-1.6769619	-9.6683899	0.8142249
H	-1.7579728	-10.3546079	-0.0448655
H	-1.6188794	-10.2549596	1.7458218
H	-2.5575071	-9.0181523	0.8465457
C	5.6727063	-10.0547936	0.2237971
H	5.6106828	-9.4698428	-0.7083524
H	5.7564194	-9.3678462	1.0817754
H	6.5529308	-10.7051803	0.1890834
C	0.1446683	3.4758724	-0.1407678
H	1.0550358	2.8715811	-0.1517867

14

C	-3.7536926	13.5381992	-0.2625195
C	-3.9836562	14.8687192	-0.5987141
C	-3.0089048	15.5834234	-1.3439863
C	-1.8303222	14.9217858	-1.7441048
C	-1.6084379	13.5884612	-1.4233976
C	-2.5749451	12.8783107	-0.6613471
C	-2.3458901	11.5310774	-0.3268489

C	-3.2354856	16.9234146	-1.7082809
O	-0.5067233	12.8782301	-1.7988751
O	-5.1043883	15.5653292	-0.2726340
C	-2.1119692	10.3619792	-0.0616990
C	-1.7954934	9.0119089	0.1958874
C	-0.5413926	8.4960134	-0.2028669
C	-0.2230407	7.1653917	0.0196402
C	-1.1502047	6.3107788	0.6353043
C	-2.3954021	6.8149149	1.0407207
C	-2.7150577	8.1477732	0.8321755
C	-3.4423128	18.0783590	-2.0498617
C	-3.7276089	19.3898231	-2.4846550
C	-2.7628911	20.1461971	-3.1881476
C	-3.0648180	21.4192228	-3.6502991
C	-4.3371638	21.9711272	-3.4254928
C	-5.3001140	21.2260934	-2.7234742
C	-5.0045021	19.9553152	-2.2544824
C	-0.8424573	4.8796492	0.8603147
O	-1.7028173	4.0987182	1.3149389
O	0.3882833	4.5221442	0.5549275
C	-4.7133175	23.3212043	-3.9051857
O	-5.8044430	23.8423556	-3.7512028
O	-3.6937938	23.9565858	-4.5621838
H	-4.4839337	12.9695129	0.3080256
H	-1.1077740	15.4878207	-2.3267212
H	0.1671765	9.1637428	-0.6908215
H	0.7379235	6.7657409	-0.2983122
H	-3.1001967	6.1367733	1.5210033
H	-3.6782217	8.5453559	1.1509422
H	-1.7798221	19.7122398	-3.3686119
H	-2.3179002	21.9958762	-4.1934941
H	-6.2825330	21.6686251	-2.5591445

H	-5.7548559	19.3689232	-1.7268355
H	0.5324208	3.5075091	0.7425390
H	-4.0615048	24.8247788	-4.8260842
C	0.4774847	13.5606206	-2.5700258
H	0.0630856	13.9130627	-3.5289922
H	0.8990033	14.4172311	-2.0188163
H	1.2678554	12.8271730	-2.7609115
C	-6.1361954	14.8686089	0.4208266
H	-6.4936851	14.0035186	-0.1612300
H	-5.7939446	14.5262594	1.4110803
H	-6.9505460	15.5899556	0.5425814
C	3.3258165	-7.1204101	2.3596085
C	3.7429202	-8.4349729	2.5319421
C	2.8922346	-9.3620856	3.1909096
C	1.6191482	-8.9363268	3.6238483
C	1.1926089	-7.6277027	3.4270703
C	2.0579001	-6.6921581	2.7991936
C	1.6555659	-5.3597573	2.5902082
C	3.3280481	-10.6823012	3.4067750
O	-0.0326954	-7.1515912	3.7879272
O	4.9386728	-8.9234383	2.0977033
C	1.3397625	-4.1969754	2.3862451
C	0.9902841	-2.8487583	2.1584838
C	-0.2868774	-2.3618827	2.5198000
C	-0.6152412	-1.0309101	2.3124082
C	0.3192280	-0.1509922	1.7427405
C	1.5851273	-0.6299957	1.3700664
C	1.9185997	-1.9601808	1.5685842
C	3.7433882	-11.8162704	3.5924961
C	4.2737217	-13.1121177	3.7626268
C	3.5922887	-14.0961316	4.5120821
C	4.1205868	-15.3719069	4.6462581

C	5.3363170	-15.7029044	4.0281179
C	6.0271763	-14.7280742	3.2930152
C	5.5098511	-13.4497439	3.1636724
C	-0.0017595	1.2752219	1.5133365
O	0.8332072	2.0488461	1.0049411
O	-1.2133184	1.6432445	1.8867588
C	5.9121300	-17.0656565	4.0885278
O	6.8919660	-17.4366234	3.4632578
O	5.2257823	-17.8978055	4.9247469
H	3.9592226	-6.3905759	1.8629983
H	0.9797279	-9.6697409	4.1083247
H	-1.0075919	-3.0496700	2.9599837
H	-1.5999705	-0.6567201	2.5870549
H	2.2930543	0.0625583	0.9172372
H	2.8957469	-2.3372631	1.2705372
H	2.6437831	-13.8381848	4.9823697
H	3.5864924	-16.1304169	5.2160166
H	6.9705992	-15.0061117	2.8240440
H	6.0408179	-12.6862622	2.5960356
H	-1.3761412	2.6409735	1.6499254
H	5.6529767	-18.7746203	4.8306288
C	-0.9326311	-8.0640382	4.4103693
H	-1.1637198	-8.9143298	3.7474560
H	-0.5258805	-8.4440438	5.3617345
H	-1.8469532	-7.4937822	4.6055489
C	5.8326892	-8.0038519	1.4744465
H	5.3909547	-7.5685993	0.5639437
H	6.1187102	-7.1934111	2.1648260
H	6.7207691	-8.5869320	1.2083785
C	-3.3707030	7.1218400	-2.3573095
C	-3.7842016	8.4348761	-2.5494018
C	-2.9129005	9.3609380	-3.1823216

C	-1.6246534	8.9364913	-3.5689402
C	-1.2030145	7.6293036	-3.3531061
C	-2.0879039	6.6942376	-2.7523723
C	-1.6905059	5.3627458	-2.5293515
C	-3.3425601	10.6800593	-3.4163646
O	0.0349764	7.1543097	-3.6690575
O	-4.9956094	8.9228262	-2.1596870
C	-1.3773207	4.2002559	-2.3199128
C	-1.0281593	2.8524522	-2.0901464
C	0.2509680	2.3673547	-2.4468811
C	0.5793605	1.0363404	-2.2405676
C	-0.3561963	0.1551593	-1.6746961
C	-1.6237766	0.6326022	-1.3056456
C	-1.9579943	1.9625012	-1.5048173
C	-3.7509571	11.8140552	-3.6165222
C	-4.2718295	13.1120969	-3.7986279
C	-3.5727717	14.0909453	-4.5384738
C	-4.0900956	15.3704348	-4.6802052
C	-5.3117881	15.7103522	-4.0788262
C	-6.0206784	14.7402549	-3.3547217
C	-5.5148730	13.4579449	-3.2188768
C	-0.0342101	-1.2707978	-1.4460377
O	-0.8678271	-2.0447731	-0.9358670
O	1.1766608	-1.6381836	-1.8221777
C	-5.8745518	17.0784207	-4.1442765
O	-6.8552955	17.4591065	-3.5262953
O	-5.1741859	17.9032741	-4.9759528
H	-4.0196303	6.3922941	-1.8806734
H	-0.9697353	9.6701253	-4.0316983
H	0.9725897	3.0564429	-2.8834993
H	1.5648005	0.6628578	-2.5134798
H	-2.3326081	-0.0609027	-0.8556276

H	-2.9367753	2.3383619	-1.2106317
H	-2.6191028	13.8263692	-4.9943349
H	-3.5422493	16.1253874	-5.2415966
H	-6.9686618	15.0251427	-2.8992469
H	-6.0597190	12.6980530	-2.6596099
H	1.3390549	-2.6370637	-1.5896541
H	-5.5930199	18.7844562	-4.8849865
C	0.9550847	8.0667269	-4.2610398
H	0.5820931	8.4440907	-5.2271823
H	1.1608507	8.9188080	-3.5922261
H	1.8767056	7.4975628	-4.4219636
C	-5.9081332	8.0049587	-1.5616407
H	-6.1681716	7.1890603	-2.2558015
H	-5.4971154	7.5769094	-0.6334208
H	-6.8064360	8.5871695	-1.3302937
C	3.7240145	-13.5365808	0.2269213
C	3.9702516	-14.8660568	0.5552921
C	3.0123790	-15.5917835	1.3119065
C	1.8337252	-14.9418232	1.7305625
C	1.5963119	-13.6089112	1.4185238
C	2.5459644	-12.8877707	0.6458130
C	2.3044927	-11.5399182	0.3227372
C	3.2575558	-16.9306865	1.6684309
O	0.4947375	-12.9090871	1.8131352
O	5.0928346	-15.5513660	0.2117460
C	2.0653856	-10.3695158	0.0681994
C	1.7495998	-9.0167334	-0.1757311
C	0.5108654	-8.4935696	0.2596525
C	0.1954644	-7.1602147	0.0492391
C	1.1107591	-6.3098584	-0.5894986
C	2.3410028	-6.8210733	-1.0303335
C	2.6568083	-8.1568821	-0.8354060

C	3.4849863	-18.0842961	2.0014354
C	3.7949021	-19.3934658	2.4265766
C	2.8470004	-20.1708788	3.1300714
C	3.1737665	-21.4409205	3.5835931
C	4.4547618	-21.9688535	3.3499597
C	5.4009291	-21.2030610	2.6474851
C	5.0804022	-19.9351985	2.1868813
C	0.8054586	-4.8766744	-0.8041071
O	1.6634720	-4.0968821	-1.2652340
O	-0.4206121	-4.5165356	-0.4838058
C	4.8568223	-23.3146725	3.8207179
O	5.9554238	-23.8168833	3.6572729
O	3.8523525	-23.9700137	4.4813041
H	4.4414095	-12.9593920	-0.3513014
H	1.1240779	-15.5161132	2.3208945
H	-0.1881572	-9.1573431	0.7663324
H	-0.7536732	-6.7550439	0.3947540
H	3.0366781	-6.1465625	-1.5287575
H	3.6074333	-8.5603274	-1.1831044
H	1.8571046	-19.7558230	3.3172788
H	2.4397948	-22.0339722	4.1267448
H	6.3904141	-21.6268658	2.4764267
H	5.8178752	-19.3326987	1.6593859
H	-0.5652251	-3.5016226	-0.6705804
H	4.2361352	-24.8333064	4.7383454
C	-0.4673618	-13.5997661	2.6045263
H	-0.8960482	-14.4576445	2.0608454
H	-0.0286828	-13.9521238	3.5526859
H	-1.2576571	-12.8717690	2.8155005
C	6.1083470	-14.8425071	-0.4933586
H	5.7492103	-14.4998650	-1.4775219
H	6.4656935	-13.9760704	0.0868085

H 6.9276201 -15.5557019 -0.6290577

(1·2)2

C 2.6781908 -12.9396902 -0.7593766

C 2.7657667 -11.5541929 -0.8178094

C 1.5890085 -10.7607475 -0.8788404

C 0.3511324 -11.4256996 -0.8332233

C 0.2643520 -12.8076147 -0.7450982

C 1.4401637 -13.6053866 -0.7349461

C 1.3178809 -15.0462229 -0.6901629

C 2.3247745 -15.9499920 -0.6263377

C 2.1468861 -17.3915825 -0.5661934

C 1.6972100 -9.3221654 -0.9699614

C 0.6688916 -8.4426951 -1.0550331

C 0.7936251 -7.0008837 -1.1440752

C 2.0273408 -6.3179990 -1.1751154

C 2.0416833 -4.9355237 -1.2584335

N 0.9240265 -4.1903533 -1.3191805

C -0.3647973 -6.2028605 -1.1957927

C 0.9023838 -18.0371610 -0.7108421

C 0.8288058 -19.4180609 -0.5836534

N 1.8837526 -20.2138175 -0.3253937

C 3.0731773 -19.6022679 -0.2101020

C 3.2531475 -18.2259428 -0.3258870

O -0.9236580 -13.4845849 -0.6508365

O 3.9533363 -10.8718702 -0.8364277

H 3.5851663 -13.5387643 -0.7350970

H -0.5562001 -10.8276754 -0.8481482

H 0.2923963 -15.4138699 -0.7003642

H 3.3625835 -15.6097358 -0.5796577

H 2.7169118 -8.9381146 -0.9719606

H	-0.3590797	-8.8129884	-1.0594374
H	2.9715237	-6.8598539	-1.1344226
H	2.9850580	-4.3855002	-1.2798015
H	-1.3497931	-6.6698339	-1.1646784
H	-0.0047627	-17.4701236	-0.9188396
H	-0.1356349	-19.9238985	-0.6860172
H	3.9285415	-20.2488399	0.0028389
H	4.2467908	-17.7942770	-0.1962266
C	-2.1197468	-12.7144199	-0.6228096
H	-2.1319996	-12.0277990	0.2400586
H	-2.2495065	-12.1349175	-1.5518436
H	-2.9385952	-13.4355962	-0.5273027
C	5.1502210	-11.6338050	-0.7212472
H	5.1698568	-12.2048171	0.2213087
H	5.2713200	-12.3263542	-1.5702286
H	5.9704096	-10.9075591	-0.7296186
C	-2.2026865	7.8320636	-2.2007093
C	-2.2876187	9.2180693	-2.2913865
C	-1.1009650	9.9986119	-2.3070372
C	0.1432603	9.3491695	-2.1845201
C	0.2259378	7.9648969	-2.0717209
C	-0.9578903	7.1796673	-2.1056623
C	-0.8831238	5.7764450	-2.0296098
C	-1.1850309	11.3975610	-2.4226474
O	1.3916017	7.2715506	-1.9304112
O	-3.4561354	9.9135895	-2.3808801
C	-0.8134290	4.5588810	-1.9583078
C	-0.6995740	3.1542555	-1.8577107
C	0.5718842	2.5548414	-1.7289572
C	0.6913928	1.1750374	-1.6405469
C	-0.4494734	0.3602457	-1.6697716
C	-1.7160935	0.9508170	-1.7822717

C	-1.8458358	2.3278734	-1.8776839
C	-1.2958959	12.6108986	-2.5113831
C	-1.5052059	14.0026648	-2.6015539
C	-0.4360867	14.9213683	-2.5117454
C	-0.6704035	16.2871675	-2.6006866
C	-1.9744797	16.7723967	-2.7804445
C	-3.0434545	15.8657217	-2.8566109
C	-2.8174920	14.5018842	-2.7683883
C	-0.3568784	-1.1287566	-1.5766323
O	-1.3560660	-1.8484565	-1.5703813
O	0.8936849	-1.5757243	-1.5056622
C	-2.2848190	18.2186214	-2.8741424
O	-3.3987929	18.6807943	-3.0567939
O	-1.1828899	19.0073540	-2.7237886
H	-3.0964370	7.2132218	-2.2079457
H	1.0384607	9.9660961	-2.1879212
H	1.4552767	3.1910178	-1.6941324
H	1.6704730	0.7117342	-1.5349390
H	-2.5892438	0.2986963	-1.7938262
H	-2.8292984	2.7887098	-1.9697834
H	0.5749139	14.5431415	-2.3659544
H	0.1567907	16.9911151	-2.5271670
H	-4.0492953	16.2629812	-2.9919850
H	-3.6432406	13.7942128	-2.8344200
H	0.8920684	-2.6258539	-1.4283444
H	-1.5145407	19.9290319	-2.7106330
C	2.6007666	8.0217279	-1.8977625
H	2.7543116	8.5805897	-2.8355392
H	2.6138435	8.7270560	-1.0491843
H	3.4039662	7.2878311	-1.7743613
C	-4.6668166	9.1678291	-2.2999324
H	-4.7533438	8.4459289	-3.1281536

H	-4.7413742	8.6349718	-1.3380672
H	-5.4753351	9.9025547	-2.3765791
C	-0.2606829	-4.8209888	-1.2831682
H	-1.1324076	-4.1642106	-1.3308144
C	-2.7080923	12.9249431	0.7045215
C	-2.7853716	11.5389232	0.7642108
C	-1.6044207	10.7557602	0.8644822
C	-0.3712993	11.4312058	0.8546979
C	-0.2938464	12.8137585	0.7669055
C	-1.4757066	13.6012822	0.7184564
C	-1.3637616	15.0428802	0.6740815
C	-2.3761900	15.9394887	0.5986671
C	-2.2077208	17.3820830	0.5366613
C	-1.7037821	9.3166862	0.9589935
C	-0.6708363	8.4458348	1.0723333
C	-0.7851115	7.0034929	1.1692006
C	-2.0134720	6.3105091	1.1856803
C	-2.0176787	4.9285297	1.2787826
N	-0.8949615	4.1931203	1.3620116
C	0.3789695	6.2155528	1.2454938
C	-0.9684843	18.0368467	0.6857538
C	-0.9041758	19.4179799	0.5558492
N	-1.9635073	20.2057378	0.2910959
C	-3.1479873	19.5854538	0.1716409
C	-3.3186222	18.2081364	0.2892545
O	0.8905620	13.5014672	0.7122562
O	-3.9665406	10.8458214	0.7453775
H	-3.6189496	13.5157539	0.6459772
H	0.5403132	10.8413118	0.9014863
H	-0.3411967	15.4179155	0.6968663
H	-3.4112807	15.5921136	0.5448084
H	-2.7203525	8.9248203	0.9382444

H	0.3537868	8.8247214	1.0961736
H	-2.9612318	6.8444619	1.1268673
H	-2.9566642	4.3707821	1.2896688
H	1.3605115	6.6904951	1.2273098
H	-0.0581251	17.4768491	0.8989640
H	0.0561907	19.9309186	0.6610281
H	-4.0070522	20.2253213	-0.0464818
H	-4.3085217	17.7690471	0.1559584
C	2.0942296	12.7427756	0.7287837
H	2.1956636	12.1660981	1.6630208
H	2.1446358	12.0549720	-0.1317339
H	2.9090150	13.4718152	0.6616776
C	-5.1645751	11.5943415	0.5704330
H	-5.3303333	12.2930733	1.4066359
H	-5.1477407	12.1560513	-0.3776969
H	-5.9771584	10.8598051	0.5493837
C	2.2508401	-7.8405406	2.1822174
C	2.3207188	-9.2282560	2.2598817
C	1.1254935	-9.9945710	2.3026917
C	-0.1132747	-9.3293784	2.2141083
C	-0.1816367	-7.9434473	2.1119522
C	1.0121241	-7.1724886	2.1231260
C	0.9496033	-5.7679614	2.0591472
C	1.1960595	-11.3947826	2.4119298
O	-1.3418508	-7.2351868	2.0036510
O	3.4821601	-9.9392335	2.3105880
C	0.8822689	-4.5497099	1.9971714
C	0.7649909	-3.1446599	1.9044308
C	-0.5092393	-2.5493862	1.7831926
C	-0.6343507	-1.1698705	1.6987328
C	0.5035277	-0.3507384	1.7261050
C	1.7727004	-0.9369297	1.8333192

C	1.9080754	-2.3139414	1.9234076
C	1.2959132	-12.6094272	2.4961502
C	1.4927631	-14.0033878	2.5810271
C	0.4137276	-14.9116966	2.5053547
C	0.6360892	-16.2797999	2.5895798
C	1.9375850	-16.7777928	2.7515797
C	3.0161582	-15.8814136	2.8138882
C	2.8023951	-14.5154104	2.7289768
C	0.4045305	1.1382350	1.6354853
O	1.4002129	1.8627334	1.6351228
O	-0.8478670	1.5793123	1.5590415
C	2.2352034	-18.2269431	2.8409285
O	3.3463494	-18.6996021	3.0135030
O	1.1248490	-19.0056013	2.6992748
H	3.1522973	-7.2329085	2.1709212
H	-1.0157901	-9.9351789	2.2373401
H	-1.3903393	-3.1888109	1.7501286
H	-1.6155865	-0.7101851	1.5972436
H	2.6436132	-0.2817449	1.8442627
H	2.8936427	-2.7712572	2.0104503
H	-0.5954614	-14.5237121	2.3738705
H	-0.1988086	-16.9756219	2.5269713
H	4.0197197	-16.2885262	2.9358533
H	3.6357691	-13.8157125	2.7829382
H	-0.8517451	2.6291798	1.4792652
H	1.4481825	-19.9302045	2.6833343
C	-2.5603652	-7.9708821	1.9890717
H	-2.5982324	-8.6693656	1.1357037
H	-2.7026650	-8.5350689	2.9254196
H	-3.3569984	-7.2266262	1.8866630
C	4.6996122	-9.2089898	2.1961370
H	4.7501351	-8.6694356	1.2365376

H 4.8227545 -8.4952614 3.0267173

H 5.5001155 -9.9551487 2.2403098

C 0.2849028 4.8333828 1.3405437

H 1.1611625 4.1841705 1.4058488

Cartesian coordinates of GFN2-XTB

1_polymer_HT

C -2.9180 -6.0120 -30.6520

C -2.4370 -6.2260 -36.2680

C -2.5360 -6.1150 -37.4680

C -2.7010 -5.9760 -38.8530

C -1.6040 -5.6860 -39.6890

C -3.9910 -6.0640 -39.3790

H -4.8000 -6.2400 -38.6880

O -4.0410 -5.9900 -30.1560

O -1.8160 -5.9750 -29.9520

H -2.0000 -5.9350 -28.9580

C -3.4330 -5.5180 -49.8720

C -3.2580 -5.8620 -44.1900

C -3.2480 -5.7920 -42.9800

C -3.1050 -5.7820 -41.5880

C -4.2250 -5.9130 -40.7360

C -1.8210 -5.6390 -41.0540

H -1.0080 -5.4820 -41.7440

O -2.4200 -5.5600 -50.5620

O -4.6290 -5.2670 -50.3610

H -4.6080 -5.1920 -51.3660

C -7.7830 -5.2990 -41.2450

C -7.6750 -4.8160 -42.5360

H -6.7010 -4.7910 -42.9930

C -8.8130 -4.3900 -43.2150

C -10.0630 -4.4440 -42.5860

C -10.1610 -4.9170 -41.2680

C -9.0170 -5.3510 -40.6110

H -9.0740 -5.7270 -39.6030

C -7.6600 -4.0380 -45.2610

H -6.8210 -3.4980 -44.8000

H -7.3980 -5.1050 -45.3200

C -7.9010 -3.4850 -46.6640

H -8.1770 -2.4330 -46.5850

H -6.9510 -3.5490 -47.2000

C -8.9860 -4.2380 -47.4390

H -8.9240 -5.3020 -47.2020

H -8.8010 -4.1290 -48.5110

C -10.3890 -3.7250 -47.1200

H -10.5240 -3.7040 -46.0390

H -10.4860 -2.7000 -47.4880

C -11.4760 -4.5900 -47.7530

H -11.4170 -5.6010 -47.3400

H -11.3060 -4.6650 -48.8310

C -12.8710 -4.0230 -47.4970

H -12.9800 -3.8180 -46.4300

H -12.9770 -3.0730 -48.0270

C -13.9770 -4.9770 -47.9410

H -13.8860 -5.9160 -47.3880

H -13.8530 -5.2080 -49.0020

C -15.3670 -4.3890 -47.7110

H -15.4700 -4.1090 -46.6600

H -15.4760 -3.4800 -48.3060

C -16.4770 -5.3700 -48.0820

H -16.3810 -6.2720 -47.4720

H -16.3620 -5.6660 -49.1260

C -17.8660 -4.7690 -47.8800

H -17.9610 -3.8710 -48.4950

H -17.9820 -4.4660 -46.8370

C -18.9770 -5.7480 -48.2480

H -18.8520 -6.0620 -49.2860

H -18.8940 -6.6420 -47.6240

C -20.3590 -5.1320 -48.0700

H -20.4650 -4.2480 -48.6970

H -21.1350 -5.8420 -48.3450

H -20.5150 -4.8340 -47.0360

C -12.1620 -4.9600 -43.5570

H -12.0330 -5.2940 -44.5970

H -12.0490 -5.8310 -42.9040

C -13.5460 -4.3480 -43.3700

H -13.6500 -4.0460 -42.3280

H -13.6180 -3.4500 -43.9870

C -14.6450 -5.3360 -43.7480

H -14.5420 -6.2400 -43.1430

H -14.5250 -5.6270 -44.7940

C -16.0420 -4.7530 -43.5460

H -16.1730 -4.4830 -42.4960

H -16.1410 -3.8380 -44.1350

C -17.1310 -5.7400 -43.9580

H -17.0270 -6.6580 -43.3740

H -16.9960 -6.0040 -45.0080

C -18.5350 -5.1740 -43.7590

H -18.6850 -4.9370 -42.7040

H -18.6310 -4.2420 -44.3220

C -19.6110 -6.1530 -44.2200

H -19.5220 -7.0830 -43.6530

H -19.4480 -6.3960 -45.2720

C -21.0200 -5.5910 -44.0480

H -21.2060 -5.3960 -42.9900

H -21.0960 -4.6360 -44.5750

C -22.0840 -6.5460 -44.5830

H -22.0120 -7.5000 -44.0550

H -21.8930 -6.7420 -45.6410

C -23.4950 -5.9850 -44.4220

H -23.6910 -5.7980 -43.3640

H -23.5640 -5.0280 -44.9460

C -24.5580 -6.9350 -44.9700

H -24.4900 -7.8930 -44.4490

H -24.3630 -7.1200 -46.0290

C -25.9630 -6.3710 -44.8050

H -26.0590 -5.4230 -45.3310

H -26.7030 -7.0610 -45.2050

H -26.1860 -6.2030 -43.7540

C -11.5670 -5.2260 -39.3610

H -11.3540 -6.2890 -39.1810

H -10.8780 -4.6170 -38.7640

C -13.0080 -4.9040 -38.9780

H -13.0960 -4.9300 -37.8900

H -13.2310 -3.8890 -39.3120

C -14.0030 -5.8800 -39.6000

H -13.8420 -5.9080 -40.6780

H -13.8190 -6.8820 -39.2070

C -15.4460 -5.4770 -39.3130

H -15.6350 -4.4870 -39.7350

H -15.5950 -5.4040 -38.2320

C -16.4450 -6.4730 -39.8940

H -16.2860 -7.4530 -39.4380

H -16.2680 -6.5760 -40.9660

C -17.8890 -6.0350 -39.6630

H -18.0470 -5.0590 -40.1280

H -18.0670 -5.9190 -38.5910

C -18.8900 -7.0350 -40.2350

H -18.6970 -7.1680 -41.3010

H -18.7500 -8.0040 -39.7510

C -20.3320 -6.5750 -40.0390

H -20.4710 -5.6090 -40.5330

H -20.5240 -6.4260 -38.9740

C -21.3380 -7.5770 -40.5980

H -21.2060 -8.5400 -40.0970

H -21.1430 -7.7290 -41.6610

C -22.7770 -7.1050 -40.4110

H -22.9770 -6.9730 -39.3450

H -22.9020 -6.1310 -40.8920

C -23.7890 -8.0850 -40.9950

H -23.6680 -9.0590 -40.5140

H -23.5890 -8.2170 -42.0600

C -25.2220 -7.6020 -40.8050

H -25.3620 -6.6330 -41.2800

H -25.9260 -8.3030 -41.2460

H -25.4540 -7.4990 -39.7480

O -11.1650 -3.9870 -43.2700

O -8.8240 -3.8920 -44.4810

O -11.4110 -4.9200 -40.7320

O -5.4450 -5.8690 -41.3140

C 1.7310 -4.4690 -38.8650

C 1.5510 -4.3710 -37.4960

H 0.5530 -4.4430 -37.0980

C 2.6530 -4.2290 -36.6630

C 3.9520 -4.1900 -37.1990

C 4.1250 -4.2690 -38.5900

C 3.0030 -4.3810 -39.4070

H 3.1150 -4.4270 -40.4780

C 1.3890 -3.7330 -34.7030

H 0.5560 -4.3960 -34.9690

H 1.1470 -2.7230 -35.0690

C 1.5410 -3.7240 -33.1820

H 1.7030 -4.7460 -32.8370

H 0.5880 -3.3760 -32.7740

C 2.6880 -2.8420 -32.6770

H 2.7480 -1.9390 -33.2890

H 2.4790 -2.5320 -31.6500

C 4.0320 -3.5690 -32.7170

H 4.1860 -3.9730 -33.7170

H 4.0030 -4.4040 -32.0130

C 5.2010 -2.6540 -32.3640

H 5.2150 -1.8070 -33.0550

H 5.0690 -2.2500 -31.3560

C 6.5330 -3.3950 -32.4520

H 6.6110 -3.8630 -33.4360

H 6.5550 -4.1910 -31.7040

C 7.7280 -2.4690 -32.2420

H 7.7130 -1.6820 -33.0010

H 7.6430 -1.9790 -31.2680

C 9.0580 -3.2170 -32.3170

H 9.1330 -3.7230 -33.2810

H 9.0840 -3.9840 -31.5400

C 10.2520 -2.2810 -32.1440

H 10.2450 -1.5340 -32.9430

H 10.1570 -1.7430 -31.1970

C 11.5810 -3.0330 -32.1660

H 11.5920 -3.7680 -31.3580

H 11.6740 -3.5770 -33.1080

C 12.7750 -2.0950 -32.0080

H 12.6700 -1.5280 -31.0790

H 12.7790 -1.3730 -32.8280

C 14.0950 -2.8550 -31.9910

H 14.1190 -3.5590 -31.1630

H 14.9330 -2.1690 -31.8830

H 14.2250 -3.4120 -32.9160

C 6.0440 -3.2440 -36.4680

H 6.0010 -2.5310 -35.6310

H 5.9340 -2.6870 -37.4010

C 7.3790 -3.9840 -36.4420

H 7.4590 -4.5940 -37.3420

H 7.3780 -4.6580 -35.5820

C 8.5580 -3.0210 -36.3430

H 8.5310 -2.3230 -37.1840

H 8.4660 -2.4330 -35.4270

C 9.8980 -3.7530 -36.3380

H 10.0070 -4.3150 -37.2670

H 9.9120 -4.4730 -35.5160

C 11.0730 -2.7920 -36.1830

H 11.0520 -2.0580 -36.9930

H 10.9690 -2.2450 -35.2440

C 12.4160 -3.5180 -36.1960

H 12.5350 -4.0420 -37.1470

H 12.4270 -4.2700 -35.4030

C 13.5880 -2.5600 -35.9960

H 13.5890 -1.8160 -36.7960

H 13.4600 -2.0270 -35.0520

C 14.9300 -3.2870 -35.9830

H 15.0770 -3.7950 -36.9390

H 14.9200 -4.0530 -35.2040

C 16.0960 -2.3330 -35.7340

H 16.1250 -1.5840 -36.5290

H 15.9360 -1.8070 -34.7900

C 17.4350 -3.0640 -35.6810

H 17.5890 -3.6060 -36.6170

H 17.4120 -3.8010 -34.8740

C 18.6040 -2.1090 -35.4570

H 18.6380 -1.3820 -36.2720

H 18.4440 -1.5560 -34.5280

C 19.9350 -2.8470 -35.3820

H 19.9330 -3.5560 -34.5570

H 20.7540 -2.1470 -35.2320

H 20.1200 -3.3970 -36.3030

C 5.6240 -4.5760 -40.4300

H 5.4060 -3.7050 -41.0640

H 4.9720 -5.4060 -40.7270

C 7.0760 -5.0070 -40.5990

H 7.1980 -5.3950 -41.6120

H 7.2760 -5.8200 -39.8980

C 8.0660 -3.8720 -40.3570

H 7.8900 -3.4550 -39.3650

H 7.9000 -3.0810 -41.0900

C 9.5050 -4.3670 -40.4550

H 9.6670 -5.1580 -39.7180

H 9.6700 -4.8040 -41.4430

C 10.5150 -3.2470 -40.2230

H 10.3660 -2.4680 -40.9730

H 10.3410 -2.8060 -39.2400

C 11.9540 -3.7470 -40.2900

H 12.1030 -4.5230 -39.5340

H 12.1360 -4.2050 -41.2650

C 12.9560 -2.6200 -40.0620

H 12.7470 -2.1420 -39.1020

H 12.8300 -1.8640 -40.8400

C 14.3980 -3.1180 -40.0690

H 14.5240 -3.8670 -39.2830

H 14.6070 -3.6080 -41.0230

C 15.3950 -1.9840 -39.8500

H 15.2870 -1.2480 -40.6500

H 15.1650 -1.4820 -38.9070

C 16.8370 -2.4820 -39.8130

H 17.0740 -2.9790 -40.7570

H 16.9420 -3.2220 -39.0160

C 17.8310 -1.3480 -39.5790

H 17.7380 -0.6150 -40.3840

H 17.5840 -0.8420 -38.6420

C 19.2660 -1.8550 -39.5160

H 19.3810 -2.5740 -38.7080

H 19.9580 -1.0330 -39.3440

H 19.5390 -2.3440 -40.4490

O 4.9730 -4.1580 -36.2870

O 2.5910 -4.1670 -35.3030

O 5.4030 -4.2500 -39.0680

O -0.4100 -5.4750 -39.0820

C -2.4980 -6.2470 -34.8660

C -1.3560 -6.2730 -34.0610

C -3.7640 -6.1880 -34.2750

C -1.4770 -6.1850 -32.6920

H -0.3840 -6.3600 -34.5250

C -3.8810 -6.1160 -32.9100

H -4.6360 -6.1910 -34.9090

C -2.7400 -6.1000 -32.1090

H -0.6040 -6.1770 -32.0560

H -4.8460 -6.0640 -32.4360

C -3.2610 -5.7530 -45.5750

C -2.2280 -5.9780 -46.3390

C -4.4580 -5.4390 -46.1670

C -2.2890 -5.8710 -47.7570

H -1.3670 -6.2270 -45.8660

C -4.5320 -5.3410 -47.7090

H -5.3170 -5.2610 -45.6760

C -3.3740 -5.5550 -48.4160

H -1.4670 -6.0290 -48.3180

H -5.4290 -5.1270 -48.1120

C -6.5670 -5.7340 -40.4680

H -6.7730 -6.6900 -39.9660

H -6.3600 -4.9740 -39.6990

C 0.5550 -4.7030 -39.7740

H 0.8990 -5.2250 -40.6770

H 0.1030 -3.7460 -40.0780

C -3.5310 -5.2330 -53.6700

C -3.7980 -4.9690 -59.3100

C -3.7540 -4.8810 -60.5150

C -3.8100 -4.8130 -61.9160

C -2.6330 -4.8080 -62.6920

C -5.0640 -4.7460 -62.5280

H -5.9310 -4.7130 -61.8890

O -4.5340 -5.1170 -52.9720

O -2.3370 -5.4510 -53.1790

H -2.3340 -5.5040 -52.1680

C -4.0240 -5.0640 -67.2840

C -4.0120 -4.9250 -66.0910

C -4.0110 -4.8270 -64.6810

C -5.1840 -4.7320 -63.9060

C -2.7600 -4.8360 -64.0740

H -1.8960 -4.8690 -64.7170

C -8.6510 -4.1240 -64.8630

C -8.5270 -4.5510 -66.1720

H -7.6170 -5.0380 -66.4740

C -9.5740 -4.3590 -67.0660

C -10.7610 -3.7570 -66.6300

C -10.8820 -3.3370 -65.2970

C -9.8170 -3.5150 -64.4230

H -9.8840 -3.1820 -63.3990

C -8.5040 -5.4770 -68.9520

H -7.5430 -4.9560 -68.8400

H -8.4280 -6.4490 -68.4450

C -8.8010 -5.6710 -70.4380

H -8.8020 -4.6960 -70.9270

H -7.9820 -6.2600 -70.8550

C -10.1370 -6.3700 -70.7060

H -10.3100 -7.1230 -69.9340

H -10.0830 -6.8910 -71.6660

C -11.3100 -5.3920 -70.7380

H -11.3120 -4.8070 -69.8210

H -11.1720 -4.6990 -71.5720

C -12.6510 -6.1040 -70.8940

H -12.8010 -6.7790 -70.0470

H -12.6410 -6.7160 -71.8000

C -13.8120 -5.1150 -70.9620

H -13.7800 -4.4660 -70.0850

H -13.6950 -4.4810 -71.8440

C -15.1660 -5.8180 -71.0230

H -15.2750 -6.4670 -70.1500

H -15.2040 -6.4560 -71.9090

C -16.3280 -4.8290 -71.0620

H -16.2890 -4.1920 -70.1760

H -16.2240 -4.1830 -71.9370

C -17.6800 -5.5370 -71.1160

H -17.7740 -6.2020 -70.2530

H -17.7250 -6.1600 -72.0130

C -18.8490 -4.5540 -71.1230

H -18.7530 -3.8870 -71.9820

H -18.8080 -3.9400 -70.2210

C -20.1980 -5.2670 -71.1870

H -20.2450 -5.8670 -72.0980

H -20.2840 -5.9510 -70.3390

C -21.3610 -4.2830 -71.1660

H -21.3060 -3.6100 -72.0180

H -22.3120 -4.8110 -71.2070

H -21.3380 -3.6870 -70.2570

C -12.9460 -4.3650 -67.3110

H -12.9300 -5.2040 -68.0220

H -12.9390 -4.7800 -66.2980

C -14.2010 -3.5250 -67.5220

H -14.2330 -2.7500 -66.7550

H -14.1330 -3.0300 -68.4910

C -15.4580 -4.3860 -67.4660

H -15.4910 -4.9230 -66.5140

H -15.4140 -5.1360 -68.2600

C -16.7330 -3.5610 -67.6240

H -16.8150 -2.8580 -66.7930

H -16.6740 -2.9760 -68.5450

C -17.9750 -4.4470 -67.6670

H -18.0100 -5.0630 -66.7650

H -17.9040 -5.1230 -68.5220

C -19.2650 -3.6380 -67.7710

H -19.3610 -2.9940 -66.8940

H -19.2170 -2.9910 -68.6500

C -20.4910 -4.5410 -67.8740

H -20.5310 -5.1950 -67.0000

H -20.3950 -5.1770 -68.7560

C -21.7920 -3.7490 -67.9670

H -21.9120 -3.1410 -67.0660

H -21.7410 -3.0640 -68.8170

C -23.0040 -4.6630 -68.1260

H -23.0550 -5.3430 -67.2720

H -22.8790 -5.2710 -69.0250

C -24.3100 -3.8810 -68.2250

H -24.4360 -3.2710 -67.3270

H -24.2610 -3.2010 -69.0790

C -25.5190 -4.8000 -68.3830

H -25.5640 -5.4850 -67.5330

H -25.3970 -5.4030 -69.2860

C -26.8220 -4.0160 -68.4700

H -26.8060 -3.3440 -69.3260

H -27.6690 -4.6900 -68.5780

H -26.9720 -3.4200 -67.5720

C -12.2860 -2.3600 -63.6260

H -12.1210 -3.1950 -62.9330

H -11.5890 -1.5510 -63.3670

C -13.7270 -1.8700 -63.5210

H -13.8470 -1.3240 -62.5840

H -13.9120 -1.1780 -64.3440

C -14.7260 -3.0220 -63.5910

H -14.5490 -3.5880 -64.5060

H -14.5650 -3.6930 -62.7450

C -16.1670 -2.5210 -63.5750

H -16.3240 -1.8490 -64.4220

H -16.3400 -1.9430 -62.6630

C -17.1720 -3.6670 -63.6490

H -17.0360 -4.3260 -62.7890

H -16.9800 -4.2550 -64.5490

C -18.6120 -3.1630 -63.6790

H -18.7410 -2.4970 -64.5360

H -18.8110 -2.5770 -62.7780

C -19.6200 -4.3050 -63.7750

H -19.4040 -4.9000 -64.6650

H -19.5120 -4.9590 -62.9070

C -21.0570 -3.7950 -63.8510

H -21.1570 -3.1350 -64.7150

H -21.2820 -3.2040 -62.9590

C -22.0670 -4.9330 -63.9680

H -21.9810 -5.5850 -63.0950

H -21.8320 -5.5330 -64.8500

C -23.4990 -4.4180 -64.0790

H -23.7410 -3.8280 -63.1910

H -23.5770 -3.7570 -64.9450

C -24.5130 -5.5500 -64.2220

H -24.4350 -6.2170 -63.3590

H -24.2740 -6.1370 -65.1120

C -25.9380 -5.0240 -64.3290

H -26.0370 -4.3650 -65.1890

H -26.6440 -5.8440 -64.4430

H -26.2060 -4.4630 -63.4360

O -11.7850 -3.5730 -67.5320

O -9.5450 -4.7170 -68.3740

O -12.0750 -2.7780 -64.9590

O -6.3470 -4.6510 -64.5940

C 0.9490 -4.4300 -61.9370

C 0.9380 -4.7000 -60.5790

H -0.0000 -4.9320 -60.1020

C 2.1320 -4.6640 -59.8640

C 3.3350 -4.3660 -60.5180

C 3.3380 -4.1000 -61.8960

C 2.1350 -4.1210 -62.5890

H 2.1110 -3.9040 -63.6440

C 1.1400 -5.2020 -57.7480

H 0.5890 -6.0590 -58.1640

H 0.4690 -4.3310 -57.7260

C 1.6030 -5.5330 -56.3290

H 2.1730 -6.4610 -56.3510

H 0.7120 -5.6990 -55.7210

C 2.4730 -4.4390 -55.7040

H 2.1380 -3.4620 -56.0590

H 2.3490 -4.4630 -54.6180

C 3.9510 -4.6380 -56.0370

H 4.0550 -4.8010 -57.1100

H 4.3050 -5.5320 -55.5190

C 4.8170 -3.4490 -55.6320

H 4.4950 -2.5600 -56.1820

H 4.6870 -3.2380 -54.5670

C 6.2890 -3.7240 -55.9260

H 6.3920 -4.0010 -56.9770

H 6.6160 -4.5780 -55.3280

C 7.1910 -2.5280 -55.6300

H 6.8790 -1.6770 -56.2410

H 7.0750 -2.2350 -54.5840

C 8.6580 -2.8440 -55.9100

H 8.7720 -3.1240 -56.9600

H 8.9600 -3.7030 -55.3070

C 9.5780 -1.6660 -55.6000

H 9.2920 -0.8080 -56.2140

H 9.4500 -1.3730 -54.5550

C 11.0450 -2.0050 -55.8530

H 11.3230 -2.8710 -55.2470

H 11.1770 -2.2810 -56.9010

C 11.9750 -0.8420 -55.5180

H 11.8370 -0.5600 -54.4700

H 11.7040 0.0260 -56.1250

C 13.4370 -1.1960 -55.7600

H 13.7290 -2.0450 -55.1460

H 14.0830 -0.3560 -55.5130

H 13.5970 -1.4580 -56.8030

C 5.2460 -3.2030 -59.7120

H 5.1270 -2.7830 -58.7030

H 4.8650 -2.4740 -60.4320

C 6.7190 -3.4890 -59.9870

H 6.8160 -3.8560 -61.0090

H 7.0490 -4.2840 -59.3150

C 7.5750 -2.2440 -59.7810

H 7.2290 -1.4480 -60.4460

H 7.4500 -1.8890 -58.7550

C 9.0550 -2.5120 -60.0420

H 9.1930 -2.8040 -61.0850

H 9.3850 -3.3470 -59.4200

C 9.9150 -1.2880 -59.7370

H 9.5850 -0.4490 -60.3540

H 9.7720 -1.0030 -58.6920

C 11.3990 -1.5450 -59.9870

H 11.5540 -1.7850 -61.0410

H 11.7180 -2.4120 -59.4030

C 12.2560 -0.3400 -59.6080

H 11.9450 0.5240 -60.1990

H 12.0880 -0.0970 -58.5570

C 13.7440 -0.5930 -59.8320

H 13.9230 -0.7980 -60.8900

H 14.0490 -1.4810 -59.2710

C 14.5950 0.5960 -59.3950

H 14.3000 1.4780 -59.9670

H 14.4040 0.8070 -58.3400

C 16.0870 0.3430 -59.5940

H 16.2810 0.1260 -60.6470

H 16.3840 -0.5380 -59.0180

C 16.9350 1.5360 -59.1610

H 16.6390 2.4160 -59.7370

H 16.7420 1.7510 -58.1080

C 18.4230 1.2790 -59.3600

H 18.7440 0.4200 -58.7750

H 19.0070 2.1430 -59.0510

H 18.6410 1.0770 -60.4070

C 4.6400 -3.8090 -63.8750

H 4.0970 -2.9420 -64.2650

H 4.2050 -4.7230 -64.3010

C 6.1160 -3.7130 -64.2450

H 6.2100 -3.8480 -65.3230

H 6.6550 -4.5250 -63.7500

C 6.7170 -2.3720 -63.8340

H 6.5720 -2.2380 -62.7630

H 6.1810 -1.5730 -64.3480

C 8.2050 -2.2820 -64.1570

H 8.7350 -3.0860 -63.6400

H 8.3570 -2.4280 -65.2290

C 8.8000 -0.9400 -63.7390

H 8.3210 -0.1370 -64.3060

H 8.5890 -0.7680 -62.6810

C 10.3090 -0.8880 -63.9630

H 10.7780 -1.7100 -63.4170

H 10.5240 -1.0360 -65.0240

C 10.9190 0.4320 -63.5000

H 10.6710 0.5920 -62.4490

H 10.4810 1.2550 -64.0710

C 12.4360 0.4490 -63.6650

H 12.8650 -0.3840 -63.1040

H 12.6880 0.3010 -64.7180

C 13.0570 1.7550 -63.1760

H 12.6470 2.5890 -63.7510

H 12.7860 1.9130 -62.1300

C 14.5780 1.7500 -63.3060

H 14.8510 1.5940 -64.3530

H 14.9840 0.9140 -62.7310

C 15.2060 3.0500 -62.8110

H 14.8030 3.8880 -63.3840

H 14.9320 3.2060 -61.7640

C 16.7240 3.0300 -62.9390

H 17.1440 2.2080 -62.3630

H 17.1530 3.9600 -62.5720

H 17.0170 2.9030 -63.9790

O 4.4920 -4.4050 -59.7750

O 2.2710 -4.9220 -58.5380

O 4.5470 -3.8550 -62.4600

O -1.4510 -4.7750 -62.0230

C -3.7530 -5.0500 -57.9080

C -2.5230 -5.2640 -57.2780

C -4.9070 -4.9080 -57.1330

C -2.4450 -5.3110 -55.9070

H -1.6410 -5.3840 -57.8860

C -4.8270 -4.9510 -55.7610

H -5.8610 -4.7750 -57.6210

C -3.5970 -5.1560 -55.1370

H -1.4970 -5.4560 -55.4130

H -5.7100 -4.8270 -55.1530

C -7.5250 -4.3350 -63.8860

H -7.7860 -5.1580 -63.2030

H -7.3700 -3.4210 -63.2950

C -0.2930 -4.5080 -62.7880

H -0.1400 -5.3090 -63.5270

H -0.4240 -3.5600 -63.3320

C -3.5580 -5.7640 -7.7770

C -4.3540 -5.4280 -13.3700

C -4.2910 -5.3020 -14.5720

C -4.2900 -5.2020 -15.9730

C -3.0760 -5.2790 -16.6890

C -5.4960 -5.0200 -16.6550

H -6.3910 -4.9240 -16.0630

O -4.4740 -5.8310 -6.9610

O -2.2920 -5.7870 -7.4310

H -2.1500 -5.8410 -6.4350

C -3.5910 -5.8830 -26.9240

C -4.2670 -5.5010 -21.3370

C -4.3510 -5.3470 -20.1420

C -4.3550 -5.2250 -18.7460

C -5.5430 -4.9720 -18.0360

C -3.1420 -5.3480 -18.0710

H -2.2610 -5.5060 -18.6720

O -2.4740 -5.8580 -27.4190

O -4.6920 -5.9730 -27.6360

H -4.4730 -5.9940 -28.6230

C -8.7170 -3.5820 -19.1770

C -8.2780 -3.2310 -20.4440

H -7.2350 -3.3570 -20.6850

C -9.1870 -2.7680 -21.3910

C -10.5440 -2.6610 -21.0570

C -10.9720 -2.9710 -19.7590

C -10.0510 -3.4300 -18.8250

H -10.3650 -3.6800 -17.8250

C -7.5690 -2.5360 -23.1420

H -6.8760 -1.9310 -22.5390

H -7.2530 -3.5870 -23.0760

C -7.5290 -2.0690 -24.5980

H -7.7680 -1.0050 -24.6350

H -6.5060 -2.1990 -24.9580

C -8.4990 -2.8350 -25.5040

H -8.5800 -3.8660 -25.1520

H -8.0970 -2.8680 -26.5200

C -9.8870 -2.1960 -25.5360

H -10.2320 -2.0440 -24.5140

H -9.8160 -1.2180 -26.0170

C -10.9010 -3.0540 -26.2890

H -10.9900 -4.0210 -25.7850

H -10.5450 -3.2460 -27.3060

C -12.2750 -2.3900 -26.3420

H -12.5610 -2.0880 -25.3320

H -12.2170 -1.4840 -26.9510

C -13.3500 -3.3130 -26.9120

H -13.3970 -4.2250 -26.3110

H -13.0770 -3.6070 -27.9290

C -14.7240 -2.6470 -26.9270

H -14.9890 -2.3480 -25.9110

H -14.6770 -1.7390 -27.5330

C -15.8090 -3.5690 -27.4790

H -15.8700 -4.4680 -26.8600

H -15.5370 -3.8880 -28.4890

C -17.1730 -2.8850 -27.5170

H -17.1030 -1.9750 -28.1190

H -17.4560 -2.5870 -26.5050

C -18.2580 -3.7880 -28.0970

H -17.9720 -4.0990 -29.1050

H -18.3390 -4.6930 -27.4890

C -19.6110 -3.0880 -28.1480

H -19.5520 -2.1900 -28.7600

H -20.3680 -3.7440 -28.5710

H -19.9270 -2.7980 -27.1490

C -12.4160 -3.1760 -22.4070

H -12.1490 -3.5510 -23.4050

H -12.4260 -4.0250 -21.7170

C -13.7960 -2.5270 -22.4460

H -14.0410 -2.1770 -21.4430

H -13.7580 -1.6580 -23.1050

C -14.8530 -3.5130 -22.9350

H -14.8730 -4.3800 -22.2690

H -14.5770 -3.8720 -23.9290

C -16.2460 -2.8930 -22.9950

H -16.5400 -2.5640 -21.9970

H -16.2220 -2.0100 -23.6390

C -17.2810 -3.8780 -23.5310

H -17.3110 -4.7600 -22.8850

H -16.9760 -4.2120 -24.5250

C -18.6780 -3.2680 -23.6150

H -19.0090 -2.9770 -22.6170

H -18.6390 -2.3620 -24.2250

C -19.6860 -4.2390 -24.2230

H -19.7400 -5.1400 -23.6070

H -19.3390 -4.5410 -25.2140

C -21.0790 -3.6270 -24.3470

H -21.4570 -3.3810 -23.3530

H -21.0150 -2.6950 -24.9140

C -22.0570 -4.5700 -25.0440

H -22.1420 -5.4940 -24.4660

H -21.6620 -4.8350 -26.0280

C -23.4400 -3.9470 -25.2090

H -23.8380 -3.6830 -24.2260

H -23.3520 -3.0250 -25.7880

C -24.4160 -4.8880 -25.9100

H -24.5190 -5.8050 -25.3240

H -24.0110 -5.1650 -26.8860

C -25.7880 -4.2520 -26.0950

H -25.7110 -3.3470 -26.6930

H -26.4650 -4.9390 -26.5960

H -26.2200 -3.9890 -25.1320

C -12.7900 -2.9200 -18.2040

H -12.6800 -3.9480 -17.8350

H -12.2260 -2.2480 -17.5450

C -14.2610 -2.5200 -18.2350

H -14.5660 -2.2010 -17.2380

H -14.3580 -1.6590 -18.8980

C -15.1680 -3.6420 -18.7290

H -14.8140 -3.9850 -19.7020

H -15.1170 -4.4850 -18.0350

C -16.6140 -3.1700 -18.8490

H -16.6600 -2.3310 -19.5490

H -16.9560 -2.8010 -17.8790

C -17.5470 -4.2760 -19.3310

H -17.5240 -5.1080 -18.6220

H -17.1920 -4.6510 -20.2930

C -18.9820 -3.7810 -19.4840

H -18.9910 -2.9130 -20.1500

H -19.3590 -3.4490 -18.5140

C -19.9060 -4.8550 -20.0500

H -19.5200 -5.1840 -21.0180

H -19.9100 -5.7200 -19.3840

C -21.3340 -4.3440 -20.2290

H -21.3160 -3.4390 -20.8410

H -21.7470 -4.0720 -19.2550

C -22.2360 -5.3800 -20.8930

H -22.2630 -6.2840 -20.2810

H -21.8150 -5.6520 -21.8640

C -23.6570 -4.8600 -21.0920

H -24.0940 -4.6170 -20.1200

H -23.6230 -3.9360 -21.6760

C -24.5470 -5.8720 -21.8080

H -24.5840 -6.7960 -21.2260

H -24.1090 -6.1100 -22.7790

C -25.9620 -5.3430 -22.0070

H -25.9450 -4.4230 -22.5870

H -26.5730 -6.0700 -22.5370

H -26.4310 -5.1330 -21.0480

O -11.4290 -2.2280 -22.0170

O -8.8860 -2.3940 -22.6600

O -12.3010 -2.8120 -19.5260

O -6.6460 -4.7110 -18.7830

C 0.4640 -4.8230 -15.8970

C 0.5130 -5.1040 -14.5430

H -0.3480 -5.5540 -14.0800

C 1.6560 -4.7800 -13.8190

C 2.7540 -4.1880 -14.4580

C 2.6980 -3.9190 -15.8330

C 1.5390 -4.2230 -16.5350

H 1.4650 -3.9980 -17.5860

C 0.7680 -5.5230 -11.7360

H 0.3890 -6.4530 -12.1830

H -0.0480 -4.7880 -11.7090

C 1.2460 -5.8160 -10.3170

H 2.0320 -6.5710 -10.3570

H 0.3920 -6.2410 -9.7890

C 1.7630 -4.5860 -9.5680

H 1.1580 -3.7180 -9.8440

H 1.6440 -4.7490 -8.4930

C 3.2360 -4.3150 -9.8670

H 3.3880 -4.3070 -10.9460

H 3.8310 -5.1320 -9.4520

C 3.7330 -3.0000 -9.2730

H 3.2210 -2.1640 -9.7590

H 3.4910 -2.9600 -8.2070

C 5.2410 -2.8560 -9.4610

H 5.4880 -3.0610 -10.5050

H 5.7470 -3.6060 -8.8480

C 5.7630 -1.4700 -9.0900

H 5.2990 -0.7220 -9.7370

H 5.4780 -1.2340 -8.0610

C 7.2820 -1.3910 -9.2290

H 7.5620 -1.6560 -10.2500

H 7.7390 -2.1240 -8.5600

C 7.8310 -0.0040 -8.9040

H 7.3990 0.7280 -9.5910

H 7.5250 0.2810 -7.8930

C 9.3540 0.0410 -9.0040

H 9.7800 -0.6970 -8.3200

H 9.6560 -0.2340 -10.0160

C 9.9180 1.4190 -8.6680

H 9.6040 1.7030 -7.6600

H 9.5040 2.1580 -9.3580

C 11.4390 1.4420 -8.7520

H 11.8700 0.7230 -8.0590

H 11.8230 2.4300 -8.5050

H 11.7690 1.1880 -9.7560

C 4.3140 -2.6050 -13.6030

H 3.9410 -2.1920 -12.6530

H 3.9140 -2.0010 -14.4230

C 5.8380 -2.5740 -13.6250

H 6.1760 -2.9300 -14.5990

H 6.2060 -3.2730 -12.8710

C 6.3870 -1.1770 -13.3550

H 5.9930 -0.4800 -14.0990

H 6.0460 -0.8380 -12.3740

C 7.9130 -1.1550 -13.3950

H 8.2510 -1.4810 -14.3800

H 8.3020 -1.8660 -12.6630

C 8.4780 0.2320 -13.0990

H 8.1010 0.9430 -13.8390

H 8.1290 0.5620 -12.1180

C 10.0040 0.2410 -13.1210

H 10.3520 -0.0830 -14.1040

H 10.3790 -0.4780 -12.3880

C 10.5760 1.6210 -12.8120

H 10.2110 2.3380 -13.5520

H 10.2190 1.9500 -11.8340

C 12.1030 1.6240 -12.8190

H 12.4600 1.3070 -13.8010

H 12.4690 0.8980 -12.0880

C 12.6760 3.0010 -12.4940

H 12.3220 3.7220 -13.2350

H 12.3060 3.3260 -11.5180

C 14.2020 2.9980 -12.4790

H 14.5710 2.6540 -13.4480

H 14.5540 2.2940 -11.7220

C 14.7770 4.3810 -12.1860

H 14.4250 5.0870 -12.9430

H 14.4090 4.7270 -11.2170

C 16.3010 4.3730 -12.1760

H 16.6730 3.6870 -11.4180

H 16.6890 5.3660 -11.9610

H 16.6860 4.0570 -13.1430

C 3.9150 -3.2930 -17.7980

H 3.3060 -2.4600 -18.1760

H 3.5640 -4.2270 -18.2530

C 5.3860 -3.0800 -18.1400

H 5.5100 -3.1540 -19.2220

H 5.9620 -3.8810 -17.6720

C 5.8990 -1.7310 -17.6420

H 5.5900 -1.6100 -16.6030

H 5.4470 -0.9290 -18.2300

C 7.4200 -1.6390 -17.7250

H 7.8600 -2.3940 -17.0680

H 7.7390 -1.8690 -18.7450

C 7.9440 -0.2600 -17.3320

H 7.5960 0.4780 -18.0580

H 7.5410 0.0190 -16.3550

C 9.4680 -0.2380 -17.2670

H 9.8050 -0.9160 -16.4780

H 9.8730 -0.6110 -18.2100

C 10.0270 1.1580 -17.0060

H 9.6280 1.5410 -16.0640

H 9.6980 1.8310 -17.8020

C 11.5520 1.1510 -16.9480

H 11.8790 0.4820 -16.1470

H 11.9440 0.7530 -17.8860

C 12.1340 2.5410 -16.7120

H 11.8000 3.2130 -17.5060

H 11.7570 2.9360 -15.7660

C 13.6600 2.5190 -16.6780

H 14.0380 2.1450 -17.6330

H 13.9930 1.8260 -15.9010

C 14.2520 3.8990 -16.4060

H 13.9200 4.5940 -17.1820

H 13.8780 4.2690 -15.4490

C 15.7750 3.8670 -16.3760

H 16.1260 3.1800 -15.6090

H 16.1770 4.8550 -16.1610

H 16.1700 3.5360 -17.3340

O 3.8810 -3.9540 -13.7080

O 1.8390 -5.0150 -12.4930

O 3.8110 -3.3710 -16.3870

O -1.9160 -5.2700 -15.9820

C -4.2170 -5.5260 -11.9730

C -2.9170 -5.5160 -11.4550

C -5.3020 -5.6120 -11.0960

C -2.7050 -5.5790 -10.1010

H -2.0920 -5.4540 -12.1470

C -5.0880 -5.6830 -9.7370

H -6.3120 -5.6270 -11.4800

C -3.7900 -5.6690 -9.2260

H -1.7060 -5.5570 -9.6990

H -5.9240 -5.7480 -9.0580

C -4.1250 -5.6320 -22.7260

C -2.8410 -5.6200 -23.2820

C -5.2440 -5.7530 -23.5550

C -2.6850 -5.7010 -24.6440

H -1.9860 -5.5300 -22.6300

C -5.0820 -5.8390 -24.9190

H -6.2270 -5.7830 -23.1090

C -3.8030 -5.8080 -25.4700

H -1.7080 -5.6730 -25.1000

H -5.9370 -5.9270 -25.5720

C -7.7590 -4.1250 -18.1460

H -8.2810 -4.8710 -17.5290

H -7.4240 -3.3080 -17.4870

C -0.7230 -5.1910 -16.7420

H -0.5360 -6.1590 -17.2320

H -0.8280 -4.4290 -17.5250

C -2.3450 -2.2750 -19.8200

C -2.8150 -2.6850 -25.4420

C -2.9400 -2.7450 -26.6440

C -3.0620 -2.8090 -28.0410

C -1.8950 -2.7740 -28.8370

C -4.3220 -2.9180 -28.6310

H -5.1780 -2.9620 -27.9780

O -3.3410 -2.2630 -19.1070

O -1.1250 -2.1780 -19.3460

H -1.1180 -2.0480 -18.3480

C -3.2600 -2.9290 -39.0670

C -3.4490 -2.9720 -33.4180

C -3.3970 -2.9190 -32.2100

C -3.3020 -2.8890 -30.8080

C -4.4610 -2.9740 -30.0080

C -2.0400 -2.8040 -30.2150

H -1.1820 -2.7670 -30.8660

O -2.1860 -2.7690 -39.6400

O -4.3990 -3.0030 -39.7150

H -4.2830 -2.8490 -40.7020

C -7.9490 -3.5540 -30.8810

C -7.7570 -4.3380 -32.0070

H -6.7980 -4.8070 -32.1490

C -8.7760 -4.4840 -32.9400

C -9.9960 -3.8250 -32.7350

C -10.1940 -3.0520 -31.5830

C -9.1680 -2.9330 -30.6510

H -9.3040 -2.3420 -29.7600

C -7.5170 -5.8970 -34.3990

H -6.6650 -5.2100 -34.3180

H -7.3630 -6.7380 -33.7070

C -7.6120 -6.4040 -35.8370

H -7.8880 -5.5650 -36.4780

H -6.6160 -6.7460 -36.1320

C -8.6110 -7.5460 -36.0340

H -8.4100 -8.3300 -35.2990

H -8.4540 -7.9710 -37.0300

C -10.0670 -7.1070 -35.9100

H -10.2570 -6.7930 -34.8850

H -10.2380 -6.2410 -36.5550

C -11.0420 -8.2210 -36.2850

H -10.8730 -9.0860 -35.6440

H -10.8690 -8.5280 -37.3210

C -12.4880 -7.7640 -36.1210

H -12.6480 -7.4940 -35.0770

H -12.6560 -6.8700 -36.7270

C -13.4980 -8.8390 -36.5140

H -13.3440 -9.7200 -35.8880

H -13.3300 -9.1360 -37.5530

C -14.9330 -8.3420 -36.3570

H -15.1110 -8.0900 -35.3080

H -15.0610 -7.4270 -36.9410

C -15.9710 -9.3680 -36.8030

H -15.8490 -10.2850 -36.2210

H -15.8020 -9.6220 -37.8530

C -17.3940 -8.8420 -36.6340

H -17.5040 -7.9160 -37.2040

H -17.5680 -8.6050 -35.5810

C -18.4480 -9.8430 -37.1010

H -18.2720 -10.0880 -38.1510

H -18.3470 -10.7670 -36.5270

C -19.8600 -9.2950 -36.9370

H -19.9860 -8.3890 -37.5250

H -20.5960 -10.0240 -37.2700

H -20.0600 -9.0590 -35.8940

C -12.1370 -4.6090 -33.4140

H -11.9730 -5.6700 -33.6510

H -12.3800 -4.5270 -32.3490

C -13.2630 -4.0390 -34.2670

H -13.4270 -3.0020 -33.9710

H -12.9290 -4.0420 -35.3050

C -14.5590 -4.8290 -34.1410

H -14.8620 -4.8750 -33.0910

H -14.3950 -5.8530 -34.4810

C -15.6800 -4.2020 -34.9670

H -15.8770 -3.1910 -34.6040

H -15.3580 -4.1200 -36.0080

C -16.9640 -5.0250 -34.8990

H -17.2690 -5.1320 -33.8550

H -16.7710 -6.0270 -35.2880

C -18.1010 -4.3870 -35.6930

H -18.3010 -3.3880 -35.3000

H -17.7970 -4.2740 -36.7360

C -19.3790 -5.2200 -35.6240

H -19.6710 -5.3440 -34.5780

H -19.1840 -6.2160 -36.0270

C -20.5280 -4.5760 -36.3940

H -20.7070 -3.5730 -36.0010

H -20.2460 -4.4710 -37.4450

C -21.8150 -5.3910 -36.2930

H -22.0990 -5.4830 -35.2410

H -21.6380 -6.4020 -36.6700

C -22.9600 -4.7520 -37.0730

H -23.1080 -3.7290 -36.7200

H -22.6910 -4.7000 -38.1300

C -24.2660 -5.5270 -36.9210

H -24.5560 -5.5460 -35.8680

H -24.1120 -6.5620 -37.2370

C -25.3900 -4.9080 -37.7430

H -25.1270 -4.8950 -38.7990

H -26.3110 -5.4750 -37.6250

H -25.5760 -3.8850 -37.4240

C -11.6840 -1.5740 -30.4200

H -11.7780 -2.1320 -29.4770

H -10.8740 -0.8420 -30.3200

C -12.9910 -0.8610 -30.7560

H -13.1970 -0.1130 -29.9880

H -12.8530 -0.3420 -31.7060

C -14.1600 -1.8360 -30.8750

H -13.8550 -2.6700 -31.5080

H -14.4020 -2.2340 -29.8870

C -15.3950 -1.1750 -31.4790

H -15.1580 -0.8340 -32.4880

H -15.6670 -0.2970 -30.8880

C -16.5860 -2.1280 -31.5440

H -16.8730 -2.4170 -30.5310

H -16.2930 -3.0350 -32.0770

C -17.7850 -1.4970 -32.2460

H -17.5120 -1.2510 -33.2750

H -18.0460 -0.5610 -31.7450

C -19.0000 -2.4210 -32.2520

H -18.7360 -3.3610 -32.7420

H -19.2810 -2.6540 -31.2230

C -20.1930 -1.7960 -32.9700

H -19.9270 -1.5990 -34.0120

H -20.4320 -0.8360 -32.5060

C -21.4250 -2.6960 -32.9240

H -21.6990 -2.8790 -31.8820

H -21.1840 -3.6610 -33.3750

C -22.6150 -2.0820 -33.6570

H -22.8490 -1.1100 -33.2160

H -22.3450 -1.9110 -34.7020

C -23.8520 -2.9730 -33.5960

H -24.1290 -3.1360 -32.5520

H -23.6140 -3.9470 -34.0300

C -25.0310 -2.3630 -34.3440

H -24.7760 -2.1990 -35.3890

H -25.8960 -3.0210 -34.3010

H -25.3060 -1.4050 -33.9070

O -10.9530 -3.8860 -33.7170

O -8.7090 -5.2140 -34.0770

O -11.4120 -2.4610 -31.4840

O -5.6370 -3.1160 -30.6660

C 1.6320 -2.6870 -27.9200

C 1.5440 -1.9480 -26.7510

H 0.6490 -1.3760 -26.5710

C 2.5920 -1.9690 -25.8360

C 3.7380 -2.7240 -26.1130

C 3.8350 -3.4430 -27.3140

C 2.7690 -3.4250 -28.2080

H 2.8160 -3.9940 -29.1230

C 1.5830 -0.3760 -24.3870

H 0.6080 -0.8800 -24.4160

H 1.5880 0.4150 -25.1520

C 1.7950 0.2330 -23.0030

H 1.7400 -0.5570 -22.2530

H 0.9670 0.9210 -22.8270

C 3.1240 0.9780 -22.8580

H 3.3860 1.4350 -23.8150

H 3.0140 1.7790 -22.1270

C 4.2620 0.0680 -22.4010

H 4.3780 -0.7590 -23.1020

H 4.0070 -0.3510 -21.4240

C 5.5670 0.8480 -22.2780

H 5.8300 1.2670 -23.2530

H 5.4110 1.6800 -21.5890

C 6.7220 -0.0170 -21.7790

H 6.8410 -0.8770 -22.4410

H 6.4850 -0.3960 -20.7820

C 8.0310 0.7670 -21.7250

H 8.2330 1.1980 -22.7090

H 7.9240 1.5970 -21.0220

C 9.2160 -0.1000 -21.3070

H 9.3330 -0.9180 -22.0190

H 9.0110 -0.5400 -20.3300

C 10.5130 0.7020 -21.2380

H 10.7010 1.1680 -22.2090

H 10.4010 1.5060 -20.5060

C 11.7140 -0.1600 -20.8560

H 11.5330 -0.6220 -19.8820

H 11.8280 -0.9660 -21.5830

C 13.0050 0.6530 -20.7940

H 12.8750 1.4860 -20.0990

H 13.2110 1.0790 -21.7790

C 14.1910 -0.1960 -20.3510

H 14.0210 -0.5990 -19.3540

H 15.1030 0.3980 -20.3290

H 14.3400 -1.0280 -21.0350

C 5.9130 -2.0320 -25.5100

H 5.8090 -1.0060 -25.1250

H 6.0370 -1.9770 -26.5980

C 7.1240 -2.7110 -24.8810

H 7.2390 -3.6990 -25.3300

H 6.9280 -2.8520 -23.8170

C 8.3910 -1.8850 -25.0800

H 8.5200 -1.6720 -26.1450

H 8.2830 -0.9270 -24.5660

C 9.6340 -2.6010 -24.5570

H 9.7740 -3.5340 -25.1090

H 9.4880 -2.8620 -23.5060

C 10.8840 -1.7350 -24.6970

H 10.9900 -1.4230 -25.7390

H 10.7650 -0.8310 -24.0970

C 12.1500 -2.4680 -24.2600

H 12.2970 -3.3450 -24.8950

H 12.0280 -2.8240 -23.2340

C 13.3810 -1.5700 -24.3420

H 13.4770 -1.1820 -25.3600

H 13.2480 -0.7130 -23.6780

C 14.6620 -2.3080 -23.9620

H 14.8150 -3.1420 -24.6510

H 14.5540 -2.7280 -22.9590

C 15.8820 -1.3920 -23.9980

H 15.9790 -0.9630 -24.9990

H 15.7340 -0.5640 -23.3000

C 17.1680 -2.1310 -23.6400

H 17.3090 -2.9640 -24.3320

H 17.0750 -2.5500 -22.6340

C 18.3910 -1.2200 -23.6940

H 18.4930 -0.8120 -24.7020

H 18.2450 -0.3800 -23.0100

C 19.6690 -1.9600 -23.3190

H 19.5970 -2.3590 -22.3090

H 20.5270 -1.2930 -23.3640

H 19.8440 -2.7890 -24.0010

C 5.2010 -4.8190 -28.7120

H 5.1030 -4.1390 -29.5700

H 4.4640 -5.6250 -28.8080

C 6.6010 -5.4180 -28.6680

H 6.7340 -6.0350 -29.5570

H 6.6610 -6.0670 -27.7940

C 7.6820 -4.3420 -28.5900

H 7.4730 -3.7000 -27.7340

H 7.6460 -3.7230 -29.4890

C 9.0770 -4.9400 -28.4390

H 9.1120 -5.5470 -27.5320

H 9.2840 -5.6020 -29.2850

C 10.1530 -3.8590 -28.3650

H 10.1410 -3.2730 -29.2850

H 9.9250 -3.1810 -27.5400

C 11.5480 -4.4440 -28.1590

H 11.5720 -5.0000 -27.2190

H 11.7670 -5.1530 -28.9630

C 12.6240 -3.3610 -28.1350

H 12.3970 -2.6450 -27.3430

H 12.6090 -2.8190 -29.0830

C 14.0190 -3.9370 -27.9070

H 14.0450 -4.4480 -26.9410

H 14.2350 -4.6830 -28.6760

C 15.0970 -2.8560 -27.9350

H 15.0830 -2.3580 -28.9080

H 14.8720 -2.1040 -27.1760

C 16.4890 -3.4260 -27.6800

H 16.7150 -4.1820 -28.4360

H 16.5030 -3.9200 -26.7060

C 17.5680 -2.3460 -27.7100

H 17.5660 -1.8610 -28.6890

H 17.3340 -1.5840 -26.9640

C 18.9520 -2.9170 -27.4300

H 18.9760 -3.3940 -26.4520

H 19.7040 -2.1310 -27.4450

H 19.2180 -3.6610 -28.1780

O 4.7480 -2.7730 -25.1800

O 2.6130 -1.3000 -24.6560

O 5.0000 -4.1060 -27.5080

O -0.7280 -2.7090 -28.1650

C -2.6840 -2.6120 -24.0450

C -1.4180 -2.6200 -23.4500

C -3.8240 -2.5350 -23.2380

C -1.2950 -2.5100 -22.0820

H -0.5460 -2.7250 -24.0770

C -3.6990 -2.4430 -21.8720

H -4.7970 -2.5560 -23.7010

C -2.4350 -2.4120 -21.2830

H -0.3210 -2.5020 -21.6190

H -4.5650 -2.3830 -21.2320

C -3.4350 -3.0230 -34.8240

C -2.2110 -2.9060 -35.4910

C -4.6090 -3.1890 -35.5660

C -2.1680 -2.9080 -36.8640

H -1.3080 -2.7980 -34.9090

C -4.5630 -3.1890 -36.9430

H -5.5540 -3.2780 -35.0520

C -3.3460 -3.0260 -37.6030

H -1.2340 -2.8050 -37.3920

H -5.4690 -3.2910 -37.5190

C -6.8080 -3.3650 -29.9150

H -6.6670 -4.2700 -29.3020

H -7.0210 -2.5210 -29.2460

C 0.4830 -2.6690 -28.8930

H 0.5510 -3.5300 -29.5710

H 0.5150 -1.7420 -29.4870

C -2.8770 -2.6480 -42.8740

C -2.2400 -2.6090 -48.5030

C -2.0200 -2.6190 -49.6930

C -1.9260 -2.5890 -51.0940

C -0.7070 -2.8050 -51.7670

C -3.0990 -2.3480 -51.8140

H -3.9970 -2.2040 -51.2360

O -3.9580 -2.5090 -42.3150

O -1.7530 -2.8080 -42.2190

H -1.8990 -2.7830 -41.2200

C -3.2870 -1.7670 -61.9010

C -1.7630 -2.1570 -56.4670

C -1.8270 -2.2810 -55.2640

C -1.8680 -2.4180 -53.8670

C -3.1060 -2.2900 -53.1980

C -0.7020 -2.6920 -53.1490

H 0.2050 -2.8150 -53.7180

O -2.5480 -1.8140 -62.8780

O -4.5960 -1.6910 -61.9980

H -4.9310 -1.6690 -62.9480

C -6.6810 -2.2220 -54.1010

C -6.6990 -2.0070 -55.4680

H -5.7730 -1.8120 -55.9820

C -7.9120 -2.0550 -56.1430

C -9.1210 -2.2940 -55.4590

C -9.0940 -2.4740 -54.0700

C -7.8660 -2.4650 -53.4210

H -7.8170 -2.6350 -52.3580

C -6.9910 -1.8750 -58.3540

H -6.3910 -0.9590 -58.2480

H -6.3510 -2.7360 -58.1200

C -7.5090 -1.9870 -59.7910

H -7.9370 -1.0300 -60.0860

H -6.6530 -2.1880 -60.4370

C -8.5710 -3.0780 -59.9700

H -8.3800 -3.8930 -59.2680

H -8.4980 -3.4920 -60.9800

C -9.9830 -2.5370 -59.7540

H -10.0450 -2.0950 -58.7610

H -10.1770 -1.7470 -60.4850

C -11.0520 -3.6170 -59.8900

H -10.9140 -4.3670 -59.1110

H -10.9500 -4.1190 -60.8580

C -12.4540 -3.0290 -59.7650

H -12.5120 -2.4540 -58.8400

H -12.6260 -2.3360 -60.5930

C -13.5410 -4.1020 -59.7650

H -13.3760 -4.7880 -58.9300

H -13.4730 -4.6900 -60.6850

C -14.9330 -3.4830 -59.6640

H -15.0010 -2.9000 -58.7440

H -15.0780 -2.7920 -60.4970

C -16.0540 -4.5200 -59.6840

H -15.9620 -5.1760 -58.8140

H -15.9540 -5.1460 -60.5740

C -17.4280 -3.8550 -59.6850

H -17.5180 -3.2230 -60.5710

H -17.5160 -3.2050 -58.8110

C -18.5700 -4.8680 -59.6770

H -18.4540 -5.5490 -60.5230

H -18.5170 -5.4680 -58.7650

C -19.9300 -4.1860 -59.7600

H -20.0170 -3.6240 -60.6870

H -20.7330 -4.9200 -59.7290

H -20.0620 -3.4940 -58.9300

C -11.3770 -3.0180 -55.9660

H -11.5820 -3.6120 -56.8650

H -11.2040 -3.7100 -55.1400

C -12.5710 -2.1070 -55.6820

H -12.4290 -1.6090 -54.7230

H -12.6100 -1.3400 -56.4590

C -13.8500 -2.9350 -55.6730

H -13.7950 -3.6490 -54.8480

H -13.8980 -3.5110 -56.6000

C -15.1350 -2.1230 -55.5360

H -15.1520 -1.6100 -54.5720

H -15.1710 -1.3570 -56.3150

C -16.3510 -3.0380 -55.6600

H -16.3040 -3.7990 -54.8780

H -16.3040 -3.5570 -56.6200

C -17.6880 -2.3090 -55.5600

H -17.7680 -1.8170 -54.5880

H -17.7400 -1.5320 -56.3270

C -18.8500 -3.2830 -55.7390

H -18.7660 -4.0790 -54.9960

H -18.7780 -3.7480 -56.7240

C -20.2150 -2.6140 -55.6010

H -20.3070 -2.1790 -54.6020

H -20.2950 -1.7970 -56.3220

C -21.3530 -3.6050 -55.8290

H -21.2460 -4.4410 -55.1350

H -21.2820 -4.0100 -56.8410

C -22.7270 -2.9670 -55.6390

H -22.8160 -2.5980 -54.6150

H -22.8230 -2.1080 -56.3070

C -23.8590 -3.9530 -55.9210

H -23.7470 -4.8250 -55.2730

H -23.7850 -4.2980 -56.9550

C -25.2280 -3.3260 -55.6930

H -25.3680 -2.4660 -56.3440

H -26.0180 -4.0450 -55.8980

H -25.3300 -2.9920 -54.6620

C -10.2940 -2.5830 -51.9990

H -9.9100 -3.5280 -51.5880

H -9.6620 -1.7600 -51.6430

C -11.7300 -2.3460 -51.5410

H -11.7210 -2.1290 -50.4710

H -12.1060 -1.4650 -52.0630

C -12.6520 -3.5300 -51.8210

H -12.5090 -3.8560 -52.8530

H -12.3850 -4.3630 -51.1660

C -14.1110 -3.1460 -51.6000

H -14.3580 -2.3130 -52.2630

H -14.2400 -2.7980 -50.5720

C -15.0820 -4.2920 -51.8660

H -14.9050 -5.0960 -51.1470

H -14.9010 -4.7020 -52.8620

C -16.5300 -3.8250 -51.7590

H -16.6880 -2.9900 -52.4450

H -16.7140 -3.4580 -50.7470

C -17.5360 -4.9260 -52.0810

H -17.3720 -5.2830 -53.1010

H -17.3780 -5.7750 -51.4110

C -18.9710 -4.4260 -51.9360

H -19.1200 -3.5670 -52.5930

H -19.1270 -4.0850 -50.9110

C -20.0060 -5.4970 -52.2690

H -19.8380 -6.3750 -51.6400

H -19.8820 -5.8120 -53.3090

C -21.4300 -4.9910 -52.0570

H -21.5570 -4.7050 -51.0110

H -21.5910 -4.0990 -52.6650

C -22.4790 -6.0400 -52.4180

H -22.3050 -6.9430 -51.8270

H -22.3720 -6.3120 -53.4710

C -23.8940 -5.5340 -52.1640

H -24.0860 -4.6340 -52.7440

H -24.6280 -6.2860 -52.4440

H -24.0300 -5.2970 -51.1110

O -10.2190 -2.2600 -56.2600

O -8.0900 -1.8390 -57.4740

O -10.2850 -2.6270 -53.4120

O -4.2270 -2.1240 -53.9530

C 2.6920 -3.7370 -50.7070

C 2.5690 -3.5760 -49.3380

H 1.6290 -3.2330 -48.9400

C 3.6570 -3.8520 -48.5130

C 4.8710 -4.2630 -49.0740

C 4.9870 -4.4190 -50.4630

C 3.8880 -4.1590 -51.2690

H 3.9450 -4.2990 -52.3350

C 2.5330 -3.2560 -46.4890

H 1.6460 -3.8380 -46.7740

H 2.3620 -2.2040 -46.7680

C 2.7380 -3.3770 -44.9790

H 2.8730 -4.4310 -44.7320

H 1.8210 -3.0260 -44.5010

C 3.9290 -2.5740 -44.4520

H 4.0010 -1.6340 -45.0040

H 3.7530 -2.3170 -43.4030

C 5.2620 -3.3130 -44.5670

H 5.4030 -3.6430 -45.5960

H 5.2380 -4.2030 -43.9330

C 6.4180 -2.4120 -44.1410

H 6.4100 -1.5060 -44.7510

H 6.2690 -2.0970 -43.1050

C 7.7850 -3.0770 -44.2730

H 7.8920 -3.4840 -45.2800

H 7.8600 -3.9110 -43.5710

C 8.9090 -2.0770 -44.0060

H 8.8250 -1.2530 -44.7180

H 8.7810 -1.6450 -43.0110

C 10.2960 -2.7040 -44.1190

H 10.4010 -3.1770 -45.0980

H 10.4050 -3.4860 -43.3640

C 11.4030 -1.6680 -43.9390

H 11.3080 -0.9040 -44.7150

H 11.2730 -1.1680 -42.9760

C 12.7970 -2.2870 -44.0050

H 12.8990 -3.0330 -43.2150

H 12.9200 -2.8010 -44.9610

C 13.9000 -1.2420 -43.8510

H 13.7640 -0.7100 -42.9060

H 13.8140 -0.5050 -44.6540

C 15.2880 -1.8690 -43.8820

H 15.4040 -2.5780 -43.0660

H 16.0580 -1.1060 -43.7830

H 15.4470 -2.3980 -44.8190

C 6.9590 -3.5100 -48.2990

H 6.7970 -2.7990 -47.4750

H 6.8980 -2.9540 -49.2410

C 8.3340 -4.1540 -48.1730

H 8.4780 -4.8320 -49.0160

H 8.3630 -4.7510 -47.2590

C 9.4310 -3.0930 -48.1470

H 9.3420 -2.4630 -49.0360

H 9.2870 -2.4490 -47.2760

C 10.8320 -3.6980 -48.1010

H 10.9950 -4.3070 -48.9920

H 10.9150 -4.3570 -47.2330

C 11.9050 -2.6150 -48.0190

H 11.7930 -1.9320 -48.8650

H 11.7560 -2.0310 -47.1080

C 13.3180 -3.1910 -48.0240

H 13.4820 -3.7420 -48.9520

H 13.4230 -3.9000 -47.1990

C 14.3760 -2.0990 -47.8870

H 14.2700 -1.3880 -48.7100

H 14.2080 -1.5500 -46.9580

C 15.7940 -2.6630 -47.8900

H 15.9750 -3.1840 -48.8330

H 15.8930 -3.3970 -47.0860

C 16.8450 -1.5700 -47.7070

H 16.7630 -0.8510 -48.5260

H 16.6490 -1.0330 -46.7760

C 18.2630 -2.1340 -47.6720

H 18.4590 -2.6780 -48.5990

H 18.3470 -2.8470 -46.8480

C 19.3130 -1.0400 -47.4990

H 19.2390 -0.3340 -48.3280

H 19.1110 -0.4900 -46.5770

C 20.7250 -1.6110 -47.4470

H 20.8280 -2.2990 -46.6100

H 21.4570 -0.8150 -47.3290

H 20.9540 -2.1520 -48.3630

C 6.4130 -4.9130 -52.3100

H 6.1810 -3.9600 -52.8050

H 5.7590 -5.6920 -52.7180

C 7.8820 -5.2610 -52.5260

H 8.0260 -5.5590 -53.5660

H 8.1340 -6.1110 -51.8890

C 8.7890 -4.0800 -52.1890

H 8.5910 -3.7680 -51.1640

H 8.5450 -3.2430 -52.8470

C 10.2690 -4.4180 -52.3400

H 10.5300 -5.2260 -51.6520

H 10.4580 -4.7800 -53.3550

C 11.1540 -3.2070 -52.0620

H 10.9040 -2.4090 -52.7640

H 10.9500 -2.8370 -51.0560

C 12.6400 -3.5340 -52.1840

H 12.8970 -4.3190 -51.4690

H 12.8450 -3.9240 -53.1850

C 13.5150 -2.3100 -51.9280

H 13.2890 -1.9090 -50.9380

H 13.2780 -1.5360 -52.6600

C 15.0030 -2.6390 -52.0080

H 15.2460 -3.3930 -51.2550

H 15.2300 -3.0680 -52.9870

C 15.8740 -1.4050 -51.7850

H 15.6630 -0.6690 -52.5640

H 15.6150 -0.9520 -50.8250

C 17.3620 -1.7420 -51.7940

H 17.6240 -2.2030 -52.7500

H 17.5700 -2.4720 -51.0080

C 18.2350 -0.5090 -51.5740

H 18.0420 0.2160 -52.3690

H 17.9610 -0.0390 -50.6270

C 19.7170 -0.8580 -51.5550

H 19.9300 -1.5670 -50.7570

H 20.3210 0.0320 -51.3910

H 20.0160 -1.3070 -52.4990

O 5.9480 -4.5080 -48.2500

O 3.6680 -3.7490 -47.1560

O 6.2080 -4.7920 -50.9150

O 0.3700 -3.1000 -50.9990

C -2.4160 -2.6200 -47.1080

C -1.3090 -2.8100 -46.2740

C -3.6810 -2.4440 -46.5370

C -1.4590 -2.8200 -44.9080

H -0.3370 -2.9410 -46.7210

C -3.8270 -2.4540 -45.1680

H -4.5340 -2.2920 -47.1810

C -2.7200 -2.6410 -44.3400

H -0.6080 -2.9590 -44.2610

H -4.7940 -2.3100 -44.7120

C -2.0250 -2.0130 -57.8420

C -1.0760 -2.0990 -58.8640

C -3.3660 -1.7850 -58.1740

C -1.4660 -2.0060 -60.1860

H -0.0330 -2.2470 -58.6220

C -3.7490 -1.6790 -59.4840

H -4.0860 -1.7060 -57.3750

C -2.8090 -1.8080 -60.5080

H -0.7370 -2.1000 -60.9770

H -4.7770 -1.5010 -59.7400

C -5.4490 -2.1690 -53.2390

H -5.4560 -3.0640 -52.6020

H -5.5240 -1.2820 -52.5920

C 1.5690 -3.4320 -51.6650

H 1.4030 -4.3110 -52.3070

H 1.8820 -2.5930 -52.3060

C -2.6540 -2.9020 -2.4260

C -2.4770 -2.9310 -3.6140

C -2.3140 -2.9090 -5.0180

C -1.0530 -2.9190 -5.6440

C -3.4840 -2.8470 -5.7730

H -4.4170 -2.8670 -5.2340

C -2.2050 -2.1520 -16.0050

C -1.9310 -2.4750 -10.3690

C -2.0840 -2.5910 -9.1750

C -2.1790 -2.7170 -7.7800

C -3.4430 -2.7430 -7.1560

C -1.0110 -2.8260 -7.0250

H -0.0740 -2.8300 -7.5580

O -1.2190 -1.9650 -16.7100

O -3.4130 -2.3030 -16.4920

H -3.4230 -2.2670 -17.5030

C -6.9510 -2.5430 -8.2720

C -6.8310 -2.5340 -9.6510

H -5.8590 -2.6810 -10.0900

C -7.9650 -2.3290 -10.4350

C -9.2160 -2.1560 -9.8270

C -9.3180 -2.1160 -8.4310

C -8.1810 -2.3210 -7.6650

H -8.2390 -2.3080 -6.5890

C -6.8570 -2.5570 -12.5510

H -6.1100 -1.7600 -12.4420

H -6.4160 -3.5020 -12.2120

C -7.2630 -2.6780 -14.0180

H -7.5930 -1.7050 -14.3830

H -6.3650 -2.9600 -14.5720

C -8.3690 -3.7130 -14.2460

H -8.2810 -4.5140 -13.5080

H -8.2410 -4.1590 -15.2350

C -9.7750 -3.1240 -14.1860

H -9.9320 -2.6490 -13.2190

H -9.8750 -2.3520 -14.9530

C -10.8290 -4.2030 -14.4180

H -10.7490 -4.9610 -13.6350

H -10.6300 -4.7060 -15.3700

C -12.2450 -3.6330 -14.4430

H -12.4360 -3.0980 -13.5120

H -12.3270 -2.9050 -15.2540

C -13.2890 -4.7310 -14.6310

H -13.2060 -5.4440 -13.8080

H -13.0760 -5.2770 -15.5540

C -14.7150 -4.1920 -14.6840

H -14.9210 -3.6170 -13.7790

H -14.8100 -3.5110 -15.5320

C -15.7470 -5.3090 -14.8250

H -15.7290 -5.9390 -13.9320

H -15.4810 -5.9420 -15.6750

C -17.1560 -4.7600 -15.0330

H -17.1720 -4.1530 -15.9410

H -17.4180 -4.1080 -14.1970

C -18.1990 -5.8680 -15.1560

H -17.9120 -6.5450 -15.9640

H -18.2190 -6.4520 -14.2320

C -19.5880 -5.3080 -15.4360

H -19.5960 -4.7640 -16.3770

H -20.3210 -6.1090 -15.4980

H -19.8900 -4.6260 -14.6440

C -11.3450 -2.9250 -10.5310

H -11.2140 -3.6570 -11.3400

H -11.2820 -3.4590 -9.5780

C -12.7000 -2.2390 -10.6580

H -12.8340 -1.5640 -9.8120

H -12.7010 -1.6330 -11.5670

C -13.8190 -3.2760 -10.6930

H -13.7820 -3.8760 -9.7790

H -13.6500 -3.9560 -11.5310

C -15.2070 -2.6580 -10.8330

H -15.4070 -2.0040 -9.9830

H -15.2390 -2.0430 -11.7360

C -16.2840 -3.7370 -10.9160

H -16.2760 -4.3250 -9.9950

H -16.0470 -4.4180 -11.7380

C -17.6790 -3.1600 -11.1350

H -17.9310 -2.4940 -10.3060

H -17.6830 -2.5620 -12.0500

C -18.7340 -4.2580 -11.2430

H -18.7380 -4.8460 -10.3220

H -18.4690 -4.9330 -12.0600

C -20.1310 -3.6960 -11.4890

H -20.4010 -3.0270 -10.6690

H -20.1260 -3.1040 -12.4080

C -21.1790 -4.8000 -11.6050

H -21.1850 -5.3880 -10.6840

H -20.9080 -5.4740 -12.4210

C -22.5770 -4.2410 -11.8550

H -22.8420 -3.5590 -11.0440

H -22.5740 -3.6640 -12.7830

C -23.6300 -5.3420 -11.9500

H -23.6320 -5.9210 -11.0240

H -23.3670 -6.0220 -12.7640

C -25.0230 -4.7760 -12.1920

H -25.0480 -4.2070 -13.1190

H -25.7560 -5.5770 -12.2620

H -25.3130 -4.1160 -11.3780

C -10.7200 -1.6180 -6.5540

H -10.5370 -2.5340 -5.9750

H -10.0100 -0.8470 -6.2280

C -12.1530 -1.1340 -6.3470

H -12.2560 -0.7610 -5.3270

H -12.3300 -0.3050 -7.0350

C -13.1740 -2.2380 -6.6110

H -12.9520 -2.6970 -7.5750

H -13.0830 -3.0090 -5.8420

C -14.6020 -1.6990 -6.6270

H -14.6950 -0.9620 -7.4290

H -14.8080 -1.1860 -5.6840

C -15.6340 -2.8040 -6.8380

H -15.5930 -3.5040 -6.0000

H -15.3860 -3.3590 -7.7450

C -17.0490 -2.2450 -6.9620

H -17.0930 -1.5660 -7.8170

H -17.2880 -1.6630 -6.0690

C -18.0930 -3.3450 -7.1410

H -17.8370 -3.9460 -8.0160

H -18.0750 -4.0060 -6.2710

C -19.4990 -2.7770 -7.3150

H -19.5170 -2.1240 -8.1910

H -19.7530 -2.1660 -6.4450

C -20.5470 -3.8740 -7.4840

H -20.5420 -4.5190 -6.6020

H -20.2850 -4.4920 -8.3460

C -21.9490 -3.3040 -7.6830

H -22.2150 -2.6870 -6.8210

H -21.9530 -2.6560 -8.5640

C -22.9980 -4.3990 -7.8620

H -23.0010 -5.0430 -6.9790

H -22.7280 -5.0170 -8.7210

C -24.3920 -3.8220 -8.0730

H -24.4100 -3.1800 -8.9500

H -25.1190 -4.6190 -8.2160

H -24.6960 -3.2300 -7.2120

O -10.3080 -1.9600 -10.6340

O -8.0030 -2.2540 -11.7880

O -10.5610 -1.8690 -7.9350

O -4.5360 -2.6480 -7.9650

C 2.3630 -2.8450 -4.3480

C 2.0270 -2.5900 -3.0320

H 0.9870 -2.5040 -2.7680

C 3.0300 -2.4550 -2.0800

C 4.3750 -2.5580 -2.4570

C 4.7060 -2.8170 -3.7950

C 3.6900 -2.9520 -4.7310

H 3.9190 -3.1680 -5.7610

C 1.5670 -1.7840 -0.2510

H 0.7900 -2.5420 -0.4250

H 1.2630 -0.8570 -0.7570

C 1.7020 -1.5420 1.2520

H 1.9690 -2.4830 1.7360

H 0.7190 -1.2370 1.6190

C 2.7430 -0.4780 1.6080

H 2.7160 0.3170 0.8590

H 2.4830 -0.0300 2.5710

C 4.1590 -1.0480 1.6940

H 4.3910 -1.5750 0.7680

H 4.2010 -1.7730 2.5100

C 5.2000 0.0410 1.9380

H 5.1840 0.7490 1.1040

H 4.9470 0.5980 2.8440

C 6.6060 -0.5400 2.0770

H 6.8220 -1.1570 1.2030

H 6.6410 -1.1910 2.9540

C 7.6710 0.5450 2.2090

H 7.6240 1.2050 1.3370

H 7.4610 1.1550 3.0910

C 9.0780 -0.0390 2.3220

H 9.2820 -0.6540 1.4420

H 9.1290 -0.6890 3.1980

C 10.1430 1.0480 2.4360

H 10.0750 1.7140 1.5710

H 9.9500 1.6520 3.3270

C 11.5530 0.4680 2.5160

H 11.6210 -0.1990 3.3780

H 11.7480 -0.1280 1.6220

C 12.6180 1.5560 2.6370

H 12.4340 2.1420 3.5400

H 12.5370 2.2350 1.7840

C 14.0240 0.9710 2.6900

H 14.1320 0.3140 3.5490

H 14.7660 1.7630 2.7690

H 14.2300 0.3970 1.7900

C 6.2330 -1.3360 -1.6300

H 5.9600 -0.5650 -0.8960

H 6.1480 -0.8980 -2.6290

C 7.6660 -1.8000 -1.3840

H 7.9440 -2.4990 -2.1730

H 7.6980 -2.3370 -0.4350

C 8.6310 -0.6180 -1.3500

H 8.5450 -0.0540 -2.2830

H 8.3470 0.0530 -0.5370

C 10.0810 -1.0550 -1.1590

H 10.3880 -1.6730 -2.0060

H 10.1580 -1.6700 -0.2590

C 11.0190 0.1430 -1.0340

H 10.9100 0.7800 -1.9150

H 10.7270 0.7360 -0.1650

C 12.4800 -0.2730 -0.8920

H 12.7880 -0.8260 -1.7820

H 12.5850 -0.9440 -0.0360

C 13.3970 0.9330 -0.7020

H 13.2870 1.6070 -1.5550

H 13.0890 1.4800 0.1910

C 14.8630 0.5330 -0.5650

H 15.1870 0.0230 -1.4760

H 14.9710 -0.1740 0.2610

C 15.7620 1.7410 -0.3150

H 15.6550 2.4460 -1.1430

H 15.4350 2.2500 0.5950

C 17.2300 1.3500 -0.1730

H 17.5590 0.8440 -1.0840

H 17.3380 0.6430 0.6530

C 18.1250 2.5610 0.0800

H 18.0120 3.2710 -0.7420

H 17.8010 3.0620 0.9950

C 19.5910 2.1670 0.2110

H 19.7280 1.4780 1.0420

H 20.2100 3.0440 0.3860

H 19.9390 1.6780 -0.6970

C 6.4090 -3.2500 -5.4040

H 6.0470 -2.5040 -6.1260

H 5.9790 -4.2260 -5.6540

C 7.9310 -3.3110 -5.4540

H 8.2310 -3.7570 -6.4050

H 8.2770 -3.9620 -4.6490

C 8.5590 -1.9280 -5.3030

H 8.2080 -1.4850 -4.3710

H 8.2250 -1.2890 -6.1230

C 10.0840 -1.9840 -5.2960

H 10.4160 -2.6420 -4.4890

H 10.4370 -2.4170 -6.2360

C 10.7050 -0.6030 -5.1060

H 10.3870 0.0530 -5.9180

H 10.3390 -0.1720 -4.1720

C 12.2290 -0.6610 -5.0660

H 12.5410 -1.3380 -4.2670

H 12.6030 -1.0750 -6.0070

C 12.8540 0.7120 -4.8320

H 12.4560 1.1320 -3.9060

H 12.5750 1.3840 -5.6470

C 14.3750 0.6360 -4.7340

H 14.6470 -0.0440 -3.9230

H 14.7780 0.2200 -5.6600

C 15.0080 2.0010 -4.4760

H 14.7620 2.6750 -5.3000

H 14.5840 2.4280 -3.5640

C 16.5240 1.9090 -4.3290

H 16.9510 1.4850 -5.2420

H 16.7640 1.2310 -3.5070

C 17.1640 3.2680 -4.0590

H 16.9240 3.9500 -4.8780

H 16.7400 3.6910 -3.1450

C 18.6770 3.1630 -3.9130

H 18.9350 2.4940 -3.0960

H 19.1130 4.1380 -3.7090

H 19.1220 2.7730 -4.8270

O 5.3340 -2.4250 -1.4800

O 2.8090 -2.2210 -0.7570

O 6.0300 -2.8800 -4.0920

O 0.0220 -3.0110 -4.8300

C -1.9470 -2.3700 -11.7700

C -0.7840 -2.2450 -12.5370

C -3.1940 -2.4020 -12.4050

C -0.8750 -2.1690 -13.9100

H 0.1800 -2.2110 -12.0510

C -3.2820 -2.3220 -13.7720

H -4.0770 -2.4950 -11.7940

C -2.1210 -2.2130 -14.5370

H 0.0130 -2.0890 -14.5200

H -4.2400 -2.3400 -14.2640

C -5.7960 -2.7940 -7.3400

H -5.8780 -3.8150 -6.9350

H -5.8850 -2.0840 -6.5080

C 1.3050 -2.9710 -5.4130

H 1.4800 -3.8870 -5.9980

H 1.3820 -2.1050 -6.0890

C -2.6590 -8.4970 -21.2980

C -2.7250 -8.9710 -26.9340

C -2.8680 -9.0070 -28.1340

C -3.1360 -9.0490 -29.5130

C -2.1110 -9.2580 -30.4570

C -4.4530 -8.8560 -29.9340

H -5.1810 -8.6470 -29.1670

O -3.6880 -8.6840 -20.6610

O -1.5260 -8.1450 -20.7380

H -1.6030 -8.1200 -19.7340

C -4.1930 -9.0890 -40.4460

C -4.1510 -9.3470 -34.7910

C -4.0500 -9.2860 -33.5870

C -3.7910 -9.2000 -32.2080

C -4.8010 -8.8930 -31.2740

C -2.4690 -9.3700 -31.7890

H -1.7350 -9.5800 -32.5510

O -3.1530 -9.1990 -41.0820

O -5.3570 -8.8530 -41.0010

H -5.2810 -8.7290 -42.0000

C -8.3930 -8.1750 -31.4730

C -8.7460 -9.1940 -32.3430

H -7.9810 -9.8580 -32.7110

C -10.0670 -9.3350 -32.7280

C -11.0770 -8.5010 -32.1980

C -10.7050 -7.4660 -31.3300

C -9.3590 -7.2980 -31.0130

H -9.0560 -6.4780 -30.3840

C -9.6960 -11.3800 -33.9080

H -9.2380 -11.7900 -32.9990

H -8.8900 -11.0750 -34.5920

C -10.5770 -12.4320 -34.5800

H -9.9640 -13.3080 -34.8000

H -10.9410 -12.0210 -35.5240

C -11.7790 -12.8250 -33.7210

H -12.3560 -11.9260 -33.5000

H -12.4110 -13.5000 -34.3030

C -11.3930 -13.5130 -32.4130

H -10.8980 -12.7980 -31.7530

H -10.6860 -14.3210 -32.6220

C -12.6100 -14.0880 -31.6910

H -13.3270 -13.2890 -31.4950

H -13.0990 -14.8190 -32.3400

C -12.2260 -14.7650 -30.3770

H -11.8230 -14.0190 -29.6880

H -11.4360 -15.4950 -30.5680

C -13.4100 -15.4740 -29.7240

H -14.2050 -14.7510 -29.5270

H -13.8060 -16.2200 -30.4170

C -13.0170 -16.1650 -28.4200

H -12.6860 -15.4160 -27.6970

H -12.1730 -16.8330 -28.6080

C -14.1700 -16.9710 -27.8260

H -15.0130 -16.3060 -27.6280

H -14.5000 -17.7140 -28.5560

C -13.7740 -17.6820 -26.5350

H -12.9160 -18.3310 -26.7280

H -13.4630 -16.9400 -25.7940

C -14.9180 -18.5170 -25.9650

H -15.2390 -19.2430 -26.7150

H -15.7670 -17.8640 -25.7510

C -14.5140 -19.2520 -24.6930

H -13.6810 -19.9250 -24.8890

H -15.3460 -19.8380 -24.3090

H -14.2080 -18.5480 -23.9230

C -13.5040 -8.1470 -32.3650

H -14.1750 -8.4750 -33.1670

H -13.3410 -7.0700 -32.4510

C -14.1550 -8.4590 -31.0150

H -15.1510 -8.0100 -31.0220

H -13.5800 -7.9810 -30.2250

C -14.2650 -9.9540 -30.7290

H -14.8230 -10.4440 -31.5300

H -13.2640 -10.3900 -30.7110

C -14.9580 -10.1930 -29.3900

H -15.9860 -9.8250 -29.4490

H -14.4480 -9.6110 -28.6190

C -14.9760 -11.6590 -28.9630

H -15.5060 -12.2530 -29.7110

H -13.9520 -12.0340 -28.9070

C -15.6530 -11.8200 -27.6040

H -16.6800 -11.4520 -27.6700

H -15.1280 -11.2000 -26.8730

C -15.6710 -13.2630 -27.1050

H -16.2210 -13.8870 -27.8140

H -14.6490 -13.6420 -27.0510

C -16.3200 -13.3650 -25.7260

H -17.3380 -12.9730 -25.7780

H -15.7620 -12.7400 -25.0250

C -16.3590 -14.7950 -25.1950

H -16.9420 -15.4190 -25.8780

H -15.3440 -15.1970 -25.1600

C -16.9750 -14.8650 -23.8000

H -17.9810 -14.4410 -23.8270

H -16.3810 -14.2550 -23.1140

C -17.0460 -16.2930 -23.2630

H -17.6570 -16.9010 -23.9350

H -16.0430 -16.7240 -23.2480

C -17.6390 -16.3400 -21.8600

H -17.0350 -15.7560 -21.1700

H -17.6840 -17.3650 -21.4970

H -18.6480 -15.9330 -21.8570

C -11.4260 -5.8280 -29.7030

H -12.3850 -5.3260 -29.5410

H -10.6800 -5.0710 -29.9790

C -10.9980 -6.5300 -28.4080

H -10.9850 -5.7730 -27.6220

H -9.9860 -6.9220 -28.5110

C -11.9230 -7.6760 -28.0130

H -11.9390 -8.4100 -28.8220

H -12.9390 -7.2990 -27.8730

C -11.4530 -8.3730 -26.7380

H -10.4170 -8.6950 -26.8700

H -11.4760 -7.6700 -25.9020

C -12.3140 -9.5900 -26.4100

H -13.3350 -9.2660 -26.1950

H -12.3510 -10.2440 -27.2850

C -11.7740 -10.3860 -25.2250

H -10.7440 -10.6840 -25.4340

H -11.7610 -9.7560 -24.3330

C -12.6100 -11.6360 -24.9570

H -12.6790 -12.2210 -25.8760

H -13.6240 -11.3400 -24.6780

C -12.0160 -12.5090 -23.8540

H -10.9870 -12.7680 -24.1170

H -11.9850 -11.9460 -22.9190

C -12.8180 -13.7920 -23.6520

H -13.8390 -13.5400 -23.3540

H -12.8770 -14.3280 -24.6030

C -12.1950 -14.7060 -22.6010

H -12.1570 -14.1860 -21.6410

H -11.1670 -14.9380 -22.8910

C -12.9750 -16.0080 -22.4420

H -13.9920 -15.7830 -22.1130

H -13.0440 -16.5020 -23.4150

C -12.3140 -16.9530 -21.4460

H -11.3170 -17.2280 -21.7860

H -12.9010 -17.8620 -21.3330

H -12.2240 -16.4780 -20.4720

O -12.3190 -8.8670 -32.6260

O -10.5210 -10.2810 -33.5910

O -11.6970 -6.6780 -30.8000

O -6.0460 -8.6610 -31.7660

C 1.5180 -8.9670 -30.2360

C 1.7410 -9.3410 -28.9210

H 0.9390 -9.8000 -28.3680

C 2.9930 -9.1400 -28.3520

C 4.0140 -8.5300 -29.1000

C 3.7660 -8.1090 -30.4150

C 2.5140 -8.3460 -30.9730

H 2.2990 -8.0490 -31.9870

C 2.3960 -9.8920 -26.1430

H 1.7690 -9.0190 -25.9170

H 1.7520 -10.7050 -26.5020

C 3.1860 -10.3220 -24.9050

H 3.4940 -11.3630 -25.0190

H 4.0890 -9.7090 -24.8890

C 2.4480 -10.1410 -23.5820

H 2.1470 -9.0960 -23.4790

H 3.1530 -10.3590 -22.7760

C 1.2220 -11.0380 -23.4040

H 0.4190 -10.7100 -24.0670

H 1.4760 -12.0660 -23.6720

C 0.7420 -10.9940 -21.9530

H 0.6660 -9.9530 -21.6330

H 1.4890 -11.4770 -21.3170

C -0.6110 -11.6650 -21.7320

H -1.3730 -11.1350 -22.3080

H -0.5810 -12.6950 -22.0940

C -0.9890 -11.6490 -20.2500

H -0.7830 -10.6560 -19.8440

H -0.3600 -12.3580 -19.7080

C -2.4590 -11.9810 -20.0030

H -3.0830 -11.2390 -20.5050

H -2.6960 -12.9550 -20.4370

C -2.7900 -11.9880 -18.5110

H -2.3630 -11.0960 -18.0450

H -2.3230 -12.8550 -18.0370

C -4.2940 -12.0080 -18.2520

H -4.7390 -12.8730 -18.7500

H -4.7390 -11.1100 -18.6860

C -4.6200 -12.0550 -16.7610

H -4.2780 -13.0050 -16.3430

H -4.0760 -11.2570 -16.2500

C -6.1120 -11.8910 -16.5020

H -6.6740 -12.6920 -16.9780

H -6.3220 -11.9100 -15.4340

H -6.4620 -10.9440 -16.9070

C 6.3070 -9.0840 -29.0150

H 6.4670 -8.8410 -30.0730

H 7.1790 -8.7540 -28.4450

C 6.0990 -10.5890 -28.8470

H 5.9390 -10.8020 -27.7890

H 5.1890 -10.8820 -29.3800

C 7.2820 -11.3930 -29.3770

H 8.1890 -11.0940 -28.8470

H 7.4280 -11.1660 -30.4360

C 7.0670 -12.8940 -29.2070

H 6.9290 -13.1200 -28.1470

H 6.1510 -13.1890 -29.7260

C 8.2360 -13.7120 -29.7500

H 9.1520 -13.4220 -29.2300

H 8.3730 -13.4850 -30.8100

C 8.0110 -15.2120 -29.5810

H 7.8740 -15.4390 -28.5210

H 7.0940 -15.4990 -30.1000

C 9.1730 -16.0370 -30.1260

H 10.0930 -15.7560 -29.6080

H 9.3120 -15.8090 -31.1860

C 8.9350 -17.5350 -29.9600

H 8.8220 -17.7670 -28.8980

H 8.0030 -17.8080 -30.4590

C 10.0740 -18.3700 -30.5390

H 11.0120 -18.0970 -30.0480

H 10.1840 -18.1430 -31.6030

C 9.8290 -19.8670 -30.3660

H 9.7580 -20.1010 -29.3010

H 8.8730 -20.1300 -30.8240

C 10.9350 -20.7110 -30.9940

H 11.8910 -20.4580 -30.5290

H 11.0140 -20.4700 -32.0570

C 10.6710 -22.2030 -30.8330

H 9.7310 -22.4770 -31.3060

H 11.4680 -22.7860 -31.2890

H 10.6120 -22.4670 -29.7790

C 4.8720 -7.3400 -32.4350

H 3.8880 -7.2850 -32.9050

H 5.3900 -6.3950 -32.6230

C 5.6870 -8.4890 -33.0370

H 5.8050 -8.2940 -34.1040

H 6.6800 -8.4830 -32.5810

C 5.0460 -9.8580 -32.8240

H 4.8710 -10.0150 -31.7580

H 4.0760 -9.8940 -33.3260

C 5.9340 -10.9840 -33.3470

H 6.9280 -10.8920 -32.9010

H 6.0500 -10.8880 -34.4290

C 5.3680 -12.3620 -33.0130

H 4.3910 -12.4790 -33.4870

H 5.2200 -12.4340 -31.9330

C 6.2940 -13.4860 -33.4690

H 7.2770 -13.3450 -33.0130

H 6.4240 -13.4330 -34.5520

C 5.7610 -14.8640 -33.0890

H 5.6140 -14.9070 -32.0070

H 4.7870 -15.0190 -33.5600

C 6.7130 -15.9800 -33.5130

H 7.6870 -15.8160 -33.0470

H 6.8540 -15.9410 -34.5960

C 6.1970 -17.3620 -33.1220

H 5.2170 -17.5250 -33.5760

H 6.0670 -17.4050 -32.0370

C 7.1490 -18.4710 -33.5630

H 7.2610 -18.4370 -34.6490

H 8.1340 -18.2980 -33.1240

C 6.6540 -19.8560 -33.1530

H 5.6550 -20.0200 -33.5650

H 6.5710 -19.9020 -32.0650

C 7.5880 -20.9580 -33.6400

H 8.5900 -20.8100 -33.2450

H 7.2310 -21.9340 -33.3190

H 7.6460 -20.9550 -34.7260

O 5.2300 -8.3280 -28.4890

O 3.3640 -9.5400 -27.1120

O 4.7840 -7.4320 -31.0250

O -0.8440 -9.3290 -29.9800

C -2.6570 -8.8990 -25.5320

C -1.4570 -8.6300 -24.8660

C -3.8350 -9.0660 -24.7940

C -1.4410 -8.5020 -23.4940

H -0.5540 -8.5010 -25.4440

C -3.8130 -8.9480 -23.4250

H -4.7570 -9.2800 -25.3130

C -2.6190 -8.6530 -22.7640

H -0.5230 -8.2770 -22.9750

H -4.7120 -9.0670 -22.8400

C -4.1970 -9.3430 -36.1970

C -2.9950 -9.3710 -36.9140

C -5.4110 -9.2800 -36.8870

C -3.0100 -9.3060 -38.2870

H -2.0600 -9.4300 -36.3770

C -5.4210 -9.2080 -38.2630

H -6.3350 -9.2840 -36.3300

C -4.2210 -9.2090 -38.9730

H -2.0930 -9.3130 -38.8560

H -6.3540 -9.1540 -38.8030

C -7.0070 -8.1080 -30.8880

H -6.7310 -7.0740 -30.6380

H -7.0380 -8.6860 -29.9520

C 0.2090 -9.2710 -30.9170

H 0.0010 -8.4970 -31.6680

H 0.2960 -10.2390 -31.4390

C -3.9920 -8.7780 -44.2510

C -4.0430 -8.3580 -49.9590

C -4.1740 -8.2710 -51.1620

C -4.4450 -8.1660 -52.5360

C -3.4550 -8.3930 -53.5160

C -5.7410 -7.8060 -52.9180

H -6.4370 -7.5810 -52.1260

O -5.0140 -8.5760 -43.6070

O -2.8650 -9.1830 -43.7130

H -2.9310 -9.2300 -42.7110

C -5.6460 -7.7790 -63.4700

C -5.5040 -8.0150 -57.8110

C -5.4260 -8.0350 -56.6030

C -5.1530 -8.0600 -55.2250

C -6.1140 -7.7230 -54.2490

C -3.8480 -8.3810 -54.8440

H -3.1520 -8.6110 -55.6350

O -4.6410 -7.9880 -64.1380

O -6.8150 -7.4980 -63.9960

H -6.7850 -7.4340 -64.9950

C -9.6370 -6.7420 -54.4460

C -9.7720 -6.6550 -55.8210

H -8.8860 -6.6020 -56.4320

C -11.0360 -6.6440 -56.3900

C -12.1960 -6.7460 -55.5930

C -12.0480 -6.8200 -54.2020

C -10.7690 -6.7980 -53.6510

H -10.6480 -6.8450 -52.5810

C -10.2700 -7.0630 -58.5990

H -9.7960 -7.9460 -58.1510

H -9.4980 -6.2970 -58.7720

C -10.9340 -7.4340 -59.9230

H -10.1810 -7.8980 -60.5650

H -11.2830 -6.5210 -60.4060

C -12.1240 -8.3760 -59.7430

H -12.8720 -7.8790 -59.1250

H -12.5630 -8.5600 -60.7270

C -11.7510 -9.7140 -59.1060

H -11.5700 -9.5750 -58.0380

H -10.8280 -10.0900 -59.5570

C -12.8540 -10.7510 -59.2980

H -13.7960 -10.3570 -58.9110

H -12.9870 -10.9310 -60.3680

C -12.5330 -12.0760 -58.6090

H -12.5920 -11.9500 -57.5250

H -11.5080 -12.3680 -58.8490

C -13.4810 -13.1880 -59.0520

H -14.5140 -12.8830 -58.8660

H -13.3720 -13.3350 -60.1290

C -13.2050 -14.5110 -58.3410

H -13.4390 -14.4120 -57.2780

H -12.1410 -14.7480 -58.4200

C -14.0180 -15.6540 -58.9430

H -15.0800 -15.4020 -58.9050

H -13.7470 -15.7670 -59.9950

C -13.7840 -16.9800 -58.2230

H -12.7150 -17.2060 -58.2200

H -14.1030 -16.8900 -57.1820

C -14.5390 -18.1280 -58.8880

H -14.2050 -18.2250 -59.9240

H -15.6060 -17.8930 -58.9100

C -14.3240 -19.4500 -58.1610

H -13.2690 -19.7150 -58.1560

H -14.8770 -20.2500 -58.6470

H -14.6620 -19.3760 -57.1300

C -14.6110 -6.4520 -55.8090

H -15.1070 -5.9680 -56.6590

H -14.5040 -5.7250 -55.0030

C -15.4700 -7.6260 -55.3330

H -16.4790 -7.2470 -55.1570

H -15.0800 -7.9910 -54.3850

C -15.5110 -8.7720 -56.3410

H -15.9000 -8.4090 -57.2950

H -14.4930 -9.1250 -56.5150

C -16.3760 -9.9220 -55.8310

H -17.4110 -9.5830 -55.7440

H -16.0390 -10.2060 -54.8320

C -16.3220 -11.1500 -56.7370

H -16.6650 -10.8820 -57.7390

H -15.2870 -11.4890 -56.8230

C -17.1810 -12.2870 -56.1890

H -18.2210 -11.9560 -56.1280

H -16.8520 -12.5270 -55.1760

C -17.1040 -13.5470 -57.0480

H -17.4380 -13.3170 -58.0630

H -16.0640 -13.8750 -57.1100

C -17.9540 -14.6790 -56.4770

H -18.9960 -14.3550 -56.4210

H -17.6220 -14.8980 -55.4600

C -17.8650 -15.9500 -57.3180

H -18.1990 -15.7350 -58.3360

H -16.8220 -16.2700 -57.3750

C -18.7060 -17.0830 -56.7380

H -19.7500 -16.7650 -56.6780

H -18.3680 -17.2980 -55.7210

C -18.6170 -18.3560 -57.5760

H -18.9620 -18.1450 -58.5910

H -17.5740 -18.6730 -57.6430

C -19.4490 -19.4870 -56.9830

H -19.1080 -19.7240 -55.9780

H -19.3680 -20.3830 -57.5940

H -20.4980 -19.2010 -56.9300

C -13.0950 -7.4850 -52.1410

H -14.1230 -7.4400 -51.7720

H -12.4730 -6.8520 -51.4940

C -12.5980 -8.9330 -52.1000

H -12.5670 -9.2440 -51.0540

H -11.5850 -8.9890 -52.5010

C -13.5000 -9.8750 -52.8940

H -13.5000 -9.5700 -53.9420

H -14.5230 -9.7930 -52.5170

C -13.0450 -11.3290 -52.7990

H -12.0510 -11.4300 -53.2390

H -12.9720 -11.6160 -51.7470

C -14.0170 -12.2690 -53.5070

H -15.0090 -12.1530 -53.0620

H -14.0930 -11.9880 -54.5600

C -13.5950 -13.7320 -53.4020

H -12.6550 -13.8820 -53.9390

H -13.4160 -13.9790 -52.3520

C -14.6600 -14.6730 -53.9590

H -14.8410 -14.4370 -55.0100

H -15.5960 -14.5080 -53.4190

C -14.2620 -16.1410 -53.8280

H -13.3800 -16.3360 -54.4440

H -13.9900 -16.3480 -52.7900

C -15.3930 -17.0780 -54.2440

H -16.2700 -16.8780 -53.6240

H -15.6710 -16.8740 -55.2810

C -15.0060 -18.5480 -54.1050

H -14.7000 -18.7460 -53.0750

H -14.1470 -18.7580 -54.7470

C -16.1570 -19.4800 -54.4760

H -17.0020 -19.2890 -53.8110

H -16.4840 -19.2610 -55.4950

C -15.7590 -20.9470 -54.3790

H -14.9320 -21.1640 -55.0520

H -16.5950 -21.5900 -54.6470

H -15.4480 -21.1930 -53.3660

O -13.3480 -6.8340 -56.3170

O -11.2620 -6.5600 -57.7280

O -13.1850 -6.9390 -53.4410

O -7.3400 -7.3600 -54.7050

C 0.1820 -8.1880 -53.4570

C 0.2900 -7.6910 -52.1720

H -0.6060 -7.5570 -51.5920

C 1.5480 -7.3960 -51.6500

C 2.6860 -7.5230 -52.4630

C 2.5630 -7.9870 -53.7800

C 1.3060 -8.3440 -54.2550

H 1.1780 -8.7230 -55.2570

C 0.7100 -6.6920 -49.5280

H 0.2750 -5.7420 -49.8630

H -0.0720 -7.4590 -49.5770

C 1.2520 -6.5930 -48.0980

H 2.3390 -6.5350 -48.1600

H 0.8960 -5.6700 -47.6420

C 0.8320 -7.7650 -47.2110

H -0.2580 -7.8540 -47.2310

H 1.1210 -7.5440 -46.1800

C 1.4550 -9.0990 -47.6160

H 1.2480 -9.3060 -48.6670

H 2.5390 -9.0390 -47.5010

C 0.9080 -10.2320 -46.7510

H -0.1680 -10.3200 -46.9190

H 1.0520 -9.9770 -45.6980

C 1.5600 -11.5850 -47.0240

H 1.4110 -11.8610 -48.0710

H 2.6360 -11.5120 -46.8540

C 0.9770 -12.6670 -46.1180

H -0.0950 -12.7600 -46.3110

H 1.0950 -12.3570 -45.0770

C 1.6400 -14.0290 -46.3060

H 1.4910 -14.3700 -47.3330

H 2.7150 -13.9340 -46.1410

C 1.0720 -15.0630 -45.3360

H -0.0030 -15.1660 -45.5060

H 1.2070 -14.7030 -44.3130

C 1.7370 -16.4300 -45.4740

H 2.8140 -16.3230 -45.3260

H 1.5740 -16.8100 -46.4860

C 1.1890 -17.4330 -44.4610

H 1.3440 -17.0420 -43.4530

H 0.1120 -17.5430 -44.6100

C 1.8580 -18.7970 -44.5800

H 2.9310 -18.7100 -44.4250

H 1.4600 -19.4840 -43.8360

H 1.6850 -19.2230 -45.5660

C 4.8150 -8.1830 -51.6620

H 5.0890 -8.7160 -52.5820

H 5.7000 -7.6750 -51.2700

C 4.2800 -9.1660 -50.6230

H 4.0130 -8.6060 -49.7250

H 3.3660 -9.6310 -51.0050

C 5.3050 -10.2450 -50.2910

H 6.2200 -9.7740 -49.9240

H 5.5590 -10.7960 -51.2000

C 4.7800 -11.2180 -49.2400

H 4.5510 -10.6650 -48.3260

H 3.8480 -11.6650 -49.5930

C 5.7780 -12.3280 -48.9210

H 6.7080 -11.8850 -48.5540

H 6.0130 -12.8770 -49.8350

C 5.2290 -13.2970 -47.8780

H 4.9900 -12.7450 -46.9660

H 4.3010 -13.7350 -48.2510

C 6.2100 -14.4170 -47.5410

H 7.1230 -13.9880 -47.1220

H 6.4840 -14.9460 -48.4560

C 5.6090 -15.4080 -46.5480

H 5.3220 -14.8760 -45.6370

H 4.7030 -15.8390 -46.9790

C 6.5740 -16.5350 -46.1870

H 7.4680 -16.1140 -45.7210

H 6.8900 -17.0460 -47.1000

C 5.9330 -17.5460 -45.2400

H 5.6170 -17.0360 -44.3260

H 5.0400 -17.9610 -45.7120

C 6.8810 -18.6860 -44.8760

H 7.7610 -18.2780 -44.3740

H 7.2220 -19.1770 -45.7910

C 6.2090 -19.7130 -43.9720

H 5.3470 -20.1530 -44.4700

H 6.9020 -20.5110 -43.7170

H 5.8700 -19.2450 -43.0500

C 3.8720 -8.8210 -55.6430

H 2.9250 -8.9940 -56.1600

H 4.5350 -8.2690 -56.3170

C 4.5200 -10.1570 -55.2680

H 4.7260 -10.7090 -56.1880

H 5.4750 -9.9510 -54.7790

C 3.6480 -10.9970 -54.3380

H 3.3870 -10.4080 -53.4570

H 2.7170 -11.2610 -54.8450

C 4.3630 -12.2670 -53.8850

H 5.3320 -11.9990 -53.4550

H 4.5550 -12.9080 -54.7480

C 3.5540 -13.0370 -52.8450

H 2.5950 -13.3330 -53.2760

H 3.3460 -12.3800 -51.9970

C 4.2940 -14.2760 -52.3500

H 5.2610 -13.9760 -51.9390

H 4.4900 -14.9420 -53.1940

C 3.5080 -15.0320 -51.2820

H 3.3070 -14.3630 -50.4420

H 2.5460 -15.3440 -51.6940

C 4.2670 -16.2570 -50.7820

H 5.2220 -15.9390 -50.3570

H 4.4870 -16.9140 -51.6270

C 3.4850 -17.0380 -49.7300

H 2.5270 -17.3530 -50.1520

H 3.2710 -16.3860 -48.8800

C 4.2520 -18.2660 -49.2490

H 4.4730 -18.9110 -50.1030

H 5.2060 -17.9500 -48.8200

C 3.4740 -19.0620 -48.2050

H 2.5130 -19.3680 -48.6260

H 3.2670 -18.4180 -47.3470

C 4.2390 -20.2950 -47.7410

H 5.1990 -20.0100 -47.3170

H 3.6750 -20.8340 -46.9830

H 4.4210 -20.9680 -48.5760

O 3.9050 -7.1350 -51.9530

O 1.7930 -7.0210 -50.3770

O 3.7150 -7.9840 -54.5140

O -2.1800 -8.5840 -53.0920

C -4.0060 -8.5860 -48.5720

C -2.9650 -8.8940 -47.8910

C -5.1820 -8.3670 -47.8770

C -2.9570 -8.9880 -46.4650

H -2.1280 -9.0560 -48.4340

C -5.1650 -8.4520 -46.3360

H -6.0720 -8.1550 -48.2950

C -3.9840 -8.7650 -45.7180

H -2.1120 -9.2330 -45.9800

H -6.0310 -8.2730 -45.8500

C -5.5620 -7.9620 -59.2160

C -4.3920 -8.1490 -59.9610

C -6.7670 -7.7130 -59.8800

C -4.4310 -8.0880 -61.3350

H -3.4620 -8.3370 -59.4470

C -6.8030 -7.6510 -61.2550

H -7.6680 -7.5760 -59.3040

C -5.6350 -7.8380 -61.9960

H -3.5370 -8.2280 -61.9240

H -7.7320 -7.4640 -61.7700

C -8.2770 -6.8150 -53.7970

H -7.9420 -5.8130 -53.4850

H -8.3580 -7.4440 -52.9000

C -1.1580 -8.5230 -54.0650

H -1.3950 -7.7420 -54.8020

H -1.0920 -9.4850 -54.5950

C -2.5150 -9.0970 -3.8430

C -2.5910 -9.0100 -5.0380

C -2.7050 -8.9060 -6.4430

C -1.5600 -8.6650 -7.2280

C -3.9580 -9.0250 -7.0430

H -4.8070 -9.1850 -6.3970

C -2.8470 -8.3770 -17.4740

C -2.9860 -8.7110 -11.8210

C -3.0250 -8.7500 -10.6130

C -2.9550 -8.7840 -9.2090

C -4.1120 -8.9260 -8.4180

C -1.7050 -8.6490 -8.6060

H -0.8530 -8.5280 -9.2560

O -1.8050 -8.1820 -18.0930

O -4.0070 -8.5390 -18.0590

H -3.9280 -8.5560 -19.0650

C -7.6610 -8.5060 -9.1910

C -7.5080 -8.1350 -10.5160

H -6.5230 -8.1450 -10.9530

C -8.6130 -7.7240 -11.2490

C -9.9020 -7.7380 -10.6800

C -10.0510 -8.1640 -9.3530

C -8.9240 -8.5210 -8.6220

H -9.0250 -8.8220 -7.5910

C -7.5650 -7.8230 -13.3860

H -7.4840 -8.9030 -13.2090

H -6.5860 -7.3590 -13.1980

C -7.9780 -7.5440 -14.8290

H -7.2600 -8.0300 -15.4910

H -7.9200 -6.4680 -14.9920

C -9.3990 -8.0060 -15.1520

H -10.0900 -7.5160 -14.4650

H -9.6380 -7.6760 -16.1670

C -9.5910 -9.5180 -15.0690

H -9.5920 -9.8350 -14.0250

H -8.7630 -10.0230 -15.5740

C -10.9040 -9.9370 -15.7270

H -11.7350 -9.4180 -15.2420

H -10.8860 -9.6230 -16.7740

C -11.1410 -11.4450 -15.6720

H -11.3480 -11.7510 -14.6440

H -10.2340 -11.9630 -15.9930

C -12.2970 -11.8590 -16.5800

H -13.2040 -11.3290 -16.2800

H -12.0640 -11.5540 -17.6030

C -12.5560 -13.3630 -16.5630

H -12.9140 -13.6650 -15.5750

H -11.6180 -13.8930 -16.7460

C -13.5750 -13.7680 -17.6250

H -14.5060 -13.2200 -17.4600

H -13.1970 -13.4790 -18.6090

C -13.8610 -15.2670 -17.6210

H -12.9230 -15.8140 -17.7520

H -14.2740 -15.5560 -16.6510

C -14.8350 -15.6660 -18.7260

H -14.4150 -15.3810 -19.6930

H -15.7690 -15.1130 -18.6000

C -15.1210 -17.1620 -18.7190

H -14.2010 -17.7260 -18.8570

H -15.8080 -17.4270 -19.5180

H -15.5640 -17.4600 -17.7720

C -12.0410 -6.6710 -11.0900

H -12.1450 -5.8460 -11.8040

H -11.8740 -6.2510 -10.0950

C -13.3220 -7.5080 -11.1040

H -14.1660 -6.8360 -10.9320

H -13.2890 -8.2250 -10.2850

C -13.4980 -8.2520 -12.4260

H -13.5510 -7.5340 -13.2470

H -12.6180 -8.8750 -12.5960

C -14.7510 -9.1220 -12.4360

H -15.6340 -8.4930 -12.2970

H -14.7110 -9.8220 -11.5970

C -14.8900 -9.9090 -13.7370

H -14.9750 -9.2120 -14.5750

H -13.9860 -10.5030 -13.8940

C -16.1020 -10.8370 -13.7210

H -17.0090 -10.2460 -13.5790

H -16.0170 -11.5210 -12.8740

C -16.2250 -11.6490 -15.0080

H -16.3390 -10.9690 -15.8570

H -15.3050 -12.2180 -15.1640

C -17.4080 -12.6140 -14.9690

H -18.3300 -12.0500 -14.8070

H -17.2850 -13.2960 -14.1250

C -17.5320 -13.4250 -16.2560

H -17.6730 -12.7460 -17.1010

H -16.6020 -13.9730 -16.4260

C -18.6920 -14.4160 -16.2050

H -19.6230 -13.8740 -16.0240

H -18.5420 -15.1010 -15.3670

C -18.8210 -15.2220 -17.4960

H -18.9910 -14.5400 -18.3320

H -17.8850 -15.7510 -17.6880

C -19.9600 -16.2310 -17.4270

H -19.7950 -16.9350 -16.6130

H -20.0310 -16.7920 -18.3560

H -20.9080 -15.7270 -17.2550

C -11.6190 -9.1470 -7.8300

H -12.6660 -8.9480 -7.5840

H -11.0190 -8.9700 -6.9280

C -11.4590 -10.5930 -8.3060

H -11.7020 -11.2560 -7.4730

H -10.4190 -10.7670 -8.5900

C -12.3690 -10.8880 -9.4960

H -12.1080 -10.2180 -10.3160

H -13.4040 -10.6830 -9.2080

C -12.2660 -12.3310 -9.9820

H -11.2580 -12.5220 -10.3560

H -12.4420 -13.0120 -9.1440

C -13.2870 -12.6130 -11.0820

H -14.2890 -12.4240 -10.6880

H -13.1240 -11.9220 -11.9120

C -13.2230 -14.0480 -11.5980

H -12.2730 -14.2120 -12.1130

H -13.2620 -14.7380 -10.7520

C -14.3810 -14.3550 -12.5450

H -14.3560 -13.6600 -13.3870

H -15.3230 -14.1920 -12.0140

C -14.3470 -15.7890 -13.0660

H -13.4710 -15.9270 -13.7040

H -14.2500 -16.4760 -12.2210

C -15.6110 -16.1380 -13.8490

H -16.4800 -16.0020 -13.2000

H -15.7180 -15.4490 -14.6900

C -15.5950 -17.5740 -14.3660

H -15.4350 -18.2580 -13.5290

H -14.7600 -17.7020 -15.0590

C -16.8990 -17.9410 -15.0710

H -17.7310 -17.8200 -14.3730

H -17.0650 -17.2530 -15.9020

C -16.8830 -19.3730 -15.5930

H -16.0650 -19.5160 -16.2950

H -17.8160 -19.6060 -16.1020

H -16.7550 -20.0760 -14.7730

O -10.9100 -7.4010 -11.5330

O -8.5500 -7.2750 -12.5320

O -11.3220 -8.1980 -8.8350

O -5.2990 -8.9510 -9.0790

C 1.7110 -7.2730 -6.4270

C 1.4020 -6.8510 -5.1460

H 0.3830 -6.9400 -4.8100

C 2.4050 -6.3530 -4.3220

C 3.7140 -6.2220 -4.8160

C 3.9990 -6.5820 -6.1410

C 2.9950 -7.1350 -6.9280

H 3.1930 -7.4490 -7.9400

C 3.3110 -5.3720 -2.3670

H 4.1800 -6.0440 -2.3590

H 3.5870 -4.4480 -2.8910

C 2.8650 -5.0690 -0.9400

H 2.1460 -4.2490 -0.9500

H 2.3500 -5.9530 -0.5640

C 4.0480 -4.7230 -0.0380

H 4.5570 -3.8500 -0.4550

H 4.7530 -5.5580 -0.0320

C 3.6310 -4.4150 1.3990

H 2.8760 -3.6260 1.3900

H 3.1770 -5.3030 1.8450

C 4.8110 -3.9620 2.2570

H 5.3020 -3.1120 1.7750

H 5.5420 -4.7710 2.3270

C 4.3770 -3.5460 3.6610

H 3.6570 -2.7270 3.5870

H 3.8790 -4.3860 4.1510

C 5.5570 -3.0870 4.5140

H 6.0560 -2.2530 4.0160

H 6.2790 -3.9020 4.6010

C 5.1180 -2.6490 5.9090

H 4.3960 -1.8340 5.8190

H 4.6160 -3.4810 6.4080

C 6.2970 -2.1870 6.7620

H 6.8000 -1.3570 6.2600

H 7.0180 -3.0020 6.8540

C 5.8570 -1.7420 8.1550

H 5.3530 -2.5720 8.6560

H 5.1360 -0.9270 8.0620

C 7.0340 -1.2780 9.0100

H 7.7540 -2.0940 9.1050

H 7.5380 -0.4490 8.5080

C 6.5860 -0.8340 10.3970

H 6.0990 -1.6530 10.9210

H 7.4380 -0.5070 10.9890

H 5.8820 -0.0080 10.3240

C 5.7580 -6.5130 -3.6470

H 6.3360 -6.7670 -4.5450

H 6.3750 -5.8920 -2.9940

C 5.3250 -7.7900 -2.9280

H 4.8220 -7.5170 -1.9990

H 4.5950 -8.3120 -3.5550

C 6.5030 -8.7150 -2.6450

H 7.2170 -8.2090 -1.9910

H 7.0170 -8.9390 -3.5840

C 6.0510 -10.0220 -1.9980

H 5.5790 -9.8050 -1.0380

H 5.2990 -10.4930 -2.6350

C 7.2060 -10.9970 -1.7870

H 7.9490 -10.5450 -1.1270

H 7.6920 -11.1920 -2.7460

C 6.7280 -12.3170 -1.1880

H 6.2650 -12.1260 -0.2170

H 5.9620 -12.7480 -1.8370

C 7.8600 -13.3260 -1.0160

H 8.6340 -12.8990 -0.3730

H 8.3160 -13.5270 -1.9890

C 7.3630 -14.6360 -0.4100

H 6.9420 -14.4370 0.5780

H 6.5620 -15.0400 -1.0330

C 8.4720 -15.6770 -0.2830

H 9.2940 -15.2620 0.3050

H 8.8660 -15.9090 -1.2760

C 7.9750 -16.9600 0.3790

H 7.6210 -16.7300 1.3870

H 7.1270 -17.3540 -0.1850

C 9.0640 -18.0270 0.4620

H 9.9270 -17.6220 0.9950

H 9.3940 -18.2860 -0.5480

C 8.5750 -19.2830 1.1740

H 7.7130 -19.7040 0.6600

H 9.3580 -20.0370 1.2050

H 8.2820 -19.0510 2.1950

C 5.8320 -6.9340 -7.6990

H 5.0830 -7.1500 -8.4650

H 6.5370 -6.2020 -8.1090

C 6.5930 -8.2090 -7.3230

H 7.1190 -8.5610 -8.2130

H 7.3410 -7.9550 -6.5690

C 5.6910 -9.3170 -6.7860

H 5.1530 -8.9580 -5.9070

H 4.9480 -9.5840 -7.5410

C 6.4970 -10.5570 -6.4080

H 7.2580 -10.2810 -5.6740

H 7.0170 -10.9350 -7.2920

C 5.6200 -11.6620 -5.8260

H 4.8650 -11.9520 -6.5610

H 5.0950 -11.2800 -4.9480

C 6.4420 -12.8870 -5.4320

H 7.1990 -12.5910 -4.7020

H 6.9650 -13.2690 -6.3120

C 5.5830 -13.9990 -4.8360

H 5.0610 -13.6200 -3.9540

H 4.8230 -14.3000 -5.5620

C 6.4220 -15.2130 -4.4460

H 7.1960 -14.9030 -3.7410

H 6.9240 -15.6040 -5.3350

C 5.5850 -16.3220 -3.8140

H 4.7940 -16.6200 -4.5060

H 5.1030 -15.9410 -2.9100

C 6.4340 -17.5400 -3.4600

H 6.8830 -17.9430 -4.3720

H 7.2480 -17.2330 -2.8000

C 5.6210 -18.6360 -2.7750

H 4.7890 -18.9270 -3.4210

H 5.1960 -18.2440 -1.8480

C 6.4730 -19.8610 -2.4660

H 7.3130 -19.5890 -1.8290

H 5.8850 -20.6200 -1.9560

H 6.8680 -20.2920 -3.3830

O 4.6600 -5.6820 -3.9800

O 2.2300 -5.9940 -3.0320

O 5.2670 -6.2930 -6.5740

O -0.3860 -8.4550 -6.5800

C -2.9520 -8.6390 -13.2260

C -1.7490 -8.3740 -13.8880

C -4.1190 -8.8290 -13.9720

C -1.7170 -8.2950 -15.2620

H -0.8440 -8.2460 -13.3120

C -4.0890 -8.7250 -15.3430

H -5.0370 -9.0660 -13.4590

C -2.8890 -8.4590 -16.0010

H -0.7890 -8.1110 -15.7810

H -4.9890 -8.8560 -15.9230

C -6.4810 -8.8530 -8.3170

H -6.3690 -8.0620 -7.5590

H -6.6750 -9.8020 -7.7950

C 0.6360 -7.8240 -7.3250

H 0.1940 -6.9880 -7.8860

H 1.0810 -8.5310 -8.0390

C -1.9980 0.4110 -49.2650

C -0.6060 0.1760 -43.7880

C -0.5340 0.0760 -42.5850

C -0.5990 -0.0200 -41.1850

C 0.5320 -0.3010 -40.3940

C -1.8520 0.1630 -40.5930

H -2.6810 0.3660 -41.2540

O -3.1830 0.4930 -49.5630

O -1.0340 0.3780 -50.1510

H -1.3950 0.4630 -51.0870

C -2.0330 0.3000 -30.2590

C -1.0530 0.0750 -35.8280

C -0.9790 -0.0480 -37.0290

C -0.8690 -0.1130 -38.4280

C -2.0190 0.0900 -39.2220

C 0.3730 -0.3420 -39.0190

H 1.2030 -0.5510 -38.3620

O -1.1540 0.1590 -29.4180

O -3.2990 0.4590 -29.9590

H -3.4650 0.4210 -28.9640

C -5.5500 -0.2770 -38.4900

C -5.5760 -0.2210 -37.1030

H -4.7610 0.2510 -36.5800

C -6.6110 -0.8340 -36.4190

C -7.6730 -1.4660 -37.1090

C -7.6720 -1.4510 -38.5080

C -6.5870 -0.8830 -39.1750

H -6.5490 -0.9310 -40.2510

C -5.8660 -0.1970 -34.2300

H -5.9390 0.8770 -34.4530

H -4.8250 -0.5150 -34.3790

C -6.2810 -0.4840 -32.7870

H -5.7120 0.1700 -32.1240

H -6.0150 -1.5150 -32.5500

C -7.7790 -0.3030 -32.5460

H -8.3220 -0.9850 -33.2010

H -7.9880 -0.5920 -31.5140

C -8.2770 1.1220 -32.7730

H -8.2020 1.3740 -33.8320

H -7.6520 1.8230 -32.2130

C -9.7290 1.2720 -32.3270

H -10.3480 0.5580 -32.8740

H -9.7980 1.0290 -31.2630

C -10.2640 2.6860 -32.5400

H -10.2620 2.9210 -33.6070

H -9.5990 3.3990 -32.0460

C -11.6750 2.8520 -31.9820

H -12.3570 2.1820 -32.5100

H -11.6770 2.5630 -30.9290

C -12.1800 4.2870 -32.1020

H -12.2350 4.5700 -33.1560

H -11.4660 4.9600 -31.6190

C -13.5510 4.4660 -31.4530

H -14.2790 3.8330 -31.9650

H -13.4990 4.1330 -30.4140

C -14.0230 5.9170 -31.4880

H -13.2900 6.5470 -30.9790

H -14.0790 6.2550 -32.5260

C -15.3860 6.0910 -30.8220

H -15.3290 5.7290 -29.7920

H -16.1240 5.4790 -31.3450

C -15.8400 7.5450 -30.8190

H -15.1350 8.1650 -30.2680

H -16.8180 7.6400 -30.3510

H -15.9090 7.9290 -31.8340

C -9.5480 -2.9680 -36.6590

H -9.6620 -3.6170 -35.7850

H -9.1920 -3.5540 -37.5120

C -10.9040 -2.3320 -36.9740

H -11.6410 -3.1350 -37.0340

H -10.8650 -1.8390 -37.9440

C -11.3220 -1.3410 -35.8910

H -11.2660 -1.8420 -34.9240

H -10.6170 -0.5080 -35.8740

C -12.7350 -0.8150 -36.1250

H -13.4370 -1.6520 -36.1200

H -12.7920 -0.3460 -37.1090

C -13.1570 0.1950 -35.0610

H -13.0240 -0.2510 -34.0730

H -12.5090 1.0730 -35.1160

C -14.6110 0.6270 -35.2350

H -15.2610 -0.2420 -35.1060

H -14.7550 0.9920 -36.2550

C -15.0250 1.7200 -34.2530

H -14.8390 1.3830 -33.2300

H -14.4140 2.6080 -34.4270

C -16.5000 2.0850 -34.4000

H -17.1110 1.1970 -34.2220

H -16.6870 2.4140 -35.4250

C -16.9240 3.1880 -33.4330

H -16.7480 2.8540 -32.4070

H -16.3090 4.0730 -33.6070

C -18.3950 3.5590 -33.5980

H -19.0120 2.6730 -33.4270

H -18.5700 3.8870 -34.6260

C -18.8250 4.6670 -32.6400

H -18.6560 4.3390 -31.6110

H -18.2100 5.5520 -32.8120

C -20.2930 5.0330 -32.8190

H -20.4770 5.3850 -33.8320

H -20.5790 5.8210 -32.1250

H -20.9290 4.1690 -32.6370

C -9.0510 -1.5350 -40.4730

H -9.9520 -2.0940 -40.7410

H -8.2680 -1.8060 -41.1910

C -9.3370 -0.0300 -40.5290

H -9.6780 0.2060 -41.5390

H -8.4200 0.5310 -40.3380

C -10.3930 0.3750 -39.5040

H -10.0300 0.1090 -38.5100

H -11.3090 -0.1910 -39.6950

C -10.7200 1.8660 -39.5050

H -9.8080 2.4410 -39.3300

H -11.1180 2.1610 -40.4790

C -11.7400 2.1750 -38.4120

H -12.6460 1.5930 -38.6020

H -11.3350 1.8470 -37.4510

C -12.1200 3.6490 -38.3110

H -11.2280 4.2470 -38.1120

H -12.5390 3.9860 -39.2620

C -13.1430 3.8650 -37.1970

H -12.7070 3.5490 -36.2460

H -14.0100 3.2280 -37.3870

C -13.6100 5.3130 -37.0800

H -12.7610 5.9550 -36.8370

H -14.0070 5.6430 -38.0430

C -14.6910 5.4640 -36.0120

H -15.5220 4.7950 -36.2500

H -14.2870 5.1530 -35.0450

C -15.2160 6.8930 -35.9040

H -15.5570 7.2280 -36.8860

H -14.4040 7.5560 -35.5940

C -16.3680 7.0000 -34.9080

H -17.1850 6.3520 -35.2340

H -16.0330 6.6410 -33.9330

C -16.8800 8.4280 -34.7720

H -16.0860 9.0880 -34.4280

H -17.7000 8.4760 -34.0590

H -17.2370 8.7980 -35.7310

O -8.5740 -2.0240 -36.2600

O -6.7320 -0.9220 -35.0710

O -8.7340 -2.0130 -39.1780

O -3.1950 0.1860 -38.5550

C 4.0940 -0.2280 -41.1430

C 3.9590 0.3710 -42.3840

H 2.9720 0.5210 -42.7880

C 5.0930 0.7830 -43.0730

C 6.3680 0.5850 -42.5160

C 6.4950 -0.0340 -41.2640

C 5.3480 -0.4390 -40.5920

H 5.4120 -0.9250 -39.6310

C 3.9010 1.5910 -45.0030

H 3.4760 0.6120 -45.2630

H 3.1560 2.1500 -44.4210

C 4.3100 2.3520 -46.2630

H 4.4780 3.3990 -46.0060

H 5.2680 1.9330 -46.5790

C 3.3230 2.2550 -47.4230

H 3.1200 1.2050 -47.6420

H 3.8080 2.6830 -48.3040

C 2.0000 2.9890 -47.2030

H 1.3840 2.4490 -46.4810

H 2.1940 3.9840 -46.7980

C 1.2500 3.1160 -48.5280

H 1.1850 2.1310 -48.9950

H 1.8270 3.7580 -49.1990

C -0.1620 3.6790 -48.3990

H -0.7670 3.0000 -47.7920

H -0.1380 4.6440 -47.8880

C -0.7960 3.8350 -49.7810

H -0.5800 2.9390 -50.3670

H -0.3340 4.6800 -50.2980

C -2.3090 4.0330 -49.7420

H -2.7700 3.1770 -49.2450

H -2.5550 4.9240 -49.1590

C -2.8820 4.1650 -51.1530

H -2.4930 3.3530 -51.7720

H -2.5400 5.1030 -51.5980

C -4.4080 4.1170 -51.1770

H -4.8090 4.9140 -50.5460

H -4.7410 3.1640 -50.7590

C -4.9590 4.2610 -52.5940

H -4.6990 5.2470 -52.9850

H -4.4880 3.5170 -53.2400

C -6.4710 4.0780 -52.6390

H -6.9650 4.8280 -52.0250

H -6.8380 4.1700 -53.6590

H -6.7430 3.0950 -52.2610

C 8.2060 2.0550 -42.7390

H 8.5900 1.8170 -41.7390

H 9.0530 2.1590 -43.4220

C 7.3870 3.3460 -42.6990

H 7.0240 3.5570 -43.7060

H 6.5120 3.1870 -42.0620

C 8.1930 4.5270 -42.1690

H 9.0650 4.6900 -42.8070

H 8.5570 4.2960 -41.1650

C 7.3520 5.8000 -42.1200

H 7.0030 6.0400 -43.1270

H 6.4690 5.6230 -41.5010

C 8.1250 6.9910 -41.5580

H 9.0020 7.1810 -42.1800

H 8.4800 6.7490 -40.5530

C 7.2610 8.2480 -41.4990

H 6.9210 8.4990 -42.5060

H 6.3750 8.0450 -40.8930

C 8.0060 9.4410 -40.9070

H 8.8960 9.6480 -41.5060

H 8.3410 9.1940 -39.8970

C 7.1270 10.6880 -40.8570

H 6.8270 10.9580 -41.8730

H 6.2180 10.4640 -40.2940

C 7.8360 11.8750 -40.2100

H 8.7560 12.0920 -40.7580

H 8.1190 11.6120 -39.1870

C 6.9530 13.1200 -40.1860

H 6.7090 13.4080 -41.2110

H 6.0140 12.8850 -39.6790

C 7.6260 14.2940 -39.4800

H 8.5660 14.5290 -39.9850

H 7.8670 14.0080 -38.4530

C 6.7360 15.5310 -39.4660

H 5.7980 15.3210 -38.9550

H 7.2290 16.3540 -38.9540

H 6.5060 15.8470 -40.4810

C 8.0530 -0.3380 -39.4420

H 7.2550 -0.8500 -38.8980

H 8.9570 -0.9500 -39.3890

C 8.3340 1.0270 -38.8050

H 8.6670 0.8570 -37.7780

H 9.1550 1.4990 -39.3490

C 7.1260 1.9600 -38.8090

H 6.7710 2.0900 -39.8330

H 6.3120 1.5110 -38.2330

C 7.4720 3.3280 -38.2270

H 8.3390 3.7320 -38.7560

H 7.7500 3.2190 -37.1760

C 6.3140 4.3150 -38.3490

H 5.4530 3.9400 -37.7910

H 6.0180 4.3900 -39.3980

C 6.6930 5.7010 -37.8340

H 7.5720 6.0560 -38.3770

H 6.9650 5.6320 -36.7780

C 5.5630 6.7130 -38.0000

H 5.2860 6.7730 -39.0550

H 4.6850 6.3720 -37.4480

C 5.9670 8.0990 -37.5050

H 6.8580 8.4280 -38.0450

H 6.2270 8.0400 -36.4450

C 4.8590 9.1310 -37.6940

H 3.9570 8.7930 -37.1780

H 4.6170 9.2120 -38.7570

C 5.2620 10.5030 -37.1590

H 5.4720 10.4240 -36.0900

H 6.1810 10.8270 -37.6520

C 4.1770 11.5540 -37.3810

H 3.2460 11.2140 -36.9220

H 3.9940 11.6630 -38.4530

C 4.5680 12.9040 -36.7910

H 5.4990 13.2560 -37.2290

H 3.7950 13.6450 -36.9810

H 4.7080 12.8230 -35.7160

O 7.4610 0.9670 -43.2580

O 5.0960 1.4200 -44.2670

O 7.7710 -0.2440 -40.8240

O 1.7040 -0.4790 -41.0580

C -0.8720 0.2740 -45.1660

C 0.0850 0.0090 -46.1500

C -2.1770 0.6120 -45.5460

C -0.2610 0.0540 -47.4840

H 1.0890 -0.2500 -45.8480

C -2.5180 0.6550 -46.8750

H -2.9100 0.8220 -44.7810

C -1.5660 0.3720 -47.8550

H 0.4630 -0.1730 -48.2510

H -3.5220 0.9040 -47.1840

C -1.2470 0.1820 -34.4400

C -0.2820 -0.2590 -33.5280

C -2.4430 0.7300 -33.9640

C -0.5310 -0.1910 -32.1760

H 0.6560 -0.6500 -33.8920

C -2.6970 0.7730 -32.6130

H -3.1570 1.1170 -34.6740

C -1.7510 0.2960 -31.7080

H 0.2110 -0.5210 -31.4650

H -3.6230 1.1820 -32.2430

C -4.4060 0.3110 -39.2790

H -4.3480 -0.2160 -40.2370

H -4.5890 1.3780 -39.4840

C 2.8910 -0.5940 -40.3090

H 2.9910 -1.6170 -39.9150

H 2.8670 0.0970 -39.4530

C -2.7590 0.3370 -26.4990

C -3.9450 0.6680 -20.9770

C -4.1210 0.6550 -19.7810

C -4.3400 0.6260 -18.3940

C -3.2560 0.8250 -17.5120

C -5.6220 0.3940 -17.8980

H -6.4060 0.2260 -18.6190

O -3.6390 0.3450 -27.3520

O -1.4820 0.2550 -26.7830

H -1.3140 0.1800 -27.7730

C -4.0630 0.3480 -7.4600

C -4.9330 0.5830 -13.0450

C -4.9430 0.5580 -14.2550

C -4.7950 0.5660 -15.6540

C -5.8770 0.3740 -16.5360

C -3.5050 0.7770 -16.1520

H -2.7130 0.9020 -15.4310

O -2.9180 0.3300 -7.0220

O -5.1210 0.2930 -6.6830

H -4.8680 0.2030 -5.7170

C -9.5140 0.1990 -16.0900

C -9.5680 0.3630 -14.7150

H -8.6500 0.4390 -14.1580

C -10.7990 0.3960 -14.0740

C -11.9980 0.3180 -14.8170

C -11.9290 0.2240 -16.2100

C -10.6840 0.1260 -16.8260

H -10.6200 0.0100 -17.8970

C -9.8770 0.8980 -11.9390

H -9.3690 1.7710 -12.3740

H -9.1690 0.0620 -11.8800

C -10.4010 1.2260 -10.5410

H -9.6030 1.7230 -9.9850

H -10.6400 0.2890 -10.0400

C -11.6590 2.0960 -10.5610

H -12.4560 1.5390 -11.0520

H -11.9610 2.2840 -9.5270

C -11.4770 3.4320 -11.2770

H -11.3470 3.2570 -12.3460

H -10.5790 3.9330 -10.9040

C -12.6850 4.3410 -11.0610

H -13.5900 3.8230 -11.3880

H -12.7910 4.5440 -9.9920

C -12.5620 5.6690 -11.8060

H -12.6190 5.4930 -12.8830

H -11.5850 6.1100 -11.5960

C -13.6510 6.6530 -11.3850

H -14.6340 6.2170 -11.5780

H -13.5730 6.8210 -10.3080

C -13.5390 7.9970 -12.1010

H -13.7550 7.8680 -13.1640

H -12.5130 8.3650 -12.0180

C -14.4880 9.0330 -11.5030

H -15.5150 8.6640 -11.5660

H -14.2520 9.1600 -10.4440

C -14.3910 10.3880 -12.2000

H -13.3520 10.7280 -12.1850

H -14.6860 10.2810 -13.2470

C -15.2720 11.4370 -11.5270

H -14.9630 11.5510 -10.4850

H -16.3090 11.0900 -11.5240

C -15.1900 12.7870 -12.2280

H -14.1690 13.1630 -12.2140

H -15.8310 13.5160 -11.7360

H -15.5060 12.6970 -13.2650

C -14.3380 -0.2040 -14.4280

H -14.7120 -0.6660 -13.5060

H -14.1640 -0.9890 -15.1680

C -15.3760 0.7960 -14.9430

H -16.3290 0.2750 -15.0570

H -15.0660 1.1530 -15.9240

C -15.5280 1.9820 -13.9930

H -15.9040 1.6330 -13.0290

H -14.5400 2.4150 -13.8210

C -16.4610 3.0500 -14.5550

H -17.4680 2.6380 -14.6560

H -16.1210 3.3330 -15.5540

C -16.5090 4.2980 -13.6770

H -16.8730 4.0310 -12.6820

H -15.4970 4.6940 -13.5620

C -17.4070 5.3780 -14.2760

H -18.4230 4.9890 -14.3740

H -17.0490 5.6240 -15.2790

C -17.4340 6.6500 -13.4320

H -17.8020 6.4140 -12.4300

H -16.4160 7.0330 -13.3270

C -18.3160 7.7280 -14.0570

H -19.3340 7.3460 -14.1580

H -17.9470 7.9550 -15.0600

C -18.3380 9.0110 -13.2300

H -18.7070 8.7880 -12.2250

H -17.3190 9.3910 -13.1280

C -19.2150 10.0870 -13.8640

H -20.2340 9.7080 -13.9680

H -18.8430 10.3090 -14.8670

C -19.2390 11.3730 -13.0410

H -19.6150 11.1530 -12.0390

H -18.2200 11.7530 -12.9330

C -20.1090 12.4440 -13.6860

H -19.7390 12.6890 -14.6790

H -20.1080 13.3510 -13.0860

H -21.1360 12.0960 -13.7800

C -13.0850 0.8060 -18.2250

H -14.1280 0.7460 -18.5480

H -12.4790 0.2010 -18.9110

C -12.6220 2.2640 -18.2540

H -12.6220 2.5940 -19.2950

H -11.6000 2.3300 -17.8760

C -13.5310 3.1650 -17.4220

H -13.5050 2.8370 -16.3810

H -14.5580 3.0630 -17.7840

C -13.1220 4.6340 -17.4950

H -12.1410 4.7650 -17.0330

H -13.0380 4.9330 -18.5430

C -14.1430 5.5330 -16.8010

H -15.1200 5.3800 -17.2680

H -14.2300 5.2430 -15.7520

C -13.7790 7.0130 -16.8920

H -12.8780 7.2070 -16.3050

H -13.5560 7.2640 -17.9320

C -14.9160 7.9050 -16.3990

H -15.1470 7.6630 -15.3600

H -15.8110 7.6960 -16.9920

C -14.5820 9.3910 -16.5150

H -13.7620 9.6350 -15.8360

H -14.2420 9.6050 -17.5310

C -15.7890 10.2700 -16.1970

H -16.6010 10.0250 -16.8860

H -16.1410 10.0530 -15.1860

C -15.4680 11.7580 -16.3120

H -15.0800 11.9690 -17.3110

H -14.6840 12.0160 -15.5950

C -16.6960 12.6290 -16.0560

H -17.4660 12.3890 -16.7950

H -17.1040 12.3970 -15.0700

C -16.3700 14.1150 -16.1310

H -15.6190 14.3790 -15.3900

H -17.2590 14.7130 -15.9460

H -15.9820 14.3720 -17.1150

O -13.1250 0.4200 -14.0550

O -10.9780 0.5370 -12.7450

O -13.1050 0.2390 -16.9270

O -7.1030 0.1920 -15.9790

C 0.2860 1.6300 -18.0260

C 0.1710 2.2870 -19.2400

H -0.8120 2.4610 -19.6440

C 1.3150 2.7200 -19.8990

C 2.5820 2.4730 -19.3440

C 2.6920 1.7910 -18.1240

C 1.5320 1.3790 -17.4760

H 1.5820 0.8490 -16.5380

C 0.1190 3.6280 -21.7650

H -0.3140 2.6590 -22.0460

H -0.6080 4.1630 -21.1410

C 0.4660 4.4340 -23.0190

H 0.3850 5.5000 -22.8020

H 1.5100 4.2190 -23.2540

C -0.3970 4.0810 -24.2290

H -0.2380 3.0330 -24.4890

H -0.0630 4.6840 -25.0770

C -1.8910 4.3150 -24.0150

H -2.2530 3.6570 -23.2210

H -2.0630 5.3450 -23.6980

C -2.6800 4.0300 -25.2920

H -2.3640 3.0620 -25.6890

H -2.4430 4.7850 -26.0450

C -4.1880 3.9920 -25.0590

H -4.4010 3.2900 -24.2490

H -4.5390 4.9770 -24.7450

C -4.9420 3.5470 -26.3110

H -4.5070 2.6150 -26.6750

H -4.8200 4.2950 -27.0980

C -6.4290 3.3190 -26.0480

H -6.5410 2.6660 -25.1800

H -6.9120 4.2700 -25.8090

C -7.1230 2.6790 -27.2490

H -6.6170 1.7420 -27.4940

H -7.0270 3.3370 -28.1160

C -8.6010 2.4060 -26.9840

H -9.1060 3.3470 -26.7550

H -8.6990 1.7600 -26.1080

C -9.2910 1.7450 -28.1750

H -9.1750 2.3800 -29.0570

H -8.8030 0.7910 -28.3910

C -10.7750 1.5130 -27.9110

H -11.2780 2.4590 -27.7210

H -11.2510 1.0380 -28.7660

H -10.9140 0.8750 -27.0410

C 4.4560 3.9150 -19.4660

H 4.8330 3.6030 -18.4850

H 5.3050 4.0450 -20.1420

C 3.6700 5.2200 -19.3430

H 3.3060 5.5010 -20.3340

H 2.7950 5.0480 -18.7100

C 4.5170 6.3440 -18.7540

H 5.3960 6.5040 -19.3830

H 4.8690 6.0490 -17.7630

C 3.7290 7.6470 -18.6440

H 3.3870 7.9450 -19.6370

H 2.8420 7.4810 -18.0270

C 4.5580 8.7750 -18.0350

H 5.4440 8.9450 -18.6510

H 4.9000 8.4760 -17.0410

C 3.7610 10.0720 -17.9250

H 3.4240 10.3740 -18.9190

H 2.8720 9.8960 -17.3150

C 4.5760 11.2030 -17.3040

H 5.4680 11.3830 -17.9100

H 4.9110 10.9030 -16.3080

C 3.7680 12.4930 -17.1970

H 3.4600 12.8100 -18.1970

H 2.8620 12.3020 -16.6190

C 4.5570 13.6180 -16.5330

H 5.4710 13.8060 -17.1020

H 4.8550 13.3060 -15.5290

C 3.7440 14.9070 -16.4400

H 3.4830 15.2420 -17.4470

H 2.8110 14.7060 -15.9090

C 4.5020 16.0190 -15.7200

H 5.4310 16.2280 -16.2550

H 4.7710 15.6810 -14.7160

C 3.6760 17.2950 -15.6200

H 2.7530 17.1110 -15.0740

H 4.2320 18.0730 -15.1010

H 3.4190 17.6610 -16.6120

C 4.2410 1.3160 -16.3230

H 3.4210 0.8030 -15.8150

H 5.1140 0.6580 -16.3220

C 4.5950 2.6130 -15.5870

H 4.9110 2.3530 -14.5740

H 5.4440 3.0750 -16.0950

C 3.4400 3.6100 -15.5300

H 3.0940 3.8210 -16.5430

H 2.6020 3.1740 -14.9810

C 3.8630 4.9190 -14.8680

H 4.7540 5.3010 -15.3720

H 4.1320 4.7320 -13.8260

C 2.7660 5.9770 -14.9310

H 1.8840 5.6230 -14.3930

H 2.4770 6.1300 -15.9730

C 3.2260 7.3070 -14.3390

H 4.1270 7.6370 -14.8610

H 3.4900 7.1640 -13.2880

C 2.1600 8.3930 -14.4490

H 1.8830 8.5200 -15.4990

H 1.2630 8.0790 -13.9090

C 2.6490 9.7270 -13.8920

H 3.5540 10.0280 -14.4250

H 2.9140 9.6010 -12.8390

C 1.6030 10.8300 -14.0190

H 0.6900 10.5260 -13.5000

H 1.3490 10.9680 -15.0730

C 2.0970 12.1530 -13.4390

H 2.3250 12.0170 -12.3790

H 3.0230 12.4430 -13.9400

C 1.0710 13.2730 -13.5930

H 0.1330 12.9720 -13.1210

H 0.8680 13.4330 -14.6540

C 1.5570 14.5740 -12.9650

H 2.4950 14.8890 -13.4160

H 0.8240 15.3660 -13.1060

H 1.7200 14.4450 -11.8980

O 3.6880 2.8800 -20.0560

O 1.3260 3.4250 -21.0590

O 3.9610 1.5380 -17.6910

O -2.0470 1.0550 -18.0840

C -3.6700 0.6320 -22.3550

C -2.3390 0.6600 -22.7830

C -4.6980 0.5350 -23.3000

C -2.0420 0.5770 -24.1230

H -1.5540 0.7230 -22.0430

C -4.3950 0.4390 -24.6390

H -5.7250 0.5250 -22.9660

C -3.0660 0.4530 -25.0610

H -1.0200 0.6050 -24.4610

H -5.1730 0.3470 -25.3810

C -4.7790 0.5660 -11.6460

C -3.4730 0.5720 -11.1360

C -5.8630 0.5200 -10.7640

C -3.2600 0.5060 -9.7810

H -2.6410 0.6190 -11.8230

C -5.6450 0.4570 -9.4030

H -6.8700 0.5280 -11.1520

C -4.3440 0.4400 -8.9030

H -2.2610 0.4990 -9.3730

H -6.4800 0.4110 -8.7220

C -8.2120 0.1350 -16.8480

H -8.1830 -0.7940 -17.4400

H -8.1800 0.9820 -17.5500

C -0.9340 1.2370 -17.2350

H -0.7300 0.3130 -16.6790

H -1.1460 2.0400 -16.5090

C -4.2490 1.2770 -66.2200

C -4.3730 1.2820 -65.0250

C -4.5310 1.2850 -63.6210

C -3.3940 1.2880 -62.7890

C -5.8110 1.2790 -63.0710

H -6.6430 1.2580 -63.7560

C -3.3140 0.7660 -52.8350

C -4.8320 1.2910 -58.2570

C -4.9400 1.3230 -59.4620

C -4.8700 1.3250 -60.8660

C -6.0050 1.2800 -61.6960

C -3.5870 1.3360 -61.4210

H -2.7520 1.3790 -60.7390

O -2.1260 0.7060 -52.5410

O -4.2730 0.6930 -51.9420

H -3.8930 0.6160 -51.0090

C -9.6150 1.0780 -61.0410

C -9.5710 1.0570 -59.6560

H -8.6170 1.1240 -59.1610

C -10.7460 0.9260 -58.9310

C -11.9950 0.8650 -59.5890

C -12.0300 0.9440 -60.9840

C -10.8340 1.0190 -61.6930

H -10.8510 1.0480 -62.7720

C -9.6780 1.1630 -56.8170

H -9.2690 2.1390 -57.1130

H -8.9150 0.3930 -56.9800

C -10.0870 1.1800 -55.3430

H -9.2680 1.6170 -54.7670

H -10.2260 0.1520 -55.0120

C -11.3840 1.9500 -55.0940

H -12.1970 1.4330 -55.6050

H -11.5920 1.9350 -54.0210

C -11.3440 3.3980 -55.5770

H -11.2930 3.4140 -56.6660

H -10.4490 3.8920 -55.1880

C -12.5810 4.1710 -55.1260

H -13.4770 3.6420 -55.4580

H -12.6030 4.2060 -54.0340

C -12.6030 5.5970 -55.6710

H -12.7290 5.5720 -56.7560

H -11.6430 6.0760 -55.4630

C -13.7180 6.4300 -55.0420

H -14.6820 5.9510 -55.2270

H -13.5680 6.4580 -53.9600

C -13.7470 7.8620 -55.5740

H -14.0400 7.8570 -56.6260

H -12.7410 8.2860 -55.5160

C -14.7040 8.7430 -54.7750

H -15.7090 8.3150 -54.8110

H -14.3900 8.7490 -53.7280

C -14.7430 10.1790 -55.2910

H -13.7270 10.5810 -55.3130

H -15.1210 10.1880 -56.3160

C -15.6180 11.0740 -54.4180

H -15.2260 11.0740 -53.3980

H -16.6300 10.6640 -54.3790

C -15.6690 12.5050 -54.9390

H -14.6730 12.9420 -54.9540

H -16.3040 13.1220 -54.3070

H -16.0660 12.5270 -55.9510

C -14.2680 0.1580 -59.1040

H -14.5290 -0.4610 -58.2360

H -14.1240 -0.4940 -59.9670

C -15.4040 1.1470 -59.3700

H -16.3320 0.5820 -59.4790

H -15.2110 1.6630 -60.3100

C -15.5330 2.1690 -58.2440

H -15.7900 1.6580 -57.3130

H -14.5630 2.6470 -58.0970

C -16.5830 3.2320 -58.5540

H -17.5680 2.7630 -58.6200

H -16.3650 3.6760 -59.5280

C -16.6170 4.3380 -57.5030

H -16.8570 3.9080 -56.5270

H -15.6250 4.7910 -57.4270

C -17.6380 5.4180 -57.8510

H -18.6330 4.9690 -57.9080

H -17.4030 5.8270 -58.8360

C -17.6560 6.5550 -56.8320

H -17.9030 6.1550 -55.8450

H -16.6580 6.9960 -56.7680

C -18.6630 7.6390 -57.2080

H -19.6610 7.1980 -57.2710

H -18.4130 8.0310 -58.1970

C -18.6810 8.7870 -56.2030

H -18.9380 8.3990 -55.2140

H -17.6810 9.2210 -56.1360

C -19.6760 9.8760 -56.5940

H -20.6770 9.4420 -56.6650

H -19.4150 10.2640 -57.5820

C -19.6980 11.0270 -55.5910

H -19.9710 10.6430 -54.6060

H -18.6960 11.4560 -55.5100

C -20.6800 12.1180 -56.0000

H -20.4120 12.5270 -56.9720

H -20.6770 12.9280 -55.2740

H -21.6900 11.7180 -56.0640

C -13.3940 1.6530 -62.8240

H -14.4500 1.5300 -63.0790

H -12.7970 1.1860 -63.6170

C -13.0530 3.1410 -62.7120

H -13.1840 3.5900 -63.6980

H -12.0060 3.2530 -62.4240

C -13.9370 3.8550 -61.6930

H -13.7820 3.4060 -60.7100

H -14.9850 3.7110 -61.9700

C -13.6430 5.3500 -61.6090

H -12.6260 5.5030 -61.2420

H -13.7030 5.7870 -62.6090

C -14.6340 6.0620 -60.6920

H -15.6460 5.9000 -61.0730

H -14.5830 5.6240 -59.6930

C -14.3750 7.5640 -60.6010

H -13.4220 7.7430 -60.1000

H -14.2990 7.9770 -61.6110

C -15.4930 8.2870 -59.8530

H -15.5810 7.8770 -58.8450

H -16.4400 8.1010 -60.3680

C -15.2610 9.7940 -59.7730

H -14.3830 9.9950 -59.1540

H -15.0530 10.1800 -60.7740

C -16.4710 10.5260 -59.1980

H -17.3410 10.3280 -59.8290

H -16.6930 10.1360 -58.2020

C -16.2490 12.0340 -59.1120

H -15.9770 12.4160 -60.1000

H -15.4120 12.2390 -58.4400

C -17.4920 12.7680 -58.6150

H -18.3180 12.5850 -59.3080

H -17.7840 12.3650 -57.6430

C -17.2590 14.2680 -58.4900

H -16.4530 14.4730 -57.7890

H -18.1580 14.7670 -58.1340

H -16.9890 14.6940 -59.4540

O -13.0620 0.8020 -58.7420

O -10.8270 0.8750 -57.5840

O -13.2590 0.9440 -61.6070

O -7.2130 1.2470 -61.0780

C 0.0180 0.3170 -63.5150

C -0.3000 -0.1580 -64.7740

H -1.3030 -0.0220 -65.1370

C 0.6760 -0.7700 -65.5540

C 1.9750 -0.9200 -65.0470

C 2.2850 -0.4580 -63.7580

C 1.2980 0.1650 -63.0050

H 1.5050 0.5320 -62.0120

C -0.8430 -1.2100 -67.3240

H -1.5070 -1.7690 -66.6540

H -1.2000 -0.1750 -67.3900

C -0.8300 -1.8720 -68.7010

H -0.0770 -2.6600 -68.7280

H -1.8060 -2.3370 -68.8500

C -0.5800 -0.8590 -69.8160

H -1.3670 -0.1020 -69.7860

H -0.6420 -1.3720 -70.7790

C 0.7750 -0.1600 -69.7100

H 0.8410 0.3410 -68.7430

H 1.5760 -0.9000 -69.7670

C 0.9490 0.8750 -70.8190

H 0.1320 1.5980 -70.7520

H 0.8740 0.3770 -71.7900

C 2.2760 1.6270 -70.7490

H 2.3680 2.1220 -69.7780

H 3.1050 0.9210 -70.8420

C 2.3670 2.6740 -71.8570

H 1.5380 3.3760 -71.7510

H 2.2610 2.1770 -72.8250

C 3.6750 3.4610 -71.8410

H 3.7590 4.0030 -70.8970

H 4.5200 2.7700 -71.9040

C 3.7330 4.4510 -73.0020

H 2.8630 5.1080 -72.9620

H 3.6880 3.8990 -73.9450

C 4.9920 5.3110 -72.9890

H 5.8790 4.6750 -73.0660

H 5.0490 5.8490 -72.0400

C 4.9750 6.3210 -74.1320

H 4.9250 5.7870 -75.0840

H 4.0720 6.9240 -74.0370

C 6.2010 7.2250 -74.1250

H 7.1140 6.6360 -74.1950

H 6.1760 7.9140 -74.9670

H 6.2390 7.8060 -73.2060

C 3.9730 -0.7140 -66.2860

H 4.5430 -0.3340 -65.4290

H 4.6170 -1.3730 -66.8720

C 3.4840 0.4630 -67.1300

H 3.0340 0.0860 -68.0500

H 2.7030 0.9870 -66.5730

C 4.6150 1.4360 -67.4440

H 5.3810 0.9340 -68.0400

H 5.0820 1.7530 -66.5080

C 4.1120 2.6710 -68.1880

H 3.7350 2.3770 -69.1700

H 3.2760 3.1050 -67.6330

C 5.2020 3.7260 -68.3510

H 6.0300 3.3110 -68.9310

H 5.5910 3.9920 -67.3650

C 4.6810 4.9870 -69.0360

H 4.3470 4.7410 -70.0470

H 3.8140 5.3590 -68.4860

C 5.7400 6.0840 -69.1020

H 6.5980 5.7290 -69.6770

H 6.0900 6.3030 -68.0900

C 5.1990 7.3640 -69.7320

H 4.8700 7.1560 -70.7530

H 4.3250 7.6990 -69.1670

C 6.2390 8.4810 -69.7530

H 7.1060 8.1580 -70.3330

H 6.5790 8.6740 -68.7330

C 5.6810 9.7710 -70.3500

H 5.3490 9.5810 -71.3730

H 4.8090 10.0850 -69.7730

C 6.7100 10.8980 -70.3560

H 7.5850 10.5860 -70.9310

H 7.0420 11.0880 -69.3320

C 6.1420 12.1800 -70.9510

H 5.2740 12.5120 -70.3850

H 6.8850 12.9740 -70.9360

H 5.8340 12.0180 -71.9820

C 4.1400 -0.0370 -62.2500

H 3.3970 0.2430 -61.5000

H 4.8220 -0.7650 -61.8010

C 4.9440 1.1900 -62.6940

H 5.4550 1.5900 -61.8150

H 5.7080 0.8660 -63.4040

C 4.0870 2.2800 -63.3320

H 3.5850 1.8830 -64.2160

H 3.3120 2.5950 -62.6280

C 4.9300 3.4900 -63.7270

H 5.7260 3.1730 -64.4060

H 5.4080 3.9010 -62.8340

C 4.0990 4.5810 -64.3990

H 3.2690 4.8560 -63.7450

H 3.6730 4.1930 -65.3270

C 4.9310 5.8250 -64.7020

H 5.7630 5.5550 -65.3580

H 5.3600 6.2030 -63.7710

C 4.1060 6.9290 -65.3580

H 3.7180 6.5720 -66.3140

H 3.2490 7.1620 -64.7210

C 4.9230 8.1980 -65.5840

H 5.7710 7.9730 -66.2360

H 5.3270 8.5400 -64.6270

C 4.0920 9.3170 -66.2050

H 3.2320 9.5260 -65.5640

H 3.7080 8.9880 -67.1740

C 4.9020 10.5980 -66.3920

H 5.2940 10.9200 -65.4230

H 5.7560 10.3940 -67.0410

C 4.0700 11.7260 -66.9950

H 3.2060 11.9200 -66.3550

H 3.6910 11.4140 -67.9710

C 4.8800 13.0070 -67.1520

H 5.7420 12.8370 -67.7940

H 4.2730 13.7950 -67.5930

H 5.2390 13.3520 -66.1840

O 2.9180 -1.5490 -65.8360

O 0.4770 -1.2300 -66.8110

O 3.5590 -0.7160 -63.3420

O -2.1890 1.2390 -63.4130

C -4.5260 1.1940 -56.8870

C -3.1690 1.1010 -56.5460

C -5.4960 1.1600 -55.8820

C -2.7960 0.9640 -55.2320

H -2.4270 1.1290 -57.3290

C -5.1160 1.0290 -54.5630

H -6.5420 1.2310 -56.1430

C -3.7660 0.9250 -54.2290

H -1.7570 0.8810 -54.9520

H -5.8620 1.0050 -53.7850

C -8.3670 1.1660 -61.8850

H -8.3080 0.2770 -62.5320

H -8.4340 2.0540 -62.5330

C -1.0310 0.9760 -62.6540

H -1.2610 0.2990 -61.8210

H -0.6380 1.9150 -62.2350

C -2.8770 -2.8550 -0.9030

C -4.1560 -2.7700 -0.3540

C -1.7610 -2.9170 -0.0690

C -4.3190 -2.7440 1.0320

H -5.0350 -2.7210 -1.0120

C -1.9240 -2.8930 1.3160

H -0.7540 -2.9850 -0.5030

C -3.2030 -2.8060 1.8670

H -5.3280 -2.6760 1.4650

H -1.0440 -2.9420 1.9740

C -2.3870 -9.2330 -2.3140

C -1.1420 -9.1170 -1.6970

C -3.5310 -9.4430 -1.5440

C -1.0400 -9.2120 -0.3090

H -0.2400 -8.9520 -2.3040

C -3.4290 -9.5370 -0.1560

H -4.5130 -9.5350 -2.0310

C -2.1840 -9.4220 0.4620

H -0.0580 -9.1210 0.1780

H -4.3310 -9.7030 0.4520

C -4.0130 -5.2410 -68.8140

C -2.7950 -5.3300 -69.4880

C -5.2100 -5.2810 -69.5290

C -2.7740 -5.4590 -70.8770

H -1.8510 -5.2980 -68.9240

C -5.1890 -5.4110 -70.9180

H -6.1700 -5.2100 -68.9970

C -3.9720 -5.5000 -71.5920

H -1.8140 -5.5300 -71.4090

H -6.1340 -5.4430 -71.4810

C -4.0880 1.2940 -67.7520

C -5.2030 1.4860 -68.5680

C -2.8280 1.1180 -68.3230

C -5.0580 1.5000 -69.9550

H -6.1970 1.6240 -68.1180

C -2.6820 1.1340 -69.7100

H -1.9490 0.9670 -67.6790

C -3.7970 1.3250 -70.5260

H -5.9370 1.6510 -70.5990

H -1.6890 0.9950 -70.1600

C -3.3830 -2.7780 3.3960

O -4.4090 -2.7060 4.0710

O -2.2000 -2.8450 4.0550

H -2.3410 -2.8260 5.0170

C -2.0710 -9.5260 1.9940

O -2.9440 -9.7070 2.8420

O -0.7910 -9.3920 2.4260

H -0.7480 -9.4620 3.3940

C -3.9480 -5.6430 -73.1250

O -4.8770 -5.6970 -73.9290

O -2.6850 -5.7150 -73.6160

H -2.6980 -5.8040 -74.5830

C -3.6370 1.3410 -72.0580

O -4.4800 1.4930 -72.9410

O -2.3490 1.1560 -72.4430

H -2.2760 1.1700 -73.4110

1_polymer_RT

C -2.8169 19.5713 -31.2562

C -2.9639 19.6483 -36.9042

C -2.9949 19.6643 -38.1122

C -3.0519 19.6623 -39.5152

C -1.8599 19.6023 -40.2692

C -4.2939 19.7333 -40.1532

H -5.1689 19.8243 -39.5302

O -3.8609 19.5563 -30.6082

O -1.6329 19.5363 -30.6962

H -1.6869 19.5223 -29.6852

C -3.0929 18.7793 -50.5002

C -3.2789 19.1313 -44.8622

C -3.2679 19.3283 -43.6702

C -3.2109 19.5043 -42.2782

C -4.3949 19.6843 -41.5332

C -1.9669 19.5063 -41.6472

H -1.0949 19.4143 -42.2752

O -2.0319 18.8473 -51.1042

O -4.2549 18.6853 -51.1102

H -4.1369 18.6933 -52.1102

C -7.8039 20.4903 -42.5862

C -7.5429 20.8203 -43.9062

H -6.5169 20.9403 -44.2122

C -8.5929 20.9493 -44.8112

C -9.9149 20.7723 -44.3792

C -10.1729 20.5173 -43.0252

C -9.1109 20.3543 -42.1452

H -9.2899 20.1093 -41.1112

C -7.1719 21.2033 -46.7102

H -6.5949 22.0863 -46.3952

H -6.6339 20.3083 -46.3742

C -7.2889 21.1843 -48.2352

H -7.7339 22.1203 -48.5732

H -6.2719 21.1253 -48.6302

C -8.1209 20.0133 -48.7692

H -7.9819 19.1443 -48.1232

H -7.7619 19.7443 -49.7662

C -9.6079 20.3553 -48.8482

H -9.9399 20.7113 -47.8742

H -9.7479 21.1693 -49.5642

C -10.4649 19.1643 -49.2712

H -10.3579 18.3643 -48.5342

H -10.1129 18.7723 -50.2302

C -11.9369 19.5573 -49.3812

H -12.2299 20.0833 -48.4722

H -12.0609 20.2563 -50.2122

C -12.8649 18.3613 -49.5822

H -12.7219 17.6533 -48.7612

H -12.6079 17.8423 -50.5082

C -14.3279 18.7933 -49.6252

H -14.5679 19.3343 -48.7082

H -14.4719 19.4863 -50.4582

C -15.2899 17.6183 -49.7782

H -15.1379 16.9163 -48.9542

H -15.0739 17.0853 -50.7062

C -16.7439 18.0823 -49.7852

H -16.8929 18.7813 -50.6132

H -16.9529 18.6223 -48.8592

C -17.7329 16.9283 -49.9252

H -17.5359 16.3953 -50.8572

H -17.5829 16.2233 -49.1042

C -19.1749 17.4203 -49.9162

H -19.3449 18.1133 -50.7382

H -19.8669 16.5883 -50.0232

H -19.3959 17.9353 -48.9842

C -11.7309 19.6963 -45.4482

H -11.4559 19.2133 -46.3952

H -11.5309 18.9903 -44.6372

C -13.2099 20.0633 -45.4572

H -13.4449 20.5683 -44.5202

H -13.3919 20.7673 -46.2712

C -14.0919 18.8293 -45.6292

H -13.8939 18.1263 -44.8162

H -13.8339 18.3263 -46.5642

C -15.5759 19.1853 -45.6382

H -15.8309 19.6963 -44.7072

H -15.7729 19.8813 -46.4572

C -16.4659 17.9563 -45.8022

H -16.2589 17.2483 -44.9952

H -16.2239 17.4563 -46.7422

C -17.9489 18.3173 -45.7862

H -18.1859 18.8263 -44.8492

H -18.1609 19.0153 -46.5992

C -18.8439 17.0903 -45.9342

H -18.6219 16.3803 -45.1332

H -18.6249 16.5923 -46.8812

C -20.3249 17.4563 -45.8862

H -20.5439 17.9453 -44.9342

H -20.5469 18.1743 -46.6802

C -21.2279 16.2363 -46.0462

H -20.9889 15.5053 -45.2692

H -21.0299 15.7643 -47.0122

C -22.7069 16.6043 -45.9542

H -22.8979 17.0873 -44.9942

H -22.9489 17.3253 -46.7392

C -23.6169 15.3873 -46.0942

H -23.3639 14.6553 -45.3232

H -23.4429 14.9153 -47.0642

C -25.0879 15.7653 -45.9712

H -25.3629 16.4813 -46.7422

H -25.7209 14.8873 -46.0762

H -25.2849 16.2153 -45.0002

C -11.7979 20.3563 -41.2922

H -11.4999 19.3843 -40.8732

H -11.2629 21.1533 -40.7572

C -13.2989 20.5633 -41.1402

H -13.5059 20.7963 -40.0952

H -13.5819 21.4303 -41.7392

C -14.1299 19.3563 -41.5662

H -13.9039 19.1073 -42.6022

H -13.8669 18.4943 -40.9482

C -15.6189 19.6543 -41.4162

H -15.8859 20.4833 -42.0762

H -15.8159 19.9823 -40.3922

C -16.5099 18.4563 -41.7322

H -16.2739 17.6363 -41.0502

H -16.3129 18.1103 -42.7472

C -17.9859 18.8203 -41.5962

H -18.2269 19.6133 -42.3092

H -18.1669 19.2223 -40.5962

C -18.9149 17.6333 -41.8342

H -18.7349 17.2243 -42.8302

H -18.6909 16.8483 -41.1092

C -20.3809 18.0363 -41.7082

H -20.6109 18.7953 -42.4602

H -20.5479 18.4933 -40.7292

C -21.3319 16.8543 -41.8802

H -21.0959 16.0893 -41.1372

H -21.1839 16.4133 -42.8682

C -22.7909 17.2743 -41.7252

H -22.9409 17.7053 -40.7322

H -23.0239 18.0533 -42.4562

C -23.7549 16.1073 -41.9202

H -23.5449 15.3363 -41.1752

H -23.5929 15.6663 -42.9052

C -25.2079 16.5513 -41.7962

H -25.4349 17.3203 -42.5322

H -25.8819 15.7123 -41.9552

H -25.3979 16.9623 -40.8072

O -10.9279 20.8643 -45.3032

O -8.4579 21.2343 -46.1332

O -11.4829 20.4153 -42.6672

O -5.5419 19.7903 -42.2472

C 1.7351 19.7473 -39.4982

C 1.7391 19.2383 -38.2112

H 0.7981 18.9653 -37.7622

C 2.9501 19.0723 -37.5502

C 4.1641 19.3973 -38.1782

C 4.1471 19.9643 -39.4622

C 2.9231 20.1493 -40.0952

H 2.8821 20.5743 -41.0852

C 2.0041 18.4563 -35.4312

H 1.1511 17.9583 -35.9082

H 1.6911 19.4723 -35.1402

C 2.4051 17.6563 -34.1922

H 2.5951 16.6233 -34.4882

H 1.5431 17.6683 -33.5232

C 3.6391 18.2043 -33.4692

H 3.6181 19.2953 -33.5012

H 3.6011 17.9063 -32.4172

C 4.9441 17.7023 -34.0872

H 4.9371 17.9123 -35.1562

H 5.0071 16.6183 -33.9592

C 6.1721 18.3563 -33.4602

H 6.0991 19.4393 -33.5912

H 6.1911 18.1653 -32.3822

C 7.4711 17.8623 -34.0962

H 7.3781 17.9243 -35.1812

H 7.6291 16.8123 -33.8362

C 8.6681 18.6903 -33.6392

H 8.5071 19.7313 -33.9352

H 8.7271 18.6593 -32.5492

C 10.0041 18.2203 -34.2082

H 9.9631 18.2343 -35.2992

H 10.1931 17.1913 -33.8942

C 11.1411 19.1193 -33.7262

H 10.9471 20.1443 -34.0522

H 11.1551 19.1213 -32.6332

C 12.5101 18.6823 -34.2362

H 12.7021 17.6523 -33.9272

H 12.5161 18.7063 -35.3282

C 13.6221 19.5843 -33.7042

H 13.6281 19.5403 -32.6122

H 13.4131 20.6173 -33.9912

C 14.9921 19.1793 -34.2302

H 15.2301 18.1593 -33.9352

H 15.7641 19.8383 -33.8382

H 15.0151 19.2343 -35.3162

C 6.3381 20.0033 -37.3452

H 6.4371 20.1773 -36.2662

H 6.0881 20.9503 -37.8242

C 7.6651 19.4733 -37.8832

H 7.6081 19.3973 -38.9682

H 7.8371 18.4713 -37.4842

C 8.7911 20.4133 -37.4642

H 8.5571 21.4163 -37.8282

H 8.8221 20.4573 -36.3732

C 10.1651 19.9923 -37.9772

H 10.1701 19.9993 -39.0682

H 10.3831 18.9703 -37.6542

C 11.2451 20.9333 -37.4482

H 10.9971 21.9573 -37.7372

H 11.2441 20.8903 -36.3562

C 12.6461 20.6043 -37.9552

H 12.6721 20.6853 -39.0432

H 12.8981 19.5723 -37.6962

C 13.6791 21.5483 -37.3442

H 13.4061 22.5803 -37.5802

H 13.6551 21.4423 -36.2572

C 15.0991 21.2853 -37.8372

H 15.1431 21.4323 -38.9182

H 15.3711 20.2463 -37.6362

C 16.1021 22.2133 -37.1552

H 15.8081 23.2513 -37.3342

H 16.0681 22.0413 -36.0762

C 17.5301 22.0053 -37.6522

H 17.5611 22.1593 -38.7332

H 17.8371 20.9753 -37.4572

C 18.5101 22.9643 -36.9792

H 18.1731 23.9913 -37.1382

H 18.5121 22.7803 -35.9022

C 19.9251 22.8123 -37.5212

H 20.2911 21.8013 -37.3572

H 20.6001 23.5073 -37.0262

H 19.9471 23.0153 -38.5892

C 5.3901 20.7233 -41.3582

H 5.0231 21.7583 -41.4002

H 4.7531 20.0943 -41.9942

C 6.8251 20.6393 -41.8722

H 6.8051 20.8183 -42.9492

H 7.1861 19.6223 -41.7072

C 7.7741 21.6323 -41.2062

H 7.7881 21.4543 -40.1322

H 7.4091 22.6473 -41.3612

C 9.1851 21.5063 -41.7782

H 9.5371 20.4793 -41.6502

H 9.1571 21.7083 -42.8512

C 10.1731 22.4593 -41.1112

H 9.8151 23.4853 -41.2172

H 10.2211 22.2333 -40.0442

C 11.5741 22.3483 -41.7092

H 11.9231 21.3163 -41.6182

H 11.5361 22.5863 -42.7742

C 12.5701 23.2753 -41.0152

H 12.5731 23.0583 -39.9452

H 12.2481 24.3123 -41.1412

C 13.9881 23.1183 -41.5572

H 14.3061 22.0793 -41.4372

H 14.0001 23.3393 -42.6272

C 14.9751 24.0333 -40.8362

H 14.6761 25.0743 -40.9832

H 14.9361 23.8273 -39.7632

C 16.4091 23.8483 -41.3252

H 16.4551 24.0373 -42.4012

H 16.7171 22.8113 -41.1652

C 17.3811 24.7823 -40.6082

H 17.0381 25.8123 -40.7242

H 17.3811 24.5503 -39.5412

C 18.7991 24.6633 -41.1512

H 19.1631 23.6423 -41.0492

H 19.4731 25.3243 -40.6122

H 18.8281 24.9283 -42.2062

O 5.2951 19.0483 -37.4982

O 3.1021 18.5353 -36.3062

O 5.3511 20.2573 -40.0242

O -0.6919 19.6403 -39.5802

C -2.9219 19.6443 -35.5002

C -1.6939 19.5333 -34.8362

C -4.0989 19.7573 -34.7562

C -1.6489 19.5223 -33.4632

H -0.7909 19.4573 -35.4212

C -4.0529 19.7383 -33.3822

H -5.0429 19.8653 -35.2702

C -2.8289 19.6133 -32.7252

H -0.7069 19.4373 -32.9432

H -4.9579 19.8243 -32.8012

C -3.2509 18.9733 -46.2582

C -2.0299 19.0753 -46.9332

C -4.4219 18.7353 -46.9832

C -1.9969 18.9983 -48.3052

H -1.1269 19.2253 -46.3622

C -4.3859 18.6653 -48.3572

H -5.3489 18.5993 -46.4472

C -3.1729 18.8133 -49.0302

H -1.0719 19.0943 -48.8502

H -5.2889 18.4963 -48.9232

C -6.6879 20.2893 -41.5932

H -7.0229 19.5863 -40.8172

H -6.4459 21.2493 -41.1092

C 0.4911 19.7633 -40.3462

H 0.5661 18.9123 -41.0412

H 0.4481 20.6903 -40.9342

C -2.3179 19.5333 -8.5662

C -3.0879 19.9363 -14.1512

C -3.1369 19.9983 -15.3582

C -3.1879 20.0563 -16.7602

C -1.9799 20.0833 -17.4922

C -4.4219 20.0903 -17.4162

H -5.3069 20.0883 -16.8002

O -3.2559 19.5133 -7.7712

O -1.0659 19.4203 -8.1922

H -0.9619 19.3273 -7.1962

C -2.9569 19.5973 -27.7692

C -3.1739 19.9033 -22.1292

C -3.2969 19.9983 -20.9302

C -3.2979 20.0663 -19.5282

C -4.5039 20.1173 -18.7992

C -2.0649 20.0723 -18.8742

H -1.1819 20.0533 -19.4922

O -1.9059 19.5493 -28.3912

O -4.1349 19.5883 -28.3512

H -4.0469 19.5563 -29.3562

C -8.0469 20.5143 -19.7422

C -7.9629 20.3913 -21.1172

H -6.9999 20.2003 -21.5592

C -9.1179 20.4953 -21.8942

C -10.3549 20.7243 -21.2802

C -10.4269 20.8693 -19.8882

C -9.2699 20.7613 -19.1312

H -9.3039 20.8633 -18.0592

C -8.0279 19.9653 -23.9502

H -7.2219 20.7023 -23.8182

H -7.6839 18.9943 -23.5652

C -8.3559 19.8503 -25.4392

H -8.6339 20.8343 -25.8162

H -7.4369 19.5383 -25.9402

C -9.4779 18.8563 -25.7532

H -9.4369 18.0263 -25.0442

H -9.3169 18.4423 -26.7522

C -10.8629 19.4993 -25.7052

H -11.0079 19.9703 -24.7332

H -10.9179 20.2843 -26.4642

C -11.9699 18.4783 -25.9572

H -11.9449 17.7193 -25.1702

H -11.7889 17.9683 -26.9072

C -13.3559 19.1203 -25.9872

H -13.5049 19.6933 -25.0702

H -13.4129 19.8213 -26.8242

C -14.4619 18.0773 -26.1202

H -14.3929 17.3753 -25.2852

H -14.3119 17.5053 -27.0392

C -15.8569 18.6983 -26.1352

H -15.9979 19.2953 -25.2332

H -15.9449 19.3713 -26.9922

C -16.9479 17.6333 -26.2152

H -16.8489 16.9533 -25.3642

H -16.8069 17.0413 -27.1232

C -18.3519 18.2313 -26.2182

H -18.4569 18.9003 -27.0762

H -18.4929 18.8293 -25.3152

C -19.4309 17.1543 -26.2872

H -19.2889 16.5613 -27.1932

H -19.3239 16.4803 -25.4332

C -20.8329 17.7503 -26.2892

H -20.9679 18.4023 -27.1492

H -21.5849 16.9653 -26.3352

H -21.0009 18.3353 -25.3872

C -12.3979 19.7313 -21.9342

H -12.2049 19.0123 -22.7432

H -12.2479 19.2163 -20.9792

C -13.8279 20.2523 -22.0232

H -14.0069 20.9203 -21.1802

H -13.9249 20.8383 -22.9382

C -14.8509 19.1193 -22.0272

H -14.7149 18.5033 -21.1342

H -14.6789 18.4793 -22.8952

C -16.2819 19.6493 -22.0642

H -16.4649 20.2623 -21.1802

H -16.4019 20.2923 -22.9392

C -17.3159 18.5273 -22.1272

H -17.1879 17.8663 -21.2662

H -17.1469 17.9313 -23.0262

C -18.7449 19.0673 -22.1412

H -18.9229 19.6453 -21.2322

H -18.8649 19.7443 -22.9902

C -19.7819 17.9523 -22.2452

H -19.6759 17.2763 -21.3932

H -19.5969 17.3713 -23.1502

C -21.2069 18.4983 -22.2802

H -21.4049 19.0543 -21.3622

H -21.3039 19.1993 -23.1142

C -22.2449 17.3893 -22.4332

H -22.1629 16.7013 -21.5892

H -22.0359 16.8213 -23.3432

C -23.6669 17.9403 -22.4992

H -23.8739 18.5143 -21.5932

H -23.7499 18.6233 -23.3492

C -24.7109 16.8363 -22.6402

H -24.6399 16.1613 -21.7852

H -24.4999 16.2533 -23.5402

C -26.1239 17.4003 -22.7222

H -26.2219 18.0623 -23.5792

H -26.8519 16.5983 -22.8232

H -26.3619 17.9663 -21.8242

C -11.8159 21.2283 -17.9822

H -11.4689 20.3193 -17.4692

H -11.2249 22.0833 -17.6242

C -13.2939 21.4623 -17.6992

H -13.4079 21.7573 -16.6562

H -13.6299 22.2933 -18.3212

C -14.1479 20.2323 -17.9932

H -13.9509 19.8993 -19.0132

H -13.8699 19.4203 -17.3182

C -15.6339 20.5413 -17.8372

H -15.9079 21.3293 -18.5422

H -15.8199 20.9283 -16.8322

C -16.5209 19.3243 -18.0812

H -16.3179 18.5643 -17.3242

H -16.2839 18.8913 -19.0552

C -17.9989 19.7003 -18.0432

H -18.2079 20.4053 -18.8512

H -18.2189 20.2113 -17.1022

C -18.9199 18.4923 -18.1852

H -18.6789 17.9583 -19.1062

H -18.7509 17.8073 -17.3512

C -20.3879 18.9083 -18.2112

H -20.5559 19.5623 -19.0712

H -20.6159 19.4863 -17.3122

C -21.3369 17.7173 -18.2962

H -21.1909 17.0733 -17.4262

H -21.0999 17.1303 -19.1872

C -22.7949 18.1633 -18.3592

H -23.0369 18.7373 -17.4612

H -22.9299 18.8223 -19.2202

C -23.7609 16.9883 -18.4812

H -23.6359 16.3253 -17.6222

H -23.5189 16.4153 -19.3792

C -25.2089 17.4553 -18.5562

H -25.3509 18.1143 -19.4102

H -25.8829 16.6093 -18.6612

H -25.4809 18.0023 -17.6552

O -11.4849 20.8173 -22.0622

O -9.1749 20.3803 -23.2462

O -11.6669 21.0863 -19.3782

O -5.6499 20.1803 -19.5252

C 1.6081 20.3053 -16.6802

C 1.6471 19.7723 -15.4032

H 0.7301 19.4133 -14.9652

C 2.8641 19.6993 -14.7332

C 4.0501 20.1413 -15.3442

C 3.9961 20.7143 -16.6252

C 2.7631 20.8103 -17.2622

H 2.6941 21.2493 -18.2442

C 1.9531 18.9483 -12.6502

H 1.1951 18.3273 -13.1442

H 1.4921 19.9133 -12.3882

C 2.4181 18.2303 -11.3842

H 2.7821 17.2383 -11.6562

H 1.5331 18.1083 -10.7562

C 3.5121 18.9723 -10.6122

H 3.3271 20.0473 -10.6702

H 3.4661 18.6873 -9.5582

C 4.9091 18.6653 -11.1482

H 4.9451 18.8923 -12.2142

H 5.1091 17.5973 -11.0292

C 5.9891 19.4623 -10.4222

H 5.7861 20.5283 -10.5482

H 5.9471 19.2513 -9.3492

C 7.3861 19.1513 -10.9582

H 7.3741 19.2343 -12.0462

H 7.6531 18.1213 -10.7082

C 8.4331 20.1053 -10.3892

H 8.1761 21.1263 -10.6842

H 8.4011 20.0613 -9.2972

C 9.8521 19.7963 -10.8632

H 9.8751 19.7813 -11.9542

H 10.1461 18.8033 -10.5122

C 10.8451 20.8343 -10.3462

H 10.5741 21.8163 -10.7422

H 10.7721 20.8853 -9.2562

C 12.2931 20.5263 -10.7182

H 12.5771 19.5583 -10.2972

H 12.3861 20.4533 -11.8032

C 13.2441 21.6013 -10.1962

H 13.1041 21.7113 -9.1172

H 12.9911 22.5603 -10.6572

C 14.7021 21.2673 -10.4832

H 14.9831 20.3363 -9.9952

H 15.3561 22.0563 -10.1162

H 14.8651 21.1543 -11.5522

C 6.1601 20.9243 -14.4852

H 6.1741 21.1733 -13.4152

H 5.8861 21.8233 -15.0392

C 7.5441 20.4393 -14.9042

H 7.5631 20.2993 -15.9852

H 7.7341 19.4693 -14.4382

C 8.6001 21.4513 -14.4752

H 8.3601 22.4133 -14.9302

H 8.5481 21.5783 -13.3902

C 10.0211 21.0423 -14.8572

H 10.1001 20.9603 -15.9432

H 10.2451 20.0593 -14.4372

C 11.0361 22.0603 -14.3422

H 10.7941 23.0433 -14.7522

H 10.9501 22.1283 -13.2552

C 12.4771 21.7133 -14.7072

H 12.5801 21.6773 -15.7942

H 12.7241 20.7213 -14.3212

C 13.4521 22.7393 -14.1342

H 13.1981 23.7293 -14.5212

H 13.3361 22.7733 -13.0492

C 14.9071 22.4293 -14.4712

H 15.0381 22.4353 -15.5552

H 15.1591 21.4273 -14.1152

C 15.8571 23.4453 -13.8392

H 15.5961 24.4463 -14.1922

H 15.7251 23.4343 -12.7552

C 17.3191 23.1633 -14.1712

H 17.4531 23.1773 -15.2552

H 17.5851 22.1633 -13.8192

C 18.2551 24.1873 -13.5342

H 17.9751 25.1883 -13.8692

H 18.1371 24.1573 -12.4492

C 19.7141 23.9313 -13.8922

H 20.0241 22.9463 -13.5502

H 20.3581 24.6743 -13.4282

H 19.8561 23.9803 -14.9692

C 5.1931 21.5863 -18.5062

H 4.7751 22.6033 -18.5362

H 4.5901 20.9343 -19.1512

C 6.6331 21.5783 -19.0132

H 6.6161 21.7953 -20.0842

H 7.0331 20.5713 -18.8802

C 7.5341 22.5793 -18.2962

H 7.5111 22.3793 -17.2262

H 7.1531 23.5903 -18.4462

C 8.9701 22.4943 -18.8112

H 9.3331 21.4693 -18.6962

H 8.9861 22.7283 -19.8792

C 9.9101 23.4413 -18.0702

H 9.5501 24.4663 -18.1762

H 9.8961 23.1943 -17.0072

C 11.3441 23.3523 -18.5862

H 11.7001 22.3243 -18.4772

H 11.3651 23.5943 -19.6512

C 12.2861 24.2883 -17.8342

H 12.2181 24.0783 -16.7642

H 11.9701 25.3233 -17.9892

C 13.7391 24.1333 -18.2772

H 14.0581 23.1013 -18.1072

H 13.8181 24.3263 -19.3502

C 14.6691 25.0763 -17.5182

H 14.3741 26.1103 -17.7152

H 14.5581 24.9003 -16.4462

C 16.1341 24.8873 -17.9032

H 16.2521 25.0373 -18.9792

H 16.4371 23.8613 -17.6822

C 17.0471 25.8563 -17.1562

H 16.7151 26.8793 -17.3452

H 16.9611 25.6713 -16.0832

C 18.5041 25.7143 -17.5762

H 18.8581 24.7023 -17.3912

H 19.1331 26.4053 -17.0182

H 18.6181 25.9263 -18.6372

O 5.1981 19.8893 -14.6512

O 3.0551 19.1663 -13.4942

O 5.1771 21.1043 -17.1772

O -0.8229 20.1113 -16.7852

C -2.9249 19.8583 -12.7582

C -1.6289 19.7553 -12.2402

C -4.0139 19.8833 -11.8832

C -1.4279 19.6603 -10.8862

H -0.7999 19.7543 -12.9292

C -3.8109 19.7933 -10.5252

H -5.0129 19.9743 -12.2792

C -2.5189 19.6683 -10.0152

H -0.4319 19.5793 -10.4812

H -4.6489 19.8153 -9.8462

C -3.1099 19.8133 -23.5282

C -1.8809 19.7093 -24.1842

C -4.2919 19.8323 -24.2742

C -1.8409 19.6423 -25.5582

H -0.9729 19.6823 -23.6022

C -4.2489 19.7633 -25.6462

H -5.2329 19.8993 -23.7532

C -3.0209 19.6693 -26.3002

H -0.9069 19.5723 -26.0902

H -5.1559 19.7833 -26.2282

C -6.8569 20.3643 -18.8232

H -7.0409 19.5003 -18.1642

H -6.7849 21.2653 -18.1932

C 0.3701 20.2543 -17.5372

H 0.4741 19.3923 -18.2152

H 0.3111 21.1683 -18.1422

C -2.2109 16.8303 -21.0522

C -2.1479 16.4133 -26.6962

C -2.1779 16.4073 -27.9042

C -2.2549 16.4313 -29.3072

C -1.0829 16.4443 -30.0902

C -3.5059 16.4963 -29.9202

H -4.3699 16.5103 -29.2752

O -3.2509 16.7213 -20.4172

O -1.0539 17.0983 -20.4932

H -1.1259 17.1483 -19.4902

C -2.3549 16.4503 -40.3632

C -2.5449 16.4203 -34.6972

C -2.5379 16.4483 -33.4882

C -2.4659 16.4713 -32.0832

C -3.6319 16.5703 -31.2982

C -1.2119 16.4233 -31.4682

H -0.3449 16.3733 -32.1082

O -1.2949 16.5733 -40.9642

O -3.5079 16.2903 -40.9662

H -3.4059 16.2123 -41.9662

C -7.0779 17.6263 -31.9522

C -7.0849 17.6603 -33.3332

H -6.1949 17.3603 -33.8602

C -8.2429 18.0483 -34.0042

C -9.3969 18.3743 -33.2782

C -9.3589 18.3963 -31.8752

C -8.1909 18.0283 -31.2272

H -8.1329 18.0243 -30.1512

C -7.3189 17.6433 -36.1712

H -6.3899 18.1953 -35.9672

H -7.1449 16.5793 -35.9582

C -7.6899 17.8263 -37.6422

H -7.9119 18.8813 -37.8142

H -6.8059 17.5603 -38.2262

C -8.8789 16.9743 -38.0952

H -8.7979 15.9803 -37.6512

H -8.8309 16.8513 -39.1802

C -10.2279 17.5903 -37.7252

H -10.2389 17.8193 -36.6602

H -10.3509 18.5333 -38.2632

C -11.3969 16.6663 -38.0592

H -11.2869 15.7303 -37.5052

H -11.3749 16.4173 -39.1242

C -12.7409 17.3013 -37.7122

H -12.7319 17.6073 -36.6652

H -12.8819 18.2033 -38.3112

C -13.9109 16.3473 -37.9462

H -13.7729 15.4523 -37.3342

H -13.9169 16.0283 -38.9922

C -15.2549 16.9873 -37.6062

H -15.2379 17.3353 -36.5722

H -15.4109 17.8613 -38.2422

C -16.4179 16.0143 -37.7892

H -16.2849 15.1623 -37.1162

H -16.4059 15.6243 -38.8112

C -17.7689 16.6693 -37.5162

H -17.9119 17.5013 -38.2072

H -17.7749 17.0783 -36.5042

C -18.9279 15.6863 -37.6662

H -18.8949 15.2373 -38.6622

H -18.8119 14.8763 -36.9412

C -20.2789 16.3613 -37.4612

H -20.4219 17.1533 -38.1932

H -21.0879 15.6433 -37.5702

H -20.3399 16.7993 -36.4682

C -11.5589 17.6283 -33.8022

H -11.5469 16.9943 -34.7012

H -11.3299 16.9933 -32.9402

C -12.9299 18.2663 -33.6242

H -12.9229 18.8513 -32.7052

H -13.0999 18.9533 -34.4552

C -14.0419 17.2223 -33.5812

H -13.8729 16.5403 -32.7442

H -14.0159 16.6263 -34.4972

C -15.4149 17.8743 -33.4402

H -15.4559 18.4313 -32.5022

H -15.5529 18.5913 -34.2532

C -16.5519 16.8563 -33.4762

H -16.4359 16.1473 -32.6532

H -16.4989 16.2873 -34.4082

C -17.9159 17.5343 -33.3752

H -17.9779 18.0803 -32.4322

H -18.0119 18.2653 -34.1822

C -19.0699 16.5393 -33.4612

H -18.9909 15.8203 -32.6422

H -18.9979 15.9793 -34.3962

C -20.4269 17.2373 -33.3902

H -20.4989 17.7883 -32.4512

H -20.5019 17.9643 -34.2032

C -21.5919 16.2563 -33.4902

H -21.5349 15.5423 -32.6652

H -21.5099 15.6883 -34.4202

C -22.9399 16.9703 -33.4512

H -23.0169 17.5393 -32.5222

H -22.9949 17.6793 -34.2812

C -24.1139 15.9993 -33.5422

H -24.0649 15.2933 -32.7102

H -24.0349 15.4243 -34.4682

C -25.4549 16.7233 -33.5092

H -25.5309 17.4243 -34.3382

H -26.2749 16.0133 -33.5842

H -25.5669 17.2773 -32.5802

C -10.4969 18.8073 -29.8262

H -10.2169 17.8323 -29.4022

H -9.7659 19.5583 -29.4982

C -11.8939 19.1933 -29.3542

H -11.8429 19.4633 -28.2982

H -12.2079 20.0733 -29.9152

C -12.9099 18.0703 -29.5522

H -12.8269 17.6923 -30.5722

H -12.6839 17.2473 -28.8702

C -14.3329 18.5643 -29.3082

H -14.5599 19.3493 -30.0342

H -14.3959 19.0103 -28.3142

C -15.3799 17.4613 -29.4332

H -15.2339 16.7243 -28.6402

H -15.2539 16.9463 -30.3882

C -16.7929 18.0323 -29.3492

H -16.9349 18.7423 -30.1672

H -16.9019 18.5843 -28.4132

C -17.8779 16.9623 -29.4292

H -17.7559 16.3893 -30.3512

H -17.7689 16.2683 -28.5922

C -19.2719 17.5833 -29.3982

H -19.3769 18.2643 -30.2472

H -19.3799 18.1743 -28.4862

C -20.3849 16.5413 -29.4552

H -20.2899 15.8593 -28.6072

H -20.2809 15.9483 -30.3672

C -21.7659 17.1903 -29.4262

H -21.8579 17.7953 -28.5222

H -21.8659 17.8593 -30.2852

C -22.8949 16.1653 -29.4572

H -22.7939 15.4913 -28.6032

H -22.8129 15.5633 -30.3652

C -24.2639 16.8323 -29.4152

H -24.3909 17.4953 -30.2692

H -25.0569 16.0883 -29.4402

H -24.3739 17.4213 -28.5072

O -10.5669 18.6403 -33.9532

O -8.3659 18.1273 -35.3562

O -10.5089 18.7413 -31.2392

O -4.8109 16.7543 -31.9512

C 2.3611 17.1023 -29.1302

C 2.1231 17.9033 -28.0242

H 1.1481 18.3453 -27.9082

C 3.1291 18.1013 -27.0822

C 4.3871 17.5153 -27.2822

C 4.6311 16.7463 -28.4272

C 3.6041 16.5283 -29.3392

H 3.7621 15.9053 -30.2052

C 1.7721 19.4343 -25.6432

H 0.9501 18.7233 -25.8052

H 1.6171 20.3033 -26.3002

C 1.7741 19.8693 -24.1782

H 1.8651 18.9823 -23.5492

H 0.8031 20.3263 -23.9812

C 2.8921 20.8563 -23.8312

H 3.1271 21.4583 -24.7082

H 2.5401 21.5303 -23.0452

C 4.1661 20.1673 -23.3482

H 4.4861 19.4413 -24.0952

H 3.9521 19.6253 -22.4222

C 5.2811 21.1793 -23.0992

H 5.5561 21.6463 -24.0472

H 4.9131 21.9693 -22.4372

C 6.5181 20.5413 -22.4712

H 6.8211 19.6823 -23.0712

H 6.2771 20.1763 -21.4692

C 7.6741 21.5333 -22.3962

H 7.9121 21.8563 -23.4112

H 7.3581 22.4173 -21.8342

C 8.9311 20.9513 -21.7552

H 9.1981 20.0233 -22.2642

H 8.7331 20.7083 -20.7082

C 10.1021 21.9273 -21.8422

H 10.3041 22.1463 -22.8932

H 9.8251 22.8673 -21.3572

C 11.3731 21.3843 -21.1942

H 11.1931 21.2113 -20.1312

H 11.6261 20.4223 -21.6472

C 12.5501 22.3423 -21.3592

H 12.2901 23.3093 -20.9212

H 12.7371 22.5013 -22.4242

C 13.8161 21.8153 -20.6982

H 13.6611 21.6823 -19.6302

H 14.6431 22.5083 -20.8412

H 14.0981 20.8543 -21.1242

C 6.2771 18.7383 -26.6142

H 5.8471 19.6893 -26.2712

H 6.4501 18.8133 -27.6942

C 7.5941 18.4633 -25.8992

H 8.0241 17.5413 -26.2972

H 7.3881 18.2983 -24.8412

C 8.5621 19.6263 -26.0772

H 8.6811 19.8333 -27.1432

H 8.1281 20.5173 -25.6192

C 9.9321 19.3453 -25.4662

H 10.3571 18.4473 -25.9222

H 9.8221 19.1473 -24.3982

C 10.8891 20.5173 -25.6682

H 10.9661 20.7413 -26.7352

H 10.4761 21.4003 -25.1762

C 12.2831 20.2393 -25.1122

H 12.7101 19.3673 -25.6152

H 12.2071 19.9993 -24.0502

C 13.2131 21.4353 -25.2922

H 13.2651 21.6993 -26.3512

H 12.7941 22.2953 -24.7632

C 14.6211 21.1613 -24.7702

H 15.0671 20.3433 -25.3422

H 14.5651 20.8393 -23.7282

C 15.5141 22.3953 -24.8662

H 15.5141 22.7603 -25.8972

H 15.1011 23.1883 -24.2392

C 16.9501 22.1083 -24.4352

H 17.3671 21.3183 -25.0642

H 16.9531 21.7443 -23.4052

C 17.8331 23.3493 -24.5352

H 17.7801 23.7463 -25.5522

H 17.4501 24.1183 -23.8612

C 19.2861 23.0493 -24.1902

H 19.3651 22.6583 -23.1782

H 19.8901 23.9523 -24.2562

H 19.6961 22.3103 -24.8752

C 6.2391 15.5353 -29.7122

H 6.0081 16.1263 -30.6102

H 5.6781 14.5913 -29.7592

C 7.7381 15.2593 -29.6472

H 8.0291 14.6883 -30.5312

H 7.9461 14.6563 -28.7622

C 8.5271 16.5633 -29.5712

H 8.2121 17.1033 -28.6782

H 8.2841 17.1793 -30.4402

C 10.0341 16.3353 -29.5202

H 10.2701 15.6403 -28.7112

H 10.3671 15.8773 -30.4552

C 10.7881 17.6433 -29.2962

H 10.5111 18.3563 -30.0762

H 10.4861 18.0703 -28.3382

C 12.3011 17.4473 -29.3072

H 12.5751 16.7113 -28.5472

H 12.6091 17.0493 -30.2772

C 13.0471 18.7513 -29.0372

H 12.7221 19.1573 -28.0762

H 12.7861 19.4823 -29.8062

C 14.5601 18.5563 -29.0132

H 14.8171 17.8363 -28.2312

H 14.8891 18.1363 -29.9662

C 15.2991 19.8663 -28.7542

H 15.0901 20.5653 -29.5672

H 14.9201 20.3123 -27.8312

C 16.8071 19.6713 -28.6292

H 17.1931 19.2173 -29.5452

H 17.0151 18.9803 -27.8072

C 17.5301 20.9913 -28.3722

H 17.3521 21.6673 -29.2102

H 17.1111 21.4563 -27.4772

C 19.0291 20.8033 -28.1832

H 19.2301 20.1523 -27.3342

H 19.5151 21.7603 -28.0042

H 19.4741 20.3533 -29.0682

O 5.3751 17.6723 -26.3392

O 3.0071 18.8233 -25.9452

O 5.8901 16.2573 -28.5502

O 0.0901 16.4733 -29.4142

C -2.1449 16.4643 -25.2902

C -0.9409 16.5073 -24.5802

C -3.3599 16.4973 -24.5952

C -0.9519 16.6183 -23.2072

H -0.0079 16.4563 -25.1212

C -3.3659 16.5973 -23.2242

H -4.2859 16.4523 -25.1482

C -2.1639 16.6763 -22.5192

H -0.0279 16.6613 -22.6522

H -4.2919 16.6303 -22.6702

C -2.5159 16.4243 -36.1042

C -1.2889 16.4903 -36.7732

C -3.6979 16.3673 -36.8492

C -1.2449 16.5163 -38.1482

H -0.3759 16.5083 -36.1962

C -3.6529 16.3803 -38.2252

H -4.6439 16.3073 -36.3322

C -2.4269 16.4573 -38.8872

H -0.3029 16.5713 -38.6722

H -4.5599 16.3273 -38.8072

C -5.9029 17.1433 -31.1462

H -6.2359 16.2903 -30.5342

H -5.5909 17.9513 -30.4702

C 1.2521 16.8593 -30.1202

H 1.5501 16.0773 -30.8312

H 1.0421 17.7843 -30.6792

C -2.1829 16.1183 -44.0502

C -2.0349 15.6923 -49.6942

C -2.0739 15.6983 -50.9032

C -2.1379 15.7313 -52.3072

C -0.9559 15.8553 -53.0682

C -3.3799 15.6923 -52.9422

H -4.2559 15.6243 -52.3162

O -3.2269 15.9513 -43.4352

O -1.0579 16.4653 -43.4692

H -1.1589 16.5233 -42.4702

C -2.9579 15.9733 -63.3132

C -2.3799 15.6633 -57.6952

C -2.3599 15.7153 -56.4872

C -2.2979 15.7583 -55.0832

C -3.4859 15.7633 -54.3222

C -1.0559 15.8233 -54.4482

H -0.1759 15.8503 -55.0712

O -2.0199 16.2073 -64.0652

O -4.2009 15.8633 -63.7302

H -4.2889 15.9893 -64.7222

C -6.9249 16.7373 -55.1002

C -6.9219 16.6683 -56.4822

H -6.0569 16.2593 -56.9782

C -8.0349 17.1103 -57.1962

C -9.1579 17.5913 -56.5112

C -9.1289 17.7093 -55.1142

C -8.0029 17.2893 -54.4212

H -7.9569 17.3663 -53.3472

C -7.1259 16.5513 -59.3402

H -6.1719 17.0603 -59.1402

H -7.0089 15.4843 -59.1032

C -7.4859 16.7243 -60.8152

H -7.6739 17.7823 -61.0032

H -6.6099 16.4243 -61.3922

C -8.7009 15.9073 -61.2632

H -8.6779 14.9303 -60.7762

H -8.6349 15.7383 -62.3412

C -10.0269 16.6013 -60.9542

H -10.0459 16.8903 -59.9042

H -10.0939 17.5153 -61.5482

C -11.2289 15.7113 -61.2632

H -11.1869 14.8163 -60.6372

H -11.1819 15.3843 -62.3052

C -12.5519 16.4353 -61.0172

H -12.5609 16.8293 -60.0012

H -12.6269 17.2883 -61.6952

C -13.7599 15.5223 -61.2172

H -13.7229 14.7073 -60.4892

H -13.7129 15.0703 -62.2122

C -15.0809 16.2753 -61.0692

H -15.1109 16.7663 -60.0952

H -15.1349 17.0573 -61.8292

C -16.2919 15.3563 -61.2112

H -16.3049 14.6373 -60.3872

H -16.2039 14.7813 -62.1382

C -17.6029 16.1373 -61.2292

H -17.5899 16.8353 -62.0692

H -17.6869 16.7263 -60.3132

C -18.8219 15.2273 -61.3572

H -18.7089 14.5943 -62.2402

H -18.8769 14.5663 -60.4892

C -20.1149 16.0253 -61.4722

H -20.0889 16.6633 -62.3532

H -20.9719 15.3603 -61.5582

H -20.2539 16.6553 -60.5972

C -11.3349 16.9573 -57.0832

H -11.2509 16.2333 -57.9072

H -11.2279 16.4073 -56.1412

C -12.6909 17.6493 -57.1232

H -12.7619 18.3243 -56.2702

H -12.7449 18.2543 -58.0292

C -13.8389 16.6453 -57.0992

H -13.7639 16.0253 -56.2022

H -13.7589 15.9793 -57.9622

C -15.1969 17.3423 -57.1242

H -15.2979 17.9713 -56.2382

H -15.2449 17.9983 -57.9962

C -16.3559 16.3523 -57.1812

H -16.3159 15.6943 -56.3102

H -16.2529 15.7253 -58.0692

C -17.7069 17.0613 -57.2202

H -17.8239 17.6683 -56.3202

H -17.7289 17.7403 -58.0762

C -18.8709 16.0813 -57.3282

H -18.8559 15.4053 -56.4692

H -18.7469 15.4723 -58.2262

C -20.2189 16.7933 -57.3842

H -20.3479 17.3943 -56.4812

H -20.2319 17.4773 -58.2372

C -21.3829 15.8143 -57.5112

H -21.3769 15.1363 -56.6542

H -21.2499 15.2093 -58.4102

C -22.7289 16.5313 -57.5782

H -22.8489 17.1543 -56.6892

H -22.7429 17.1903 -58.4492

C -23.8989 15.5543 -57.6672

H -23.8899 14.9003 -56.7922

H -23.7759 14.9253 -58.5522

C -25.2389 16.2763 -57.7422

H -25.2759 16.9243 -58.6152

H -26.0559 15.5613 -57.8122

H -25.3949 16.8863 -56.8552

C -10.3119 18.3323 -53.1352

H -10.0859 17.3703 -52.6522

H -9.5789 19.0793 -52.8012

C -11.7279 18.7713 -52.7772

H -11.7569 19.0493 -51.7232

H -11.9669 19.6553 -53.3702

C -12.7499 17.6723 -53.0602

H -12.5789 17.2823 -54.0642

H -12.6069 16.8503 -52.3532

C -14.1809 18.1913 -52.9592

H -14.3269 18.9703 -53.7112

H -14.3329 18.6503 -51.9802

C -15.2229 17.0963 -53.1682

H -15.1339 16.3503 -52.3742

H -15.0339 16.5893 -54.1162

C -16.6369 17.6703 -53.1722

H -16.7309 18.3763 -54.0012

H -16.8009 18.2263 -52.2482

C -17.7159 16.5993 -53.3092

H -17.5519 16.0313 -54.2272

H -17.6419 15.8993 -52.4732

C -19.1119 17.2143 -53.3352

H -19.1979 17.8683 -54.2062

H -19.2489 17.8323 -52.4452

C -20.2169 16.1623 -53.3902

H -20.1489 15.5203 -52.5092

H -20.0729 15.5323 -54.2712

C -21.6029 16.7983 -53.4472

H -21.7489 17.4253 -52.5642

H -21.6649 17.4453 -54.3262

C -22.7179 15.7583 -53.5182

H -22.6679 15.1153 -52.6362

H -22.5649 15.1273 -54.3962

C -24.0949 16.4063 -53.5962

H -24.1689 17.0353 -54.4802

H -24.8739 15.6483 -53.6512

H -24.2769 17.0243 -52.7192

O -10.2959 17.9233 -57.2112

O -8.1539 17.1063 -58.5502

O -10.2549 18.2013 -54.5402

O -4.6579 15.8593 -55.0062

C 2.3921 16.8073 -52.0282

C 2.0541 17.5643 -50.9182

H 1.0461 17.9353 -50.8322

C 3.0011 17.8043 -49.9292

C 4.3071 17.3093 -50.0782

C 4.6531 16.5903 -51.2312

C 3.6821 16.3313 -52.1932

H 3.9191 15.7453 -53.0662

C 1.5091 19.0333 -48.5232

H 0.7171 18.3113 -48.7642

H 1.3661 19.9273 -49.1482

C 1.4181 19.3973 -47.0422

H 1.4731 18.4813 -46.4502

H 0.4381 19.8473 -46.8822

C 2.5111 20.3643 -46.5822

H 2.7471 21.0483 -47.3962

H 2.1331 20.9543 -45.7422

C 3.7921 19.6533 -46.1522

H 4.1121 18.9833 -46.9482

H 3.5921 19.0513 -45.2622

C 4.9071 20.6523 -45.8582

H 5.1001 21.2383 -46.7592

H 4.5871 21.3433 -45.0722

C 6.1981 19.9613 -45.4292

H 6.4461 19.1963 -46.1672

H 6.0541 19.4603 -44.4682

C 7.3521 20.9543 -45.3332

H 7.4351 21.4683 -46.2922

H 7.1251 21.7113 -44.5772

C 8.6871 20.2913 -45.0052

H 8.8561 19.4643 -45.6982

H 8.6561 19.8763 -43.9952

C 9.8431 21.2823 -45.1192

H 9.8811 21.6593 -46.1442

H 9.6551 22.1383 -44.4662

C 11.1921 20.6633 -44.7652

H 11.1781 20.3323 -43.7242

H 11.3611 19.7843 -45.3912

C 12.3381 21.6513 -44.9682

H 12.1571 22.5433 -44.3632

H 12.3571 21.9633 -46.0152

C 13.6881 21.0533 -44.5942

H 13.6971 20.7593 -43.5472

H 14.4861 21.7763 -44.7532

H 13.8961 20.1753 -45.2012

C 6.2591 18.4113 -49.3162

H 6.0081 19.3773 -48.8562

H 6.3641 18.5683 -50.3922

C 7.5721 17.8803 -48.7482

H 7.8321 16.9563 -49.2672

H 7.4341 17.6393 -47.6932

C 8.6721 18.9233 -48.9182

H 8.6981 19.2403 -49.9642

H 8.4191 19.8003 -48.3202

C 10.0581 18.4223 -48.5192

H 10.3141 17.5413 -49.1122

H 10.0541 18.1193 -47.4702

C 11.1091 19.5103 -48.7342

H 11.0541 19.8563 -49.7702

H 10.8751 20.3623 -48.0932

C 12.5331 19.0413 -48.4462

H 12.7791 18.1983 -49.0962

H 12.6011 18.6913 -47.4142

C 13.5401 20.1673 -48.6672

H 13.4371 20.5473 -49.6882

H 13.3091 20.9923 -47.9892

C 14.9841 19.7263 -48.4452

H 15.2381 18.9383 -49.1572

H 15.0861 19.3093 -47.4402

C 15.9571 20.8913 -48.6062

H 15.8411 21.3213 -49.6052

H 15.7061 21.6713 -47.8832

C 17.4111 20.4713 -48.4102

H 17.6711 19.7093 -49.1482

H 17.5271 20.0263 -47.4192

C 18.3701 21.6523 -48.5452

H 18.2581 22.0943 -49.5392

H 18.1041 22.4163 -47.8112

C 19.8221 21.2393 -48.3432

H 19.9621 20.8063 -47.3552

H 20.4811 22.0993 -48.4372

H 20.1151 20.5013 -49.0862

C 6.3181 15.3313 -52.3852

H 6.1671 15.8433 -53.3462

H 5.7081 14.4183 -52.3732

C 7.7931 14.9803 -52.2182

H 8.0621 14.2453 -52.9792

H 7.9351 14.5253 -51.2362

C 8.6751 16.2173 -52.3472

H 8.4021 16.9233 -51.5622

H 8.4791 16.6943 -53.3102

C 10.1611 15.8913 -52.2452

H 10.3581 15.3663 -51.3082

H 10.4421 15.2223 -53.0632

C 11.0121 17.1553 -52.3092

H 10.7481 17.7193 -53.2072

H 10.7841 17.7873 -51.4482

C 12.5081 16.8543 -52.3302

H 12.7751 16.2773 -51.4412

H 12.7411 16.2403 -53.2032

C 13.3401 18.1333 -52.3742

H 13.1131 18.7403 -51.4962

H 13.0571 18.7163 -53.2542

C 14.8391 17.8493 -52.4132

H 15.1221 17.2893 -51.5192

H 15.0701 17.2233 -53.2782

C 15.6581 19.1353 -52.4852

H 15.4081 19.6713 -53.4052

H 15.3871 19.7793 -51.6472

C 17.1601 18.8693 -52.4462

H 17.4401 18.2373 -53.2932

H 17.4041 18.3213 -51.5332

C 17.9681 20.1633 -52.4872

H 17.7441 20.6993 -53.4132

H 17.6641 20.8003 -51.6552

C 19.4671 19.9053 -52.4022

H 19.7101 19.3743 -51.4842

H 20.0181 20.8433 -52.4112

H 19.8011 19.3013 -53.2432

O 5.2011 17.4933 -49.0552

O 2.7791 18.4743 -48.7772

O 5.9461 16.1753 -51.3162

O 0.1971 15.9903 -52.3722

C -2.0399 15.7353 -48.2882

C -0.8549 15.9343 -47.5712

C -3.2509 15.6073 -47.5972

C -0.8879 16.0473 -46.1982

H 0.0801 16.0023 -48.1062

C -3.2769 15.7113 -46.2262

H -4.1599 15.4363 -48.1532

C -2.1009 15.9523 -45.5152

H 0.0201 16.2123 -45.6392

H -4.2029 15.6233 -45.6782

C -2.4839 15.6633 -59.0972

C -1.4009 16.0203 -59.9072

C -3.7039 15.3253 -59.6932

C -1.5509 16.0953 -61.2722

H -0.4499 16.2463 -59.4492

C -3.8519 15.3993 -61.0582

H -4.5229 15.0073 -59.0662

C -2.7859 15.8123 -61.8592

H -0.7269 16.3883 -61.9042

H -4.7889 15.1353 -61.5242

C -5.8019 16.2003 -54.2502

H -6.1679 15.3103 -53.7132

H -5.5399 16.9663 -53.5092

C 1.3341 16.4863 -53.0512

H 1.7151 15.7423 -53.7642

H 1.0551 17.3963 -53.6062

C -1.5319 25.6183 -31.4612

C -1.3269 25.6313 -37.1252

C -1.3939 25.5783 -38.3322

C -1.5789 25.5213 -39.7252

C -0.5139 25.2533 -40.6092

C -2.8749 25.6923 -40.2232

H -3.6639 25.8543 -39.5052

O -2.6159 25.6343 -30.8952

O -0.3879 25.5913 -30.8132

H -0.5269 25.6123 -29.8152

C -1.8019 25.0693 -50.7132

C -2.1719 25.4683 -45.0742

C -2.2149 25.4813 -43.8652

C -2.0669 25.4763 -42.4662

C -3.1489 25.6423 -41.5802

C -0.7809 25.2673 -41.9682

H -0.0079 25.0943 -42.6982

O -0.7229 25.0233 -51.2902

O -2.9559 25.0023 -51.3312

H -2.8449 24.9263 -52.3302

C -6.7629 25.5283 -42.1292

C -6.7249 25.7783 -43.4882

H -5.7739 25.9863 -43.9472

C -7.9009 25.7553 -44.2292

C -9.1249 25.4903 -43.6012

C -9.1569 25.2583 -42.2172

C -7.9679 25.2553 -41.4972

H -7.9699 25.0543 -40.4372

C -6.7819 26.0383 -46.3032

H -6.1559 26.8853 -45.9852

H -6.2239 25.1053 -46.1302

C -7.1109 26.1813 -47.7872

H -7.6339 27.1263 -47.9422

H -6.1629 26.2273 -48.3272

C -7.9729 25.0323 -48.3172

H -7.7129 24.1153 -47.7862

H -7.7559 24.8773 -49.3782

C -9.4659 25.3033 -48.1462

H -9.6629 25.5633 -47.1062

H -9.7429 26.1603 -48.7652

C -10.3249 24.1023 -48.5372

H -10.1249 23.2743 -47.8552

H -10.0639 23.7733 -49.5482

C -11.8089 24.4543 -48.4882

H -12.0339 24.8893 -47.5132

H -12.0249 25.2143 -49.2442

C -12.7109 23.2443 -48.7142

H -12.5119 22.5073 -47.9332

H -12.4709 22.7803 -49.6732

C -14.1859 23.6363 -48.6922

H -14.4129 24.1223 -47.7412

H -14.3759 24.3653 -49.4852

C -15.1159 22.4413 -48.8782

H -14.9309 21.7183 -48.0792

H -14.8889 21.9473 -49.8252

C -16.5869 22.8483 -48.8562

H -16.7729 23.5693 -49.6562

H -16.8099 23.3433 -47.9082

C -17.5169 21.6513 -49.0302

H -17.2859 21.1533 -49.9742

H -17.3319 20.9343 -48.2262

C -18.9849 22.0543 -49.0152

H -19.1909 22.7713 -49.8062

H -19.6229 21.1863 -49.1662

H -19.2459 22.5123 -48.0642

C -11.0849 24.3933 -44.4072

H -10.8439 23.8123 -45.3072

H -10.9079 23.7563 -43.5372

C -12.5399 24.8483 -44.4432

H -12.7569 25.3933 -43.5232

H -12.6649 25.5453 -45.2752

C -13.4879 23.6673 -44.6002

H -13.3239 22.9653 -43.7792

H -13.2529 23.1403 -45.5262

C -14.9509 24.1033 -44.6152

H -15.1819 24.6353 -43.6892

H -15.1129 24.8013 -45.4402

C -15.8919 22.9123 -44.7672

H -15.7139 22.2093 -43.9482

H -15.6629 22.3903 -45.6982

C -17.3629 23.3183 -44.7642

H -17.5959 23.8383 -43.8332

H -17.5499 24.0163 -45.5842

C -18.2749 22.1043 -44.9132

H -18.0719 21.4043 -44.0982

H -18.0399 21.5903 -45.8472

C -19.7559 22.4683 -44.8972

H -19.9979 22.9663 -43.9552

H -19.9679 23.1743 -45.7042

C -20.6349 21.2313 -45.0602

H -20.3959 20.5193 -44.2652

H -20.4019 20.7463 -46.0102

C -22.1249 21.5533 -45.0082

H -22.3589 22.0353 -44.0562

H -22.3739 22.2583 -45.8052

C -22.9779 20.2963 -45.1602

H -22.6949 19.5783 -44.3862

H -22.7669 19.8333 -46.1272

C -24.4679 20.5893 -45.0502

H -24.7749 21.3003 -45.8142

H -25.0469 19.6773 -45.1792

H -24.7029 21.0113 -44.0752

C -10.5309 25.0393 -40.2652

H -10.2189 24.0603 -39.8722

H -9.9069 25.8163 -39.8072

C -11.9949 25.3143 -39.9302

H -12.0869 25.4053 -38.8452

H -12.2699 26.2713 -40.3782

C -12.9379 24.2293 -40.4432

H -12.7719 24.0933 -41.5122

H -12.7089 23.2843 -39.9462

C -14.3979 24.6023 -40.1922

H -14.6309 25.5233 -40.7322

H -14.5399 24.8053 -39.1262

C -15.3639 23.5043 -40.6272

H -15.1639 22.6003 -40.0492

H -15.1919 23.2693 -41.6782

C -16.8259 23.9053 -40.4392

H -17.0519 24.7633 -41.0762

H -16.9879 24.2153 -39.4042

C -17.7739 22.7573 -40.7762

H -17.5799 22.4153 -41.7942

H -17.5709 21.9193 -40.1052

C -19.2429 23.1533 -40.6522

H -19.4669 23.9463 -41.3702

H -19.4279 23.5563 -39.6532

C -20.1699 21.9653 -40.8972

H -19.9579 21.1903 -40.1592

H -19.9649 21.5443 -41.8832

C -21.6459 22.3463 -40.8102

H -21.8449 22.8153 -39.8432

H -21.8769 23.0833 -41.5842

C -22.5559 21.1323 -40.9782

H -22.3379 20.4093 -40.1882

H -22.3419 20.6503 -41.9332

C -24.0309 21.5093 -40.9222

H -24.2719 22.2273 -41.7022

H -24.6559 20.6293 -41.0592

H -24.2749 21.9573 -39.9612

O -10.2529 25.5473 -44.3732

O -7.9779 26.0023 -45.5612

O -10.3889 25.0593 -41.6702

O -4.3799 25.7413 -42.1352

C 2.9881 24.2283 -40.3022

C 3.0681 23.5773 -39.0842

H 2.1571 23.2713 -38.5972

C 4.3111 23.3553 -38.5022

C 5.4811 23.7663 -39.1602

C 5.3901 24.3983 -40.4082

C 4.1381 24.6373 -40.9572

H 4.0451 25.1413 -41.9052

C 3.3881 22.4903 -36.4852

H 2.7311 21.7513 -36.9682

H 2.8161 23.4163 -36.3282

C 3.8751 21.9423 -35.1452

H 4.3201 20.9603 -35.3142

H 2.9931 21.8143 -34.5122

C 4.8951 22.8533 -34.4482

H 4.6981 23.8923 -34.7172

H 4.7741 22.7673 -33.3642

C 6.3331 22.4933 -34.8192

H 6.4351 22.5083 -35.9032

H 6.5361 21.4763 -34.4722

C 7.3691 23.4323 -34.2022

H 7.2871 24.4183 -34.6652

H 7.1711 23.5553 -33.1342

C 8.7851 22.8923 -34.3982

H 8.9121 22.6053 -35.4442

H 8.9111 21.9923 -33.7902

C 9.8671 23.9013 -34.0222

H 9.7731 24.7843 -34.6582

H 9.7201 24.2233 -32.9882

C 11.2711 23.3203 -34.1742

H 11.4031 22.9573 -35.1972

H 11.3801 22.4633 -33.5052

C 12.3581 24.3443 -33.8582

H 12.2611 25.1943 -34.5372

H 12.2131 24.7173 -32.8422

C 13.7631 23.7603 -33.9822

H 13.8631 22.9103 -33.3022

H 13.9151 23.3883 -34.9982

C 14.8421 24.7903 -33.6552

H 14.6811 25.1693 -32.6442

H 14.7541 25.6343 -34.3422

C 16.2441 24.2013 -33.7552

H 16.3611 23.3703 -33.0622

H 16.9931 24.9533 -33.5182

H 16.4331 23.8333 -34.7622

C 7.4191 24.6783 -38.1652

H 7.0891 24.9763 -37.1592

H 7.2251 25.5093 -38.8502

C 8.9041 24.3413 -38.1662

H 9.1621 23.9753 -39.1602

H 9.0771 23.5333 -37.4542

C 9.7851 25.5343 -37.8092

H 9.6101 26.3493 -38.5162

H 9.5231 25.9003 -36.8142

C 11.2591 25.1373 -37.8392

H 11.5041 24.7573 -38.8342

H 11.4221 24.3263 -37.1272

C 12.2031 26.2883 -37.5002

H 12.0551 27.1083 -38.2082

H 11.9721 26.6703 -36.5032

C 13.6581 25.8283 -37.5472

H 13.8801 25.4513 -38.5492

H 13.7921 25.0013 -36.8472

C 14.6481 26.9373 -37.2012

H 14.5291 27.7673 -37.9022

H 14.4331 27.3193 -36.2012

C 16.0861 26.4283 -37.2542

H 16.2911 26.0443 -38.2572

H 16.1981 25.5973 -36.5532

C 17.1081 27.5093 -36.9132

H 16.9971 28.3453 -37.6082

H 16.9151 27.8883 -35.9072

C 18.5381 26.9773 -36.9872

H 18.7171 26.5633 -37.9832

H 18.6581 26.1633 -36.2682

C 19.5761 28.0573 -36.7012

H 19.4401 28.8843 -37.4012

H 19.4221 28.4493 -35.6932

C 20.9971 27.5213 -36.8252

H 21.1591 26.7043 -36.1252

H 21.7241 28.3023 -36.6152

H 21.1761 27.1473 -37.8322

C 6.5051 25.4503 -42.2362

H 6.0211 26.4263 -42.0872

H 5.9241 24.8783 -42.9732

C 7.9231 25.6413 -42.7612

H 7.8581 26.1803 -43.7082

H 8.3581 24.6603 -42.9572

C 8.8201 26.4053 -41.7912

H 8.9281 25.8233 -40.8762

H 8.3531 27.3573 -41.5302

C 10.1931 26.6603 -42.4072

H 10.6001 25.7153 -42.7752

H 10.0831 27.3253 -43.2662

C 11.1841 27.2703 -41.4202

H 10.7801 28.2033 -41.0192

H 11.3261 26.5843 -40.5822

C 12.5311 27.5403 -42.0862

H 12.8801 26.6213 -42.5652

H 12.4021 28.2903 -42.8702

C 13.5951 28.0163 -41.1002

H 13.7261 27.2593 -40.3232

H 13.2601 28.9353 -40.6132

C 14.9341 28.2623 -41.7922

H 15.2241 27.3633 -42.3402

H 14.8241 29.0683 -42.5222

C 16.0401 28.6183 -40.8032

H 15.7741 29.5353 -40.2702

H 16.1261 27.8213 -40.0612

C 17.3891 28.8053 -41.4942

H 17.3321 29.6523 -42.1822

H 17.6171 27.9163 -42.0882

C 18.5181 29.0363 -40.4932

H 18.3011 29.9313 -39.9042

H 18.5621 28.1893 -39.8062

C 19.8681 29.1973 -41.1802

H 20.1081 28.3073 -41.7582

H 20.6551 29.3523 -40.4462

H 19.8551 30.0493 -41.8562

O 6.6961 23.5193 -38.5662

O 4.5061 22.7563 -37.3012

O 6.5611 24.7523 -41.0082

O 0.7061 24.9863 -40.0762

C -1.3329 25.6543 -35.7182

C -0.1659 25.4623 -34.9722

C -2.5479 25.8503 -35.0502

C -0.2119 25.4593 -33.5952

H 0.7711 25.3183 -35.4912

C -2.5929 25.8333 -33.6762

H -3.4479 26.0033 -35.6272

C -1.4269 25.6383 -32.9342

H 0.6881 25.3193 -33.0162

H -3.5249 25.9683 -33.1482

C -2.0999 25.4233 -46.4792

C -0.8539 25.4993 -47.1102

C -3.2529 25.2743 -47.2542

C -0.7639 25.3973 -48.4802

H 0.0321 25.6353 -46.5082

C -3.1599 25.1733 -48.6242

H -4.2109 25.2253 -46.7642

C -1.9139 25.2193 -49.2482

H 0.1901 25.4493 -48.9802

H -4.0449 25.0423 -49.2262

C -5.5049 25.5903 -41.2992

H -5.4039 24.6713 -40.7042

H -5.5799 26.4433 -40.6052

C 1.6701 24.4753 -40.9782

H 1.2901 23.5343 -41.4062

H 1.8351 25.1893 -41.7952

C -0.8479 25.6623 -8.4002

C -1.3109 26.0793 -14.0302

C -1.4659 26.0853 -15.2292

C -1.7059 26.0853 -16.6152

C -0.6439 25.9803 -17.5372

C -3.0239 26.1423 -17.0762

H -3.8079 26.1843 -16.3372

O -1.8439 25.6533 -7.6872

O 0.3731 25.5563 -7.9182

H 0.3771 25.4843 -6.9192

C -1.8809 25.8493 -27.5882

C -2.4099 26.1883 -21.9542

C -2.4349 26.1663 -20.7442

C -2.2579 26.1233 -19.3492

C -3.3259 26.1283 -18.4302

C -0.9429 26.0333 -18.8882

H -0.1669 25.9883 -19.6352

O -0.7849 25.7913 -28.1292

O -3.0129 25.7993 -28.2492

H -2.8639 25.7363 -29.2422

C -6.8819 25.5123 -18.9232

C -6.7759 25.1763 -20.2612

H -5.7949 25.0143 -20.6742

C -7.9219 25.0793 -21.0392

C -9.1869 25.2833 -20.4632

C -9.2849 25.5763 -19.0952

C -8.1249 25.6923 -18.3382

H -8.1789 25.9393 -17.2892

C -6.7509 24.5253 -23.0482

H -5.9579 25.2313 -22.7702

H -6.4209 23.5113 -22.7752

C -7.0029 24.6133 -24.5532

H -7.2349 25.6473 -24.8132

H -6.0719 24.3393 -25.0542

C -8.1459 23.7073 -25.0182

H -8.1539 22.8043 -24.4072

H -7.9739 23.4123 -26.0572

C -9.5129 24.3803 -24.9082

H -9.6249 24.7943 -23.9072

H -9.5699 25.2043 -25.6252

C -10.6509 23.3963 -25.1652

H -10.5949 22.5893 -24.4322

H -10.5429 22.9543 -26.1602

C -12.0139 24.0743 -25.0532

H -12.0679 24.5943 -24.0962

H -12.1199 24.8253 -25.8412

C -13.1579 23.0683 -25.1422

H -13.0299 22.3303 -24.3472

H -13.1039 22.5373 -26.0952

C -14.5279 23.7303 -25.0092

H -14.5719 24.2803 -24.0672

H -14.6629 24.4543 -25.8172

C -15.6609 22.7083 -25.0502

H -15.5259 21.9973 -24.2312

H -15.6059 22.1443 -25.9842

C -17.0389 23.3533 -24.9302

H -17.1819 24.0593 -25.7532

H -17.0929 23.9213 -23.9992

C -18.1579 22.3163 -24.9552

H -18.0959 21.7403 -25.8812

H -18.0169 21.6193 -24.1252

C -19.5369 22.9533 -24.8472

H -19.7019 23.6513 -25.6652

H -20.3159 22.1933 -24.8842

H -19.6339 23.4983 -23.9112

C -11.3019 24.3233 -21.0232

H -11.2439 23.5223 -21.7722

H -11.1639 23.8753 -20.0372

C -12.6609 25.0143 -21.1022

H -12.7439 25.7263 -20.2792

H -12.7119 25.5803 -22.0342

C -13.7849 23.9873 -21.0462

H -13.6509 23.3673 -20.1562

H -13.7009 23.3283 -21.9112

C -15.1759 24.6143 -21.0172

H -15.2699 25.2633 -20.1432

H -15.3169 25.2373 -21.9032

C -16.2569 23.5363 -20.9752

H -16.0799 22.8903 -20.1102

H -16.1719 22.9133 -21.8672

C -17.6719 24.1003 -20.8882

H -17.7719 24.7023 -19.9822

H -17.8569 24.7583 -21.7412

C -18.7099 22.9813 -20.8752

H -18.5139 22.3213 -20.0252

H -18.6009 22.3833 -21.7822

C -20.1439 23.4943 -20.7822

H -20.2719 24.0543 -19.8532

H -20.3389 24.1823 -21.6092

C -21.1519 22.3493 -20.8302

H -20.9649 21.6733 -19.9912

H -21.0029 21.7793 -21.7512

C -22.5969 22.8343 -20.7682

H -22.7509 23.3993 -19.8462

H -22.7879 23.5083 -21.6062

C -23.5889 21.6743 -20.8192

H -23.4139 21.0143 -19.9662

H -23.4149 21.0943 -21.7292

C -25.0349 22.1523 -20.7932

H -25.2389 22.7993 -21.6432

H -25.7179 21.3073 -20.8342

H -25.2359 22.7103 -19.8812

C -10.6949 26.2023 -17.2692

H -10.3779 25.4163 -16.5672

H -10.0789 27.0953 -17.0982

C -12.1629 26.5483 -17.0422

H -12.2559 27.0163 -16.0602

H -12.4649 27.2763 -17.7972

C -13.0719 25.3253 -17.1222

H -12.9549 24.8623 -18.1022

H -12.7639 24.5963 -16.3692

C -14.5349 25.6993 -16.8982

H -14.8279 26.4593 -17.6262

H -14.6459 26.1383 -15.9032

C -15.4659 24.4963 -17.0262

H -15.1589 23.7233 -16.3192

H -15.3759 24.0773 -18.0292

C -16.9239 24.8663 -16.7682

H -17.2349 25.6283 -17.4872

H -17.0179 25.3023 -15.7702

C -17.8469 23.6563 -16.8822

H -17.7249 23.2033 -17.8682

H -17.5559 22.9103 -16.1402

C -19.3139 24.0243 -16.6802

H -19.6089 24.7553 -17.4372

H -19.4389 24.4983 -15.7032

C -20.2239 22.8033 -16.7742

H -19.9509 22.0903 -15.9922

H -20.0679 22.3133 -17.7372

C -21.6999 23.1643 -16.6282

H -21.8639 23.6483 -15.6632

H -21.9739 23.8803 -17.4062

C -22.5999 21.9363 -16.7372

H -22.3419 21.2293 -15.9432

H -22.4169 21.4423 -17.6932

C -24.0759 22.2963 -16.6312

H -24.3529 22.9983 -17.4152

H -24.6959 21.4093 -16.7312

H -24.2889 22.7593 -15.6692

O -10.2669 25.2593 -21.3022

O -7.9469 24.8273 -22.3702

O -10.5439 25.7563 -18.6002

O -4.5799 26.1133 -18.9402

C 2.9031 25.1913 -17.2382

C 2.9191 24.4683 -16.0592

H 1.9851 24.1073 -15.6652

C 4.1201 24.2583 -15.3902

C 5.3201 24.7483 -15.9322

C 5.2981 25.4363 -17.1552

C 4.0811 25.6703 -17.7832

H 4.0381 26.2273 -18.7052

C 3.0451 23.2213 -13.5432

H 2.5171 22.4543 -14.1282

H 2.3851 24.0943 -13.4342

C 3.3871 22.6633 -12.1632

H 3.9621 21.7453 -12.2892

H 2.4381 22.4113 -11.6822

C 4.1701 23.6473 -11.2882

H 3.8331 24.6653 -11.4962

H 3.9621 23.4423 -10.2342

C 5.6761 23.5493 -11.5252

H 5.8761 23.6393 -12.5922

H 6.0161 22.5613 -11.2042

C 6.4731 24.6103 -10.7702

H 6.2411 25.5983 -11.1762

H 6.1821 24.6153 -9.7152

C 7.9721 24.3453 -10.8912

H 8.2161 24.1833 -11.9432

H 8.2161 23.4273 -10.3492

C 8.8301 25.4843 -10.3492

H 8.6291 26.3933 -10.9202

H 8.5581 25.6863 -9.3102

C 10.3181 25.1503 -10.4332

H 10.5701 24.9013 -11.4672

H 10.5241 24.2693 -9.8212

C 11.2021 26.3013 -9.9662

H 11.0251 27.1753 -10.5982

H 10.9261 26.5783 -8.9452

C 12.6841 25.9363 -10.0102

H 12.8611 25.0683 -9.3712

H 12.9551 25.6563 -11.0312

C 13.5771 27.0853 -9.5522

H 13.2891 27.3853 -8.5412

H 13.4191 27.9483 -10.2042

C 15.0511 26.6983 -9.5682

H 15.2311 25.8553 -8.9052

H 15.6711 27.5303 -9.2422

H 15.3581 26.4163 -10.5722

C 7.1801 25.6673 -14.7842

H 6.8271 25.8993 -13.7692

H 6.9731 26.5283 -15.4262

C 8.6741 25.3733 -14.7772

H 8.9651 25.0923 -15.7902

H 8.8561 24.5163 -14.1272

C 9.5161 26.5553 -14.3052

H 9.3241 27.4233 -14.9402

H 9.2341 26.8233 -13.2852

C 11.0031 26.2093 -14.3502

H 11.2721 25.9293 -15.3712

H 11.1851 25.3403 -13.7132

C 11.9031 27.3533 -13.8912

H 11.7391 28.2283 -14.5242

H 11.6441 27.6323 -12.8682

C 13.3751 26.9513 -13.9492

H 13.6291 26.6693 -14.9742

H 13.5291 26.0703 -13.3212

C 14.3131 28.0613 -13.4862

H 14.1831 28.9403 -14.1222

H 14.0571 28.3513 -12.4662

C 15.7721 27.6133 -13.5312

H 16.0251 27.3213 -14.5542

H 15.8971 26.7313 -12.8992

C 16.7351 28.7023 -13.0672

H 16.6251 29.5803 -13.7092

H 16.4791 29.0023 -12.0492

C 18.1861 28.2273 -13.1052

H 18.4241 27.8773 -14.1142

H 18.3061 27.3783 -12.4282

C 19.1691 29.3263 -12.7122

H 19.0321 30.1863 -13.3712

H 18.9551 29.6533 -11.6922

C 20.6131 28.8473 -12.8002

H 20.7741 27.9983 -12.1402

H 21.2991 29.6403 -12.5132

H 20.8501 28.5403 -13.8172

C 6.5031 26.5683 -18.8712

H 6.0061 27.5383 -18.7312

H 5.9651 26.0063 -19.6482

C 7.9431 26.7763 -19.3222

H 7.9201 27.3123 -20.2722

H 8.3981 25.7993 -19.4962

C 8.7861 27.5503 -18.3132

H 8.8571 26.9723 -17.3912

H 8.2971 28.4993 -18.0762

C 10.1821 27.8173 -18.8672

H 10.6141 26.8743 -19.2102

H 10.1041 28.4743 -19.7362

C 11.1231 28.4463 -17.8432

H 10.6981 29.3833 -17.4782

H 11.2271 27.7743 -16.9892

C 12.5001 28.7093 -18.4492

H 12.8761 27.7823 -18.8882

H 12.4051 29.4393 -19.2572

C 13.5101 29.2163 -17.4252

H 13.6051 28.4823 -16.6222

H 13.1481 30.1463 -16.9812

C 14.8821 29.4503 -18.0542

H 15.2061 28.5353 -18.5562

H 14.8061 30.2293 -18.8162

C 15.9311 29.8493 -17.0192

H 15.6311 30.7823 -16.5362

H 15.9801 29.0793 -16.2462

C 17.3151 30.0223 -17.6402

H 17.2871 30.8293 -18.3762

H 17.5911 29.1053 -18.1692

C 18.3781 30.3293 -16.5892

H 18.1151 31.2533 -16.0682

H 18.3911 29.5253 -15.8512

C 19.7661 30.4693 -17.2022

H 20.0521 29.5513 -17.7102

H 20.5051 30.6813 -16.4322

H 19.7841 31.2803 -17.9272

O 6.4931 24.5113 -15.2572

O 4.2301 23.6023 -14.2082

O 6.4971 25.8473 -17.6552

O 0.6061 25.8213 -17.0382

C -1.1719 26.0163 -12.6312

C 0.0841 25.8353 -12.0412

C -2.3059 26.1063 -11.8142

C 0.2021 25.7263 -10.6732

H 0.9571 25.7833 -12.6752

C -2.1859 25.9933 -10.4492

H -3.2739 26.2533 -12.2692

C -0.9329 25.7983 -9.8642

H 1.1701 25.5833 -10.2172

H -3.0539 26.0463 -9.8092

C -2.3099 26.1663 -23.3582

C -1.0459 26.0063 -23.9392

C -3.4309 26.2793 -24.1852

C -0.9139 25.9233 -25.3052

H -0.1799 25.9373 -23.2972

C -3.2979 26.1833 -25.5532

H -4.4019 26.4393 -23.7432

C -2.0399 25.9893 -26.1262

H 0.0541 25.7943 -25.7642

H -4.1619 26.2563 -26.1942

C -5.6359 25.6993 -18.0962

H -5.3599 24.7533 -17.6062

H -5.8259 26.4523 -17.3182

C 1.6101 25.4443 -17.9592

H 1.2921 24.5293 -18.4832

H 1.7631 26.2383 -18.7022

C -2.1019 23.0003 -20.1552

C -2.0219 22.7103 -25.7992

C -2.0029 22.6393 -27.0052

C -2.0959 22.5863 -28.4052

C -0.9409 22.5933 -29.2122

C -3.3649 22.5323 -28.9802

H -4.2009 22.5023 -28.3022

O -3.1469 23.0663 -19.5232

O -0.9199 22.9853 -19.5822

H -1.0049 23.0223 -18.5772

C -2.4029 22.5873 -39.4252

C -2.4429 22.6613 -33.7742

C -2.4729 22.6223 -32.5652

C -2.3859 22.5853 -31.1642

C -3.5399 22.5203 -30.3552

C -1.1129 22.6043 -30.5862

H -0.2689 22.6353 -31.2562

O -1.3529 22.4963 -40.0532

O -3.5769 22.6603 -40.0062

H -3.5089 22.6093 -41.0162

C -7.1639 22.2693 -30.9042

C -7.2299 22.1983 -32.2832

H -6.3169 22.0753 -32.8422

C -8.4639 22.3193 -32.9152

C -9.6329 22.4983 -32.1612

C -9.5579 22.5453 -30.7622

C -8.3189 22.4183 -30.1492

H -8.2369 22.4573 -29.0742

C -7.6419 21.8983 -35.1222

H -6.7339 22.5063 -34.9922

H -7.3929 20.8503 -34.9042

C -8.1429 22.0353 -36.5592

H -8.3539 23.0873 -36.7572

H -7.3279 21.7223 -37.2172

C -9.3959 21.2003 -36.8412

H -9.3309 20.2603 -36.2922

H -9.4279 20.9673 -37.9112

C -10.6969 21.9053 -36.4572

H -10.6439 22.2143 -35.4152

H -10.8189 22.8053 -37.0672

C -11.8979 20.9833 -36.6602

H -11.7739 20.1093 -36.0182

H -11.9129 20.6303 -37.6952

C -13.2259 21.6683 -36.3442

H -13.2129 22.0143 -35.3102

H -13.3439 22.5483 -36.9812

C -14.4159 20.7343 -36.5542

H -14.2979 19.8523 -35.9202

H -14.4229 20.3813 -37.5882

C -15.7569 21.3963 -36.2452

H -15.7729 21.7123 -35.2002

H -15.8729 22.2913 -36.8612

C -16.9239 20.4473 -36.5062

H -16.7929 19.5423 -35.9072

H -16.9119 20.1393 -37.5552

C -18.2779 21.0713 -36.1812

H -18.4209 21.9683 -36.7882

H -18.2939 21.3763 -35.1332

C -19.4269 20.0993 -36.4382

H -19.4059 19.7833 -37.4842

H -19.2829 19.2033 -35.8302

C -20.7839 20.7133 -36.1212

H -20.9559 21.5953 -36.7332

H -21.5829 20.0003 -36.3152

H -20.8339 21.0073 -35.0752

C -11.8579 21.7783 -32.5952

H -11.9149 21.0963 -33.4522

H -11.6459 21.1813 -31.7052

C -13.1689 22.5433 -32.4282

H -13.0999 23.1663 -31.5362

H -13.2909 23.2083 -33.2862

C -14.3599 21.5973 -32.3272

H -14.2329 20.9523 -31.4532

H -14.3809 20.9513 -33.2072

C -15.6919 22.3353 -32.2162

H -15.6879 22.9663 -31.3252

H -15.8149 22.9913 -33.0822

C -16.8659 21.3623 -32.1472

H -16.7579 20.7343 -31.2592

H -16.8409 20.7033 -33.0182

C -18.2169 22.0703 -32.0932

H -18.2479 22.7283 -31.2232

H -18.3339 22.6963 -32.9822

C -19.3739 21.0763 -32.0172

H -19.2719 20.4783 -31.1092

H -19.3209 20.3943 -32.8692

C -20.7359 21.7663 -32.0082

H -20.7859 22.4563 -31.1632

H -20.8479 22.3573 -32.9212

C -21.8819 20.7633 -31.9092

H -21.7859 20.2023 -30.9762

H -21.8109 20.0483 -32.7332

C -23.2499 21.4393 -31.9492

H -23.3189 22.1653 -31.1352

H -23.3539 21.9883 -32.8882

C -24.3909 20.4333 -31.8252

H -24.2979 19.9003 -30.8762

H -24.3099 19.6993 -32.6302

C -25.7559 21.1043 -31.8922

H -25.8789 21.6253 -32.8392

H -26.5509 20.3673 -31.8012

H -25.8679 21.8283 -31.0882

C -10.6889 23.0713 -28.7202

H -10.4499 22.1823 -28.1182

H -9.9199 23.8343 -28.5412

C -12.0569 23.6243 -28.3302

H -11.9949 24.0293 -27.3192

H -12.2979 24.4423 -29.0132

C -13.1499 22.5633 -28.4032

H -13.1089 22.0843 -29.3822

H -12.9629 21.7973 -27.6462

C -14.5389 23.1573 -28.1962

H -14.7339 23.8963 -28.9772

H -14.5739 23.6773 -27.2362

C -15.6269 22.0883 -28.2322

H -15.4659 21.3833 -27.4112

H -15.5519 21.5263 -29.1642

C -17.0249 22.6883 -28.1172

H -17.1969 23.3623 -28.9592

H -17.0879 23.2823 -27.2032

C -18.1109 21.6173 -28.0972

H -18.0319 21.0023 -28.9962

H -17.9529 20.9633 -27.2362

C -19.5099 22.2223 -28.0212

H -19.6879 22.8283 -28.9132

H -19.5699 22.8853 -27.1562

C -20.5939 21.1543 -27.9102

H -20.4359 20.5793 -26.9942

H -20.5099 20.4663 -28.7552

C -21.9969 21.7553 -27.8902

H -22.0869 22.4353 -27.0392

H -22.1489 22.3423 -28.7992

C -23.0819 20.6853 -27.8002

H -22.9439 20.1063 -26.8832

H -22.9799 19.9993 -28.6442

C -24.4799 21.2893 -27.8112

H -24.6399 21.8593 -28.7232

H -25.2369 20.5093 -27.7582

H -24.6149 21.9573 -26.9622

O -10.8019 22.6993 -32.8432

O -8.6609 22.3333 -34.2562

O -10.7289 22.7343 -30.0922

O -4.7409 22.4423 -30.9782

C 2.6341 22.5883 -28.4362

C 2.6111 23.2683 -27.2292

H 1.7011 23.7683 -26.9412

C 3.7501 23.3023 -26.4282

C 4.9191 22.6673 -26.8672

C 4.9361 21.9893 -28.0912

C 3.7821 21.9403 -28.8642

H 3.7741 21.4063 -29.8012

C 2.7121 24.5843 -24.7062

H 1.8421 23.9153 -24.7452

H 2.4881 25.4813 -25.3022

C 2.9761 24.9703 -23.2532

H 3.1681 24.0603 -22.6812

H 2.0571 25.4213 -22.8742

C 4.1491 25.9403 -23.0742

H 4.2511 26.5533 -23.9722

H 3.9341 26.6153 -22.2422

C 5.4731 25.2293 -22.7942

H 5.6661 24.5003 -23.5812

H 5.3901 24.6853 -21.8492

C 6.6311 26.2223 -22.7002

H 6.8041 26.6663 -23.6832

H 6.3541 27.0333 -22.0202

C 7.9241 25.5803 -22.2012

H 8.1831 24.7383 -22.8452

H 7.7651 25.1893 -21.1932

C 9.0801 26.5783 -22.1822

H 9.2801 26.9133 -23.2022

H 8.7891 27.4583 -21.6022

C 10.3581 25.9923 -21.5882

H 10.6581 25.1153 -22.1652

H 10.1631 25.6653 -20.5642

C 11.4981 27.0083 -21.5862

H 11.6881 27.3343 -22.6112

H 11.1971 27.8873 -21.0112

C 12.7871 26.4433 -20.9952

H 12.6031 26.1203 -19.9682

H 13.0941 25.5643 -21.5672

C 13.9181 27.4693 -21.0092

H 13.6191 28.3443 -20.4282

H 14.0921 27.7973 -22.0352

C 15.2091 26.9013 -20.4332

H 15.0591 26.5903 -19.4022

H 16.0011 27.6463 -20.4562

H 15.5351 26.0343 -21.0052

C 6.8701 23.8673 -26.4002

H 6.3981 24.7663 -25.9792

H 6.9511 24.0003 -27.4882

C 8.2491 23.6533 -25.7942

H 8.6871 22.7583 -26.2392

H 8.1271 23.4703 -24.7252

C 9.1721 24.8483 -26.0102

H 9.2581 25.0563 -27.0792

H 8.7381 25.7313 -25.5362

C 10.5601 24.5903 -25.4282

H 11.0041 23.7253 -25.9252

H 10.4631 24.3473 -24.3682

C 11.4921 25.7893 -25.5832

H 11.5781 26.0483 -26.6412

H 11.0621 26.6513 -25.0682

C 12.8801 25.5023 -25.0162

H 13.3241 24.6613 -25.5542

H 12.7851 25.2093 -23.9682

C 13.8111 26.7083 -25.1162

H 13.8841 27.0243 -26.1602

H 13.3851 27.5423 -24.5522

C 15.2061 26.3933 -24.5822

H 15.6411 25.5823 -25.1692

H 15.1261 26.0463 -23.5502

C 16.1331 27.6043 -24.6342

H 16.1891 27.9723 -25.6622

H 15.7181 28.4093 -24.0222

C 17.5381 27.2683 -24.1402

H 17.9401 26.4423 -24.7312

H 17.4851 26.9363 -23.1002

C 18.4891 28.4573 -24.2412

H 18.5251 28.8023 -25.2772

H 18.1071 29.2803 -23.6322

C 19.8951 28.0943 -23.7812

H 19.8841 27.7593 -22.7462

H 20.5601 28.9513 -23.8572

H 20.3001 27.2913 -24.3942

C 6.3211 21.0063 -29.7662

H 6.0021 21.7913 -30.4662

H 5.7351 20.0993 -29.9702

C 7.8101 20.7263 -29.9452

H 7.9821 20.4493 -30.9842

H 8.0901 19.8843 -29.3112

C 8.6551 21.9433 -29.5762

H 8.4121 22.2313 -28.5532

H 8.3941 22.7763 -30.2332

C 10.1531 21.6733 -29.6762

H 10.4151 20.8293 -29.0342

H 10.4091 21.3973 -30.7022

C 10.9591 22.8993 -29.2572

H 10.7031 23.7363 -29.9112

H 10.6751 23.1793 -28.2402

C 12.4661 22.6693 -29.3082

H 12.7311 21.8473 -28.6382

H 12.7551 22.3753 -30.3192

C 13.2371 23.9223 -28.9032

H 12.9241 24.2263 -27.9022

H 12.9841 24.7373 -29.5862

C 14.7481 23.7093 -28.9132

H 15.0031 22.9133 -28.2102

H 15.0621 23.3823 -29.9062

C 15.5051 24.9783 -28.5342

H 15.2771 25.7683 -29.2552

H 15.1631 25.3203 -27.5552

C 17.0151 24.7593 -28.4872

H 17.3661 24.4293 -29.4682

H 17.2391 23.9633 -27.7742

C 17.7681 26.0223 -28.0782

H 17.5991 26.8023 -28.8242

H 17.3721 26.3833 -27.1282

C 19.2651 25.7723 -27.9352

H 19.4541 25.0163 -27.1762

H 19.7801 26.6853 -27.6452

H 19.6861 25.4233 -28.8762

O 6.0721 22.7273 -26.1102

O 3.8491 23.9353 -25.2312

O 6.1231 21.4373 -28.4362

O 0.2461 22.5993 -28.5622

C -2.0329 22.7823 -24.3972

C -0.8369 22.7153 -23.6762

C -3.2459 22.9183 -23.7142

C -0.8519 22.7863 -22.3032

H 0.0921 22.5993 -24.2152

C -3.2569 22.9863 -22.3402

H -4.1659 22.9643 -24.2762

C -2.0619 22.9243 -21.6252

H 0.0671 22.7333 -21.7402

H -4.1789 23.0863 -21.7912

C -2.4329 22.6703 -35.1792

C -1.2439 22.4343 -35.8772

C -3.6159 22.8983 -35.8892

C -1.2429 22.4143 -37.2512

H -0.3329 22.2653 -35.3222

C -3.6139 22.8733 -37.2632

H -4.5259 23.0893 -35.3412

C -2.4279 22.6283 -37.9562

H -0.3369 22.2243 -37.8052

H -4.5249 23.0383 -37.8172

C -5.8639 22.2183 -30.1472

H -5.7579 21.2373 -29.6582

H -5.9079 22.9913 -29.3682

C 1.4251 22.5993 -29.3352

H 1.4461 21.7203 -29.9942

H 1.4561 23.5063 -29.9612

C -2.3559 22.4203 -42.6992

C -2.2699 22.0383 -48.3372

C -2.2469 21.9453 -49.5422

C -2.3169 21.8843 -50.9432

C -1.1479 21.9323 -51.7282

C -3.5729 21.7893 -51.5422

H -4.4239 21.7253 -50.8842

O -3.4059 22.5133 -42.0782

O -1.1789 22.3933 -42.1172

H -1.2679 22.4613 -41.1142

C -2.4679 22.1583 -61.9672

C -2.5529 21.9943 -56.3162

C -2.6159 21.9413 -55.1102

C -2.5549 21.8933 -53.7072

C -3.7199 21.7843 -52.9202

C -1.2939 21.9473 -53.1052

H -0.4409 22.0133 -53.7612

O -1.4189 22.2223 -62.6022

O -3.6479 22.1483 -62.5422

H -3.5949 22.2193 -63.5422

C -7.3009 21.5113 -53.6462

C -7.2579 21.3533 -55.0192

H -6.3139 21.1213 -55.4822

C -8.4129 21.5393 -55.7722

C -9.6199 21.8653 -55.1362

C -9.6659 21.9733 -53.7382

C -8.4989 21.7943 -53.0062

H -8.5069 21.8953 -51.9322

C -7.4309 20.8733 -57.8392

H -6.4729 21.3593 -57.6092

H -7.3619 19.8143 -57.5522

C -7.7039 20.9953 -59.3372

H -7.7709 22.0523 -59.6012

H -6.8369 20.5723 -59.8532

C -8.9819 20.2743 -59.7722

H -9.0929 19.3623 -59.1822

H -8.8899 19.9893 -60.8252

C -10.2379 21.1273 -59.6042

H -10.2649 21.5283 -58.5912

H -10.2009 21.9703 -60.3012

C -11.4959 20.3013 -59.8582

H -11.5119 19.4763 -59.1432

H -11.4539 19.8653 -60.8602

C -12.7789 21.1173 -59.7222

H -12.7809 21.6303 -58.7592

H -12.8079 21.8843 -60.5022

C -14.0189 20.2333 -59.8322

H -13.9999 19.4953 -59.0262

H -13.9839 19.6783 -60.7732

C -15.3279 21.0153 -59.7612

H -15.3819 21.5453 -58.8082

H -15.3509 21.7643 -60.5562

C -16.5349 20.0893 -59.8972

H -16.4909 19.3303 -59.1122

H -16.4799 19.5643 -60.8552

C -17.8659 20.8293 -59.8032

H -17.9249 21.5733 -60.6002

H -17.9179 21.3593 -58.8502

C -19.0509 19.8733 -59.9142

H -18.9969 19.3413 -60.8662

H -18.9839 19.1273 -59.1192

C -20.3889 20.5953 -59.8122

H -20.4819 21.3383 -60.6002

H -21.2129 19.8913 -59.9082

H -20.4779 21.1003 -58.8532

C -11.8149 21.2513 -55.7992

H -11.8209 20.5983 -56.6812

H -11.7019 20.6203 -54.9142

C -13.1099 22.0543 -55.7202

H -13.0899 22.6623 -54.8152

H -13.1509 22.7333 -56.5742

C -14.3289 21.1383 -55.7202

H -14.2679 20.4623 -54.8642

H -14.3139 20.5243 -56.6222

C -15.6489 21.9033 -55.6562

H -15.6809 22.5033 -54.7442

H -15.7109 22.5903 -56.5042

C -16.8419 20.9513 -55.6842

H -16.7819 20.2803 -54.8232

H -16.7859 20.3353 -56.5842

C -18.1839 21.6763 -55.6562

H -18.2509 22.2843 -54.7512

H -18.2509 22.3523 -56.5122

C -19.3509 20.6923 -55.6952

H -19.2959 20.0383 -54.8212

H -19.2599 20.0633 -56.5832

C -20.7079 21.3893 -55.7102

H -20.8099 22.0063 -54.8152

H -20.7639 22.0543 -56.5752

C -21.8579 20.3873 -55.7682

H -21.8239 19.7523 -54.8792

H -21.7309 19.7413 -56.6402

C -23.2209 21.0703 -55.8442

H -23.3459 21.7273 -54.9812

H -23.2619 21.6923 -56.7412

C -24.3659 20.0603 -55.8812

H -24.3429 19.4583 -54.9702

H -24.2239 19.3883 -56.7292

C -25.7239 20.7393 -55.9972

H -25.7779 21.3323 -56.9082

H -26.5219 20.0003 -56.0222

H -25.8969 21.4003 -55.1502

C -10.9429 22.6323 -51.8262

H -10.7599 21.7543 -51.1892

H -10.1829 23.3933 -51.6052

C -12.3319 23.2033 -51.5522

H -12.3339 23.6533 -50.5582

H -12.5259 23.9903 -52.2832

C -13.4259 22.1423 -51.6452

H -13.3429 21.6343 -52.6062

H -13.2759 21.3973 -50.8592

C -14.8219 22.7443 -51.5132

H -14.9789 23.4683 -52.3162

H -14.8989 23.2823 -50.5662

C -15.9099 21.6753 -51.5802

H -15.7939 20.9883 -50.7382

H -15.7899 21.0943 -52.4962

C -17.3139 22.2743 -51.5472

H -17.4339 22.9533 -52.3952

H -17.4359 22.8633 -50.6352

C -18.3959 21.2003 -51.6042

H -18.2499 20.5883 -52.4972

H -18.3019 20.5443 -50.7362

C -19.7999 21.7973 -51.6272

H -19.9019 22.4453 -52.5012

H -19.9449 22.4173 -50.7402

C -20.8799 20.7193 -51.6712

H -20.7899 20.0833 -50.7862

H -20.7249 20.0893 -52.5492

C -22.2859 21.3123 -51.7202

H -22.4499 21.9293 -50.8332

H -22.3709 21.9623 -52.5942

C -23.3619 20.2333 -51.7922

H -23.2819 19.5853 -50.9162

H -23.1899 19.6163 -52.6772

C -24.7629 20.8263 -51.8572

H -24.8629 21.4693 -52.7282

H -25.5109 20.0403 -51.9252

H -24.9689 21.4213 -50.9692

O -10.7079 22.1363 -55.9222

O -8.4809 21.4823 -57.1252

O -10.8779 22.2683 -53.1912

O -4.9009 21.6823 -53.5762

C 2.4291 21.9713 -50.9502

C 2.4341 22.6583 -49.7472

H 1.5351 23.1693 -49.4452

C 3.5911 22.6873 -48.9722

C 4.7471 22.0433 -49.4302

C 4.7371 21.3663 -50.6562

C 3.5651 21.3173 -51.4002

H 3.5351 20.7803 -52.3352

C 2.6321 24.0353 -47.2492

H 1.7281 23.4123 -47.2582

H 2.4441 24.9293 -47.8602

C 2.9451 24.4313 -45.8082

H 3.1431 23.5243 -45.2362

H 2.0441 24.8983 -45.4062

C 4.1331 25.3893 -45.6712

H 4.1801 26.0343 -46.5512

H 3.9751 26.0343 -44.8032

C 5.4671 24.6623 -45.5022

H 5.6031 23.9613 -46.3242

H 5.4411 24.0833 -44.5762

C 6.6371 25.6433 -45.4472

H 6.7311 26.1493 -46.4112

H 6.4261 26.4113 -44.6972

C 7.9591 24.9613 -45.1032

H 8.1671 24.1823 -45.8372

H 7.8691 24.4773 -44.1282

C 9.1201 25.9523 -45.0692

H 9.2511 26.3893 -46.0622

H 8.8791 26.7703 -44.3852

C 10.4301 25.3043 -44.6262

H 10.6811 24.4913 -45.3102

H 10.2971 24.8733 -43.6322

C 11.5811 26.3073 -44.5902

H 11.7111 26.7433 -45.5832

H 11.3301 27.1213 -43.9062

C 12.8951 25.6703 -44.1462

H 12.7661 25.2323 -43.1532

H 13.1541 24.8603 -44.8322

C 14.0391 26.6793 -44.1062

H 13.7851 27.4883 -43.4182

H 14.1681 27.1183 -45.0982

C 15.3481 26.0363 -43.6652

H 15.2401 25.6013 -42.6742

H 16.1471 26.7733 -43.6322

H 15.6381 25.2473 -44.3552

C 6.7781 23.1523 -49.0552

H 6.4381 24.0883 -48.5862

H 6.7621 23.2943 -50.1432

C 8.1851 22.8043 -48.5902

H 8.5251 21.9223 -49.1352

H 8.1361 22.5383 -47.5332

C 9.1691 23.9533 -48.7852

H 9.1821 24.2523 -49.8372

H 8.8381 24.8173 -48.2042

C 10.5751 23.5553 -48.3472

H 10.9311 22.7313 -48.9702

H 10.5371 23.1923 -47.3172

C 11.5661 24.7123 -48.4252

H 11.5801 25.1133 -49.4422

H 11.2371 25.5143 -47.7612

C 12.9731 24.2733 -48.0302

H 13.3201 23.5013 -48.7202

H 12.9431 23.8313 -47.0312

C 13.9661 25.4323 -48.0292

H 13.9681 25.9093 -49.0122

H 13.6461 26.1813 -47.3022

C 15.3791 24.9653 -47.6902

H 15.7061 24.2383 -48.4372

H 15.3681 24.4583 -46.7222

C 16.3761 26.1193 -47.6432

H 16.3431 26.6613 -48.5922

H 16.0871 26.8203 -46.8572

C 17.7991 25.6313 -47.3902

H 18.0691 24.9033 -48.1592

H 17.8411 25.1233 -46.4242

C 18.8161 26.7693 -47.4042

H 18.7641 27.2863 -48.3662

H 18.5621 27.4923 -46.6262

C 20.2351 26.2593 -47.1832

H 20.3121 25.7503 -46.2242

H 20.9451 27.0833 -47.1922

H 20.5131 25.5583 -47.9662

C 6.0611 20.2983 -52.3352

H 5.7251 21.0323 -53.0812

H 5.4601 19.3843 -52.4482

C 7.5421 19.9913 -52.5342

H 7.6781 19.5443 -53.5182

H 7.8491 19.2653 -51.7802

C 8.3961 21.2493 -52.4022

H 8.1351 21.7463 -51.4682

H 8.1661 21.9323 -53.2232

C 9.8901 20.9413 -52.3992

H 10.1131 20.2493 -51.5842

H 10.1671 20.4463 -53.3332

C 10.7191 22.2103 -52.2242

H 10.5361 22.8783 -53.0682

H 10.3941 22.7263 -51.3192

C 12.2141 21.9223 -52.1242

H 12.3991 21.2753 -51.2642

H 12.5421 21.3833 -53.0152

C 13.0331 23.2023 -51.9772

H 12.6861 23.7543 -51.1012

H 12.8681 23.8373 -52.8512

C 14.5241 22.9153 -51.8332

H 14.6861 22.3163 -50.9332

H 14.8641 22.3233 -52.6852

C 15.3581 24.1903 -51.7422

H 15.2321 24.7733 -52.6582

H 14.9981 24.7993 -50.9112

C 16.8391 23.8843 -51.5392

H 17.1971 23.2683 -52.3672

H 16.9601 23.3053 -50.6202

C 17.6921 25.1463 -51.4452

H 17.5811 25.7273 -52.3642

H 17.3321 25.7633 -50.6192

C 19.1641 24.8183 -51.2272

H 19.2971 24.2473 -50.3102

H 19.7541 25.7283 -51.1512

H 19.5491 24.2263 -52.0552

O 5.9071 22.0913 -48.6852

O 3.7261 23.3213 -47.7792

O 5.9191 20.8243 -51.0332

O 0.0311 21.9693 -51.0632

C -2.2849 22.1343 -46.9362

C -1.0849 22.2223 -46.2252

C -3.5019 22.1453 -46.2462

C -1.0989 22.3103 -44.8522

H -0.1529 22.2173 -46.7702

C -3.5119 22.2373 -44.8742

H -4.4259 22.0823 -46.8012

C -2.3129 22.3183 -44.1672

H -0.1779 22.3773 -44.2962

H -4.4359 22.2463 -44.3172

C -2.5269 22.0363 -57.7202

C -1.3219 21.8873 -58.4162

C -3.7139 22.2243 -58.4352

C -1.3089 21.9293 -59.7902

H -0.4059 21.7473 -57.8612

C -3.7029 22.2433 -59.8082

H -4.6349 22.3593 -57.8882

C -2.4989 22.1013 -60.4992

H -0.3879 21.8193 -60.3402

H -4.6199 22.3723 -60.3602

C -6.0549 21.4053 -52.8052

H -5.9669 20.3943 -52.3782

H -6.1419 22.1243 -51.9812

C 1.2121 21.9853 -51.8362

H 1.2361 21.1133 -52.5032

H 1.2351 22.8993 -52.4522

C -1.8059 24.8643 -55.3142

C -2.2689 25.2813 -60.9432

C -2.4239 25.2883 -62.1432

C -2.6639 25.2883 -63.5292

C -1.6019 25.1823 -64.4512

C -3.9819 25.3453 -63.9902

H -4.7659 25.3863 -63.2502

O -2.8019 24.8563 -54.6012

O -0.5849 24.7583 -54.8322

H -0.5809 24.6873 -53.8332

C -2.8389 25.0513 -74.5022

C -3.3679 25.3903 -68.8682

C -3.3929 25.3683 -67.6582

C -3.2159 25.3253 -66.2632

C -4.2839 25.3303 -65.3432

C -1.9009 25.2353 -65.8022

H -1.1249 25.1903 -66.5492

O -1.7429 24.9943 -75.0432

O -3.9709 25.0023 -75.1632

H -3.8219 24.9393 -76.1562

C -7.8399 24.7153 -65.8372

C -7.7339 24.3783 -67.1752

H -6.7529 24.2163 -67.5882

C -8.8799 24.2813 -67.9522

C -10.1449 24.4853 -67.3772

C -10.2429 24.7793 -66.0092

C -9.0829 24.8953 -65.2512

H -9.1369 25.1413 -64.2032

C -7.7089 23.7283 -69.9622

H -6.9159 24.4333 -69.6842

H -7.3789 22.7143 -69.6892

C -7.9609 23.8153 -71.4672

H -8.1929 24.8493 -71.7262

H -7.0289 23.5413 -71.9682

C -9.1039 22.9093 -71.9322

H -9.1119 22.0073 -71.3212

H -8.9309 22.6143 -72.9712

C -10.4709 23.5823 -71.8222

H -10.5829 23.9973 -70.8212

H -10.5279 24.4063 -72.5392

C -11.6089 22.5993 -72.0792

H -11.5529 21.7913 -71.3452

H -11.5009 22.1563 -73.0742

C -12.9719 23.2763 -71.9672

H -13.0259 23.7963 -71.0092

H -13.0779 24.0273 -72.7552

C -14.1159 22.2703 -72.0562

H -13.9879 21.5333 -71.2612

H -14.0619 21.7393 -73.0092

C -15.4859 22.9323 -71.9232

H -15.5299 23.4823 -70.9812

H -15.6209 23.6563 -72.7312

C -16.6189 21.9103 -71.9642

H -16.4839 21.1993 -71.1452

H -16.5639 21.3463 -72.8972

C -17.9969 22.5563 -71.8442

H -18.1399 23.2613 -72.6672

H -18.0509 23.1243 -70.9132

C -19.1159 21.5183 -71.8692

H -19.0539 20.9433 -72.7952

H -18.9749 20.8213 -71.0392

C -20.4949 22.1553 -71.7602

H -20.6599 22.8533 -72.5792

H -21.2739 21.3963 -71.7982

H -20.5919 22.7003 -70.8252

C -12.2599 23.5263 -67.9372

H -12.2019 22.7253 -68.6862

H -12.1219 23.0773 -66.9512

C -13.6189 24.2173 -68.0162

H -13.7019 24.9293 -67.1932

H -13.6699 24.7823 -68.9482

C -14.7429 23.1893 -67.9592

H -14.6079 22.5693 -67.0702

H -14.6589 22.5303 -68.8252

C -16.1339 23.8163 -67.9312

H -16.2279 24.4653 -67.0572

H -16.2749 24.4403 -68.8172

C -17.2149 22.7383 -67.8882

H -17.0379 22.0923 -67.0242

H -17.1299 22.1153 -68.7812

C -18.6299 23.3023 -67.8022

H -18.7299 23.9053 -66.8962

H -18.8149 23.9603 -68.6552

C -19.6679 22.1833 -67.7892

H -19.4719 21.5243 -66.9392

H -19.5589 21.5853 -68.6962

C -21.1019 22.6973 -67.6962

H -21.2299 23.2563 -66.7672

H -21.2969 23.3843 -68.5232

C -22.1099 21.5513 -67.7442

H -21.9229 20.8753 -66.9052

H -21.9609 20.9813 -68.6642

C -23.5549 22.0363 -67.6812

H -23.7089 22.6023 -66.7602

H -23.7459 22.7103 -68.5202

C -24.5469 20.8763 -67.7332

H -24.3719 20.2163 -66.8802

H -24.3729 20.2973 -68.6432

C -25.9919 21.3543 -67.7062

H -26.1969 22.0013 -68.5572

H -26.6759 20.5093 -67.7482

H -26.1939 21.9123 -66.7952

C -11.6529 25.4043 -64.1832

H -11.3359 24.6193 -63.4812

H -11.0359 26.2973 -64.0122

C -13.1209 25.7503 -63.9562

H -13.2139 26.2183 -62.9732

H -13.4229 26.4783 -64.7112

C -14.0299 24.5273 -64.0362

H -13.9129 24.0643 -65.0162

H -13.7219 23.7983 -63.2832

C -15.4929 24.9013 -63.8122

H -15.7859 25.6623 -64.5402

H -15.6039 25.3413 -62.8172

C -16.4239 23.6983 -63.9402

H -16.1169 22.9253 -63.2332

H -16.3339 23.2793 -64.9432

C -17.8819 24.0683 -63.6822

H -18.1929 24.8313 -64.4002

H -17.9749 24.5043 -62.6842

C -18.8049 22.8583 -63.7962

H -18.6829 22.4053 -64.7812

H -18.5139 22.1123 -63.0542

C -20.2709 23.2263 -63.5942

H -20.5669 23.9573 -64.3512

H -20.3969 23.7003 -62.6172

C -21.1819 22.0053 -63.6872

H -20.9089 21.2923 -62.9062

H -21.0259 21.5153 -64.6512

C -22.6579 22.3663 -63.5422

H -22.8219 22.8513 -62.5762

H -22.9319 23.0833 -64.3202

C -23.5579 21.1383 -63.6502

H -23.2999 20.4323 -62.8572

H -23.3749 20.6453 -64.6072

C -25.0339 21.4993 -63.5452

H -25.3109 22.2003 -64.3292

H -25.6539 20.6113 -63.6452

H -25.2469 21.9613 -62.5832

O -11.2249 24.4623 -68.2162

O -8.9049 24.0293 -69.2842

O -11.5019 24.9583 -65.5142

O -5.5379 25.3153 -65.8542

C 1.9451 24.3933 -64.1522

C 1.9611 23.6703 -62.9722

H 1.0271 23.3093 -62.5792

C 3.1621 23.4603 -62.3042

C 4.3621 23.9503 -62.8452

C 4.3401 24.6383 -64.0682

C 3.1231 24.8733 -64.6972

H 3.0801 25.4293 -65.6192

C 2.0871 22.4233 -60.4572

H 1.5591 21.6563 -61.0422

H 1.4271 23.2963 -60.3482

C 2.4291 21.8653 -59.0772

H 3.0041 20.9473 -59.2032

H 1.4801 21.6133 -58.5962

C 3.2121 22.8503 -58.2022

H 2.8751 23.8683 -58.4102

H 3.0041 22.6443 -57.1482

C 4.7181 22.7523 -58.4392

H 4.9181 22.8413 -59.5062

H 5.0581 21.7643 -58.1182

C 5.5151 23.8123 -57.6842

H 5.2831 24.8003 -58.0902

H 5.2241 23.8173 -56.6292

C 7.0141 23.5473 -57.8052

H 7.2581 23.3853 -58.8562

H 7.2581 22.6303 -57.2632

C 7.8721 24.6863 -57.2632

H 7.6711 25.5953 -57.8342

H 7.6001 24.8883 -56.2242

C 9.3601 24.3523 -57.3472

H 9.6121 24.1033 -58.3812

H 9.5661 23.4713 -56.7352

C 10.2441 25.5043 -56.8802

H 10.0671 26.3773 -57.5112

H 9.9681 25.7813 -55.8592

C 11.7261 25.1383 -56.9242

H 11.9031 24.2703 -56.2852

H 11.9971 24.8583 -57.9452

C 12.6191 26.2883 -56.4662

H 12.3311 26.5873 -55.4552

H 12.4621 27.1503 -57.1182

C 14.0931 25.9013 -56.4822

H 14.2731 25.0573 -55.8192

H 14.7131 26.7323 -56.1562

H 14.4001 25.6183 -57.4862

C 6.2221 24.8693 -61.6982

H 5.8691 25.1013 -60.6832

H 6.0151 25.7313 -62.3402

C 7.7161 24.5753 -61.6912

H 8.0071 24.2943 -62.7042

H 7.8981 23.7183 -61.0412

C 8.5581 25.7583 -61.2192

H 8.3661 26.6263 -61.8542

H 8.2761 26.0253 -60.1992

C 10.0451 25.4123 -61.2642

H 10.3141 25.1313 -62.2852

H 10.2271 24.5423 -60.6272

C 10.9451 26.5553 -60.8052

H 10.7811 27.4303 -61.4382

H 10.6861 26.8343 -59.7822

C 12.4171 26.1533 -60.8632

H 12.6711 25.8723 -61.8882

H 12.5711 25.2723 -60.2352

C 13.3551 27.2633 -60.4002

H 13.2251 28.1423 -61.0362

H 13.0991 27.5533 -59.3792

C 14.8141 26.8163 -60.4452

H 15.0671 26.5243 -61.4682

H 14.9391 25.9333 -59.8132

C 15.7771 27.9043 -59.9812

H 15.6671 28.7823 -60.6232

H 15.5211 28.2053 -58.9632

C 17.2281 27.4293 -60.0192

H 17.4661 27.0803 -61.0272

H 17.3481 26.5803 -59.3412

C 18.2111 28.5283 -59.6262

H 18.0741 29.3883 -60.2852

H 17.9971 28.8553 -58.6062

C 19.6551 28.0503 -59.7142

H 19.8161 27.2003 -59.0542

H 20.3411 28.8433 -59.4272

H 19.8921 27.7423 -60.7312

C 5.5451 25.7703 -65.7852

H 5.0481 26.7403 -65.6452

H 5.0071 25.2083 -66.5622

C 6.9851 25.9783 -66.2362

H 6.9621 26.5153 -67.1862

H 7.4401 25.0023 -66.4102

C 7.8281 26.7533 -65.2272

H 7.8991 26.1753 -64.3052

H 7.3391 27.7013 -64.9902

C 9.2241 27.0193 -65.7812

H 9.6561 26.0763 -66.1242

H 9.1461 27.6763 -66.6502

C 10.1651 27.6483 -64.7572

H 9.7401 28.5853 -64.3922

H 10.2691 26.9763 -63.9032

C 11.5421 27.9113 -65.3632

H 11.9181 26.9843 -65.8022

H 11.4471 28.6413 -66.1712

C 12.5521 28.4183 -64.3392

H 12.6471 27.6843 -63.5352

H 12.1901 29.3483 -63.8952

C 13.9241 28.6523 -64.9672

H 14.2491 27.7373 -65.4692

H 13.8481 29.4323 -65.7292

C 14.9731 29.0513 -63.9332

H 14.6731 29.9843 -63.4502

H 15.0221 28.2823 -63.1592

C 16.3571 29.2243 -64.5542

H 16.3291 30.0313 -65.2902

H 16.6331 28.3083 -65.0832

C 17.4201 29.5313 -63.5032

H 17.1571 30.4553 -62.9822

H 17.4331 28.7273 -62.7652

C 18.8081 29.6723 -64.1162

H 19.0941 28.7533 -64.6242

H 19.5471 29.8833 -63.3462

H 18.8261 30.4823 -64.8412

O 5.5351 23.7133 -62.1712

O 3.2721 22.8043 -61.1222

O 5.5391 25.0493 -64.5692

O -0.3519 25.0243 -63.9512

C -2.1299 25.2193 -59.5452

C -0.8739 25.0373 -58.9552

C -3.2639 25.3083 -58.7282

C -0.7559 24.9283 -57.5872

H -0.0009 24.9853 -59.5892

C -3.1439 25.1953 -57.3632

H -4.2319 25.4563 -59.1832

C -1.8909 25.0003 -56.7782

H 0.2121 24.7853 -57.1312

H -4.0119 25.2483 -56.7232

C -3.2679 25.3683 -70.2722

C -2.0039 25.2083 -70.8522

C -4.3889 25.4823 -71.0992

C -1.8719 25.1253 -72.2192

H -1.1379 25.1403 -70.2112

C -4.2559 25.3853 -72.4672

H -5.3599 25.6423 -70.6572

C -2.9979 25.1923 -73.0392

H -0.9039 24.9963 -72.6782

H -5.1199 25.4583 -73.1072

C -6.5939 24.9023 -65.0102

H -6.3179 23.9553 -64.5202

H -6.7839 25.6543 -64.2322

C 0.6521 24.6463 -64.8732

H 0.3341 23.7313 -65.3972

H 0.8051 25.4403 -65.6162

C -1.6889 23.2553 2.7198

C -1.6769 22.8783 -2.9192

C -1.6689 22.7873 -4.1242

C -1.7579 22.7263 -5.5242

C -0.5989 22.7743 -6.3252

C -3.0219 22.6323 -6.1072

H -3.8639 22.5683 -5.4382

O -2.7309 23.3483 3.3548

O -0.5049 23.2283 3.2868

H -0.5809 23.2953 4.2908

C -2.0529 23.0103 -16.5452

C -2.0639 22.8413 -10.8942

C -2.1109 22.7873 -9.6862

C -2.0319 22.7373 -8.2852

C -3.1869 22.6293 -7.4832

C -0.7639 22.7913 -7.6992

H 0.0811 22.8573 -8.3662

O -1.0119 23.0743 -17.1942

O -3.2399 23.0003 -17.1042

H -3.1999 23.0723 -18.1052

C -6.7769 22.3573 -8.1622

C -6.7519 22.2003 -9.5362

H -5.8139 21.9683 -10.0112

C -7.9169 22.3873 -10.2732

C -9.1149 22.7133 -9.6212

C -9.1429 22.8193 -8.2232

C -7.9659 22.6403 -7.5062

H -7.9599 22.7403 -6.4322

C -6.9609 21.7233 -12.3532

H -6.0009 22.2083 -12.1352

H -6.8889 20.6643 -12.0682

C -7.2539 21.8453 -13.8472

H -7.3249 22.9033 -14.1102

H -6.3949 21.4233 -14.3752

C -8.5379 21.1253 -14.2662

H -8.6409 20.2133 -13.6762

H -8.4589 20.8413 -15.3202

C -9.7919 21.9793 -14.0812

H -9.8049 22.3793 -13.0682

H -9.7639 22.8223 -14.7782

C -11.0529 21.1543 -14.3202

H -11.0599 20.3283 -13.6052

H -11.0239 20.7183 -15.3232

C -12.3339 21.9703 -14.1662

H -12.3229 22.4813 -13.2032

H -12.3729 22.7373 -14.9452

C -13.5749 21.0863 -14.2612

H -13.5469 20.3473 -13.4562

H -13.5529 20.5313 -15.2022

C -14.8829 21.8683 -14.1722

H -14.9249 22.3973 -13.2182

H -14.9169 22.6173 -14.9662

C -16.0919 20.9433 -14.2932

H -16.0379 20.1833 -13.5092

H -16.0499 20.4193 -15.2522

C -17.4219 21.6833 -14.1812

H -17.4909 22.4273 -14.9772

H -17.4609 22.2123 -13.2272

C -18.6089 20.7273 -14.2772

H -18.5669 20.1963 -15.2312

H -18.5309 19.9803 -13.4842

C -19.9439 21.4493 -14.1572

H -20.0469 22.1933 -14.9432

H -20.7689 20.7453 -14.2432

H -20.0199 21.9533 -13.1972

C -11.3179 22.1003 -10.2562

H -11.3359 21.4483 -11.1382

H -11.1939 21.4683 -9.3732

C -12.6129 22.9033 -10.1592

H -12.5799 23.5113 -9.2542

H -12.6649 23.5823 -11.0122

C -13.8309 21.9883 -10.1442

H -13.7589 21.3103 -9.2892

H -13.8289 21.3743 -11.0472

C -15.1499 22.7533 -10.0632

H -15.1699 23.3523 -9.1502

H -15.2239 23.4403 -10.9092

C -16.3429 21.8013 -10.0762

H -16.2729 21.1303 -9.2162

H -16.2999 21.1853 -10.9772

C -17.6849 22.5263 -10.0292

H -17.7409 23.1343 -9.1242

H -17.7629 23.2033 -10.8842

C -18.8529 21.5423 -10.0542

H -18.7859 20.8883 -9.1812

H -18.7739 20.9143 -10.9442

C -20.2099 22.2403 -10.0512

H -20.2999 22.8563 -9.1542

H -20.2769 22.9063 -10.9152

C -21.3609 21.2383 -10.0952

H -21.3149 20.6023 -9.2072

H -21.2449 20.5933 -10.9692

C -22.7239 21.9213 -10.1532

H -22.8379 22.5783 -9.2872

H -22.7769 22.5453 -11.0482

C -23.8699 20.9123 -10.1752

H -23.8349 20.3093 -9.2642

H -23.7389 20.2413 -11.0262

C -25.2299 21.5923 -10.2732

H -25.2949 22.1853 -11.1832

H -26.0269 20.8523 -10.2882

H -25.3909 22.2523 -9.4232

C -10.3949 23.4773 -6.2942

H -10.2029 22.5993 -5.6602

H -9.6309 24.2383 -6.0822

C -11.7799 24.0483 -6.0012

H -11.7679 24.4983 -5.0062

H -11.9829 24.8363 -6.7292

C -12.8749 22.9883 -6.0812

H -12.8049 22.4813 -7.0432

H -12.7149 22.2423 -5.2982

C -14.2689 23.5903 -5.9292

H -14.4359 24.3143 -6.7302

H -14.3339 24.1283 -4.9812

C -15.3579 22.5213 -5.9832

H -15.2309 21.8333 -5.1432

H -15.2499 21.9413 -6.9022

C -16.7609 23.1213 -5.9312

H -16.8919 23.8003 -6.7772

H -16.8709 23.7093 -5.0182

C -17.8449 22.0473 -5.9762

H -17.7109 21.4363 -6.8712

H -17.7389 21.3903 -5.1092

C -19.2489 22.6443 -5.9802

H -19.3609 23.2933 -6.8522

H -19.3819 23.2633 -5.0902

C -20.3289 21.5673 -6.0102

H -20.2279 20.9293 -5.1282

H -20.1849 20.9383 -6.8912

C -21.7349 22.1603 -6.0402

H -21.8879 22.7773 -5.1512

H -21.8319 22.8113 -6.9132

C -22.8129 21.0813 -6.1002

H -22.7219 20.4323 -5.2252

H -22.6529 20.4653 -6.9872

C -24.2139 21.6753 -6.1462

H -24.3259 22.3193 -7.0152

H -24.9639 20.8893 -6.2042

H -24.4079 22.2693 -5.2552

O -10.2129 22.9853 -10.3922

O -8.0019 22.3313 -11.6252

O -10.3479 23.1153 -7.6592

O -4.3759 22.5273 -8.1232

C 2.9881 22.8123 -5.5932

C 3.0091 23.4983 -4.3892

H 2.1141 24.0103 -4.0752

C 4.1751 23.5263 -3.6302

C 5.3251 22.8823 -4.1032

C 5.2991 22.2073 -5.3302

C 4.1181 22.1583 -6.0582

H 4.0751 21.6223 -6.9932

C 3.2391 24.8733 -1.8932

H 2.3351 24.2503 -1.8912

H 3.0431 25.7683 -2.5012

C 3.5711 25.2683 -0.4562

H 3.7771 24.3603 0.1118

H 2.6761 25.7343 -0.0412

C 4.7621 26.2253 -0.3342

H 4.7971 26.8713 -1.2132

H 4.6151 26.8693 0.5368

C 6.0971 25.4983 -0.1832

H 6.2231 24.7973 -1.0072

H 6.0841 24.9183 0.7428

C 7.2681 26.4783 -0.1422

H 7.3501 26.9853 -1.1072

H 7.0671 27.2463 0.6108

C 8.5941 25.7963 0.1838

H 8.7931 25.0183 -0.5532

H 8.5171 25.3113 1.1598

C 9.7561 26.7873 0.2038

H 9.8741 27.2243 -0.7912

H 9.5241 27.6043 0.8908

C 11.0721 26.1383 0.6288

H 11.3131 25.3263 -0.0592

H 10.9511 25.7063 1.6238

C 12.2231 27.1413 0.6508

H 12.3401 27.5773 -0.3442

H 11.9811 27.9543 1.3388

C 13.5431 26.5023 1.0768

H 13.4261 26.0643 2.0708

H 13.7931 25.6943 0.3868

C 14.6881 27.5113 1.1028

H 14.4421 28.3203 1.7948

H 14.8031 27.9523 0.1098

C 16.0021 26.8683 1.5258

H 15.9071 26.4323 2.5178

H 16.8021 27.6053 1.5488

H 16.2831 26.0793 0.8318

C 7.3611 23.9913 -3.7542

H 7.0281 24.9263 -3.2792

H 7.3311 24.1333 -4.8422

C 8.7751 23.6413 -3.3082

H 9.1071 22.7603 -3.8582

H 8.7391 23.3753 -2.2502

C 9.7551 24.7913 -3.5152

H 9.7561 25.0903 -4.5662

H 9.4331 25.6543 -2.9282

C 11.1681 24.3923 -3.0952

H 11.5151 23.5683 -3.7232

H 11.1421 24.0283 -2.0652

C 12.1581 25.5493 -3.1852

H 12.1591 25.9513 -4.2022

H 11.8371 26.3513 -2.5162

C 13.5701 25.1093 -2.8092

H 13.9071 24.3373 -3.5042

H 13.5521 24.6663 -1.8102

C 14.5631 26.2673 -2.8202

H 14.5521 26.7463 -3.8032

H 14.2531 27.0163 -2.0892

C 15.9801 25.8003 -2.5002

H 16.2971 25.0743 -3.2522

H 15.9811 25.2933 -1.5322

C 16.9771 26.9543 -2.4652

H 16.9331 27.4973 -3.4132

H 16.6991 27.6543 -1.6742

C 18.4041 26.4653 -2.2312

H 18.6641 25.7383 -3.0042

H 18.4581 25.9573 -1.2652

C 19.4211 27.6033 -2.2582

H 19.3561 28.1213 -3.2182

H 19.1771 28.3253 -1.4752

C 20.8421 27.0933 -2.0552

H 20.9321 26.5833 -1.0982

H 21.5521 27.9163 -2.0732

H 21.1101 26.3923 -2.8422

C 6.6011 21.1393 -7.0272

H 6.2551 21.8743 -7.7672

H 5.9981 20.2263 -7.1322

C 8.0791 20.8323 -7.2452

H 8.2021 20.3863 -8.2312

H 8.3951 20.1053 -6.4962

C 8.9351 22.0903 -7.1242

H 8.6861 22.5863 -6.1852

H 8.6951 22.7743 -7.9402

C 10.4291 21.7813 -7.1402

H 10.6631 21.0893 -6.3282

H 10.6931 21.2873 -8.0782

C 11.2611 23.0503 -6.9752

H 11.0671 23.7193 -7.8162

H 10.9471 23.5663 -6.0662

C 12.7561 22.7623 -6.8942

H 12.9531 22.1143 -6.0372

H 13.0731 22.2233 -7.7902

C 13.5781 24.0413 -6.7572

H 13.2421 24.5923 -5.8772

H 13.4021 24.6773 -7.6282

C 15.0711 23.7543 -6.6332

H 15.2441 23.1543 -5.7362

H 15.3991 23.1623 -7.4902

C 15.9061 25.0293 -6.5522

H 15.7681 25.6133 -7.4652

H 15.5571 25.6373 -5.7152

C 17.3891 24.7223 -6.3682

H 17.7371 24.1073 -7.2012

H 17.5221 24.1423 -5.4522

C 18.2431 25.9843 -6.2842

H 18.1211 26.5653 -7.2012

H 17.8951 26.6003 -5.4532

C 19.7181 25.6553 -6.0862

H 19.8631 25.0833 -5.1722

H 20.3101 26.5653 -6.0172

H 20.0931 25.0643 -6.9192

O 6.4951 22.9303 -3.3732

O 4.3271 24.1593 -2.4382

O 6.4761 21.6643 -5.7222

O 0.5881 22.8113 -5.6752

C -1.6739 22.9733 -1.5182

C -0.4639 23.0603 -0.8222

C -2.8819 22.9843 -0.8112

C -0.4609 23.1473 0.5508

H 0.4601 23.0563 -1.3792

C -2.8729 23.0743 0.5598

H -3.8119 22.9213 -1.3542

C -1.6659 23.1553 1.2518

H 0.4681 23.2133 1.0948

H -3.7899 23.0833 1.1288

C -2.0559 22.8843 -12.2982

C -0.8609 22.7353 -13.0092

C -3.2519 23.0733 -12.9962

C -0.8659 22.7783 -14.3842

H 0.0621 22.5943 -12.4672

C -3.2589 23.0933 -14.3702

H -4.1659 23.2083 -12.4382

C -2.0639 22.9523 -15.0772

H 0.0481 22.6683 -14.9452

H -4.1839 23.2233 -14.9102

C -5.5199 22.2503 -7.3382

H -5.4269 21.2383 -6.9132

H -5.5959 22.9693 -6.5122

C 1.7591 22.8273 -6.4622

H 1.7741 21.9553 -7.1312

H 1.7741 23.7413 -7.0782

C -3.1439 19.0143 -55.0662

C -3.4529 19.3943 -60.7092

C -3.5759 19.3933 -61.9132

C -3.7779 19.3853 -63.3042

C -2.6919 19.2673 -64.1962

C -5.0819 19.4473 -63.8012

H -5.8859 19.4983 -63.0832

O -4.1589 19.0163 -54.3802

O -1.9379 18.9043 -54.5512

H -1.9599 18.8403 -53.5522

C -3.6579 19.0733 -74.2762

C -4.3369 19.4553 -68.6612

C -4.3949 19.4413 -67.4522

C -4.2559 19.4073 -66.0522

C -5.3479 19.4253 -65.1622

C -2.9539 19.3133 -65.5552

H -2.1589 19.2583 -66.2802

O -2.5489 19.0063 -74.7872

O -4.7729 19.0263 -74.9672

H -4.5969 18.9553 -75.9552

C -8.8929 18.8273 -65.7472

C -8.7529 18.4803 -67.0792

H -7.7629 18.3093 -67.4642

C -9.8789 18.3853 -67.8872

C -11.1569 18.6003 -67.3472

C -11.2909 18.9043 -65.9852

C -10.1499 19.0183 -65.1972

H -10.2309 19.2733 -64.1522

C -8.6569 17.8103 -69.8602

H -7.8669 18.5133 -69.5662

H -8.3409 16.7963 -69.5712

C -8.8669 17.8893 -71.3722

H -9.0859 18.9223 -71.6452

H -7.9249 17.6063 -71.8462

C -10.0039 16.9873 -71.8612

H -10.0329 16.0883 -71.2452

H -9.8049 16.6833 -72.8932

C -11.3689 17.6683 -71.7932

H -11.5049 18.0903 -70.7982

H -11.4019 18.4873 -72.5172

C -12.5059 16.6903 -72.0742

H -12.4739 15.8873 -71.3342

H -12.3739 16.2403 -73.0632

C -13.8669 17.3763 -72.0032

H -13.9429 17.9033 -71.0512

H -13.9469 18.1223 -72.8002

C -15.0139 16.3773 -72.1172

H -14.9119 15.6443 -71.3142

H -14.9379 15.8383 -73.0642

C -16.3829 17.0473 -72.0252

H -16.4489 17.6043 -71.0892

H -16.4909 17.7673 -72.8412

C -17.5199 16.0323 -72.0902

H -17.4119 15.3263 -71.2622

H -17.4439 15.4613 -73.0182

C -18.8969 16.6863 -72.0122

H -19.0139 17.3873 -72.8432

H -18.9729 17.2613 -71.0862

C -20.0219 15.6553 -72.0602

H -19.9379 15.0733 -72.9792

H -19.9069 14.9633 -71.2212

C -21.3999 16.3013 -71.9932

H -21.5379 16.9953 -72.8202

H -22.1809 15.5463 -72.0462

H -21.5179 16.8533 -71.0642

C -13.2619 17.6493 -67.9582

H -13.1889 16.8433 -68.7002

H -13.1529 17.2073 -66.9652

C -14.6139 18.3483 -68.0782

H -14.7149 19.0663 -67.2622

H -14.6369 18.9073 -69.0152

C -15.7449 17.3283 -68.0452

H -15.6389 16.7133 -67.1482

H -15.6429 16.6623 -68.9032

C -17.1329 17.9633 -68.0582

H -17.2459 18.6193 -67.1922

H -17.2459 18.5813 -68.9522

C -18.2209 16.8923 -68.0382

H -18.0719 16.2513 -67.1642

H -18.1159 16.2623 -68.9232

C -19.6349 17.4653 -67.9932

H -19.7559 18.0743 -67.0942

H -19.7929 18.1183 -68.8552

C -20.6799 16.3523 -68.0012

H -20.5099 15.6973 -67.1412

H -20.5499 15.7473 -68.9002

C -22.1119 16.8753 -67.9502

H -22.2609 17.4413 -67.0282

H -22.2809 17.5583 -68.7862

C -23.1249 15.7353 -68.0172

H -22.9649 15.0643 -67.1692

H -22.9549 15.1583 -68.9302

C -24.5689 16.2283 -67.9972

H -24.7439 16.8013 -67.0842

H -24.7329 16.8983 -68.8452

C -25.5659 15.0743 -68.0682

H -25.4179 14.4193 -67.2052

H -25.3709 14.4873 -68.9682

C -27.0089 15.5613 -68.0832

H -27.1859 16.2033 -68.9432

H -27.6969 14.7203 -68.1372

H -27.2319 16.1273 -67.1822

C -12.7449 19.5503 -64.2022

H -12.4519 18.7683 -63.4862

H -12.1279 20.4413 -64.0202

C -14.2169 19.9073 -64.0162

H -14.3329 20.3823 -63.0402

H -14.4929 20.6313 -64.7842

C -15.1309 18.6893 -64.1132

H -14.9899 18.2183 -65.0862

H -14.8469 17.9633 -63.3462

C -16.5969 19.0733 -63.9312

H -16.8659 19.8303 -64.6722

H -16.7319 19.5203 -62.9422

C -17.5309 17.8753 -64.0762

H -17.2479 17.1053 -63.3552

H -17.4169 17.4483 -65.0732

C -18.9929 18.2553 -63.8602

H -19.2809 19.0143 -64.5922

H -19.1109 18.6983 -62.8682

C -19.9199 17.0493 -63.9912

H -19.7739 16.5893 -64.9692

H -19.6539 16.3073 -63.2352

C -21.3889 17.4283 -63.8312

H -21.6599 18.1553 -64.6002

H -21.5379 17.9093 -62.8612

C -22.3049 16.2123 -63.9402

H -22.0569 15.5023 -63.1472

H -22.1259 15.7143 -64.8962

C -23.7809 16.5823 -63.8372

H -23.9689 17.0743 -62.8802

H -24.0299 17.2953 -64.6272

C -24.6859 15.3593 -63.9622

H -24.4529 14.6563 -63.1572

H -24.4799 14.8573 -64.9092

C -26.1619 15.7293 -63.8982

H -26.4129 16.4263 -64.6942

H -26.7849 14.8443 -64.0092

H -26.3979 16.2003 -62.9462

O -12.2139 18.5773 -68.2152

O -9.8689 18.1243 -69.2162

O -12.5609 19.0943 -65.5252

O -6.5879 19.4143 -65.7062

C 0.8411 18.4593 -63.7962

C 0.8201 17.7453 -62.6122

H -0.1259 17.3923 -62.2412

C 2.0011 17.5323 -61.9102

C 3.2191 18.0113 -62.4222

C 3.2341 18.6903 -63.6502

C 2.0361 18.9283 -64.3132

H 2.0211 19.4783 -65.2392

C 0.8711 16.5143 -60.0852

H 0.3541 15.7463 -60.6802

H 0.2141 17.3923 -60.0002

C 1.1721 15.9643 -58.6932

H 1.7451 15.0423 -58.7972

H 0.2091 15.7203 -58.2362

C 1.9381 16.9503 -57.8042

H 1.6121 17.9683 -58.0282

H 1.7001 16.7533 -56.7542

C 3.4481 16.8413 -58.0002

H 3.6781 16.9223 -59.0622

H 3.7741 15.8533 -57.6632

C 4.2311 17.9023 -57.2302

H 4.0171 18.8883 -57.6492

H 3.9121 17.9163 -56.1842

C 5.7311 17.6273 -57.3092

H 6.0031 17.4573 -58.3522

H 5.9551 16.7123 -56.7542

C 6.5821 18.7653 -56.7522

H 6.4011 19.6713 -57.3352

H 6.2831 18.9763 -55.7222

C 8.0691 18.4213 -56.7942

H 8.3471 18.1643 -57.8182

H 8.2531 17.5433 -56.1702

C 8.9481 19.5713 -56.3102

H 8.7921 20.4413 -56.9532

H 8.6461 19.8573 -55.2992

C 10.4281 19.1963 -56.3122

H 10.5821 18.3323 -55.6622

H 10.7241 18.9083 -57.3232

C 11.3151 20.3443 -55.8382

H 11.0021 20.6523 -54.8372

H 11.1801 21.2033 -56.4992

C 12.7861 19.9483 -55.8112

H 12.9431 19.1083 -55.1382

H 13.4031 20.7783 -55.4742

H 13.1191 19.6563 -56.8052

C 5.0531 18.9273 -61.2312

H 4.6741 19.1683 -60.2272

H 4.8681 19.7853 -61.8842

C 6.5451 18.6243 -61.1812

H 6.8611 18.3353 -62.1852

H 6.7031 17.7703 -60.5212

C 7.3801 19.8053 -60.6952

H 7.2111 20.6703 -61.3412

H 7.0721 20.0813 -59.6852

C 8.8651 19.4503 -60.6972

H 9.1611 19.1613 -61.7092

H 9.0251 18.5843 -60.0502

C 9.7601 20.5913 -60.2222

H 9.6191 21.4633 -60.8662

H 9.4761 20.8793 -59.2082

C 11.2301 20.1803 -60.2382

H 11.5101 19.8903 -61.2542

H 11.3631 19.3023 -59.6002

C 12.1631 21.2883 -59.7572

H 12.0551 22.1633 -60.4022

H 11.8811 21.5863 -58.7462

C 13.6201 20.8313 -59.7602

H 13.8981 20.5313 -60.7732

H 13.7221 19.9523 -59.1182

C 14.5771 21.9173 -59.2782

H 14.4891 22.7913 -59.9282

H 14.2951 22.2263 -58.2682

C 16.0251 21.4333 -59.2732

H 16.2881 21.0753 -60.2722

H 16.1211 20.5883 -58.5872

C 17.0031 22.5293 -58.8612

H 16.8891 23.3863 -59.5292

H 16.7641 22.8643 -57.8492

C 18.4461 22.0413 -58.9072

H 18.5851 21.1953 -58.2372

H 19.1301 22.8323 -58.6062

H 18.7091 21.7263 -59.9142

C 4.4921 19.8033 -65.3412

H 3.9971 20.7783 -65.2212

H 3.9721 19.2393 -66.1282

C 5.9451 20.0003 -65.7542

H 5.9501 20.5303 -66.7082

H 6.3981 19.0193 -65.9092

C 6.7651 20.7763 -64.7282

H 6.8071 20.2043 -63.8002

H 6.2761 21.7293 -64.5112

C 8.1771 21.0303 -65.2462

H 8.6131 20.0823 -65.5702

H 8.1261 21.6823 -66.1212

C 9.0941 21.6613 -64.2022

H 8.6641 22.6033 -63.8542

H 9.1701 20.9943 -63.3402

C 10.4881 21.9123 -64.7722

H 10.8701 20.9803 -65.1942

H 10.4191 22.6373 -65.5862

C 11.4741 22.4203 -63.7242

H 11.5421 21.6913 -62.9132

H 11.1061 23.3553 -63.2962

C 12.8631 22.6413 -64.3172

H 13.1951 21.7213 -64.8042

H 12.8131 23.4163 -65.0862

C 13.8871 23.0413 -63.2582

H 13.5791 23.9793 -62.7892

H 13.9101 22.2773 -62.4772

C 15.2881 23.2023 -63.8422

H 15.2841 24.0043 -64.5842

H 15.5721 22.2803 -64.3562

C 16.3241 23.5103 -62.7642

H 16.0531 24.4393 -62.2572

H 16.3121 22.7113 -62.0212

C 17.7281 23.6383 -63.3412

H 18.0231 22.7143 -63.8352

H 18.4481 23.8503 -62.5532

H 17.7711 24.4433 -64.0712

O 4.3721 17.7723 -61.7142

O 2.0751 16.8833 -60.7212

O 4.4491 19.0913 -64.1202

O -1.4569 19.1053 -63.6622

C -3.3519 19.3413 -59.3062

C -2.1139 19.1563 -58.6822

C -4.5069 19.4433 -58.5212

C -2.0329 19.0563 -57.3112

H -1.2239 19.0953 -59.2912

C -4.4239 19.3393 -57.1532

H -5.4609 19.5933 -59.0042

C -3.1889 19.1403 -56.5332

H -1.0789 18.9113 -56.8272

H -5.3089 19.4013 -56.5372

C -4.1999 19.4223 -70.0612

C -2.9209 19.2513 -70.6062

C -5.2969 19.5373 -70.9192

C -2.7529 19.1583 -71.9692

H -2.0729 19.1823 -69.9412

C -5.1269 19.4303 -72.2822

H -6.2789 19.7063 -70.5042

C -3.8559 19.2253 -72.8192

H -1.7739 19.0203 -72.4002

H -5.9739 19.5043 -72.9462

C -7.6689 19.0123 -64.8882

H -7.4119 18.0673 -64.3842

H -7.8749 19.7713 -64.1202

C -0.4309 18.7143 -64.5542

H -0.7399 17.7983 -65.0802

H -0.2529 19.5033 -65.2982

C -1.1269 16.7793 1.7928

C -0.9789 16.3543 -3.8512

C -1.0179 16.3593 -5.0602

C -1.0819 16.3923 -6.4642

C 0.0991 16.5173 -7.2252

C -2.3239 16.3533 -7.0992

H -3.1999 16.2853 -6.4742

O -2.1719 16.6123 2.4078

O -0.0019 17.1263 2.3738

H -0.1039 17.1843 3.3728

C -1.9029 16.6353 -17.4702

C -1.3249 16.3243 -11.8522

C -1.3049 16.3773 -10.6442

C -1.2429 16.4203 -9.2412

C -2.4299 16.4253 -8.4792

C -0.0009 16.4843 -8.6052

H 0.8791 16.5113 -9.2292

O -0.9639 16.8683 -18.2232

O -3.1449 16.5253 -17.8872

H -3.2339 16.6503 -18.8792

C -5.8689 17.3983 -9.2582

C -5.8659 17.3293 -10.6392

H -5.0019 16.9213 -11.1352

C -6.9789 17.7713 -11.3532

C -8.1019 18.2533 -10.6682

C -8.0739 18.3703 -9.2712

C -6.9479 17.9503 -8.5782

H -6.9019 18.0273 -7.5042

C -6.0709 17.2123 -13.4972

H -5.1169 17.7213 -13.2972

H -5.9529 16.1453 -13.2602

C -6.4299 17.3863 -14.9722

H -6.6189 18.4433 -15.1602

H -5.5539 17.0853 -15.5502

C -7.6459 16.5693 -15.4202

H -7.6219 15.5913 -14.9332

H -7.5799 16.4003 -16.4982

C -8.9709 17.2623 -15.1112

H -8.9899 17.5523 -14.0612

H -9.0379 18.1763 -15.7052

C -10.1739 16.3733 -15.4202

H -10.1309 15.4773 -14.7942

H -10.1269 16.0453 -16.4622

C -11.4959 17.0963 -15.1752

H -11.5059 17.4903 -14.1582

H -11.5719 17.9493 -15.8532

C -12.7049 16.1833 -15.3742

H -12.6669 15.3693 -14.6462

H -12.6569 15.7323 -16.3692

C -14.0249 16.9363 -15.2262

H -14.0559 17.4273 -14.2522

H -14.0789 17.7183 -15.9862

C -15.2369 16.0173 -15.3692

H -15.2489 15.2993 -14.5442

H -15.1479 15.4433 -16.2952

C -16.5479 16.7993 -15.3862

H -16.5349 17.4963 -16.2262

H -16.6309 17.3873 -14.4702

C -17.7669 15.8893 -15.5142

H -17.6529 15.2553 -16.3982

H -17.8209 15.2273 -14.6462

C -19.0599 16.6863 -15.6292

H -19.0329 17.3243 -16.5102

H -19.9159 16.0213 -15.7152

H -19.1989 17.3163 -14.7542

C -10.2789 17.6193 -11.2402

H -10.1959 16.8943 -12.0652

H -10.1719 17.0683 -10.2992

C -11.6349 18.3103 -11.2802

H -11.7069 18.9853 -10.4272

H -11.6889 18.9153 -12.1872

C -12.7839 17.3063 -11.2572

H -12.7089 16.6873 -10.3592

H -12.7029 16.6413 -12.1192

C -14.1409 18.0043 -11.2812

H -14.2429 18.6323 -10.3952

H -14.1899 18.6603 -12.1532

C -15.3009 17.0133 -11.3382

H -15.2599 16.3553 -10.4672

H -15.1979 16.3863 -12.2272

C -16.6519 17.7223 -11.3772

H -16.7689 18.3293 -10.4772

H -16.6739 18.4013 -12.2332

C -17.8159 16.7423 -11.4852

H -17.7999 16.0663 -10.6262

H -17.6919 16.1333 -12.3832

C -19.1639 17.4553 -11.5412

H -19.2929 18.0553 -10.6392

H -19.1759 18.1383 -12.3942

C -20.3269 16.4763 -11.6682

H -20.3219 15.7983 -10.8112

H -20.1949 15.8703 -12.5682

C -21.6739 17.1923 -11.7352

H -21.7929 17.8163 -10.8462

H -21.6879 17.8513 -12.6072

C -22.8429 16.2163 -11.8242

H -22.8339 15.5623 -10.9492

H -22.7199 15.5863 -12.7092

C -24.1839 16.9373 -11.8992

H -24.2209 17.5863 -12.7722

H -24.9999 16.2223 -11.9702

H -24.3399 17.5473 -11.0122

C -9.2569 18.9933 -7.2922

H -9.0299 18.0323 -6.8092

H -8.5229 19.7403 -6.9582

C -10.6719 19.4333 -6.9352

H -10.7009 19.7113 -5.8812

H -10.9109 20.3163 -7.5272

C -11.6949 18.3333 -7.2172

H -11.5229 17.9433 -8.2212

H -11.5509 17.5123 -6.5102

C -13.1249 18.8523 -7.1162

H -13.2719 19.6313 -7.8682

H -13.2769 19.3113 -6.1382

C -14.1679 17.7573 -7.3252

H -14.0789 17.0113 -6.5312

H -13.9789 17.2503 -8.2742

C -15.5819 18.3313 -7.3292

H -15.6749 19.0373 -8.1582

H -15.7449 18.8883 -6.4052

C -16.6599 17.2603 -7.4662

H -16.4969 16.6923 -8.3842

H -16.5869 16.5613 -6.6302

C -18.0569 17.8753 -7.4922

H -18.1419 18.5303 -8.3632

H -18.1939 18.4933 -6.6022

C -19.1609 16.8243 -7.5482

H -19.0939 16.1813 -6.6662

H -19.0169 16.1943 -8.4282

C -20.5479 17.4603 -7.6042

H -20.6939 18.0863 -6.7212

H -20.6089 18.1063 -8.4832

C -21.6619 16.4193 -7.6752

H -21.6129 15.7763 -6.7932

H -21.5099 15.7883 -8.5532

C -23.0389 17.0673 -7.7532

H -23.1139 17.6963 -8.6372

H -23.8179 16.3103 -7.8082

H -23.2219 17.6853 -6.8772

O -9.2409 18.5843 -11.3682

O -7.0989 17.7673 -12.7072

O -9.1989 18.8623 -8.6982

O -3.6019 16.5213 -9.1632

C 3.4471 17.4683 -6.1862

C 3.1091 18.2253 -5.0752

H 2.1011 18.5963 -4.9892

C 4.0571 18.4653 -4.0872

C 5.3631 17.9703 -4.2352

C 5.7081 17.2523 -5.3882

C 4.7381 16.9933 -6.3502

H 4.9741 16.4063 -7.2242

C 2.5641 19.6943 -2.6802

H 1.7731 18.9733 -2.9212

H 2.4221 20.5883 -3.3052

C 2.4741 20.0583 -1.1992

H 2.5291 19.1433 -0.6072

H 1.4931 20.5083 -1.0402

C 3.5661 21.0253 -0.7392

H 3.8031 21.7093 -1.5532

H 3.1891 21.6163 0.1008

C 4.8481 20.3153 -0.3092

H 5.1671 19.6443 -1.1052

H 4.6481 19.7123 0.5808

C 5.9631 21.3143 -0.0152

H 6.1551 21.9003 -0.9162

H 5.6421 22.0043 0.7708

C 7.2541 20.6223 0.4128

H 7.5021 19.8583 -0.3242

H 7.1091 20.1223 1.3748

C 8.4071 21.6153 0.5098

H 8.4901 22.1303 -0.4492

H 8.1811 22.3733 1.2648

C 9.7431 20.9523 0.8368

H 9.9121 20.1253 0.1448

H 9.7111 20.5373 1.8468

C 10.8981 21.9433 0.7238

H 10.9371 22.3203 -0.3012

H 10.7111 22.7993 1.3768

C 12.2481 21.3243 1.0768

H 12.2331 20.9933 2.1178

H 12.4161 20.4453 0.4518

C 13.3931 22.3123 0.8748

H 13.2121 23.2043 1.4798

H 13.4131 22.6243 -0.1722

C 14.7431 21.7153 1.2478

H 14.7521 21.4203 2.2948

H 15.5411 22.4373 1.0888

H 14.9511 20.8363 0.6418

C 7.3151 19.0733 -3.4732

H 7.0641 20.0383 -3.0132

H 7.4191 19.2303 -4.5492

C 8.6271 18.5423 -2.9052

H 8.8881 17.6173 -3.4242

H 8.4901 18.3013 -1.8502

C 9.7271 19.5853 -3.0752

H 9.7531 19.9013 -4.1212

H 9.4741 20.4613 -2.4772

C 11.1131 19.0843 -2.6762

H 11.3701 18.2033 -3.2702

H 11.1101 18.7803 -1.6272

C 12.1641 20.1713 -2.8922

H 12.1091 20.5183 -3.9282

H 11.9301 21.0233 -2.2502

C 13.5881 19.7033 -2.6032

H 13.8341 18.8593 -3.2532

H 13.6561 19.3523 -1.5712

C 14.5961 20.8293 -2.8242

H 14.4921 21.2083 -3.8452

H 14.3651 21.6533 -2.1462

C 16.0391 20.3883 -2.6022

H 16.2931 19.5993 -3.3142

H 16.1411 19.9703 -1.5972

C 17.0131 21.5533 -2.7632

H 16.8971 21.9833 -3.7622

H 16.7611 22.3323 -2.0402

C 18.4671 21.1333 -2.5672

H 18.7261 20.3703 -3.3052

H 18.5831 20.6873 -1.5762

C 19.4251 22.3133 -2.7022

H 19.3141 22.7553 -3.6962

H 19.1591 23.0773 -1.9682

C 20.8771 21.9013 -2.5012

H 21.0171 21.4683 -1.5132

H 21.5371 22.7613 -2.5942

H 21.1711 21.1623 -3.2432

C 7.3741 15.9923 -6.5422

H 7.2221 16.5053 -7.5032

H 6.7631 15.0793 -6.5302

C 8.8481 15.6413 -6.3752

H 9.1181 14.9073 -7.1372

H 8.9911 15.1873 -5.3942

C 9.7301 16.8793 -6.5042

H 9.4581 17.5843 -5.7192

H 9.5341 17.3563 -7.4672

C 11.2171 16.5523 -6.4022

H 11.4141 16.0273 -5.4652

H 11.4971 15.8833 -7.2202

C 12.0681 17.8173 -6.4662

H 11.8031 18.3803 -7.3642

H 11.8391 18.4483 -5.6062

C 13.5631 17.5163 -6.4872

H 13.8311 16.9393 -5.5982

H 13.7971 16.9013 -7.3602

C 14.3951 18.7943 -6.5312

H 14.1681 19.4013 -5.6532

H 14.1131 19.3773 -7.4112

C 15.8941 18.5113 -6.5702

H 16.1781 17.9503 -5.6762

H 16.1251 17.8843 -7.4362

C 16.7131 19.7963 -6.6422

H 16.4641 20.3323 -7.5622

H 16.4421 20.4413 -5.8042

C 18.2151 19.5303 -6.6042

H 18.4961 18.8993 -7.4502

H 18.4601 18.9833 -5.6902

C 19.0241 20.8243 -6.6452

H 18.8001 21.3603 -7.5702

H 18.7191 21.4613 -5.8122

C 20.5221 20.5673 -6.5592

H 20.7661 20.0353 -5.6422

H 21.0741 21.5043 -6.5682

H 20.8571 19.9623 -7.4002

O 6.2571 18.1543 -3.2122

O 3.8341 19.1363 -2.9352

O 7.0021 16.8373 -5.4732

O 1.2531 16.6513 -6.5292

C -0.9839 16.3973 -2.4452

C 0.2011 16.5953 -1.7282

C -2.1949 16.2683 -1.7542

C 0.1681 16.7093 -0.3552

H 1.1361 16.6643 -2.2642

C -2.2219 16.3723 -0.3832

H -3.1049 16.0973 -2.3102

C -1.0449 16.6143 0.3268

H 1.0761 16.8733 0.2028

H -3.1469 16.2843 0.1648

C -1.4289 16.3243 -13.2542

C -0.3449 16.6813 -14.0642

C -2.6489 15.9863 -13.8512

C -0.4959 16.7563 -15.4302

H 0.6061 16.9073 -13.6062

C -2.7969 16.0603 -15.2162

H -3.4679 15.6693 -13.2242

C -1.7309 16.4733 -16.0162

H 0.3291 17.0493 -16.0612

H -3.7329 15.7963 -15.6812

C -4.7459 16.8623 -8.4072

H -5.1129 15.9723 -7.8702

H -4.4849 17.6273 -7.6662

C 2.3901 17.1483 -7.2092

H 2.7711 16.4043 -7.9212

H 2.1101 18.0573 -7.7632

1_polymer_RT_propagated

C -2.8908 -4.6072 -30.9126

C -3.0378 -4.5302 -36.5606

C -3.0688 -4.5142 -37.7686

C -3.1258 -4.5162 -39.1716

C -1.9338 -4.5762 -39.9256

C -4.3678 -4.4452 -39.8096

H -5.2428 -4.3542 -39.1866

O -3.9348 -4.6222 -30.2646

O -1.7068 -4.6422 -30.3526

H -1.7608 -4.6562 -29.3416

C -3.1668 -5.3992 -50.1566

C -3.3528 -5.0472 -44.5186

C -3.3418 -4.8502 -43.3266

C -3.2848 -4.6742 -41.9346

C -4.4688 -4.4942 -41.1896

C -2.0408 -4.6722 -41.3036

H -1.1688 -4.7642 -41.9316

O -2.1058 -5.3312 -50.7606

O -4.3288 -5.4932 -50.7666

H -4.2108 -5.4852 -51.7666

C -7.8778 -3.6882 -42.2426

C -7.6168 -3.3582 -43.5626

H -6.5908 -3.2382 -43.8686

C -8.6668 -3.2292 -44.4676

C -9.9888 -3.4062 -44.0356

C -10.2468 -3.6612 -42.6816

C -9.1848 -3.8242 -41.8016

H -9.3638 -4.0692 -40.7676

C -7.2458 -2.9752 -46.3666

H -6.6688 -2.0922 -46.0516

H -6.7078 -3.8702 -46.0306

C -7.3628 -2.9942 -47.8916

H -7.8078 -2.0582 -48.2296

H -6.3458 -3.0532 -48.2866

C -8.1948 -4.1652 -48.4256

H -8.0558 -5.0342 -47.7796

H -7.8358 -4.4342 -49.4226

C -9.6818 -3.8232 -48.5046

H -10.0138 -3.4672 -47.5306

H -9.8218 -3.0092 -49.2206

C -10.5388 -5.0142 -48.9276

H -10.4318 -5.8142 -48.1906

H -10.1868 -5.4062 -49.8866

C -12.0108 -4.6212 -49.0376

H -12.3038 -4.0952 -48.1286

H -12.1348 -3.9222 -49.8686

C -12.9388 -5.8172 -49.2386

H -12.7958 -6.5252 -48.4176

H -12.6818 -6.3362 -50.1646

C -14.4018 -5.3852 -49.2816

H -14.6418 -4.8442 -48.3646

H -14.5458 -4.6922 -50.1146

C -15.3638 -6.5602 -49.4346

H -15.2118 -7.2622 -48.6106

H -15.1478 -7.0932 -50.3626

C -16.8178 -6.0962 -49.4416

H -16.9668 -5.3972 -50.2696

H -17.0268 -5.5562 -48.5156

C -17.8068 -7.2502 -49.5816

H -17.6098 -7.7832 -50.5136

H -17.6568 -7.9552 -48.7606

C -19.2488 -6.7582 -49.5726

H -19.4188 -6.0652 -50.3946

H -19.9408 -7.5902 -49.6796

H -19.4698 -6.2432 -48.6406

C -11.8048 -4.4822 -45.1046

H -11.5298 -4.9652 -46.0516

H -11.6048 -5.1882 -44.2936

C -13.2838 -4.1152 -45.1136

H -13.5188 -3.6102 -44.1766

H -13.4658 -3.4112 -45.9276

C -14.1658 -5.3492 -45.2856

H -13.9678 -6.0522 -44.4726

H -13.9078 -5.8522 -46.2206

C -15.6498 -4.9932 -45.2946

H -15.9048 -4.4822 -44.3636

H -15.8468 -4.2972 -46.1136

C -16.5398 -6.2222 -45.4586

H -16.3328 -6.9302 -44.6516

H -16.2978 -6.7222 -46.3986

C -18.0228 -5.8612 -45.4426

H -18.2598 -5.3522 -44.5056

H -18.2348 -5.1632 -46.2556

C -18.9178 -7.0882 -45.5906

H -18.6958 -7.7982 -44.7896

H -18.6988 -7.5862 -46.5376

C -20.3988 -6.7222 -45.5426

H -20.6178 -6.2332 -44.5906

H -20.6208 -6.0042 -46.3366

C -21.3018 -7.9422 -45.7026

H -21.0628 -8.6732 -44.9256

H -21.1038 -8.4142 -46.6686

C -22.7808 -7.5742 -45.6106

H -22.9718 -7.0912 -44.6506

H -23.0228 -6.8532 -46.3956

C -23.6908 -8.7912 -45.7506

H -23.4378 -9.5232 -44.9796

H -23.5168 -9.2632 -46.7206

C -25.1618 -8.4132 -45.6276

H -25.4368 -7.6972 -46.3986

H -25.7948 -9.2912 -45.7326

H -25.3588 -7.9632 -44.6566

C -11.8718 -3.8222 -40.9486

H -11.5738 -4.7942 -40.5296

H -11.3368 -3.0252 -40.4136

C -13.3728 -3.6152 -40.7966

H -13.5798 -3.3822 -39.7516

H -13.6558 -2.7482 -41.3956

C -14.2038 -4.8222 -41.2226

H -13.9778 -5.0712 -42.2586

H -13.9408 -5.6842 -40.6046

C -15.6928 -4.5242 -41.0726

H -15.9598 -3.6952 -41.7326

H -15.8898 -4.1962 -40.0486

C -16.5838 -5.7222 -41.3886

H -16.3478 -6.5422 -40.7066

H -16.3868 -6.0682 -42.4036

C -18.0598 -5.3582 -41.2526

H -18.3008 -4.5652 -41.9656

H -18.2408 -4.9562 -40.2526

C -18.9888 -6.5452 -41.4906

H -18.8088 -6.9542 -42.4866

H -18.7648 -7.3302 -40.7656

C -20.4548 -6.1422 -41.3646

H -20.6848 -5.3832 -42.1166

H -20.6218 -5.6852 -40.3856

C -21.4058 -7.3242 -41.5366

H -21.1698 -8.0892 -40.7936

H -21.2578 -7.7652 -42.5246

C -22.8648 -6.9042 -41.3816

H -23.0148 -6.4732 -40.3886

H -23.0978 -6.1252 -42.1126

C -23.8288 -8.0712 -41.5766

H -23.6188 -8.8422 -40.8316

H -23.6668 -8.5122 -42.5616

C -25.2818 -7.6272 -41.4526

H -25.5088 -6.8582 -42.1886

H -25.9558 -8.4662 -41.6116

H -25.4718 -7.2162 -40.4636

O -11.0018 -3.3142 -44.9596

O -8.5318 -2.9442 -45.7896

O -11.5568 -3.7632 -42.3236

O -5.6158 -4.3882 -41.9036

C 1.6612 -4.4312 -39.1546

C 1.6652 -4.9402 -37.8676

H 0.7242 -5.2132 -37.4186

C 2.8762 -5.1062 -37.2066

C 4.0902 -4.7812 -37.8346

C 4.0732 -4.2142 -39.1186

C 2.8492 -4.0292 -39.7516

H 2.8082 -3.6042 -40.7416

C 1.9302 -5.7222 -35.0876

H 1.0772 -6.2202 -35.5646

H 1.6172 -4.7062 -34.7966

C 2.3312 -6.5222 -33.8486

H 2.5212 -7.5552 -34.1446

H 1.4692 -6.5102 -33.1796

C 3.5652 -5.9742 -33.1256

H 3.5442 -4.8832 -33.1576

H 3.5272 -6.2722 -32.0736

C 4.8702 -6.4762 -33.7436

H 4.8632 -6.2662 -34.8126

H 4.9332 -7.5602 -33.6156

C 6.0982 -5.8222 -33.1166

H 6.0252 -4.7392 -33.2476

H 6.1172 -6.0132 -32.0386

C 7.3972 -6.3162 -33.7526

H 7.3042 -6.2542 -34.8376

H 7.5552 -7.3662 -33.4926

C 8.5942 -5.4882 -33.2956

H 8.4332 -4.4472 -33.5916

H 8.6532 -5.5192 -32.2056

C 9.9302 -5.9582 -33.8646

H 9.8892 -5.9442 -34.9556

H 10.1192 -6.9872 -33.5506

C 11.0672 -5.0592 -33.3826

H 10.8732 -4.0342 -33.7086

H 11.0812 -5.0572 -32.2896

C 12.4362 -5.4962 -33.8926

H 12.6282 -6.5262 -33.5836

H 12.4422 -5.4722 -34.9846

C 13.5482 -4.5942 -33.3606

H 13.5542 -4.6382 -32.2686

H 13.3392 -3.5612 -33.6476

C 14.9182 -4.9992 -33.8866

H 15.1562 -6.0192 -33.5916

H 15.6902 -4.3402 -33.4946

H 14.9412 -4.9442 -34.9726

C 6.2642 -4.1752 -37.0016

H 6.3632 -4.0012 -35.9226

H 6.0142 -3.2282 -37.4806

C 7.5912 -4.7052 -37.5396

H 7.5342 -4.7812 -38.6246

H 7.7632 -5.7072 -37.1406

C 8.7172 -3.7652 -37.1206

H 8.4832 -2.7622 -37.4846

H 8.7482 -3.7212 -36.0296

C 10.0912 -4.1862 -37.6336

H 10.0962 -4.1792 -38.7246

H 10.3092 -5.2082 -37.3106

C 11.1712 -3.2452 -37.1046

H 10.9232 -2.2212 -37.3936

H 11.1702 -3.2882 -36.0126

C 12.5722 -3.5742 -37.6116

H 12.5982 -3.4932 -38.6996

H 12.8242 -4.6062 -37.3526

C 13.6052 -2.6302 -37.0006

H 13.3322 -1.5982 -37.2366

H 13.5812 -2.7362 -35.9136

C 15.0252 -2.8932 -37.4936

H 15.0692 -2.7462 -38.5746

H 15.2972 -3.9322 -37.2926

C 16.0282 -1.9652 -36.8116

H 15.7342 -0.9272 -36.9906

H 15.9942 -2.1372 -35.7326

C 17.4562 -2.1732 -37.3086

H 17.4872 -2.0192 -38.3896

H 17.7632 -3.2032 -37.1136

C 18.4362 -1.2142 -36.6356

H 18.0992 -0.1872 -36.7946

H 18.4382 -1.3982 -35.5586

C 19.8512 -1.3662 -37.1776

H 20.2172 -2.3772 -37.0136

H 20.5262 -0.6712 -36.6826

H 19.8732 -1.1632 -38.2456

C 5.3162 -3.4552 -41.0146

H 4.9492 -2.4202 -41.0566

H 4.6792 -4.0842 -41.6506

C 6.7512 -3.5392 -41.5286

H 6.7312 -3.3602 -42.6056

H 7.1122 -4.5562 -41.3636

C 7.7002 -2.5462 -40.8626

H 7.7142 -2.7242 -39.7886

H 7.3352 -1.5312 -41.0176

C 9.1112 -2.6722 -41.4346

H 9.4632 -3.6992 -41.3066

H 9.0832 -2.4702 -42.5076

C 10.0992 -1.7192 -40.7676

H 9.7412 -0.6932 -40.8736

H 10.1472 -1.9452 -39.7006

C 11.5002 -1.8302 -41.3656

H 11.8492 -2.8622 -41.2746

H 11.4622 -1.5922 -42.4306

C 12.4962 -0.9032 -40.6716

H 12.4992 -1.1202 -39.6016

H 12.1742 0.1338 -40.7976

C 13.9142 -1.0602 -41.2136

H 14.2322 -2.0992 -41.0936

H 13.9262 -0.8392 -42.2836

C 14.9012 -0.1452 -40.4926

H 14.6022 0.8958 -40.6396

H 14.8622 -0.3512 -39.4196

C 16.3352 -0.3302 -40.9816

H 16.3812 -0.1412 -42.0576

H 16.6432 -1.3672 -40.8216

C 17.3072 0.6038 -40.2646

H 16.9642 1.6338 -40.3806

H 17.3072 0.3718 -39.1976

C 18.7252 0.4848 -40.8076

H 19.0892 -0.5362 -40.7056

H 19.3992 1.1458 -40.2686

H 18.7542 0.7498 -41.8626

O 5.2212 -5.1302 -37.1546

O 3.0282 -5.6432 -35.9626

O 5.2772 -3.9212 -39.6806

O -0.7658 -4.5382 -39.2366

C -2.9958 -4.5342 -35.1566

C -1.7678 -4.6452 -34.4926

C -4.1728 -4.4212 -34.4126

C -1.7228 -4.6562 -33.1196

H -0.8648 -4.7212 -35.0776

C -4.1268 -4.4402 -33.0386

H -5.1168 -4.3132 -34.9266

C -2.9028 -4.5652 -32.3816

H -0.7808 -4.7412 -32.5996

H -5.0318 -4.3542 -32.4576

C -3.3248 -5.2052 -45.9146

C -2.1038 -5.1032 -46.5896

C -4.4958 -5.4432 -46.6396

C -2.0708 -5.1802 -47.9616

H -1.2008 -4.9532 -46.0186

C -4.4598 -5.5132 -48.0136

H -5.4228 -5.5792 -46.1036

C -3.2468 -5.3652 -48.6866

H -1.1458 -5.0842 -48.5066

H -5.3628 -5.6822 -48.5796

C -6.7618 -3.8892 -41.2496

H -7.0968 -4.5922 -40.4736

H -6.5198 -2.9292 -40.7656

C 0.4172 -4.4152 -40.0026

H 0.4922 -5.2662 -40.6976

H 0.3742 -3.4882 -40.5906

C -2.3918 -4.6452 -8.2226

C -3.1618 -4.2422 -13.8076

C -3.2108 -4.1802 -15.0146

C -3.2618 -4.1222 -16.4166

C -2.0538 -4.0952 -17.1486

C -4.4958 -4.0882 -17.0726

H -5.3808 -4.0902 -16.4566

O -3.3298 -4.6652 -7.4276

O -1.1398 -4.7582 -7.8486

H -1.0358 -4.8512 -6.8526

C -3.0308 -4.5812 -27.4256

C -3.2478 -4.2752 -21.7856

C -3.3708 -4.1802 -20.5866

C -3.3718 -4.1122 -19.1846

C -4.5778 -4.0612 -18.4556

C -2.1388 -4.1062 -18.5306

H -1.2558 -4.1252 -19.1486

O -1.9798 -4.6292 -28.0476

O -4.2088 -4.5902 -28.0076

H -4.1208 -4.6222 -29.0126

C -8.1208 -3.6642 -19.3986

C -8.0368 -3.7872 -20.7736

H -7.0738 -3.9782 -21.2156

C -9.1918 -3.6832 -21.5506

C -10.4288 -3.4542 -20.9366

C -10.5008 -3.3092 -19.5446

C -9.3438 -3.4172 -18.7876

H -9.3778 -3.3152 -17.7156

C -8.1018 -4.2132 -23.6066

H -7.2958 -3.4762 -23.4746

H -7.7578 -5.1842 -23.2216

C -8.4298 -4.3282 -25.0956

H -8.7078 -3.3442 -25.4726

H -7.5108 -4.6402 -25.5966

C -9.5518 -5.3222 -25.4096

H -9.5108 -6.1522 -24.7006

H -9.3908 -5.7362 -26.4086

C -10.9368 -4.6792 -25.3616

H -11.0818 -4.2082 -24.3896

H -10.9918 -3.8942 -26.1206

C -12.0438 -5.7002 -25.6136

H -12.0188 -6.4592 -24.8266

H -11.8628 -6.2102 -26.5636

C -13.4298 -5.0582 -25.6436

H -13.5788 -4.4852 -24.7266

H -13.4868 -4.3572 -26.4806

C -14.5358 -6.1012 -25.7766

H -14.4668 -6.8032 -24.9416

H -14.3858 -6.6732 -26.6956

C -15.9308 -5.4802 -25.7916

H -16.0718 -4.8832 -24.8896

H -16.0188 -4.8072 -26.6486

C -17.0218 -6.5452 -25.8716

H -16.9228 -7.2252 -25.0206

H -16.8808 -7.1372 -26.7796

C -18.4258 -5.9472 -25.8746

H -18.5308 -5.2782 -26.7326

H -18.5668 -5.3492 -24.9716

C -19.5048 -7.0242 -25.9436

H -19.3628 -7.6172 -26.8496

H -19.3978 -7.6982 -25.0896

C -20.9068 -6.4282 -25.9456

H -21.0418 -5.7762 -26.8056

H -21.6588 -7.2132 -25.9916

H -21.0748 -5.8432 -25.0436

C -12.4718 -4.4472 -21.5906

H -12.2788 -5.1662 -22.3996

H -12.3218 -4.9622 -20.6356

C -13.9018 -3.9262 -21.6796

H -14.0808 -3.2582 -20.8366

H -13.9988 -3.3402 -22.5946

C -14.9248 -5.0592 -21.6836

H -14.7888 -5.6752 -20.7906

H -14.7528 -5.6992 -22.5516

C -16.3558 -4.5292 -21.7206

H -16.5388 -3.9162 -20.8366

H -16.4758 -3.8862 -22.5956

C -17.3898 -5.6512 -21.7836

H -17.2618 -6.3122 -20.9226

H -17.2208 -6.2472 -22.6826

C -18.8188 -5.1112 -21.7976

H -18.9968 -4.5332 -20.8886

H -18.9388 -4.4342 -22.6466

C -19.8558 -6.2262 -21.9016

H -19.7498 -6.9022 -21.0496

H -19.6708 -6.8072 -22.8066

C -21.2808 -5.6802 -21.9366

H -21.4788 -5.1242 -21.0186

H -21.3778 -4.9792 -22.7706

C -22.3188 -6.7892 -22.0896

H -22.2368 -7.4772 -21.2456

H -22.1098 -7.3572 -22.9996

C -23.7408 -6.2382 -22.1556

H -23.9478 -5.6642 -21.2496

H -23.8238 -5.5552 -23.0056

C -24.7848 -7.3422 -22.2966

H -24.7138 -8.0172 -21.4416

H -24.5738 -7.9252 -23.1966

C -26.1978 -6.7782 -22.3786

H -26.2958 -6.1162 -23.2356

H -26.9258 -7.5802 -22.4796

H -26.4358 -6.2122 -21.4806

C -11.8898 -2.9502 -17.6386

H -11.5428 -3.8592 -17.1256

H -11.2988 -2.0952 -17.2806

C -13.3678 -2.7162 -17.3556

H -13.4818 -2.4212 -16.3126

H -13.7038 -1.8852 -17.9776

C -14.2218 -3.9462 -17.6496

H -14.0248 -4.2792 -18.6696

H -13.9438 -4.7582 -16.9746

C -15.7078 -3.6372 -17.4936

H -15.9818 -2.8492 -18.1986

H -15.8938 -3.2502 -16.4886

C -16.5948 -4.8542 -17.7376

H -16.3918 -5.6142 -16.9806

H -16.3578 -5.2872 -18.7116

C -18.0728 -4.4782 -17.6996

H -18.2818 -3.7732 -18.5076

H -18.2928 -3.9672 -16.7586

C -18.9938 -5.6862 -17.8416

H -18.7528 -6.2202 -18.7626

H -18.8248 -6.3712 -17.0076

C -20.4618 -5.2702 -17.8676

H -20.6298 -4.6162 -18.7276

H -20.6898 -4.6922 -16.9686

C -21.4108 -6.4612 -17.9526

H -21.2648 -7.1052 -17.0826

H -21.1738 -7.0482 -18.8436

C -22.8688 -6.0152 -18.0156

H -23.1108 -5.4412 -17.1176

H -23.0038 -5.3562 -18.8766

C -23.8348 -7.1902 -18.1376

H -23.7098 -7.8532 -17.2786

H -23.5928 -7.7632 -19.0356

C -25.2828 -6.7232 -18.2126

H -25.4248 -6.0642 -19.0666

H -25.9568 -7.5692 -18.3176

H -25.5548 -6.1762 -17.3116

O -11.5588 -3.3612 -21.7186

O -9.2488 -3.7982 -22.9026

O -11.7408 -3.0922 -19.0346

O -5.7238 -3.9982 -19.1816

C 1.5342 -3.8732 -16.3366

C 1.5732 -4.4062 -15.0596

H 0.6562 -4.7652 -14.6216

C 2.7902 -4.4792 -14.3896

C 3.9762 -4.0372 -15.0006

C 3.9222 -3.4642 -16.2816

C 2.6892 -3.3682 -16.9186

H 2.6202 -2.9292 -17.9006

C 1.8792 -5.2302 -12.3066

H 1.1212 -5.8512 -12.8006

H 1.4182 -4.2652 -12.0446

C 2.3442 -5.9482 -11.0406

H 2.7082 -6.9402 -11.3126

H 1.4592 -6.0702 -10.4126

C 3.4382 -5.2062 -10.2686

H 3.2532 -4.1312 -10.3266

H 3.3922 -5.4912 -9.2146

C 4.8352 -5.5132 -10.8046

H 4.8712 -5.2862 -11.8706

H 5.0352 -6.5812 -10.6856

C 5.9152 -4.7162 -10.0786

H 5.7122 -3.6502 -10.2046

H 5.8732 -4.9272 -9.0056

C 7.3122 -5.0272 -10.6146

H 7.3002 -4.9442 -11.7026

H 7.5792 -6.0572 -10.3646

C 8.3592 -4.0732 -10.0456

H 8.1022 -3.0522 -10.3406

H 8.3272 -4.1172 -8.9536

C 9.7782 -4.3822 -10.5196

H 9.8012 -4.3972 -11.6106

H 10.0722 -5.3752 -10.1686

C 10.7712 -3.3442 -10.0026

H 10.5002 -2.3622 -10.3986

H 10.6982 -3.2932 -8.9126

C 12.2192 -3.6522 -10.3746

H 12.5032 -4.6202 -9.9536

H 12.3122 -3.7252 -11.4596

C 13.1702 -2.5772 -9.8526

H 13.0302 -2.4672 -8.7736

H 12.9172 -1.6182 -10.3136

C 14.6282 -2.9112 -10.1396

H 14.9092 -3.8422 -9.6516

H 15.2822 -2.1222 -9.7726

H 14.7912 -3.0242 -11.2086

C 6.0862 -3.2542 -14.1416

H 6.1002 -3.0052 -13.0716

H 5.8122 -2.3552 -14.6956

C 7.4702 -3.7392 -14.5606

H 7.4892 -3.8792 -15.6416

H 7.6602 -4.7092 -14.0946

C 8.5262 -2.7272 -14.1316

H 8.2862 -1.7652 -14.5866

H 8.4742 -2.6002 -13.0466

C 9.9472 -3.1362 -14.5136

H 10.0262 -3.2182 -15.5996

H 10.1712 -4.1192 -14.0936

C 10.9622 -2.1182 -13.9986

H 10.7202 -1.1352 -14.4086

H 10.8762 -2.0502 -12.9116

C 12.4032 -2.4652 -14.3636

H 12.5062 -2.5012 -15.4506

H 12.6502 -3.4572 -13.9776

C 13.3782 -1.4392 -13.7906

H 13.1242 -0.4492 -14.1776

H 13.2622 -1.4052 -12.7056

C 14.8332 -1.7492 -14.1276

H 14.9642 -1.7432 -15.2116

H 15.0852 -2.7512 -13.7716

C 15.7832 -0.7332 -13.4956

H 15.5222 0.2678 -13.8486

H 15.6512 -0.7442 -12.4116

C 17.2452 -1.0152 -13.8276

H 17.3792 -1.0012 -14.9116

H 17.5112 -2.0152 -13.4756

C 18.1812 0.0088 -13.1906

H 17.9012 1.0098 -13.5256

H 18.0632 -0.0212 -12.1056

C 19.6402 -0.2472 -13.5486

H 19.9502 -1.2322 -13.2066

H 20.2842 0.4958 -13.0846

H 19.7822 -0.1982 -14.6256

C 5.1192 -2.5922 -18.1626

H 4.7012 -1.5752 -18.1926

H 4.5162 -3.2442 -18.8076

C 6.5592 -2.6002 -18.6696

H 6.5422 -2.3832 -19.7406

H 6.9592 -3.6072 -18.5366

C 7.4602 -1.5992 -17.9526

H 7.4372 -1.7992 -16.8826

H 7.0792 -0.5882 -18.1026

C 8.8962 -1.6842 -18.4676

H 9.2592 -2.7092 -18.3526

H 8.9122 -1.4502 -19.5356

C 9.8362 -0.7372 -17.7266

H 9.4762 0.2878 -17.8326

H 9.8222 -0.9842 -16.6636

C 11.2702 -0.8262 -18.2426

H 11.6262 -1.8542 -18.1336

H 11.2912 -0.5842 -19.3076

C 12.2122 0.1098 -17.4906

H 12.1442 -0.1002 -16.4206

H 11.8962 1.1448 -17.6456

C 13.6652 -0.0452 -17.9336

H 13.9842 -1.0772 -17.7636

H 13.7442 0.1478 -19.0066

C 14.5952 0.8978 -17.1746

H 14.3002 1.9318 -17.3716

H 14.4842 0.7218 -16.1026

C 16.0602 0.7088 -17.5596

H 16.1782 0.8588 -18.6356

H 16.3632 -0.3172 -17.3386

C 16.9732 1.6778 -16.8126

H 16.6412 2.7008 -17.0016

H 16.8872 1.4928 -15.7396

C 18.4302 1.5358 -17.2326

H 18.7842 0.5238 -17.0476

H 19.0592 2.2268 -16.6746

H 18.5442 1.7478 -18.2936

O 5.1242 -4.2892 -14.3076

O 2.9812 -5.0122 -13.1506

O 5.1032 -3.0742 -16.8336

O -0.8968 -4.0672 -16.4416

C -2.9988 -4.3202 -12.4146

C -1.7028 -4.4232 -11.8966

C -4.0878 -4.2952 -11.5396

C -1.5018 -4.5182 -10.5426

H -0.8738 -4.4242 -12.5856

C -3.8848 -4.3852 -10.1816

H -5.0868 -4.2042 -11.9356

C -2.5928 -4.5102 -9.6716

H -0.5058 -4.5992 -10.1376

H -4.7228 -4.3632 -9.5026

C -3.1838 -4.3652 -23.1846

C -1.9548 -4.4692 -23.8406

C -4.3658 -4.3462 -23.9306

C -1.9148 -4.5362 -25.2146

H -1.0468 -4.4962 -23.2586

C -4.3228 -4.4152 -25.3026

H -5.3068 -4.2792 -23.4096

C -3.0948 -4.5092 -25.9566

H -0.9808 -4.6062 -25.7466

H -5.2298 -4.3952 -25.8846

C -6.9308 -3.8142 -18.4796

H -7.1148 -4.6782 -17.8206

H -6.8588 -2.9132 -17.8496

C 0.2962 -3.9242 -17.1936

H 0.4002 -4.7862 -17.8716

H 0.2372 -3.0102 -17.7986

C -2.2848 -7.3482 -20.7086

C -2.2218 -7.7652 -26.3526

C -2.2518 -7.7712 -27.5606

C -2.3288 -7.7472 -28.9636

C -1.1568 -7.7342 -29.7466

C -3.5798 -7.6822 -29.5766

H -4.4438 -7.6682 -28.9316

O -3.3248 -7.4572 -20.0736

O -1.1278 -7.0802 -20.1496

H -1.1998 -7.0302 -19.1466

C -2.4288 -7.7282 -40.0196

C -2.6188 -7.7582 -34.3536

C -2.6118 -7.7302 -33.1446

C -2.5398 -7.7072 -31.7396

C -3.7058 -7.6082 -30.9546

C -1.2858 -7.7552 -31.1246

H -0.4188 -7.8052 -31.7646

O -1.3688 -7.6052 -40.6206

O -3.5818 -7.8882 -40.6226

H -3.4798 -7.9662 -41.6226

C -7.1518 -6.5522 -31.6086

C -7.1588 -6.5182 -32.9896

H -6.2688 -6.8182 -33.5166

C -8.3168 -6.1302 -33.6606

C -9.4708 -5.8042 -32.9346

C -9.4328 -5.7822 -31.5316

C -8.2648 -6.1502 -30.8836

H -8.2068 -6.1542 -29.8076

C -7.3928 -6.5352 -35.8276

H -6.4638 -5.9832 -35.6236

H -7.2188 -7.5992 -35.6146

C -7.7638 -6.3522 -37.2986

H -7.9858 -5.2972 -37.4706

H -6.8798 -6.6182 -37.8826

C -8.9528 -7.2042 -37.7516

H -8.8718 -8.1982 -37.3076

H -8.9048 -7.3272 -38.8366

C -10.3018 -6.5882 -37.3816

H -10.3128 -6.3592 -36.3166

H -10.4248 -5.6452 -37.9196

C -11.4708 -7.5122 -37.7156

H -11.3608 -8.4482 -37.1616

H -11.4488 -7.7612 -38.7806

C -12.8148 -6.8772 -37.3686

H -12.8058 -6.5712 -36.3216

H -12.9558 -5.9752 -37.9676

C -13.9848 -7.8312 -37.6026

H -13.8468 -8.7262 -36.9906

H -13.9908 -8.1502 -38.6486

C -15.3288 -7.1912 -37.2626

H -15.3118 -6.8432 -36.2286

H -15.4848 -6.3172 -37.8986

C -16.4918 -8.1642 -37.4456

H -16.3588 -9.0162 -36.7726

H -16.4798 -8.5542 -38.4676

C -17.8428 -7.5092 -37.1726

H -17.9858 -6.6772 -37.8636

H -17.8488 -7.1002 -36.1606

C -19.0018 -8.4922 -37.3226

H -18.9688 -8.9412 -38.3186

H -18.8858 -9.3022 -36.5976

C -20.3528 -7.8172 -37.1176

H -20.4958 -7.0252 -37.8496

H -21.1618 -8.5352 -37.2266

H -20.4138 -7.3792 -36.1246

C -11.6328 -6.5502 -33.4586

H -11.6208 -7.1842 -34.3576

H -11.4038 -7.1852 -32.5966

C -13.0038 -5.9122 -33.2806

H -12.9968 -5.3272 -32.3616

H -13.1738 -5.2252 -34.1116

C -14.1158 -6.9562 -33.2376

H -13.9468 -7.6382 -32.4006

H -14.0898 -7.5522 -34.1536

C -15.4888 -6.3042 -33.0966

H -15.5298 -5.7472 -32.1586

H -15.6268 -5.5872 -33.9096

C -16.6258 -7.3222 -33.1326

H -16.5098 -8.0312 -32.3096

H -16.5728 -7.8912 -34.0646

C -17.9898 -6.6442 -33.0316

H -18.0518 -6.0982 -32.0886

H -18.0858 -5.9132 -33.8386

C -19.1438 -7.6392 -33.1176

H -19.0648 -8.3582 -32.2986

H -19.0718 -8.1992 -34.0526

C -20.5008 -6.9412 -33.0466

H -20.5728 -6.3902 -32.1076

H -20.5758 -6.2142 -33.8596

C -21.6658 -7.9222 -33.1466

H -21.6088 -8.6362 -32.3216

H -21.5838 -8.4902 -34.0766

C -23.0138 -7.2082 -33.1076

H -23.0908 -6.6392 -32.1786

H -23.0688 -6.4992 -33.9376

C -24.1878 -8.1792 -33.1986

H -24.1388 -8.8852 -32.3666

H -24.1088 -8.7542 -34.1246

C -25.5288 -7.4552 -33.1656

H -25.6048 -6.7542 -33.9946

H -26.3488 -8.1652 -33.2406

H -25.6408 -6.9012 -32.2366

C -10.5708 -5.3712 -29.4826

H -10.2908 -6.3462 -29.0586

H -9.8398 -4.6202 -29.1546

C -11.9678 -4.9852 -29.0106

H -11.9168 -4.7152 -27.9546

H -12.2818 -4.1052 -29.5716

C -12.9838 -6.1082 -29.2086

H -12.9008 -6.4862 -30.2286

H -12.7578 -6.9312 -28.5266

C -14.4068 -5.6142 -28.9646

H -14.6338 -4.8292 -29.6906

H -14.4698 -5.1682 -27.9706

C -15.4538 -6.7172 -29.0896

H -15.3078 -7.4542 -28.2966

H -15.3278 -7.2322 -30.0446

C -16.8668 -6.1462 -29.0056

H -17.0088 -5.4362 -29.8236

H -16.9758 -5.5942 -28.0696

C -17.9518 -7.2162 -29.0856

H -17.8298 -7.7892 -30.0076

H -17.8428 -7.9102 -28.2486

C -19.3458 -6.5952 -29.0546

H -19.4508 -5.9142 -29.9036

H -19.4538 -6.0042 -28.1426

C -20.4588 -7.6372 -29.1116

H -20.3638 -8.3192 -28.2636

H -20.3548 -8.2302 -30.0236

C -21.8398 -6.9882 -29.0826

H -21.9318 -6.3832 -28.1786

H -21.9398 -6.3192 -29.9416

C -22.9688 -8.0132 -29.1136

H -22.8678 -8.6872 -28.2596

H -22.8868 -8.6152 -30.0216

C -24.3378 -7.3462 -29.0716

H -24.4648 -6.6832 -29.9256

H -25.1308 -8.0902 -29.0966

H -24.4478 -6.7572 -28.1636

O -10.6408 -5.5382 -33.6096

O -8.4398 -6.0512 -35.0126

O -10.5828 -5.4372 -30.8956

O -4.8848 -7.4242 -31.6076

C 2.2872 -7.0762 -28.7866

C 2.0492 -6.2752 -27.6806

H 1.0742 -5.8332 -27.5646

C 3.0552 -6.0772 -26.7386

C 4.3132 -6.6632 -26.9386

C 4.5572 -7.4322 -28.0836

C 3.5302 -7.6502 -28.9956

H 3.6882 -8.2732 -29.8616

C 1.6982 -4.7442 -25.2996

H 0.8762 -5.4552 -25.4616

H 1.5432 -3.8752 -25.9566

C 1.7002 -4.3092 -23.8346

H 1.7912 -5.1962 -23.2056

H 0.7292 -3.8522 -23.6376

C 2.8182 -3.3222 -23.4876

H 3.0532 -2.7202 -24.3646

H 2.4662 -2.6482 -22.7016

C 4.0922 -4.0112 -23.0046

H 4.4122 -4.7372 -23.7516

H 3.8782 -4.5532 -22.0786

C 5.2072 -2.9992 -22.7556

H 5.4822 -2.5322 -23.7036

H 4.8392 -2.2092 -22.0936

C 6.4442 -3.6372 -22.1276

H 6.7472 -4.4962 -22.7276

H 6.2032 -4.0022 -21.1256

C 7.6002 -2.6452 -22.0526

H 7.8382 -2.3222 -23.0676

H 7.2842 -1.7612 -21.4906

C 8.8572 -3.2272 -21.4116

H 9.1242 -4.1552 -21.9206

H 8.6592 -3.4702 -20.3646

C 10.0282 -2.2512 -21.4986

H 10.2302 -2.0322 -22.5496

H 9.7512 -1.3112 -21.0136

C 11.2992 -2.7942 -20.8506

H 11.1192 -2.9672 -19.7876

H 11.5522 -3.7562 -21.3036

C 12.4762 -1.8362 -21.0156

H 12.2162 -0.8692 -20.5776

H 12.6632 -1.6772 -22.0806

C 13.7422 -2.3632 -20.3546

H 13.5872 -2.4962 -19.2866

H 14.5692 -1.6702 -20.4976

H 14.0242 -3.3242 -20.7806

C 6.2032 -5.4402 -26.2706

H 5.7732 -4.4892 -25.9276

H 6.3762 -5.3652 -27.3506

C 7.5202 -5.7152 -25.5556

H 7.9502 -6.6372 -25.9536

H 7.3142 -5.8802 -24.4976

C 8.4882 -4.5522 -25.7336

H 8.6072 -4.3452 -26.7996

H 8.0542 -3.6612 -25.2756

C 9.8582 -4.8332 -25.1226

H 10.2832 -5.7312 -25.5786

H 9.7482 -5.0312 -24.0546

C 10.8152 -3.6612 -25.3246

H 10.8922 -3.4372 -26.3916

H 10.4022 -2.7782 -24.8326

C 12.2092 -3.9392 -24.7686

H 12.6362 -4.8112 -25.2716

H 12.1332 -4.1792 -23.7066

C 13.1392 -2.7432 -24.9486

H 13.1912 -2.4792 -26.0076

H 12.7202 -1.8832 -24.4196

C 14.5472 -3.0172 -24.4266

H 14.9932 -3.8352 -24.9986

H 14.4912 -3.3392 -23.3846

C 15.4402 -1.7832 -24.5226

H 15.4402 -1.4182 -25.5536

H 15.0272 -0.9902 -23.8956

C 16.8762 -2.0702 -24.0916

H 17.2932 -2.8602 -24.7206

H 16.8792 -2.4342 -23.0616

C 17.7592 -0.8292 -24.1916

H 17.7062 -0.4322 -25.2086

H 17.3762 -0.0602 -23.5176

C 19.2122 -1.1292 -23.8466

H 19.2912 -1.5202 -22.8346

H 19.8162 -0.2262 -23.9126

H 19.6222 -1.8682 -24.5316

C 6.1652 -8.6432 -29.3686

H 5.9342 -8.0522 -30.2666

H 5.6042 -9.5872 -29.4156

C 7.6642 -8.9192 -29.3036

H 7.9552 -9.4902 -30.1876

H 7.8722 -9.5222 -28.4186

C 8.4532 -7.6152 -29.2276

H 8.1382 -7.0752 -28.3346

H 8.2102 -6.9992 -30.0966

C 9.9602 -7.8432 -29.1766

H 10.1962 -8.5382 -28.3676

H 10.2932 -8.3012 -30.1116

C 10.7142 -6.5352 -28.9526

H 10.4372 -5.8222 -29.7326

H 10.4122 -6.1082 -27.9946

C 12.2272 -6.7312 -28.9636

H 12.5012 -7.4672 -28.2036

H 12.5352 -7.1292 -29.9336

C 12.9732 -5.4272 -28.6936

H 12.6482 -5.0212 -27.7326

H 12.7122 -4.6962 -29.4626

C 14.4862 -5.6222 -28.6696

H 14.7432 -6.3422 -27.8876

H 14.8152 -6.0422 -29.6226

C 15.2252 -4.3122 -28.4106

H 15.0162 -3.6132 -29.2236

H 14.8462 -3.8662 -27.4876

C 16.7332 -4.5072 -28.2856

H 17.1192 -4.9612 -29.2016

H 16.9412 -5.1982 -27.4636

C 17.4562 -3.1872 -28.0286

H 17.2782 -2.5112 -28.8666

H 17.0372 -2.7222 -27.1336

C 18.9552 -3.3752 -27.8396

H 19.1562 -4.0262 -26.9906

H 19.4412 -2.4182 -27.6606

H 19.4002 -3.8252 -28.7246

O 5.3012 -6.5062 -25.9956

O 2.9332 -5.3552 -25.6016

O 5.8162 -7.9212 -28.2066

O 0.0162 -7.7052 -29.0706

C -2.2188 -7.7142 -24.9466

C -1.0148 -7.6712 -24.2366

C -3.4338 -7.6812 -24.2516

C -1.0258 -7.5602 -22.8636

H -0.0818 -7.7222 -24.7776

C -3.4398 -7.5812 -22.8806

H -4.3598 -7.7262 -24.8046

C -2.2378 -7.5022 -22.1756

H -0.1018 -7.5172 -22.3086

H -4.3658 -7.5482 -22.3266

C -2.5898 -7.7542 -35.7606

C -1.3628 -7.6882 -36.4296

C -3.7718 -7.8112 -36.5056

C -1.3188 -7.6622 -37.8046

H -0.4498 -7.6702 -35.8526

C -3.7268 -7.7982 -37.8816

H -4.7178 -7.8712 -35.9886

C -2.5008 -7.7212 -38.5436

H -0.3768 -7.6072 -38.3286

H -4.6338 -7.8512 -38.4636

C -5.9768 -7.0352 -30.8026

H -6.3098 -7.8882 -30.1906

H -5.6648 -6.2272 -30.1266

C 1.1782 -7.3192 -29.7766

H 1.4762 -8.1012 -30.4876

H 0.9682 -6.3942 -30.3356

C -2.2568 -8.0602 -43.7066

C -2.1088 -8.4862 -49.3506

C -2.1478 -8.4802 -50.5596

C -2.2118 -8.4472 -51.9636

C -1.0298 -8.3232 -52.7246

C -3.4538 -8.4862 -52.5986

H -4.3298 -8.5542 -51.9726

O -3.3008 -8.2272 -43.0916

O -1.1318 -7.7132 -43.1256

H -1.2328 -7.6552 -42.1266

C -3.0318 -8.2052 -62.9696

C -2.4538 -8.5152 -57.3516

C -2.4338 -8.4632 -56.1436

C -2.3718 -8.4202 -54.7396

C -3.5598 -8.4152 -53.9786

C -1.1298 -8.3552 -54.1046

H -0.2498 -8.3282 -54.7276

O -2.0938 -7.9712 -63.7216

O -4.2748 -8.3152 -63.3866

H -4.3628 -8.1892 -64.3786

C -6.9988 -7.4412 -54.7566

C -6.9958 -7.5102 -56.1386

H -6.1308 -7.9192 -56.6346

C -8.1088 -7.0682 -56.8526

C -9.2318 -6.5872 -56.1676

C -9.2028 -6.4692 -54.7706

C -8.0768 -6.8892 -54.0776

H -8.0308 -6.8122 -53.0036

C -7.1998 -7.6272 -58.9966

H -6.2458 -7.1182 -58.7966

H -7.0828 -8.6942 -58.7596

C -7.5598 -7.4542 -60.4716

H -7.7478 -6.3962 -60.6596

H -6.6838 -7.7542 -61.0486

C -8.7748 -8.2712 -60.9196

H -8.7518 -9.2482 -60.4326

H -8.7088 -8.4402 -61.9976

C -10.1008 -7.5772 -60.6106

H -10.1198 -7.2882 -59.5606

H -10.1678 -6.6632 -61.2046

C -11.3028 -8.4672 -60.9196

H -11.2608 -9.3622 -60.2936

H -11.2558 -8.7942 -61.9616

C -12.6258 -7.7432 -60.6736

H -12.6348 -7.3492 -59.6576

H -12.7008 -6.8902 -61.3516

C -13.8338 -8.6562 -60.8736

H -13.7968 -9.4712 -60.1456

H -13.7868 -9.1082 -61.8686

C -15.1548 -7.9032 -60.7256

H -15.1848 -7.4122 -59.7516

H -15.2088 -7.1212 -61.4856

C -16.3658 -8.8222 -60.8676

H -16.3788 -9.5412 -60.0436

H -16.2778 -9.3972 -61.7946

C -17.6768 -8.0412 -60.8856

H -17.6638 -7.3432 -61.7256

H -17.7608 -7.4522 -59.9696

C -18.8958 -8.9512 -61.0136

H -18.7828 -9.5842 -61.8966

H -18.9508 -9.6122 -60.1456

C -20.1888 -8.1532 -61.1286

H -20.1628 -7.5152 -62.0096

H -21.0458 -8.8182 -61.2146

H -20.3278 -7.5232 -60.2536

C -11.4088 -7.2212 -56.7396

H -11.3248 -7.9452 -57.5636

H -11.3018 -7.7712 -55.7976

C -12.7648 -6.5292 -56.7796

H -12.8358 -5.8542 -55.9266

H -12.8188 -5.9242 -57.6856

C -13.9128 -7.5332 -56.7556

H -13.8378 -8.1532 -55.8586

H -13.8328 -8.1992 -57.6186

C -15.2708 -6.8362 -56.7806

H -15.3718 -6.2072 -55.8946

H -15.3188 -6.1802 -57.6526

C -16.4298 -7.8262 -56.8376

H -16.3898 -8.4842 -55.9666

H -16.3268 -8.4532 -57.7256

C -17.7808 -7.1172 -56.8766

H -17.8978 -6.5102 -55.9766

H -17.8028 -6.4382 -57.7326

C -18.9448 -8.0972 -56.9846

H -18.9298 -8.7732 -56.1256

H -18.8208 -8.7062 -57.8826

C -20.2928 -7.3852 -57.0406

H -20.4218 -6.7842 -56.1376

H -20.3058 -6.7012 -57.8936

C -21.4568 -8.3642 -57.1676

H -21.4508 -9.0422 -56.3106

H -21.3238 -8.9692 -58.0666

C -22.8028 -7.6472 -57.2346

H -22.9228 -7.0242 -56.3456

H -22.8168 -6.9882 -58.1056

C -23.9728 -8.6242 -57.3236

H -23.9638 -9.2782 -56.4486

H -23.8498 -9.2532 -58.2086

C -25.3128 -7.9022 -57.3986

H -25.3498 -7.2542 -58.2716

H -26.1298 -8.6172 -57.4686

H -25.4688 -7.2922 -56.5116

C -10.3858 -5.8462 -52.7916

H -10.1598 -6.8082 -52.3086

H -9.6528 -5.0992 -52.4576

C -11.8018 -5.4072 -52.4336

H -11.8308 -5.1292 -51.3796

H -12.0408 -4.5232 -53.0266

C -12.8238 -6.5062 -52.7166

H -12.6528 -6.8962 -53.7206

H -12.6808 -7.3282 -52.0096

C -14.2548 -5.9872 -52.6156

H -14.4008 -5.2082 -53.3676

H -14.4068 -5.5282 -51.6366

C -15.2968 -7.0822 -52.8246

H -15.2078 -7.8282 -52.0306

H -15.1078 -7.5892 -53.7726

C -16.7108 -6.5082 -52.8286

H -16.8048 -5.8022 -53.6576

H -16.8748 -5.9522 -51.9046

C -17.7898 -7.5792 -52.9656

H -17.6258 -8.1472 -53.8836

H -17.7158 -8.2792 -52.1296

C -19.1858 -6.9642 -52.9916

H -19.2718 -6.3102 -53.8626

H -19.3228 -6.3462 -52.1016

C -20.2908 -8.0162 -53.0466

H -20.2228 -8.6582 -52.1656

H -20.1468 -8.6462 -53.9276

C -21.6768 -7.3802 -53.1036

H -21.8228 -6.7532 -52.2206

H -21.7388 -6.7332 -53.9826

C -22.7918 -8.4202 -53.1746

H -22.7418 -9.0632 -52.2926

H -22.6388 -9.0512 -54.0526

C -24.1688 -7.7722 -53.2526

H -24.2428 -7.1432 -54.1366

H -24.9478 -8.5302 -53.3076

H -24.3508 -7.1542 -52.3756

O -10.3698 -6.2552 -56.8676

O -8.2278 -7.0722 -58.2066

O -10.3288 -5.9772 -54.1966

O -4.7318 -8.3192 -54.6626

C 2.3182 -7.3712 -51.6846

C 1.9802 -6.6142 -50.5746

H 0.9722 -6.2432 -50.4886

C 2.9272 -6.3742 -49.5856

C 4.2332 -6.8692 -49.7346

C 4.5792 -7.5882 -50.8876

C 3.6082 -7.8472 -51.8496

H 3.8452 -8.4332 -52.7226

C 1.4352 -5.1452 -48.1796

H 0.6432 -5.8672 -48.4206

H 1.2922 -4.2512 -48.8046

C 1.3442 -4.7812 -46.6986

H 1.3992 -5.6972 -46.1066

H 0.3642 -4.3312 -46.5386

C 2.4372 -3.8142 -46.2386

H 2.6732 -3.1302 -47.0526

H 2.0592 -3.2242 -45.3986

C 3.7182 -4.5252 -45.8086

H 4.0382 -5.1952 -46.6046

H 3.5182 -5.1272 -44.9186

C 4.8332 -3.5262 -45.5146

H 5.0262 -2.9402 -46.4156

H 4.5132 -2.8352 -44.7286

C 6.1242 -4.2172 -45.0856

H 6.3722 -4.9822 -45.8236

H 5.9802 -4.7182 -44.1246

C 7.2782 -3.2242 -44.9896

H 7.3612 -2.7102 -45.9486

H 7.0512 -2.4672 -44.2336

C 8.6132 -3.8872 -44.6616

H 8.7822 -4.7142 -45.3546

H 8.5822 -4.3022 -43.6516

C 9.7692 -2.8962 -44.7756

H 9.8072 -2.5192 -45.8006

H 9.5812 -2.0402 -44.1226

C 11.1182 -3.5152 -44.4216

H 11.1042 -3.8462 -43.3806

H 11.2872 -4.3942 -45.0476

C 12.2642 -2.5272 -44.6246

H 12.0832 -1.6352 -44.0196

H 12.2832 -2.2152 -45.6716

C 13.6142 -3.1252 -44.2506

H 13.6232 -3.4192 -43.2036

H 14.4122 -2.4022 -44.4096

H 13.8222 -4.0032 -44.8576

C 6.1852 -5.7672 -48.9726

H 5.9342 -4.8012 -48.5126

H 6.2902 -5.6102 -50.0486

C 7.4982 -6.2982 -48.4046

H 7.7582 -7.2222 -48.9236

H 7.3602 -6.5392 -47.3496

C 8.5982 -5.2552 -48.5746

H 8.6242 -4.9382 -49.6206

H 8.3452 -4.3782 -47.9766

C 9.9842 -5.7562 -48.1756

H 10.2402 -6.6372 -48.7686

H 9.9802 -6.0592 -47.1266

C 11.0352 -4.6682 -48.3906

H 10.9802 -4.3222 -49.4266

H 10.8012 -3.8162 -47.7496

C 12.4592 -5.1372 -48.1026

H 12.7052 -5.9802 -48.7526

H 12.5272 -5.4872 -47.0706

C 13.4662 -4.0112 -48.3236

H 13.3632 -3.6312 -49.3446

H 13.2352 -3.1862 -47.6456

C 14.9102 -4.4522 -48.1016

H 15.1642 -5.2402 -48.8136

H 15.0122 -4.8692 -47.0966

C 15.8832 -3.2872 -48.2626

H 15.7672 -2.8572 -49.2616

H 15.6322 -2.5072 -47.5396

C 17.3372 -3.7072 -48.0666

H 17.5972 -4.4692 -48.8046

H 17.4532 -4.1522 -47.0756

C 18.2962 -2.5262 -48.2016

H 18.1842 -2.0842 -49.1956

H 18.0302 -1.7622 -47.4676

C 19.7482 -2.9392 -47.9996

H 19.8882 -3.3722 -47.0116

H 20.4072 -2.0792 -48.0936

H 20.0412 -3.6772 -48.7426

C 6.2442 -8.8472 -52.0416

H 6.0932 -8.3352 -53.0026

H 5.6342 -9.7602 -52.0296

C 7.7192 -9.1982 -51.8746

H 7.9882 -9.9332 -52.6356

H 7.8612 -9.6532 -50.8926

C 8.6012 -7.9612 -52.0036

H 8.3282 -7.2552 -51.2186

H 8.4052 -7.4842 -52.9666

C 10.0872 -8.2872 -51.9016

H 10.2842 -8.8122 -50.9646

H 10.3682 -8.9562 -52.7196

C 10.9382 -7.0232 -51.9656

H 10.6742 -6.4592 -52.8636

H 10.7102 -6.3912 -51.1046

C 12.4342 -7.3242 -51.9866

H 12.7012 -7.9012 -51.0976

H 12.6672 -7.9382 -52.8596

C 13.2662 -6.0452 -52.0306

H 13.0392 -5.4382 -51.1526

H 12.9832 -5.4622 -52.9106

C 14.7652 -6.3292 -52.0696

H 15.0482 -6.8892 -51.1756

H 14.9962 -6.9552 -52.9346

C 15.5842 -5.0432 -52.1416

H 15.3342 -4.5072 -53.0616

H 15.3132 -4.3992 -51.3036

C 17.0862 -5.3092 -52.1026

H 17.3662 -5.9412 -52.9496

H 17.3302 -5.8572 -51.1896

C 17.8942 -4.0152 -52.1436

H 17.6702 -3.4792 -53.0696

H 17.5902 -3.3782 -51.3116

C 19.3932 -4.2732 -52.0586

H 19.6362 -4.8042 -51.1406

H 19.9442 -3.3352 -52.0676

H 19.7272 -4.8772 -52.8996

O 5.1272 -6.6852 -48.7116

O 2.7052 -5.7042 -48.4336

O 5.8722 -8.0032 -50.9726

O 0.1232 -8.1882 -52.0286

C -2.1138 -8.4432 -47.9446

C -0.9288 -8.2442 -47.2276

C -3.3248 -8.5712 -47.2536

C -0.9618 -8.1312 -45.8546

H 0.0062 -8.1762 -47.7626

C -3.3508 -8.4672 -45.8826

H -4.2338 -8.7422 -47.8096

C -2.1748 -8.2262 -45.1716

H -0.0538 -7.9662 -45.2956

H -4.2768 -8.5552 -45.3346

C -2.5578 -8.5152 -58.7536

C -1.4748 -8.1582 -59.5636

C -3.7778 -8.8532 -59.3496

C -1.6248 -8.0832 -60.9286

H -0.5238 -7.9322 -59.1056

C -3.9258 -8.7792 -60.7146

H -4.5968 -9.1712 -58.7226

C -2.8598 -8.3662 -61.5156

H -0.8008 -7.7902 -61.5606

H -4.8628 -9.0432 -61.1806

C -5.8758 -7.9782 -53.9066

H -6.2418 -8.8682 -53.3696

H -5.6138 -7.2122 -53.1656

C 1.2602 -7.6922 -52.7076

H 1.6412 -8.4362 -53.4206

H 0.9812 -6.7822 -53.2626

C -1.6058 1.4398 -31.1176

C -1.4008 1.4528 -36.7816

C -1.4678 1.3998 -37.9886

C -1.6528 1.3428 -39.3816

C -0.5878 1.0748 -40.2656

C -2.9488 1.5138 -39.8796

H -3.7378 1.6758 -39.1616

O -2.6898 1.4558 -30.5516

O -0.4618 1.4128 -30.4696

H -0.6008 1.4338 -29.4716

C -1.8758 0.8908 -50.3696

C -2.2458 1.2898 -44.7306

C -2.2888 1.3028 -43.5216

C -2.1408 1.2978 -42.1226

C -3.2228 1.4638 -41.2366

C -0.8548 1.0888 -41.6246

H -0.0818 0.9158 -42.3546

O -0.7968 0.8448 -50.9466

O -3.0298 0.8238 -50.9876

H -2.9188 0.7478 -51.9866

C -6.8368 1.3498 -41.7856

C -6.7988 1.5998 -43.1446

H -5.8478 1.8078 -43.6036

C -7.9748 1.5768 -43.8856

C -9.1988 1.3118 -43.2576

C -9.2308 1.0798 -41.8736

C -8.0418 1.0768 -41.1536

H -8.0438 0.8758 -40.0936

C -6.8558 1.8598 -45.9596

H -6.2298 2.7068 -45.6416

H -6.2978 0.9268 -45.7866

C -7.1848 2.0028 -47.4436

H -7.7078 2.9478 -47.5986

H -6.2368 2.0488 -47.9836

C -8.0468 0.8538 -47.9736

H -7.7868 -0.0632 -47.4426

H -7.8298 0.6988 -49.0346

C -9.5398 1.1248 -47.8026

H -9.7368 1.3848 -46.7626

H -9.8168 1.9818 -48.4216

C -10.3988 -0.0762 -48.1936

H -10.1988 -0.9042 -47.5116

H -10.1378 -0.4052 -49.2046

C -11.8828 0.2758 -48.1446

H -12.1078 0.7108 -47.1696

H -12.0988 1.0358 -48.9006

C -12.7848 -0.9342 -48.3706

H -12.5858 -1.6712 -47.5896

H -12.5448 -1.3982 -49.3296

C -14.2598 -0.5422 -48.3486

H -14.4868 -0.0562 -47.3976

H -14.4498 0.1868 -49.1416

C -15.1898 -1.7372 -48.5346

H -15.0048 -2.4602 -47.7356

H -14.9628 -2.2312 -49.4816

C -16.6608 -1.3302 -48.5126

H -16.8468 -0.6092 -49.3126

H -16.8838 -0.8352 -47.5646

C -17.5908 -2.5272 -48.6866

H -17.3598 -3.0252 -49.6306

H -17.4058 -3.2442 -47.8826

C -19.0588 -2.1242 -48.6716

H -19.2648 -1.4072 -49.4626

H -19.6968 -2.9922 -48.8226

H -19.3198 -1.6662 -47.7206

C -11.1588 0.2148 -44.0636

H -10.9178 -0.3662 -44.9636

H -10.9818 -0.4222 -43.1936

C -12.6138 0.6698 -44.0996

H -12.8308 1.2148 -43.1796

H -12.7388 1.3668 -44.9316

C -13.5618 -0.5112 -44.2566

H -13.3978 -1.2132 -43.4356

H -13.3268 -1.0382 -45.1826

C -15.0248 -0.0752 -44.2716

H -15.2558 0.4568 -43.3456

H -15.1868 0.6228 -45.0966

C -15.9658 -1.2662 -44.4236

H -15.7878 -1.9692 -43.6046

H -15.7368 -1.7882 -45.3546

C -17.4368 -0.8602 -44.4206

H -17.6698 -0.3402 -43.4896

H -17.6238 -0.1622 -45.2406

C -18.3488 -2.0742 -44.5696

H -18.1458 -2.7742 -43.7546

H -18.1138 -2.5882 -45.5036

C -19.8298 -1.7102 -44.5536

H -20.0718 -1.2122 -43.6116

H -20.0418 -1.0042 -45.3606

C -20.7088 -2.9472 -44.7166

H -20.4698 -3.6592 -43.9216

H -20.4758 -3.4322 -45.6666

C -22.1988 -2.6252 -44.6646

H -22.4328 -2.1432 -43.7126

H -22.4478 -1.9202 -45.4616

C -23.0518 -3.8822 -44.8166

H -22.7688 -4.6002 -44.0426

H -22.8408 -4.3452 -45.7836

C -24.5418 -3.5892 -44.7066

H -24.8488 -2.8782 -45.4706

H -25.1208 -4.5012 -44.8356

H -24.7768 -3.1672 -43.7316

C -10.6048 0.8608 -39.9216

H -10.2928 -0.1182 -39.5286

H -9.9808 1.6378 -39.4636

C -12.0688 1.1358 -39.5866

H -12.1608 1.2268 -38.5016

H -12.3438 2.0928 -40.0346

C -13.0118 0.0508 -40.0996

H -12.8458 -0.0852 -41.1686

H -12.7828 -0.8942 -39.6026

C -14.4718 0.4238 -39.8486

H -14.7048 1.3448 -40.3886

H -14.6138 0.6268 -38.7826

C -15.4378 -0.6742 -40.2836

H -15.2378 -1.5782 -39.7056

H -15.2658 -0.9092 -41.3346

C -16.8998 -0.2732 -40.0956

H -17.1258 0.5848 -40.7326

H -17.0618 0.0368 -39.0606

C -17.8478 -1.4212 -40.4326

H -17.6538 -1.7632 -41.4506

H -17.6448 -2.2592 -39.7616

C -19.3168 -1.0252 -40.3086

H -19.5408 -0.2322 -41.0266

H -19.5018 -0.6222 -39.3096

C -20.2438 -2.2132 -40.5536

H -20.0318 -2.9882 -39.8156

H -20.0388 -2.6342 -41.5396

C -21.7198 -1.8322 -40.4666

H -21.9188 -1.3632 -39.4996

H -21.9508 -1.0952 -41.2406

C -22.6298 -3.0462 -40.6346

H -22.4118 -3.7692 -39.8446

H -22.4158 -3.5282 -41.5896

C -24.1048 -2.6692 -40.5786

H -24.3458 -1.9512 -41.3586

H -24.7298 -3.5492 -40.7156

H -24.3488 -2.2212 -39.6176

O -10.3268 1.3688 -44.0296

O -8.0518 1.8238 -45.2176

O -10.4628 0.8808 -41.3266

O -4.4538 1.5628 -41.7916

C 2.9142 0.0498 -39.9586

C 2.9942 -0.6012 -38.7406

H 2.0832 -0.9072 -38.2536

C 4.2372 -0.8232 -38.1586

C 5.4072 -0.4122 -38.8166

C 5.3162 0.2198 -40.0646

C 4.0642 0.4588 -40.6136

H 3.9712 0.9628 -41.5616

C 3.3142 -1.6882 -36.1416

H 2.6572 -2.4272 -36.6246

H 2.7422 -0.7622 -35.9846

C 3.8012 -2.2362 -34.8016

H 4.2462 -3.2182 -34.9706

H 2.9192 -2.3642 -34.1686

C 4.8212 -1.3252 -34.1046

H 4.6242 -0.2862 -34.3736

H 4.7002 -1.4112 -33.0206

C 6.2592 -1.6852 -34.4756

H 6.3612 -1.6702 -35.5596

H 6.4622 -2.7022 -34.1286

C 7.2952 -0.7462 -33.8586

H 7.2132 0.2398 -34.3216

H 7.0972 -0.6232 -32.7906

C 8.7112 -1.2862 -34.0546

H 8.8382 -1.5732 -35.1006

H 8.8372 -2.1862 -33.4466

C 9.7932 -0.2772 -33.6786

H 9.6992 0.6058 -34.3146

H 9.6462 0.0448 -32.6446

C 11.1972 -0.8582 -33.8306

H 11.3292 -1.2212 -34.8536

H 11.3062 -1.7152 -33.1616

C 12.2842 0.1658 -33.5146

H 12.1872 1.0158 -34.1936

H 12.1392 0.5388 -32.4986

C 13.6892 -0.4182 -33.6386

H 13.7892 -1.2682 -32.9586

H 13.8412 -0.7902 -34.6546

C 14.7682 0.6118 -33.3116

H 14.6072 0.9908 -32.3006

H 14.6802 1.4558 -33.9986

C 16.1702 0.0228 -33.4116

H 16.2872 -0.8082 -32.7186

H 16.9192 0.7748 -33.1746

H 16.3592 -0.3452 -34.4186

C 7.3452 0.4998 -37.8216

H 7.0152 0.7978 -36.8156

H 7.1512 1.3308 -38.5066

C 8.8302 0.1628 -37.8226

H 9.0882 -0.2032 -38.8166

H 9.0032 -0.6452 -37.1106

C 9.7112 1.3558 -37.4656

H 9.5362 2.1708 -38.1726

H 9.4492 1.7218 -36.4706

C 11.1852 0.9588 -37.4956

H 11.4302 0.5788 -38.4906

H 11.3482 0.1478 -36.7836

C 12.1292 2.1098 -37.1566

H 11.9812 2.9298 -37.8646

H 11.8982 2.4918 -36.1596

C 13.5842 1.6498 -37.2036

H 13.8062 1.2728 -38.2056

H 13.7182 0.8228 -36.5036

C 14.5742 2.7588 -36.8576

H 14.4552 3.5888 -37.5586

H 14.3592 3.1408 -35.8576

C 16.0122 2.2498 -36.9106

H 16.2172 1.8658 -37.9136

H 16.1242 1.4188 -36.2096

C 17.0342 3.3308 -36.5696

H 16.9232 4.1668 -37.2646

H 16.8412 3.7098 -35.5636

C 18.4642 2.7988 -36.6436

H 18.6432 2.3848 -37.6396

H 18.5842 1.9848 -35.9246

C 19.5022 3.8788 -36.3576

H 19.3662 4.7058 -37.0576

H 19.3482 4.2708 -35.3496

C 20.9232 3.3428 -36.4816

H 21.0852 2.5258 -35.7816

H 21.6502 4.1238 -36.2716

H 21.1022 2.9688 -37.4886

C 6.4312 1.2718 -41.8926

H 5.9472 2.2478 -41.7436

H 5.8502 0.6998 -42.6296

C 7.8492 1.4628 -42.4176

H 7.7842 2.0018 -43.3646

H 8.2842 0.4818 -42.6136

C 8.7462 2.2268 -41.4476

H 8.8542 1.6448 -40.5326

H 8.2792 3.1788 -41.1866

C 10.1192 2.4818 -42.0636

H 10.5262 1.5368 -42.4316

H 10.0092 3.1468 -42.9226

C 11.1102 3.0918 -41.0766

H 10.7062 4.0248 -40.6756

H 11.2522 2.4058 -40.2386

C 12.4572 3.3618 -41.7426

H 12.8062 2.4428 -42.2216

H 12.3282 4.1118 -42.5266

C 13.5212 3.8378 -40.7566

H 13.6522 3.0808 -39.9796

H 13.1862 4.7568 -40.2696

C 14.8602 4.0838 -41.4486

H 15.1502 3.1848 -41.9966

H 14.7502 4.8898 -42.1786

C 15.9662 4.4398 -40.4596

H 15.7002 5.3568 -39.9266

H 16.0522 3.6428 -39.7176

C 17.3152 4.6268 -41.1506

H 17.2582 5.4738 -41.8386

H 17.5432 3.7378 -41.7446

C 18.4442 4.8578 -40.1496

H 18.2272 5.7528 -39.5606

H 18.4882 4.0108 -39.4626

C 19.7942 5.0188 -40.8366

H 20.0342 4.1288 -41.4146

H 20.5812 5.1738 -40.1026

H 19.7812 5.8708 -41.5126

O 6.6222 -0.6592 -38.2226

O 4.4322 -1.4222 -36.9576

O 6.4872 0.5738 -40.6646

O 0.6322 0.8078 -39.7326

C -1.4068 1.4758 -35.3746

C -0.2398 1.2838 -34.6286

C -2.6218 1.6718 -34.7066

C -0.2858 1.2808 -33.2516

H 0.6972 1.1398 -35.1476

C -2.6668 1.6548 -33.3326

H -3.5218 1.8248 -35.2836

C -1.5008 1.4598 -32.5906

H 0.6142 1.1408 -32.6726

H -3.5988 1.7898 -32.8046

C -2.1738 1.2448 -46.1356

C -0.9278 1.3208 -46.7666

C -3.3268 1.0958 -46.9106

C -0.8378 1.2188 -48.1366

H -0.0418 1.4568 -46.1646

C -3.2338 0.9948 -48.2806

H -4.2848 1.0468 -46.4206

C -1.9878 1.0408 -48.9046

H 0.1162 1.2708 -48.6366

H -4.1188 0.8638 -48.8826

C -5.5788 1.4118 -40.9556

H -5.4778 0.4928 -40.3606

H -5.6538 2.2648 -40.2616

C 1.5962 0.2968 -40.6346

H 1.2162 -0.6442 -41.0626

H 1.7612 1.0108 -41.4516

C -0.9218 1.4838 -8.0566

C -1.3848 1.9008 -13.6866

C -1.5398 1.9068 -14.8856

C -1.7798 1.9068 -16.2716

C -0.7178 1.8018 -17.1936

C -3.0978 1.9638 -16.7326

H -3.8818 2.0058 -15.9936

O -1.9178 1.4748 -7.3436

O 0.2992 1.3778 -7.5746

H 0.3032 1.3058 -6.5756

C -1.9548 1.6708 -27.2446

C -2.4838 2.0098 -21.6106

C -2.5088 1.9878 -20.4006

C -2.3318 1.9448 -19.0056

C -3.3998 1.9498 -18.0866

C -1.0168 1.8548 -18.5446

H -0.2408 1.8098 -19.2916

O -0.8588 1.6128 -27.7856

O -3.0868 1.6208 -27.9056

H -2.9378 1.5578 -28.8986

C -6.9558 1.3338 -18.5796

C -6.8498 0.9978 -19.9176

H -5.8688 0.8358 -20.3306

C -7.9958 0.9008 -20.6956

C -9.2608 1.1048 -20.1196

C -9.3588 1.3978 -18.7516

C -8.1988 1.5138 -17.9946

H -8.2528 1.7608 -16.9456

C -6.8248 0.3468 -22.7046

H -6.0318 1.0528 -22.4266

H -6.4948 -0.6672 -22.4316

C -7.0768 0.4348 -24.2096

H -7.3088 1.4688 -24.4696

H -6.1458 0.1608 -24.7106

C -8.2198 -0.4712 -24.6746

H -8.2278 -1.3742 -24.0636

H -8.0478 -0.7662 -25.7136

C -9.5868 0.2018 -24.5646

H -9.6988 0.6158 -23.5636

H -9.6438 1.0258 -25.2816

C -10.7248 -0.7822 -24.8216

H -10.6688 -1.5892 -24.0886

H -10.6168 -1.2242 -25.8166

C -12.0878 -0.1042 -24.7096

H -12.1418 0.4158 -23.7526

H -12.1938 0.6468 -25.4976

C -13.2318 -1.1102 -24.7986

H -13.1038 -1.8482 -24.0036

H -13.1778 -1.6412 -25.7516

C -14.6018 -0.4482 -24.6656

H -14.6458 0.1018 -23.7236

H -14.7368 0.2758 -25.4736

C -15.7348 -1.4702 -24.7066

H -15.5998 -2.1812 -23.8876

H -15.6798 -2.0342 -25.6406

C -17.1128 -0.8252 -24.5866

H -17.2558 -0.1192 -25.4096

H -17.1668 -0.2572 -23.6556

C -18.2318 -1.8622 -24.6116

H -18.1698 -2.4382 -25.5376

H -18.0908 -2.5592 -23.7816

C -19.6108 -1.2252 -24.5036

H -19.7758 -0.5272 -25.3216

H -20.3898 -1.9852 -24.5406

H -19.7078 -0.6802 -23.5676

C -11.3758 0.1448 -20.6796

H -11.3178 -0.6562 -21.4286

H -11.2378 -0.3032 -19.6936

C -12.7348 0.8358 -20.7586

H -12.8178 1.5478 -19.9356

H -12.7858 1.4018 -21.6906

C -13.8588 -0.1912 -20.7026

H -13.7248 -0.8112 -19.8126

H -13.7748 -0.8502 -21.5676

C -15.2498 0.4358 -20.6736

H -15.3438 1.0848 -19.7996

H -15.3908 1.0588 -21.5596

C -16.3308 -0.6422 -20.6316

H -16.1538 -1.2882 -19.7666

H -16.2458 -1.2652 -21.5236

C -17.7458 -0.0782 -20.5446

H -17.8458 0.5238 -19.6386

H -17.9308 0.5798 -21.3976

C -18.7838 -1.1972 -20.5316

H -18.5878 -1.8572 -19.6816

H -18.6748 -1.7952 -21.4386

C -20.2178 -0.6842 -20.4386

H -20.3458 -0.1242 -19.5096

H -20.4128 0.0038 -21.2656

C -21.2258 -1.8292 -20.4866

H -21.0388 -2.5052 -19.6476

H -21.0768 -2.3992 -21.4076

C -22.6708 -1.3442 -20.4246

H -22.8248 -0.7792 -19.5026

H -22.8618 -0.6702 -21.2626

C -23.6628 -2.5042 -20.4756

H -23.4878 -3.1642 -19.6226

H -23.4888 -3.0842 -21.3856

C -25.1088 -2.0262 -20.4496

H -25.3128 -1.3792 -21.2996

H -25.7918 -2.8712 -20.4906

H -25.3098 -1.4682 -19.5376

C -10.7688 2.0238 -16.9256

H -10.4518 1.2378 -16.2236

H -10.1528 2.9168 -16.7546

C -12.2368 2.3698 -16.6986

H -12.3298 2.8378 -15.7166

H -12.5388 3.0978 -17.4536

C -13.1458 1.1468 -16.7786

H -13.0288 0.6838 -17.7586

H -12.8378 0.4178 -16.0256

C -14.6088 1.5208 -16.5546

H -14.9018 2.2808 -17.2826

H -14.7198 1.9598 -15.5596

C -15.5398 0.3178 -16.6826

H -15.2328 -0.4552 -15.9756

H -15.4498 -0.1012 -17.6856

C -16.9978 0.6878 -16.4246

H -17.3088 1.4498 -17.1436

H -17.0918 1.1238 -15.4266

C -17.9208 -0.5222 -16.5386

H -17.7988 -0.9752 -17.5246

H -17.6298 -1.2682 -15.7966

C -19.3878 -0.1542 -16.3366

H -19.6828 0.5768 -17.0936

H -19.5128 0.3198 -15.3596

C -20.2978 -1.3752 -16.4306

H -20.0248 -2.0882 -15.6486

H -20.1418 -1.8652 -17.3936

C -21.7738 -1.0142 -16.2846

H -21.9378 -0.5302 -15.3196

H -22.0478 -0.2982 -17.0626

C -22.6738 -2.2422 -16.3936

H -22.4158 -2.9492 -15.5996

H -22.4908 -2.7362 -17.3496

C -24.1498 -1.8822 -16.2876

H -24.4268 -1.1802 -17.0716

H -24.7698 -2.7692 -16.3876

H -24.3628 -1.4192 -15.3256

O -10.3408 1.0808 -20.9586

O -8.0208 0.6488 -22.0266

O -10.6178 1.5778 -18.2566

O -4.6538 1.9348 -18.5966

C 2.8292 1.0128 -16.8946

C 2.8452 0.2898 -15.7156

H 1.9112 -0.0712 -15.3216

C 4.0462 0.0798 -15.0466

C 5.2462 0.5698 -15.5886

C 5.2242 1.2578 -16.8116

C 4.0072 1.4918 -17.4396

H 3.9642 2.0488 -18.3616

C 2.9712 -0.9572 -13.1996

H 2.4432 -1.7242 -13.7846

H 2.3112 -0.0842 -13.0906

C 3.3132 -1.5152 -11.8196

H 3.8882 -2.4332 -11.9456

H 2.3642 -1.7672 -11.3386

C 4.0962 -0.5312 -10.9446

H 3.7592 0.4868 -11.1526

H 3.8882 -0.7362 -9.8906

C 5.6022 -0.6292 -11.1816

H 5.8022 -0.5392 -12.2486

H 5.9422 -1.6172 -10.8606

C 6.3992 0.4318 -10.4266

H 6.1672 1.4198 -10.8326

H 6.1082 0.4368 -9.3716

C 7.8982 0.1668 -10.5476

H 8.1422 0.0048 -11.5996

H 8.1422 -0.7512 -10.0056

C 8.7562 1.3058 -10.0056

H 8.5552 2.2148 -10.5766

H 8.4842 1.5078 -8.9666

C 10.2442 0.9718 -10.0896

H 10.4962 0.7228 -11.1236

H 10.4502 0.0908 -9.4776

C 11.1282 2.1228 -9.6226

H 10.9512 2.9968 -10.2546

H 10.8522 2.3998 -8.6016

C 12.6102 1.7578 -9.6666

H 12.7872 0.8898 -9.0276

H 12.8812 1.4778 -10.6876

C 13.5032 2.9068 -9.2086

H 13.2152 3.2068 -8.1976

H 13.3452 3.7698 -9.8606

C 14.9772 2.5198 -9.2246

H 15.1572 1.6768 -8.5616

H 15.5972 3.3518 -8.8986

H 15.2842 2.2378 -10.2286

C 7.1062 1.4888 -14.4406

H 6.7532 1.7208 -13.4256

H 6.8992 2.3498 -15.0826

C 8.6002 1.1948 -14.4336

H 8.8912 0.9138 -15.4466

H 8.7822 0.3378 -13.7836

C 9.4422 2.3768 -13.9616

H 9.2502 3.2448 -14.5966

H 9.1602 2.6448 -12.9416

C 10.9292 2.0308 -14.0066

H 11.1982 1.7508 -15.0276

H 11.1112 1.1618 -13.3696

C 11.8292 3.1748 -13.5476

H 11.6652 4.0498 -14.1806

H 11.5702 3.4538 -12.5246

C 13.3012 2.7728 -13.6056

H 13.5552 2.4908 -14.6306

H 13.4552 1.8918 -12.9776

C 14.2392 3.8828 -13.1426

H 14.1092 4.7618 -13.7786

H 13.9832 4.1728 -12.1226

C 15.6982 3.4348 -13.1876

H 15.9512 3.1428 -14.2106

H 15.8232 2.5528 -12.5556

C 16.6612 4.5238 -12.7236

H 16.5512 5.4018 -13.3656

H 16.4052 4.8238 -11.7056

C 18.1122 4.0488 -12.7616

H 18.3502 3.6988 -13.7706

H 18.2322 3.1998 -12.0846

C 19.0952 5.1478 -12.3686

H 18.9582 6.0078 -13.0276

H 18.8812 5.4748 -11.3486

C 20.5392 4.6688 -12.4566

H 20.7002 3.8198 -11.7966

H 21.2252 5.4618 -12.1696

H 20.7762 4.3618 -13.4736

C 6.4292 2.3898 -18.5276

H 5.9322 3.3598 -18.3876

H 5.8912 1.8278 -19.3046

C 7.8692 2.5978 -18.9786

H 7.8462 3.1338 -19.9286

H 8.3242 1.6208 -19.1526

C 8.7122 3.3718 -17.9696

H 8.7832 2.7938 -17.0476

H 8.2232 4.3208 -17.7326

C 10.1082 3.6388 -18.5236

H 10.5402 2.6958 -18.8666

H 10.0302 4.2958 -19.3926

C 11.0492 4.2678 -17.4996

H 10.6242 5.2048 -17.1346

H 11.1532 3.5958 -16.6456

C 12.4262 4.5308 -18.1056

H 12.8022 3.6038 -18.5446

H 12.3312 5.2608 -18.9136

C 13.4362 5.0378 -17.0816

H 13.5312 4.3038 -16.2786

H 13.0742 5.9678 -16.6376

C 14.8082 5.2718 -17.7106

H 15.1322 4.3568 -18.2126

H 14.7322 6.0508 -18.4726

C 15.8572 5.6708 -16.6756

H 15.5572 6.6038 -16.1926

H 15.9062 4.9008 -15.9026

C 17.2412 5.8438 -17.2966

H 17.2132 6.6508 -18.0326

H 17.5172 4.9268 -17.8256

C 18.3042 6.1508 -16.2456

H 18.0412 7.0748 -15.7246

H 18.3172 5.3468 -15.5076

C 19.6922 6.2908 -16.8586

H 19.9782 5.3728 -17.3666

H 20.4312 6.5028 -16.0886

H 19.7102 7.1018 -17.5836

O 6.4192 0.3328 -14.9136

O 4.1562 -0.5762 -13.8646

O 6.4232 1.6688 -17.3116

O 0.5322 1.6428 -16.6946

C -1.2458 1.8378 -12.2876

C 0.0102 1.6568 -11.6976

C -2.3798 1.9278 -11.4706

C 0.1282 1.5478 -10.3296

H 0.8832 1.6048 -12.3316

C -2.2598 1.8148 -10.1056

H -3.3478 2.0748 -11.9256

C -1.0068 1.6198 -9.5206

H 1.0962 1.4048 -9.8736

H -3.1278 1.8678 -9.4656

C -2.3838 1.9878 -23.0146

C -1.1198 1.8278 -23.5956

C -3.5048 2.1008 -23.8416

C -0.9878 1.7448 -24.9616

H -0.2538 1.7588 -22.9536

C -3.3718 2.0048 -25.2096

H -4.4758 2.2608 -23.3996

C -2.1138 1.8108 -25.7826

H -0.0198 1.6158 -25.4206

H -4.2358 2.0778 -25.8506

C -5.7098 1.5208 -17.7526

H -5.4338 0.5748 -17.2626

H -5.8998 2.2738 -16.9746

C 1.5362 1.2658 -17.6156

H 1.2182 0.3508 -18.1396

H 1.6892 2.0598 -18.3586

C -2.1758 -1.1782 -19.8116

C -2.0958 -1.4682 -25.4556

C -2.0768 -1.5392 -26.6616

C -2.1698 -1.5922 -28.0616

C -1.0148 -1.5852 -28.8686

C -3.4388 -1.6462 -28.6366

H -4.2748 -1.6762 -27.9586

O -3.2208 -1.1122 -19.1796

O -0.9938 -1.1932 -19.2386

H -1.0788 -1.1562 -18.2336

C -2.4768 -1.5912 -39.0816

C -2.5168 -1.5172 -33.4306

C -2.5468 -1.5562 -32.2216

C -2.4598 -1.5932 -30.8206

C -3.6138 -1.6582 -30.0116

C -1.1868 -1.5742 -30.2426

H -0.3428 -1.5432 -30.9126

O -1.4268 -1.6822 -39.7096

O -3.6508 -1.5182 -39.6626

H -3.5828 -1.5692 -40.6726

C -7.2378 -1.9092 -30.5606

C -7.3038 -1.9802 -31.9396

H -6.3908 -2.1032 -32.4986

C -8.5378 -1.8592 -32.5716

C -9.7068 -1.6802 -31.8176

C -9.6318 -1.6332 -30.4186

C -8.3928 -1.7602 -29.8056

H -8.3108 -1.7212 -28.7306

C -7.7158 -2.2802 -34.7786

H -6.8078 -1.6722 -34.6486

H -7.4668 -3.3282 -34.5606

C -8.2168 -2.1432 -36.2156

H -8.4278 -1.0912 -36.4136

H -7.4018 -2.4562 -36.8736

C -9.4698 -2.9782 -36.4976

H -9.4048 -3.9182 -35.9486

H -9.5018 -3.2112 -37.5676

C -10.7708 -2.2732 -36.1136

H -10.7178 -1.9642 -35.0716

H -10.8928 -1.3732 -36.7236

C -11.9718 -3.1952 -36.3166

H -11.8478 -4.0692 -35.6746

H -11.9868 -3.5482 -37.3516

C -13.2998 -2.5102 -36.0006

H -13.2868 -2.1642 -34.9666

H -13.4178 -1.6302 -36.6376

C -14.4898 -3.4442 -36.2106

H -14.3718 -4.3262 -35.5766

H -14.4968 -3.7972 -37.2446

C -15.8308 -2.7822 -35.9016

H -15.8468 -2.4662 -34.8566

H -15.9468 -1.8872 -36.5176

C -16.9978 -3.7312 -36.1626

H -16.8668 -4.6362 -35.5636

H -16.9858 -4.0392 -37.2116

C -18.3518 -3.1072 -35.8376

H -18.4948 -2.2102 -36.4446

H -18.3678 -2.8022 -34.7896

C -19.5008 -4.0792 -36.0946

H -19.4798 -4.3952 -37.1406

H -19.3568 -4.9752 -35.4866

C -20.8578 -3.4652 -35.7776

H -21.0298 -2.5832 -36.3896

H -21.6568 -4.1782 -35.9716

H -20.9078 -3.1712 -34.7316

C -11.9318 -2.4002 -32.2516

H -11.9888 -3.0822 -33.1086

H -11.7198 -2.9972 -31.3616

C -13.2428 -1.6352 -32.0846

H -13.1738 -1.0122 -31.1926

H -13.3648 -0.9702 -32.9426

C -14.4338 -2.5812 -31.9836

H -14.3068 -3.2262 -31.1096

H -14.4548 -3.2272 -32.8636

C -15.7658 -1.8432 -31.8726

H -15.7618 -1.2122 -30.9816

H -15.8888 -1.1872 -32.7386

C -16.9398 -2.8162 -31.8036

H -16.8318 -3.4442 -30.9156

H -16.9148 -3.4752 -32.6746

C -18.2908 -2.1082 -31.7496

H -18.3218 -1.4502 -30.8796

H -18.4078 -1.4822 -32.6386

C -19.4478 -3.1022 -31.6736

H -19.3458 -3.7002 -30.7656

H -19.3948 -3.7842 -32.5256

C -20.8098 -2.4122 -31.6646

H -20.8598 -1.7222 -30.8196

H -20.9218 -1.8212 -32.5776

C -21.9558 -3.4152 -31.5656

H -21.8598 -3.9762 -30.6326

H -21.8848 -4.1302 -32.3896

C -23.3238 -2.7392 -31.6056

H -23.3928 -2.0132 -30.7916

H -23.4278 -2.1902 -32.5446

C -24.4648 -3.7452 -31.4816

H -24.3718 -4.2782 -30.5326

H -24.3838 -4.4792 -32.2866

C -25.8298 -3.0742 -31.5486

H -25.9528 -2.5532 -32.4956

H -26.6248 -3.8112 -31.4576

H -25.9418 -2.3502 -30.7446

C -10.7628 -1.1072 -28.3766

H -10.5238 -1.9962 -27.7746

H -9.9938 -0.3442 -28.1976

C -12.1308 -0.5542 -27.9866

H -12.0688 -0.1492 -26.9756

H -12.3718 0.2638 -28.6696

C -13.2238 -1.6152 -28.0596

H -13.1828 -2.0942 -29.0386

H -13.0368 -2.3812 -27.3026

C -14.6128 -1.0212 -27.8526

H -14.8078 -0.2822 -28.6336

H -14.6478 -0.5012 -26.8926

C -15.7008 -2.0902 -27.8886

H -15.5398 -2.7952 -27.0676

H -15.6258 -2.6522 -28.8206

C -17.0988 -1.4902 -27.7736

H -17.2708 -0.8162 -28.6156

H -17.1618 -0.8962 -26.8596

C -18.1848 -2.5612 -27.7536

H -18.1058 -3.1762 -28.6526

H -18.0268 -3.2152 -26.8926

C -19.5838 -1.9562 -27.6776

H -19.7618 -1.3502 -28.5696

H -19.6438 -1.2932 -26.8126

C -20.6678 -3.0242 -27.5666

H -20.5098 -3.5992 -26.6506

H -20.5838 -3.7122 -28.4116

C -22.0708 -2.4232 -27.5466

H -22.1608 -1.7432 -26.6956

H -22.2228 -1.8362 -28.4556

C -23.1558 -3.4932 -27.4566

H -23.0178 -4.0722 -26.5396

H -23.0538 -4.1792 -28.3006

C -24.5538 -2.8892 -27.4676

H -24.7138 -2.3192 -28.3796

H -25.3108 -3.6692 -27.4146

H -24.6888 -2.2212 -26.6186

O -10.8758 -1.4792 -32.4996

O -8.7348 -1.8452 -33.9126

O -10.8028 -1.4442 -29.7486

O -4.8148 -1.7362 -30.6346

C 2.5602 -1.5902 -28.0926

C 2.5372 -0.9102 -26.8856

H 1.6272 -0.4102 -26.5976

C 3.6762 -0.8762 -26.0846

C 4.8452 -1.5112 -26.5236

C 4.8622 -2.1892 -27.7476

C 3.7082 -2.2382 -28.5206

H 3.7002 -2.7722 -29.4576

C 2.6382 0.4058 -24.3626

H 1.7682 -0.2632 -24.4016

H 2.4142 1.3028 -24.9586

C 2.9022 0.7918 -22.9096

H 3.0942 -0.1182 -22.3376

H 1.9832 1.2428 -22.5306

C 4.0752 1.7618 -22.7306

H 4.1772 2.3748 -23.6286

H 3.8602 2.4368 -21.8986

C 5.3992 1.0508 -22.4506

H 5.5922 0.3218 -23.2376

H 5.3162 0.5068 -21.5056

C 6.5572 2.0438 -22.3566

H 6.7302 2.4878 -23.3396

H 6.2802 2.8548 -21.6766

C 7.8502 1.4018 -21.8576

H 8.1092 0.5598 -22.5016

H 7.6912 1.0108 -20.8496

C 9.0062 2.3998 -21.8386

H 9.2062 2.7348 -22.8586

H 8.7152 3.2798 -21.2586

C 10.2842 1.8138 -21.2446

H 10.5842 0.9368 -21.8216

H 10.0892 1.4868 -20.2206

C 11.4242 2.8298 -21.2426

H 11.6142 3.1558 -22.2676

H 11.1232 3.7088 -20.6676

C 12.7132 2.2648 -20.6516

H 12.5292 1.9418 -19.6246

H 13.0202 1.3858 -21.2236

C 13.8442 3.2908 -20.6656

H 13.5452 4.1658 -20.0846

H 14.0182 3.6188 -21.6916

C 15.1352 2.7228 -20.0896

H 14.9852 2.4118 -19.0586

H 15.9272 3.4678 -20.1126

H 15.4612 1.8558 -20.6616

C 6.7962 -0.3112 -26.0566

H 6.3242 0.5878 -25.6356

H 6.8772 -0.1782 -27.1446

C 8.1752 -0.5252 -25.4506

H 8.6132 -1.4202 -25.8956

H 8.0532 -0.7082 -24.3816

C 9.0982 0.6698 -25.6666

H 9.1842 0.8778 -26.7356

H 8.6642 1.5528 -25.1926

C 10.4862 0.4118 -25.0846

H 10.9302 -0.4532 -25.5816

H 10.3892 0.1688 -24.0246

C 11.4182 1.6108 -25.2396

H 11.5042 1.8698 -26.2976

H 10.9882 2.4728 -24.7246

C 12.8062 1.3238 -24.6726

H 13.2502 0.4828 -25.2106

H 12.7112 1.0308 -23.6246

C 13.7372 2.5298 -24.7726

H 13.8102 2.8458 -25.8166

H 13.3112 3.3638 -24.2086

C 15.1322 2.2148 -24.2386

H 15.5672 1.4038 -24.8256

H 15.0522 1.8678 -23.2066

C 16.0592 3.4258 -24.2906

H 16.1152 3.7938 -25.3186

H 15.6442 4.2308 -23.6786

C 17.4642 3.0898 -23.7966

H 17.8662 2.2638 -24.3876

H 17.4112 2.7578 -22.7566

C 18.4152 4.2788 -23.8976

H 18.4512 4.6238 -24.9336

H 18.0332 5.1018 -23.2886

C 19.8212 3.9158 -23.4376

H 19.8102 3.5808 -22.4026

H 20.4862 4.7728 -23.5136

H 20.2262 3.1128 -24.0506

C 6.2472 -3.1722 -29.4226

H 5.9282 -2.3872 -30.1226

H 5.6612 -4.0792 -29.6266

C 7.7362 -3.4522 -29.6016

H 7.9082 -3.7292 -30.6406

H 8.0162 -4.2942 -28.9676

C 8.5812 -2.2352 -29.2326

H 8.3382 -1.9472 -28.2096

H 8.3202 -1.4022 -29.8896

C 10.0792 -2.5052 -29.3326

H 10.3412 -3.3492 -28.6906

H 10.3352 -2.7812 -30.3586

C 10.8852 -1.2792 -28.9136

H 10.6292 -0.4422 -29.5676

H 10.6012 -0.9992 -27.8966

C 12.3922 -1.5092 -28.9646

H 12.6572 -2.3312 -28.2946

H 12.6812 -1.8032 -29.9756

C 13.1632 -0.2562 -28.5596

H 12.8502 0.0478 -27.5586

H 12.9102 0.5588 -29.2426

C 14.6742 -0.4692 -28.5696

H 14.9292 -1.2652 -27.8666

H 14.9882 -0.7962 -29.5626

C 15.4312 0.7998 -28.1906

H 15.2032 1.5898 -28.9116

H 15.0892 1.1418 -27.2116

C 16.9412 0.5808 -28.1436

H 17.2922 0.2508 -29.1246

H 17.1652 -0.2152 -27.4306

C 17.6942 1.8438 -27.7346

H 17.5252 2.6238 -28.4806

H 17.2982 2.2048 -26.7846

C 19.1912 1.5938 -27.5916

H 19.3802 0.8378 -26.8326

H 19.7062 2.5068 -27.3016

H 19.6122 1.2448 -28.5326

O 5.9982 -1.4512 -25.7666

O 3.7752 -0.2432 -24.8876

O 6.0492 -2.7412 -28.0926

O 0.1722 -1.5792 -28.2186

C -2.1068 -1.3962 -24.0536

C -0.9108 -1.4632 -23.3326

C -3.3198 -1.2602 -23.3706

C -0.9258 -1.3922 -21.9596

H 0.0182 -1.5792 -23.8716

C -3.3308 -1.1922 -21.9966

H -4.2398 -1.2142 -23.9326

C -2.1358 -1.2542 -21.2816

H -0.0068 -1.4452 -21.3966

H -4.2528 -1.0922 -21.4476

C -2.5068 -1.5082 -34.8356

C -1.3178 -1.7442 -35.5336

C -3.6898 -1.2802 -35.5456

C -1.3168 -1.7642 -36.9076

H -0.4068 -1.9132 -34.9786

C -3.6878 -1.3052 -36.9196

H -4.5998 -1.0892 -34.9976

C -2.5018 -1.5502 -37.6126

H -0.4108 -1.9542 -37.4616

H -4.5988 -1.1402 -37.4736

C -5.9378 -1.9602 -29.8036

H -5.8318 -2.9412 -29.3146

H -5.9818 -1.1872 -29.0246

C 1.3512 -1.5792 -28.9916

H 1.3722 -2.4582 -29.6506

H 1.3822 -0.6722 -29.6176

C -2.4298 -1.7582 -42.3556

C -2.3438 -2.1402 -47.9936

C -2.3208 -2.2332 -49.1986

C -2.3908 -2.2942 -50.5996

C -1.2218 -2.2462 -51.3846

C -3.6468 -2.3892 -51.1986

H -4.4978 -2.4532 -50.5406

O -3.4798 -1.6652 -41.7346

O -1.2528 -1.7852 -41.7736

H -1.3418 -1.7172 -40.7706

C -2.5418 -2.0202 -61.6236

C -2.6268 -2.1842 -55.9726

C -2.6898 -2.2372 -54.7666

C -2.6288 -2.2852 -53.3636

C -3.7938 -2.3942 -52.5766

C -1.3678 -2.2312 -52.7616

H -0.5148 -2.1652 -53.4176

O -1.4928 -1.9562 -62.2586

O -3.7218 -2.0302 -62.1986

H -3.6688 -1.9592 -63.1986

C -7.3748 -2.6672 -53.3026

C -7.3318 -2.8252 -54.6756

H -6.3878 -3.0572 -55.1386

C -8.4868 -2.6392 -55.4286

C -9.6938 -2.3132 -54.7926

C -9.7398 -2.2052 -53.3946

C -8.5728 -2.3842 -52.6626

H -8.5808 -2.2832 -51.5886

C -7.5048 -3.3052 -57.4956

H -6.5468 -2.8192 -57.2656

H -7.4358 -4.3642 -57.2086

C -7.7778 -3.1832 -58.9936

H -7.8448 -2.1262 -59.2576

H -6.9108 -3.6062 -59.5096

C -9.0558 -3.9042 -59.4286

H -9.1668 -4.8162 -58.8386

H -8.9638 -4.1892 -60.4816

C -10.3118 -3.0512 -59.2606

H -10.3388 -2.6502 -58.2476

H -10.2748 -2.2082 -59.9576

C -11.5698 -3.8772 -59.5146

H -11.5858 -4.7022 -58.7996

H -11.5278 -4.3132 -60.5166

C -12.8528 -3.0612 -59.3786

H -12.8548 -2.5482 -58.4156

H -12.8818 -2.2942 -60.1586

C -14.0928 -3.9452 -59.4886

H -14.0738 -4.6832 -58.6826

H -14.0578 -4.5002 -60.4296

C -15.4018 -3.1632 -59.4176

H -15.4558 -2.6332 -58.4646

H -15.4248 -2.4142 -60.2126

C -16.6088 -4.0892 -59.5536

H -16.5648 -4.8482 -58.7686

H -16.5538 -4.6142 -60.5116

C -17.9398 -3.3492 -59.4596

H -17.9988 -2.6052 -60.2566

H -17.9918 -2.8192 -58.5066

C -19.1248 -4.3052 -59.5706

H -19.0708 -4.8372 -60.5226

H -19.0578 -5.0512 -58.7756

C -20.4628 -3.5832 -59.4686

H -20.5558 -2.8402 -60.2566

H -21.2868 -4.2872 -59.5646

H -20.5518 -3.0782 -58.5096

C -11.8888 -2.9272 -55.4556

H -11.8948 -3.5802 -56.3376

H -11.7758 -3.5582 -54.5706

C -13.1838 -2.1242 -55.3766

H -13.1638 -1.5162 -54.4716

H -13.2248 -1.4452 -56.2306

C -14.4028 -3.0402 -55.3766

H -14.3418 -3.7162 -54.5206

H -14.3878 -3.6542 -56.2786

C -15.7228 -2.2752 -55.3126

H -15.7548 -1.6752 -54.4006

H -15.7848 -1.5882 -56.1606

C -16.9158 -3.2272 -55.3406

H -16.8558 -3.8982 -54.4796

H -16.8598 -3.8432 -56.2406

C -18.2578 -2.5022 -55.3126

H -18.3248 -1.8942 -54.4076

H -18.3248 -1.8262 -56.1686

C -19.4248 -3.4862 -55.3516

H -19.3698 -4.1402 -54.4776

H -19.3338 -4.1152 -56.2396

C -20.7818 -2.7892 -55.3666

H -20.8838 -2.1722 -54.4716

H -20.8378 -2.1242 -56.2316

C -21.9318 -3.7912 -55.4246

H -21.8978 -4.4262 -54.5356

H -21.8048 -4.4372 -56.2966

C -23.2948 -3.1082 -55.5006

H -23.4198 -2.4512 -54.6376

H -23.3358 -2.4862 -56.3976

C -24.4398 -4.1182 -55.5376

H -24.4168 -4.7202 -54.6266

H -24.2978 -4.7902 -56.3856

C -25.7978 -3.4392 -55.6536

H -25.8518 -2.8462 -56.5646

H -26.5958 -4.1782 -55.6786

H -25.9708 -2.7782 -54.8066

C -11.0168 -1.5462 -51.4826

H -10.8338 -2.4242 -50.8456

H -10.2568 -0.7852 -51.2616

C -12.4058 -0.9752 -51.2086

H -12.4078 -0.5252 -50.2146

H -12.5998 -0.1882 -51.9396

C -13.4998 -2.0362 -51.3016

H -13.4168 -2.5442 -52.2626

H -13.3498 -2.7812 -50.5156

C -14.8958 -1.4342 -51.1696

H -15.0528 -0.7102 -51.9726

H -14.9728 -0.8962 -50.2226

C -15.9838 -2.5032 -51.2366

H -15.8678 -3.1902 -50.3946

H -15.8638 -3.0842 -52.1526

C -17.3878 -1.9042 -51.2036

H -17.5078 -1.2252 -52.0516

H -17.5098 -1.3152 -50.2916

C -18.4698 -2.9782 -51.2606

H -18.3238 -3.5902 -52.1536

H -18.3758 -3.6342 -50.3926

C -19.8738 -2.3812 -51.2836

H -19.9758 -1.7332 -52.1576

H -20.0188 -1.7612 -50.3966

C -20.9538 -3.4592 -51.3276

H -20.8638 -4.0952 -50.4426

H -20.7988 -4.0892 -52.2056

C -22.3598 -2.8662 -51.3766

H -22.5238 -2.2492 -50.4896

H -22.4448 -2.2162 -52.2506

C -23.4358 -3.9452 -51.4486

H -23.3558 -4.5932 -50.5726

H -23.2638 -4.5622 -52.3336

C -24.8368 -3.3522 -51.5136

H -24.9368 -2.7092 -52.3846

H -25.5848 -4.1382 -51.5816

H -25.0428 -2.7572 -50.6256

O -10.7818 -2.0422 -55.5786

O -8.5548 -2.6962 -56.7816

O -10.9518 -1.9102 -52.8476

O -4.9748 -2.4962 -53.2326

C 2.3552 -2.2072 -50.6066

C 2.3602 -1.5202 -49.4036

H 1.4612 -1.0092 -49.1016

C 3.5172 -1.4912 -48.6286

C 4.6732 -2.1352 -49.0866

C 4.6632 -2.8122 -50.3126

C 3.4912 -2.8612 -51.0566

H 3.4612 -3.3982 -51.9916

C 2.5582 -0.1432 -46.9056

H 1.6542 -0.7662 -46.9146

H 2.3702 0.7508 -47.5166

C 2.8712 0.2528 -45.4646

H 3.0692 -0.6542 -44.8926

H 1.9702 0.7198 -45.0626

C 4.0592 1.2108 -45.3276

H 4.1062 1.8558 -46.2076

H 3.9012 1.8558 -44.4596

C 5.3932 0.4838 -45.1586

H 5.5292 -0.2172 -45.9806

H 5.3672 -0.0952 -44.2326

C 6.5632 1.4648 -45.1036

H 6.6572 1.9708 -46.0676

H 6.3522 2.2328 -44.3536

C 7.8852 0.7828 -44.7596

H 8.0932 0.0038 -45.4936

H 7.7952 0.2988 -43.7846

C 9.0462 1.7738 -44.7256

H 9.1772 2.2108 -45.7186

H 8.8052 2.5918 -44.0416

C 10.3562 1.1258 -44.2826

H 10.6072 0.3128 -44.9666

H 10.2232 0.6948 -43.2886

C 11.5072 2.1288 -44.2466

H 11.6372 2.5648 -45.2396

H 11.2562 2.9428 -43.5626

C 12.8212 1.4918 -43.8026

H 12.6922 1.0538 -42.8096

H 13.0802 0.6818 -44.4886

C 13.9652 2.5008 -43.7626

H 13.7112 3.3098 -43.0746

H 14.0942 2.9398 -44.7546

C 15.2742 1.8578 -43.3216

H 15.1662 1.4228 -42.3306

H 16.0732 2.5948 -43.2886

H 15.5642 1.0688 -44.0116

C 6.7042 -1.0262 -48.7116

H 6.3642 -0.0902 -48.2426

H 6.6882 -0.8842 -49.7996

C 8.1112 -1.3742 -48.2466

H 8.4512 -2.2562 -48.7916

H 8.0622 -1.6402 -47.1896

C 9.0952 -0.2252 -48.4416

H 9.1082 0.0738 -49.4936

H 8.7642 0.6388 -47.8606

C 10.5012 -0.6232 -48.0036

H 10.8572 -1.4472 -48.6266

H 10.4632 -0.9862 -46.9736

C 11.4922 0.5338 -48.0816

H 11.5062 0.9348 -49.0986

H 11.1632 1.3358 -47.4176

C 12.8992 0.0948 -47.6866

H 13.2462 -0.6772 -48.3766

H 12.8692 -0.3472 -46.6876

C 13.8922 1.2538 -47.6856

H 13.8942 1.7308 -48.6686

H 13.5722 2.0028 -46.9586

C 15.3052 0.7868 -47.3466

H 15.6322 0.0598 -48.0936

H 15.2942 0.2798 -46.3786

C 16.3022 1.9408 -47.2996

H 16.2692 2.4828 -48.2486

H 16.0132 2.6418 -46.5136

C 17.7252 1.4528 -47.0466

H 17.9952 0.7248 -47.8156

H 17.7672 0.9448 -46.0806

C 18.7422 2.5908 -47.0606

H 18.6902 3.1078 -48.0226

H 18.4882 3.3138 -46.2826

C 20.1612 2.0808 -46.8396

H 20.2382 1.5718 -45.8806

H 20.8712 2.9048 -46.8486

H 20.4392 1.3798 -47.6226

C 5.9872 -3.8802 -51.9916

H 5.6512 -3.1462 -52.7376

H 5.3862 -4.7942 -52.1046

C 7.4682 -4.1872 -52.1906

H 7.6042 -4.6342 -53.1746

H 7.7752 -4.9132 -51.4366

C 8.3222 -2.9292 -52.0586

H 8.0612 -2.4322 -51.1246

H 8.0922 -2.2462 -52.8796

C 9.8162 -3.2372 -52.0556

H 10.0392 -3.9292 -51.2406

H 10.0932 -3.7322 -52.9896

C 10.6452 -1.9682 -51.8806

H 10.4622 -1.3002 -52.7246

H 10.3202 -1.4522 -50.9756

C 12.1402 -2.2562 -51.7806

H 12.3252 -2.9032 -50.9206

H 12.4682 -2.7952 -52.6716

C 12.9592 -0.9762 -51.6336

H 12.6122 -0.4242 -50.7576

H 12.7942 -0.3412 -52.5076

C 14.4502 -1.2632 -51.4896

H 14.6122 -1.8622 -50.5896

H 14.7902 -1.8552 -52.3416

C 15.2842 0.0118 -51.3986

H 15.1582 0.5948 -52.3146

H 14.9242 0.6208 -50.5676

C 16.7652 -0.2942 -51.1956

H 17.1232 -0.9102 -52.0236

H 16.8862 -0.8732 -50.2766

C 17.6182 0.9678 -51.1016

H 17.5072 1.5488 -52.0206

H 17.2582 1.5848 -50.2756

C 19.0902 0.6398 -50.8836

H 19.2232 0.0688 -49.9666

H 19.6802 1.5498 -50.8076

H 19.4752 0.0478 -51.7116

O 5.8332 -2.0872 -48.3416

O 3.6522 -0.8572 -47.4356

O 5.8452 -3.3542 -50.6896

O -0.0428 -2.2092 -50.7196

C -2.3588 -2.0442 -46.5926

C -1.1588 -1.9562 -45.8816

C -3.5758 -2.0332 -45.9026

C -1.1728 -1.8682 -44.5086

H -0.2268 -1.9612 -46.4266

C -3.5858 -1.9412 -44.5306

H -4.4998 -2.0962 -46.4576

C -2.3868 -1.8602 -43.8236

H -0.2518 -1.8012 -43.9526

H -4.5098 -1.9322 -43.9736

C -2.6008 -2.1422 -57.3766

C -1.3958 -2.2912 -58.0726

C -3.7878 -1.9542 -58.0916

C -1.3828 -2.2492 -59.4466

H -0.4798 -2.4312 -57.5176

C -3.7768 -1.9352 -59.4646

H -4.7088 -1.8192 -57.5446

C -2.5728 -2.0772 -60.1556

H -0.4618 -2.3592 -59.9966

H -4.6938 -1.8062 -60.0166

C -6.1288 -2.7732 -52.4616

H -6.0408 -3.7842 -52.0346

H -6.2158 -2.0542 -51.6376

C 1.1382 -2.1932 -51.4926

H 1.1622 -3.0652 -52.1596

H 1.1612 -1.2792 -52.1086

C -1.8798 0.6858 -54.9706

C -2.3428 1.1028 -60.5996

C -2.4978 1.1098 -61.7996

C -2.7378 1.1098 -63.1856

C -1.6758 1.0038 -64.1076

C -4.0558 1.1668 -63.6466

H -4.8398 1.2078 -62.9066

O -2.8758 0.6778 -54.2576

O -0.6588 0.5798 -54.4886

H -0.6548 0.5088 -53.4896

C -2.9128 0.8728 -74.1586

C -3.4418 1.2118 -68.5246

C -3.4668 1.1898 -67.3146

C -3.2898 1.1468 -65.9196

C -4.3578 1.1518 -64.9996

C -1.9748 1.0568 -65.4586

H -1.1988 1.0118 -66.2056

O -1.8168 0.8158 -74.6996

O -4.0448 0.8238 -74.8196

H -3.8958 0.7608 -75.8126

C -7.9138 0.5368 -65.4936

C -7.8078 0.1998 -66.8316

H -6.8268 0.0378 -67.2446

C -8.9538 0.1028 -67.6086

C -10.2188 0.3068 -67.0336

C -10.3168 0.6008 -65.6656

C -9.1568 0.7168 -64.9076

H -9.2108 0.9628 -63.8596

C -7.7828 -0.4502 -69.6186

H -6.9898 0.2548 -69.3406

H -7.4528 -1.4642 -69.3456

C -8.0348 -0.3632 -71.1236

H -8.2668 0.6708 -71.3826

H -7.1028 -0.6372 -71.6246

C -9.1778 -1.2692 -71.5886

H -9.1858 -2.1712 -70.9776

H -9.0048 -1.5642 -72.6276

C -10.5448 -0.5962 -71.4786

H -10.6568 -0.1812 -70.4776

H -10.6018 0.2278 -72.1956

C -11.6828 -1.5792 -71.7356

H -11.6268 -2.3872 -71.0016

H -11.5748 -2.0222 -72.7306

C -13.0458 -0.9022 -71.6236

H -13.0998 -0.3822 -70.6656

H -13.1518 -0.1512 -72.4116

C -14.1898 -1.9082 -71.7126

H -14.0618 -2.6452 -70.9176

H -14.1358 -2.4392 -72.6656

C -15.5598 -1.2462 -71.5796

H -15.6038 -0.6962 -70.6376

H -15.6948 -0.5222 -72.3876

C -16.6928 -2.2682 -71.6206

H -16.5578 -2.9792 -70.8016

H -16.6378 -2.8322 -72.5536

C -18.0708 -1.6222 -71.5006

H -18.2138 -0.9172 -72.3236

H -18.1248 -1.0542 -70.5696

C -19.1898 -2.6602 -71.5256

H -19.1278 -3.2352 -72.4516

H -19.0488 -3.3572 -70.6956

C -20.5688 -2.0232 -71.4166

H -20.7338 -1.3252 -72.2356

H -21.3478 -2.7822 -71.4546

H -20.6658 -1.4782 -70.4816

C -12.3338 -0.6522 -67.5936

H -12.2758 -1.4532 -68.3426

H -12.1958 -1.1012 -66.6076

C -13.6928 0.0388 -67.6726

H -13.7758 0.7508 -66.8496

H -13.7438 0.6038 -68.6046

C -14.8168 -0.9892 -67.6156

H -14.6818 -1.6092 -66.7266

H -14.7328 -1.6482 -68.4816

C -16.2078 -0.3622 -67.5876

H -16.3018 0.2868 -66.7136

H -16.3488 0.2618 -68.4736

C -17.2888 -1.4402 -67.5446

H -17.1118 -2.0862 -66.6806

H -17.2038 -2.0632 -68.4376

C -18.7038 -0.8762 -67.4586

H -18.8038 -0.2732 -66.5526

H -18.8888 -0.2182 -68.3116

C -19.7418 -1.9952 -67.4456

H -19.5458 -2.6542 -66.5956

H -19.6328 -2.5932 -68.3526

C -21.1758 -1.4812 -67.3526

H -21.3038 -0.9222 -66.4236

H -21.3708 -0.7942 -68.1796

C -22.1838 -2.6272 -67.4006

H -21.9968 -3.3032 -66.5616

H -22.0348 -3.1972 -68.3206

C -23.6288 -2.1422 -67.3376

H -23.7828 -1.5762 -66.4166

H -23.8198 -1.4682 -68.1766

C -24.6208 -3.3022 -67.3896

H -24.4458 -3.9622 -66.5366

H -24.4468 -3.8812 -68.2996

C -26.0658 -2.8242 -67.3626

H -26.2708 -2.1772 -68.2136

H -26.7498 -3.6692 -67.4046

H -26.2678 -2.2662 -66.4516

C -11.7268 1.2258 -63.8396

H -11.4098 0.4408 -63.1376

H -11.1098 2.1188 -63.6686

C -13.1948 1.5718 -63.6126

H -13.2878 2.0398 -62.6296

H -13.4968 2.2998 -64.3676

C -14.1038 0.3488 -63.6926

H -13.9868 -0.1142 -64.6726

H -13.7958 -0.3802 -62.9396

C -15.5668 0.7228 -63.4686

H -15.8598 1.4838 -64.1966

H -15.6778 1.1628 -62.4736

C -16.4978 -0.4802 -63.5966

H -16.1908 -1.2532 -62.8896

H -16.4078 -0.8992 -64.5996

C -17.9558 -0.1102 -63.3386

H -18.2668 0.6528 -64.0566

H -18.0488 0.3258 -62.3406

C -18.8788 -1.3202 -63.4526

H -18.7568 -1.7732 -64.4376

H -18.5878 -2.0662 -62.7106

C -20.3448 -0.9522 -63.2506

H -20.6408 -0.2212 -64.0076

H -20.4708 -0.4782 -62.2736

C -21.2558 -2.1732 -63.3436

H -20.9828 -2.8862 -62.5626

H -21.0998 -2.6632 -64.3076

C -22.7318 -1.8122 -63.1986

H -22.8958 -1.3272 -62.2326

H -23.0058 -1.0952 -63.9766

C -23.6318 -3.0402 -63.3066

H -23.3738 -3.7462 -62.5136

H -23.4488 -3.5332 -64.2636

C -25.1078 -2.6792 -63.2016

H -25.3848 -1.9782 -63.9856

H -25.7278 -3.5672 -63.3016

H -25.3208 -2.2172 -62.2396

O -11.2988 0.2838 -67.8726

O -8.9788 -0.1492 -68.9406

O -11.5758 0.7798 -65.1706

O -5.6118 1.1368 -65.5106

C 1.8712 0.2148 -63.8086

C 1.8872 -0.5082 -62.6286

H 0.9532 -0.8692 -62.2356

C 3.0882 -0.7182 -61.9606

C 4.2882 -0.2282 -62.5016

C 4.2662 0.4598 -63.7246

C 3.0492 0.6948 -64.3536

H 3.0062 1.2508 -65.2756

C 2.0132 -1.7552 -60.1136

H 1.4852 -2.5222 -60.6986

H 1.3532 -0.8822 -60.0046

C 2.3552 -2.3132 -58.7336

H 2.9302 -3.2312 -58.8596

H 1.4062 -2.5652 -58.2526

C 3.1382 -1.3282 -57.8586

H 2.8012 -0.3102 -58.0666

H 2.9302 -1.5342 -56.8046

C 4.6442 -1.4262 -58.0956

H 4.8442 -1.3372 -59.1626

H 4.9842 -2.4142 -57.7746

C 5.4412 -0.3662 -57.3406

H 5.2092 0.6218 -57.7466

H 5.1502 -0.3612 -56.2856

C 6.9402 -0.6312 -57.4616

H 7.1842 -0.7932 -58.5126

H 7.1842 -1.5482 -56.9196

C 7.7982 0.5078 -56.9196

H 7.5972 1.4168 -57.4906

H 7.5262 0.7098 -55.8806

C 9.2862 0.1738 -57.0036

H 9.5382 -0.0752 -58.0376

H 9.4922 -0.7072 -56.3916

C 10.1702 1.3258 -56.5366

H 9.9932 2.1988 -57.1676

H 9.8942 1.6028 -55.5156

C 11.6522 0.9598 -56.5806

H 11.8292 0.0918 -55.9416

H 11.9232 0.6798 -57.6016

C 12.5452 2.1098 -56.1226

H 12.2572 2.4088 -55.1116

H 12.3882 2.9718 -56.7746

C 14.0192 1.7228 -56.1386

H 14.1992 0.8788 -55.4756

H 14.6392 2.5538 -55.8126

H 14.3262 1.4398 -57.1426

C 6.1482 0.6908 -61.3546

H 5.7952 0.9228 -60.3396

H 5.9412 1.5528 -61.9966

C 7.6422 0.3968 -61.3476

H 7.9332 0.1158 -62.3606

H 7.8242 -0.4602 -60.6976

C 8.4842 1.5798 -60.8756

H 8.2922 2.4478 -61.5106

H 8.2022 1.8468 -59.8556

C 9.9712 1.2338 -60.9206

H 10.2402 0.9528 -61.9416

H 10.1532 0.3638 -60.2836

C 10.8712 2.3768 -60.4616

H 10.7072 3.2518 -61.0946

H 10.6122 2.6558 -59.4386

C 12.3432 1.9748 -60.5196

H 12.5972 1.6938 -61.5446

H 12.4972 1.0938 -59.8916

C 13.2812 3.0848 -60.0566

H 13.1512 3.9638 -60.6926

H 13.0252 3.3748 -59.0356

C 14.7402 2.6378 -60.1016

H 14.9932 2.3458 -61.1246

H 14.8652 1.7548 -59.4696

C 15.7032 3.7258 -59.6376

H 15.5932 4.6038 -60.2796

H 15.4472 4.0268 -58.6196

C 17.1542 3.2508 -59.6756

H 17.3922 2.9018 -60.6836

H 17.2742 2.4018 -58.9976

C 18.1372 4.3498 -59.2826

H 18.0002 5.2098 -59.9416

H 17.9232 4.6768 -58.2626

C 19.5812 3.8718 -59.3706

H 19.7422 3.0218 -58.7106

H 20.2672 4.6648 -59.0836

H 19.8182 3.5638 -60.3876

C 5.4712 1.5918 -65.4416

H 4.9742 2.5618 -65.3016

H 4.9332 1.0298 -66.2186

C 6.9112 1.7998 -65.8926

H 6.8882 2.3368 -66.8426

H 7.3662 0.8238 -66.0666

C 7.7542 2.5748 -64.8836

H 7.8252 1.9968 -63.9616

H 7.2652 3.5228 -64.6466

C 9.1502 2.8408 -65.4376

H 9.5822 1.8978 -65.7806

H 9.0722 3.4978 -66.3066

C 10.0912 3.4698 -64.4136

H 9.6662 4.4068 -64.0486

H 10.1952 2.7978 -63.5596

C 11.4682 3.7328 -65.0196

H 11.8442 2.8058 -65.4586

H 11.3732 4.4628 -65.8276

C 12.4782 4.2398 -63.9956

H 12.5732 3.5058 -63.1916

H 12.1162 5.1698 -63.5516

C 13.8502 4.4738 -64.6236

H 14.1752 3.5588 -65.1256

H 13.7742 5.2538 -65.3856

C 14.8992 4.8728 -63.5896

H 14.5992 5.8058 -63.1066

H 14.9482 4.1038 -62.8156

C 16.2832 5.0458 -64.2106

H 16.2552 5.8528 -64.9466

H 16.5592 4.1298 -64.7396

C 17.3462 5.3528 -63.1596

H 17.0832 6.2768 -62.6386

H 17.3592 4.5488 -62.4216

C 18.7342 5.4938 -63.7726

H 19.0202 4.5748 -64.2806

H 19.4732 5.7048 -63.0026

H 18.7522 6.3038 -64.4976

O 5.4612 -0.4652 -61.8276

O 3.1982 -1.3742 -60.7786

O 5.4652 0.8708 -64.2256

O -0.4258 0.8458 -63.6076

C -2.2038 1.0408 -59.2016

C -0.9478 0.8588 -58.6116

C -3.3378 1.1298 -58.3846

C -0.8298 0.7498 -57.2436

H -0.0748 0.8068 -59.2456

C -3.2178 1.0168 -57.0196

H -4.3058 1.2778 -58.8396

C -1.9648 0.8218 -56.4346

H 0.1382 0.6068 -56.7876

H -4.0858 1.0698 -56.3796

C -3.3418 1.1898 -69.9286

C -2.0778 1.0298 -70.5086

C -4.4628 1.3038 -70.7556

C -1.9458 0.9468 -71.8756

H -1.2118 0.9618 -69.8676

C -4.3298 1.2068 -72.1236

H -5.4338 1.4638 -70.3136

C -3.0718 1.0138 -72.6956

H -0.9778 0.8178 -72.3346

H -5.1938 1.2798 -72.7636

C -6.6678 0.7238 -64.6666

H -6.3918 -0.2232 -64.1766

H -6.8578 1.4758 -63.8886

C 0.5782 0.4678 -64.5296

H 0.2602 -0.4472 -65.0536

H 0.7312 1.2618 -65.2726

C -1.7628 -0.9232 3.0634

C -1.7508 -1.3002 -2.5756

C -1.7428 -1.3912 -3.7806

C -1.8318 -1.4522 -5.1806

C -0.6728 -1.4042 -5.9816

C -3.0958 -1.5462 -5.7636

H -3.9378 -1.6102 -5.0946

O -2.8048 -0.8302 3.6984

O -0.5788 -0.9502 3.6304

H -0.6548 -0.8832 4.6344

C -2.1268 -1.1682 -16.2016

C -2.1378 -1.3372 -10.5506

C -2.1848 -1.3912 -9.3426

C -2.1058 -1.4412 -7.9416

C -3.2608 -1.5492 -7.1396

C -0.8378 -1.3872 -7.3556

H 0.0072 -1.3212 -8.0226

O -1.0858 -1.1042 -16.8506

O -3.3138 -1.1782 -16.7606

H -3.2738 -1.1062 -17.7616

C -6.8508 -1.8212 -7.8186

C -6.8258 -1.9782 -9.1926

H -5.8878 -2.2102 -9.6676

C -7.9908 -1.7912 -9.9296

C -9.1888 -1.4652 -9.2776

C -9.2168 -1.3592 -7.8796

C -8.0398 -1.5382 -7.1626

H -8.0338 -1.4382 -6.0886

C -7.0348 -2.4552 -12.0096

H -6.0748 -1.9702 -11.7916

H -6.9628 -3.5142 -11.7246

C -7.3278 -2.3332 -13.5036

H -7.3988 -1.2752 -13.7666

H -6.4688 -2.7552 -14.0316

C -8.6118 -3.0532 -13.9226

H -8.7148 -3.9652 -13.3326

H -8.5328 -3.3372 -14.9766

C -9.8658 -2.1992 -13.7376

H -9.8788 -1.7992 -12.7246

H -9.8378 -1.3562 -14.4346

C -11.1268 -3.0242 -13.9766

H -11.1338 -3.8502 -13.2616

H -11.0978 -3.4602 -14.9796

C -12.4078 -2.2082 -13.8226

H -12.3968 -1.6972 -12.8596

H -12.4468 -1.4412 -14.6016

C -13.6488 -3.0922 -13.9176

H -13.6208 -3.8312 -13.1126

H -13.6268 -3.6472 -14.8586

C -14.9568 -2.3102 -13.8286

H -14.9988 -1.7812 -12.8746

H -14.9908 -1.5612 -14.6226

C -16.1658 -3.2352 -13.9496

H -16.1118 -3.9952 -13.1656

H -16.1238 -3.7592 -14.9086

C -17.4958 -2.4952 -13.8376

H -17.5648 -1.7512 -14.6336

H -17.5348 -1.9662 -12.8836

C -18.6828 -3.4512 -13.9336

H -18.6408 -3.9822 -14.8876

H -18.6048 -4.1982 -13.1406

C -20.0178 -2.7292 -13.8136

H -20.1208 -1.9852 -14.5996

H -20.8428 -3.4332 -13.8996

H -20.0938 -2.2252 -12.8536

C -11.3918 -2.0782 -9.9126

H -11.4098 -2.7302 -10.7946

H -11.2678 -2.7102 -9.0296

C -12.6868 -1.2752 -9.8156

H -12.6538 -0.6672 -8.9106

H -12.7388 -0.5962 -10.6686

C -13.9048 -2.1902 -9.8006

H -13.8328 -2.8682 -8.9456

H -13.9028 -2.8042 -10.7036

C -15.2238 -1.4252 -9.7196

H -15.2438 -0.8262 -8.8066

H -15.2978 -0.7382 -10.5656

C -16.4168 -2.3772 -9.7326

H -16.3468 -3.0482 -8.8726

H -16.3738 -2.9932 -10.6336

C -17.7588 -1.6522 -9.6856

H -17.8148 -1.0442 -8.7806

H -17.8368 -0.9752 -10.5406

C -18.9268 -2.6362 -9.7106

H -18.8598 -3.2902 -8.8376

H -18.8478 -3.2642 -10.6006

C -20.2838 -1.9382 -9.7076

H -20.3738 -1.3222 -8.8106

H -20.3508 -1.2722 -10.5716

C -21.4348 -2.9402 -9.7516

H -21.3888 -3.5762 -8.8636

H -21.3188 -3.5852 -10.6256

C -22.7978 -2.2572 -9.8096

H -22.9118 -1.6002 -8.9436

H -22.8508 -1.6332 -10.7046

C -23.9438 -3.2662 -9.8316

H -23.9088 -3.8692 -8.9206

H -23.8128 -3.9372 -10.6826

C -25.3038 -2.5862 -9.9296

H -25.3688 -1.9932 -10.8396

H -26.1008 -3.3262 -9.9446

H -25.4648 -1.9262 -9.0796

C -10.4688 -0.7012 -5.9506

H -10.2768 -1.5792 -5.3166

H -9.7048 0.0598 -5.7386

C -11.8538 -0.1302 -5.6576

H -11.8418 0.3198 -4.6626

H -12.0568 0.6578 -6.3856

C -12.9488 -1.1902 -5.7376

H -12.8788 -1.6972 -6.6996

H -12.7888 -1.9362 -4.9546

C -14.3428 -0.5882 -5.5856

H -14.5098 0.1358 -6.3866

H -14.4078 -0.0502 -4.6376

C -15.4318 -1.6572 -5.6396

H -15.3048 -2.3452 -4.7996

H -15.3238 -2.2372 -6.5586

C -16.8348 -1.0572 -5.5876

H -16.9658 -0.3782 -6.4336

H -16.9448 -0.4692 -4.6746

C -17.9188 -2.1312 -5.6326

H -17.7848 -2.7422 -6.5276

H -17.8128 -2.7882 -4.7656

C -19.3228 -1.5342 -5.6366

H -19.4348 -0.8852 -6.5086

H -19.4558 -0.9152 -4.7466

C -20.4028 -2.6112 -5.6666

H -20.3018 -3.2492 -4.7846

H -20.2588 -3.2402 -6.5476

C -21.8088 -2.0182 -5.6966

H -21.9618 -1.4012 -4.8076

H -21.9058 -1.3672 -6.5696

C -22.8868 -3.0972 -5.7566

H -22.7958 -3.7462 -4.8816

H -22.7268 -3.7132 -6.6436

C -24.2878 -2.5032 -5.8026

H -24.3998 -1.8592 -6.6716

H -25.0378 -3.2892 -5.8606

H -24.4818 -1.9092 -4.9116

O -10.2868 -1.1932 -10.0486

O -8.0758 -1.8472 -11.2816

O -10.4218 -1.0632 -7.3156

O -4.4498 -1.6512 -7.7796

C 2.9142 -1.3662 -5.2496

C 2.9352 -0.6802 -4.0456

H 2.0402 -0.1682 -3.7316

C 4.1012 -0.6522 -3.2866

C 5.2512 -1.2962 -3.7596

C 5.2252 -1.9712 -4.9866

C 4.0442 -2.0202 -5.7146

H 4.0012 -2.5562 -6.6496

C 3.1652 0.6948 -1.5496

H 2.2612 0.0718 -1.5476

H 2.9692 1.5898 -2.1576

C 3.4972 1.0898 -0.1126

H 3.7032 0.1818 0.4554

H 2.6022 1.5558 0.3024

C 4.6882 2.0468 0.0094

H 4.7232 2.6928 -0.8696

H 4.5412 2.6908 0.8804

C 6.0232 1.3198 0.1604

H 6.1492 0.6188 -0.6636

H 6.0102 0.7398 1.0864

C 7.1942 2.2998 0.2014

H 7.2762 2.8068 -0.7636

H 6.9932 3.0678 0.9544

C 8.5202 1.6178 0.5274

H 8.7192 0.8398 -0.2096

H 8.4432 1.1328 1.5034

C 9.6822 2.6088 0.5474

H 9.8002 3.0458 -0.4476

H 9.4502 3.4258 1.2344

C 10.9982 1.9598 0.9724

H 11.2392 1.1478 0.2844

H 10.8772 1.5278 1.9674

C 12.1492 2.9628 0.9944

H 12.2662 3.3988 -0.0006

H 11.9072 3.7758 1.6824

C 13.4692 2.3238 1.4204

H 13.3522 1.8858 2.4144

H 13.7192 1.5158 0.7304

C 14.6142 3.3328 1.4464

H 14.3682 4.1418 2.1384

H 14.7292 3.7738 0.4534

C 15.9282 2.6898 1.8694

H 15.8332 2.2538 2.8614

H 16.7282 3.4268 1.8924

H 16.2092 1.9008 1.1754

C 7.2872 -0.1872 -3.4106

H 6.9542 0.7478 -2.9356

H 7.2572 -0.0452 -4.4986

C 8.7012 -0.5372 -2.9646

H 9.0332 -1.4182 -3.5146

H 8.6652 -0.8032 -1.9066

C 9.6812 0.6128 -3.1716

H 9.6822 0.9118 -4.2226

H 9.3592 1.4758 -2.5846

C 11.0942 0.2138 -2.7516

H 11.4412 -0.6102 -3.3796

H 11.0682 -0.1502 -1.7216

C 12.0842 1.3708 -2.8416

H 12.0852 1.7728 -3.8586

H 11.7632 2.1728 -2.1726

C 13.4962 0.9308 -2.4656

H 13.8332 0.1588 -3.1606

H 13.4782 0.4878 -1.4666

C 14.4892 2.0888 -2.4766

H 14.4782 2.5678 -3.4596

H 14.1792 2.8378 -1.7456

C 15.9062 1.6218 -2.1566

H 16.2232 0.8958 -2.9086

H 15.9072 1.1148 -1.1886

C 16.9032 2.7758 -2.1216

H 16.8592 3.3188 -3.0696

H 16.6252 3.4758 -1.3306

C 18.3302 2.2868 -1.8876

H 18.5902 1.5598 -2.6606

H 18.3842 1.7788 -0.9216

C 19.3472 3.4248 -1.9146

H 19.2822 3.9428 -2.8746

H 19.1032 4.1468 -1.1316

C 20.7682 2.9148 -1.7116

H 20.8582 2.4048 -0.7546

H 21.4782 3.7378 -1.7296

H 21.0362 2.2138 -2.4986

C 6.5272 -3.0392 -6.6836

H 6.1812 -2.3042 -7.4236

H 5.9242 -3.9522 -6.7886

C 8.0052 -3.3462 -6.9016

H 8.1282 -3.7922 -7.8876

H 8.3212 -4.0732 -6.1526

C 8.8612 -2.0882 -6.7806

H 8.6122 -1.5922 -5.8416

H 8.6212 -1.4042 -7.5966

C 10.3552 -2.3972 -6.7966

H 10.5892 -3.0892 -5.9846

H 10.6192 -2.8912 -7.7346

C 11.1872 -1.1282 -6.6316

H 10.9932 -0.4592 -7.4726

H 10.8732 -0.6122 -5.7226

C 12.6822 -1.4162 -6.5506

H 12.8792 -2.0642 -5.6936

H 12.9992 -1.9552 -7.4466

C 13.5042 -0.1372 -6.4136

H 13.1682 0.4138 -5.5336

H 13.3282 0.4988 -7.2846

C 14.9972 -0.4242 -6.2896

H 15.1702 -1.0242 -5.3926

H 15.3252 -1.0162 -7.1466

C 15.8322 0.8508 -6.2086

H 15.6942 1.4348 -7.1216

H 15.4832 1.4588 -5.3716

C 17.3152 0.5438 -6.0246

H 17.6632 -0.0712 -6.8576

H 17.4482 -0.0362 -5.1086

C 18.1692 1.8058 -5.9406

H 18.0472 2.3868 -6.8576

H 17.8212 2.4218 -5.1096

C 19.6442 1.4768 -5.7426

H 19.7892 0.9048 -4.8286

H 20.2362 2.3868 -5.6736

H 20.0192 0.8858 -6.5756

O 6.4212 -1.2482 -3.0296

O 4.2532 -0.0192 -2.0946

O 6.4022 -2.5142 -5.3786

O 0.5142 -1.3672 -5.3316

C -1.7478 -1.2052 -1.1746

C -0.5378 -1.1182 -0.4786

C -2.9558 -1.1942 -0.4676

C -0.5348 -1.0312 0.8944

H 0.3862 -1.1222 -1.0356

C -2.9468 -1.1042 0.9034

H -3.8858 -1.2572 -1.0106

C -1.7398 -1.0232 1.5954

H 0.3942 -0.9652 1.4384

H -3.8638 -1.0952 1.4724

C -2.1298 -1.2942 -11.9546

C -0.9348 -1.4432 -12.6656

C -3.3258 -1.1052 -12.6526

C -0.9398 -1.4002 -14.0406

H -0.0118 -1.5842 -12.1236

C -3.3328 -1.0852 -14.0266

H -4.2398 -0.9702 -12.0946

C -2.1378 -1.2262 -14.7336

H -0.0258 -1.5102 -14.6016

H -4.2578 -0.9552 -14.5666

C -5.5938 -1.9282 -6.9946

H -5.5008 -2.9402 -6.5696

H -5.6698 -1.2092 -6.1686

C 1.6852 -1.3512 -6.1186

H 1.7002 -2.2232 -6.7876

H 1.7002 -0.4372 -6.7346

C -3.2178 -5.1642 -54.7226

C -3.5268 -4.7842 -60.3656

C -3.6498 -4.7852 -61.5696

C -3.8518 -4.7932 -62.9606

C -2.7658 -4.9112 -63.8526

C -5.1558 -4.7312 -63.4576

H -5.9598 -4.6802 -62.7396

O -4.2328 -5.1622 -54.0366

O -2.0118 -5.2742 -54.2076

H -2.0338 -5.3382 -53.2086

C -3.7318 -5.1052 -73.9326

C -4.4108 -4.7232 -68.3176

C -4.4688 -4.7372 -67.1086

C -4.3298 -4.7712 -65.7086

C -5.4218 -4.7532 -64.8186

C -3.0278 -4.8652 -65.2116

H -2.2328 -4.9202 -65.9366

O -2.6228 -5.1722 -74.4436

O -4.8468 -5.1522 -74.6236

H -4.6708 -5.2232 -75.6116

C -8.9668 -5.3512 -65.4036

C -8.8268 -5.6982 -66.7356

H -7.8368 -5.8692 -67.1206

C -9.9528 -5.7932 -67.5436

C -11.2308 -5.5782 -67.0036

C -11.3648 -5.2742 -65.6416

C -10.2238 -5.1602 -64.8536

H -10.3048 -4.9052 -63.8086

C -8.7308 -6.3682 -69.5166

H -7.9408 -5.6652 -69.2226

H -8.4148 -7.3822 -69.2276

C -8.9408 -6.2892 -71.0286

H -9.1598 -5.2562 -71.3016

H -7.9988 -6.5722 -71.5026

C -10.0778 -7.1912 -71.5176

H -10.1068 -8.0902 -70.9016

H -9.8788 -7.4952 -72.5496

C -11.4428 -6.5102 -71.4496

H -11.5788 -6.0882 -70.4546

H -11.4758 -5.6912 -72.1736

C -12.5798 -7.4882 -71.7306

H -12.5478 -8.2912 -70.9906

H -12.4478 -7.9382 -72.7196

C -13.9408 -6.8022 -71.6596

H -14.0168 -6.2752 -70.7076

H -14.0208 -6.0562 -72.4566

C -15.0878 -7.8012 -71.7736

H -14.9858 -8.5342 -70.9706

H -15.0118 -8.3402 -72.7206

C -16.4568 -7.1312 -71.6816

H -16.5228 -6.5742 -70.7456

H -16.5648 -6.4112 -72.4976

C -17.5938 -8.1462 -71.7466

H -17.4858 -8.8522 -70.9186

H -17.5178 -8.7172 -72.6746

C -18.9708 -7.4922 -71.6686

H -19.0878 -6.7912 -72.4996

H -19.0468 -6.9172 -70.7426

C -20.0958 -8.5232 -71.7166

H -20.0118 -9.1052 -72.6356

H -19.9808 -9.2152 -70.8776

C -21.4738 -7.8772 -71.6496

H -21.6118 -7.1832 -72.4766

H -22.2548 -8.6322 -71.7026

H -21.5918 -7.3252 -70.7206

C -13.3358 -6.5292 -67.6146

H -13.2628 -7.3352 -68.3566

H -13.2268 -6.9712 -66.6216

C -14.6878 -5.8302 -67.7346

H -14.7888 -5.1122 -66.9186

H -14.7108 -5.2712 -68.6716

C -15.8188 -6.8502 -67.7016

H -15.7128 -7.4652 -66.8046

H -15.7168 -7.5162 -68.5596

C -17.2068 -6.2152 -67.7146

H -17.3198 -5.5592 -66.8486

H -17.3198 -5.5972 -68.6086

C -18.2948 -7.2862 -67.6946

H -18.1458 -7.9272 -66.8206

H -18.1898 -7.9162 -68.5796

C -19.7088 -6.7132 -67.6496

H -19.8298 -6.1042 -66.7506

H -19.8668 -6.0602 -68.5116

C -20.7538 -7.8262 -67.6576

H -20.5838 -8.4812 -66.7976

H -20.6238 -8.4312 -68.5566

C -22.1858 -7.3032 -67.6066

H -22.3348 -6.7372 -66.6846

H -22.3548 -6.6202 -68.4426

C -23.1988 -8.4432 -67.6736

H -23.0388 -9.1142 -66.8256

H -23.0288 -9.0202 -68.5866

C -24.6428 -7.9502 -67.6536

H -24.8178 -7.3772 -66.7406

H -24.8068 -7.2802 -68.5016

C -25.6398 -9.1042 -67.7246

H -25.4918 -9.7592 -66.8616

H -25.4448 -9.6912 -68.6246

C -27.0828 -8.6172 -67.7396

H -27.2598 -7.9752 -68.5996

H -27.7708 -9.4582 -67.7936

H -27.3058 -8.0512 -66.8386

C -12.8188 -4.6282 -63.8586

H -12.5258 -5.4102 -63.1426

H -12.2018 -3.7372 -63.6766

C -14.2908 -4.2712 -63.6726

H -14.4068 -3.7962 -62.6966

H -14.5668 -3.5472 -64.4406

C -15.2048 -5.4892 -63.7696

H -15.0638 -5.9602 -64.7426

H -14.9208 -6.2152 -63.0026

C -16.6708 -5.1052 -63.5876

H -16.9398 -4.3482 -64.3286

H -16.8058 -4.6582 -62.5986

C -17.6048 -6.3032 -63.7326

H -17.3218 -7.0732 -63.0116

H -17.4908 -6.7302 -64.7296

C -19.0668 -5.9232 -63.5166

H -19.3548 -5.1642 -64.2486

H -19.1848 -5.4802 -62.5246

C -19.9938 -7.1292 -63.6476

H -19.8478 -7.5892 -64.6256

H -19.7278 -7.8712 -62.8916

C -21.4628 -6.7502 -63.4876

H -21.7338 -6.0232 -64.2566

H -21.6118 -6.2692 -62.5176

C -22.3788 -7.9662 -63.5966

H -22.1308 -8.6762 -62.8036

H -22.1998 -8.4642 -64.5526

C -23.8548 -7.5962 -63.4936

H -24.0428 -7.1042 -62.5366

H -24.1038 -6.8832 -64.2836

C -24.7598 -8.8192 -63.6186

H -24.5268 -9.5222 -62.8136

H -24.5538 -9.3212 -64.5656

C -26.2358 -8.4492 -63.5546

H -26.4868 -7.7522 -64.3506

H -26.8588 -9.3342 -63.6656

H -26.4718 -7.9782 -62.6026

O -12.2878 -5.6012 -67.8716

O -9.9428 -6.0542 -68.8726

O -12.6348 -5.0842 -65.1816

O -6.6618 -4.7642 -65.3626

C 0.7672 -5.7192 -63.4526

C 0.7462 -6.4332 -62.2686

H -0.1998 -6.7862 -61.8976

C 1.9272 -6.6462 -61.5666

C 3.1452 -6.1672 -62.0786

C 3.1602 -5.4882 -63.3066

C 1.9622 -5.2502 -63.9696

H 1.9472 -4.7002 -64.8956

C 0.7972 -7.6642 -59.7416

H 0.2802 -8.4322 -60.3366

H 0.1402 -6.7862 -59.6566

C 1.0982 -8.2142 -58.3496

H 1.6712 -9.1362 -58.4536

H 0.1352 -8.4582 -57.8926

C 1.8642 -7.2282 -57.4606

H 1.5382 -6.2102 -57.6846

H 1.6262 -7.4252 -56.4106

C 3.3742 -7.3372 -57.6566

H 3.6042 -7.2562 -58.7186

H 3.7002 -8.3252 -57.3196

C 4.1572 -6.2762 -56.8866

H 3.9432 -5.2902 -57.3056

H 3.8382 -6.2622 -55.8406

C 5.6572 -6.5512 -56.9656

H 5.9292 -6.7212 -58.0086

H 5.8812 -7.4662 -56.4106

C 6.5082 -5.4132 -56.4086

H 6.3272 -4.5072 -56.9916

H 6.2092 -5.2022 -55.3786

C 7.9952 -5.7572 -56.4506

H 8.2732 -6.0142 -57.4746

H 8.1792 -6.6352 -55.8266

C 8.8742 -4.6072 -55.9666

H 8.7182 -3.7372 -56.6096

H 8.5722 -4.3212 -54.9556

C 10.3542 -4.9822 -55.9686

H 10.5082 -5.8462 -55.3186

H 10.6502 -5.2702 -56.9796

C 11.2412 -3.8342 -55.4946

H 10.9282 -3.5262 -54.4936

H 11.1062 -2.9752 -56.1556

C 12.7122 -4.2302 -55.4676

H 12.8692 -5.0702 -54.7946

H 13.3292 -3.4002 -55.1306

H 13.0452 -4.5222 -56.4616

C 4.9792 -5.2512 -60.8876

H 4.6002 -5.0102 -59.8836

H 4.7942 -4.3932 -61.5406

C 6.4712 -5.5542 -60.8376

H 6.7872 -5.8432 -61.8416

H 6.6292 -6.4082 -60.1776

C 7.3062 -4.3732 -60.3516

H 7.1372 -3.5082 -60.9976

H 6.9982 -4.0972 -59.3416

C 8.7912 -4.7282 -60.3536

H 9.0872 -5.0172 -61.3656

H 8.9512 -5.5942 -59.7066

C 9.6862 -3.5872 -59.8786

H 9.5452 -2.7152 -60.5226

H 9.4022 -3.2992 -58.8646

C 11.1562 -3.9982 -59.8946

H 11.4362 -4.2882 -60.9106

H 11.2892 -4.8762 -59.2566

C 12.0892 -2.8902 -59.4136

H 11.9812 -2.0152 -60.0586

H 11.8072 -2.5922 -58.4026

C 13.5462 -3.3472 -59.4166

H 13.8242 -3.6472 -60.4296

H 13.6482 -4.2262 -58.7746

C 14.5032 -2.2612 -58.9346

H 14.4152 -1.3872 -59.5846

H 14.2212 -1.9522 -57.9246

C 15.9512 -2.7452 -58.9296

H 16.2142 -3.1032 -59.9286

H 16.0472 -3.5902 -58.2436

C 16.9292 -1.6492 -58.5176

H 16.8152 -0.7922 -59.1856

H 16.6902 -1.3142 -57.5056

C 18.3722 -2.1372 -58.5636

H 18.5112 -2.9832 -57.8936

H 19.0562 -1.3462 -58.2626

H 18.6352 -2.4522 -59.5706

C 4.4182 -4.3752 -64.9976

H 3.9232 -3.4002 -64.8776

H 3.8982 -4.9392 -65.7846

C 5.8712 -4.1782 -65.4106

H 5.8762 -3.6482 -66.3646

H 6.3242 -5.1592 -65.5656

C 6.6912 -3.4022 -64.3846

H 6.7332 -3.9742 -63.4566

H 6.2022 -2.4492 -64.1676

C 8.1032 -3.1482 -64.9026

H 8.5392 -4.0962 -65.2266

H 8.0522 -2.4962 -65.7776

C 9.0202 -2.5172 -63.8586

H 8.5902 -1.5752 -63.5106

H 9.0962 -3.1842 -62.9966

C 10.4142 -2.2662 -64.4286

H 10.7962 -3.1982 -64.8506

H 10.3452 -1.5412 -65.2426

C 11.4002 -1.7582 -63.3806

H 11.4682 -2.4872 -62.5696

H 11.0322 -0.8232 -62.9526

C 12.7892 -1.5372 -63.9736

H 13.1212 -2.4572 -64.4606

H 12.7392 -0.7622 -64.7426

C 13.8132 -1.1372 -62.9146

H 13.5052 -0.1992 -62.4456

H 13.8362 -1.9012 -62.1336

C 15.2142 -0.9762 -63.4986

H 15.2102 -0.1742 -64.2406

H 15.4982 -1.8982 -64.0126

C 16.2502 -0.6682 -62.4206

H 15.9792 0.2608 -61.9136

H 16.2382 -1.4672 -61.6776

C 17.6542 -0.5402 -62.9976

H 17.9492 -1.4642 -63.4916

H 18.3742 -0.3282 -62.2096

H 17.6972 0.2648 -63.7276

O 4.2982 -6.4062 -61.3706

O 2.0012 -7.2952 -60.3776

O 4.3752 -5.0872 -63.7766

O -1.5308 -5.0732 -63.3186

C -3.4258 -4.8372 -58.9626

C -2.1878 -5.0222 -58.3386

C -4.5808 -4.7352 -58.1776

C -2.1068 -5.1222 -56.9676

H -1.2978 -5.0832 -58.9476

C -4.4978 -4.8392 -56.8096

H -5.5348 -4.5852 -58.6606

C -3.2628 -5.0382 -56.1896

H -1.1528 -5.2672 -56.4836

H -5.3828 -4.7772 -56.1936

C -4.2738 -4.7562 -69.7176

C -2.9948 -4.9272 -70.2626

C -5.3708 -4.6412 -70.5756

C -2.8268 -5.0202 -71.6256

H -2.1468 -4.9962 -69.5976

C -5.2008 -4.7482 -71.9386

H -6.3528 -4.4722 -70.1606

C -3.9298 -4.9532 -72.4756

H -1.8478 -5.1582 -72.0566

H -6.0478 -4.6742 -72.6026

C -7.7428 -5.1662 -64.5446

H -7.4858 -6.1112 -64.0406

H -7.9488 -4.4072 -63.7766

C -0.5048 -5.4642 -64.2106

H -0.8138 -6.3802 -64.7366

H -0.3268 -4.6752 -64.9546

C -1.2008 -7.3992 2.1364

C -1.0528 -7.8242 -3.5076

C -1.0918 -7.8192 -4.7166

C -1.1558 -7.7862 -6.1206

C 0.0252 -7.6612 -6.8816

C -2.3978 -7.8252 -6.7556

H -3.2738 -7.8932 -6.1306

O -2.2458 -7.5662 2.7514

O -0.0758 -7.0522 2.7174

H -0.1778 -6.9942 3.7164

C -1.9768 -7.5432 -17.1266

C -1.3988 -7.8542 -11.5086

C -1.3788 -7.8012 -10.3006

C -1.3168 -7.7582 -8.8976

C -2.5038 -7.7532 -8.1356

C -0.0748 -7.6942 -8.2616

H 0.8052 -7.6672 -8.8856

O -1.0378 -7.3102 -17.8796

O -3.2188 -7.6532 -17.5436

H -3.3078 -7.5282 -18.5356

C -5.9428 -6.7802 -8.9146

C -5.9398 -6.8492 -10.2956

H -5.0758 -7.2572 -10.7916

C -7.0528 -6.4072 -11.0096

C -8.1758 -5.9252 -10.3246

C -8.1478 -5.8082 -8.9276

C -7.0218 -6.2282 -8.2346

H -6.9758 -6.1512 -7.1606

C -6.1448 -6.9662 -13.1536

H -5.1908 -6.4572 -12.9536

H -6.0268 -8.0332 -12.9166

C -6.5038 -6.7922 -14.6286

H -6.6928 -5.7352 -14.8166

H -5.6278 -7.0932 -15.2066

C -7.7198 -7.6092 -15.0766

H -7.6958 -8.5872 -14.5896

H -7.6538 -7.7782 -16.1546

C -9.0448 -6.9162 -14.7676

H -9.0638 -6.6262 -13.7176

H -9.1118 -6.0022 -15.3616

C -10.2478 -7.8052 -15.0766

H -10.2048 -8.7012 -14.4506

H -10.2008 -8.1332 -16.1186

C -11.5698 -7.0822 -14.8316

H -11.5798 -6.6882 -13.8146

H -11.6458 -6.2292 -15.5096

C -12.7788 -7.9952 -15.0306

H -12.7408 -8.8092 -14.3026

H -12.7308 -8.4462 -16.0256

C -14.0988 -7.2422 -14.8826

H -14.1298 -6.7512 -13.9086

H -14.1528 -6.4602 -15.6426

C -15.3108 -8.1612 -15.0256

H -15.3228 -8.8792 -14.2006

H -15.2218 -8.7352 -15.9516

C -16.6218 -7.3792 -15.0426

H -16.6088 -6.6822 -15.8826

H -16.7048 -6.7912 -14.1266

C -17.8408 -8.2892 -15.1706

H -17.7268 -8.9232 -16.0546

H -17.8948 -8.9512 -14.3026

C -19.1338 -7.4922 -15.2856

H -19.1068 -6.8542 -16.1666

H -19.9898 -8.1572 -15.3716

H -19.2728 -6.8622 -14.4106

C -10.3528 -6.5592 -10.8966

H -10.2698 -7.2842 -11.7216

H -10.2458 -7.1102 -9.9556

C -11.7088 -5.8682 -10.9366

H -11.7808 -5.1932 -10.0836

H -11.7628 -5.2632 -11.8436

C -12.8578 -6.8722 -10.9136

H -12.7828 -7.4912 -10.0156

H -12.7768 -7.5372 -11.7756

C -14.2148 -6.1742 -10.9376

H -14.3168 -5.5462 -10.0516

H -14.2638 -5.5182 -11.8096

C -15.3748 -7.1652 -10.9946

H -15.3338 -7.8232 -10.1236

H -15.2718 -7.7922 -11.8836

C -16.7258 -6.4562 -11.0336

H -16.8428 -5.8492 -10.1336

H -16.7478 -5.7772 -11.8896

C -17.8898 -7.4362 -11.1416

H -17.8738 -8.1122 -10.2826

H -17.7658 -8.0452 -12.0396

C -19.2378 -6.7232 -11.1976

H -19.3668 -6.1232 -10.2956

H -19.2498 -6.0402 -12.0506

C -20.4008 -7.7022 -11.3246

H -20.3958 -8.3802 -10.4676

H -20.2688 -8.3082 -12.2246

C -21.7478 -6.9862 -11.3916

H -21.8668 -6.3622 -10.5026

H -21.7618 -6.3272 -12.2636

C -22.9168 -7.9622 -11.4806

H -22.9078 -8.6162 -10.6056

H -22.7938 -8.5922 -12.3656

C -24.2578 -7.2412 -11.5556

H -24.2948 -6.5922 -12.4286

H -25.0738 -7.9562 -11.6266

H -24.4138 -6.6312 -10.6686

C -9.3308 -5.1852 -6.9486

H -9.1038 -6.1462 -6.4656

H -8.5968 -4.4382 -6.6146

C -10.7458 -4.7452 -6.5916

H -10.7748 -4.4672 -5.5376

H -10.9848 -3.8622 -7.1836

C -11.7688 -5.8452 -6.8736

H -11.5968 -6.2352 -7.8776

H -11.6248 -6.6662 -6.1666

C -13.1988 -5.3262 -6.7726

H -13.3458 -4.5472 -7.5246

H -13.3508 -4.8672 -5.7946

C -14.2418 -6.4212 -6.9816

H -14.1528 -7.1672 -6.1876

H -14.0528 -6.9282 -7.9306

C -15.6558 -5.8472 -6.9856

H -15.7488 -5.1412 -7.8146

H -15.8188 -5.2902 -6.0616

C -16.7338 -6.9182 -7.1226

H -16.5708 -7.4862 -8.0406

H -16.6608 -7.6172 -6.2866

C -18.1308 -6.3032 -7.1486

H -18.2158 -5.6482 -8.0196

H -18.2678 -5.6852 -6.2586

C -19.2348 -7.3542 -7.2046

H -19.1678 -7.9972 -6.3226

H -19.0908 -7.9842 -8.0846

C -20.6218 -6.7182 -7.2606

H -20.7678 -6.0922 -6.3776

H -20.6828 -6.0722 -8.1396

C -21.7358 -7.7592 -7.3316

H -21.6868 -8.4022 -6.4496

H -21.5838 -8.3902 -8.2096

C -23.1128 -7.1112 -7.4096

H -23.1878 -6.4822 -8.2936

H -23.8918 -7.8682 -7.4646

H -23.2958 -6.4932 -6.5336

O -9.3148 -5.5942 -11.0246

O -7.1728 -6.4112 -12.3636

O -9.2728 -5.3162 -8.3546

O -3.6758 -7.6572 -8.8196

C 3.3732 -6.7102 -5.8426

C 3.0352 -5.9532 -4.7316

H 2.0272 -5.5822 -4.6456

C 3.9832 -5.7132 -3.7436

C 5.2892 -6.2082 -3.8916

C 5.6342 -6.9262 -5.0446

C 4.6642 -7.1852 -6.0066

H 4.9002 -7.7722 -6.8806

C 2.4902 -4.4842 -2.3366

H 1.6992 -5.2052 -2.5776

H 2.3482 -3.5902 -2.9616

C 2.4002 -4.1202 -0.8556

H 2.4552 -5.0352 -0.2636

H 1.4192 -3.6702 -0.6966

C 3.4922 -3.1532 -0.3956

H 3.7292 -2.4692 -1.2096

H 3.1152 -2.5622 0.4444

C 4.7742 -3.8632 0.0344

H 5.0932 -4.5342 -0.7616

H 4.5742 -4.4662 0.9244

C 5.8892 -2.8642 0.3284

H 6.0812 -2.2782 -0.5726

H 5.5682 -2.1742 1.1144

C 7.1802 -3.5562 0.7564

H 7.4282 -4.3202 0.0194

H 7.0352 -4.0562 1.7184

C 8.3332 -2.5632 0.8534

H 8.4162 -2.0482 -0.1056

H 8.1072 -1.8052 1.6084

C 9.6692 -3.2262 1.1804

H 9.8382 -4.0532 0.4884

H 9.6372 -3.6412 2.1904

C 10.8242 -2.2352 1.0674

H 10.8632 -1.8582 0.0424

H 10.6372 -1.3792 1.7204

C 12.1742 -2.8542 1.4204

H 12.1592 -3.1852 2.4614

H 12.3422 -3.7332 0.7954

C 13.3192 -1.8662 1.2184

H 13.1382 -0.9742 1.8234

H 13.3392 -1.5542 0.1714

C 14.6692 -2.4632 1.5914

H 14.6782 -2.7582 2.6384

H 15.4672 -1.7412 1.4324

H 14.8772 -3.3422 0.9854

C 7.2412 -5.1052 -3.1296

H 6.9902 -4.1402 -2.6696

H 7.3452 -4.9482 -4.2056

C 8.5532 -5.6362 -2.5616

H 8.8142 -6.5612 -3.0806

H 8.4162 -5.8772 -1.5066

C 9.6532 -4.5932 -2.7316

H 9.6792 -4.2772 -3.7776

H 9.4002 -3.7172 -2.1336

C 11.0392 -5.0942 -2.3326

H 11.2962 -5.9752 -2.9266

H 11.0362 -5.3982 -1.2836

C 12.0902 -4.0072 -2.5486

H 12.0352 -3.6602 -3.5846

H 11.8562 -3.1552 -1.9066

C 13.5142 -4.4752 -2.2596

H 13.7602 -5.3192 -2.9096

H 13.5822 -4.8262 -1.2276

C 14.5222 -3.3492 -2.4806

H 14.4182 -2.9702 -3.5016

H 14.2912 -2.5252 -1.8026

C 15.9652 -3.7902 -2.2586

H 16.2192 -4.5792 -2.9706

H 16.0672 -4.2082 -1.2536

C 16.9392 -2.6252 -2.4196

H 16.8232 -2.1952 -3.4186

H 16.6872 -1.8462 -1.6966

C 18.3932 -3.0452 -2.2236

H 18.6522 -3.8082 -2.9616

H 18.5092 -3.4912 -1.2326

C 19.3512 -1.8652 -2.3586

H 19.2402 -1.4232 -3.3526

H 19.0852 -1.1012 -1.6246

C 20.8032 -2.2772 -2.1576

H 20.9432 -2.7102 -1.1696

H 21.4632 -1.4172 -2.2506

H 21.0972 -3.0162 -2.8996

C 7.3002 -8.1862 -6.1986

H 7.1482 -7.6732 -7.1596

H 6.6892 -9.0992 -6.1866

C 8.7742 -8.5372 -6.0316

H 9.0442 -9.2712 -6.7936

H 8.9172 -8.9912 -5.0506

C 9.6562 -7.2992 -6.1606

H 9.3842 -6.5942 -5.3756

H 9.4602 -6.8222 -7.1236

C 11.1432 -7.6262 -6.0586

H 11.3402 -8.1512 -5.1216

H 11.4232 -8.2952 -6.8766

C 11.9942 -6.3612 -6.1226

H 11.7292 -5.7982 -7.0206

H 11.7652 -5.7302 -5.2626

C 13.4892 -6.6622 -6.1436

H 13.7572 -7.2392 -5.2546

H 13.7232 -7.2772 -7.0166

C 14.3212 -5.3842 -6.1876

H 14.0942 -4.7772 -5.3096

H 14.0392 -4.8012 -7.0676

C 15.8202 -5.6672 -6.2266

H 16.1042 -6.2282 -5.3326

H 16.0512 -6.2942 -7.0926

C 16.6392 -4.3822 -6.2986

H 16.3902 -3.8462 -7.2186

H 16.3682 -3.7372 -5.4606

C 18.1412 -4.6482 -6.2606

H 18.4222 -5.2792 -7.1066

H 18.3862 -5.1952 -5.3466

C 18.9502 -3.3542 -6.3016

H 18.7262 -2.8182 -7.2266

H 18.6452 -2.7172 -5.4686

C 20.4482 -3.6112 -6.2156

H 20.6922 -4.1432 -5.2986

H 21.0002 -2.6742 -6.2246

H 20.7832 -4.2162 -7.0566

O 6.1832 -6.0242 -2.8686

O 3.7602 -5.0422 -2.5916

O 6.9282 -7.3412 -5.1296

O 1.1792 -7.5272 -6.1856

C -1.0578 -7.7812 -2.1016

C 0.1272 -7.5832 -1.3846

C -2.2688 -7.9102 -1.4106

C 0.0942 -7.4692 -0.0116

H 1.0622 -7.5142 -1.9206

C -2.2958 -7.8062 -0.0396

H -3.1788 -8.0812 -1.9666

C -1.1188 -7.5642 0.6704

H 1.0022 -7.3052 0.5464

H -3.2208 -7.8942 0.5084

C -1.5028 -7.8542 -12.9106

C -0.4188 -7.4972 -13.7206

C -2.7228 -8.1922 -13.5076

C -0.5698 -7.4222 -15.0866

H 0.5322 -7.2712 -13.2626

C -2.8708 -8.1182 -14.8726

H -3.5418 -8.5092 -12.8806

C -1.8048 -7.7052 -15.6726

H 0.2552 -7.1292 -15.7176

H -3.8068 -8.3822 -15.3376

C -4.8198 -7.3162 -8.0636

H -5.1868 -8.2062 -7.5266

H -4.5588 -6.5512 -7.3226

C 2.3162 -7.0302 -6.8656

H 2.6972 -7.7742 -7.5776

H 2.0362 -6.1212 -7.4196

3504

acid_LT 0.000000

C -2.8039 7.6670 -30.8238

C -2.9509 7.7440 -36.4718

C -2.9819 7.7600 -37.6798

C -3.0389 7.7580 -39.0828

C -1.8469 7.6980 -39.8368

C -4.2809 7.8290 -39.7208

H -5.1559 7.9200 -39.0978

O -3.8479 7.6520 -30.1758

O -1.6199 7.6320 -30.2638

H -1.6739 7.6180 -29.2528

C -3.0799 6.8750 -50.0678

C -3.2659 7.2270 -44.4298

C -3.2549 7.4240 -43.2378

C -3.1979 7.6000 -41.8458

C -4.3819 7.7800 -41.1008

C -1.9539 7.6020 -41.2148

H -1.0819 7.5100 -41.8428

O -2.0189 6.9430 -50.6718

O -4.2419 6.7810 -50.6778

H -4.1239 6.7890 -51.6778

C -7.7909 8.5860 -42.1538

C -7.5299 8.9160 -43.4738

H -6.5039 9.0360 -43.7798

C -8.5799 9.0450 -44.3788

C -9.9019 8.8680 -43.9468

C -10.1599 8.6130 -42.5928

C -9.0979 8.4500 -41.7128

H -9.2769 8.2050 -40.6788

C -7.1589 9.2990 -46.2778

H -6.5819 10.1820 -45.9628

H -6.6209 8.4040 -45.9418

C -7.2759 9.2800 -47.8028

H -7.7209 10.2160 -48.1408

H -6.2589 9.2210 -48.1978

C -8.1079 8.1090 -48.3368

H -7.9689 7.2400 -47.6908

H -7.7489 7.8400 -49.3338

C -9.5949 8.4510 -48.4158

H -9.9269 8.8070 -47.4418

H -9.7349 9.2650 -49.1318

C -10.4519 7.2600 -48.8388

H -10.3449 6.4600 -48.1018

H -10.0999 6.8680 -49.7978

C -11.9239 7.6530 -48.9488

H -12.2169 8.1790 -48.0398

H -12.0479 8.3520 -49.7798

C -12.8519 6.4570 -49.1498

H -12.7089 5.7490 -48.3288

H -12.5949 5.9380 -50.0758

C -14.3149 6.8890 -49.1928

H -14.5549 7.4300 -48.2758

H -14.4589 7.5820 -50.0258

C -15.2769 5.7140 -49.3458

H -15.1249 5.0120 -48.5218

H -15.0609 5.1810 -50.2738

C -16.7309 6.1780 -49.3528

H -16.8799 6.8770 -50.1808

H -16.9399 6.7180 -48.4268

C -17.7199 5.0240 -49.4928

H -17.5229 4.4910 -50.4248

H -17.5699 4.3190 -48.6718

C -19.1619 5.5160 -49.4838

H -19.3319 6.2090 -50.3058

H -19.8539 4.6840 -49.5908

H -19.3829 6.0310 -48.5518

C -11.7179 7.7920 -45.0158

H -11.4429 7.3090 -45.9628

H -11.5179 7.0860 -44.2048

C -13.1969 8.1590 -45.0248

H -13.4319 8.6640 -44.0878

H -13.3789 8.8630 -45.8388

C -14.0789 6.9250 -45.1968

H -13.8809 6.2220 -44.3838

H -13.8209 6.4220 -46.1318

C -15.5629 7.2810 -45.2058

H -15.8179 7.7920 -44.2748

H -15.7599 7.9770 -46.0248

C -16.4529 6.0520 -45.3698

H -16.2459 5.3440 -44.5628

H -16.2109 5.5520 -46.3098

C -17.9359 6.4130 -45.3538

H -18.1729 6.9220 -44.4168

H -18.1479 7.1110 -46.1668

C -18.8309 5.1860 -45.5018

H -18.6089 4.4760 -44.7008

H -18.6119 4.6880 -46.4488

C -20.3119 5.5520 -45.4538

H -20.5309 6.0410 -44.5018

H -20.5339 6.2700 -46.2478

C -21.2149 4.3320 -45.6138

H -20.9759 3.6010 -44.8368

H -21.0169 3.8600 -46.5798

C -22.6939 4.7000 -45.5218

H -22.8849 5.1830 -44.5618

H -22.9359 5.4210 -46.3068

C -23.6039 3.4830 -45.6618

H -23.3509 2.7510 -44.8908

H -23.4299 3.0110 -46.6318

C -25.0749 3.8610 -45.5388

H -25.3499 4.5770 -46.3098

H -25.7079 2.9830 -45.6438

H -25.2719 4.3110 -44.5678

C -11.7849 8.4520 -40.8598

H -11.4869 7.4800 -40.4408

H -11.2499 9.2490 -40.3248

C -13.2859 8.6590 -40.7078

H -13.4929 8.8920 -39.6628

H -13.5689 9.5260 -41.3068

C -14.1169 7.4520 -41.1338

H -13.8909 7.2030 -42.1698

H -13.8539 6.5900 -40.5158

C -15.6059 7.7500 -40.9838

H -15.8729 8.5790 -41.6438

H -15.8029 8.0780 -39.9598

C -16.4969 6.5520 -41.2998

H -16.2609 5.7320 -40.6178

H -16.2999 6.2060 -42.3148

C -17.9729 6.9160 -41.1638

H -18.2139 7.7090 -41.8768

H -18.1539 7.3180 -40.1638

C -18.9019 5.7290 -41.4018

H -18.7219 5.3200 -42.3978

H -18.6779 4.9440 -40.6768

C -20.3679 6.1320 -41.2758

H -20.5979 6.8910 -42.0278

H -20.5349 6.5890 -40.2968

C -21.3189 4.9500 -41.4478

H -21.0829 4.1850 -40.7048

H -21.1709 4.5090 -42.4358

C -22.7779 5.3700 -41.2928

H -22.9279 5.8010 -40.2998

H -23.0109 6.1490 -42.0238

C -23.7419 4.2030 -41.4878

H -23.5319 3.4320 -40.7428

H -23.5799 3.7620 -42.4728

C -25.1949 4.6470 -41.3638

H -25.4219 5.4160 -42.0998

H -25.8689 3.8080 -41.5228

H -25.3849 5.0580 -40.3748

O -10.9149 8.9600 -44.8708

O -8.4449 9.3300 -45.7008

O -11.4699 8.5110 -42.2348

O -5.5289 7.8860 -41.8148

C 1.7481 7.8430 -39.0658

C 1.7521 7.3340 -37.7788

H 0.8111 7.0610 -37.3298

C 2.9631 7.1680 -37.1178

C 4.1771 7.4930 -37.7458

C 4.1601 8.0600 -39.0298

C 2.9361 8.2450 -39.6628

H 2.8951 8.6700 -40.6528

C 2.0171 6.5520 -34.9988

H 1.1641 6.0540 -35.4758

H 1.7041 7.5680 -34.7078

C 2.4181 5.7520 -33.7598

H 2.6081 4.7190 -34.0558

H 1.5561 5.7640 -33.0908

C 3.6521 6.3000 -33.0368

H 3.6311 7.3910 -33.0688

H 3.6141 6.0020 -31.9848

C 4.9571 5.7980 -33.6548

H 4.9501 6.0080 -34.7238

H 5.0201 4.7140 -33.5268

C 6.1851 6.4520 -33.0278

H 6.1121 7.5350 -33.1588

H 6.2041 6.2610 -31.9498

C 7.4841 5.9580 -33.6638

H 7.3911 6.0200 -34.7488

H 7.6421 4.9080 -33.4038

C 8.6811 6.7860 -33.2068

H 8.5201 7.8270 -33.5028

H 8.7401 6.7550 -32.1168

C 10.0171 6.3160 -33.7758

H 9.9761 6.3300 -34.8668

H 10.2061 5.2870 -33.4618

C 11.1541 7.2150 -33.2938

H 10.9601 8.2400 -33.6198

H 11.1681 7.2170 -32.2008

C 12.5231 6.7780 -33.8038

H 12.7151 5.7480 -33.4948

H 12.5291 6.8020 -34.8958

C 13.6351 7.6800 -33.2718

H 13.6411 7.6360 -32.1798

H 13.4261 8.7130 -33.5588

C 15.0051 7.2750 -33.7978

H 15.2431 6.2550 -33.5028

H 15.7771 7.9340 -33.4058

H 15.0281 7.3300 -34.8838

C 6.3511 8.0990 -36.9128

H 6.4501 8.2730 -35.8338

H 6.1011 9.0460 -37.3918

C 7.6781 7.5690 -37.4508

H 7.6211 7.4930 -38.5358

H 7.8501 6.5670 -37.0518

C 8.8041 8.5090 -37.0318

H 8.5701 9.5120 -37.3958

H 8.8351 8.5530 -35.9408

C 10.1781 8.0880 -37.5448

H 10.1831 8.0950 -38.6358

H 10.3961 7.0660 -37.2218

C 11.2581 9.0290 -37.0158

H 11.0101 10.0530 -37.3048

H 11.2571 8.9860 -35.9238

C 12.6591 8.7000 -37.5228

H 12.6851 8.7810 -38.6108

H 12.9111 7.6680 -37.2638

C 13.6921 9.6440 -36.9118

H 13.4191 10.6760 -37.1478

H 13.6681 9.5380 -35.8248

C 15.1121 9.3810 -37.4048

H 15.1561 9.5280 -38.4858

H 15.3841 8.3420 -37.2038

C 16.1151 10.3090 -36.7228

H 15.8211 11.3470 -36.9018

H 16.0811 10.1370 -35.6438

C 17.5431 10.1010 -37.2198

H 17.5741 10.2550 -38.3008

H 17.8501 9.0710 -37.0248

C 18.5231 11.0600 -36.5468

H 18.1861 12.0870 -36.7058

H 18.5251 10.8760 -35.4698

C 19.9381 10.9080 -37.0888

H 20.3041 9.8970 -36.9248

H 20.6131 11.6030 -36.5938

H 19.9601 11.1110 -38.1568

C 5.4031 8.8190 -40.9258

H 5.0361 9.8540 -40.9678

H 4.7661 8.1900 -41.5618

C 6.8381 8.7350 -41.4398

H 6.8181 8.9140 -42.5168

H 7.1991 7.7180 -41.2748

C 7.7871 9.7280 -40.7738

H 7.8011 9.5500 -39.6998

H 7.4221 10.7430 -40.9288

C 9.1981 9.6020 -41.3458

H 9.5501 8.5750 -41.2178

H 9.1701 9.8040 -42.4188

C 10.1861 10.5550 -40.6788

H 9.8281 11.5810 -40.7848

H 10.2341 10.3290 -39.6118

C 11.5871 10.4440 -41.2768

H 11.9361 9.4120 -41.1858

H 11.5491 10.6820 -42.3418

C 12.5831 11.3710 -40.5828

H 12.5861 11.1540 -39.5128

H 12.2611 12.4080 -40.7088

C 14.0011 11.2140 -41.1248

H 14.3191 10.1750 -41.0048

H 14.0131 11.4350 -42.1948

C 14.9881 12.1290 -40.4038

H 14.6891 13.1700 -40.5508

H 14.9491 11.9230 -39.3308

C 16.4221 11.9440 -40.8928

H 16.4681 12.1330 -41.9688

H 16.7301 10.9070 -40.7328

C 17.3941 12.8780 -40.1758

H 17.0511 13.9080 -40.2918

H 17.3941 12.6460 -39.1088

C 18.8121 12.7590 -40.7188

H 19.1761 11.7380 -40.6168

H 19.4861 13.4200 -40.1798

H 18.8411 13.0240 -41.7738

O 5.3081 7.1440 -37.0658

O 3.1151 6.6310 -35.8738

O 5.3641 8.3530 -39.5918

O -0.6789 7.7360 -39.1478

C -2.9089 7.7400 -35.0678

C -1.6809 7.6290 -34.4038

C -4.0859 7.8530 -34.3238

C -1.6359 7.6180 -33.0308

H -0.7779 7.5530 -34.9888

C -4.0399 7.8340 -32.9498

H -5.0299 7.9610 -34.8378

C -2.8159 7.7090 -32.2928

H -0.6939 7.5330 -32.5108

H -4.9449 7.9200 -32.3688

C -3.2379 7.0690 -45.8258

C -2.0169 7.1710 -46.5008

C -4.4089 6.8310 -46.5508

C -1.9839 7.0940 -47.8728

H -1.1139 7.3210 -45.9298

C -4.3729 6.7610 -47.9248

H -5.3359 6.6950 -46.0148

C -3.1599 6.9090 -48.5978

H -1.0589 7.1900 -48.4178

H -5.2759 6.5920 -48.4908

C -6.6749 8.3850 -41.1608

H -7.0099 7.6820 -40.3848

H -6.4329 9.3450 -40.6768

C 0.5041 7.8590 -39.9138

H 0.5791 7.0080 -40.6088

H 0.4611 8.7860 -40.5018

C -2.3049 7.6290 -8.1338

C -3.0749 8.0320 -13.7188

C -3.1239 8.0940 -14.9258

C -3.1749 8.1520 -16.3278

C -1.9669 8.1790 -17.0598

C -4.4089 8.1860 -16.9838

H -5.2939 8.1840 -16.3678

O -3.2429 7.6090 -7.3388

O -1.0529 7.5160 -7.7598

H -0.9489 7.4230 -6.7638

C -2.9439 7.6930 -27.3368

C -3.1609 7.9990 -21.6968

C -3.2839 8.0940 -20.4978

C -3.2849 8.1620 -19.0958

C -4.4909 8.2130 -18.3668

C -2.0519 8.1680 -18.4418

H -1.1689 8.1490 -19.0598

O -1.8929 7.6450 -27.9588

O -4.1219 7.6840 -27.9188

H -4.0339 7.6520 -28.9238

C -8.0339 8.6100 -19.3098

C -7.9499 8.4870 -20.6848

H -6.9869 8.2960 -21.1268

C -9.1049 8.5910 -21.4618

C -10.3419 8.8200 -20.8478

C -10.4139 8.9650 -19.4558

C -9.2569 8.8570 -18.6988

H -9.2909 8.9590 -17.6268

C -8.0149 8.0610 -23.5178

H -7.2089 8.7980 -23.3858

H -7.6709 7.0900 -23.1328

C -8.3429 7.9460 -25.0068

H -8.6209 8.9300 -25.3838

H -7.4239 7.6340 -25.5078

C -9.4649 6.9520 -25.3208

H -9.4239 6.1220 -24.6118

H -9.3039 6.5380 -26.3198

C -10.8499 7.5950 -25.2728

H -10.9949 8.0660 -24.3008

H -10.9049 8.3800 -26.0318

C -11.9569 6.5740 -25.5248

H -11.9319 5.8150 -24.7378

H -11.7759 6.0640 -26.4748

C -13.3429 7.2160 -25.5548

H -13.4919 7.7890 -24.6378

H -13.3999 7.9170 -26.3918

C -14.4489 6.1730 -25.6878

H -14.3799 5.4710 -24.8528

H -14.2989 5.6010 -26.6068

C -15.8439 6.7940 -25.7028

H -15.9849 7.3910 -24.8008

H -15.9319 7.4670 -26.5598

C -16.9349 5.7290 -25.7828

H -16.8359 5.0490 -24.9318

H -16.7939 5.1370 -26.6908

C -18.3389 6.3270 -25.7858

H -18.4439 6.9960 -26.6438

H -18.4799 6.9250 -24.8828

C -19.4179 5.2500 -25.8548

H -19.2759 4.6570 -26.7608

H -19.3109 4.5760 -25.0008

C -20.8199 5.8460 -25.8568

H -20.9549 6.4980 -26.7168

H -21.5719 5.0610 -25.9028

H -20.9879 6.4310 -24.9548

C -12.3849 7.8270 -21.5018

H -12.1919 7.1080 -22.3108

H -12.2349 7.3120 -20.5468

C -13.8149 8.3480 -21.5908

H -13.9939 9.0160 -20.7478

H -13.9119 8.9340 -22.5058

C -14.8379 7.2150 -21.5948

H -14.7019 6.5990 -20.7018

H -14.6659 6.5750 -22.4628

C -16.2689 7.7450 -21.6318

H -16.4519 8.3580 -20.7478

H -16.3889 8.3880 -22.5068

C -17.3029 6.6230 -21.6948

H -17.1749 5.9620 -20.8338

H -17.1339 6.0270 -22.5938

C -18.7319 7.1630 -21.7088

H -18.9099 7.7410 -20.7998

H -18.8519 7.8400 -22.5578

C -19.7689 6.0480 -21.8128

H -19.6629 5.3720 -20.9608

H -19.5839 5.4670 -22.7178

C -21.1939 6.5940 -21.8478

H -21.3919 7.1500 -20.9298

H -21.2909 7.2950 -22.6818

C -22.2319 5.4850 -22.0008

H -22.1499 4.7970 -21.1568

H -22.0229 4.9170 -22.9108

C -23.6539 6.0360 -22.0668

H -23.8609 6.6100 -21.1608

H -23.7369 6.7190 -22.9168

C -24.6979 4.9320 -22.2078

H -24.6269 4.2570 -21.3528

H -24.4869 4.3490 -23.1078

C -26.1109 5.4960 -22.2898

H -26.2089 6.1580 -23.1468

H -26.8389 4.6940 -22.3908

H -26.3489 6.0620 -21.3918

C -11.8029 9.3240 -17.5498

H -11.4559 8.4150 -17.0368

H -11.2119 10.1790 -17.1918

C -13.2809 9.5580 -17.2668

H -13.3949 9.8530 -16.2238

H -13.6169 10.3890 -17.8888

C -14.1349 8.3280 -17.5608

H -13.9379 7.9950 -18.5808

H -13.8569 7.5160 -16.8858

C -15.6209 8.6370 -17.4048

H -15.8949 9.4250 -18.1098

H -15.8069 9.0240 -16.3998

C -16.5079 7.4200 -17.6488

H -16.3049 6.6600 -16.8918

H -16.2709 6.9870 -18.6228

C -17.9859 7.7960 -17.6108

H -18.1949 8.5010 -18.4188

H -18.2059 8.3070 -16.6698

C -18.9069 6.5880 -17.7528

H -18.6659 6.0540 -18.6738

H -18.7379 5.9030 -16.9188

C -20.3749 7.0040 -17.7788

H -20.5429 7.6580 -18.6388

H -20.6029 7.5820 -16.8798

C -21.3239 5.8130 -17.8638

H -21.1779 5.1690 -16.9938

H -21.0869 5.2260 -18.7548

C -22.7819 6.2590 -17.9268

H -23.0239 6.8330 -17.0288

H -22.9169 6.9180 -18.7878

C -23.7479 5.0840 -18.0488

H -23.6229 4.4210 -17.1898

H -23.5059 4.5110 -18.9468

C -25.1959 5.5510 -18.1238

H -25.3379 6.2100 -18.9778

H -25.8699 4.7050 -18.2288

H -25.4679 6.0980 -17.2228

O -11.4719 8.9130 -21.6298

O -9.1619 8.4760 -22.8138

O -11.6539 9.1820 -18.9458

O -5.6369 8.2760 -19.0928

C 1.6211 8.4010 -16.2478

C 1.6601 7.8680 -14.9708

H 0.7431 7.5090 -14.5328

C 2.8771 7.7950 -14.3008

C 4.0631 8.2370 -14.9118

C 4.0091 8.8100 -16.1928

C 2.7761 8.9060 -16.8298

H 2.7071 9.3450 -17.8118

C 1.9661 7.0440 -12.2178

H 1.2081 6.4230 -12.7118

H 1.5051 8.0090 -11.9558

C 2.4311 6.3260 -10.9518

H 2.7951 5.3340 -11.2238

H 1.5461 6.2040 -10.3238

C 3.5251 7.0680 -10.1798

H 3.3401 8.1430 -10.2378

H 3.4791 6.7830 -9.1258

C 4.9221 6.7610 -10.7158

H 4.9581 6.9880 -11.7818

H 5.1221 5.6930 -10.5968

C 6.0021 7.5580 -9.9898

H 5.7991 8.6240 -10.1158

H 5.9601 7.3470 -8.9168

C 7.3991 7.2470 -10.5258

H 7.3871 7.3300 -11.6138

H 7.6661 6.2170 -10.2758

C 8.4461 8.2010 -9.9568

H 8.1891 9.2220 -10.2518

H 8.4141 8.1570 -8.8648

C 9.8651 7.8920 -10.4308

H 9.8881 7.8770 -11.5218

H 10.1591 6.8990 -10.0798

C 10.8581 8.9300 -9.9138

H 10.5871 9.9120 -10.3098

H 10.7851 8.9810 -8.8238

C 12.3061 8.6220 -10.2858

H 12.5901 7.6540 -9.8648

H 12.3991 8.5490 -11.3708

C 13.2571 9.6970 -9.7638

H 13.1171 9.8070 -8.6848

H 13.0041 10.6560 -10.2248

C 14.7151 9.3630 -10.0508

H 14.9961 8.4320 -9.5628

H 15.3691 10.1520 -9.6838

H 14.8781 9.2500 -11.1198

C 6.1731 9.0200 -14.0528

H 6.1871 9.2690 -12.9828

H 5.8991 9.9190 -14.6068

C 7.5571 8.5350 -14.4718

H 7.5761 8.3950 -15.5528

H 7.7471 7.5650 -14.0058

C 8.6131 9.5470 -14.0428

H 8.3731 10.5090 -14.4978

H 8.5611 9.6740 -12.9578

C 10.0341 9.1380 -14.4248

H 10.1131 9.0560 -15.5108

H 10.2581 8.1550 -14.0048

C 11.0491 10.1560 -13.9098

H 10.8071 11.1390 -14.3198

H 10.9631 10.2240 -12.8228

C 12.4901 9.8090 -14.2748

H 12.5931 9.7730 -15.3618

H 12.7371 8.8170 -13.8888

C 13.4651 10.8350 -13.7018

H 13.2111 11.8250 -14.0888

H 13.3491 10.8690 -12.6168

C 14.9201 10.5250 -14.0388

H 15.0511 10.5310 -15.1228

H 15.1721 9.5230 -13.6828

C 15.8701 11.5410 -13.4068

H 15.6091 12.5420 -13.7598

H 15.7381 11.5300 -12.3228

C 17.3321 11.2590 -13.7388

H 17.4661 11.2730 -14.8228

H 17.5981 10.2590 -13.3868

C 18.2681 12.2830 -13.1018

H 17.9881 13.2840 -13.4368

H 18.1501 12.2530 -12.0168

C 19.7271 12.0270 -13.4598

H 20.0371 11.0420 -13.1178

H 20.3711 12.7700 -12.9958

H 19.8691 12.0760 -14.5368

C 5.2061 9.6820 -18.0738

H 4.7881 10.6990 -18.1038

H 4.6031 9.0300 -18.7188

C 6.6461 9.6740 -18.5808

H 6.6291 9.8910 -19.6518

H 7.0461 8.6670 -18.4478

C 7.5471 10.6750 -17.8638

H 7.5241 10.4750 -16.7938

H 7.1661 11.6860 -18.0138

C 8.9831 10.5900 -18.3788

H 9.3461 9.5650 -18.2638

H 8.9991 10.8240 -19.4468

C 9.9231 11.5370 -17.6378

H 9.5631 12.5620 -17.7438

H 9.9091 11.2900 -16.5748

C 11.3571 11.4480 -18.1538

H 11.7131 10.4200 -18.0448

H 11.3781 11.6900 -19.2188

C 12.2991 12.3840 -17.4018

H 12.2311 12.1740 -16.3318

H 11.9831 13.4190 -17.5568

C 13.7521 12.2290 -17.8448

H 14.0711 11.1970 -17.6748

H 13.8311 12.4220 -18.9178

C 14.6821 13.1720 -17.0858

H 14.3871 14.2060 -17.2828

H 14.5711 12.9960 -16.0138

C 16.1471 12.9830 -17.4708

H 16.2651 13.1330 -18.5468

H 16.4501 11.9570 -17.2498

C 17.0601 13.9520 -16.7238

H 16.7281 14.9750 -16.9128

H 16.9741 13.7670 -15.6508

C 18.5171 13.8100 -17.1438

H 18.8711 12.7980 -16.9588

H 19.1461 14.5010 -16.5858

H 18.6311 14.0220 -18.2048

O 5.2111 7.9850 -14.2188

O 3.0681 7.2620 -13.0618

O 5.1901 9.2000 -16.7448

O -0.8099 8.2070 -16.3528

C -2.9119 7.9540 -12.3258

C -1.6159 7.8510 -11.8078

C -4.0009 7.9790 -11.4508

C -1.4149 7.7560 -10.4538

H -0.7869 7.8500 -12.4968

C -3.7979 7.8890 -10.0928

H -4.9999 8.0700 -11.8468

C -2.5059 7.7640 -9.5828

H -0.4189 7.6750 -10.0488

H -4.6359 7.9110 -9.4138

C -3.0969 7.9090 -23.0958

C -1.8679 7.8050 -23.7518

C -4.2789 7.9280 -23.8418

C -1.8279 7.7380 -25.1258

H -0.9599 7.7780 -23.1698

C -4.2359 7.8590 -25.2138

H -5.2199 7.9950 -23.3208

C -3.0079 7.7650 -25.8678

H -0.8939 7.6680 -25.6578

H -5.1429 7.8790 -25.7958

C -6.8439 8.4600 -18.3908

H -7.0279 7.5960 -17.7318

H -6.7719 9.3610 -17.7608

C 0.3831 8.3500 -17.1048

H 0.4871 7.4880 -17.7828

H 0.3241 9.2640 -17.7098

C -2.1979 4.9260 -20.6198

C -2.1349 4.5090 -26.2638

C -2.1649 4.5030 -27.4718

C -2.2419 4.5270 -28.8748

C -1.0699 4.5400 -29.6578

C -3.4929 4.5920 -29.4878

H -4.3569 4.6060 -28.8428

O -3.2379 4.8170 -19.9848

O -1.0409 5.1940 -20.0608

H -1.1129 5.2440 -19.0578

C -2.3419 4.5460 -39.9308

C -2.5319 4.5160 -34.2648

C -2.5249 4.5440 -33.0558

C -2.4529 4.5670 -31.6508

C -3.6189 4.6660 -30.8658

C -1.1989 4.5190 -31.0358

H -0.3319 4.4690 -31.6758

O -1.2819 4.6690 -40.5318

O -3.4949 4.3860 -40.5338

H -3.3929 4.3080 -41.5338

C -7.0649 5.7220 -31.5198

C -7.0719 5.7560 -32.9008

H -6.1819 5.4560 -33.4278

C -8.2299 6.1440 -33.5718

C -9.3839 6.4700 -32.8458

C -9.3459 6.4920 -31.4428

C -8.1779 6.1240 -30.7948

H -8.1199 6.1200 -29.7188

C -7.3059 5.7390 -35.7388

H -6.3769 6.2910 -35.5348

H -7.1319 4.6750 -35.5258

C -7.6769 5.9220 -37.2098

H -7.8989 6.9770 -37.3818

H -6.7929 5.6560 -37.7938

C -8.8659 5.0700 -37.6628

H -8.7849 4.0760 -37.2188

H -8.8179 4.9470 -38.7478

C -10.2149 5.6860 -37.2928

H -10.2259 5.9150 -36.2278

H -10.3379 6.6290 -37.8308

C -11.3839 4.7620 -37.6268

H -11.2739 3.8260 -37.0728

H -11.3619 4.5130 -38.6918

C -12.7279 5.3970 -37.2798

H -12.7189 5.7030 -36.2328

H -12.8689 6.2990 -37.8788

C -13.8979 4.4430 -37.5138

H -13.7599 3.5480 -36.9018

H -13.9039 4.1240 -38.5598

C -15.2419 5.0830 -37.1738

H -15.2249 5.4310 -36.1398

H -15.3979 5.9570 -37.8098

C -16.4049 4.1100 -37.3568

H -16.2719 3.2580 -36.6838

H -16.3929 3.7200 -38.3788

C -17.7559 4.7650 -37.0838

H -17.8989 5.5970 -37.7748

H -17.7619 5.1740 -36.0718

C -18.9149 3.7820 -37.2338

H -18.8819 3.3330 -38.2298

H -18.7989 2.9720 -36.5088

C -20.2659 4.4570 -37.0288

H -20.4089 5.2490 -37.7608

H -21.0749 3.7390 -37.1378

H -20.3269 4.8950 -36.0358

C -11.5459 5.7240 -33.3698

H -11.5339 5.0900 -34.2688

H -11.3169 5.0890 -32.5078

C -12.9169 6.3620 -33.1918

H -12.9099 6.9470 -32.2728

H -13.0869 7.0490 -34.0228

C -14.0289 5.3180 -33.1488

H -13.8599 4.6360 -32.3118

H -14.0029 4.7220 -34.0648

C -15.4019 5.9700 -33.0078

H -15.4429 6.5270 -32.0698

H -15.5399 6.6870 -33.8208

C -16.5389 4.9520 -33.0438

H -16.4229 4.2430 -32.2208

H -16.4859 4.3830 -33.9758

C -17.9029 5.6300 -32.9428

H -17.9649 6.1760 -31.9998

H -17.9989 6.3610 -33.7498

C -19.0569 4.6350 -33.0288

H -18.9779 3.9160 -32.2098

H -18.9849 4.0750 -33.9638

C -20.4139 5.3330 -32.9578

H -20.4859 5.8840 -32.0188

H -20.4889 6.0600 -33.7708

C -21.5789 4.3520 -33.0578

H -21.5219 3.6380 -32.2328

H -21.4969 3.7840 -33.9878

C -22.9269 5.0660 -33.0188

H -23.0039 5.6350 -32.0898

H -22.9819 5.7750 -33.8488

C -24.1009 4.0950 -33.1098

H -24.0519 3.3890 -32.2778

H -24.0219 3.5200 -34.0358

C -25.4419 4.8190 -33.0768

H -25.5179 5.5200 -33.9058

H -26.2619 4.1090 -33.1518

H -25.5539 5.3730 -32.1478

C -10.4839 6.9030 -29.3938

H -10.2039 5.9280 -28.9698

H -9.7529 7.6540 -29.0658

C -11.8809 7.2890 -28.9218

H -11.8299 7.5590 -27.8658

H -12.1949 8.1690 -29.4828

C -12.8969 6.1660 -29.1198

H -12.8139 5.7880 -30.1398

H -12.6709 5.3430 -28.4378

C -14.3199 6.6600 -28.8758

H -14.5469 7.4450 -29.6018

H -14.3829 7.1060 -27.8818

C -15.3669 5.5570 -29.0008

H -15.2209 4.8200 -28.2078

H -15.2409 5.0420 -29.9558

C -16.7799 6.1280 -28.9168

H -16.9219 6.8380 -29.7348

H -16.8889 6.6800 -27.9808

C -17.8649 5.0580 -28.9968

H -17.7429 4.4850 -29.9188

H -17.7559 4.3640 -28.1598

C -19.2589 5.6790 -28.9658

H -19.3639 6.3600 -29.8148

H -19.3669 6.2700 -28.0538

C -20.3719 4.6370 -29.0228

H -20.2769 3.9550 -28.1748

H -20.2679 4.0440 -29.9348

C -21.7529 5.2860 -28.9938

H -21.8449 5.8910 -28.0898

H -21.8529 5.9550 -29.8528

C -22.8819 4.2610 -29.0248

H -22.7809 3.5870 -28.1708

H -22.7999 3.6590 -29.9328

C -24.2509 4.9280 -28.9828

H -24.3779 5.5910 -29.8368

H -25.0439 4.1840 -29.0078

H -24.3609 5.5170 -28.0748

O -10.5539 6.7360 -33.5208

O -8.3529 6.2230 -34.9238

O -10.4959 6.8370 -30.8068

O -4.7979 4.8500 -31.5188

C 2.3741 5.1980 -28.6978

C 2.1361 5.9990 -27.5918

H 1.1611 6.4410 -27.4758

C 3.1421 6.1970 -26.6498

C 4.4001 5.6110 -26.8498

C 4.6441 4.8420 -27.9948

C 3.6171 4.6240 -28.9068

H 3.7751 4.0010 -29.7728

C 1.7851 7.5300 -25.2108

H 0.9631 6.8190 -25.3728

H 1.6301 8.3990 -25.8678

C 1.7871 7.9650 -23.7458

H 1.8781 7.0780 -23.1168

H 0.8161 8.4220 -23.5488

C 2.9051 8.9520 -23.3988

H 3.1401 9.5540 -24.2758

H 2.5531 9.6260 -22.6128

C 4.1791 8.2630 -22.9158

H 4.4991 7.5370 -23.6628

H 3.9651 7.7210 -21.9898

C 5.2941 9.2750 -22.6668

H 5.5691 9.7420 -23.6148

H 4.9261 10.0650 -22.0048

C 6.5311 8.6370 -22.0388

H 6.8341 7.7780 -22.6388

H 6.2901 8.2720 -21.0368

C 7.6871 9.6290 -21.9638

H 7.9251 9.9520 -22.9788

H 7.3711 10.5130 -21.4018

C 8.9441 9.0470 -21.3228

H 9.2111 8.1190 -21.8318

H 8.7461 8.8040 -20.2758

C 10.1151 10.0230 -21.4098

H 10.3171 10.2420 -22.4608

H 9.8381 10.9630 -20.9248

C 11.3861 9.4800 -20.7618

H 11.2061 9.3070 -19.6988

H 11.6391 8.5180 -21.2148

C 12.5631 10.4380 -20.9268

H 12.3031 11.4050 -20.4888

H 12.7501 10.5970 -21.9918

C 13.8291 9.9110 -20.2658

H 13.6741 9.7780 -19.1978

H 14.6561 10.6040 -20.4088

H 14.1111 8.9500 -20.6918

C 6.2901 6.8340 -26.1818

H 5.8601 7.7850 -25.8388

H 6.4631 6.9090 -27.2618

C 7.6071 6.5590 -25.4668

H 8.0371 5.6370 -25.8648

H 7.4011 6.3940 -24.4088

C 8.5751 7.7220 -25.6448

H 8.6941 7.9290 -26.7108

H 8.1411 8.6130 -25.1868

C 9.9451 7.4410 -25.0338

H 10.3701 6.5430 -25.4898

H 9.8351 7.2430 -23.9658

C 10.9021 8.6130 -25.2358

H 10.9791 8.8370 -26.3028

H 10.4891 9.4960 -24.7438

C 12.2961 8.3350 -24.6798

H 12.7231 7.4630 -25.1828

H 12.2201 8.0950 -23.6178

C 13.2261 9.5310 -24.8598

H 13.2781 9.7950 -25.9188

H 12.8071 10.3910 -24.3308

C 14.6341 9.2570 -24.3378

H 15.0801 8.4390 -24.9098

H 14.5781 8.9350 -23.2958

C 15.5271 10.4910 -24.4338

H 15.5271 10.8560 -25.4648

H 15.1141 11.2840 -23.8068

C 16.9631 10.2040 -24.0028

H 17.3801 9.4140 -24.6318

H 16.9661 9.8400 -22.9728

C 17.8461 11.4450 -24.1028

H 17.7931 11.8420 -25.1198

H 17.4631 12.2140 -23.4288

C 19.2991 11.1450 -23.7578

H 19.3781 10.7540 -22.7458

H 19.9031 12.0480 -23.8238

H 19.7091 10.4060 -24.4428

C 6.2521 3.6310 -29.2798

H 6.0211 4.2220 -30.1778

H 5.6911 2.6870 -29.3268

C 7.7511 3.3550 -29.2148

H 8.0421 2.7840 -30.0988

H 7.9591 2.7520 -28.3298

C 8.5401 4.6590 -29.1388

H 8.2251 5.1990 -28.2458

H 8.2971 5.2750 -30.0078

C 10.0471 4.4310 -29.0878

H 10.2831 3.7360 -28.2788

H 10.3801 3.9730 -30.0228

C 10.8011 5.7390 -28.8638

H 10.5241 6.4520 -29.6438

H 10.4991 6.1660 -27.9058

C 12.3141 5.5430 -28.8748

H 12.5881 4.8070 -28.1148

H 12.6221 5.1450 -29.8448

C 13.0601 6.8470 -28.6048

H 12.7351 7.2530 -27.6438

H 12.7991 7.5780 -29.3738

C 14.5731 6.6520 -28.5808

H 14.8301 5.9320 -27.7988

H 14.9021 6.2320 -29.5338

C 15.3121 7.9620 -28.3218

H 15.1031 8.6610 -29.1348

H 14.9331 8.4080 -27.3988

C 16.8201 7.7670 -28.1968

H 17.2061 7.3130 -29.1128

H 17.0281 7.0760 -27.3748

C 17.5431 9.0870 -27.9398

H 17.3651 9.7630 -28.7778

H 17.1241 9.5520 -27.0448

C 19.0421 8.8990 -27.7508

H 19.2431 8.2480 -26.9018

H 19.5281 9.8560 -27.5718

H 19.4871 8.4490 -28.6358

O 5.3881 5.7680 -25.9068

O 3.0201 6.9190 -25.5128

O 5.9031 4.3530 -28.1178

O 0.1031 4.5690 -28.9818

C -2.1319 4.5600 -24.8578

C -0.9279 4.6030 -24.1478

C -3.3469 4.5930 -24.1628

C -0.9389 4.7140 -22.7748

H 0.0051 4.5520 -24.6888

C -3.3529 4.6930 -22.7918

H -4.2729 4.5480 -24.7158

C -2.1509 4.7720 -22.0868

H -0.0149 4.7570 -22.2198

H -4.2789 4.7260 -22.2378

C -2.5029 4.5200 -35.6718

C -1.2759 4.5860 -36.3408

C -3.6849 4.4630 -36.4168

C -1.2319 4.6120 -37.7158

H -0.3629 4.6040 -35.7638

C -3.6399 4.4760 -37.7928

H -4.6309 4.4030 -35.8998

C -2.4139 4.5530 -38.4548

H -0.2899 4.6670 -38.2398

H -4.5469 4.4230 -38.3748

C -5.8899 5.2390 -30.7138

H -6.2229 4.3860 -30.1018

H -5.5779 6.0470 -30.0378

C 1.2651 4.9550 -29.6878

H 1.5631 4.1730 -30.3988

H 1.0551 5.8800 -30.2468

C -2.1699 4.2140 -43.6178

C -2.0219 3.7880 -49.2618

C -2.0609 3.7940 -50.4708

C -2.1249 3.8270 -51.8748

C -0.9429 3.9510 -52.6358

C -3.3669 3.7880 -52.5098

H -4.2429 3.7200 -51.8838

O -3.2139 4.0470 -43.0028

O -1.0449 4.5610 -43.0368

H -1.1459 4.6190 -42.0378

C -2.9449 4.0690 -62.8808

C -2.3669 3.7590 -57.2628

C -2.3469 3.8110 -56.0548

C -2.2849 3.8540 -54.6508

C -3.4729 3.8590 -53.8898

C -1.0429 3.9190 -54.0158

H -0.1629 3.9460 -54.6388

O -2.0069 4.3030 -63.6328

O -4.1879 3.9590 -63.2978

H -4.2759 4.0850 -64.2898

C -6.9119 4.8330 -54.6678

C -6.9089 4.7640 -56.0498

H -6.0439 4.3550 -56.5458

C -8.0219 5.2060 -56.7638

C -9.1449 5.6870 -56.0788

C -9.1159 5.8050 -54.6818

C -7.9899 5.3850 -53.9888

H -7.9439 5.4620 -52.9148

C -7.1129 4.6470 -58.9078

H -6.1589 5.1560 -58.7078

H -6.9959 3.5800 -58.6708

C -7.4729 4.8200 -60.3828

H -7.6609 5.8780 -60.5708

H -6.5969 4.5200 -60.9598

C -8.6879 4.0030 -60.8308

H -8.6649 3.0260 -60.3438

H -8.6219 3.8340 -61.9088

C -10.0139 4.6970 -60.5218

H -10.0329 4.9860 -59.4718

H -10.0809 5.6110 -61.1158

C -11.2159 3.8070 -60.8308

H -11.1739 2.9120 -60.2048

H -11.1689 3.4800 -61.8728

C -12.5389 4.5310 -60.5848

H -12.5479 4.9250 -59.5688

H -12.6139 5.3840 -61.2628

C -13.7469 3.6180 -60.7848

H -13.7099 2.8030 -60.0568

H -13.6999 3.1660 -61.7798

C -15.0679 4.3710 -60.6368

H -15.0979 4.8620 -59.6628

H -15.1219 5.1530 -61.3968

C -16.2789 3.4520 -60.7788

H -16.2919 2.7330 -59.9548

H -16.1909 2.8770 -61.7058

C -17.5899 4.2330 -60.7968

H -17.5769 4.9310 -61.6368

H -17.6739 4.8220 -59.8808

C -18.8089 3.3230 -60.9248

H -18.6959 2.6900 -61.8078

H -18.8639 2.6620 -60.0568

C -20.1019 4.1210 -61.0398

H -20.0759 4.7590 -61.9208

H -20.9589 3.4560 -61.1258

H -20.2409 4.7510 -60.1648

C -11.3219 5.0530 -56.6508

H -11.2379 4.3290 -57.4748

H -11.2149 4.5030 -55.7088

C -12.6779 5.7450 -56.6908

H -12.7489 6.4200 -55.8378

H -12.7319 6.3500 -57.5968

C -13.8259 4.7410 -56.6668

H -13.7509 4.1210 -55.7698

H -13.7459 4.0750 -57.5298

C -15.1839 5.4380 -56.6918

H -15.2849 6.0670 -55.8058

H -15.2319 6.0940 -57.5638

C -16.3429 4.4480 -56.7488

H -16.3029 3.7900 -55.8778

H -16.2399 3.8210 -57.6368

C -17.6939 5.1570 -56.7878

H -17.8109 5.7640 -55.8878

H -17.7159 5.8360 -57.6438

C -18.8579 4.1770 -56.8958

H -18.8429 3.5010 -56.0368

H -18.7339 3.5680 -57.7938

C -20.2059 4.8890 -56.9518

H -20.3349 5.4900 -56.0488

H -20.2189 5.5730 -57.8048

C -21.3699 3.9100 -57.0788

H -21.3639 3.2320 -56.2218

H -21.2369 3.3050 -57.9778

C -22.7159 4.6270 -57.1458

H -22.8359 5.2500 -56.2568

H -22.7299 5.2860 -58.0168

C -23.8859 3.6500 -57.2348

H -23.8769 2.9960 -56.3598

H -23.7629 3.0210 -58.1198

C -25.2259 4.3720 -57.3098

H -25.2629 5.0200 -58.1828

H -26.0429 3.6570 -57.3798

H -25.3819 4.9820 -56.4228

C -10.2989 6.4280 -52.7028

H -10.0729 5.4660 -52.2198

H -9.5659 7.1750 -52.3688

C -11.7149 6.8670 -52.3448

H -11.7439 7.1450 -51.2908

H -11.9539 7.7510 -52.9378

C -12.7369 5.7680 -52.6278

H -12.5659 5.3780 -53.6318

H -12.5939 4.9460 -51.9208

C -14.1679 6.2870 -52.5268

H -14.3139 7.0660 -53.2788

H -14.3199 6.7460 -51.5478

C -15.2099 5.1920 -52.7358

H -15.1209 4.4460 -51.9418

H -15.0209 4.6850 -53.6838

C -16.6239 5.7660 -52.7398

H -16.7179 6.4720 -53.5688

H -16.7879 6.3220 -51.8158

C -17.7029 4.6950 -52.8768

H -17.5389 4.1270 -53.7948

H -17.6289 3.9950 -52.0408

C -19.0989 5.3100 -52.9028

H -19.1849 5.9640 -53.7738

H -19.2359 5.9280 -52.0128

C -20.2039 4.2580 -52.9578

H -20.1359 3.6160 -52.0768

H -20.0599 3.6280 -53.8388

C -21.5899 4.8940 -53.0148

H -21.7359 5.5210 -52.1318

H -21.6519 5.5410 -53.8938

C -22.7049 3.8540 -53.0858

H -22.6549 3.2110 -52.2038

H -22.5519 3.2230 -53.9638

C -24.0819 4.5020 -53.1638

H -24.1559 5.1310 -54.0478

H -24.8609 3.7440 -53.2188

H -24.2639 5.1200 -52.2868

O -10.2829 6.0190 -56.7788

O -8.1409 5.2020 -58.1178

O -10.2419 6.2970 -54.1078

O -4.6449 3.9550 -54.5738

C 2.4051 4.9030 -51.5958

C 2.0671 5.6600 -50.4858

H 1.0591 6.0310 -50.3998

C 3.0141 5.9000 -49.4968

C 4.3201 5.4050 -49.6458

C 4.6661 4.6860 -50.7988

C 3.6951 4.4270 -51.7608

H 3.9321 3.8410 -52.6338

C 1.5221 7.1290 -48.0908

H 0.7301 6.4070 -48.3318

H 1.3791 8.0230 -48.7158

C 1.4311 7.4930 -46.6098

H 1.4861 6.5770 -46.0178

H 0.4511 7.9430 -46.4498

C 2.5241 8.4600 -46.1498

H 2.7601 9.1440 -46.9638

H 2.1461 9.0500 -45.3098

C 3.8051 7.7490 -45.7198

H 4.1251 7.0790 -46.5158

H 3.6051 7.1470 -44.8298

C 4.9201 8.7480 -45.4258

H 5.1131 9.3340 -46.3268

H 4.6001 9.4390 -44.6398

C 6.2111 8.0570 -44.9968

H 6.4591 7.2920 -45.7348

H 6.0671 7.5560 -44.0358

C 7.3651 9.0500 -44.9008

H 7.4481 9.5640 -45.8598

H 7.1381 9.8070 -44.1448

C 8.7001 8.3870 -44.5728

H 8.8691 7.5600 -45.2658

H 8.6691 7.9720 -43.5628

C 9.8561 9.3780 -44.6868

H 9.8941 9.7550 -45.7118

H 9.6681 10.2340 -44.0338

C 11.2051 8.7590 -44.3328

H 11.1911 8.4280 -43.2918

H 11.3741 7.8800 -44.9588

C 12.3511 9.7470 -44.5358

H 12.1701 10.6390 -43.9308

H 12.3701 10.0590 -45.5828

C 13.7011 9.1490 -44.1618

H 13.7101 8.8550 -43.1148

H 14.4991 9.8720 -44.3208

H 13.9091 8.2710 -44.7688

C 6.2721 6.5070 -48.8838

H 6.0211 7.4730 -48.4238

H 6.3771 6.6640 -49.9598

C 7.5851 5.9760 -48.3158

H 7.8451 5.0520 -48.8348

H 7.4471 5.7350 -47.2608

C 8.6851 7.0190 -48.4858

H 8.7111 7.3360 -49.5318

H 8.4321 7.8960 -47.8878

C 10.0711 6.5180 -48.0868

H 10.3271 5.6370 -48.6798

H 10.0671 6.2150 -47.0378

C 11.1221 7.6060 -48.3018

H 11.0671 7.9520 -49.3378

H 10.8881 8.4580 -47.6608

C 12.5461 7.1370 -48.0138

H 12.7921 6.2940 -48.6638

H 12.6141 6.7870 -46.9818

C 13.5531 8.2630 -48.2348

H 13.4501 8.6430 -49.2558

H 13.3221 9.0880 -47.5568

C 14.9971 7.8220 -48.0128

H 15.2511 7.0340 -48.7248

H 15.0991 7.4050 -47.0078

C 15.9701 8.9870 -48.1738

H 15.8541 9.4170 -49.1728

H 15.7191 9.7670 -47.4508

C 17.4241 8.5670 -47.9778

H 17.6841 7.8050 -48.7158

H 17.5401 8.1220 -46.9868

C 18.3831 9.7480 -48.1128

H 18.2711 10.1900 -49.1068

H 18.1171 10.5120 -47.3788

C 19.8351 9.3350 -47.9108

H 19.9751 8.9020 -46.9228

H 20.4941 10.1950 -48.0048

H 20.1281 8.5970 -48.6538

C 6.3311 3.4270 -51.9528

H 6.1801 3.9390 -52.9138

H 5.7211 2.5140 -51.9408

C 7.8061 3.0760 -51.7858

H 8.0751 2.3410 -52.5468

H 7.9481 2.6210 -50.8038

C 8.6881 4.3130 -51.9148

H 8.4151 5.0190 -51.1298

H 8.4921 4.7900 -52.8778

C 10.1741 3.9870 -51.8128

H 10.3711 3.4620 -50.8758

H 10.4551 3.3180 -52.6308

C 11.0251 5.2510 -51.8768

H 10.7611 5.8150 -52.7748

H 10.7971 5.8830 -51.0158

C 12.5211 4.9500 -51.8978

H 12.7881 4.3730 -51.0088

H 12.7541 4.3360 -52.7708

C 13.3531 6.2290 -51.9418

H 13.1261 6.8360 -51.0638

H 13.0701 6.8120 -52.8218

C 14.8521 5.9450 -51.9808

H 15.1351 5.3850 -51.0868

H 15.0831 5.3190 -52.8458

C 15.6711 7.2310 -52.0528

H 15.4211 7.7670 -52.9728

H 15.4001 7.8750 -51.2148

C 17.1731 6.9650 -52.0138

H 17.4531 6.3330 -52.8608

H 17.4171 6.4170 -51.1008

C 17.9811 8.2590 -52.0548

H 17.7571 8.7950 -52.9808

H 17.6771 8.8960 -51.2228

C 19.4801 8.0010 -51.9698

H 19.7231 7.4700 -51.0518

H 20.0311 8.9390 -51.9788

H 19.8141 7.3970 -52.8108

O 5.2141 5.5890 -48.6228

O 2.7921 6.5700 -48.3448

O 5.9591 4.2710 -50.8838

O 0.2101 4.0860 -51.9398

C -2.0269 3.8310 -47.8558

C -0.8419 4.0300 -47.1388

C -3.2379 3.7030 -47.1648

C -0.8749 4.1430 -45.7658

H 0.0931 4.0980 -47.6738

C -3.2639 3.8070 -45.7938

H -4.1469 3.5320 -47.7208

C -2.0879 4.0480 -45.0828

H 0.0331 4.3080 -45.2068

H -4.1899 3.7190 -45.2458

C -2.4709 3.7590 -58.6648

C -1.3879 4.1160 -59.4748

C -3.6909 3.4210 -59.2608

C -1.5379 4.1910 -60.8398

H -0.4369 4.3420 -59.0168

C -3.8389 3.4950 -60.6258

H -4.5099 3.1030 -58.6338

C -2.7729 3.9080 -61.4268

H -0.7139 4.4840 -61.4718

H -4.7759 3.2310 -61.0918

C -5.7889 4.2960 -53.8178

H -6.1549 3.4060 -53.2808

H -5.5269 5.0620 -53.0768

C 1.3471 4.5820 -52.6188

H 1.7281 3.8380 -53.3318

H 1.0681 5.4920 -53.1738

C -1.5189 13.7140 -31.0288

C -1.3139 13.7270 -36.6928

C -1.3809 13.6740 -37.8998

C -1.5659 13.6170 -39.2928

C -0.5009 13.3490 -40.1768

C -2.8619 13.7880 -39.7908

H -3.6509 13.9500 -39.0728

O -2.6029 13.7300 -30.4628

O -0.3749 13.6870 -30.3808

H -0.5139 13.7080 -29.3828

C -1.7889 13.1650 -50.2808

C -2.1589 13.5640 -44.6418

C -2.2019 13.5770 -43.4328

C -2.0539 13.5720 -42.0338

C -3.1359 13.7380 -41.1478

C -0.7679 13.3630 -41.5358

H 0.0051 13.1900 -42.2658

O -0.7099 13.1190 -50.8578

O -2.9429 13.0980 -50.8988

H -2.8319 13.0220 -51.8978

C -6.7499 13.6240 -41.6968

C -6.7119 13.8740 -43.0558

H -5.7609 14.0820 -43.5148

C -7.8879 13.8510 -43.7968

C -9.1119 13.5860 -43.1688

C -9.1439 13.3540 -41.7848

C -7.9549 13.3510 -41.0648

H -7.9569 13.1500 -40.0048

C -6.7689 14.1340 -45.8708

H -6.1429 14.9810 -45.5528

H -6.2109 13.2010 -45.6978

C -7.0979 14.2770 -47.3548

H -7.6209 15.2220 -47.5098

H -6.1499 14.3230 -47.8948

C -7.9599 13.1280 -47.8848

H -7.6999 12.2110 -47.3538

H -7.7429 12.9730 -48.9458

C -9.4529 13.3990 -47.7138

H -9.6499 13.6590 -46.6738

H -9.7299 14.2560 -48.3328

C -10.3119 12.1980 -48.1048

H -10.1119 11.3700 -47.4228

H -10.0509 11.8690 -49.1158

C -11.7959 12.5500 -48.0558

H -12.0209 12.9850 -47.0808

H -12.0119 13.3100 -48.8118

C -12.6979 11.3400 -48.2818

H -12.4989 10.6030 -47.5008

H -12.4579 10.8760 -49.2408

C -14.1729 11.7320 -48.2598

H -14.3999 12.2180 -47.3088

H -14.3629 12.4610 -49.0528

C -15.1029 10.5370 -48.4458

H -14.9179 9.8140 -47.6468

H -14.8759 10.0430 -49.3928

C -16.5739 10.9440 -48.4238

H -16.7599 11.6650 -49.2238

H -16.7969 11.4390 -47.4758

C -17.5039 9.7470 -48.5978

H -17.2729 9.2490 -49.5418

H -17.3189 9.0300 -47.7938

C -18.9719 10.1500 -48.5828

H -19.1779 10.8670 -49.3738

H -19.6099 9.2820 -48.7338

H -19.2329 10.6080 -47.6318

C -11.0719 12.4890 -43.9748

H -10.8309 11.9080 -44.8748

H -10.8949 11.8520 -43.1048

C -12.5269 12.9440 -44.0108

H -12.7439 13.4890 -43.0908

H -12.6519 13.6410 -44.8428

C -13.4749 11.7630 -44.1678

H -13.3109 11.0610 -43.3468

H -13.2399 11.2360 -45.0938

C -14.9379 12.1990 -44.1828

H -15.1689 12.7310 -43.2568

H -15.0999 12.8970 -45.0078

C -15.8789 11.0080 -44.3348

H -15.7009 10.3050 -43.5158

H -15.6499 10.4860 -45.2658

C -17.3499 11.4140 -44.3318

H -17.5829 11.9340 -43.4008

H -17.5369 12.1120 -45.1518

C -18.2619 10.2000 -44.4808

H -18.0589 9.5000 -43.6658

H -18.0269 9.6860 -45.4148

C -19.7429 10.5640 -44.4648

H -19.9849 11.0620 -43.5228

H -19.9549 11.2700 -45.2718

C -20.6219 9.3270 -44.6278

H -20.3829 8.6150 -43.8328

H -20.3889 8.8420 -45.5778

C -22.1119 9.6490 -44.5758

H -22.3459 10.1310 -43.6238

H -22.3609 10.3540 -45.3728

C -22.9649 8.3920 -44.7278

H -22.6819 7.6740 -43.9538

H -22.7539 7.9290 -45.6948

C -24.4549 8.6850 -44.6178

H -24.7619 9.3960 -45.3818

H -25.0339 7.7730 -44.7468

H -24.6899 9.1070 -43.6428

C -10.5179 13.1350 -39.8328

H -10.2059 12.1560 -39.4398

H -9.8939 13.9120 -39.3748

C -11.9819 13.4100 -39.4978

H -12.0739 13.5010 -38.4128

H -12.2569 14.3670 -39.9458

C -12.9249 12.3250 -40.0108

H -12.7589 12.1890 -41.0798

H -12.6959 11.3800 -39.5138

C -14.3849 12.6980 -39.7598

H -14.6179 13.6190 -40.2998

H -14.5269 12.9010 -38.6938

C -15.3509 11.6000 -40.1948

H -15.1509 10.6960 -39.6168

H -15.1789 11.3650 -41.2458

C -16.8129 12.0010 -40.0068

H -17.0389 12.8590 -40.6438

H -16.9749 12.3110 -38.9718

C -17.7609 10.8530 -40.3438

H -17.5669 10.5110 -41.3618

H -17.5579 10.0150 -39.6728

C -19.2299 11.2490 -40.2198

H -19.4539 12.0420 -40.9378

H -19.4149 11.6520 -39.2208

C -20.1569 10.0610 -40.4648

H -19.9449 9.2860 -39.7268

H -19.9519 9.6400 -41.4508

C -21.6329 10.4420 -40.3778

H -21.8319 10.9110 -39.4108

H -21.8639 11.1790 -41.1518

C -22.5429 9.2280 -40.5458

H -22.3249 8.5050 -39.7558

H -22.3289 8.7460 -41.5008

C -24.0179 9.6050 -40.4898

H -24.2589 10.3230 -41.2698

H -24.6429 8.7250 -40.6268

H -24.2619 10.0530 -39.5288

O -10.2399 13.6430 -43.9408

O -7.9649 14.0980 -45.1288

O -10.3759 13.1550 -41.2378

O -4.3669 13.8370 -41.7028

C 3.0011 12.3240 -39.8698

C 3.0811 11.6730 -38.6518

H 2.1701 11.3670 -38.1648

C 4.3241 11.4510 -38.0698

C 5.4941 11.8620 -38.7278

C 5.4031 12.4940 -39.9758

C 4.1511 12.7330 -40.5248

H 4.0581 13.2370 -41.4728

C 3.4011 10.5860 -36.0528

H 2.7441 9.8470 -36.5358

H 2.8291 11.5120 -35.8958

C 3.8881 10.0380 -34.7128

H 4.3331 9.0560 -34.8818

H 3.0061 9.9100 -34.0798

C 4.9081 10.9490 -34.0158

H 4.7111 11.9880 -34.2848

H 4.7871 10.8630 -32.9318

C 6.3461 10.5890 -34.3868

H 6.4481 10.6040 -35.4708

H 6.5491 9.5720 -34.0398

C 7.3821 11.5280 -33.7698

H 7.3001 12.5140 -34.2328

H 7.1841 11.6510 -32.7018

C 8.7981 10.9880 -33.9658

H 8.9251 10.7010 -35.0118

H 8.9241 10.0880 -33.3578

C 9.8801 11.9970 -33.5898

H 9.7861 12.8800 -34.2258

H 9.7331 12.3190 -32.5558

C 11.2841 11.4160 -33.7418

H 11.4161 11.0530 -34.7648

H 11.3931 10.5590 -33.0728

C 12.3711 12.4400 -33.4258

H 12.2741 13.2900 -34.1048

H 12.2261 12.8130 -32.4098

C 13.7761 11.8560 -33.5498

H 13.8761 11.0060 -32.8698

H 13.9281 11.4840 -34.5658

C 14.8551 12.8860 -33.2228

H 14.6941 13.2650 -32.2118

H 14.7671 13.7300 -33.9098

C 16.2571 12.2970 -33.3228

H 16.3741 11.4660 -32.6298

H 17.0061 13.0490 -33.0858

H 16.4461 11.9290 -34.3298

C 7.4321 12.7740 -37.7328

H 7.1021 13.0720 -36.7268

H 7.2381 13.6050 -38.4178

C 8.9171 12.4370 -37.7338

H 9.1751 12.0710 -38.7278

H 9.0901 11.6290 -37.0218

C 9.7981 13.6300 -37.3768

H 9.6231 14.4450 -38.0838

H 9.5361 13.9960 -36.3818

C 11.2721 13.2330 -37.4068

H 11.5171 12.8530 -38.4018

H 11.4351 12.4220 -36.6948

C 12.2161 14.3840 -37.0678

H 12.0681 15.2040 -37.7758

H 11.9851 14.7660 -36.0708

C 13.6711 13.9240 -37.1148

H 13.8931 13.5470 -38.1168

H 13.8051 13.0970 -36.4148

C 14.6611 15.0330 -36.7688

H 14.5421 15.8630 -37.4698

H 14.4461 15.4150 -35.7688

C 16.0991 14.5240 -36.8218

H 16.3041 14.1400 -37.8248

H 16.2111 13.6930 -36.1208

C 17.1211 15.6050 -36.4808

H 17.0101 16.4410 -37.1758

H 16.9281 15.9840 -35.4748

C 18.5511 15.0730 -36.5548

H 18.7301 14.6590 -37.5508

H 18.6711 14.2590 -35.8358

C 19.5891 16.1530 -36.2688

H 19.4531 16.9800 -36.9688

H 19.4351 16.5450 -35.2608

C 21.0101 15.6170 -36.3928

H 21.1721 14.8000 -35.6928

H 21.7371 16.3980 -36.1828

H 21.1891 15.2430 -37.3998

C 6.5181 13.5460 -41.8038

H 6.0341 14.5220 -41.6548

H 5.9371 12.9740 -42.5408

C 7.9361 13.7370 -42.3288

H 7.8711 14.2760 -43.2758

H 8.3711 12.7560 -42.5248

C 8.8331 14.5010 -41.3588

H 8.9411 13.9190 -40.4438

H 8.3661 15.4530 -41.0978

C 10.2061 14.7560 -41.9748

H 10.6131 13.8110 -42.3428

H 10.0961 15.4210 -42.8338

C 11.1971 15.3660 -40.9878

H 10.7931 16.2990 -40.5868

H 11.3391 14.6800 -40.1498

C 12.5441 15.6360 -41.6538

H 12.8931 14.7170 -42.1328

H 12.4151 16.3860 -42.4378

C 13.6081 16.1120 -40.6678

H 13.7391 15.3550 -39.8908

H 13.2731 17.0310 -40.1808

C 14.9471 16.3580 -41.3598

H 15.2371 15.4590 -41.9078

H 14.8371 17.1640 -42.0898

C 16.0531 16.7140 -40.3708

H 15.7871 17.6310 -39.8378

H 16.1391 15.9170 -39.6288

C 17.4021 16.9010 -41.0618

H 17.3451 17.7480 -41.7498

H 17.6301 16.0120 -41.6558

C 18.5311 17.1320 -40.0608

H 18.3141 18.0270 -39.4718

H 18.5751 16.2850 -39.3738

C 19.8811 17.2930 -40.7478

H 20.1211 16.4030 -41.3258

H 20.6681 17.4480 -40.0138

H 19.8681 18.1450 -41.4238

O 6.7091 11.6150 -38.1338

O 4.5191 10.8520 -36.8688

O 6.5741 12.8480 -40.5758

O 0.7191 13.0820 -39.6438

C -1.3199 13.7500 -35.2858

C -0.1529 13.5580 -34.5398

C -2.5349 13.9460 -34.6178

C -0.1989 13.5550 -33.1628

H 0.7841 13.4140 -35.0588

C -2.5799 13.9290 -33.2438

H -3.4349 14.0990 -35.1948

C -1.4139 13.7340 -32.5018

H 0.7011 13.4150 -32.5838

H -3.5119 14.0640 -32.7158

C -2.0869 13.5190 -46.0468

C -0.8409 13.5950 -46.6778

C -3.2399 13.3700 -46.8218

C -0.7509 13.4930 -48.0478

H 0.0451 13.7310 -46.0758

C -3.1469 13.2690 -48.1918

H -4.1979 13.3210 -46.3318

C -1.9009 13.3150 -48.8158

H 0.2031 13.5450 -48.5478

H -4.0319 13.1380 -48.7938

C -5.4919 13.6860 -40.8668

H -5.3909 12.7670 -40.2718

H -5.5669 14.5390 -40.1728

C 1.6831 12.5710 -40.5458

H 1.3031 11.6300 -40.9738

H 1.8481 13.2850 -41.3628

C -0.8349 13.7580 -7.9678

C -1.2979 14.1750 -13.5978

C -1.4529 14.1810 -14.7968

C -1.6929 14.1810 -16.1828

C -0.6309 14.0760 -17.1048

C -3.0109 14.2380 -16.6438

H -3.7949 14.2800 -15.9048

O -1.8309 13.7490 -7.2548

O 0.3861 13.6520 -7.4858

H 0.3901 13.5800 -6.4868

C -1.8679 13.9450 -27.1558

C -2.3969 14.2840 -21.5218

C -2.4219 14.2620 -20.3118

C -2.2449 14.2190 -18.9168

C -3.3129 14.2240 -17.9978

C -0.9299 14.1290 -18.4558

H -0.1539 14.0840 -19.2028

O -0.7719 13.8870 -27.6968

O -2.9999 13.8950 -27.8168

H -2.8509 13.8320 -28.8098

C -6.8689 13.6080 -18.4908

C -6.7629 13.2720 -19.8288

H -5.7819 13.1100 -20.2418

C -7.9089 13.1750 -20.6068

C -9.1739 13.3790 -20.0308

C -9.2719 13.6720 -18.6628

C -8.1119 13.7880 -17.9058

H -8.1659 14.0350 -16.8568

C -6.7379 12.6210 -22.6158

H -5.9449 13.3270 -22.3378

H -6.4079 11.6070 -22.3428

C -6.9899 12.7090 -24.1208

H -7.2219 13.7430 -24.3808

H -6.0589 12.4350 -24.6218

C -8.1329 11.8030 -24.5858

H -8.1409 10.9000 -23.9748

H -7.9609 11.5080 -25.6248

C -9.4999 12.4760 -24.4758

H -9.6119 12.8900 -23.4748

H -9.5569 13.3000 -25.1928

C -10.6379 11.4920 -24.7328

H -10.5819 10.6850 -23.9998

H -10.5299 11.0500 -25.7278

C -12.0009 12.1700 -24.6208

H -12.0549 12.6900 -23.6638

H -12.1069 12.9210 -25.4088

C -13.1449 11.1640 -24.7098

H -13.0169 10.4260 -23.9148

H -13.0909 10.6330 -25.6628

C -14.5149 11.8260 -24.5768

H -14.5589 12.3760 -23.6348

H -14.6499 12.5500 -25.3848

C -15.6479 10.8040 -24.6178

H -15.5129 10.0930 -23.7988

H -15.5929 10.2400 -25.5518

C -17.0259 11.4490 -24.4978

H -17.1689 12.1550 -25.3208

H -17.0799 12.0170 -23.5668

C -18.1449 10.4120 -24.5228

H -18.0829 9.8360 -25.4488

H -18.0039 9.7150 -23.6928

C -19.5239 11.0490 -24.4148

H -19.6889 11.7470 -25.2328

H -20.3029 10.2890 -24.4518

H -19.6209 11.5940 -23.4788

C -11.2889 12.4190 -20.5908

H -11.2309 11.6180 -21.3398

H -11.1509 11.9710 -19.6048

C -12.6479 13.1100 -20.6698

H -12.7309 13.8220 -19.8468

H -12.6989 13.6760 -21.6018

C -13.7719 12.0830 -20.6138

H -13.6379 11.4630 -19.7238

H -13.6879 11.4240 -21.4788

C -15.1629 12.7100 -20.5848

H -15.2569 13.3590 -19.7108

H -15.3039 13.3330 -21.4708

C -16.2439 11.6320 -20.5428

H -16.0669 10.9860 -19.6778

H -16.1589 11.0090 -21.4348

C -17.6589 12.1960 -20.4558

H -17.7589 12.7980 -19.5498

H -17.8439 12.8540 -21.3088

C -18.6969 11.0770 -20.4428

H -18.5009 10.4170 -19.5928

H -18.5879 10.4790 -21.3498

C -20.1309 11.5900 -20.3498

H -20.2589 12.1500 -19.4208

H -20.3259 12.2780 -21.1768

C -21.1389 10.4450 -20.3978

H -20.9519 9.7690 -19.5588

H -20.9899 9.8750 -21.3188

C -22.5839 10.9300 -20.3358

H -22.7379 11.4950 -19.4138

H -22.7749 11.6040 -21.1738

C -23.5759 9.7700 -20.3868

H -23.4009 9.1100 -19.5338

H -23.4019 9.1900 -21.2968

C -25.0219 10.2480 -20.3608

H -25.2259 10.8950 -21.2108

H -25.7049 9.4030 -20.4018

H -25.2229 10.8060 -19.4488

C -10.6819 14.2980 -16.8368

H -10.3649 13.5120 -16.1348

H -10.0659 15.1910 -16.6658

C -12.1499 14.6440 -16.6098

H -12.2429 15.1120 -15.6278

H -12.4519 15.3720 -17.3648

C -13.0589 13.4210 -16.6898

H -12.9419 12.9580 -17.6698

H -12.7509 12.6920 -15.9368

C -14.5219 13.7950 -16.4658

H -14.8149 14.5550 -17.1938

H -14.6329 14.2340 -15.4708

C -15.4529 12.5920 -16.5938

H -15.1459 11.8190 -15.8868

H -15.3629 12.1730 -17.5968

C -16.9109 12.9620 -16.3358

H -17.2219 13.7240 -17.0548

H -17.0049 13.3980 -15.3378

C -17.8339 11.7520 -16.4498

H -17.7119 11.2990 -17.4358

H -17.5429 11.0060 -15.7078

C -19.3009 12.1200 -16.2478

H -19.5959 12.8510 -17.0048

H -19.4259 12.5940 -15.2708

C -20.2109 10.8990 -16.3418

H -19.9379 10.1860 -15.5598

H -20.0549 10.4090 -17.3048

C -21.6869 11.2600 -16.1958

H -21.8509 11.7440 -15.2308

H -21.9609 11.9760 -16.9738

C -22.5869 10.0320 -16.3048

H -22.3289 9.3250 -15.5108

H -22.4039 9.5380 -17.2608

C -24.0629 10.3920 -16.1988

H -24.3399 11.0940 -16.9828

H -24.6829 9.5050 -16.2988

H -24.2759 10.8550 -15.2368

O -10.2539 13.3550 -20.8698

O -7.9339 12.9230 -21.9378

O -10.5309 13.8520 -18.1678

O -4.5669 14.2090 -18.5078

C 2.9161 13.2870 -16.8058

C 2.9321 12.5640 -15.6268

H 1.9981 12.2030 -15.2328

C 4.1331 12.3540 -14.9578

C 5.3331 12.8440 -15.4998

C 5.3111 13.5320 -16.7228

C 4.0941 13.7660 -17.3508

H 4.0511 14.3230 -18.2728

C 3.0581 11.3170 -13.1108

H 2.5301 10.5500 -13.6958

H 2.3981 12.1900 -13.0018

C 3.4001 10.7590 -11.7308

H 3.9751 9.8410 -11.8568

H 2.4511 10.5070 -11.2498

C 4.1831 11.7430 -10.8558

H 3.8461 12.7610 -11.0638

H 3.9751 11.5380 -9.8018

C 5.6891 11.6450 -11.0928

H 5.8891 11.7350 -12.1598

H 6.0291 10.6570 -10.7718

C 6.4861 12.7060 -10.3378

H 6.2541 13.6940 -10.7438

H 6.1951 12.7110 -9.2828

C 7.9851 12.4410 -10.4588

H 8.2291 12.2790 -11.5108

H 8.2291 11.5230 -9.9168

C 8.8431 13.5800 -9.9168

H 8.6421 14.4890 -10.4878

H 8.5711 13.7820 -8.8778

C 10.3311 13.2460 -10.0008

H 10.5831 12.9970 -11.0348

H 10.5371 12.3650 -9.3888

C 11.2151 14.3970 -9.5338

H 11.0381 15.2710 -10.1658

H 10.9391 14.6740 -8.5128

C 12.6971 14.0320 -9.5778

H 12.8741 13.1640 -8.9388

H 12.9681 13.7520 -10.5988

C 13.5901 15.1810 -9.1198

H 13.3021 15.4810 -8.1088

H 13.4321 16.0440 -9.7718

C 15.0641 14.7940 -9.1358

H 15.2441 13.9510 -8.4728

H 15.6841 15.6260 -8.8098

H 15.3711 14.5120 -10.1398

C 7.1931 13.7630 -14.3518

H 6.8401 13.9950 -13.3368

H 6.9861 14.6240 -14.9938

C 8.6871 13.4690 -14.3448

H 8.9781 13.1880 -15.3578

H 8.8691 12.6120 -13.6948

C 9.5291 14.6510 -13.8728

H 9.3371 15.5190 -14.5078

H 9.2471 14.9190 -12.8528

C 11.0161 14.3050 -13.9178

H 11.2851 14.0250 -14.9388

H 11.1981 13.4360 -13.2808

C 11.9161 15.4490 -13.4588

H 11.7521 16.3240 -14.0918

H 11.6571 15.7280 -12.4358

C 13.3881 15.0470 -13.5168

H 13.6421 14.7650 -14.5418

H 13.5421 14.1660 -12.8888

C 14.3261 16.1570 -13.0538

H 14.1961 17.0360 -13.6898

H 14.0701 16.4470 -12.0338

C 15.7851 15.7090 -13.0988

H 16.0381 15.4170 -14.1218

H 15.9101 14.8270 -12.4668

C 16.7481 16.7980 -12.6348

H 16.6381 17.6760 -13.2768

H 16.4921 17.0980 -11.6168

C 18.1991 16.3230 -12.6728

H 18.4371 15.9730 -13.6818

H 18.3191 15.4740 -11.9958

C 19.1821 17.4220 -12.2798

H 19.0451 18.2820 -12.9388

H 18.9681 17.7490 -11.2598

C 20.6261 16.9430 -12.3678

H 20.7871 16.0940 -11.7078

H 21.3121 17.7360 -12.0808

H 20.8631 16.6360 -13.3848

C 6.5161 14.6640 -18.4388

H 6.0191 15.6340 -18.2988

H 5.9781 14.1020 -19.2158

C 7.9561 14.8720 -18.8898

H 7.9331 15.4080 -19.8398

H 8.4111 13.8950 -19.0638

C 8.7991 15.6460 -17.8808

H 8.8701 15.0680 -16.9588

H 8.3101 16.5950 -17.6438

C 10.1951 15.9130 -18.4348

H 10.6271 14.9700 -18.7778

H 10.1171 16.5700 -19.3038

C 11.1361 16.5420 -17.4108

H 10.7111 17.4790 -17.0458

H 11.2401 15.8700 -16.5568

C 12.5131 16.8050 -18.0168

H 12.8891 15.8780 -18.4558

H 12.4181 17.5350 -18.8248

C 13.5231 17.3120 -16.9928

H 13.6181 16.5780 -16.1898

H 13.1611 18.2420 -16.5488

C 14.8951 17.5460 -17.6218

H 15.2191 16.6310 -18.1238

H 14.8191 18.3250 -18.3838

C 15.9441 17.9450 -16.5868

H 15.6441 18.8780 -16.1038

H 15.9931 17.1750 -15.8138

C 17.3281 18.1180 -17.2078

H 17.3001 18.9250 -17.9438

H 17.6041 17.2010 -17.7368

C 18.3911 18.4250 -16.1568

H 18.1281 19.3490 -15.6358

H 18.4041 17.6210 -15.4188

C 19.7791 18.5650 -16.7698

H 20.0651 17.6470 -17.2778

H 20.5181 18.7770 -15.9998

H 19.7971 19.3760 -17.4948

O 6.5061 12.6070 -14.8248

O 4.2431 11.6980 -13.7758

O 6.5101 13.9430 -17.2228

O 0.6191 13.9170 -16.6058

C -1.1589 14.1120 -12.1988

C 0.0971 13.9310 -11.6088

C -2.2929 14.2020 -11.3818

C 0.2151 13.8220 -10.2408

H 0.9701 13.8790 -12.2428

C -2.1729 14.0890 -10.0168

H -3.2609 14.3490 -11.8368

C -0.9199 13.8940 -9.4318

H 1.1831 13.6790 -9.7848

H -3.0409 14.1420 -9.3768

C -2.2969 14.2620 -22.9258

C -1.0329 14.1020 -23.5068

C -3.4179 14.3750 -23.7528

C -0.9009 14.0190 -24.8728

H -0.1669 14.0330 -22.8648

C -3.2849 14.2790 -25.1208

H -4.3889 14.5350 -23.3108

C -2.0269 14.0850 -25.6938

H 0.0671 13.8900 -25.3318

H -4.1489 14.3520 -25.7618

C -5.6229 13.7950 -17.6638

H -5.3469 12.8490 -17.1738

H -5.8129 14.5480 -16.8858

C 1.6231 13.5400 -17.5268

H 1.3051 12.6250 -18.0508

H 1.7761 14.3340 -18.2698

C -2.0889 11.0960 -19.7228

C -2.0089 10.8060 -25.3668

C -1.9899 10.7350 -26.5728

C -2.0829 10.6820 -27.9728

C -0.9279 10.6890 -28.7798

C -3.3519 10.6280 -28.5478

H -4.1879 10.5980 -27.8698

O -3.1339 11.1620 -19.0908

O -0.9069 11.0810 -19.1498

H -0.9919 11.1180 -18.1448

C -2.3899 10.6830 -38.9928

C -2.4299 10.7570 -33.3418

C -2.4599 10.7180 -32.1328

C -2.3729 10.6810 -30.7318

C -3.5269 10.6160 -29.9228

C -1.0999 10.7000 -30.1538

H -0.2559 10.7310 -30.8238

O -1.3399 10.5920 -39.6208

O -3.5639 10.7560 -39.5738

H -3.4959 10.7050 -40.5838

C -7.1509 10.3650 -30.4718

C -7.2169 10.2940 -31.8508

H -6.3039 10.1710 -32.4098

C -8.4509 10.4150 -32.4828

C -9.6199 10.5940 -31.7288

C -9.5449 10.6410 -30.3298

C -8.3059 10.5140 -29.7168

H -8.2239 10.5530 -28.6418

C -7.6289 9.9940 -34.6898

H -6.7209 10.6020 -34.5598

H -7.3799 8.9460 -34.4718

C -8.1299 10.1310 -36.1268

H -8.3409 11.1830 -36.3248

H -7.3149 9.8180 -36.7848

C -9.3829 9.2960 -36.4088

H -9.3179 8.3560 -35.8598

H -9.4149 9.0630 -37.4788

C -10.6839 10.0010 -36.0248

H -10.6309 10.3100 -34.9828

H -10.8059 10.9010 -36.6348

C -11.8849 9.0790 -36.2278

H -11.7609 8.2050 -35.5858

H -11.8999 8.7260 -37.2628

C -13.2129 9.7640 -35.9118

H -13.1999 10.1100 -34.8778

H -13.3309 10.6440 -36.5488

C -14.4029 8.8300 -36.1218

H -14.2849 7.9480 -35.4878

H -14.4099 8.4770 -37.1558

C -15.7439 9.4920 -35.8128

H -15.7599 9.8080 -34.7678

H -15.8599 10.3870 -36.4288

C -16.9109 8.5430 -36.0738

H -16.7799 7.6380 -35.4748

H -16.8989 8.2350 -37.1228

C -18.2649 9.1670 -35.7488

H -18.4079 10.0640 -36.3558

H -18.2809 9.4720 -34.7008

C -19.4139 8.1950 -36.0058

H -19.3929 7.8790 -37.0518

H -19.2699 7.2990 -35.3978

C -20.7709 8.8090 -35.6888

H -20.9429 9.6910 -36.3008

H -21.5699 8.0960 -35.8828

H -20.8209 9.1030 -34.6428

C -11.8449 9.8740 -32.1628

H -11.9019 9.1920 -33.0198

H -11.6329 9.2770 -31.2728

C -13.1559 10.6390 -31.9958

H -13.0869 11.2620 -31.1038

H -13.2779 11.3040 -32.8538

C -14.3469 9.6930 -31.8948

H -14.2199 9.0480 -31.0208

H -14.3679 9.0470 -32.7748

C -15.6789 10.4310 -31.7838

H -15.6749 11.0620 -30.8928

H -15.8019 11.0870 -32.6498

C -16.8529 9.4580 -31.7148

H -16.7449 8.8300 -30.8268

H -16.8279 8.7990 -32.5858

C -18.2039 10.1660 -31.6608

H -18.2349 10.8240 -30.7908

H -18.3209 10.7920 -32.5498

C -19.3609 9.1720 -31.5848

H -19.2589 8.5740 -30.6768

H -19.3079 8.4900 -32.4368

C -20.7229 9.8620 -31.5758

H -20.7729 10.5520 -30.7308

H -20.8349 10.4530 -32.4888

C -21.8689 8.8590 -31.4768

H -21.7729 8.2980 -30.5438

H -21.7979 8.1440 -32.3008

C -23.2369 9.5350 -31.5168

H -23.3059 10.2610 -30.7028

H -23.3409 10.0840 -32.4558

C -24.3779 8.5290 -31.3928

H -24.2849 7.9960 -30.4438

H -24.2969 7.7950 -32.1978

C -25.7429 9.2000 -31.4598

H -25.8659 9.7210 -32.4068

H -26.5379 8.4630 -31.3688

H -25.8549 9.9240 -30.6558

C -10.6759 11.1670 -28.2878

H -10.4369 10.2780 -27.6858

H -9.9069 11.9300 -28.1088

C -12.0439 11.7200 -27.8978

H -11.9819 12.1250 -26.8868

H -12.2849 12.5380 -28.5808

C -13.1369 10.6590 -27.9708

H -13.0959 10.1800 -28.9498

H -12.9499 9.8930 -27.2138

C -14.5259 11.2530 -27.7638

H -14.7209 11.9920 -28.5448

H -14.5609 11.7730 -26.8038

C -15.6139 10.1840 -27.7998

H -15.4529 9.4790 -26.9788

H -15.5389 9.6220 -28.7318

C -17.0119 10.7840 -27.6848

H -17.1839 11.4580 -28.5268

H -17.0749 11.3780 -26.7708

C -18.0979 9.7130 -27.6648

H -18.0189 9.0980 -28.5638

H -17.9399 9.0590 -26.8038

C -19.4969 10.3180 -27.5888

H -19.6749 10.9240 -28.4808

H -19.5569 10.9810 -26.7238

C -20.5809 9.2500 -27.4778

H -20.4229 8.6750 -26.5618

H -20.4969 8.5620 -28.3228

C -21.9839 9.8510 -27.4578

H -22.0739 10.5310 -26.6068

H -22.1359 10.4380 -28.3668

C -23.0689 8.7810 -27.3678

H -22.9309 8.2020 -26.4508

H -22.9669 8.0950 -28.2118

C -24.4669 9.3850 -27.3788

H -24.6269 9.9550 -28.2908

H -25.2239 8.6050 -27.3258

H -24.6019 10.0530 -26.5298

O -10.7889 10.7950 -32.4108

O -8.6479 10.4290 -33.8238

O -10.7159 10.8300 -29.6598

O -4.7279 10.5380 -30.5458

C 2.6471 10.6840 -28.0038

C 2.6241 11.3640 -26.7968

H 1.7141 11.8640 -26.5088

C 3.7631 11.3980 -25.9958

C 4.9321 10.7630 -26.4348

C 4.9491 10.0850 -27.6588

C 3.7951 10.0360 -28.4318

H 3.7871 9.5020 -29.3688

C 2.7251 12.6800 -24.2738

H 1.8551 12.0110 -24.3128

H 2.5011 13.5770 -24.8698

C 2.9891 13.0660 -22.8208

H 3.1811 12.1560 -22.2488

H 2.0701 13.5170 -22.4418

C 4.1621 14.0360 -22.6418

H 4.2641 14.6490 -23.5398

H 3.9471 14.7110 -21.8098

C 5.4861 13.3250 -22.3618

H 5.6791 12.5960 -23.1488

H 5.4031 12.7810 -21.4168

C 6.6441 14.3180 -22.2678

H 6.8171 14.7620 -23.2508

H 6.3671 15.1290 -21.5878

C 7.9371 13.6760 -21.7688

H 8.1961 12.8340 -22.4128

H 7.7781 13.2850 -20.7608

C 9.0931 14.6740 -21.7498

H 9.2931 15.0090 -22.7698

H 8.8021 15.5540 -21.1698

C 10.3711 14.0880 -21.1558

H 10.6711 13.2110 -21.7328

H 10.1761 13.7610 -20.1318

C 11.5111 15.1040 -21.1538

H 11.7011 15.4300 -22.1788

H 11.2101 15.9830 -20.5788

C 12.8001 14.5390 -20.5628

H 12.6161 14.2160 -19.5358

H 13.1071 13.6600 -21.1348

C 13.9311 15.5650 -20.5768

H 13.6321 16.4400 -19.9958

H 14.1051 15.8930 -21.6028

C 15.2221 14.9970 -20.0008

H 15.0721 14.6860 -18.9698

H 16.0141 15.7420 -20.0238

H 15.5481 14.1300 -20.5728

C 6.8831 11.9630 -25.9678

H 6.4111 12.8620 -25.5468

H 6.9641 12.0960 -27.0558

C 8.2621 11.7490 -25.3618

H 8.7001 10.8540 -25.8068

H 8.1401 11.5660 -24.2928

C 9.1851 12.9440 -25.5778

H 9.2711 13.1520 -26.6468

H 8.7511 13.8270 -25.1038

C 10.5731 12.6860 -24.9958

H 11.0171 11.8210 -25.4928

H 10.4761 12.4430 -23.9358

C 11.5051 13.8850 -25.1508

H 11.5911 14.1440 -26.2088

H 11.0751 14.7470 -24.6358

C 12.8931 13.5980 -24.5838

H 13.3371 12.7570 -25.1218

H 12.7981 13.3050 -23.5358

C 13.8241 14.8040 -24.6838

H 13.8971 15.1200 -25.7278

H 13.3981 15.6380 -24.1198

C 15.2191 14.4890 -24.1498

H 15.6541 13.6780 -24.7368

H 15.1391 14.1420 -23.1178

C 16.1461 15.7000 -24.2018

H 16.2021 16.0680 -25.2298

H 15.7311 16.5050 -23.5898

C 17.5511 15.3640 -23.7078

H 17.9531 14.5380 -24.2988

H 17.4981 15.0320 -22.6678

C 18.5021 16.5530 -23.8088

H 18.5381 16.8980 -24.8448

H 18.1201 17.3760 -23.1998

C 19.9081 16.1900 -23.3488

H 19.8971 15.8550 -22.3138

H 20.5731 17.0470 -23.4248

H 20.3131 15.3870 -23.9618

C 6.3341 9.1020 -29.3338

H 6.0151 9.8870 -30.0338

H 5.7481 8.1950 -29.5378

C 7.8231 8.8220 -29.5128

H 7.9951 8.5450 -30.5518

H 8.1031 7.9800 -28.8788

C 8.6681 10.0390 -29.1438

H 8.4251 10.3270 -28.1208

H 8.4071 10.8720 -29.8008

C 10.1661 9.7690 -29.2438

H 10.4281 8.9250 -28.6018

H 10.4221 9.4930 -30.2698

C 10.9721 10.9950 -28.8248

H 10.7161 11.8320 -29.4788

H 10.6881 11.2750 -27.8078

C 12.4791 10.7650 -28.8758

H 12.7441 9.9430 -28.2058

H 12.7681 10.4710 -29.8868

C 13.2501 12.0180 -28.4708

H 12.9371 12.3220 -27.4698

H 12.9971 12.8330 -29.1538

C 14.7611 11.8050 -28.4808

H 15.0161 11.0090 -27.7778

H 15.0751 11.4780 -29.4738

C 15.5181 13.0740 -28.1018

H 15.2901 13.8640 -28.8228

H 15.1761 13.4160 -27.1228

C 17.0281 12.8550 -28.0548

H 17.3791 12.5250 -29.0358

H 17.2521 12.0590 -27.3418

C 17.7811 14.1180 -27.6458

H 17.6121 14.8980 -28.3918

H 17.3851 14.4790 -26.6958

C 19.2781 13.8680 -27.5028

H 19.4671 13.1120 -26.7438

H 19.7931 14.7810 -27.2128

H 19.6991 13.5190 -28.4438

O 6.0851 10.8230 -25.6778

O 3.8621 12.0310 -24.7988

O 6.1361 9.5330 -28.0038

O 0.2591 10.6950 -28.1298

C -2.0199 10.8780 -23.9648

C -0.8239 10.8110 -23.2438

C -3.2329 11.0140 -23.2818

C -0.8389 10.8820 -21.8708

H 0.1051 10.6950 -23.7828

C -3.2439 11.0820 -21.9078

H -4.1529 11.0600 -23.8438

C -2.0489 11.0200 -21.1928

H 0.0801 10.8290 -21.3078

H -4.1659 11.1820 -21.3588

C -2.4199 10.7660 -34.7468

C -1.2309 10.5300 -35.4448

C -3.6029 10.9940 -35.4568

C -1.2299 10.5100 -36.8188

H -0.3199 10.3610 -34.8898

C -3.6009 10.9690 -36.8308

H -4.5129 11.1850 -34.9088

C -2.4149 10.7240 -37.5238

H -0.3239 10.3200 -37.3728

H -4.5119 11.1340 -37.3848

C -5.8509 10.3140 -29.7148

H -5.7449 9.3330 -29.2258

H -5.8949 11.0870 -28.9358

C 1.4381 10.6950 -28.9028

H 1.4591 9.8160 -29.5618

H 1.4691 11.6020 -29.5288

C -2.3429 10.5160 -42.2668

C -2.2569 10.1340 -47.9048

C -2.2339 10.0410 -49.1098

C -2.3039 9.9800 -50.5108

C -1.1349 10.0280 -51.2958

C -3.5599 9.8850 -51.1098

H -4.4109 9.8210 -50.4518

O -3.3929 10.6090 -41.6458

O -1.1659 10.4890 -41.6848

H -1.2549 10.5570 -40.6818

C -2.4549 10.2540 -61.5348

C -2.5399 10.0900 -55.8838

C -2.6029 10.0370 -54.6778

C -2.5419 9.9890 -53.2748

C -3.7069 9.8800 -52.4878

C -1.2809 10.0430 -52.6728

H -0.4279 10.1090 -53.3288

O -1.4059 10.3180 -62.1698

O -3.6349 10.2440 -62.1098

H -3.5819 10.3150 -63.1098

C -7.2879 9.6070 -53.2138

C -7.2449 9.4490 -54.5868

H -6.3009 9.2170 -55.0498

C -8.3999 9.6350 -55.3398

C -9.6069 9.9610 -54.7038

C -9.6529 10.0690 -53.3058

C -8.4859 9.8900 -52.5738

H -8.4939 9.9910 -51.4998

C -7.4179 8.9690 -57.4068

H -6.4599 9.4550 -57.1768

H -7.3489 7.9100 -57.1198

C -7.6909 9.0910 -58.9048

H -7.7579 10.1480 -59.1688

H -6.8239 8.6680 -59.4208

C -8.9689 8.3700 -59.3398

H -9.0799 7.4580 -58.7498

H -8.8769 8.0850 -60.3928

C -10.2249 9.2230 -59.1718

H -10.2519 9.6240 -58.1588

H -10.1879 10.0660 -59.8688

C -11.4829 8.3970 -59.4258

H -11.4989 7.5720 -58.7108

H -11.4409 7.9610 -60.4278

C -12.7659 9.2130 -59.2898

H -12.7679 9.7260 -58.3268

H -12.7949 9.9800 -60.0698

C -14.0059 8.3290 -59.3998

H -13.9869 7.5910 -58.5938

H -13.9709 7.7740 -60.3408

C -15.3149 9.1110 -59.3288

H -15.3689 9.6410 -58.3758

H -15.3379 9.8600 -60.1238

C -16.5219 8.1850 -59.4648

H -16.4779 7.4260 -58.6798

H -16.4669 7.6600 -60.4228

C -17.8529 8.9250 -59.3708

H -17.9119 9.6690 -60.1678

H -17.9049 9.4550 -58.4178

C -19.0379 7.9690 -59.4818

H -18.9839 7.4370 -60.4338

H -18.9709 7.2230 -58.6868

C -20.3759 8.6910 -59.3798

H -20.4689 9.4340 -60.1678

H -21.1999 7.9870 -59.4758

H -20.4649 9.1960 -58.4208

C -11.8019 9.3470 -55.3668

H -11.8079 8.6940 -56.2488

H -11.6889 8.7160 -54.4818

C -13.0969 10.1500 -55.2878

H -13.0769 10.7580 -54.3828

H -13.1379 10.8290 -56.1418

C -14.3159 9.2340 -55.2878

H -14.2549 8.5580 -54.4318

H -14.3009 8.6200 -56.1898

C -15.6359 9.9990 -55.2238

H -15.6679 10.5990 -54.3118

H -15.6979 10.6860 -56.0718

C -16.8289 9.0470 -55.2518

H -16.7689 8.3760 -54.3908

H -16.7729 8.4310 -56.1518

C -18.1709 9.7720 -55.2238

H -18.2379 10.3800 -54.3188

H -18.2379 10.4480 -56.0798

C -19.3379 8.7880 -55.2628

H -19.2829 8.1340 -54.3888

H -19.2469 8.1590 -56.1508

C -20.6949 9.4850 -55.2778

H -20.7969 10.1020 -54.3828

H -20.7509 10.1500 -56.1428

C -21.8449 8.4830 -55.3358

H -21.8109 7.8480 -54.4468

H -21.7179 7.8370 -56.2078

C -23.2079 9.1660 -55.4118

H -23.3329 9.8230 -54.5488

H -23.2489 9.7880 -56.3088

C -24.3529 8.1560 -55.4488

H -24.3299 7.5540 -54.5378

H -24.2109 7.4840 -56.2968

C -25.7109 8.8350 -55.5648

H -25.7649 9.4280 -56.4758

H -26.5089 8.0960 -55.5898

H -25.8839 9.4960 -54.7178

C -10.9299 10.7280 -51.3938

H -10.7469 9.8500 -50.7568

H -10.1699 11.4890 -51.1728

C -12.3189 11.2990 -51.1198

H -12.3209 11.7490 -50.1258

H -12.5129 12.0860 -51.8508

C -13.4129 10.2380 -51.2128

H -13.3299 9.7300 -52.1738

H -13.2629 9.4930 -50.4268

C -14.8089 10.8400 -51.0808

H -14.9659 11.5640 -51.8838

H -14.8859 11.3780 -50.1338

C -15.8969 9.7710 -51.1478

H -15.7809 9.0840 -50.3058

H -15.7769 9.1900 -52.0638

C -17.3009 10.3700 -51.1148

H -17.4209 11.0490 -51.9628

H -17.4229 10.9590 -50.2028

C -18.3829 9.2960 -51.1718

H -18.2369 8.6840 -52.0648

H -18.2889 8.6400 -50.3038

C -19.7869 9.8930 -51.1948

H -19.8889 10.5410 -52.0688

H -19.9319 10.5130 -50.3078

C -20.8669 8.8150 -51.2388

H -20.7769 8.1790 -50.3538

H -20.7119 8.1850 -52.1168

C -22.2729 9.4080 -51.2878

H -22.4369 10.0250 -50.4008

H -22.3579 10.0580 -52.1618

C -23.3489 8.3290 -51.3598

H -23.2689 7.6810 -50.4838

H -23.1769 7.7120 -52.2448

C -24.7499 8.9220 -51.4248

H -24.8499 9.5650 -52.2958

H -25.4979 8.1360 -51.4928

H -24.9559 9.5170 -50.5368

O -10.6949 10.2320 -55.4898

O -8.4679 9.5780 -56.6928

O -10.8649 10.3640 -52.7588

O -4.8879 9.7780 -53.1438

C 2.4421 10.0670 -50.5178

C 2.4471 10.7540 -49.3148

H 1.5481 11.2650 -49.0128

C 3.6041 10.7830 -48.5398

C 4.7601 10.1390 -48.9978

C 4.7501 9.4620 -50.2238

C 3.5781 9.4130 -50.9678

H 3.5481 8.8760 -51.9028

C 2.6451 12.1310 -46.8168

H 1.7411 11.5080 -46.8258

H 2.4571 13.0250 -47.4278

C 2.9581 12.5270 -45.3758

H 3.1561 11.6200 -44.8038

H 2.0571 12.9940 -44.9738

C 4.1461 13.4850 -45.2388

H 4.1931 14.1300 -46.1188

H 3.9881 14.1300 -44.3708

C 5.4801 12.7580 -45.0698

H 5.6161 12.0570 -45.8918

H 5.4541 12.1790 -44.1438

C 6.6501 13.7390 -45.0148

H 6.7441 14.2450 -45.9788

H 6.4391 14.5070 -44.2648

C 7.9721 13.0570 -44.6708

H 8.1801 12.2780 -45.4048

H 7.8821 12.5730 -43.6958

C 9.1331 14.0480 -44.6368

H 9.2641 14.4850 -45.6298

H 8.8921 14.8660 -43.9528

C 10.4431 13.4000 -44.1938

H 10.6941 12.5870 -44.8778

H 10.3101 12.9690 -43.1998

C 11.5941 14.4030 -44.1578

H 11.7241 14.8390 -45.1508

H 11.3431 15.2170 -43.4738

C 12.9081 13.7660 -43.7138

H 12.7791 13.3280 -42.7208

H 13.1671 12.9560 -44.3998

C 14.0521 14.7750 -43.6738

H 13.7981 15.5840 -42.9858

H 14.1811 15.2140 -44.6658

C 15.3611 14.1320 -43.2328

H 15.2531 13.6970 -42.2418

H 16.1601 14.8690 -43.1998

H 15.6511 13.3430 -43.9228

C 6.7911 11.2480 -48.6228

H 6.4511 12.1840 -48.1538

H 6.7751 11.3900 -49.7108

C 8.1981 10.9000 -48.1578

H 8.5381 10.0180 -48.7028

H 8.1491 10.6340 -47.1008

C 9.1821 12.0490 -48.3528

H 9.1951 12.3480 -49.4048

H 8.8511 12.9130 -47.7718

C 10.5881 11.6510 -47.9148

H 10.9441 10.8270 -48.5378

H 10.5501 11.2880 -46.8848

C 11.5791 12.8080 -47.9928

H 11.5931 13.2090 -49.0098

H 11.2501 13.6100 -47.3288

C 12.9861 12.3690 -47.5978

H 13.3331 11.5970 -48.2878

H 12.9561 11.9270 -46.5988

C 13.9791 13.5280 -47.5968

H 13.9811 14.0050 -48.5798

H 13.6591 14.2770 -46.8698

C 15.3921 13.0610 -47.2578

H 15.7191 12.3340 -48.0048

H 15.3811 12.5540 -46.2898

C 16.3891 14.2150 -47.2108

H 16.3561 14.7570 -48.1598

H 16.1001 14.9160 -46.4248

C 17.8121 13.7270 -46.9578

H 18.0821 12.9990 -47.7268

H 17.8541 13.2190 -45.9918

C 18.8291 14.8650 -46.9718

H 18.7771 15.3820 -47.9338

H 18.5751 15.5880 -46.1938

C 20.2481 14.3550 -46.7508

H 20.3251 13.8460 -45.7918

H 20.9581 15.1790 -46.7598

H 20.5261 13.6540 -47.5338

C 6.0741 8.3940 -51.9028

H 5.7381 9.1280 -52.6488

H 5.4731 7.4800 -52.0158

C 7.5551 8.0870 -52.1018

H 7.6911 7.6400 -53.0858

H 7.8621 7.3610 -51.3478

C 8.4091 9.3450 -51.9698

H 8.1481 9.8420 -51.0358

H 8.1791 10.0280 -52.7908

C 9.9031 9.0370 -51.9668

H 10.1261 8.3450 -51.1518

H 10.1801 8.5420 -52.9008

C 10.7321 10.3060 -51.7918

H 10.5491 10.9740 -52.6358

H 10.4071 10.8220 -50.8868

C 12.2271 10.0180 -51.6918

H 12.4121 9.3710 -50.8318

H 12.5551 9.4790 -52.5828

C 13.0461 11.2980 -51.5448

H 12.6991 11.8500 -50.6688

H 12.8811 11.9330 -52.4188

C 14.5371 11.0110 -51.4008

H 14.6991 10.4120 -50.5008

H 14.8771 10.4190 -52.2528

C 15.3711 12.2860 -51.3098

H 15.2451 12.8690 -52.2258

H 15.0111 12.8950 -50.4788

C 16.8521 11.9800 -51.1068

H 17.2101 11.3640 -51.9348

H 16.9731 11.4010 -50.1878

C 17.7051 13.2420 -51.0128

H 17.5941 13.8230 -51.9318

H 17.3451 13.8590 -50.1868

C 19.1771 12.9140 -50.7948

H 19.3101 12.3430 -49.8778

H 19.7671 13.8240 -50.7188

H 19.5621 12.3220 -51.6228

O 5.9201 10.1870 -48.2528

O 3.7391 11.4170 -47.3468

O 5.9321 8.9200 -50.6008

O 0.0441 10.0650 -50.6308

C -2.2719 10.2300 -46.5038

C -1.0719 10.3180 -45.7928

C -3.4889 10.2410 -45.8138

C -1.0859 10.4060 -44.4198

H -0.1399 10.3130 -46.3378

C -3.4989 10.3330 -44.4418

H -4.4129 10.1780 -46.3688

C -2.2999 10.4140 -43.7348

H -0.1649 10.4730 -43.8638

H -4.4229 10.3420 -43.8848

C -2.5139 10.1320 -57.2878

C -1.3089 9.9830 -57.9838

C -3.7009 10.3200 -58.0028

C -1.2959 10.0250 -59.3578

H -0.3929 9.8430 -57.4288

C -3.6899 10.3390 -59.3758

H -4.6219 10.4550 -57.4558

C -2.4859 10.1970 -60.0668

H -0.3749 9.9150 -59.9078

H -4.6069 10.4680 -59.9278

C -6.0419 9.5010 -52.3728

H -5.9539 8.4900 -51.9458

H -6.1289 10.2200 -51.5488

C 1.2251 10.0810 -51.4038

H 1.2491 9.2090 -52.0708

H 1.2481 10.9950 -52.0198

C -1.7929 12.9600 -54.8818

C -2.2559 13.3770 -60.5108

C -2.4109 13.3840 -61.7108

C -2.6509 13.3840 -63.0968

C -1.5889 13.2780 -64.0188

C -3.9689 13.4410 -63.5578

H -4.7529 13.4820 -62.8178

O -2.7889 12.9520 -54.1688

O -0.5719 12.8540 -54.3998

H -0.5679 12.7830 -53.4008

C -2.8259 13.1470 -74.0698

C -3.3549 13.4860 -68.4358

C -3.3799 13.4640 -67.2258

C -3.2029 13.4210 -65.8308

C -4.2709 13.4260 -64.9108

C -1.8879 13.3310 -65.3698

H -1.1119 13.2860 -66.1168

O -1.7299 13.0900 -74.6108

O -3.9579 13.0980 -74.7308

H -3.8089 13.0350 -75.7238

C -7.8269 12.8110 -65.4048

C -7.7209 12.4740 -66.7428

H -6.7399 12.3120 -67.1558

C -8.8669 12.3770 -67.5198

C -10.1319 12.5810 -66.9448

C -10.2299 12.8750 -65.5768

C -9.0699 12.9910 -64.8188

H -9.1239 13.2370 -63.7708

C -7.6959 11.8240 -69.5298

H -6.9029 12.5290 -69.2518

H -7.3659 10.8100 -69.2568

C -7.9479 11.9110 -71.0348

H -8.1799 12.9450 -71.2938

H -7.0159 11.6370 -71.5358

C -9.0909 11.0050 -71.4998

H -9.0989 10.1030 -70.8888

H -8.9179 10.7100 -72.5388

C -10.4579 11.6780 -71.3898

H -10.5699 12.0930 -70.3888

H -10.5149 12.5020 -72.1068

C -11.5959 10.6950 -71.6468

H -11.5399 9.8870 -70.9128

H -11.4879 10.2520 -72.6418

C -12.9589 11.3720 -71.5348

H -13.0129 11.8920 -70.5768

H -13.0649 12.1230 -72.3228

C -14.1029 10.3660 -71.6238

H -13.9749 9.6290 -70.8288

H -14.0489 9.8350 -72.5768

C -15.4729 11.0280 -71.4908

H -15.5169 11.5780 -70.5488

H -15.6079 11.7520 -72.2988

C -16.6059 10.0060 -71.5318

H -16.4709 9.2950 -70.7128

H -16.5509 9.4420 -72.4648

C -17.9839 10.6520 -71.4118

H -18.1269 11.3570 -72.2348

H -18.0379 11.2200 -70.4808

C -19.1029 9.6140 -71.4368

H -19.0409 9.0390 -72.3628

H -18.9619 8.9170 -70.6068

C -20.4819 10.2510 -71.3278

H -20.6469 10.9490 -72.1468

H -21.2609 9.4920 -71.3658

H -20.5789 10.7960 -70.3928

C -12.2469 11.6220 -67.5048

H -12.1889 10.8210 -68.2538

H -12.1089 11.1730 -66.5188

C -13.6059 12.3130 -67.5838

H -13.6889 13.0250 -66.7608

H -13.6569 12.8780 -68.5158

C -14.7299 11.2850 -67.5268

H -14.5949 10.6650 -66.6378

H -14.6459 10.6260 -68.3928

C -16.1209 11.9120 -67.4988

H -16.2149 12.5610 -66.6248

H -16.2619 12.5360 -68.3848

C -17.2019 10.8340 -67.4558

H -17.0249 10.1880 -66.5918

H -17.1169 10.2110 -68.3488

C -18.6169 11.3980 -67.3698

H -18.7169 12.0010 -66.4638

H -18.8019 12.0560 -68.2228

C -19.6549 10.2790 -67.3568

H -19.4589 9.6200 -66.5068

H -19.5459 9.6810 -68.2638

C -21.0889 10.7930 -67.2638

H -21.2169 11.3520 -66.3348

H -21.2839 11.4800 -68.0908

C -22.0969 9.6470 -67.3118

H -21.9099 8.9710 -66.4728

H -21.9479 9.0770 -68.2318

C -23.5419 10.1320 -67.2488

H -23.6959 10.6980 -66.3278

H -23.7329 10.8060 -68.0878

C -24.5339 8.9720 -67.3008

H -24.3589 8.3120 -66.4478

H -24.3599 8.3930 -68.2108

C -25.9789 9.4500 -67.2738

H -26.1839 10.0970 -68.1248

H -26.6629 8.6050 -67.3158

H -26.1809 10.0080 -66.3628

C -11.6399 13.5000 -63.7508

H -11.3229 12.7150 -63.0488

H -11.0229 14.3930 -63.5798

C -13.1079 13.8460 -63.5238

H -13.2009 14.3140 -62.5408

H -13.4099 14.5740 -64.2788

C -14.0169 12.6230 -63.6038

H -13.8999 12.1600 -64.5838

H -13.7089 11.8940 -62.8508

C -15.4799 12.9970 -63.3798

H -15.7729 13.7580 -64.1078

H -15.5909 13.4370 -62.3848

C -16.4109 11.7940 -63.5078

H -16.1039 11.0210 -62.8008

H -16.3209 11.3750 -64.5108

C -17.8689 12.1640 -63.2498

H -18.1799 12.9270 -63.9678

H -17.9619 12.6000 -62.2518

C -18.7919 10.9540 -63.3638

H -18.6699 10.5010 -64.3488

H -18.5009 10.2080 -62.6218

C -20.2579 11.3220 -63.1618

H -20.5539 12.0530 -63.9188

H -20.3839 11.7960 -62.1848

C -21.1689 10.1010 -63.2548

H -20.8959 9.3880 -62.4738

H -21.0129 9.6110 -64.2188

C -22.6449 10.4620 -63.1098

H -22.8089 10.9470 -62.1438

H -22.9189 11.1790 -63.8878

C -23.5449 9.2340 -63.2178

H -23.2869 8.5280 -62.4248

H -23.3619 8.7410 -64.1748

C -25.0209 9.5950 -63.1128

H -25.2979 10.2960 -63.8968

H -25.6409 8.7070 -63.2128

H -25.2339 10.0570 -62.1508

O -11.2119 12.5580 -67.7838

O -8.8919 12.1250 -68.8518

O -11.4889 13.0540 -65.0818

O -5.5249 13.4110 -65.4218

C 1.9581 12.4890 -63.7198

C 1.9741 11.7660 -62.5398

H 1.0401 11.4050 -62.1468

C 3.1751 11.5560 -61.8718

C 4.3751 12.0460 -62.4128

C 4.3531 12.7340 -63.6358

C 3.1361 12.9690 -64.2648

H 3.0931 13.5250 -65.1868

C 2.1001 10.5190 -60.0248

H 1.5721 9.7520 -60.6098

H 1.4401 11.3920 -59.9158

C 2.4421 9.9610 -58.6448

H 3.0171 9.0430 -58.7708

H 1.4931 9.7090 -58.1638

C 3.2251 10.9460 -57.7698

H 2.8881 11.9640 -57.9778

H 3.0171 10.7400 -56.7158

C 4.7311 10.8480 -58.0068

H 4.9311 10.9370 -59.0738

H 5.0711 9.8600 -57.6858

C 5.5281 11.9080 -57.2518

H 5.2961 12.8960 -57.6578

H 5.2371 11.9130 -56.1968

C 7.0271 11.6430 -57.3728

H 7.2711 11.4810 -58.4238

H 7.2711 10.7260 -56.8308

C 7.8851 12.7820 -56.8308

H 7.6841 13.6910 -57.4018

H 7.6131 12.9840 -55.7918

C 9.3731 12.4480 -56.9148

H 9.6251 12.1990 -57.9488

H 9.5791 11.5670 -56.3028

C 10.2571 13.6000 -56.4478

H 10.0801 14.4730 -57.0788

H 9.9811 13.8770 -55.4268

C 11.7391 13.2340 -56.4918

H 11.9161 12.3660 -55.8528

H 12.0101 12.9540 -57.5128

C 12.6321 14.3840 -56.0338

H 12.3441 14.6830 -55.0228

H 12.4751 15.2460 -56.6858

C 14.1061 13.9970 -56.0498

H 14.2861 13.1530 -55.3868

H 14.7261 14.8280 -55.7238

H 14.4131 13.7140 -57.0538

C 6.2351 12.9650 -61.2658

H 5.8821 13.1970 -60.2508

H 6.0281 13.8270 -61.9078

C 7.7291 12.6710 -61.2588

H 8.0201 12.3900 -62.2718

H 7.9111 11.8140 -60.6088

C 8.5711 13.8540 -60.7868

H 8.3791 14.7220 -61.4218

H 8.2891 14.1210 -59.7668

C 10.0581 13.5080 -60.8318

H 10.3271 13.2270 -61.8528

H 10.2401 12.6380 -60.1948

C 10.9581 14.6510 -60.3728

H 10.7941 15.5260 -61.0058

H 10.6991 14.9300 -59.3498

C 12.4301 14.2490 -60.4308

H 12.6841 13.9680 -61.4558

H 12.5841 13.3680 -59.8028

C 13.3681 15.3590 -59.9678

H 13.2381 16.2380 -60.6038

H 13.1121 15.6490 -58.9468

C 14.8271 14.9120 -60.0128

H 15.0801 14.6200 -61.0358

H 14.9521 14.0290 -59.3808

C 15.7901 16.0000 -59.5488

H 15.6801 16.8780 -60.1908

H 15.5341 16.3010 -58.5308

C 17.2411 15.5250 -59.5868

H 17.4791 15.1760 -60.5948

H 17.3611 14.6760 -58.9088

C 18.2241 16.6240 -59.1938

H 18.0871 17.4840 -59.8528

H 18.0101 16.9510 -58.1738

C 19.6681 16.1460 -59.2818

H 19.8291 15.2960 -58.6218

H 20.3541 16.9390 -58.9948

H 19.9051 15.8380 -60.2988

C 5.5581 13.8660 -65.3528

H 5.0611 14.8360 -65.2128

H 5.0201 13.3040 -66.1298

C 6.9981 14.0740 -65.8038

H 6.9751 14.6110 -66.7538

H 7.4531 13.0980 -65.9778

C 7.8411 14.8490 -64.7948

H 7.9121 14.2710 -63.8728

H 7.3521 15.7970 -64.5578

C 9.2371 15.1150 -65.3488

H 9.6691 14.1720 -65.6918

H 9.1591 15.7720 -66.2178

C 10.1781 15.7440 -64.3248

H 9.7531 16.6810 -63.9598

H 10.2821 15.0720 -63.4708

C 11.5551 16.0070 -64.9308

H 11.9311 15.0800 -65.3698

H 11.4601 16.7370 -65.7388

C 12.5651 16.5140 -63.9068

H 12.6601 15.7800 -63.1028

H 12.2031 17.4440 -63.4628

C 13.9371 16.7480 -64.5348

H 14.2621 15.8330 -65.0368

H 13.8611 17.5280 -65.2968

C 14.9861 17.1470 -63.5008

H 14.6861 18.0800 -63.0178

H 15.0351 16.3780 -62.7268

C 16.3701 17.3200 -64.1218

H 16.3421 18.1270 -64.8578

H 16.6461 16.4040 -64.6508

C 17.4331 17.6270 -63.0708

H 17.1701 18.5510 -62.5498

H 17.4461 16.8230 -62.3328

C 18.8211 17.7680 -63.6838

H 19.1071 16.8490 -64.1918

H 19.5601 17.9790 -62.9138

H 18.8391 18.5780 -64.4088

O 5.5481 11.8090 -61.7388

O 3.2851 10.9000 -60.6898

O 5.5521 13.1450 -64.1368

O -0.3389 13.1200 -63.5188

C -2.1169 13.3150 -59.1128

C -0.8609 13.1330 -58.5228

C -3.2509 13.4040 -58.2958

C -0.7429 13.0240 -57.1548

H 0.0121 13.0810 -59.1568

C -3.1309 13.2910 -56.9308

H -4.2189 13.5520 -58.7508

C -1.8779 13.0960 -56.3458

H 0.2251 12.8810 -56.6988

H -3.9989 13.3440 -56.2908

C -3.2549 13.4640 -69.8398

C -1.9909 13.3040 -70.4198

C -4.3759 13.5780 -70.6668

C -1.8589 13.2210 -71.7868

H -1.1249 13.2360 -69.7788

C -4.2429 13.4810 -72.0348

H -5.3469 13.7380 -70.2248

C -2.9849 13.2880 -72.6068

H -0.8909 13.0920 -72.2458

H -5.1069 13.5540 -72.6748

C -6.5809 12.9980 -64.5778

H -6.3049 12.0510 -64.0878

H -6.7709 13.7500 -63.7998

C 0.6651 12.7420 -64.4408

H 0.3471 11.8270 -64.9648

H 0.8181 13.5360 -65.1838

C -1.6759 11.3510 3.1522

C -1.6639 10.9740 -2.4868

C -1.6559 10.8830 -3.6918

C -1.7449 10.8220 -5.0918

C -0.5859 10.8700 -5.8928

C -3.0089 10.7280 -5.6748

H -3.8509 10.6640 -5.0058

O -2.7179 11.4440 3.7872

O -0.4919 11.3240 3.7192

H -0.5679 11.3910 4.7232

C -2.0399 11.1060 -16.1128

C -2.0509 10.9370 -10.4618

C -2.0979 10.8830 -9.2538

C -2.0189 10.8330 -7.8528

C -3.1739 10.7250 -7.0508

C -0.7509 10.8870 -7.2668

H 0.0941 10.9530 -7.9338

O -0.9989 11.1700 -16.7618

O -3.2269 11.0960 -16.6718

H -3.1869 11.1680 -17.6728

C -6.7639 10.4530 -7.7298

C -6.7389 10.2960 -9.1038

H -5.8009 10.0640 -9.5788

C -7.9039 10.4830 -9.8408

C -9.1019 10.8090 -9.1888

C -9.1299 10.9150 -7.7908

C -7.9529 10.7360 -7.0738

H -7.9469 10.8360 -5.9998

C -6.9479 9.8190 -11.9208

H -5.9879 10.3040 -11.7028

H -6.8759 8.7600 -11.6358

C -7.2409 9.9410 -13.4148

H -7.3119 10.9990 -13.6778

H -6.3819 9.5190 -13.9428

C -8.5249 9.2210 -13.8338

H -8.6279 8.3090 -13.2438

H -8.4459 8.9370 -14.8878

C -9.7789 10.0750 -13.6488

H -9.7919 10.4750 -12.6358

H -9.7509 10.9180 -14.3458

C -11.0399 9.2500 -13.8878

H -11.0469 8.4240 -13.1728

H -11.0109 8.8140 -14.8908

C -12.3209 10.0660 -13.7338

H -12.3099 10.5770 -12.7708

H -12.3599 10.8330 -14.5128

C -13.5619 9.1820 -13.8288

H -13.5339 8.4430 -13.0238

H -13.5399 8.6270 -14.7698

C -14.8699 9.9640 -13.7398

H -14.9119 10.4930 -12.7858

H -14.9039 10.7130 -14.5338

C -16.0789 9.0390 -13.8608

H -16.0249 8.2790 -13.0768

H -16.0369 8.5150 -14.8198

C -17.4089 9.7790 -13.7488

H -17.4779 10.5230 -14.5448

H -17.4479 10.3080 -12.7948

C -18.5959 8.8230 -13.8448

H -18.5539 8.2920 -14.7988

H -18.5179 8.0760 -13.0518

C -19.9309 9.5450 -13.7248

H -20.0339 10.2890 -14.5108

H -20.7559 8.8410 -13.8108

H -20.0069 10.0490 -12.7648

C -11.3049 10.1960 -9.8238

H -11.3229 9.5440 -10.7058

H -11.1809 9.5640 -8.9408

C -12.5999 10.9990 -9.7268

H -12.5669 11.6070 -8.8218

H -12.6519 11.6780 -10.5798

C -13.8179 10.0840 -9.7118

H -13.7459 9.4060 -8.8568

H -13.8159 9.4700 -10.6148

C -15.1369 10.8490 -9.6308

H -15.1569 11.4480 -8.7178

H -15.2109 11.5360 -10.4768

C -16.3299 9.8970 -9.6438

H -16.2599 9.2260 -8.7838

H -16.2869 9.2810 -10.5448

C -17.6719 10.6220 -9.5968

H -17.7279 11.2300 -8.6918

H -17.7499 11.2990 -10.4518

C -18.8399 9.6380 -9.6218

H -18.7729 8.9840 -8.7488

H -18.7609 9.0100 -10.5118

C -20.1969 10.3360 -9.6188

H -20.2869 10.9520 -8.7218

H -20.2639 11.0020 -10.4828

C -21.3479 9.3340 -9.6628

H -21.3019 8.6980 -8.7748

H -21.2319 8.6890 -10.5368

C -22.7109 10.0170 -9.7208

H -22.8249 10.6740 -8.8548

H -22.7639 10.6410 -10.6158

C -23.8569 9.0080 -9.7428

H -23.8219 8.4050 -8.8318

H -23.7259 8.3370 -10.5938

C -25.2169 9.6880 -9.8408

H -25.2819 10.2810 -10.7508

H -26.0139 8.9480 -9.8558

H -25.3779 10.3480 -8.9908

C -10.3819 11.5730 -5.8618

H -10.1899 10.6950 -5.2278

H -9.6179 12.3340 -5.6498

C -11.7669 12.1440 -5.5688

H -11.7549 12.5940 -4.5738

H -11.9699 12.9320 -6.2968

C -12.8619 11.0840 -5.6488

H -12.7919 10.5770 -6.6108

H -12.7019 10.3380 -4.8658

C -14.2559 11.6860 -5.4968

H -14.4229 12.4100 -6.2978

H -14.3209 12.2240 -4.5488

C -15.3449 10.6170 -5.5508

H -15.2179 9.9290 -4.7108

H -15.2369 10.0370 -6.4698

C -16.7479 11.2170 -5.4988

H -16.8789 11.8960 -6.3448

H -16.8579 11.8050 -4.5858

C -17.8319 10.1430 -5.5438

H -17.6979 9.5320 -6.4388

H -17.7259 9.4860 -4.6768

C -19.2359 10.7400 -5.5478

H -19.3479 11.3890 -6.4198

H -19.3689 11.3590 -4.6578

C -20.3159 9.6630 -5.5778

H -20.2149 9.0250 -4.6958

H -20.1719 9.0340 -6.4588

C -21.7219 10.2560 -5.6078

H -21.8749 10.8730 -4.7188

H -21.8189 10.9070 -6.4808

C -22.7999 9.1770 -5.6678

H -22.7089 8.5280 -4.7928

H -22.6399 8.5610 -6.5548

C -24.2009 9.7710 -5.7138

H -24.3129 10.4150 -6.5828

H -24.9509 8.9850 -5.7718

H -24.3949 10.3650 -4.8228

O -10.1999 11.0810 -9.9598

O -7.9889 10.4270 -11.1928

O -10.3349 11.2110 -7.2268

O -4.3629 10.6230 -7.6908

C 3.0011 10.9080 -5.1608

C 3.0221 11.5940 -3.9568

H 2.1271 12.1060 -3.6428

C 4.1881 11.6220 -3.1978

C 5.3381 10.9780 -3.6708

C 5.3121 10.3030 -4.8978

C 4.1311 10.2540 -5.6258

H 4.0881 9.7180 -6.5608

C 3.2521 12.9690 -1.4608

H 2.3481 12.3460 -1.4588

H 3.0561 13.8640 -2.0688

C 3.5841 13.3640 -0.0238

H 3.7901 12.4560 0.5442

H 2.6891 13.8300 0.3912

C 4.7751 14.3210 0.0982

H 4.8101 14.9670 -0.7808

H 4.6281 14.9650 0.9692

C 6.1101 13.5940 0.2492

H 6.2361 12.8930 -0.5748

H 6.0971 13.0140 1.1752

C 7.2811 14.5740 0.2902

H 7.3631 15.0810 -0.6748

H 7.0801 15.3420 1.0432

C 8.6071 13.8920 0.6162

H 8.8061 13.1140 -0.1208

H 8.5301 13.4070 1.5922

C 9.7691 14.8830 0.6362

H 9.8871 15.3200 -0.3588

H 9.5371 15.7000 1.3232

C 11.0851 14.2340 1.0612

H 11.3261 13.4220 0.3732

H 10.9641 13.8020 2.0562

C 12.2361 15.2370 1.0832

H 12.3531 15.6730 0.0882

H 11.9941 16.0500 1.7712

C 13.5561 14.5980 1.5092

H 13.4391 14.1600 2.5032

H 13.8061 13.7900 0.8192

C 14.7011 15.6070 1.5352

H 14.4551 16.4160 2.2272

H 14.8161 16.0480 0.5422

C 16.0151 14.9640 1.9582

H 15.9201 14.5280 2.9502

H 16.8151 15.7010 1.9812

H 16.2961 14.1750 1.2642

C 7.3741 12.0870 -3.3218

H 7.0411 13.0220 -2.8468

H 7.3441 12.2290 -4.4098

C 8.7881 11.7370 -2.8758

H 9.1201 10.8560 -3.4258

H 8.7521 11.4710 -1.8178

C 9.7681 12.8870 -3.0828

H 9.7691 13.1860 -4.1338

H 9.4461 13.7500 -2.4958

C 11.1811 12.4880 -2.6628

H 11.5281 11.6640 -3.2908

H 11.1551 12.1240 -1.6328

C 12.1711 13.6450 -2.7528

H 12.1721 14.0470 -3.7698

H 11.8501 14.4470 -2.0838

C 13.5831 13.2050 -2.3768

H 13.9201 12.4330 -3.0718

H 13.5651 12.7620 -1.3778

C 14.5761 14.3630 -2.3878

H 14.5651 14.8420 -3.3708

H 14.2661 15.1120 -1.6568

C 15.9931 13.8960 -2.0678

H 16.3101 13.1700 -2.8198

H 15.9941 13.3890 -1.0998

C 16.9901 15.0500 -2.0328

H 16.9461 15.5930 -2.9808

H 16.7121 15.7500 -1.2418

C 18.4171 14.5610 -1.7988

H 18.6771 13.8340 -2.5718

H 18.4711 14.0530 -0.8328

C 19.4341 15.6990 -1.8258

H 19.3691 16.2170 -2.7858

H 19.1901 16.4210 -1.0428

C 20.8551 15.1890 -1.6228

H 20.9451 14.6790 -0.6658

H 21.5651 16.0120 -1.6408

H 21.1231 14.4880 -2.4098

C 6.6141 9.2350 -6.5948

H 6.2681 9.9700 -7.3348

H 6.0111 8.3220 -6.6998

C 8.0921 8.9280 -6.8128

H 8.2151 8.4820 -7.7988

H 8.4081 8.2010 -6.0638

C 8.9481 10.1860 -6.6918

H 8.6991 10.6820 -5.7528

H 8.7081 10.8700 -7.5078

C 10.4421 9.8770 -6.7078

H 10.6761 9.1850 -5.8958

H 10.7061 9.3830 -7.6458

C 11.2741 11.1460 -6.5428

H 11.0801 11.8150 -7.3838

H 10.9601 11.6620 -5.6338

C 12.7691 10.8580 -6.4618

H 12.9661 10.2100 -5.6048

H 13.0861 10.3190 -7.3578

C 13.5911 12.1370 -6.3248

H 13.2551 12.6880 -5.4448

H 13.4151 12.7730 -7.1958

C 15.0841 11.8500 -6.2008

H 15.2571 11.2500 -5.3038

H 15.4121 11.2580 -7.0578

C 15.9191 13.1250 -6.1198

H 15.7811 13.7090 -7.0328

H 15.5701 13.7330 -5.2828

C 17.4021 12.8180 -5.9358

H 17.7501 12.2030 -6.7688

H 17.5351 12.2380 -5.0198

C 18.2561 14.0800 -5.8518

H 18.1341 14.6610 -6.7688

H 17.9081 14.6960 -5.0208

C 19.7311 13.7510 -5.6538

H 19.8761 13.1790 -4.7398

H 20.3231 14.6610 -5.5848

H 20.1061 13.1600 -6.4868

O 6.5081 11.0260 -2.9408

O 4.3401 12.2550 -2.0058

O 6.4891 9.7600 -5.2898

O 0.6011 10.9070 -5.2428

C -1.6609 11.0690 -1.0858

C -0.4509 11.1560 -0.3898

C -2.8689 11.0800 -0.3788

C -0.4479 11.2430 0.9832

H 0.4731 11.1520 -0.9468

C -2.8599 11.1700 0.9922

H -3.7989 11.0170 -0.9218

C -1.6529 11.2510 1.6842

H 0.4811 11.3090 1.5272

H -3.7769 11.1790 1.5612

C -2.0429 10.9800 -11.8658

C -0.8479 10.8310 -12.5768

C -3.2389 11.1690 -12.5638

C -0.8529 10.8740 -13.9518

H 0.0751 10.6900 -12.0348

C -3.2459 11.1890 -13.9378

H -4.1529 11.3040 -12.0058

C -2.0509 11.0480 -14.6448

H 0.0611 10.7640 -14.5128

H -4.1709 11.3190 -14.4778

C -5.5069 10.3460 -6.9058

H -5.4139 9.3340 -6.4808

H -5.5829 11.0650 -6.0798

C 1.7721 10.9230 -6.0298

H 1.7871 10.0510 -6.6988

H 1.7871 11.8370 -6.6458

C -3.1309 7.1100 -54.6338

C -3.4399 7.4900 -60.2768

C -3.5629 7.4890 -61.4808

C -3.7649 7.4810 -62.8718

C -2.6789 7.3630 -63.7638

C -5.0689 7.5430 -63.3688

H -5.8729 7.5940 -62.6508

O -4.1459 7.1120 -53.9478

O -1.9249 7.0000 -54.1188

H -1.9469 6.9360 -53.1198

C -3.6449 7.1690 -73.8438

C -4.3239 7.5510 -68.2288

C -4.3819 7.5370 -67.0198

C -4.2429 7.5030 -65.6198

C -5.3349 7.5210 -64.7298

C -2.9409 7.4090 -65.1228

H -2.1459 7.3540 -65.8478

O -2.5359 7.1020 -74.3548

O -4.7599 7.1220 -74.5348

H -4.5839 7.0510 -75.5228

C -8.8799 6.9230 -65.3148

C -8.7399 6.5760 -66.6468

H -7.7499 6.4050 -67.0318

C -9.8659 6.4810 -67.4548

C -11.1439 6.6960 -66.9148

C -11.2779 7.0000 -65.5528

C -10.1369 7.1140 -64.7648

H -10.2179 7.3690 -63.7198

C -8.6439 5.9060 -69.4278

H -7.8539 6.6090 -69.1338

H -8.3279 4.8920 -69.1388

C -8.8539 5.9850 -70.9398

H -9.0729 7.0180 -71.2128

H -7.9119 5.7020 -71.4138

C -9.9909 5.0830 -71.4288

H -10.0199 4.1840 -70.8128

H -9.7919 4.7790 -72.4608

C -11.3559 5.7640 -71.3608

H -11.4919 6.1860 -70.3658

H -11.3889 6.5830 -72.0848

C -12.4929 4.7860 -71.6418

H -12.4609 3.9830 -70.9018

H -12.3609 4.3360 -72.6308

C -13.8539 5.4720 -71.5708

H -13.9299 5.9990 -70.6188

H -13.9339 6.2180 -72.3678

C -15.0009 4.4730 -71.6848

H -14.8989 3.7400 -70.8818

H -14.9249 3.9340 -72.6318

C -16.3699 5.1430 -71.5928

H -16.4359 5.7000 -70.6568

H -16.4779 5.8630 -72.4088

C -17.5069 4.1280 -71.6578

H -17.3989 3.4220 -70.8298

H -17.4309 3.5570 -72.5858

C -18.8839 4.7820 -71.5798

H -19.0009 5.4830 -72.4108

H -18.9599 5.3570 -70.6538

C -20.0089 3.7510 -71.6278

H -19.9249 3.1690 -72.5468

H -19.8939 3.0590 -70.7888

C -21.3869 4.3970 -71.5608

H -21.5249 5.0910 -72.3878

H -22.1679 3.6420 -71.6138

H -21.5049 4.9490 -70.6318

C -13.2489 5.7450 -67.5258

H -13.1759 4.9390 -68.2678

H -13.1399 5.3030 -66.5328

C -14.6009 6.4440 -67.6458

H -14.7019 7.1620 -66.8298

H -14.6239 7.0030 -68.5828

C -15.7319 5.4240 -67.6128

H -15.6259 4.8090 -66.7158

H -15.6299 4.7580 -68.4708

C -17.1199 6.0590 -67.6258

H -17.2329 6.7150 -66.7598

H -17.2329 6.6770 -68.5198

C -18.2079 4.9880 -67.6058

H -18.0589 4.3470 -66.7318

H -18.1029 4.3580 -68.4908

C -19.6219 5.5610 -67.5608

H -19.7429 6.1700 -66.6618

H -19.7799 6.2140 -68.4228

C -20.6669 4.4480 -67.5688

H -20.4969 3.7930 -66.7088

H -20.5369 3.8430 -68.4678

C -22.0989 4.9710 -67.5178

H -22.2479 5.5370 -66.5958

H -22.2679 5.6540 -68.3538

C -23.1119 3.8310 -67.5848

H -22.9519 3.1600 -66.7368

H -22.9419 3.2540 -68.4978

C -24.5559 4.3240 -67.5648

H -24.7309 4.8970 -66.6518

H -24.7199 4.9940 -68.4128

C -25.5529 3.1700 -67.6358

H -25.4049 2.5150 -66.7728

H -25.3579 2.5830 -68.5358

C -26.9959 3.6570 -67.6508

H -27.1729 4.2990 -68.5108

H -27.6839 2.8160 -67.7048

H -27.2189 4.2230 -66.7498

C -12.7319 7.6460 -63.7698

H -12.4389 6.8640 -63.0538

H -12.1149 8.5370 -63.5878

C -14.2039 8.0030 -63.5838

H -14.3199 8.4780 -62.6078

H -14.4799 8.7270 -64.3518

C -15.1179 6.7850 -63.6808

H -14.9769 6.3140 -64.6538

H -14.8339 6.0590 -62.9138

C -16.5839 7.1690 -63.4988

H -16.8529 7.9260 -64.2398

H -16.7189 7.6160 -62.5098

C -17.5179 5.9710 -63.6438

H -17.2349 5.2010 -62.9228

H -17.4039 5.5440 -64.6408

C -18.9799 6.3510 -63.4278

H -19.2679 7.1100 -64.1598

H -19.0979 6.7940 -62.4358

C -19.9069 5.1450 -63.5588

H -19.7609 4.6850 -64.5368

H -19.6409 4.4030 -62.8028

C -21.3759 5.5240 -63.3988

H -21.6469 6.2510 -64.1678

H -21.5249 6.0050 -62.4288

C -22.2919 4.3080 -63.5078

H -22.0439 3.5980 -62.7148

H -22.1129 3.8100 -64.4638

C -23.7679 4.6780 -63.4048

H -23.9559 5.1700 -62.4478

H -24.0169 5.3910 -64.1948

C -24.6729 3.4550 -63.5298

H -24.4399 2.7520 -62.7248

H -24.4669 2.9530 -64.4768

C -26.1489 3.8250 -63.4658

H -26.3999 4.5220 -64.2618

H -26.7719 2.9400 -63.5768

H -26.3849 4.2960 -62.5138

O -12.2009 6.6730 -67.7828

O -9.8559 6.2200 -68.7838

O -12.5479 7.1900 -65.0928

O -6.5749 7.5100 -65.2738

C 0.8541 6.5550 -63.3638

C 0.8331 5.8410 -62.1798

H -0.1129 5.4880 -61.8088

C 2.0141 5.6280 -61.4778

C 3.2321 6.1070 -61.9898

C 3.2471 6.7860 -63.2178

C 2.0491 7.0240 -63.8808

H 2.0341 7.5740 -64.8068

C 0.8841 4.6100 -59.6528

H 0.3671 3.8420 -60.2478

H 0.2271 5.4880 -59.5678

C 1.1851 4.0600 -58.2608

H 1.7581 3.1380 -58.3648

H 0.2221 3.8160 -57.8038

C 1.9511 5.0460 -57.3718

H 1.6251 6.0640 -57.5958

H 1.7131 4.8490 -56.3218

C 3.4611 4.9370 -57.5678

H 3.6911 5.0180 -58.6298

H 3.7871 3.9490 -57.2308

C 4.2441 5.9980 -56.7978

H 4.0301 6.9840 -57.2168

H 3.9251 6.0120 -55.7518

C 5.7441 5.7230 -56.8768

H 6.0161 5.5530 -57.9198

H 5.9681 4.8080 -56.3218

C 6.5951 6.8610 -56.3198

H 6.4141 7.7670 -56.9028

H 6.2961 7.0720 -55.2898

C 8.0821 6.5170 -56.3618

H 8.3601 6.2600 -57.3858

H 8.2661 5.6390 -55.7378

C 8.9611 7.6670 -55.8778

H 8.8051 8.5370 -56.5208

H 8.6591 7.9530 -54.8668

C 10.4411 7.2920 -55.8798

H 10.5951 6.4280 -55.2298

H 10.7371 7.0040 -56.8908

C 11.3281 8.4400 -55.4058

H 11.0151 8.7480 -54.4048

H 11.1931 9.2990 -56.0668

C 12.7991 8.0440 -55.3788

H 12.9561 7.2040 -54.7058

H 13.4161 8.8740 -55.0418

H 13.1321 7.7520 -56.3728

C 5.0661 7.0230 -60.7988

H 4.6871 7.2640 -59.7948

H 4.8811 7.8810 -61.4518

C 6.5581 6.7200 -60.7488

H 6.8741 6.4310 -61.7528

H 6.7161 5.8660 -60.0888

C 7.3931 7.9010 -60.2628

H 7.2241 8.7660 -60.9088

H 7.0851 8.1770 -59.2528

C 8.8781 7.5460 -60.2648

H 9.1741 7.2570 -61.2768

H 9.0381 6.6800 -59.6178

C 9.7731 8.6870 -59.7898

H 9.6321 9.5590 -60.4338

H 9.4891 8.9750 -58.7758

C 11.2431 8.2760 -59.8058

H 11.5231 7.9860 -60.8218

H 11.3761 7.3980 -59.1678

C 12.1761 9.3840 -59.3248

H 12.0681 10.2590 -59.9698

H 11.8941 9.6820 -58.3138

C 13.6331 8.9270 -59.3278

H 13.9111 8.6270 -60.3408

H 13.7351 8.0480 -58.6858

C 14.5901 10.0130 -58.8458

H 14.5021 10.8870 -59.4958

H 14.3081 10.3220 -57.8358

C 16.0381 9.5290 -58.8408

H 16.3011 9.1710 -59.8398

H 16.1341 8.6840 -58.1548

C 17.0161 10.6250 -58.4288

H 16.9021 11.4820 -59.0968

H 16.7771 10.9600 -57.4168

C 18.4591 10.1370 -58.4748

H 18.5981 9.2910 -57.8048

H 19.1431 10.9280 -58.1738

H 18.7221 9.8220 -59.4818

C 4.5051 7.8990 -64.9088

H 4.0101 8.8740 -64.7888

H 3.9851 7.3350 -65.6958

C 5.9581 8.0960 -65.3218

H 5.9631 8.6260 -66.2758

H 6.4111 7.1150 -65.4768

C 6.7781 8.8720 -64.2958

H 6.8201 8.3000 -63.3678

H 6.2891 9.8250 -64.0788

C 8.1901 9.1260 -64.8138

H 8.6261 8.1780 -65.1378

H 8.1391 9.7780 -65.6888

C 9.1071 9.7570 -63.7698

H 8.6771 10.6990 -63.4218

H 9.1831 9.0900 -62.9078

C 10.5011 10.0080 -64.3398

H 10.8831 9.0760 -64.7618

H 10.4321 10.7330 -65.1538

C 11.4871 10.5160 -63.2918

H 11.5551 9.7870 -62.4808

H 11.1191 11.4510 -62.8638

C 12.8761 10.7370 -63.8848

H 13.2081 9.8170 -64.3718

H 12.8261 11.5120 -64.6538

C 13.9001 11.1370 -62.8258

H 13.5921 12.0750 -62.3568

H 13.9231 10.3730 -62.0448

C 15.3011 11.2980 -63.4098

H 15.2971 12.1000 -64.1518

H 15.5851 10.3760 -63.9238

C 16.3371 11.6060 -62.3318

H 16.0661 12.5350 -61.8248

H 16.3251 10.8070 -61.5888

C 17.7411 11.7340 -62.9088

H 18.0361 10.8100 -63.4028

H 18.4611 11.9460 -62.1208

H 17.7841 12.5390 -63.6388

O 4.3851 5.8680 -61.2818

O 2.0881 4.9790 -60.2888

O 4.4621 7.1870 -63.6878

O -1.4439 7.2010 -63.2298

C -3.3389 7.4370 -58.8738

C -2.1009 7.2520 -58.2498

C -4.4939 7.5390 -58.0888

C -2.0199 7.1520 -56.8788

H -1.2109 7.1910 -58.8588

C -4.4109 7.4350 -56.7208

H -5.4479 7.6890 -58.5718

C -3.1759 7.2360 -56.1008

H -1.0659 7.0070 -56.3948

H -5.2959 7.4970 -56.1048

C -4.1869 7.5180 -69.6288

C -2.9079 7.3470 -70.1738

C -5.2839 7.6330 -70.4868

C -2.7399 7.2540 -71.5368

H -2.0599 7.2780 -69.5088

C -5.1139 7.5260 -71.8498

H -6.2659 7.8020 -70.0718

C -3.8429 7.3210 -72.3868

H -1.7609 7.1160 -71.9678

H -5.9609 7.6000 -72.5138

C -7.6559 7.1080 -64.4558

H -7.3989 6.1630 -63.9518

H -7.8619 7.8670 -63.6878

C -0.4179 6.8100 -64.1218

H -0.7269 5.8940 -64.6478

H -0.2399 7.5990 -64.8658

C -1.1139 4.8750 2.2252

C -0.9659 4.4500 -3.4188

C -1.0049 4.4550 -4.6278

C -1.0689 4.4880 -6.0318

C 0.1121 4.6130 -6.7928

C -2.3109 4.4490 -6.6668

H -3.1869 4.3810 -6.0418

O -2.1589 4.7080 2.8402

O 0.0111 5.2220 2.8062

H -0.0909 5.2800 3.8052

C -1.8899 4.7310 -17.0378

C -1.3119 4.4200 -11.4198

C -1.2919 4.4730 -10.2118

C -1.2299 4.5160 -8.8088

C -2.4169 4.5210 -8.0468

C 0.0121 4.5800 -8.1728

H 0.8921 4.6070 -8.7968

O -0.9509 4.9640 -17.7908

O -3.1319 4.6210 -17.4548

H -3.2209 4.7460 -18.4468

C -5.8559 5.4940 -8.8258

C -5.8529 5.4250 -10.2068

H -4.9889 5.0170 -10.7028

C -6.9659 5.8670 -10.9208

C -8.0889 6.3490 -10.2358

C -8.0609 6.4660 -8.8388

C -6.9349 6.0460 -8.1458

H -6.8889 6.1230 -7.0718

C -6.0579 5.3080 -13.0648

H -5.1039 5.8170 -12.8648

H -5.9399 4.2410 -12.8278

C -6.4169 5.4820 -14.5398

H -6.6059 6.5390 -14.7278

H -5.5409 5.1810 -15.1178

C -7.6329 4.6650 -14.9878

H -7.6089 3.6870 -14.5008

H -7.5669 4.4960 -16.0658

C -8.9579 5.3580 -14.6788

H -8.9769 5.6480 -13.6288

H -9.0249 6.2720 -15.2728

C -10.1609 4.4690 -14.9878

H -10.1179 3.5730 -14.3618

H -10.1139 4.1410 -16.0298

C -11.4829 5.1920 -14.7428

H -11.4929 5.5860 -13.7258

H -11.5589 6.0450 -15.4208

C -12.6919 4.2790 -14.9418

H -12.6539 3.4650 -14.2138

H -12.6439 3.8280 -15.9368

C -14.0119 5.0320 -14.7938

H -14.0429 5.5230 -13.8198

H -14.0659 5.8140 -15.5538

C -15.2239 4.1130 -14.9368

H -15.2359 3.3950 -14.1118

H -15.1349 3.5390 -15.8628

C -16.5349 4.8950 -14.9538

H -16.5219 5.5920 -15.7938

H -16.6179 5.4830 -14.0378

C -17.7539 3.9850 -15.0818

H -17.6399 3.3510 -15.9658

H -17.8079 3.3230 -14.2138

C -19.0469 4.7820 -15.1968

H -19.0199 5.4200 -16.0778

H -19.9029 4.1170 -15.2828

H -19.1859 5.4120 -14.3218

C -10.2659 5.7150 -10.8078

H -10.1829 4.9900 -11.6328

H -10.1589 5.1640 -9.8668

C -11.6219 6.4060 -10.8478

H -11.6939 7.0810 -9.9948

H -11.6759 7.0110 -11.7548

C -12.7709 5.4020 -10.8248

H -12.6959 4.7830 -9.9268

H -12.6899 4.7370 -11.6868

C -14.1279 6.1000 -10.8488

H -14.2299 6.7280 -9.9628

H -14.1769 6.7560 -11.7208

C -15.2879 5.1090 -10.9058

H -15.2469 4.4510 -10.0348

H -15.1849 4.4820 -11.7948

C -16.6389 5.8180 -10.9448

H -16.7559 6.4250 -10.0448

H -16.6609 6.4970 -11.8008

C -17.8029 4.8380 -11.0528

H -17.7869 4.1620 -10.1938

H -17.6789 4.2290 -11.9508

C -19.1509 5.5510 -11.1088

H -19.2799 6.1510 -10.2068

H -19.1629 6.2340 -11.9618

C -20.3139 4.5720 -11.2358

H -20.3089 3.8940 -10.3788

H -20.1819 3.9660 -12.1358

C -21.6609 5.2880 -11.3028

H -21.7799 5.9120 -10.4138

H -21.6749 5.9470 -12.1748

C -22.8299 4.3120 -11.3918

H -22.8209 3.6580 -10.5168

H -22.7069 3.6820 -12.2768

C -24.1709 5.0330 -11.4668

H -24.2079 5.6820 -12.3398

H -24.9869 4.3180 -11.5378

H -24.3269 5.6430 -10.5798

C -9.2439 7.0890 -6.8598

H -9.0169 6.1280 -6.3768

H -8.5099 7.8360 -6.5258

C -10.6589 7.5290 -6.5028

H -10.6879 7.8070 -5.4488

H -10.8979 8.4120 -7.0948

C -11.6819 6.4290 -6.7848

H -11.5099 6.0390 -7.7888

H -11.5379 5.6080 -6.0778

C -13.1119 6.9480 -6.6838

H -13.2589 7.7270 -7.4358

H -13.2639 7.4070 -5.7058

C -14.1549 5.8530 -6.8928

H -14.0659 5.1070 -6.0988

H -13.9659 5.3460 -7.8418

C -15.5689 6.4270 -6.8968

H -15.6619 7.1330 -7.7258

H -15.7319 6.9840 -5.9728

C -16.6469 5.3560 -7.0338

H -16.4839 4.7880 -7.9518

H -16.5739 4.6570 -6.1978

C -18.0439 5.9710 -7.0598

H -18.1289 6.6260 -7.9308

H -18.1809 6.5890 -6.1698

C -19.1479 4.9200 -7.1158

H -19.0809 4.2770 -6.2338

H -19.0039 4.2900 -7.9958

C -20.5349 5.5560 -7.1718

H -20.6809 6.1820 -6.2888

H -20.5959 6.2020 -8.0508

C -21.6489 4.5150 -7.2428

H -21.5999 3.8720 -6.3608

H -21.4969 3.8840 -8.1208

C -23.0259 5.1630 -7.3208

H -23.1009 5.7920 -8.2048

H -23.8049 4.4060 -7.3758

H -23.2089 5.7810 -6.4448

O -9.2279 6.6800 -10.9358

O -7.0859 5.8630 -12.2748

O -9.1859 6.9580 -8.2658

O -3.5889 4.6170 -8.7308

C 3.4601 5.5640 -5.7538

C 3.1221 6.3210 -4.6428

H 2.1141 6.6920 -4.5568

C 4.0701 6.5610 -3.6548

C 5.3761 6.0660 -3.8028

C 5.7211 5.3480 -4.9558

C 4.7511 5.0890 -5.9178

H 4.9871 4.5020 -6.7918

C 2.5771 7.7900 -2.2478

H 1.7861 7.0690 -2.4888

H 2.4351 8.6840 -2.8728

C 2.4871 8.1540 -0.7668

H 2.5421 7.2390 -0.1748

H 1.5061 8.6040 -0.6078

C 3.5791 9.1210 -0.3068

H 3.8161 9.8050 -1.1208

H 3.2021 9.7120 0.5332

C 4.8611 8.4110 0.1232

H 5.1801 7.7400 -0.6728

H 4.6611 7.8080 1.0132

C 5.9761 9.4100 0.4172

H 6.1681 9.9960 -0.4838

H 5.6551 10.1000 1.2032

C 7.2671 8.7180 0.8452

H 7.5151 7.9540 0.1082

H 7.1221 8.2180 1.8072

C 8.4201 9.7110 0.9422

H 8.5031 10.2260 -0.0168

H 8.1941 10.4690 1.6972

C 9.7561 9.0480 1.2692

H 9.9251 8.2210 0.5772

H 9.7241 8.6330 2.2792

C 10.9111 10.0390 1.1562

H 10.9501 10.4160 0.1312

H 10.7241 10.8950 1.8092

C 12.2611 9.4200 1.5092

H 12.2461 9.0890 2.5502

H 12.4291 8.5410 0.8842

C 13.4061 10.4080 1.3072

H 13.2251 11.3000 1.9122

H 13.4261 10.7200 0.2602

C 14.7561 9.8110 1.6802

H 14.7651 9.5160 2.7272

H 15.5541 10.5330 1.5212

H 14.9641 8.9320 1.0742

C 7.3281 7.1690 -3.0408

H 7.0771 8.1340 -2.5808

H 7.4321 7.3260 -4.1168

C 8.6401 6.6380 -2.4728

H 8.9011 5.7130 -2.9918

H 8.5031 6.3970 -1.4178

C 9.7401 7.6810 -2.6428

H 9.7661 7.9970 -3.6888

H 9.4871 8.5570 -2.0448

C 11.1261 7.1800 -2.2438

H 11.3831 6.2990 -2.8378

H 11.1231 6.8760 -1.1948

C 12.1771 8.2670 -2.4598

H 12.1221 8.6140 -3.4958

H 11.9431 9.1190 -1.8178

C 13.6011 7.7990 -2.1708

H 13.8471 6.9550 -2.8208

H 13.6691 7.4480 -1.1388

C 14.6091 8.9250 -2.3918

H 14.5051 9.3040 -3.4128

H 14.3781 9.7490 -1.7138

C 16.0521 8.4840 -2.1698

H 16.3061 7.6950 -2.8818

H 16.1541 8.0660 -1.1648

C 17.0261 9.6490 -2.3308

H 16.9101 10.0790 -3.3298

H 16.7741 10.4280 -1.6078

C 18.4801 9.2290 -2.1348

H 18.7391 8.4660 -2.8728

H 18.5961 8.7830 -1.1438

C 19.4381 10.4090 -2.2698

H 19.3271 10.8510 -3.2638

H 19.1721 11.1730 -1.5358

C 20.8901 9.9970 -2.0688

H 21.0301 9.5640 -1.0808

H 21.5501 10.8570 -2.1618

H 21.1841 9.2580 -2.8108

C 7.3871 4.0880 -6.1098

H 7.2351 4.6010 -7.0708

H 6.7761 3.1750 -6.0978

C 8.8611 3.7370 -5.9428

H 9.1311 3.0030 -6.7048

H 9.0041 3.2830 -4.9618

C 9.7431 4.9750 -6.0718

H 9.4711 5.6800 -5.2868

H 9.5471 5.4520 -7.0348

C 11.2301 4.6480 -5.9698

H 11.4271 4.1230 -5.0328

H 11.5101 3.9790 -6.7878

C 12.0811 5.9130 -6.0338

H 11.8161 6.4760 -6.9318

H 11.8521 6.5440 -5.1738

C 13.5761 5.6120 -6.0548

H 13.8441 5.0350 -5.1658

H 13.8101 4.9970 -6.9278

C 14.4081 6.8900 -6.0988

H 14.1811 7.4970 -5.2208

H 14.1261 7.4730 -6.9788

C 15.9071 6.6070 -6.1378

H 16.1911 6.0460 -5.2438

H 16.1381 5.9800 -7.0038

C 16.7261 7.8920 -6.2098

H 16.4771 8.4280 -7.1298

H 16.4551 8.5370 -5.3718

C 18.2281 7.6260 -6.1718

H 18.5091 6.9950 -7.0178

H 18.4731 7.0790 -5.2578

C 19.0371 8.9200 -6.2128

H 18.8131 9.4560 -7.1378

H 18.7321 9.5570 -5.3798

C 20.5351 8.6630 -6.1268

H 20.7791 8.1310 -5.2098

H 21.0871 9.6000 -6.1358

H 20.8701 8.0580 -6.9678

O 6.2701 6.2500 -2.7798

O 3.8471 7.2320 -2.5028

O 7.0151 4.9330 -5.0408

O 1.2661 4.7470 -6.0968

C -0.9709 4.4930 -2.0128

C 0.2141 4.6910 -1.2958

C -2.1819 4.3640 -1.3218

C 0.1811 4.8050 0.0772

H 1.1491 4.7600 -1.8318

C -2.2089 4.4680 0.0492

H -3.0919 4.1930 -1.8778

C -1.0319 4.7100 0.7592

H 1.0891 4.9690 0.6352

H -3.1339 4.3800 0.5972

C -1.4159 4.4200 -12.8218

C -0.3319 4.7770 -13.6318

C -2.6359 4.0820 -13.4188

C -0.4829 4.8520 -14.9978

H 0.6191 5.0030 -13.1738

C -2.7839 4.1560 -14.7838

H -3.4549 3.7650 -12.7918

C -1.7179 4.5690 -15.5838

H 0.3421 5.1450 -15.6288

H -3.7199 3.8920 -15.2488

C -4.7329 4.9580 -7.9748

H -5.0999 4.0680 -7.4378

H -4.4719 5.7230 -7.2338

C 2.4031 5.2440 -6.7768

H 2.7841 4.5000 -7.4888

H 2.1231 6.1530 -7.3308

3504

acid_LT 0.000000

C -2.8169 19.5713 -31.2562

C -2.9639 19.6483 -36.9042

C -2.9949 19.6643 -38.1122

C -3.0519 19.6623 -39.5152

C -1.8599 19.6023 -40.2692

C -4.2939 19.7333 -40.1532

H -5.1689 19.8243 -39.5302

O -3.8609 19.5563 -30.6082

O -1.6329 19.5363 -30.6962

H -1.6869 19.5223 -29.6852

C -3.0929 18.7793 -50.5002

C -3.2789 19.1313 -44.8622

C -3.2679 19.3283 -43.6702

C -3.2109 19.5043 -42.2782

C -4.3949 19.6843 -41.5332

C -1.9669 19.5063 -41.6472

H -1.0949 19.4143 -42.2752

O -2.0319 18.8473 -51.1042

O -4.2549 18.6853 -51.1102

H -4.1369 18.6933 -52.1102

C -7.8039 20.4903 -42.5862

C -7.5429 20.8203 -43.9062

H -6.5169 20.9403 -44.2122

C -8.5929 20.9493 -44.8112

C -9.9149 20.7723 -44.3792

C -10.1729 20.5173 -43.0252

C -9.1109 20.3543 -42.1452

H -9.2899 20.1093 -41.1112

C -7.1719 21.2033 -46.7102

H -6.5949 22.0863 -46.3952

H -6.6339 20.3083 -46.3742

C -7.2889 21.1843 -48.2352

H -7.7339 22.1203 -48.5732

H -6.2719 21.1253 -48.6302

C -8.1209 20.0133 -48.7692

H -7.9819 19.1443 -48.1232

H -7.7619 19.7443 -49.7662

C -9.6079 20.3553 -48.8482

H -9.9399 20.7113 -47.8742

H -9.7479 21.1693 -49.5642

C -10.4649 19.1643 -49.2712

H -10.3579 18.3643 -48.5342

H -10.1129 18.7723 -50.2302

C -11.9369 19.5573 -49.3812

H -12.2299 20.0833 -48.4722

H -12.0609 20.2563 -50.2122

C -12.8649 18.3613 -49.5822

H -12.7219 17.6533 -48.7612

H -12.6079 17.8423 -50.5082

C -14.3279 18.7933 -49.6252

H -14.5679 19.3343 -48.7082

H -14.4719 19.4863 -50.4582

C -15.2899 17.6183 -49.7782

H -15.1379 16.9163 -48.9542

H -15.0739 17.0853 -50.7062

C -16.7439 18.0823 -49.7852

H -16.8929 18.7813 -50.6132

H -16.9529 18.6223 -48.8592

C -17.7329 16.9283 -49.9252

H -17.5359 16.3953 -50.8572

H -17.5829 16.2233 -49.1042

C -19.1749 17.4203 -49.9162

H -19.3449 18.1133 -50.7382

H -19.8669 16.5883 -50.0232

H -19.3959 17.9353 -48.9842

C -11.7309 19.6963 -45.4482

H -11.4559 19.2133 -46.3952

H -11.5309 18.9903 -44.6372

C -13.2099 20.0633 -45.4572

H -13.4449 20.5683 -44.5202

H -13.3919 20.7673 -46.2712

C -14.0919 18.8293 -45.6292

H -13.8939 18.1263 -44.8162

H -13.8339 18.3263 -46.5642

C -15.5759 19.1853 -45.6382

H -15.8309 19.6963 -44.7072

H -15.7729 19.8813 -46.4572

C -16.4659 17.9563 -45.8022

H -16.2589 17.2483 -44.9952

H -16.2239 17.4563 -46.7422

C -17.9489 18.3173 -45.7862

H -18.1859 18.8263 -44.8492

H -18.1609 19.0153 -46.5992

C -18.8439 17.0903 -45.9342

H -18.6219 16.3803 -45.1332

H -18.6249 16.5923 -46.8812

C -20.3249 17.4563 -45.8862

H -20.5439 17.9453 -44.9342

H -20.5469 18.1743 -46.6802

C -21.2279 16.2363 -46.0462

H -20.9889 15.5053 -45.2692

H -21.0299 15.7643 -47.0122

C -22.7069 16.6043 -45.9542

H -22.8979 17.0873 -44.9942

H -22.9489 17.3253 -46.7392

C -23.6169 15.3873 -46.0942

H -23.3639 14.6553 -45.3232

H -23.4429 14.9153 -47.0642

C -25.0879 15.7653 -45.9712

H -25.3629 16.4813 -46.7422

H -25.7209 14.8873 -46.0762

H -25.2849 16.2153 -45.0002

C -11.7979 20.3563 -41.2922

H -11.4999 19.3843 -40.8732

H -11.2629 21.1533 -40.7572

C -13.2989 20.5633 -41.1402

H -13.5059 20.7963 -40.0952

H -13.5819 21.4303 -41.7392

C -14.1299 19.3563 -41.5662

H -13.9039 19.1073 -42.6022

H -13.8669 18.4943 -40.9482

C -15.6189 19.6543 -41.4162

H -15.8859 20.4833 -42.0762

H -15.8159 19.9823 -40.3922

C -16.5099 18.4563 -41.7322

H -16.2739 17.6363 -41.0502

H -16.3129 18.1103 -42.7472

C -17.9859 18.8203 -41.5962

H -18.2269 19.6133 -42.3092

H -18.1669 19.2223 -40.5962

C -18.9149 17.6333 -41.8342

H -18.7349 17.2243 -42.8302

H -18.6909 16.8483 -41.1092

C -20.3809 18.0363 -41.7082

H -20.6109 18.7953 -42.4602

H -20.5479 18.4933 -40.7292

C -21.3319 16.8543 -41.8802

H -21.0959 16.0893 -41.1372

H -21.1839 16.4133 -42.8682

C -22.7909 17.2743 -41.7252

H -22.9409 17.7053 -40.7322

H -23.0239 18.0533 -42.4562

C -23.7549 16.1073 -41.9202

H -23.5449 15.3363 -41.1752

H -23.5929 15.6663 -42.9052

C -25.2079 16.5513 -41.7962

H -25.4349 17.3203 -42.5322

H -25.8819 15.7123 -41.9552

H -25.3979 16.9623 -40.8072

O -10.9279 20.8643 -45.3032

O -8.4579 21.2343 -46.1332

O -11.4829 20.4153 -42.6672

O -5.5419 19.7903 -42.2472

C 1.7351 19.7473 -39.4982

C 1.7391 19.2383 -38.2112

H 0.7981 18.9653 -37.7622

C 2.9501 19.0723 -37.5502

C 4.1641 19.3973 -38.1782

C 4.1471 19.9643 -39.4622

C 2.9231 20.1493 -40.0952

H 2.8821 20.5743 -41.0852

C 2.0041 18.4563 -35.4312

H 1.1511 17.9583 -35.9082

H 1.6911 19.4723 -35.1402

C 2.4051 17.6563 -34.1922

H 2.5951 16.6233 -34.4882

H 1.5431 17.6683 -33.5232

C 3.6391 18.2043 -33.4692

H 3.6181 19.2953 -33.5012

H 3.6011 17.9063 -32.4172

C 4.9441 17.7023 -34.0872

H 4.9371 17.9123 -35.1562

H 5.0071 16.6183 -33.9592

C 6.1721 18.3563 -33.4602

H 6.0991 19.4393 -33.5912

H 6.1911 18.1653 -32.3822

C 7.4711 17.8623 -34.0962

H 7.3781 17.9243 -35.1812

H 7.6291 16.8123 -33.8362

C 8.6681 18.6903 -33.6392

H 8.5071 19.7313 -33.9352

H 8.7271 18.6593 -32.5492

C 10.0041 18.2203 -34.2082

H 9.9631 18.2343 -35.2992

H 10.1931 17.1913 -33.8942

C 11.1411 19.1193 -33.7262

H 10.9471 20.1443 -34.0522

H 11.1551 19.1213 -32.6332

C 12.5101 18.6823 -34.2362

H 12.7021 17.6523 -33.9272

H 12.5161 18.7063 -35.3282

C 13.6221 19.5843 -33.7042

H 13.6281 19.5403 -32.6122

H 13.4131 20.6173 -33.9912

C 14.9921 19.1793 -34.2302

H 15.2301 18.1593 -33.9352

H 15.7641 19.8383 -33.8382

H 15.0151 19.2343 -35.3162

C 6.3381 20.0033 -37.3452

H 6.4371 20.1773 -36.2662

H 6.0881 20.9503 -37.8242

C 7.6651 19.4733 -37.8832

H 7.6081 19.3973 -38.9682

H 7.8371 18.4713 -37.4842

C 8.7911 20.4133 -37.4642

H 8.5571 21.4163 -37.8282

H 8.8221 20.4573 -36.3732

C 10.1651 19.9923 -37.9772

H 10.1701 19.9993 -39.0682

H 10.3831 18.9703 -37.6542

C 11.2451 20.9333 -37.4482

H 10.9971 21.9573 -37.7372

H 11.2441 20.8903 -36.3562

C 12.6461 20.6043 -37.9552

H 12.6721 20.6853 -39.0432

H 12.8981 19.5723 -37.6962

C 13.6791 21.5483 -37.3442

H 13.4061 22.5803 -37.5802

H 13.6551 21.4423 -36.2572

C 15.0991 21.2853 -37.8372

H 15.1431 21.4323 -38.9182

H 15.3711 20.2463 -37.6362

C 16.1021 22.2133 -37.1552

H 15.8081 23.2513 -37.3342

H 16.0681 22.0413 -36.0762

C 17.5301 22.0053 -37.6522

H 17.5611 22.1593 -38.7332

H 17.8371 20.9753 -37.4572

C 18.5101 22.9643 -36.9792

H 18.1731 23.9913 -37.1382

H 18.5121 22.7803 -35.9022

C 19.9251 22.8123 -37.5212

H 20.2911 21.8013 -37.3572

H 20.6001 23.5073 -37.0262

H 19.9471 23.0153 -38.5892

C 5.3901 20.7233 -41.3582

H 5.0231 21.7583 -41.4002

H 4.7531 20.0943 -41.9942

C 6.8251 20.6393 -41.8722

H 6.8051 20.8183 -42.9492

H 7.1861 19.6223 -41.7072

C 7.7741 21.6323 -41.2062

H 7.7881 21.4543 -40.1322

H 7.4091 22.6473 -41.3612

C 9.1851 21.5063 -41.7782

H 9.5371 20.4793 -41.6502

H 9.1571 21.7083 -42.8512

C 10.1731 22.4593 -41.1112

H 9.8151 23.4853 -41.2172

H 10.2211 22.2333 -40.0442

C 11.5741 22.3483 -41.7092

H 11.9231 21.3163 -41.6182

H 11.5361 22.5863 -42.7742

C 12.5701 23.2753 -41.0152

H 12.5731 23.0583 -39.9452

H 12.2481 24.3123 -41.1412

C 13.9881 23.1183 -41.5572

H 14.3061 22.0793 -41.4372

H 14.0001 23.3393 -42.6272

C 14.9751 24.0333 -40.8362

H 14.6761 25.0743 -40.9832

H 14.9361 23.8273 -39.7632

C 16.4091 23.8483 -41.3252

H 16.4551 24.0373 -42.4012

H 16.7171 22.8113 -41.1652

C 17.3811 24.7823 -40.6082

H 17.0381 25.8123 -40.7242

H 17.3811 24.5503 -39.5412

C 18.7991 24.6633 -41.1512

H 19.1631 23.6423 -41.0492

H 19.4731 25.3243 -40.6122

H 18.8281 24.9283 -42.2062

O 5.2951 19.0483 -37.4982

O 3.1021 18.5353 -36.3062

O 5.3511 20.2573 -40.0242

O -0.6919 19.6403 -39.5802

C -2.9219 19.6443 -35.5002

C -1.6939 19.5333 -34.8362

C -4.0989 19.7573 -34.7562

C -1.6489 19.5223 -33.4632

H -0.7909 19.4573 -35.4212

C -4.0529 19.7383 -33.3822

H -5.0429 19.8653 -35.2702

C -2.8289 19.6133 -32.7252

H -0.7069 19.4373 -32.9432

H -4.9579 19.8243 -32.8012

C -3.2509 18.9733 -46.2582

C -2.0299 19.0753 -46.9332

C -4.4219 18.7353 -46.9832

C -1.9969 18.9983 -48.3052

H -1.1269 19.2253 -46.3622

C -4.3859 18.6653 -48.3572

H -5.3489 18.5993 -46.4472

C -3.1729 18.8133 -49.0302

H -1.0719 19.0943 -48.8502

H -5.2889 18.4963 -48.9232

C -6.6879 20.2893 -41.5932

H -7.0229 19.5863 -40.8172

H -6.4459 21.2493 -41.1092

C 0.4911 19.7633 -40.3462

H 0.5661 18.9123 -41.0412

H 0.4481 20.6903 -40.9342

C -2.3179 19.5333 -8.5662

C -3.0879 19.9363 -14.1512

C -3.1369 19.9983 -15.3582

C -3.1879 20.0563 -16.7602

C -1.9799 20.0833 -17.4922

C -4.4219 20.0903 -17.4162

H -5.3069 20.0883 -16.8002

O -3.2559 19.5133 -7.7712

O -1.0659 19.4203 -8.1922

H -0.9619 19.3273 -7.1962

C -2.9569 19.5973 -27.7692

C -3.1739 19.9033 -22.1292

C -3.2969 19.9983 -20.9302

C -3.2979 20.0663 -19.5282

C -4.5039 20.1173 -18.7992

C -2.0649 20.0723 -18.8742

H -1.1819 20.0533 -19.4922

O -1.9059 19.5493 -28.3912

O -4.1349 19.5883 -28.3512

H -4.0469 19.5563 -29.3562

C -8.0469 20.5143 -19.7422

C -7.9629 20.3913 -21.1172

H -6.9999 20.2003 -21.5592

C -9.1179 20.4953 -21.8942

C -10.3549 20.7243 -21.2802

C -10.4269 20.8693 -19.8882

C -9.2699 20.7613 -19.1312

H -9.3039 20.8633 -18.0592

C -8.0279 19.9653 -23.9502

H -7.2219 20.7023 -23.8182

H -7.6839 18.9943 -23.5652

C -8.3559 19.8503 -25.4392

H -8.6339 20.8343 -25.8162

H -7.4369 19.5383 -25.9402

C -9.4779 18.8563 -25.7532

H -9.4369 18.0263 -25.0442

H -9.3169 18.4423 -26.7522

C -10.8629 19.4993 -25.7052

H -11.0079 19.9703 -24.7332

H -10.9179 20.2843 -26.4642

C -11.9699 18.4783 -25.9572

H -11.9449 17.7193 -25.1702

H -11.7889 17.9683 -26.9072

C -13.3559 19.1203 -25.9872

H -13.5049 19.6933 -25.0702

H -13.4129 19.8213 -26.8242

C -14.4619 18.0773 -26.1202

H -14.3929 17.3753 -25.2852

H -14.3119 17.5053 -27.0392

C -15.8569 18.6983 -26.1352

H -15.9979 19.2953 -25.2332

H -15.9449 19.3713 -26.9922

C -16.9479 17.6333 -26.2152

H -16.8489 16.9533 -25.3642

H -16.8069 17.0413 -27.1232

C -18.3519 18.2313 -26.2182

H -18.4569 18.9003 -27.0762

H -18.4929 18.8293 -25.3152

C -19.4309 17.1543 -26.2872

H -19.2889 16.5613 -27.1932

H -19.3239 16.4803 -25.4332

C -20.8329 17.7503 -26.2892

H -20.9679 18.4023 -27.1492

H -21.5849 16.9653 -26.3352

H -21.0009 18.3353 -25.3872

C -12.3979 19.7313 -21.9342

H -12.2049 19.0123 -22.7432

H -12.2479 19.2163 -20.9792

C -13.8279 20.2523 -22.0232

H -14.0069 20.9203 -21.1802

H -13.9249 20.8383 -22.9382

C -14.8509 19.1193 -22.0272

H -14.7149 18.5033 -21.1342

H -14.6789 18.4793 -22.8952

C -16.2819 19.6493 -22.0642

H -16.4649 20.2623 -21.1802

H -16.4019 20.2923 -22.9392

C -17.3159 18.5273 -22.1272

H -17.1879 17.8663 -21.2662

H -17.1469 17.9313 -23.0262

C -18.7449 19.0673 -22.1412

H -18.9229 19.6453 -21.2322

H -18.8649 19.7443 -22.9902

C -19.7819 17.9523 -22.2452

H -19.6759 17.2763 -21.3932

H -19.5969 17.3713 -23.1502

C -21.2069 18.4983 -22.2802

H -21.4049 19.0543 -21.3622

H -21.3039 19.1993 -23.1142

C -22.2449 17.3893 -22.4332

H -22.1629 16.7013 -21.5892

H -22.0359 16.8213 -23.3432

C -23.6669 17.9403 -22.4992

H -23.8739 18.5143 -21.5932

H -23.7499 18.6233 -23.3492

C -24.7109 16.8363 -22.6402

H -24.6399 16.1613 -21.7852

H -24.4999 16.2533 -23.5402

C -26.1239 17.4003 -22.7222

H -26.2219 18.0623 -23.5792

H -26.8519 16.5983 -22.8232

H -26.3619 17.9663 -21.8242

C -11.8159 21.2283 -17.9822

H -11.4689 20.3193 -17.4692

H -11.2249 22.0833 -17.6242

C -13.2939 21.4623 -17.6992

H -13.4079 21.7573 -16.6562

H -13.6299 22.2933 -18.3212

C -14.1479 20.2323 -17.9932

H -13.9509 19.8993 -19.0132

H -13.8699 19.4203 -17.3182

C -15.6339 20.5413 -17.8372

H -15.9079 21.3293 -18.5422

H -15.8199 20.9283 -16.8322

C -16.5209 19.3243 -18.0812

H -16.3179 18.5643 -17.3242

H -16.2839 18.8913 -19.0552

C -17.9989 19.7003 -18.0432

H -18.2079 20.4053 -18.8512

H -18.2189 20.2113 -17.1022

C -18.9199 18.4923 -18.1852

H -18.6789 17.9583 -19.1062

H -18.7509 17.8073 -17.3512

C -20.3879 18.9083 -18.2112

H -20.5559 19.5623 -19.0712

H -20.6159 19.4863 -17.3122

C -21.3369 17.7173 -18.2962

H -21.1909 17.0733 -17.4262

H -21.0999 17.1303 -19.1872

C -22.7949 18.1633 -18.3592

H -23.0369 18.7373 -17.4612

H -22.9299 18.8223 -19.2202

C -23.7609 16.9883 -18.4812

H -23.6359 16.3253 -17.6222

H -23.5189 16.4153 -19.3792

C -25.2089 17.4553 -18.5562

H -25.3509 18.1143 -19.4102

H -25.8829 16.6093 -18.6612

H -25.4809 18.0023 -17.6552

O -11.4849 20.8173 -22.0622

O -9.1749 20.3803 -23.2462

O -11.6669 21.0863 -19.3782

O -5.6499 20.1803 -19.5252

C 1.6081 20.3053 -16.6802

C 1.6471 19.7723 -15.4032

H 0.7301 19.4133 -14.9652

C 2.8641 19.6993 -14.7332

C 4.0501 20.1413 -15.3442

C 3.9961 20.7143 -16.6252

C 2.7631 20.8103 -17.2622

H 2.6941 21.2493 -18.2442

C 1.9531 18.9483 -12.6502

H 1.1951 18.3273 -13.1442

H 1.4921 19.9133 -12.3882

C 2.4181 18.2303 -11.3842

H 2.7821 17.2383 -11.6562

H 1.5331 18.1083 -10.7562

C 3.5121 18.9723 -10.6122

H 3.3271 20.0473 -10.6702

H 3.4661 18.6873 -9.5582

C 4.9091 18.6653 -11.1482

H 4.9451 18.8923 -12.2142

H 5.1091 17.5973 -11.0292

C 5.9891 19.4623 -10.4222

H 5.7861 20.5283 -10.5482

H 5.9471 19.2513 -9.3492

C 7.3861 19.1513 -10.9582

H 7.3741 19.2343 -12.0462

H 7.6531 18.1213 -10.7082

C 8.4331 20.1053 -10.3892

H 8.1761 21.1263 -10.6842

H 8.4011 20.0613 -9.2972

C 9.8521 19.7963 -10.8632

H 9.8751 19.7813 -11.9542

H 10.1461 18.8033 -10.5122

C 10.8451 20.8343 -10.3462

H 10.5741 21.8163 -10.7422

H 10.7721 20.8853 -9.2562

C 12.2931 20.5263 -10.7182

H 12.5771 19.5583 -10.2972

H 12.3861 20.4533 -11.8032

C 13.2441 21.6013 -10.1962

H 13.1041 21.7113 -9.1172

H 12.9911 22.5603 -10.6572

C 14.7021 21.2673 -10.4832

H 14.9831 20.3363 -9.9952

H 15.3561 22.0563 -10.1162

H 14.8651 21.1543 -11.5522

C 6.1601 20.9243 -14.4852

H 6.1741 21.1733 -13.4152

H 5.8861 21.8233 -15.0392

C 7.5441 20.4393 -14.9042

H 7.5631 20.2993 -15.9852

H 7.7341 19.4693 -14.4382

C 8.6001 21.4513 -14.4752

H 8.3601 22.4133 -14.9302

H 8.5481 21.5783 -13.3902

C 10.0211 21.0423 -14.8572

H 10.1001 20.9603 -15.9432

H 10.2451 20.0593 -14.4372

C 11.0361 22.0603 -14.3422

H 10.7941 23.0433 -14.7522

H 10.9501 22.1283 -13.2552

C 12.4771 21.7133 -14.7072

H 12.5801 21.6773 -15.7942

H 12.7241 20.7213 -14.3212

C 13.4521 22.7393 -14.1342

H 13.1981 23.7293 -14.5212

H 13.3361 22.7733 -13.0492

C 14.9071 22.4293 -14.4712

H 15.0381 22.4353 -15.5552

H 15.1591 21.4273 -14.1152

C 15.8571 23.4453 -13.8392

H 15.5961 24.4463 -14.1922

H 15.7251 23.4343 -12.7552

C 17.3191 23.1633 -14.1712

H 17.4531 23.1773 -15.2552

H 17.5851 22.1633 -13.8192

C 18.2551 24.1873 -13.5342

H 17.9751 25.1883 -13.8692

H 18.1371 24.1573 -12.4492

C 19.7141 23.9313 -13.8922

H 20.0241 22.9463 -13.5502

H 20.3581 24.6743 -13.4282

H 19.8561 23.9803 -14.9692

C 5.1931 21.5863 -18.5062

H 4.7751 22.6033 -18.5362

H 4.5901 20.9343 -19.1512

C 6.6331 21.5783 -19.0132

H 6.6161 21.7953 -20.0842

H 7.0331 20.5713 -18.8802

C 7.5341 22.5793 -18.2962

H 7.5111 22.3793 -17.2262

H 7.1531 23.5903 -18.4462

C 8.9701 22.4943 -18.8112

H 9.3331 21.4693 -18.6962

H 8.9861 22.7283 -19.8792

C 9.9101 23.4413 -18.0702

H 9.5501 24.4663 -18.1762

H 9.8961 23.1943 -17.0072

C 11.3441 23.3523 -18.5862

H 11.7001 22.3243 -18.4772

H 11.3651 23.5943 -19.6512

C 12.2861 24.2883 -17.8342

H 12.2181 24.0783 -16.7642

H 11.9701 25.3233 -17.9892

C 13.7391 24.1333 -18.2772

H 14.0581 23.1013 -18.1072

H 13.8181 24.3263 -19.3502

C 14.6691 25.0763 -17.5182

H 14.3741 26.1103 -17.7152

H 14.5581 24.9003 -16.4462

C 16.1341 24.8873 -17.9032

H 16.2521 25.0373 -18.9792

H 16.4371 23.8613 -17.6822

C 17.0471 25.8563 -17.1562

H 16.7151 26.8793 -17.3452

H 16.9611 25.6713 -16.0832

C 18.5041 25.7143 -17.5762

H 18.8581 24.7023 -17.3912

H 19.1331 26.4053 -17.0182

H 18.6181 25.9263 -18.6372

O 5.1981 19.8893 -14.6512

O 3.0551 19.1663 -13.4942

O 5.1771 21.1043 -17.1772

O -0.8229 20.1113 -16.7852

C -2.9249 19.8583 -12.7582

C -1.6289 19.7553 -12.2402

C -4.0139 19.8833 -11.8832

C -1.4279 19.6603 -10.8862

H -0.7999 19.7543 -12.9292

C -3.8109 19.7933 -10.5252

H -5.0129 19.9743 -12.2792

C -2.5189 19.6683 -10.0152

H -0.4319 19.5793 -10.4812

H -4.6489 19.8153 -9.8462

C -3.1099 19.8133 -23.5282

C -1.8809 19.7093 -24.1842

C -4.2919 19.8323 -24.2742

C -1.8409 19.6423 -25.5582

H -0.9729 19.6823 -23.6022

C -4.2489 19.7633 -25.6462

H -5.2329 19.8993 -23.7532

C -3.0209 19.6693 -26.3002

H -0.9069 19.5723 -26.0902

H -5.1559 19.7833 -26.2282

C -6.8569 20.3643 -18.8232

H -7.0409 19.5003 -18.1642

H -6.7849 21.2653 -18.1932

C 0.3701 20.2543 -17.5372

H 0.4741 19.3923 -18.2152

H 0.3111 21.1683 -18.1422

C -2.2109 16.8303 -21.0522

C -2.1479 16.4133 -26.6962

C -2.1779 16.4073 -27.9042

C -2.2549 16.4313 -29.3072

C -1.0829 16.4443 -30.0902

C -3.5059 16.4963 -29.9202

H -4.3699 16.5103 -29.2752

O -3.2509 16.7213 -20.4172

O -1.0539 17.0983 -20.4932

H -1.1259 17.1483 -19.4902

C -2.3549 16.4503 -40.3632

C -2.5449 16.4203 -34.6972

C -2.5379 16.4483 -33.4882

C -2.4659 16.4713 -32.0832

C -3.6319 16.5703 -31.2982

C -1.2119 16.4233 -31.4682

H -0.3449 16.3733 -32.1082

O -1.2949 16.5733 -40.9642

O -3.5079 16.2903 -40.9662

H -3.4059 16.2123 -41.9662

C -7.0779 17.6263 -31.9522

C -7.0849 17.6603 -33.3332

H -6.1949 17.3603 -33.8602

C -8.2429 18.0483 -34.0042

C -9.3969 18.3743 -33.2782

C -9.3589 18.3963 -31.8752

C -8.1909 18.0283 -31.2272

H -8.1329 18.0243 -30.1512

C -7.3189 17.6433 -36.1712

H -6.3899 18.1953 -35.9672

H -7.1449 16.5793 -35.9582

C -7.6899 17.8263 -37.6422

H -7.9119 18.8813 -37.8142

H -6.8059 17.5603 -38.2262

C -8.8789 16.9743 -38.0952

H -8.7979 15.9803 -37.6512

H -8.8309 16.8513 -39.1802

C -10.2279 17.5903 -37.7252

H -10.2389 17.8193 -36.6602

H -10.3509 18.5333 -38.2632

C -11.3969 16.6663 -38.0592

H -11.2869 15.7303 -37.5052

H -11.3749 16.4173 -39.1242

C -12.7409 17.3013 -37.7122

H -12.7319 17.6073 -36.6652

H -12.8819 18.2033 -38.3112

C -13.9109 16.3473 -37.9462

H -13.7729 15.4523 -37.3342

H -13.9169 16.0283 -38.9922

C -15.2549 16.9873 -37.6062

H -15.2379 17.3353 -36.5722

H -15.4109 17.8613 -38.2422

C -16.4179 16.0143 -37.7892

H -16.2849 15.1623 -37.1162

H -16.4059 15.6243 -38.8112

C -17.7689 16.6693 -37.5162

H -17.9119 17.5013 -38.2072

H -17.7749 17.0783 -36.5042

C -18.9279 15.6863 -37.6662

H -18.8949 15.2373 -38.6622

H -18.8119 14.8763 -36.9412

C -20.2789 16.3613 -37.4612

H -20.4219 17.1533 -38.1932

H -21.0879 15.6433 -37.5702

H -20.3399 16.7993 -36.4682

C -11.5589 17.6283 -33.8022

H -11.5469 16.9943 -34.7012

H -11.3299 16.9933 -32.9402

C -12.9299 18.2663 -33.6242

H -12.9229 18.8513 -32.7052

H -13.0999 18.9533 -34.4552

C -14.0419 17.2223 -33.5812

H -13.8729 16.5403 -32.7442

H -14.0159 16.6263 -34.4972

C -15.4149 17.8743 -33.4402

H -15.4559 18.4313 -32.5022

H -15.5529 18.5913 -34.2532

C -16.5519 16.8563 -33.4762

H -16.4359 16.1473 -32.6532

H -16.4989 16.2873 -34.4082

C -17.9159 17.5343 -33.3752

H -17.9779 18.0803 -32.4322

H -18.0119 18.2653 -34.1822

C -19.0699 16.5393 -33.4612

H -18.9909 15.8203 -32.6422

H -18.9979 15.9793 -34.3962

C -20.4269 17.2373 -33.3902

H -20.4989 17.7883 -32.4512

H -20.5019 17.9643 -34.2032

C -21.5919 16.2563 -33.4902

H -21.5349 15.5423 -32.6652

H -21.5099 15.6883 -34.4202

C -22.9399 16.9703 -33.4512

H -23.0169 17.5393 -32.5222

H -22.9949 17.6793 -34.2812

C -24.1139 15.9993 -33.5422

H -24.0649 15.2933 -32.7102

H -24.0349 15.4243 -34.4682

C -25.4549 16.7233 -33.5092

H -25.5309 17.4243 -34.3382

H -26.2749 16.0133 -33.5842

H -25.5669 17.2773 -32.5802

C -10.4969 18.8073 -29.8262

H -10.2169 17.8323 -29.4022

H -9.7659 19.5583 -29.4982

C -11.8939 19.1933 -29.3542

H -11.8429 19.4633 -28.2982

H -12.2079 20.0733 -29.9152

C -12.9099 18.0703 -29.5522

H -12.8269 17.6923 -30.5722

H -12.6839 17.2473 -28.8702

C -14.3329 18.5643 -29.3082

H -14.5599 19.3493 -30.0342

H -14.3959 19.0103 -28.3142

C -15.3799 17.4613 -29.4332

H -15.2339 16.7243 -28.6402

H -15.2539 16.9463 -30.3882

C -16.7929 18.0323 -29.3492

H -16.9349 18.7423 -30.1672

H -16.9019 18.5843 -28.4132

C -17.8779 16.9623 -29.4292

H -17.7559 16.3893 -30.3512

H -17.7689 16.2683 -28.5922

C -19.2719 17.5833 -29.3982

H -19.3769 18.2643 -30.2472

H -19.3799 18.1743 -28.4862

C -20.3849 16.5413 -29.4552

H -20.2899 15.8593 -28.6072

H -20.2809 15.9483 -30.3672

C -21.7659 17.1903 -29.4262

H -21.8579 17.7953 -28.5222

H -21.8659 17.8593 -30.2852

C -22.8949 16.1653 -29.4572

H -22.7939 15.4913 -28.6032

H -22.8129 15.5633 -30.3652

C -24.2639 16.8323 -29.4152

H -24.3909 17.4953 -30.2692

H -25.0569 16.0883 -29.4402

H -24.3739 17.4213 -28.5072

O -10.5669 18.6403 -33.9532

O -8.3659 18.1273 -35.3562

O -10.5089 18.7413 -31.2392

O -4.8109 16.7543 -31.9512

C 2.3611 17.1023 -29.1302

C 2.1231 17.9033 -28.0242

H 1.1481 18.3453 -27.9082

C 3.1291 18.1013 -27.0822

C 4.3871 17.5153 -27.2822

C 4.6311 16.7463 -28.4272

C 3.6041 16.5283 -29.3392

H 3.7621 15.9053 -30.2052

C 1.7721 19.4343 -25.6432

H 0.9501 18.7233 -25.8052

H 1.6171 20.3033 -26.3002

C 1.7741 19.8693 -24.1782

H 1.8651 18.9823 -23.5492

H 0.8031 20.3263 -23.9812

C 2.8921 20.8563 -23.8312

H 3.1271 21.4583 -24.7082

H 2.5401 21.5303 -23.0452

C 4.1661 20.1673 -23.3482

H 4.4861 19.4413 -24.0952

H 3.9521 19.6253 -22.4222

C 5.2811 21.1793 -23.0992

H 5.5561 21.6463 -24.0472

H 4.9131 21.9693 -22.4372

C 6.5181 20.5413 -22.4712

H 6.8211 19.6823 -23.0712

H 6.2771 20.1763 -21.4692

C 7.6741 21.5333 -22.3962

H 7.9121 21.8563 -23.4112

H 7.3581 22.4173 -21.8342

C 8.9311 20.9513 -21.7552

H 9.1981 20.0233 -22.2642

H 8.7331 20.7083 -20.7082

C 10.1021 21.9273 -21.8422

H 10.3041 22.1463 -22.8932

H 9.8251 22.8673 -21.3572

C 11.3731 21.3843 -21.1942

H 11.1931 21.2113 -20.1312

H 11.6261 20.4223 -21.6472

C 12.5501 22.3423 -21.3592

H 12.2901 23.3093 -20.9212

H 12.7371 22.5013 -22.4242

C 13.8161 21.8153 -20.6982

H 13.6611 21.6823 -19.6302

H 14.6431 22.5083 -20.8412

H 14.0981 20.8543 -21.1242

C 6.2771 18.7383 -26.6142

H 5.8471 19.6893 -26.2712

H 6.4501 18.8133 -27.6942

C 7.5941 18.4633 -25.8992

H 8.0241 17.5413 -26.2972

H 7.3881 18.2983 -24.8412

C 8.5621 19.6263 -26.0772

H 8.6811 19.8333 -27.1432

H 8.1281 20.5173 -25.6192

C 9.9321 19.3453 -25.4662

H 10.3571 18.4473 -25.9222

H 9.8221 19.1473 -24.3982

C 10.8891 20.5173 -25.6682

H 10.9661 20.7413 -26.7352

H 10.4761 21.4003 -25.1762

C 12.2831 20.2393 -25.1122

H 12.7101 19.3673 -25.6152

H 12.2071 19.9993 -24.0502

C 13.2131 21.4353 -25.2922

H 13.2651 21.6993 -26.3512

H 12.7941 22.2953 -24.7632

C 14.6211 21.1613 -24.7702

H 15.0671 20.3433 -25.3422

H 14.5651 20.8393 -23.7282

C 15.5141 22.3953 -24.8662

H 15.5141 22.7603 -25.8972

H 15.1011 23.1883 -24.2392

C 16.9501 22.1083 -24.4352

H 17.3671 21.3183 -25.0642

H 16.9531 21.7443 -23.4052

C 17.8331 23.3493 -24.5352

H 17.7801 23.7463 -25.5522

H 17.4501 24.1183 -23.8612

C 19.2861 23.0493 -24.1902

H 19.3651 22.6583 -23.1782

H 19.8901 23.9523 -24.2562

H 19.6961 22.3103 -24.8752

C 6.2391 15.5353 -29.7122

H 6.0081 16.1263 -30.6102

H 5.6781 14.5913 -29.7592

C 7.7381 15.2593 -29.6472

H 8.0291 14.6883 -30.5312

H 7.9461 14.6563 -28.7622

C 8.5271 16.5633 -29.5712

H 8.2121 17.1033 -28.6782

H 8.2841 17.1793 -30.4402

C 10.0341 16.3353 -29.5202

H 10.2701 15.6403 -28.7112

H 10.3671 15.8773 -30.4552

C 10.7881 17.6433 -29.2962

H 10.5111 18.3563 -30.0762

H 10.4861 18.0703 -28.3382

C 12.3011 17.4473 -29.3072

H 12.5751 16.7113 -28.5472

H 12.6091 17.0493 -30.2772

C 13.0471 18.7513 -29.0372

H 12.7221 19.1573 -28.0762

H 12.7861 19.4823 -29.8062

C 14.5601 18.5563 -29.0132

H 14.8171 17.8363 -28.2312

H 14.8891 18.1363 -29.9662

C 15.2991 19.8663 -28.7542

H 15.0901 20.5653 -29.5672

H 14.9201 20.3123 -27.8312

C 16.8071 19.6713 -28.6292

H 17.1931 19.2173 -29.5452

H 17.0151 18.9803 -27.8072

C 17.5301 20.9913 -28.3722

H 17.3521 21.6673 -29.2102

H 17.1111 21.4563 -27.4772

C 19.0291 20.8033 -28.1832

H 19.2301 20.1523 -27.3342

H 19.5151 21.7603 -28.0042

H 19.4741 20.3533 -29.0682

O 5.3751 17.6723 -26.3392

O 3.0071 18.8233 -25.9452

O 5.8901 16.2573 -28.5502

O 0.0901 16.4733 -29.4142

C -2.1449 16.4643 -25.2902

C -0.9409 16.5073 -24.5802

C -3.3599 16.4973 -24.5952

C -0.9519 16.6183 -23.2072

H -0.0079 16.4563 -25.1212

C -3.3659 16.5973 -23.2242

H -4.2859 16.4523 -25.1482

C -2.1639 16.6763 -22.5192

H -0.0279 16.6613 -22.6522

H -4.2919 16.6303 -22.6702

C -2.5159 16.4243 -36.1042

C -1.2889 16.4903 -36.7732

C -3.6979 16.3673 -36.8492

C -1.2449 16.5163 -38.1482

H -0.3759 16.5083 -36.1962

C -3.6529 16.3803 -38.2252

H -4.6439 16.3073 -36.3322

C -2.4269 16.4573 -38.8872

H -0.3029 16.5713 -38.6722

H -4.5599 16.3273 -38.8072

C -5.9029 17.1433 -31.1462

H -6.2359 16.2903 -30.5342

H -5.5909 17.9513 -30.4702

C 1.2521 16.8593 -30.1202

H 1.5501 16.0773 -30.8312

H 1.0421 17.7843 -30.6792

C -2.1829 16.1183 -44.0502

C -2.0349 15.6923 -49.6942

C -2.0739 15.6983 -50.9032

C -2.1379 15.7313 -52.3072

C -0.9559 15.8553 -53.0682

C -3.3799 15.6923 -52.9422

H -4.2559 15.6243 -52.3162

O -3.2269 15.9513 -43.4352

O -1.0579 16.4653 -43.4692

H -1.1589 16.5233 -42.4702

C -2.9579 15.9733 -63.3132

C -2.3799 15.6633 -57.6952

C -2.3599 15.7153 -56.4872

C -2.2979 15.7583 -55.0832

C -3.4859 15.7633 -54.3222

C -1.0559 15.8233 -54.4482

H -0.1759 15.8503 -55.0712

O -2.0199 16.2073 -64.0652

O -4.2009 15.8633 -63.7302

H -4.2889 15.9893 -64.7222

C -6.9249 16.7373 -55.1002

C -6.9219 16.6683 -56.4822

H -6.0569 16.2593 -56.9782

C -8.0349 17.1103 -57.1962

C -9.1579 17.5913 -56.5112

C -9.1289 17.7093 -55.1142

C -8.0029 17.2893 -54.4212

H -7.9569 17.3663 -53.3472

C -7.1259 16.5513 -59.3402

H -6.1719 17.0603 -59.1402

H -7.0089 15.4843 -59.1032

C -7.4859 16.7243 -60.8152

H -7.6739 17.7823 -61.0032

H -6.6099 16.4243 -61.3922

C -8.7009 15.9073 -61.2632

H -8.6779 14.9303 -60.7762

H -8.6349 15.7383 -62.3412

C -10.0269 16.6013 -60.9542

H -10.0459 16.8903 -59.9042

H -10.0939 17.5153 -61.5482

C -11.2289 15.7113 -61.2632

H -11.1869 14.8163 -60.6372

H -11.1819 15.3843 -62.3052

C -12.5519 16.4353 -61.0172

H -12.5609 16.8293 -60.0012

H -12.6269 17.2883 -61.6952

C -13.7599 15.5223 -61.2172

H -13.7229 14.7073 -60.4892

H -13.7129 15.0703 -62.2122

C -15.0809 16.2753 -61.0692

H -15.1109 16.7663 -60.0952

H -15.1349 17.0573 -61.8292

C -16.2919 15.3563 -61.2112

H -16.3049 14.6373 -60.3872

H -16.2039 14.7813 -62.1382

C -17.6029 16.1373 -61.2292

H -17.5899 16.8353 -62.0692

H -17.6869 16.7263 -60.3132

C -18.8219 15.2273 -61.3572

H -18.7089 14.5943 -62.2402

H -18.8769 14.5663 -60.4892

C -20.1149 16.0253 -61.4722

H -20.0889 16.6633 -62.3532

H -20.9719 15.3603 -61.5582

H -20.2539 16.6553 -60.5972

C -11.3349 16.9573 -57.0832

H -11.2509 16.2333 -57.9072

H -11.2279 16.4073 -56.1412

C -12.6909 17.6493 -57.1232

H -12.7619 18.3243 -56.2702

H -12.7449 18.2543 -58.0292

C -13.8389 16.6453 -57.0992

H -13.7639 16.0253 -56.2022

H -13.7589 15.9793 -57.9622

C -15.1969 17.3423 -57.1242

H -15.2979 17.9713 -56.2382

H -15.2449 17.9983 -57.9962

C -16.3559 16.3523 -57.1812

H -16.3159 15.6943 -56.3102

H -16.2529 15.7253 -58.0692

C -17.7069 17.0613 -57.2202

H -17.8239 17.6683 -56.3202

H -17.7289 17.7403 -58.0762

C -18.8709 16.0813 -57.3282

H -18.8559 15.4053 -56.4692

H -18.7469 15.4723 -58.2262

C -20.2189 16.7933 -57.3842

H -20.3479 17.3943 -56.4812

H -20.2319 17.4773 -58.2372

C -21.3829 15.8143 -57.5112

H -21.3769 15.1363 -56.6542

H -21.2499 15.2093 -58.4102

C -22.7289 16.5313 -57.5782

H -22.8489 17.1543 -56.6892

H -22.7429 17.1903 -58.4492

C -23.8989 15.5543 -57.6672

H -23.8899 14.9003 -56.7922

H -23.7759 14.9253 -58.5522

C -25.2389 16.2763 -57.7422

H -25.2759 16.9243 -58.6152

H -26.0559 15.5613 -57.8122

H -25.3949 16.8863 -56.8552

C -10.3119 18.3323 -53.1352

H -10.0859 17.3703 -52.6522

H -9.5789 19.0793 -52.8012

C -11.7279 18.7713 -52.7772

H -11.7569 19.0493 -51.7232

H -11.9669 19.6553 -53.3702

C -12.7499 17.6723 -53.0602

H -12.5789 17.2823 -54.0642

H -12.6069 16.8503 -52.3532

C -14.1809 18.1913 -52.9592

H -14.3269 18.9703 -53.7112

H -14.3329 18.6503 -51.9802

C -15.2229 17.0963 -53.1682

H -15.1339 16.3503 -52.3742

H -15.0339 16.5893 -54.1162

C -16.6369 17.6703 -53.1722

H -16.7309 18.3763 -54.0012

H -16.8009 18.2263 -52.2482

C -17.7159 16.5993 -53.3092

H -17.5519 16.0313 -54.2272

H -17.6419 15.8993 -52.4732

C -19.1119 17.2143 -53.3352

H -19.1979 17.8683 -54.2062

H -19.2489 17.8323 -52.4452

C -20.2169 16.1623 -53.3902

H -20.1489 15.5203 -52.5092

H -20.0729 15.5323 -54.2712

C -21.6029 16.7983 -53.4472

H -21.7489 17.4253 -52.5642

H -21.6649 17.4453 -54.3262

C -22.7179 15.7583 -53.5182

H -22.6679 15.1153 -52.6362

H -22.5649 15.1273 -54.3962

C -24.0949 16.4063 -53.5962

H -24.1689 17.0353 -54.4802

H -24.8739 15.6483 -53.6512

H -24.2769 17.0243 -52.7192

O -10.2959 17.9233 -57.2112

O -8.1539 17.1063 -58.5502

O -10.2549 18.2013 -54.5402

O -4.6579 15.8593 -55.0062

C 2.3921 16.8073 -52.0282

C 2.0541 17.5643 -50.9182

H 1.0461 17.9353 -50.8322

C 3.0011 17.8043 -49.9292

C 4.3071 17.3093 -50.0782

C 4.6531 16.5903 -51.2312

C 3.6821 16.3313 -52.1932

H 3.9191 15.7453 -53.0662

C 1.5091 19.0333 -48.5232

H 0.7171 18.3113 -48.7642

H 1.3661 19.9273 -49.1482

C 1.4181 19.3973 -47.0422

H 1.4731 18.4813 -46.4502

H 0.4381 19.8473 -46.8822

C 2.5111 20.3643 -46.5822

H 2.7471 21.0483 -47.3962

H 2.1331 20.9543 -45.7422

C 3.7921 19.6533 -46.1522

H 4.1121 18.9833 -46.9482

H 3.5921 19.0513 -45.2622

C 4.9071 20.6523 -45.8582

H 5.1001 21.2383 -46.7592

H 4.5871 21.3433 -45.0722

C 6.1981 19.9613 -45.4292

H 6.4461 19.1963 -46.1672

H 6.0541 19.4603 -44.4682

C 7.3521 20.9543 -45.3332

H 7.4351 21.4683 -46.2922

H 7.1251 21.7113 -44.5772

C 8.6871 20.2913 -45.0052

H 8.8561 19.4643 -45.6982

H 8.6561 19.8763 -43.9952

C 9.8431 21.2823 -45.1192

H 9.8811 21.6593 -46.1442

H 9.6551 22.1383 -44.4662

C 11.1921 20.6633 -44.7652

H 11.1781 20.3323 -43.7242

H 11.3611 19.7843 -45.3912

C 12.3381 21.6513 -44.9682

H 12.1571 22.5433 -44.3632

H 12.3571 21.9633 -46.0152

C 13.6881 21.0533 -44.5942

H 13.6971 20.7593 -43.5472

H 14.4861 21.7763 -44.7532

H 13.8961 20.1753 -45.2012

C 6.2591 18.4113 -49.3162

H 6.0081 19.3773 -48.8562

H 6.3641 18.5683 -50.3922

C 7.5721 17.8803 -48.7482

H 7.8321 16.9563 -49.2672

H 7.4341 17.6393 -47.6932

C 8.6721 18.9233 -48.9182

H 8.6981 19.2403 -49.9642

H 8.4191 19.8003 -48.3202

C 10.0581 18.4223 -48.5192

H 10.3141 17.5413 -49.1122

H 10.0541 18.1193 -47.4702

C 11.1091 19.5103 -48.7342

H 11.0541 19.8563 -49.7702

H 10.8751 20.3623 -48.0932

C 12.5331 19.0413 -48.4462

H 12.7791 18.1983 -49.0962

H 12.6011 18.6913 -47.4142

C 13.5401 20.1673 -48.6672

H 13.4371 20.5473 -49.6882

H 13.3091 20.9923 -47.9892

C 14.9841 19.7263 -48.4452

H 15.2381 18.9383 -49.1572

H 15.0861 19.3093 -47.4402

C 15.9571 20.8913 -48.6062

H 15.8411 21.3213 -49.6052

H 15.7061 21.6713 -47.8832

C 17.4111 20.4713 -48.4102

H 17.6711 19.7093 -49.1482

H 17.5271 20.0263 -47.4192

C 18.3701 21.6523 -48.5452

H 18.2581 22.0943 -49.5392

H 18.1041 22.4163 -47.8112

C 19.8221 21.2393 -48.3432

H 19.9621 20.8063 -47.3552

H 20.4811 22.0993 -48.4372

H 20.1151 20.5013 -49.0862

C 6.3181 15.3313 -52.3852

H 6.1671 15.8433 -53.3462

H 5.7081 14.4183 -52.3732

C 7.7931 14.9803 -52.2182

H 8.0621 14.2453 -52.9792

H 7.9351 14.5253 -51.2362

C 8.6751 16.2173 -52.3472

H 8.4021 16.9233 -51.5622

H 8.4791 16.6943 -53.3102

C 10.1611 15.8913 -52.2452

H 10.3581 15.3663 -51.3082

H 10.4421 15.2223 -53.0632

C 11.0121 17.1553 -52.3092

H 10.7481 17.7193 -53.2072

H 10.7841 17.7873 -51.4482

C 12.5081 16.8543 -52.3302

H 12.7751 16.2773 -51.4412

H 12.7411 16.2403 -53.2032

C 13.3401 18.1333 -52.3742

H 13.1131 18.7403 -51.4962

H 13.0571 18.7163 -53.2542

C 14.8391 17.8493 -52.4132

H 15.1221 17.2893 -51.5192

H 15.0701 17.2233 -53.2782

C 15.6581 19.1353 -52.4852

H 15.4081 19.6713 -53.4052

H 15.3871 19.7793 -51.6472

C 17.1601 18.8693 -52.4462

H 17.4401 18.2373 -53.2932

H 17.4041 18.3213 -51.5332

C 17.9681 20.1633 -52.4872

H 17.7441 20.6993 -53.4132

H 17.6641 20.8003 -51.6552

C 19.4671 19.9053 -52.4022

H 19.7101 19.3743 -51.4842

H 20.0181 20.8433 -52.4112

H 19.8011 19.3013 -53.2432

O 5.2011 17.4933 -49.0552

O 2.7791 18.4743 -48.7772

O 5.9461 16.1753 -51.3162

O 0.1971 15.9903 -52.3722

C -2.0399 15.7353 -48.2882

C -0.8549 15.9343 -47.5712

C -3.2509 15.6073 -47.5972

C -0.8879 16.0473 -46.1982

H 0.0801 16.0023 -48.1062

C -3.2769 15.7113 -46.2262

H -4.1599 15.4363 -48.1532

C -2.1009 15.9523 -45.5152

H 0.0201 16.2123 -45.6392

H -4.2029 15.6233 -45.6782

C -2.4839 15.6633 -59.0972

C -1.4009 16.0203 -59.9072

C -3.7039 15.3253 -59.6932

C -1.5509 16.0953 -61.2722

H -0.4499 16.2463 -59.4492

C -3.8519 15.3993 -61.0582

H -4.5229 15.0073 -59.0662

C -2.7859 15.8123 -61.8592

H -0.7269 16.3883 -61.9042

H -4.7889 15.1353 -61.5242

C -5.8019 16.2003 -54.2502

H -6.1679 15.3103 -53.7132

H -5.5399 16.9663 -53.5092

C 1.3341 16.4863 -53.0512

H 1.7151 15.7423 -53.7642

H 1.0551 17.3963 -53.6062

C -1.5319 25.6183 -31.4612

C -1.3269 25.6313 -37.1252

C -1.3939 25.5783 -38.3322

C -1.5789 25.5213 -39.7252

C -0.5139 25.2533 -40.6092

C -2.8749 25.6923 -40.2232

H -3.6639 25.8543 -39.5052

O -2.6159 25.6343 -30.8952

O -0.3879 25.5913 -30.8132

H -0.5269 25.6123 -29.8152

C -1.8019 25.0693 -50.7132

C -2.1719 25.4683 -45.0742

C -2.2149 25.4813 -43.8652

C -2.0669 25.4763 -42.4662

C -3.1489 25.6423 -41.5802

C -0.7809 25.2673 -41.9682

H -0.0079 25.0943 -42.6982

O -0.7229 25.0233 -51.2902

O -2.9559 25.0023 -51.3312

H -2.8449 24.9263 -52.3302

C -6.7629 25.5283 -42.1292

C -6.7249 25.7783 -43.4882

H -5.7739 25.9863 -43.9472

C -7.9009 25.7553 -44.2292

C -9.1249 25.4903 -43.6012

C -9.1569 25.2583 -42.2172

C -7.9679 25.2553 -41.4972

H -7.9699 25.0543 -40.4372

C -6.7819 26.0383 -46.3032

H -6.1559 26.8853 -45.9852

H -6.2239 25.1053 -46.1302

C -7.1109 26.1813 -47.7872

H -7.6339 27.1263 -47.9422

H -6.1629 26.2273 -48.3272

C -7.9729 25.0323 -48.3172

H -7.7129 24.1153 -47.7862

H -7.7559 24.8773 -49.3782

C -9.4659 25.3033 -48.1462

H -9.6629 25.5633 -47.1062

H -9.7429 26.1603 -48.7652

C -10.3249 24.1023 -48.5372

H -10.1249 23.2743 -47.8552

H -10.0639 23.7733 -49.5482

C -11.8089 24.4543 -48.4882

H -12.0339 24.8893 -47.5132

H -12.0249 25.2143 -49.2442

C -12.7109 23.2443 -48.7142

H -12.5119 22.5073 -47.9332

H -12.4709 22.7803 -49.6732

C -14.1859 23.6363 -48.6922

H -14.4129 24.1223 -47.7412

H -14.3759 24.3653 -49.4852

C -15.1159 22.4413 -48.8782

H -14.9309 21.7183 -48.0792

H -14.8889 21.9473 -49.8252

C -16.5869 22.8483 -48.8562

H -16.7729 23.5693 -49.6562

H -16.8099 23.3433 -47.9082

C -17.5169 21.6513 -49.0302

H -17.2859 21.1533 -49.9742

H -17.3319 20.9343 -48.2262

C -18.9849 22.0543 -49.0152

H -19.1909 22.7713 -49.8062

H -19.6229 21.1863 -49.1662

H -19.2459 22.5123 -48.0642

C -11.0849 24.3933 -44.4072

H -10.8439 23.8123 -45.3072

H -10.9079 23.7563 -43.5372

C -12.5399 24.8483 -44.4432

H -12.7569 25.3933 -43.5232

H -12.6649 25.5453 -45.2752

C -13.4879 23.6673 -44.6002

H -13.3239 22.9653 -43.7792

H -13.2529 23.1403 -45.5262

C -14.9509 24.1033 -44.6152

H -15.1819 24.6353 -43.6892

H -15.1129 24.8013 -45.4402

C -15.8919 22.9123 -44.7672

H -15.7139 22.2093 -43.9482

H -15.6629 22.3903 -45.6982

C -17.3629 23.3183 -44.7642

H -17.5959 23.8383 -43.8332

H -17.5499 24.0163 -45.5842

C -18.2749 22.1043 -44.9132

H -18.0719 21.4043 -44.0982

H -18.0399 21.5903 -45.8472

C -19.7559 22.4683 -44.8972

H -19.9979 22.9663 -43.9552

H -19.9679 23.1743 -45.7042

C -20.6349 21.2313 -45.0602

H -20.3959 20.5193 -44.2652

H -20.4019 20.7463 -46.0102

C -22.1249 21.5533 -45.0082

H -22.3589 22.0353 -44.0562

H -22.3739 22.2583 -45.8052

C -22.9779 20.2963 -45.1602

H -22.6949 19.5783 -44.3862

H -22.7669 19.8333 -46.1272

C -24.4679 20.5893 -45.0502

H -24.7749 21.3003 -45.8142

H -25.0469 19.6773 -45.1792

H -24.7029 21.0113 -44.0752

C -10.5309 25.0393 -40.2652

H -10.2189 24.0603 -39.8722

H -9.9069 25.8163 -39.8072

C -11.9949 25.3143 -39.9302

H -12.0869 25.4053 -38.8452

H -12.2699 26.2713 -40.3782

C -12.9379 24.2293 -40.4432

H -12.7719 24.0933 -41.5122

H -12.7089 23.2843 -39.9462

C -14.3979 24.6023 -40.1922

H -14.6309 25.5233 -40.7322

H -14.5399 24.8053 -39.1262

C -15.3639 23.5043 -40.6272

H -15.1639 22.6003 -40.0492

H -15.1919 23.2693 -41.6782

C -16.8259 23.9053 -40.4392

H -17.0519 24.7633 -41.0762

H -16.9879 24.2153 -39.4042

C -17.7739 22.7573 -40.7762

H -17.5799 22.4153 -41.7942

H -17.5709 21.9193 -40.1052

C -19.2429 23.1533 -40.6522

H -19.4669 23.9463 -41.3702

H -19.4279 23.5563 -39.6532

C -20.1699 21.9653 -40.8972

H -19.9579 21.1903 -40.1592

H -19.9649 21.5443 -41.8832

C -21.6459 22.3463 -40.8102

H -21.8449 22.8153 -39.8432

H -21.8769 23.0833 -41.5842

C -22.5559 21.1323 -40.9782

H -22.3379 20.4093 -40.1882

H -22.3419 20.6503 -41.9332

C -24.0309 21.5093 -40.9222

H -24.2719 22.2273 -41.7022

H -24.6559 20.6293 -41.0592

H -24.2749 21.9573 -39.9612

O -10.2529 25.5473 -44.3732

O -7.9779 26.0023 -45.5612

O -10.3889 25.0593 -41.6702

O -4.3799 25.7413 -42.1352

C 2.9881 24.2283 -40.3022

C 3.0681 23.5773 -39.0842

H 2.1571 23.2713 -38.5972

C 4.3111 23.3553 -38.5022

C 5.4811 23.7663 -39.1602

C 5.3901 24.3983 -40.4082

C 4.1381 24.6373 -40.9572

H 4.0451 25.1413 -41.9052

C 3.3881 22.4903 -36.4852

H 2.7311 21.7513 -36.9682

H 2.8161 23.4163 -36.3282

C 3.8751 21.9423 -35.1452

H 4.3201 20.9603 -35.3142

H 2.9931 21.8143 -34.5122

C 4.8951 22.8533 -34.4482

H 4.6981 23.8923 -34.7172

H 4.7741 22.7673 -33.3642

C 6.3331 22.4933 -34.8192

H 6.4351 22.5083 -35.9032

H 6.5361 21.4763 -34.4722

C 7.3691 23.4323 -34.2022

H 7.2871 24.4183 -34.6652

H 7.1711 23.5553 -33.1342

C 8.7851 22.8923 -34.3982

H 8.9121 22.6053 -35.4442

H 8.9111 21.9923 -33.7902

C 9.8671 23.9013 -34.0222

H 9.7731 24.7843 -34.6582

H 9.7201 24.2233 -32.9882

C 11.2711 23.3203 -34.1742

H 11.4031 22.9573 -35.1972

H 11.3801 22.4633 -33.5052

C 12.3581 24.3443 -33.8582

H 12.2611 25.1943 -34.5372

H 12.2131 24.7173 -32.8422

C 13.7631 23.7603 -33.9822

H 13.8631 22.9103 -33.3022

H 13.9151 23.3883 -34.9982

C 14.8421 24.7903 -33.6552

H 14.6811 25.1693 -32.6442

H 14.7541 25.6343 -34.3422

C 16.2441 24.2013 -33.7552

H 16.3611 23.3703 -33.0622

H 16.9931 24.9533 -33.5182

H 16.4331 23.8333 -34.7622

C 7.4191 24.6783 -38.1652

H 7.0891 24.9763 -37.1592

H 7.2251 25.5093 -38.8502

C 8.9041 24.3413 -38.1662

H 9.1621 23.9753 -39.1602

H 9.0771 23.5333 -37.4542

C 9.7851 25.5343 -37.8092

H 9.6101 26.3493 -38.5162

H 9.5231 25.9003 -36.8142

C 11.2591 25.1373 -37.8392

H 11.5041 24.7573 -38.8342

H 11.4221 24.3263 -37.1272

C 12.2031 26.2883 -37.5002

H 12.0551 27.1083 -38.2082

H 11.9721 26.6703 -36.5032

C 13.6581 25.8283 -37.5472

H 13.8801 25.4513 -38.5492

H 13.7921 25.0013 -36.8472

C 14.6481 26.9373 -37.2012

H 14.5291 27.7673 -37.9022

H 14.4331 27.3193 -36.2012

C 16.0861 26.4283 -37.2542

H 16.2911 26.0443 -38.2572

H 16.1981 25.5973 -36.5532

C 17.1081 27.5093 -36.9132

H 16.9971 28.3453 -37.6082

H 16.9151 27.8883 -35.9072

C 18.5381 26.9773 -36.9872

H 18.7171 26.5633 -37.9832

H 18.6581 26.1633 -36.2682

C 19.5761 28.0573 -36.7012

H 19.4401 28.8843 -37.4012

H 19.4221 28.4493 -35.6932

C 20.9971 27.5213 -36.8252

H 21.1591 26.7043 -36.1252

H 21.7241 28.3023 -36.6152

H 21.1761 27.1473 -37.8322

C 6.5051 25.4503 -42.2362

H 6.0211 26.4263 -42.0872

H 5.9241 24.8783 -42.9732

C 7.9231 25.6413 -42.7612

H 7.8581 26.1803 -43.7082

H 8.3581 24.6603 -42.9572

C 8.8201 26.4053 -41.7912

H 8.9281 25.8233 -40.8762

H 8.3531 27.3573 -41.5302

C 10.1931 26.6603 -42.4072

H 10.6001 25.7153 -42.7752

H 10.0831 27.3253 -43.2662

C 11.1841 27.2703 -41.4202

H 10.7801 28.2033 -41.0192

H 11.3261 26.5843 -40.5822

C 12.5311 27.5403 -42.0862

H 12.8801 26.6213 -42.5652

H 12.4021 28.2903 -42.8702

C 13.5951 28.0163 -41.1002

H 13.7261 27.2593 -40.3232

H 13.2601 28.9353 -40.6132

C 14.9341 28.2623 -41.7922

H 15.2241 27.3633 -42.3402

H 14.8241 29.0683 -42.5222

C 16.0401 28.6183 -40.8032

H 15.7741 29.5353 -40.2702

H 16.1261 27.8213 -40.0612

C 17.3891 28.8053 -41.4942

H 17.3321 29.6523 -42.1822

H 17.6171 27.9163 -42.0882

C 18.5181 29.0363 -40.4932

H 18.3011 29.9313 -39.9042

H 18.5621 28.1893 -39.8062

C 19.8681 29.1973 -41.1802

H 20.1081 28.3073 -41.7582

H 20.6551 29.3523 -40.4462

H 19.8551 30.0493 -41.8562

O 6.6961 23.5193 -38.5662

O 4.5061 22.7563 -37.3012

O 6.5611 24.7523 -41.0082

O 0.7061 24.9863 -40.0762

C -1.3329 25.6543 -35.7182

C -0.1659 25.4623 -34.9722

C -2.5479 25.8503 -35.0502

C -0.2119 25.4593 -33.5952

H 0.7711 25.3183 -35.4912

C -2.5929 25.8333 -33.6762

H -3.4479 26.0033 -35.6272

C -1.4269 25.6383 -32.9342

H 0.6881 25.3193 -33.0162

H -3.5249 25.9683 -33.1482

C -2.0999 25.4233 -46.4792

C -0.8539 25.4993 -47.1102

C -3.2529 25.2743 -47.2542

C -0.7639 25.3973 -48.4802

H 0.0321 25.6353 -46.5082

C -3.1599 25.1733 -48.6242

H -4.2109 25.2253 -46.7642

C -1.9139 25.2193 -49.2482

H 0.1901 25.4493 -48.9802

H -4.0449 25.0423 -49.2262

C -5.5049 25.5903 -41.2992

H -5.4039 24.6713 -40.7042

H -5.5799 26.4433 -40.6052

C 1.6701 24.4753 -40.9782

H 1.2901 23.5343 -41.4062

H 1.8351 25.1893 -41.7952

C -0.8479 25.6623 -8.4002

C -1.3109 26.0793 -14.0302

C -1.4659 26.0853 -15.2292

C -1.7059 26.0853 -16.6152

C -0.6439 25.9803 -17.5372

C -3.0239 26.1423 -17.0762

H -3.8079 26.1843 -16.3372

O -1.8439 25.6533 -7.6872

O 0.3731 25.5563 -7.9182

H 0.3771 25.4843 -6.9192

C -1.8809 25.8493 -27.5882

C -2.4099 26.1883 -21.9542

C -2.4349 26.1663 -20.7442

C -2.2579 26.1233 -19.3492

C -3.3259 26.1283 -18.4302

C -0.9429 26.0333 -18.8882

H -0.1669 25.9883 -19.6352

O -0.7849 25.7913 -28.1292

O -3.0129 25.7993 -28.2492

H -2.8639 25.7363 -29.2422

C -6.8819 25.5123 -18.9232

C -6.7759 25.1763 -20.2612

H -5.7949 25.0143 -20.6742

C -7.9219 25.0793 -21.0392

C -9.1869 25.2833 -20.4632

C -9.2849 25.5763 -19.0952

C -8.1249 25.6923 -18.3382

H -8.1789 25.9393 -17.2892

C -6.7509 24.5253 -23.0482

H -5.9579 25.2313 -22.7702

H -6.4209 23.5113 -22.7752

C -7.0029 24.6133 -24.5532

H -7.2349 25.6473 -24.8132

H -6.0719 24.3393 -25.0542

C -8.1459 23.7073 -25.0182

H -8.1539 22.8043 -24.4072

H -7.9739 23.4123 -26.0572

C -9.5129 24.3803 -24.9082

H -9.6249 24.7943 -23.9072

H -9.5699 25.2043 -25.6252

C -10.6509 23.3963 -25.1652

H -10.5949 22.5893 -24.4322

H -10.5429 22.9543 -26.1602

C -12.0139 24.0743 -25.0532

H -12.0679 24.5943 -24.0962

H -12.1199 24.8253 -25.8412

C -13.1579 23.0683 -25.1422

H -13.0299 22.3303 -24.3472

H -13.1039 22.5373 -26.0952

C -14.5279 23.7303 -25.0092

H -14.5719 24.2803 -24.0672

H -14.6629 24.4543 -25.8172

C -15.6609 22.7083 -25.0502

H -15.5259 21.9973 -24.2312

H -15.6059 22.1443 -25.9842

C -17.0389 23.3533 -24.9302

H -17.1819 24.0593 -25.7532

H -17.0929 23.9213 -23.9992

C -18.1579 22.3163 -24.9552

H -18.0959 21.7403 -25.8812

H -18.0169 21.6193 -24.1252

C -19.5369 22.9533 -24.8472

H -19.7019 23.6513 -25.6652

H -20.3159 22.1933 -24.8842

H -19.6339 23.4983 -23.9112

C -11.3019 24.3233 -21.0232

H -11.2439 23.5223 -21.7722

H -11.1639 23.8753 -20.0372

C -12.6609 25.0143 -21.1022

H -12.7439 25.7263 -20.2792

H -12.7119 25.5803 -22.0342

C -13.7849 23.9873 -21.0462

H -13.6509 23.3673 -20.1562

H -13.7009 23.3283 -21.9112

C -15.1759 24.6143 -21.0172

H -15.2699 25.2633 -20.1432

H -15.3169 25.2373 -21.9032

C -16.2569 23.5363 -20.9752

H -16.0799 22.8903 -20.1102

H -16.1719 22.9133 -21.8672

C -17.6719 24.1003 -20.8882

H -17.7719 24.7023 -19.9822

H -17.8569 24.7583 -21.7412

C -18.7099 22.9813 -20.8752

H -18.5139 22.3213 -20.0252

H -18.6009 22.3833 -21.7822

C -20.1439 23.4943 -20.7822

H -20.2719 24.0543 -19.8532

H -20.3389 24.1823 -21.6092

C -21.1519 22.3493 -20.8302

H -20.9649 21.6733 -19.9912

H -21.0029 21.7793 -21.7512

C -22.5969 22.8343 -20.7682

H -22.7509 23.3993 -19.8462

H -22.7879 23.5083 -21.6062

C -23.5889 21.6743 -20.8192

H -23.4139 21.0143 -19.9662

H -23.4149 21.0943 -21.7292

C -25.0349 22.1523 -20.7932

H -25.2389 22.7993 -21.6432

H -25.7179 21.3073 -20.8342

H -25.2359 22.7103 -19.8812

C -10.6949 26.2023 -17.2692

H -10.3779 25.4163 -16.5672

H -10.0789 27.0953 -17.0982

C -12.1629 26.5483 -17.0422

H -12.2559 27.0163 -16.0602

H -12.4649 27.2763 -17.7972

C -13.0719 25.3253 -17.1222

H -12.9549 24.8623 -18.1022

H -12.7639 24.5963 -16.3692

C -14.5349 25.6993 -16.8982

H -14.8279 26.4593 -17.6262

H -14.6459 26.1383 -15.9032

C -15.4659 24.4963 -17.0262

H -15.1589 23.7233 -16.3192

H -15.3759 24.0773 -18.0292

C -16.9239 24.8663 -16.7682

H -17.2349 25.6283 -17.4872

H -17.0179 25.3023 -15.7702

C -17.8469 23.6563 -16.8822

H -17.7249 23.2033 -17.8682

H -17.5559 22.9103 -16.1402

C -19.3139 24.0243 -16.6802

H -19.6089 24.7553 -17.4372

H -19.4389 24.4983 -15.7032

C -20.2239 22.8033 -16.7742

H -19.9509 22.0903 -15.9922

H -20.0679 22.3133 -17.7372

C -21.6999 23.1643 -16.6282

H -21.8639 23.6483 -15.6632

H -21.9739 23.8803 -17.4062

C -22.5999 21.9363 -16.7372

H -22.3419 21.2293 -15.9432

H -22.4169 21.4423 -17.6932

C -24.0759 22.2963 -16.6312

H -24.3529 22.9983 -17.4152

H -24.6959 21.4093 -16.7312

H -24.2889 22.7593 -15.6692

O -10.2669 25.2593 -21.3022

O -7.9469 24.8273 -22.3702

O -10.5439 25.7563 -18.6002

O -4.5799 26.1133 -18.9402

C 2.9031 25.1913 -17.2382

C 2.9191 24.4683 -16.0592

H 1.9851 24.1073 -15.6652

C 4.1201 24.2583 -15.3902

C 5.3201 24.7483 -15.9322

C 5.2981 25.4363 -17.1552

C 4.0811 25.6703 -17.7832

H 4.0381 26.2273 -18.7052

C 3.0451 23.2213 -13.5432

H 2.5171 22.4543 -14.1282

H 2.3851 24.0943 -13.4342

C 3.3871 22.6633 -12.1632

H 3.9621 21.7453 -12.2892

H 2.4381 22.4113 -11.6822

C 4.1701 23.6473 -11.2882

H 3.8331 24.6653 -11.4962

H 3.9621 23.4423 -10.2342

C 5.6761 23.5493 -11.5252

H 5.8761 23.6393 -12.5922

H 6.0161 22.5613 -11.2042

C 6.4731 24.6103 -10.7702

H 6.2411 25.5983 -11.1762

H 6.1821 24.6153 -9.7152

C 7.9721 24.3453 -10.8912

H 8.2161 24.1833 -11.9432

H 8.2161 23.4273 -10.3492

C 8.8301 25.4843 -10.3492

H 8.6291 26.3933 -10.9202

H 8.5581 25.6863 -9.3102

C 10.3181 25.1503 -10.4332

H 10.5701 24.9013 -11.4672

H 10.5241 24.2693 -9.8212

C 11.2021 26.3013 -9.9662

H 11.0251 27.1753 -10.5982

H 10.9261 26.5783 -8.9452

C 12.6841 25.9363 -10.0102

H 12.8611 25.0683 -9.3712

H 12.9551 25.6563 -11.0312

C 13.5771 27.0853 -9.5522

H 13.2891 27.3853 -8.5412

H 13.4191 27.9483 -10.2042

C 15.0511 26.6983 -9.5682

H 15.2311 25.8553 -8.9052

H 15.6711 27.5303 -9.2422

H 15.3581 26.4163 -10.5722

C 7.1801 25.6673 -14.7842

H 6.8271 25.8993 -13.7692

H 6.9731 26.5283 -15.4262

C 8.6741 25.3733 -14.7772

H 8.9651 25.0923 -15.7902

H 8.8561 24.5163 -14.1272

C 9.5161 26.5553 -14.3052

H 9.3241 27.4233 -14.9402

H 9.2341 26.8233 -13.2852

C 11.0031 26.2093 -14.3502

H 11.2721 25.9293 -15.3712

H 11.1851 25.3403 -13.7132

C 11.9031 27.3533 -13.8912

H 11.7391 28.2283 -14.5242

H 11.6441 27.6323 -12.8682

C 13.3751 26.9513 -13.9492

H 13.6291 26.6693 -14.9742

H 13.5291 26.0703 -13.3212

C 14.3131 28.0613 -13.4862

H 14.1831 28.9403 -14.1222

H 14.0571 28.3513 -12.4662

C 15.7721 27.6133 -13.5312

H 16.0251 27.3213 -14.5542

H 15.8971 26.7313 -12.8992

C 16.7351 28.7023 -13.0672

H 16.6251 29.5803 -13.7092

H 16.4791 29.0023 -12.0492

C 18.1861 28.2273 -13.1052

H 18.4241 27.8773 -14.1142

H 18.3061 27.3783 -12.4282

C 19.1691 29.3263 -12.7122

H 19.0321 30.1863 -13.3712

H 18.9551 29.6533 -11.6922

C 20.6131 28.8473 -12.8002

H 20.7741 27.9983 -12.1402

H 21.2991 29.6403 -12.5132

H 20.8501 28.5403 -13.8172

C 6.5031 26.5683 -18.8712

H 6.0061 27.5383 -18.7312

H 5.9651 26.0063 -19.6482

C 7.9431 26.7763 -19.3222

H 7.9201 27.3123 -20.2722

H 8.3981 25.7993 -19.4962

C 8.7861 27.5503 -18.3132

H 8.8571 26.9723 -17.3912

H 8.2971 28.4993 -18.0762

C 10.1821 27.8173 -18.8672

H 10.6141 26.8743 -19.2102

H 10.1041 28.4743 -19.7362

C 11.1231 28.4463 -17.8432

H 10.6981 29.3833 -17.4782

H 11.2271 27.7743 -16.9892

C 12.5001 28.7093 -18.4492

H 12.8761 27.7823 -18.8882

H 12.4051 29.4393 -19.2572

C 13.5101 29.2163 -17.4252

H 13.6051 28.4823 -16.6222

H 13.1481 30.1463 -16.9812

C 14.8821 29.4503 -18.0542

H 15.2061 28.5353 -18.5562

H 14.8061 30.2293 -18.8162

C 15.9311 29.8493 -17.0192

H 15.6311 30.7823 -16.5362

H 15.9801 29.0793 -16.2462

C 17.3151 30.0223 -17.6402

H 17.2871 30.8293 -18.3762

H 17.5911 29.1053 -18.1692

C 18.3781 30.3293 -16.5892

H 18.1151 31.2533 -16.0682

H 18.3911 29.5253 -15.8512

C 19.7661 30.4693 -17.2022

H 20.0521 29.5513 -17.7102

H 20.5051 30.6813 -16.4322

H 19.7841 31.2803 -17.9272

O 6.4931 24.5113 -15.2572

O 4.2301 23.6023 -14.2082

O 6.4971 25.8473 -17.6552

O 0.6061 25.8213 -17.0382

C -1.1719 26.0163 -12.6312

C 0.0841 25.8353 -12.0412

C -2.3059 26.1063 -11.8142

C 0.2021 25.7263 -10.6732

H 0.9571 25.7833 -12.6752

C -2.1859 25.9933 -10.4492

H -3.2739 26.2533 -12.2692

C -0.9329 25.7983 -9.8642

H 1.1701 25.5833 -10.2172

H -3.0539 26.0463 -9.8092

C -2.3099 26.1663 -23.3582

C -1.0459 26.0063 -23.9392

C -3.4309 26.2793 -24.1852

C -0.9139 25.9233 -25.3052

H -0.1799 25.9373 -23.2972

C -3.2979 26.1833 -25.5532

H -4.4019 26.4393 -23.7432

C -2.0399 25.9893 -26.1262

H 0.0541 25.7943 -25.7642

H -4.1619 26.2563 -26.1942

C -5.6359 25.6993 -18.0962

H -5.3599 24.7533 -17.6062

H -5.8259 26.4523 -17.3182

C 1.6101 25.4443 -17.9592

H 1.2921 24.5293 -18.4832

H 1.7631 26.2383 -18.7022

C -2.1019 23.0003 -20.1552

C -2.0219 22.7103 -25.7992

C -2.0029 22.6393 -27.0052

C -2.0959 22.5863 -28.4052

C -0.9409 22.5933 -29.2122

C -3.3649 22.5323 -28.9802

H -4.2009 22.5023 -28.3022

O -3.1469 23.0663 -19.5232

O -0.9199 22.9853 -19.5822

H -1.0049 23.0223 -18.5772

C -2.4029 22.5873 -39.4252

C -2.4429 22.6613 -33.7742

C -2.4729 22.6223 -32.5652

C -2.3859 22.5853 -31.1642

C -3.5399 22.5203 -30.3552

C -1.1129 22.6043 -30.5862

H -0.2689 22.6353 -31.2562

O -1.3529 22.4963 -40.0532

O -3.5769 22.6603 -40.0062

H -3.5089 22.6093 -41.0162

C -7.1639 22.2693 -30.9042

C -7.2299 22.1983 -32.2832

H -6.3169 22.0753 -32.8422

C -8.4639 22.3193 -32.9152

C -9.6329 22.4983 -32.1612

C -9.5579 22.5453 -30.7622

C -8.3189 22.4183 -30.1492

H -8.2369 22.4573 -29.0742

C -7.6419 21.8983 -35.1222

H -6.7339 22.5063 -34.9922

H -7.3929 20.8503 -34.9042

C -8.1429 22.0353 -36.5592

H -8.3539 23.0873 -36.7572

H -7.3279 21.7223 -37.2172

C -9.3959 21.2003 -36.8412

H -9.3309 20.2603 -36.2922

H -9.4279 20.9673 -37.9112

C -10.6969 21.9053 -36.4572

H -10.6439 22.2143 -35.4152

H -10.8189 22.8053 -37.0672

C -11.8979 20.9833 -36.6602

H -11.7739 20.1093 -36.0182

H -11.9129 20.6303 -37.6952

C -13.2259 21.6683 -36.3442

H -13.2129 22.0143 -35.3102

H -13.3439 22.5483 -36.9812

C -14.4159 20.7343 -36.5542

H -14.2979 19.8523 -35.9202

H -14.4229 20.3813 -37.5882

C -15.7569 21.3963 -36.2452

H -15.7729 21.7123 -35.2002

H -15.8729 22.2913 -36.8612

C -16.9239 20.4473 -36.5062

H -16.7929 19.5423 -35.9072

H -16.9119 20.1393 -37.5552

C -18.2779 21.0713 -36.1812

H -18.4209 21.9683 -36.7882

H -18.2939 21.3763 -35.1332

C -19.4269 20.0993 -36.4382

H -19.4059 19.7833 -37.4842

H -19.2829 19.2033 -35.8302

C -20.7839 20.7133 -36.1212

H -20.9559 21.5953 -36.7332

H -21.5829 20.0003 -36.3152

H -20.8339 21.0073 -35.0752

C -11.8579 21.7783 -32.5952

H -11.9149 21.0963 -33.4522

H -11.6459 21.1813 -31.7052

C -13.1689 22.5433 -32.4282

H -13.0999 23.1663 -31.5362

H -13.2909 23.2083 -33.2862

C -14.3599 21.5973 -32.3272

H -14.2329 20.9523 -31.4532

H -14.3809 20.9513 -33.2072

C -15.6919 22.3353 -32.2162

H -15.6879 22.9663 -31.3252

H -15.8149 22.9913 -33.0822

C -16.8659 21.3623 -32.1472

H -16.7579 20.7343 -31.2592

H -16.8409 20.7033 -33.0182

C -18.2169 22.0703 -32.0932

H -18.2479 22.7283 -31.2232

H -18.3339 22.6963 -32.9822

C -19.3739 21.0763 -32.0172

H -19.2719 20.4783 -31.1092

H -19.3209 20.3943 -32.8692

C -20.7359 21.7663 -32.0082

H -20.7859 22.4563 -31.1632

H -20.8479 22.3573 -32.9212

C -21.8819 20.7633 -31.9092

H -21.7859 20.2023 -30.9762

H -21.8109 20.0483 -32.7332

C -23.2499 21.4393 -31.9492

H -23.3189 22.1653 -31.1352

H -23.3539 21.9883 -32.8882

C -24.3909 20.4333 -31.8252

H -24.2979 19.9003 -30.8762

H -24.3099 19.6993 -32.6302

C -25.7559 21.1043 -31.8922

H -25.8789 21.6253 -32.8392

H -26.5509 20.3673 -31.8012

H -25.8679 21.8283 -31.0882

C -10.6889 23.0713 -28.7202

H -10.4499 22.1823 -28.1182

H -9.9199 23.8343 -28.5412

C -12.0569 23.6243 -28.3302

H -11.9949 24.0293 -27.3192

H -12.2979 24.4423 -29.0132

C -13.1499 22.5633 -28.4032

H -13.1089 22.0843 -29.3822

H -12.9629 21.7973 -27.6462

C -14.5389 23.1573 -28.1962

H -14.7339 23.8963 -28.9772

H -14.5739 23.6773 -27.2362

C -15.6269 22.0883 -28.2322

H -15.4659 21.3833 -27.4112

H -15.5519 21.5263 -29.1642

C -17.0249 22.6883 -28.1172

H -17.1969 23.3623 -28.9592

H -17.0879 23.2823 -27.2032

C -18.1109 21.6173 -28.0972

H -18.0319 21.0023 -28.9962

H -17.9529 20.9633 -27.2362

C -19.5099 22.2223 -28.0212

H -19.6879 22.8283 -28.9132

H -19.5699 22.8853 -27.1562

C -20.5939 21.1543 -27.9102

H -20.4359 20.5793 -26.9942

H -20.5099 20.4663 -28.7552

C -21.9969 21.7553 -27.8902

H -22.0869 22.4353 -27.0392

H -22.1489 22.3423 -28.7992

C -23.0819 20.6853 -27.8002

H -22.9439 20.1063 -26.8832

H -22.9799 19.9993 -28.6442

C -24.4799 21.2893 -27.8112

H -24.6399 21.8593 -28.7232

H -25.2369 20.5093 -27.7582

H -24.6149 21.9573 -26.9622

O -10.8019 22.6993 -32.8432

O -8.6609 22.3333 -34.2562

O -10.7289 22.7343 -30.0922

O -4.7409 22.4423 -30.9782

C 2.6341 22.5883 -28.4362

C 2.6111 23.2683 -27.2292

H 1.7011 23.7683 -26.9412

C 3.7501 23.3023 -26.4282

C 4.9191 22.6673 -26.8672

C 4.9361 21.9893 -28.0912

C 3.7821 21.9403 -28.8642

H 3.7741 21.4063 -29.8012

C 2.7121 24.5843 -24.7062

H 1.8421 23.9153 -24.7452

H 2.4881 25.4813 -25.3022

C 2.9761 24.9703 -23.2532

H 3.1681 24.0603 -22.6812

H 2.0571 25.4213 -22.8742

C 4.1491 25.9403 -23.0742

H 4.2511 26.5533 -23.9722

H 3.9341 26.6153 -22.2422

C 5.4731 25.2293 -22.7942

H 5.6661 24.5003 -23.5812

H 5.3901 24.6853 -21.8492

C 6.6311 26.2223 -22.7002

H 6.8041 26.6663 -23.6832

H 6.3541 27.0333 -22.0202

C 7.9241 25.5803 -22.2012

H 8.1831 24.7383 -22.8452

H 7.7651 25.1893 -21.1932

C 9.0801 26.5783 -22.1822

H 9.2801 26.9133 -23.2022

H 8.7891 27.4583 -21.6022

C 10.3581 25.9923 -21.5882

H 10.6581 25.1153 -22.1652

H 10.1631 25.6653 -20.5642

C 11.4981 27.0083 -21.5862

H 11.6881 27.3343 -22.6112

H 11.1971 27.8873 -21.0112

C 12.7871 26.4433 -20.9952

H 12.6031 26.1203 -19.9682

H 13.0941 25.5643 -21.5672

C 13.9181 27.4693 -21.0092

H 13.6191 28.3443 -20.4282

H 14.0921 27.7973 -22.0352

C 15.2091 26.9013 -20.4332

H 15.0591 26.5903 -19.4022

H 16.0011 27.6463 -20.4562

H 15.5351 26.0343 -21.0052

C 6.8701 23.8673 -26.4002

H 6.3981 24.7663 -25.9792

H 6.9511 24.0003 -27.4882

C 8.2491 23.6533 -25.7942

H 8.6871 22.7583 -26.2392

H 8.1271 23.4703 -24.7252

C 9.1721 24.8483 -26.0102

H 9.2581 25.0563 -27.0792

H 8.7381 25.7313 -25.5362

C 10.5601 24.5903 -25.4282

H 11.0041 23.7253 -25.9252

H 10.4631 24.3473 -24.3682

C 11.4921 25.7893 -25.5832

H 11.5781 26.0483 -26.6412

H 11.0621 26.6513 -25.0682

C 12.8801 25.5023 -25.0162

H 13.3241 24.6613 -25.5542

H 12.7851 25.2093 -23.9682

C 13.8111 26.7083 -25.1162

H 13.8841 27.0243 -26.1602

H 13.3851 27.5423 -24.5522

C 15.2061 26.3933 -24.5822

H 15.6411 25.5823 -25.1692

H 15.1261 26.0463 -23.5502

C 16.1331 27.6043 -24.6342

H 16.1891 27.9723 -25.6622

H 15.7181 28.4093 -24.0222

C 17.5381 27.2683 -24.1402

H 17.9401 26.4423 -24.7312

H 17.4851 26.9363 -23.1002

C 18.4891 28.4573 -24.2412

H 18.5251 28.8023 -25.2772

H 18.1071 29.2803 -23.6322

C 19.8951 28.0943 -23.7812

H 19.8841 27.7593 -22.7462

H 20.5601 28.9513 -23.8572

H 20.3001 27.2913 -24.3942

C 6.3211 21.0063 -29.7662

H 6.0021 21.7913 -30.4662

H 5.7351 20.0993 -29.9702

C 7.8101 20.7263 -29.9452

H 7.9821 20.4493 -30.9842

H 8.0901 19.8843 -29.3112

C 8.6551 21.9433 -29.5762

H 8.4121 22.2313 -28.5532

H 8.3941 22.7763 -30.2332

C 10.1531 21.6733 -29.6762

H 10.4151 20.8293 -29.0342

H 10.4091 21.3973 -30.7022

C 10.9591 22.8993 -29.2572

H 10.7031 23.7363 -29.9112

H 10.6751 23.1793 -28.2402

C 12.4661 22.6693 -29.3082

H 12.7311 21.8473 -28.6382

H 12.7551 22.3753 -30.3192

C 13.2371 23.9223 -28.9032

H 12.9241 24.2263 -27.9022

H 12.9841 24.7373 -29.5862

C 14.7481 23.7093 -28.9132

H 15.0031 22.9133 -28.2102

H 15.0621 23.3823 -29.9062

C 15.5051 24.9783 -28.5342

H 15.2771 25.7683 -29.2552

H 15.1631 25.3203 -27.5552

C 17.0151 24.7593 -28.4872

H 17.3661 24.4293 -29.4682

H 17.2391 23.9633 -27.7742

C 17.7681 26.0223 -28.0782

H 17.5991 26.8023 -28.8242

H 17.3721 26.3833 -27.1282

C 19.2651 25.7723 -27.9352

H 19.4541 25.0163 -27.1762

H 19.7801 26.6853 -27.6452

H 19.6861 25.4233 -28.8762

O 6.0721 22.7273 -26.1102

O 3.8491 23.9353 -25.2312

O 6.1231 21.4373 -28.4362

O 0.2461 22.5993 -28.5622

C -2.0329 22.7823 -24.3972

C -0.8369 22.7153 -23.6762

C -3.2459 22.9183 -23.7142

C -0.8519 22.7863 -22.3032

H 0.0921 22.5993 -24.2152

C -3.2569 22.9863 -22.3402

H -4.1659 22.9643 -24.2762

C -2.0619 22.9243 -21.6252

H 0.0671 22.7333 -21.7402

H -4.1789 23.0863 -21.7912

C -2.4329 22.6703 -35.1792

C -1.2439 22.4343 -35.8772

C -3.6159 22.8983 -35.8892

C -1.2429 22.4143 -37.2512

H -0.3329 22.2653 -35.3222

C -3.6139 22.8733 -37.2632

H -4.5259 23.0893 -35.3412

C -2.4279 22.6283 -37.9562

H -0.3369 22.2243 -37.8052

H -4.5249 23.0383 -37.8172

C -5.8639 22.2183 -30.1472

H -5.7579 21.2373 -29.6582

H -5.9079 22.9913 -29.3682

C 1.4251 22.5993 -29.3352

H 1.4461 21.7203 -29.9942

H 1.4561 23.5063 -29.9612

C -2.3559 22.4203 -42.6992

C -2.2699 22.0383 -48.3372

C -2.2469 21.9453 -49.5422

C -2.3169 21.8843 -50.9432

C -1.1479 21.9323 -51.7282

C -3.5729 21.7893 -51.5422

H -4.4239 21.7253 -50.8842

O -3.4059 22.5133 -42.0782

O -1.1789 22.3933 -42.1172

H -1.2679 22.4613 -41.1142

C -2.4679 22.1583 -61.9672

C -2.5529 21.9943 -56.3162

C -2.6159 21.9413 -55.1102

C -2.5549 21.8933 -53.7072

C -3.7199 21.7843 -52.9202

C -1.2939 21.9473 -53.1052

H -0.4409 22.0133 -53.7612

O -1.4189 22.2223 -62.6022

O -3.6479 22.1483 -62.5422

H -3.5949 22.2193 -63.5422

C -7.3009 21.5113 -53.6462

C -7.2579 21.3533 -55.0192

H -6.3139 21.1213 -55.4822

C -8.4129 21.5393 -55.7722

C -9.6199 21.8653 -55.1362

C -9.6659 21.9733 -53.7382

C -8.4989 21.7943 -53.0062

H -8.5069 21.8953 -51.9322

C -7.4309 20.8733 -57.8392

H -6.4729 21.3593 -57.6092

H -7.3619 19.8143 -57.5522

C -7.7039 20.9953 -59.3372

H -7.7709 22.0523 -59.6012

H -6.8369 20.5723 -59.8532

C -8.9819 20.2743 -59.7722

H -9.0929 19.3623 -59.1822

H -8.8899 19.9893 -60.8252

C -10.2379 21.1273 -59.6042

H -10.2649 21.5283 -58.5912

H -10.2009 21.9703 -60.3012

C -11.4959 20.3013 -59.8582

H -11.5119 19.4763 -59.1432

H -11.4539 19.8653 -60.8602

C -12.7789 21.1173 -59.7222

H -12.7809 21.6303 -58.7592

H -12.8079 21.8843 -60.5022

C -14.0189 20.2333 -59.8322

H -13.9999 19.4953 -59.0262

H -13.9839 19.6783 -60.7732

C -15.3279 21.0153 -59.7612

H -15.3819 21.5453 -58.8082

H -15.3509 21.7643 -60.5562

C -16.5349 20.0893 -59.8972

H -16.4909 19.3303 -59.1122

H -16.4799 19.5643 -60.8552

C -17.8659 20.8293 -59.8032

H -17.9249 21.5733 -60.6002

H -17.9179 21.3593 -58.8502

C -19.0509 19.8733 -59.9142

H -18.9969 19.3413 -60.8662

H -18.9839 19.1273 -59.1192

C -20.3889 20.5953 -59.8122

H -20.4819 21.3383 -60.6002

H -21.2129 19.8913 -59.9082

H -20.4779 21.1003 -58.8532

C -11.8149 21.2513 -55.7992

H -11.8209 20.5983 -56.6812

H -11.7019 20.6203 -54.9142

C -13.1099 22.0543 -55.7202

H -13.0899 22.6623 -54.8152

H -13.1509 22.7333 -56.5742

C -14.3289 21.1383 -55.7202

H -14.2679 20.4623 -54.8642

H -14.3139 20.5243 -56.6222

C -15.6489 21.9033 -55.6562

H -15.6809 22.5033 -54.7442

H -15.7109 22.5903 -56.5042

C -16.8419 20.9513 -55.6842

H -16.7819 20.2803 -54.8232

H -16.7859 20.3353 -56.5842

C -18.1839 21.6763 -55.6562

H -18.2509 22.2843 -54.7512

H -18.2509 22.3523 -56.5122

C -19.3509 20.6923 -55.6952

H -19.2959 20.0383 -54.8212

H -19.2599 20.0633 -56.5832

C -20.7079 21.3893 -55.7102

H -20.8099 22.0063 -54.8152

H -20.7639 22.0543 -56.5752

C -21.8579 20.3873 -55.7682

H -21.8239 19.7523 -54.8792

H -21.7309 19.7413 -56.6402

C -23.2209 21.0703 -55.8442

H -23.3459 21.7273 -54.9812

H -23.2619 21.6923 -56.7412

C -24.3659 20.0603 -55.8812

H -24.3429 19.4583 -54.9702

H -24.2239 19.3883 -56.7292

C -25.7239 20.7393 -55.9972

H -25.7779 21.3323 -56.9082

H -26.5219 20.0003 -56.0222

H -25.8969 21.4003 -55.1502

C -10.9429 22.6323 -51.8262

H -10.7599 21.7543 -51.1892

H -10.1829 23.3933 -51.6052

C -12.3319 23.2033 -51.5522

H -12.3339 23.6533 -50.5582

H -12.5259 23.9903 -52.2832

C -13.4259 22.1423 -51.6452

H -13.3429 21.6343 -52.6062

H -13.2759 21.3973 -50.8592

C -14.8219 22.7443 -51.5132

H -14.9789 23.4683 -52.3162

H -14.8989 23.2823 -50.5662

C -15.9099 21.6753 -51.5802

H -15.7939 20.9883 -50.7382

H -15.7899 21.0943 -52.4962

C -17.3139 22.2743 -51.5472

H -17.4339 22.9533 -52.3952

H -17.4359 22.8633 -50.6352

C -18.3959 21.2003 -51.6042

H -18.2499 20.5883 -52.4972

H -18.3019 20.5443 -50.7362

C -19.7999 21.7973 -51.6272

H -19.9019 22.4453 -52.5012

H -19.9449 22.4173 -50.7402

C -20.8799 20.7193 -51.6712

H -20.7899 20.0833 -50.7862

H -20.7249 20.0893 -52.5492

C -22.2859 21.3123 -51.7202

H -22.4499 21.9293 -50.8332

H -22.3709 21.9623 -52.5942

C -23.3619 20.2333 -51.7922

H -23.2819 19.5853 -50.9162

H -23.1899 19.6163 -52.6772

C -24.7629 20.8263 -51.8572

H -24.8629 21.4693 -52.7282

H -25.5109 20.0403 -51.9252

H -24.9689 21.4213 -50.9692

O -10.7079 22.1363 -55.9222

O -8.4809 21.4823 -57.1252

O -10.8779 22.2683 -53.1912

O -4.9009 21.6823 -53.5762

C 2.4291 21.9713 -50.9502

C 2.4341 22.6583 -49.7472

H 1.5351 23.1693 -49.4452

C 3.5911 22.6873 -48.9722

C 4.7471 22.0433 -49.4302

C 4.7371 21.3663 -50.6562

C 3.5651 21.3173 -51.4002

H 3.5351 20.7803 -52.3352

C 2.6321 24.0353 -47.2492

H 1.7281 23.4123 -47.2582

H 2.4441 24.9293 -47.8602

C 2.9451 24.4313 -45.8082

H 3.1431 23.5243 -45.2362

H 2.0441 24.8983 -45.4062

C 4.1331 25.3893 -45.6712

H 4.1801 26.0343 -46.5512

H 3.9751 26.0343 -44.8032

C 5.4671 24.6623 -45.5022

H 5.6031 23.9613 -46.3242

H 5.4411 24.0833 -44.5762

C 6.6371 25.6433 -45.4472

H 6.7311 26.1493 -46.4112

H 6.4261 26.4113 -44.6972

C 7.9591 24.9613 -45.1032

H 8.1671 24.1823 -45.8372

H 7.8691 24.4773 -44.1282

C 9.1201 25.9523 -45.0692

H 9.2511 26.3893 -46.0622

H 8.8791 26.7703 -44.3852

C 10.4301 25.3043 -44.6262

H 10.6811 24.4913 -45.3102

H 10.2971 24.8733 -43.6322

C 11.5811 26.3073 -44.5902

H 11.7111 26.7433 -45.5832

H 11.3301 27.1213 -43.9062

C 12.8951 25.6703 -44.1462

H 12.7661 25.2323 -43.1532

H 13.1541 24.8603 -44.8322

C 14.0391 26.6793 -44.1062

H 13.7851 27.4883 -43.4182

H 14.1681 27.1183 -45.0982

C 15.3481 26.0363 -43.6652

H 15.2401 25.6013 -42.6742

H 16.1471 26.7733 -43.6322

H 15.6381 25.2473 -44.3552

C 6.7781 23.1523 -49.0552

H 6.4381 24.0883 -48.5862

H 6.7621 23.2943 -50.1432

C 8.1851 22.8043 -48.5902

H 8.5251 21.9223 -49.1352

H 8.1361 22.5383 -47.5332

C 9.1691 23.9533 -48.7852

H 9.1821 24.2523 -49.8372

H 8.8381 24.8173 -48.2042

C 10.5751 23.5553 -48.3472

H 10.9311 22.7313 -48.9702

H 10.5371 23.1923 -47.3172

C 11.5661 24.7123 -48.4252

H 11.5801 25.1133 -49.4422

H 11.2371 25.5143 -47.7612

C 12.9731 24.2733 -48.0302

H 13.3201 23.5013 -48.7202

H 12.9431 23.8313 -47.0312

C 13.9661 25.4323 -48.0292

H 13.9681 25.9093 -49.0122

H 13.6461 26.1813 -47.3022

C 15.3791 24.9653 -47.6902

H 15.7061 24.2383 -48.4372

H 15.3681 24.4583 -46.7222

C 16.3761 26.1193 -47.6432

H 16.3431 26.6613 -48.5922

H 16.0871 26.8203 -46.8572

C 17.7991 25.6313 -47.3902

H 18.0691 24.9033 -48.1592

H 17.8411 25.1233 -46.4242

C 18.8161 26.7693 -47.4042

H 18.7641 27.2863 -48.3662

H 18.5621 27.4923 -46.6262

C 20.2351 26.2593 -47.1832

H 20.3121 25.7503 -46.2242

H 20.9451 27.0833 -47.1922

H 20.5131 25.5583 -47.9662

C 6.0611 20.2983 -52.3352

H 5.7251 21.0323 -53.0812

H 5.4601 19.3843 -52.4482

C 7.5421 19.9913 -52.5342

H 7.6781 19.5443 -53.5182

H 7.8491 19.2653 -51.7802

C 8.3961 21.2493 -52.4022

H 8.1351 21.7463 -51.4682

H 8.1661 21.9323 -53.2232

C 9.8901 20.9413 -52.3992

H 10.1131 20.2493 -51.5842

H 10.1671 20.4463 -53.3332

C 10.7191 22.2103 -52.2242

H 10.5361 22.8783 -53.0682

H 10.3941 22.7263 -51.3192

C 12.2141 21.9223 -52.1242

H 12.3991 21.2753 -51.2642

H 12.5421 21.3833 -53.0152

C 13.0331 23.2023 -51.9772

H 12.6861 23.7543 -51.1012

H 12.8681 23.8373 -52.8512

C 14.5241 22.9153 -51.8332

H 14.6861 22.3163 -50.9332

H 14.8641 22.3233 -52.6852

C 15.3581 24.1903 -51.7422

H 15.2321 24.7733 -52.6582

H 14.9981 24.7993 -50.9112

C 16.8391 23.8843 -51.5392

H 17.1971 23.2683 -52.3672

H 16.9601 23.3053 -50.6202

C 17.6921 25.1463 -51.4452

H 17.5811 25.7273 -52.3642

H 17.3321 25.7633 -50.6192

C 19.1641 24.8183 -51.2272

H 19.2971 24.2473 -50.3102

H 19.7541 25.7283 -51.1512

H 19.5491 24.2263 -52.0552

O 5.9071 22.0913 -48.6852

O 3.7261 23.3213 -47.7792

O 5.9191 20.8243 -51.0332

O 0.0311 21.9693 -51.0632

C -2.2849 22.1343 -46.9362

C -1.0849 22.2223 -46.2252

C -3.5019 22.1453 -46.2462

C -1.0989 22.3103 -44.8522

H -0.1529 22.2173 -46.7702

C -3.5119 22.2373 -44.8742

H -4.4259 22.0823 -46.8012

C -2.3129 22.3183 -44.1672

H -0.1779 22.3773 -44.2962

H -4.4359 22.2463 -44.3172

C -2.5269 22.0363 -57.7202

C -1.3219 21.8873 -58.4162

C -3.7139 22.2243 -58.4352

C -1.3089 21.9293 -59.7902

H -0.4059 21.7473 -57.8612

C -3.7029 22.2433 -59.8082

H -4.6349 22.3593 -57.8882

C -2.4989 22.1013 -60.4992

H -0.3879 21.8193 -60.3402

H -4.6199 22.3723 -60.3602

C -6.0549 21.4053 -52.8052

H -5.9669 20.3943 -52.3782

H -6.1419 22.1243 -51.9812

C 1.2121 21.9853 -51.8362

H 1.2361 21.1133 -52.5032

H 1.2351 22.8993 -52.4522

C -1.8059 24.8643 -55.3142

C -2.2689 25.2813 -60.9432

C -2.4239 25.2883 -62.1432

C -2.6639 25.2883 -63.5292

C -1.6019 25.1823 -64.4512

C -3.9819 25.3453 -63.9902

H -4.7659 25.3863 -63.2502

O -2.8019 24.8563 -54.6012

O -0.5849 24.7583 -54.8322

H -0.5809 24.6873 -53.8332

C -2.8389 25.0513 -74.5022

C -3.3679 25.3903 -68.8682

C -3.3929 25.3683 -67.6582

C -3.2159 25.3253 -66.2632

C -4.2839 25.3303 -65.3432

C -1.9009 25.2353 -65.8022

H -1.1249 25.1903 -66.5492

O -1.7429 24.9943 -75.0432

O -3.9709 25.0023 -75.1632

H -3.8219 24.9393 -76.1562

C -7.8399 24.7153 -65.8372

C -7.7339 24.3783 -67.1752

H -6.7529 24.2163 -67.5882

C -8.8799 24.2813 -67.9522

C -10.1449 24.4853 -67.3772

C -10.2429 24.7793 -66.0092

C -9.0829 24.8953 -65.2512

H -9.1369 25.1413 -64.2032

C -7.7089 23.7283 -69.9622

H -6.9159 24.4333 -69.6842

H -7.3789 22.7143 -69.6892

C -7.9609 23.8153 -71.4672

H -8.1929 24.8493 -71.7262

H -7.0289 23.5413 -71.9682

C -9.1039 22.9093 -71.9322

H -9.1119 22.0073 -71.3212

H -8.9309 22.6143 -72.9712

C -10.4709 23.5823 -71.8222

H -10.5829 23.9973 -70.8212

H -10.5279 24.4063 -72.5392

C -11.6089 22.5993 -72.0792

H -11.5529 21.7913 -71.3452

H -11.5009 22.1563 -73.0742

C -12.9719 23.2763 -71.9672

H -13.0259 23.7963 -71.0092

H -13.0779 24.0273 -72.7552

C -14.1159 22.2703 -72.0562

H -13.9879 21.5333 -71.2612

H -14.0619 21.7393 -73.0092

C -15.4859 22.9323 -71.9232

H -15.5299 23.4823 -70.9812

H -15.6209 23.6563 -72.7312

C -16.6189 21.9103 -71.9642

H -16.4839 21.1993 -71.1452

H -16.5639 21.3463 -72.8972

C -17.9969 22.5563 -71.8442

H -18.1399 23.2613 -72.6672

H -18.0509 23.1243 -70.9132

C -19.1159 21.5183 -71.8692

H -19.0539 20.9433 -72.7952

H -18.9749 20.8213 -71.0392

C -20.4949 22.1553 -71.7602

H -20.6599 22.8533 -72.5792

H -21.2739 21.3963 -71.7982

H -20.5919 22.7003 -70.8252

C -12.2599 23.5263 -67.9372

H -12.2019 22.7253 -68.6862

H -12.1219 23.0773 -66.9512

C -13.6189 24.2173 -68.0162

H -13.7019 24.9293 -67.1932

H -13.6699 24.7823 -68.9482

C -14.7429 23.1893 -67.9592

H -14.6079 22.5693 -67.0702

H -14.6589 22.5303 -68.8252

C -16.1339 23.8163 -67.9312

H -16.2279 24.4653 -67.0572

H -16.2749 24.4403 -68.8172

C -17.2149 22.7383 -67.8882

H -17.0379 22.0923 -67.0242

H -17.1299 22.1153 -68.7812

C -18.6299 23.3023 -67.8022

H -18.7299 23.9053 -66.8962

H -18.8149 23.9603 -68.6552

C -19.6679 22.1833 -67.7892

H -19.4719 21.5243 -66.9392

H -19.5589 21.5853 -68.6962

C -21.1019 22.6973 -67.6962

H -21.2299 23.2563 -66.7672

H -21.2969 23.3843 -68.5232

C -22.1099 21.5513 -67.7442

H -21.9229 20.8753 -66.9052

H -21.9609 20.9813 -68.6642

C -23.5549 22.0363 -67.6812

H -23.7089 22.6023 -66.7602

H -23.7459 22.7103 -68.5202

C -24.5469 20.8763 -67.7332

H -24.3719 20.2163 -66.8802

H -24.3729 20.2973 -68.6432

C -25.9919 21.3543 -67.7062

H -26.1969 22.0013 -68.5572

H -26.6759 20.5093 -67.7482

H -26.1939 21.9123 -66.7952

C -11.6529 25.4043 -64.1832

H -11.3359 24.6193 -63.4812

H -11.0359 26.2973 -64.0122

C -13.1209 25.7503 -63.9562

H -13.2139 26.2183 -62.9732

H -13.4229 26.4783 -64.7112

C -14.0299 24.5273 -64.0362

H -13.9129 24.0643 -65.0162

H -13.7219 23.7983 -63.2832

C -15.4929 24.9013 -63.8122

H -15.7859 25.6623 -64.5402

H -15.6039 25.3413 -62.8172

C -16.4239 23.6983 -63.9402

H -16.1169 22.9253 -63.2332

H -16.3339 23.2793 -64.9432

C -17.8819 24.0683 -63.6822

H -18.1929 24.8313 -64.4002

H -17.9749 24.5043 -62.6842

C -18.8049 22.8583 -63.7962

H -18.6829 22.4053 -64.7812

H -18.5139 22.1123 -63.0542

C -20.2709 23.2263 -63.5942

H -20.5669 23.9573 -64.3512

H -20.3969 23.7003 -62.6172

C -21.1819 22.0053 -63.6872

H -20.9089 21.2923 -62.9062

H -21.0259 21.5153 -64.6512

C -22.6579 22.3663 -63.5422

H -22.8219 22.8513 -62.5762

H -22.9319 23.0833 -64.3202

C -23.5579 21.1383 -63.6502

H -23.2999 20.4323 -62.8572

H -23.3749 20.6453 -64.6072

C -25.0339 21.4993 -63.5452

H -25.3109 22.2003 -64.3292

H -25.6539 20.6113 -63.6452

H -25.2469 21.9613 -62.5832

O -11.2249 24.4623 -68.2162

O -8.9049 24.0293 -69.2842

O -11.5019 24.9583 -65.5142

O -5.5379 25.3153 -65.8542

C 1.9451 24.3933 -64.1522

C 1.9611 23.6703 -62.9722

H 1.0271 23.3093 -62.5792

C 3.1621 23.4603 -62.3042

C 4.3621 23.9503 -62.8452

C 4.3401 24.6383 -64.0682

C 3.1231 24.8733 -64.6972

H 3.0801 25.4293 -65.6192

C 2.0871 22.4233 -60.4572

H 1.5591 21.6563 -61.0422

H 1.4271 23.2963 -60.3482

C 2.4291 21.8653 -59.0772

H 3.0041 20.9473 -59.2032

H 1.4801 21.6133 -58.5962

C 3.2121 22.8503 -58.2022

H 2.8751 23.8683 -58.4102

H 3.0041 22.6443 -57.1482

C 4.7181 22.7523 -58.4392

H 4.9181 22.8413 -59.5062

H 5.0581 21.7643 -58.1182

C 5.5151 23.8123 -57.6842

H 5.2831 24.8003 -58.0902

H 5.2241 23.8173 -56.6292

C 7.0141 23.5473 -57.8052

H 7.2581 23.3853 -58.8562

H 7.2581 22.6303 -57.2632

C 7.8721 24.6863 -57.2632

H 7.6711 25.5953 -57.8342

H 7.6001 24.8883 -56.2242

C 9.3601 24.3523 -57.3472

H 9.6121 24.1033 -58.3812

H 9.5661 23.4713 -56.7352

C 10.2441 25.5043 -56.8802

H 10.0671 26.3773 -57.5112

H 9.9681 25.7813 -55.8592

C 11.7261 25.1383 -56.9242

H 11.9031 24.2703 -56.2852

H 11.9971 24.8583 -57.9452

C 12.6191 26.2883 -56.4662

H 12.3311 26.5873 -55.4552

H 12.4621 27.1503 -57.1182

C 14.0931 25.9013 -56.4822

H 14.2731 25.0573 -55.8192

H 14.7131 26.7323 -56.1562

H 14.4001 25.6183 -57.4862

C 6.2221 24.8693 -61.6982

H 5.8691 25.1013 -60.6832

H 6.0151 25.7313 -62.3402

C 7.7161 24.5753 -61.6912

H 8.0071 24.2943 -62.7042

H 7.8981 23.7183 -61.0412

C 8.5581 25.7583 -61.2192

H 8.3661 26.6263 -61.8542

H 8.2761 26.0253 -60.1992

C 10.0451 25.4123 -61.2642

H 10.3141 25.1313 -62.2852

H 10.2271 24.5423 -60.6272

C 10.9451 26.5553 -60.8052

H 10.7811 27.4303 -61.4382

H 10.6861 26.8343 -59.7822

C 12.4171 26.1533 -60.8632

H 12.6711 25.8723 -61.8882

H 12.5711 25.2723 -60.2352

C 13.3551 27.2633 -60.4002

H 13.2251 28.1423 -61.0362

H 13.0991 27.5533 -59.3792

C 14.8141 26.8163 -60.4452

H 15.0671 26.5243 -61.4682

H 14.9391 25.9333 -59.8132

C 15.7771 27.9043 -59.9812

H 15.6671 28.7823 -60.6232

H 15.5211 28.2053 -58.9632

C 17.2281 27.4293 -60.0192

H 17.4661 27.0803 -61.0272

H 17.3481 26.5803 -59.3412

C 18.2111 28.5283 -59.6262

H 18.0741 29.3883 -60.2852

H 17.9971 28.8553 -58.6062

C 19.6551 28.0503 -59.7142

H 19.8161 27.2003 -59.0542

H 20.3411 28.8433 -59.4272

H 19.8921 27.7423 -60.7312

C 5.5451 25.7703 -65.7852

H 5.0481 26.7403 -65.6452

H 5.0071 25.2083 -66.5622

C 6.9851 25.9783 -66.2362

H 6.9621 26.5153 -67.1862

H 7.4401 25.0023 -66.4102

C 7.8281 26.7533 -65.2272

H 7.8991 26.1753 -64.3052

H 7.3391 27.7013 -64.9902

C 9.2241 27.0193 -65.7812

H 9.6561 26.0763 -66.1242

H 9.1461 27.6763 -66.6502

C 10.1651 27.6483 -64.7572

H 9.7401 28.5853 -64.3922

H 10.2691 26.9763 -63.9032

C 11.5421 27.9113 -65.3632

H 11.9181 26.9843 -65.8022

H 11.4471 28.6413 -66.1712

C 12.5521 28.4183 -64.3392

H 12.6471 27.6843 -63.5352

H 12.1901 29.3483 -63.8952

C 13.9241 28.6523 -64.9672

H 14.2491 27.7373 -65.4692

H 13.8481 29.4323 -65.7292

C 14.9731 29.0513 -63.9332

H 14.6731 29.9843 -63.4502

H 15.0221 28.2823 -63.1592

C 16.3571 29.2243 -64.5542

H 16.3291 30.0313 -65.2902

H 16.6331 28.3083 -65.0832

C 17.4201 29.5313 -63.5032

H 17.1571 30.4553 -62.9822

H 17.4331 28.7273 -62.7652

C 18.8081 29.6723 -64.1162

H 19.0941 28.7533 -64.6242

H 19.5471 29.8833 -63.3462

H 18.8261 30.4823 -64.8412

O 5.5351 23.7133 -62.1712

O 3.2721 22.8043 -61.1222

O 5.5391 25.0493 -64.5692

O -0.3519 25.0243 -63.9512

C -2.1299 25.2193 -59.5452

C -0.8739 25.0373 -58.9552

C -3.2639 25.3083 -58.7282

C -0.7559 24.9283 -57.5872

H -0.0009 24.9853 -59.5892

C -3.1439 25.1953 -57.3632

H -4.2319 25.4563 -59.1832

C -1.8909 25.0003 -56.7782

H 0.2121 24.7853 -57.1312

H -4.0119 25.2483 -56.7232

C -3.2679 25.3683 -70.2722

C -2.0039 25.2083 -70.8522

C -4.3889 25.4823 -71.0992

C -1.8719 25.1253 -72.2192

H -1.1379 25.1403 -70.2112

C -4.2559 25.3853 -72.4672

H -5.3599 25.6423 -70.6572

C -2.9979 25.1923 -73.0392

H -0.9039 24.9963 -72.6782

H -5.1199 25.4583 -73.1072

C -6.5939 24.9023 -65.0102

H -6.3179 23.9553 -64.5202

H -6.7839 25.6543 -64.2322

C 0.6521 24.6463 -64.8732

H 0.3341 23.7313 -65.3972

H 0.8051 25.4403 -65.6162

C -1.6889 23.2553 2.7198

C -1.6769 22.8783 -2.9192

C -1.6689 22.7873 -4.1242

C -1.7579 22.7263 -5.5242

C -0.5989 22.7743 -6.3252

C -3.0219 22.6323 -6.1072

H -3.8639 22.5683 -5.4382

O -2.7309 23.3483 3.3548

O -0.5049 23.2283 3.2868

H -0.5809 23.2953 4.2908

C -2.0529 23.0103 -16.5452

C -2.0639 22.8413 -10.8942

C -2.1109 22.7873 -9.6862

C -2.0319 22.7373 -8.2852

C -3.1869 22.6293 -7.4832

C -0.7639 22.7913 -7.6992

H 0.0811 22.8573 -8.3662

O -1.0119 23.0743 -17.1942

O -3.2399 23.0003 -17.1042

H -3.1999 23.0723 -18.1052

C -6.7769 22.3573 -8.1622

C -6.7519 22.2003 -9.5362

H -5.8139 21.9683 -10.0112

C -7.9169 22.3873 -10.2732

C -9.1149 22.7133 -9.6212

C -9.1429 22.8193 -8.2232

C -7.9659 22.6403 -7.5062

H -7.9599 22.7403 -6.4322

C -6.9609 21.7233 -12.3532

H -6.0009 22.2083 -12.1352

H -6.8889 20.6643 -12.0682

C -7.2539 21.8453 -13.8472

H -7.3249 22.9033 -14.1102

H -6.3949 21.4233 -14.3752

C -8.5379 21.1253 -14.2662

H -8.6409 20.2133 -13.6762

H -8.4589 20.8413 -15.3202

C -9.7919 21.9793 -14.0812

H -9.8049 22.3793 -13.0682

H -9.7639 22.8223 -14.7782

C -11.0529 21.1543 -14.3202

H -11.0599 20.3283 -13.6052

H -11.0239 20.7183 -15.3232

C -12.3339 21.9703 -14.1662

H -12.3229 22.4813 -13.2032

H -12.3729 22.7373 -14.9452

C -13.5749 21.0863 -14.2612

H -13.5469 20.3473 -13.4562

H -13.5529 20.5313 -15.2022

C -14.8829 21.8683 -14.1722

H -14.9249 22.3973 -13.2182

H -14.9169 22.6173 -14.9662

C -16.0919 20.9433 -14.2932

H -16.0379 20.1833 -13.5092

H -16.0499 20.4193 -15.2522

C -17.4219 21.6833 -14.1812

H -17.4909 22.4273 -14.9772

H -17.4609 22.2123 -13.2272

C -18.6089 20.7273 -14.2772

H -18.5669 20.1963 -15.2312

H -18.5309 19.9803 -13.4842

C -19.9439 21.4493 -14.1572

H -20.0469 22.1933 -14.9432

H -20.7689 20.7453 -14.2432

H -20.0199 21.9533 -13.1972

C -11.3179 22.1003 -10.2562

H -11.3359 21.4483 -11.1382

H -11.1939 21.4683 -9.3732

C -12.6129 22.9033 -10.1592

H -12.5799 23.5113 -9.2542

H -12.6649 23.5823 -11.0122

C -13.8309 21.9883 -10.1442

H -13.7589 21.3103 -9.2892

H -13.8289 21.3743 -11.0472

C -15.1499 22.7533 -10.0632

H -15.1699 23.3523 -9.1502

H -15.2239 23.4403 -10.9092

C -16.3429 21.8013 -10.0762

H -16.2729 21.1303 -9.2162

H -16.2999 21.1853 -10.9772

C -17.6849 22.5263 -10.0292

H -17.7409 23.1343 -9.1242

H -17.7629 23.2033 -10.8842

C -18.8529 21.5423 -10.0542

H -18.7859 20.8883 -9.1812

H -18.7739 20.9143 -10.9442

C -20.2099 22.2403 -10.0512

H -20.2999 22.8563 -9.1542

H -20.2769 22.9063 -10.9152

C -21.3609 21.2383 -10.0952

H -21.3149 20.6023 -9.2072

H -21.2449 20.5933 -10.9692

C -22.7239 21.9213 -10.1532

H -22.8379 22.5783 -9.2872

H -22.7769 22.5453 -11.0482

C -23.8699 20.9123 -10.1752

H -23.8349 20.3093 -9.2642

H -23.7389 20.2413 -11.0262

C -25.2299 21.5923 -10.2732

H -25.2949 22.1853 -11.1832

H -26.0269 20.8523 -10.2882

H -25.3909 22.2523 -9.4232

C -10.3949 23.4773 -6.2942

H -10.2029 22.5993 -5.6602

H -9.6309 24.2383 -6.0822

C -11.7799 24.0483 -6.0012

H -11.7679 24.4983 -5.0062

H -11.9829 24.8363 -6.7292

C -12.8749 22.9883 -6.0812

H -12.8049 22.4813 -7.0432

H -12.7149 22.2423 -5.2982

C -14.2689 23.5903 -5.9292

H -14.4359 24.3143 -6.7302

H -14.3339 24.1283 -4.9812

C -15.3579 22.5213 -5.9832

H -15.2309 21.8333 -5.1432

H -15.2499 21.9413 -6.9022

C -16.7609 23.1213 -5.9312

H -16.8919 23.8003 -6.7772

H -16.8709 23.7093 -5.0182

C -17.8449 22.0473 -5.9762

H -17.7109 21.4363 -6.8712

H -17.7389 21.3903 -5.1092

C -19.2489 22.6443 -5.9802

H -19.3609 23.2933 -6.8522

H -19.3819 23.2633 -5.0902

C -20.3289 21.5673 -6.0102

H -20.2279 20.9293 -5.1282

H -20.1849 20.9383 -6.8912

C -21.7349 22.1603 -6.0402

H -21.8879 22.7773 -5.1512

H -21.8319 22.8113 -6.9132

C -22.8129 21.0813 -6.1002

H -22.7219 20.4323 -5.2252

H -22.6529 20.4653 -6.9872

C -24.2139 21.6753 -6.1462

H -24.3259 22.3193 -7.0152

H -24.9639 20.8893 -6.2042

H -24.4079 22.2693 -5.2552

O -10.2129 22.9853 -10.3922

O -8.0019 22.3313 -11.6252

O -10.3479 23.1153 -7.6592

O -4.3759 22.5273 -8.1232

C 2.9881 22.8123 -5.5932

C 3.0091 23.4983 -4.3892

H 2.1141 24.0103 -4.0752

C 4.1751 23.5263 -3.6302

C 5.3251 22.8823 -4.1032

C 5.2991 22.2073 -5.3302

C 4.1181 22.1583 -6.0582

H 4.0751 21.6223 -6.9932

C 3.2391 24.8733 -1.8932

H 2.3351 24.2503 -1.8912

H 3.0431 25.7683 -2.5012

C 3.5711 25.2683 -0.4562

H 3.7771 24.3603 0.1118

H 2.6761 25.7343 -0.0412

C 4.7621 26.2253 -0.3342

H 4.7971 26.8713 -1.2132

H 4.6151 26.8693 0.5368

C 6.0971 25.4983 -0.1832

H 6.2231 24.7973 -1.0072

H 6.0841 24.9183 0.7428

C 7.2681 26.4783 -0.1422

H 7.3501 26.9853 -1.1072

H 7.0671 27.2463 0.6108

C 8.5941 25.7963 0.1838

H 8.7931 25.0183 -0.5532

H 8.5171 25.3113 1.1598

C 9.7561 26.7873 0.2038

H 9.8741 27.2243 -0.7912

H 9.5241 27.6043 0.8908

C 11.0721 26.1383 0.6288

H 11.3131 25.3263 -0.0592

H 10.9511 25.7063 1.6238

C 12.2231 27.1413 0.6508

H 12.3401 27.5773 -0.3442

H 11.9811 27.9543 1.3388

C 13.5431 26.5023 1.0768

H 13.4261 26.0643 2.0708

H 13.7931 25.6943 0.3868

C 14.6881 27.5113 1.1028

H 14.4421 28.3203 1.7948

H 14.8031 27.9523 0.1098

C 16.0021 26.8683 1.5258

H 15.9071 26.4323 2.5178

H 16.8021 27.6053 1.5488

H 16.2831 26.0793 0.8318

C 7.3611 23.9913 -3.7542

H 7.0281 24.9263 -3.2792

H 7.3311 24.1333 -4.8422

C 8.7751 23.6413 -3.3082

H 9.1071 22.7603 -3.8582

H 8.7391 23.3753 -2.2502

C 9.7551 24.7913 -3.5152

H 9.7561 25.0903 -4.5662

H 9.4331 25.6543 -2.9282

C 11.1681 24.3923 -3.0952

H 11.5151 23.5683 -3.7232

H 11.1421 24.0283 -2.0652

C 12.1581 25.5493 -3.1852

H 12.1591 25.9513 -4.2022

H 11.8371 26.3513 -2.5162

C 13.5701 25.1093 -2.8092

H 13.9071 24.3373 -3.5042

H 13.5521 24.6663 -1.8102

C 14.5631 26.2673 -2.8202

H 14.5521 26.7463 -3.8032

H 14.2531 27.0163 -2.0892

C 15.9801 25.8003 -2.5002

H 16.2971 25.0743 -3.2522

H 15.9811 25.2933 -1.5322

C 16.9771 26.9543 -2.4652

H 16.9331 27.4973 -3.4132

H 16.6991 27.6543 -1.6742

C 18.4041 26.4653 -2.2312

H 18.6641 25.7383 -3.0042

H 18.4581 25.9573 -1.2652

C 19.4211 27.6033 -2.2582

H 19.3561 28.1213 -3.2182

H 19.1771 28.3253 -1.4752

C 20.8421 27.0933 -2.0552

H 20.9321 26.5833 -1.0982

H 21.5521 27.9163 -2.0732

H 21.1101 26.3923 -2.8422

C 6.6011 21.1393 -7.0272

H 6.2551 21.8743 -7.7672

H 5.9981 20.2263 -7.1322

C 8.0791 20.8323 -7.2452

H 8.2021 20.3863 -8.2312

H 8.3951 20.1053 -6.4962

C 8.9351 22.0903 -7.1242

H 8.6861 22.5863 -6.1852

H 8.6951 22.7743 -7.9402

C 10.4291 21.7813 -7.1402

H 10.6631 21.0893 -6.3282

H 10.6931 21.2873 -8.0782

C 11.2611 23.0503 -6.9752

H 11.0671 23.7193 -7.8162

H 10.9471 23.5663 -6.0662

C 12.7561 22.7623 -6.8942

H 12.9531 22.1143 -6.0372

H 13.0731 22.2233 -7.7902

C 13.5781 24.0413 -6.7572

H 13.2421 24.5923 -5.8772

H 13.4021 24.6773 -7.6282

C 15.0711 23.7543 -6.6332

H 15.2441 23.1543 -5.7362

H 15.3991 23.1623 -7.4902

C 15.9061 25.0293 -6.5522

H 15.7681 25.6133 -7.4652

H 15.5571 25.6373 -5.7152

C 17.3891 24.7223 -6.3682

H 17.7371 24.1073 -7.2012

H 17.5221 24.1423 -5.4522

C 18.2431 25.9843 -6.2842

H 18.1211 26.5653 -7.2012

H 17.8951 26.6003 -5.4532

C 19.7181 25.6553 -6.0862

H 19.8631 25.0833 -5.1722

H 20.3101 26.5653 -6.0172

H 20.0931 25.0643 -6.9192

O 6.4951 22.9303 -3.3732

O 4.3271 24.1593 -2.4382

O 6.4761 21.6643 -5.7222

O 0.5881 22.8113 -5.6752

C -1.6739 22.9733 -1.5182

C -0.4639 23.0603 -0.8222

C -2.8819 22.9843 -0.8112

C -0.4609 23.1473 0.5508

H 0.4601 23.0563 -1.3792

C -2.8729 23.0743 0.5598

H -3.8119 22.9213 -1.3542

C -1.6659 23.1553 1.2518

H 0.4681 23.2133 1.0948

H -3.7899 23.0833 1.1288

C -2.0559 22.8843 -12.2982

C -0.8609 22.7353 -13.0092

C -3.2519 23.0733 -12.9962

C -0.8659 22.7783 -14.3842

H 0.0621 22.5943 -12.4672

C -3.2589 23.0933 -14.3702

H -4.1659 23.2083 -12.4382

C -2.0639 22.9523 -15.0772

H 0.0481 22.6683 -14.9452

H -4.1839 23.2233 -14.9102

C -5.5199 22.2503 -7.3382

H -5.4269 21.2383 -6.9132

H -5.5959 22.9693 -6.5122

C 1.7591 22.8273 -6.4622

H 1.7741 21.9553 -7.1312

H 1.7741 23.7413 -7.0782

C -3.1439 19.0143 -55.0662

C -3.4529 19.3943 -60.7092

C -3.5759 19.3933 -61.9132

C -3.7779 19.3853 -63.3042

C -2.6919 19.2673 -64.1962

C -5.0819 19.4473 -63.8012

H -5.8859 19.4983 -63.0832

O -4.1589 19.0163 -54.3802

O -1.9379 18.9043 -54.5512

H -1.9599 18.8403 -53.5522

C -3.6579 19.0733 -74.2762

C -4.3369 19.4553 -68.6612

C -4.3949 19.4413 -67.4522

C -4.2559 19.4073 -66.0522

C -5.3479 19.4253 -65.1622

C -2.9539 19.3133 -65.5552

H -2.1589 19.2583 -66.2802

O -2.5489 19.0063 -74.7872

O -4.7729 19.0263 -74.9672

H -4.5969 18.9553 -75.9552

C -8.8929 18.8273 -65.7472

C -8.7529 18.4803 -67.0792

H -7.7629 18.3093 -67.4642

C -9.8789 18.3853 -67.8872

C -11.1569 18.6003 -67.3472

C -11.2909 18.9043 -65.9852

C -10.1499 19.0183 -65.1972

H -10.2309 19.2733 -64.1522

C -8.6569 17.8103 -69.8602

H -7.8669 18.5133 -69.5662

H -8.3409 16.7963 -69.5712

C -8.8669 17.8893 -71.3722

H -9.0859 18.9223 -71.6452

H -7.9249 17.6063 -71.8462

C -10.0039 16.9873 -71.8612

H -10.0329 16.0883 -71.2452

H -9.8049 16.6833 -72.8932

C -11.3689 17.6683 -71.7932

H -11.5049 18.0903 -70.7982

H -11.4019 18.4873 -72.5172

C -12.5059 16.6903 -72.0742

H -12.4739 15.8873 -71.3342

H -12.3739 16.2403 -73.0632

C -13.8669 17.3763 -72.0032

H -13.9429 17.9033 -71.0512

H -13.9469 18.1223 -72.8002

C -15.0139 16.3773 -72.1172

H -14.9119 15.6443 -71.3142

H -14.9379 15.8383 -73.0642

C -16.3829 17.0473 -72.0252

H -16.4489 17.6043 -71.0892

H -16.4909 17.7673 -72.8412

C -17.5199 16.0323 -72.0902

H -17.4119 15.3263 -71.2622

H -17.4439 15.4613 -73.0182

C -18.8969 16.6863 -72.0122

H -19.0139 17.3873 -72.8432

H -18.9729 17.2613 -71.0862

C -20.0219 15.6553 -72.0602

H -19.9379 15.0733 -72.9792

H -19.9069 14.9633 -71.2212

C -21.3999 16.3013 -71.9932

H -21.5379 16.9953 -72.8202

H -22.1809 15.5463 -72.0462

H -21.5179 16.8533 -71.0642

C -13.2619 17.6493 -67.9582

H -13.1889 16.8433 -68.7002

H -13.1529 17.2073 -66.9652

C -14.6139 18.3483 -68.0782

H -14.7149 19.0663 -67.2622

H -14.6369 18.9073 -69.0152

C -15.7449 17.3283 -68.0452

H -15.6389 16.7133 -67.1482

H -15.6429 16.6623 -68.9032

C -17.1329 17.9633 -68.0582

H -17.2459 18.6193 -67.1922

H -17.2459 18.5813 -68.9522

C -18.2209 16.8923 -68.0382

H -18.0719 16.2513 -67.1642

H -18.1159 16.2623 -68.9232

C -19.6349 17.4653 -67.9932

H -19.7559 18.0743 -67.0942

H -19.7929 18.1183 -68.8552

C -20.6799 16.3523 -68.0012

H -20.5099 15.6973 -67.1412

H -20.5499 15.7473 -68.9002

C -22.1119 16.8753 -67.9502

H -22.2609 17.4413 -67.0282

H -22.2809 17.5583 -68.7862

C -23.1249 15.7353 -68.0172

H -22.9649 15.0643 -67.1692

H -22.9549 15.1583 -68.9302

C -24.5689 16.2283 -67.9972

H -24.7439 16.8013 -67.0842

H -24.7329 16.8983 -68.8452

C -25.5659 15.0743 -68.0682

H -25.4179 14.4193 -67.2052

H -25.3709 14.4873 -68.9682

C -27.0089 15.5613 -68.0832

H -27.1859 16.2033 -68.9432

H -27.6969 14.7203 -68.1372

H -27.2319 16.1273 -67.1822

C -12.7449 19.5503 -64.2022

H -12.4519 18.7683 -63.4862

H -12.1279 20.4413 -64.0202

C -14.2169 19.9073 -64.0162

H -14.3329 20.3823 -63.0402

H -14.4929 20.6313 -64.7842

C -15.1309 18.6893 -64.1132

H -14.9899 18.2183 -65.0862

H -14.8469 17.9633 -63.3462

C -16.5969 19.0733 -63.9312

H -16.8659 19.8303 -64.6722

H -16.7319 19.5203 -62.9422

C -17.5309 17.8753 -64.0762

H -17.2479 17.1053 -63.3552

H -17.4169 17.4483 -65.0732

C -18.9929 18.2553 -63.8602

H -19.2809 19.0143 -64.5922

H -19.1109 18.6983 -62.8682

C -19.9199 17.0493 -63.9912

H -19.7739 16.5893 -64.9692

H -19.6539 16.3073 -63.2352

C -21.3889 17.4283 -63.8312

H -21.6599 18.1553 -64.6002

H -21.5379 17.9093 -62.8612

C -22.3049 16.2123 -63.9402

H -22.0569 15.5023 -63.1472

H -22.1259 15.7143 -64.8962

C -23.7809 16.5823 -63.8372

H -23.9689 17.0743 -62.8802

H -24.0299 17.2953 -64.6272

C -24.6859 15.3593 -63.9622

H -24.4529 14.6563 -63.1572

H -24.4799 14.8573 -64.9092

C -26.1619 15.7293 -63.8982

H -26.4129 16.4263 -64.6942

H -26.7849 14.8443 -64.0092

H -26.3979 16.2003 -62.9462

O -12.2139 18.5773 -68.2152

O -9.8689 18.1243 -69.2162

O -12.5609 19.0943 -65.5252

O -6.5879 19.4143 -65.7062

C 0.8411 18.4593 -63.7962

C 0.8201 17.7453 -62.6122

H -0.1259 17.3923 -62.2412

C 2.0011 17.5323 -61.9102

C 3.2191 18.0113 -62.4222

C 3.2341 18.6903 -63.6502

C 2.0361 18.9283 -64.3132

H 2.0211 19.4783 -65.2392

C 0.8711 16.5143 -60.0852

H 0.3541 15.7463 -60.6802

H 0.2141 17.3923 -60.0002

C 1.1721 15.9643 -58.6932

H 1.7451 15.0423 -58.7972

H 0.2091 15.7203 -58.2362

C 1.9381 16.9503 -57.8042

H 1.6121 17.9683 -58.0282

H 1.7001 16.7533 -56.7542

C 3.4481 16.8413 -58.0002

H 3.6781 16.9223 -59.0622

H 3.7741 15.8533 -57.6632

C 4.2311 17.9023 -57.2302

H 4.0171 18.8883 -57.6492

H 3.9121 17.9163 -56.1842

C 5.7311 17.6273 -57.3092

H 6.0031 17.4573 -58.3522

H 5.9551 16.7123 -56.7542

C 6.5821 18.7653 -56.7522

H 6.4011 19.6713 -57.3352

H 6.2831 18.9763 -55.7222

C 8.0691 18.4213 -56.7942

H 8.3471 18.1643 -57.8182

H 8.2531 17.5433 -56.1702

C 8.9481 19.5713 -56.3102

H 8.7921 20.4413 -56.9532

H 8.6461 19.8573 -55.2992

C 10.4281 19.1963 -56.3122

H 10.5821 18.3323 -55.6622

H 10.7241 18.9083 -57.3232

C 11.3151 20.3443 -55.8382

H 11.0021 20.6523 -54.8372

H 11.1801 21.2033 -56.4992

C 12.7861 19.9483 -55.8112

H 12.9431 19.1083 -55.1382

H 13.4031 20.7783 -55.4742

H 13.1191 19.6563 -56.8052

C 5.0531 18.9273 -61.2312

H 4.6741 19.1683 -60.2272

H 4.8681 19.7853 -61.8842

C 6.5451 18.6243 -61.1812

H 6.8611 18.3353 -62.1852

H 6.7031 17.7703 -60.5212

C 7.3801 19.8053 -60.6952

H 7.2111 20.6703 -61.3412

H 7.0721 20.0813 -59.6852

C 8.8651 19.4503 -60.6972

H 9.1611 19.1613 -61.7092

H 9.0251 18.5843 -60.0502

C 9.7601 20.5913 -60.2222

H 9.6191 21.4633 -60.8662

H 9.4761 20.8793 -59.2082

C 11.2301 20.1803 -60.2382

H 11.5101 19.8903 -61.2542

H 11.3631 19.3023 -59.6002

C 12.1631 21.2883 -59.7572

H 12.0551 22.1633 -60.4022

H 11.8811 21.5863 -58.7462

C 13.6201 20.8313 -59.7602

H 13.8981 20.5313 -60.7732

H 13.7221 19.9523 -59.1182

C 14.5771 21.9173 -59.2782

H 14.4891 22.7913 -59.9282

H 14.2951 22.2263 -58.2682

C 16.0251 21.4333 -59.2732

H 16.2881 21.0753 -60.2722

H 16.1211 20.5883 -58.5872

C 17.0031 22.5293 -58.8612

H 16.8891 23.3863 -59.5292

H 16.7641 22.8643 -57.8492

C 18.4461 22.0413 -58.9072

H 18.5851 21.1953 -58.2372

H 19.1301 22.8323 -58.6062

H 18.7091 21.7263 -59.9142

C 4.4921 19.8033 -65.3412

H 3.9971 20.7783 -65.2212

H 3.9721 19.2393 -66.1282

C 5.9451 20.0003 -65.7542

H 5.9501 20.5303 -66.7082

H 6.3981 19.0193 -65.9092

C 6.7651 20.7763 -64.7282

H 6.8071 20.2043 -63.8002

H 6.2761 21.7293 -64.5112

C 8.1771 21.0303 -65.2462

H 8.6131 20.0823 -65.5702

H 8.1261 21.6823 -66.1212

C 9.0941 21.6613 -64.2022

H 8.6641 22.6033 -63.8542

H 9.1701 20.9943 -63.3402

C 10.4881 21.9123 -64.7722

H 10.8701 20.9803 -65.1942

H 10.4191 22.6373 -65.5862

C 11.4741 22.4203 -63.7242

H 11.5421 21.6913 -62.9132

H 11.1061 23.3553 -63.2962

C 12.8631 22.6413 -64.3172

H 13.1951 21.7213 -64.8042

H 12.8131 23.4163 -65.0862

C 13.8871 23.0413 -63.2582

H 13.5791 23.9793 -62.7892

H 13.9101 22.2773 -62.4772

C 15.2881 23.2023 -63.8422

H 15.2841 24.0043 -64.5842

H 15.5721 22.2803 -64.3562

C 16.3241 23.5103 -62.7642

H 16.0531 24.4393 -62.2572

H 16.3121 22.7113 -62.0212

C 17.7281 23.6383 -63.3412

H 18.0231 22.7143 -63.8352

H 18.4481 23.8503 -62.5532

H 17.7711 24.4433 -64.0712

O 4.3721 17.7723 -61.7142

O 2.0751 16.8833 -60.7212

O 4.4491 19.0913 -64.1202

O -1.4569 19.1053 -63.6622

C -3.3519 19.3413 -59.3062

C -2.1139 19.1563 -58.6822

C -4.5069 19.4433 -58.5212

C -2.0329 19.0563 -57.3112

H -1.2239 19.0953 -59.2912

C -4.4239 19.3393 -57.1532

H -5.4609 19.5933 -59.0042

C -3.1889 19.1403 -56.5332

H -1.0789 18.9113 -56.8272

H -5.3089 19.4013 -56.5372

C -4.1999 19.4223 -70.0612

C -2.9209 19.2513 -70.6062

C -5.2969 19.5373 -70.9192

C -2.7529 19.1583 -71.9692

H -2.0729 19.1823 -69.9412

C -5.1269 19.4303 -72.2822

H -6.2789 19.7063 -70.5042

C -3.8559 19.2253 -72.8192

H -1.7739 19.0203 -72.4002

H -5.9739 19.5043 -72.9462

C -7.6689 19.0123 -64.8882

H -7.4119 18.0673 -64.3842

H -7.8749 19.7713 -64.1202

C -0.4309 18.7143 -64.5542

H -0.7399 17.7983 -65.0802

H -0.2529 19.5033 -65.2982

C -1.1269 16.7793 1.7928

C -0.9789 16.3543 -3.8512

C -1.0179 16.3593 -5.0602

C -1.0819 16.3923 -6.4642

C 0.0991 16.5173 -7.2252

C -2.3239 16.3533 -7.0992

H -3.1999 16.2853 -6.4742

O -2.1719 16.6123 2.4078

O -0.0019 17.1263 2.3738

H -0.1039 17.1843 3.3728

C -1.9029 16.6353 -17.4702

C -1.3249 16.3243 -11.8522

C -1.3049 16.3773 -10.6442

C -1.2429 16.4203 -9.2412

C -2.4299 16.4253 -8.4792

C -0.0009 16.4843 -8.6052

H 0.8791 16.5113 -9.2292

O -0.9639 16.8683 -18.2232

O -3.1449 16.5253 -17.8872

H -3.2339 16.6503 -18.8792

C -5.8689 17.3983 -9.2582

C -5.8659 17.3293 -10.6392

H -5.0019 16.9213 -11.1352

C -6.9789 17.7713 -11.3532

C -8.1019 18.2533 -10.6682

C -8.0739 18.3703 -9.2712

C -6.9479 17.9503 -8.5782

H -6.9019 18.0273 -7.5042

C -6.0709 17.2123 -13.4972

H -5.1169 17.7213 -13.2972

H -5.9529 16.1453 -13.2602

C -6.4299 17.3863 -14.9722

H -6.6189 18.4433 -15.1602

H -5.5539 17.0853 -15.5502

C -7.6459 16.5693 -15.4202

H -7.6219 15.5913 -14.9332

H -7.5799 16.4003 -16.4982

C -8.9709 17.2623 -15.1112

H -8.9899 17.5523 -14.0612

H -9.0379 18.1763 -15.7052

C -10.1739 16.3733 -15.4202

H -10.1309 15.4773 -14.7942

H -10.1269 16.0453 -16.4622

C -11.4959 17.0963 -15.1752

H -11.5059 17.4903 -14.1582

H -11.5719 17.9493 -15.8532

C -12.7049 16.1833 -15.3742

H -12.6669 15.3693 -14.6462

H -12.6569 15.7323 -16.3692

C -14.0249 16.9363 -15.2262

H -14.0559 17.4273 -14.2522

H -14.0789 17.7183 -15.9862

C -15.2369 16.0173 -15.3692

H -15.2489 15.2993 -14.5442

H -15.1479 15.4433 -16.2952

C -16.5479 16.7993 -15.3862

H -16.5349 17.4963 -16.2262

H -16.6309 17.3873 -14.4702

C -17.7669 15.8893 -15.5142

H -17.6529 15.2553 -16.3982

H -17.8209 15.2273 -14.6462

C -19.0599 16.6863 -15.6292

H -19.0329 17.3243 -16.5102

H -19.9159 16.0213 -15.7152

H -19.1989 17.3163 -14.7542

C -10.2789 17.6193 -11.2402

H -10.1959 16.8943 -12.0652

H -10.1719 17.0683 -10.2992

C -11.6349 18.3103 -11.2802

H -11.7069 18.9853 -10.4272

H -11.6889 18.9153 -12.1872

C -12.7839 17.3063 -11.2572

H -12.7089 16.6873 -10.3592

H -12.7029 16.6413 -12.1192

C -14.1409 18.0043 -11.2812

H -14.2429 18.6323 -10.3952

H -14.1899 18.6603 -12.1532

C -15.3009 17.0133 -11.3382

H -15.2599 16.3553 -10.4672

H -15.1979 16.3863 -12.2272

C -16.6519 17.7223 -11.3772

H -16.7689 18.3293 -10.4772

H -16.6739 18.4013 -12.2332

C -17.8159 16.7423 -11.4852

H -17.7999 16.0663 -10.6262

H -17.6919 16.1333 -12.3832

C -19.1639 17.4553 -11.5412

H -19.2929 18.0553 -10.6392

H -19.1759 18.1383 -12.3942

C -20.3269 16.4763 -11.6682

H -20.3219 15.7983 -10.8112

H -20.1949 15.8703 -12.5682

C -21.6739 17.1923 -11.7352

H -21.7929 17.8163 -10.8462

H -21.6879 17.8513 -12.6072

C -22.8429 16.2163 -11.8242

H -22.8339 15.5623 -10.9492

H -22.7199 15.5863 -12.7092

C -24.1839 16.9373 -11.8992

H -24.2209 17.5863 -12.7722

H -24.9999 16.2223 -11.9702

H -24.3399 17.5473 -11.0122

C -9.2569 18.9933 -7.2922

H -9.0299 18.0323 -6.8092

H -8.5229 19.7403 -6.9582

C -10.6719 19.4333 -6.9352

H -10.7009 19.7113 -5.8812

H -10.9109 20.3163 -7.5272

C -11.6949 18.3333 -7.2172

H -11.5229 17.9433 -8.2212

H -11.5509 17.5123 -6.5102

C -13.1249 18.8523 -7.1162

H -13.2719 19.6313 -7.8682

H -13.2769 19.3113 -6.1382

C -14.1679 17.7573 -7.3252

H -14.0789 17.0113 -6.5312

H -13.9789 17.2503 -8.2742

C -15.5819 18.3313 -7.3292

H -15.6749 19.0373 -8.1582

H -15.7449 18.8883 -6.4052

C -16.6599 17.2603 -7.4662

H -16.4969 16.6923 -8.3842

H -16.5869 16.5613 -6.6302

C -18.0569 17.8753 -7.4922

H -18.1419 18.5303 -8.3632

H -18.1939 18.4933 -6.6022

C -19.1609 16.8243 -7.5482

H -19.0939 16.1813 -6.6662

H -19.0169 16.1943 -8.4282

C -20.5479 17.4603 -7.6042

H -20.6939 18.0863 -6.7212

H -20.6089 18.1063 -8.4832

C -21.6619 16.4193 -7.6752

H -21.6129 15.7763 -6.7932

H -21.5099 15.7883 -8.5532

C -23.0389 17.0673 -7.7532

H -23.1139 17.6963 -8.6372

H -23.8179 16.3103 -7.8082

H -23.2219 17.6853 -6.8772

O -9.2409 18.5843 -11.3682

O -7.0989 17.7673 -12.7072

O -9.1989 18.8623 -8.6982

O -3.6019 16.5213 -9.1632

C 3.4471 17.4683 -6.1862

C 3.1091 18.2253 -5.0752

H 2.1011 18.5963 -4.9892

C 4.0571 18.4653 -4.0872

C 5.3631 17.9703 -4.2352

C 5.7081 17.2523 -5.3882

C 4.7381 16.9933 -6.3502

H 4.9741 16.4063 -7.2242

C 2.5641 19.6943 -2.6802

H 1.7731 18.9733 -2.9212

H 2.4221 20.5883 -3.3052

C 2.4741 20.0583 -1.1992

H 2.5291 19.1433 -0.6072

H 1.4931 20.5083 -1.0402

C 3.5661 21.0253 -0.7392

H 3.8031 21.7093 -1.5532

H 3.1891 21.6163 0.1008

C 4.8481 20.3153 -0.3092

H 5.1671 19.6443 -1.1052

H 4.6481 19.7123 0.5808

C 5.9631 21.3143 -0.0152

H 6.1551 21.9003 -0.9162

H 5.6421 22.0043 0.7708

C 7.2541 20.6223 0.4128

H 7.5021 19.8583 -0.3242

H 7.1091 20.1223 1.3748

C 8.4071 21.6153 0.5098

H 8.4901 22.1303 -0.4492

H 8.1811 22.3733 1.2648

C 9.7431 20.9523 0.8368

H 9.9121 20.1253 0.1448

H 9.7111 20.5373 1.8468

C 10.8981 21.9433 0.7238

H 10.9371 22.3203 -0.3012

H 10.7111 22.7993 1.3768

C 12.2481 21.3243 1.0768

H 12.2331 20.9933 2.1178

H 12.4161 20.4453 0.4518

C 13.3931 22.3123 0.8748

H 13.2121 23.2043 1.4798

H 13.4131 22.6243 -0.1722

C 14.7431 21.7153 1.2478

H 14.7521 21.4203 2.2948

H 15.5411 22.4373 1.0888

H 14.9511 20.8363 0.6418

C 7.3151 19.0733 -3.4732

H 7.0641 20.0383 -3.0132

H 7.4191 19.2303 -4.5492

C 8.6271 18.5423 -2.9052

H 8.8881 17.6173 -3.4242

H 8.4901 18.3013 -1.8502

C 9.7271 19.5853 -3.0752

H 9.7531 19.9013 -4.1212

H 9.4741 20.4613 -2.4772

C 11.1131 19.0843 -2.6762

H 11.3701 18.2033 -3.2702

H 11.1101 18.7803 -1.6272

C 12.1641 20.1713 -2.8922

H 12.1091 20.5183 -3.9282

H 11.9301 21.0233 -2.2502

C 13.5881 19.7033 -2.6032

H 13.8341 18.8593 -3.2532

H 13.6561 19.3523 -1.5712

C 14.5961 20.8293 -2.8242

H 14.4921 21.2083 -3.8452

H 14.3651 21.6533 -2.1462

C 16.0391 20.3883 -2.6022

H 16.2931 19.5993 -3.3142

H 16.1411 19.9703 -1.5972

C 17.0131 21.5533 -2.7632

H 16.8971 21.9833 -3.7622

H 16.7611 22.3323 -2.0402

C 18.4671 21.1333 -2.5672

H 18.7261 20.3703 -3.3052

H 18.5831 20.6873 -1.5762

C 19.4251 22.3133 -2.7022

H 19.3141 22.7553 -3.6962

H 19.1591 23.0773 -1.9682

C 20.8771 21.9013 -2.5012

H 21.0171 21.4683 -1.5132

H 21.5371 22.7613 -2.5942

H 21.1711 21.1623 -3.2432

C 7.3741 15.9923 -6.5422

H 7.2221 16.5053 -7.5032

H 6.7631 15.0793 -6.5302

C 8.8481 15.6413 -6.3752

H 9.1181 14.9073 -7.1372

H 8.9911 15.1873 -5.3942

C 9.7301 16.8793 -6.5042

H 9.4581 17.5843 -5.7192

H 9.5341 17.3563 -7.4672

C 11.2171 16.5523 -6.4022

H 11.4141 16.0273 -5.4652

H 11.4971 15.8833 -7.2202

C 12.0681 17.8173 -6.4662

H 11.8031 18.3803 -7.3642

H 11.8391 18.4483 -5.6062

C 13.5631 17.5163 -6.4872

H 13.8311 16.9393 -5.5982

H 13.7971 16.9013 -7.3602

C 14.3951 18.7943 -6.5312

H 14.1681 19.4013 -5.6532

H 14.1131 19.3773 -7.4112

C 15.8941 18.5113 -6.5702

H 16.1781 17.9503 -5.6762

H 16.1251 17.8843 -7.4362

C 16.7131 19.7963 -6.6422

H 16.4641 20.3323 -7.5622

H 16.4421 20.4413 -5.8042

C 18.2151 19.5303 -6.6042

H 18.4961 18.8993 -7.4502

H 18.4601 18.9833 -5.6902

C 19.0241 20.8243 -6.6452

H 18.8001 21.3603 -7.5702

H 18.7191 21.4613 -5.8122

C 20.5221 20.5673 -6.5592

H 20.7661 20.0353 -5.6422

H 21.0741 21.5043 -6.5682

H 20.8571 19.9623 -7.4002

O 6.2571 18.1543 -3.2122

O 3.8341 19.1363 -2.9352

O 7.0021 16.8373 -5.4732

O 1.2531 16.6513 -6.5292

C -0.9839 16.3973 -2.4452

C 0.2011 16.5953 -1.7282

C -2.1949 16.2683 -1.7542

C 0.1681 16.7093 -0.3552

H 1.1361 16.6643 -2.2642

C -2.2219 16.3723 -0.3832

H -3.1049 16.0973 -2.3102

C -1.0449 16.6143 0.3268

H 1.0761 16.8733 0.2028

H -3.1469 16.2843 0.1648

C -1.4289 16.3243 -13.2542

C -0.3449 16.6813 -14.0642

C -2.6489 15.9863 -13.8512

C -0.4959 16.7563 -15.4302

H 0.6061 16.9073 -13.6062

C -2.7969 16.0603 -15.2162

H -3.4679 15.6693 -13.2242

C -1.7309 16.4733 -16.0162

H 0.3291 17.0493 -16.0612

H -3.7329 15.7963 -15.6812

C -4.7459 16.8623 -8.4072

H -5.1129 15.9723 -7.8702

H -4.4849 17.6273 -7.6662

C 2.3901 17.1483 -7.2092

H 2.7711 16.4043 -7.9212

H 2.1101 18.0573 -7.7632

1_2_polymer

C -3.0840 12.1170 -34.0930

C -2.2870 11.8880 -35.1490

C -2.6060 12.1090 -36.5400

C -1.5890 11.9640 -37.5090

C -3.8830 12.4730 -36.9820

H -4.6540 12.5990 -36.2410

C -2.5530 12.2810 -41.6550

C -3.4480 12.5880 -40.7020

C -3.1810 12.4620 -39.2870

C -4.1900 12.6510 -38.3200

C -1.9000 12.1620 -38.8370

H -1.1300 12.1030 -39.5800

O -5.4070 13.0150 -38.8030

C 1.9830 11.0790 -37.6540

C 2.1900 10.5930 -36.3780

H 1.3780 10.6300 -35.6730

C 3.4420 10.0930 -36.0280

C 4.4840 10.0870 -36.9670

C 4.2500 10.5440 -38.2770

C 2.9980 11.0470 -38.6000

H 2.7980 11.4250 -39.5890

C 2.7770 9.6350 -33.7820

H 1.8320 9.1860 -34.1200

H 2.5890 10.6840 -33.5100

C 3.2710 8.8540 -32.5690

H 3.4170 7.8180 -32.8720

H 2.4710 8.8800 -31.8260

C 4.5650 9.3860 -31.9500

H 4.5150 10.4740 -31.8760

H 4.6440 8.9900 -30.9330

C 5.8110 8.9730 -32.7310

H 5.8020 9.4430 -33.7130

H 5.7850 7.8930 -32.8910

C 7.0970 9.3380 -31.9930

H 7.1140 10.4120 -31.7940

H 7.1190 8.8260 -31.0270

C 8.3360 8.9520 -32.7980

H 8.3480 9.5240 -33.7270

H 8.2740 7.8950 -33.0650

C 9.6360 9.2020 -32.0370

H 9.6960 10.2570 -31.7570

H 9.6350 8.6180 -31.1130

C 10.8590 8.8290 -32.8710

H 10.8750 9.4390 -33.7770

H 10.7770 7.7840 -33.1820

C 12.1680 9.0260 -32.1120

H 12.2550 10.0710 -31.8040

H 12.1560 8.4180 -31.2030

C 13.3810 8.6460 -32.9580

H 13.2830 7.6070 -33.2830

H 13.4020 9.2680 -33.8560

C 14.6950 8.8140 -32.2000

H 14.6740 8.1960 -31.2990

H 14.7980 9.8540 -31.8810

C 15.8960 8.4260 -33.0550

H 15.8140 7.3920 -33.3830

H 16.8200 8.5370 -32.4930

H 15.9530 9.0570 -33.9390

C 6.7800 10.5150 -36.5450

H 6.7720 11.0130 -35.5640

H 6.6670 11.2830 -37.3160

C 8.0740 9.7360 -36.7360

H 8.0520 9.2750 -37.7220

H 8.1000 8.9340 -35.9960

C 9.3340 10.5860 -36.6110

H 9.2950 11.4050 -37.3330

H 9.3900 11.0280 -35.6130

C 10.5710 9.7270 -36.8660

H 10.5140 9.3100 -37.8730

H 10.5680 8.8880 -36.1660

C 11.8890 10.4820 -36.7160

H 11.9120 11.3250 -37.4120

H 11.9660 10.8900 -35.7050

C 13.0760 9.5600 -36.9860

H 13.0050 9.1710 -38.0050

H 13.0230 8.7050 -36.3070

C 14.4240 10.2510 -36.8060

H 14.4870 11.1110 -37.4780

H 14.5070 10.6300 -35.7840

C 15.5820 9.2970 -37.0890

H 15.4990 8.9230 -38.1130

H 15.5100 8.4370 -36.4190

C 16.9450 9.9590 -36.9060

H 17.0140 10.8290 -37.5650

H 17.0410 10.3220 -35.8800

C 18.0900 8.9980 -37.2120

H 17.9690 8.6030 -38.2230

H 18.0450 8.1530 -36.5200

C 19.4560 9.6670 -37.0950

H 19.5050 10.5120 -37.7870

H 19.5790 10.0620 -36.0830

C 20.5900 8.6970 -37.4030

H 20.5670 7.8540 -36.7160

H 21.5540 9.1910 -37.3070

H 20.5000 8.3140 -38.4170

C 5.0780 10.8560 -40.4920

H 4.9390 11.9450 -40.5140

H 4.1860 10.3730 -40.9110

C 6.2940 10.4530 -41.3200

H 6.1200 10.7740 -42.3480

H 6.3610 9.3650 -41.3270

C 7.5870 11.0570 -40.7800

H 7.6760 10.8090 -39.7210

H 7.5440 12.1430 -40.8570

C 8.8120 10.5380 -41.5270

H 8.8300 9.4480 -41.4670

H 8.7400 10.8050 -42.5840

C 10.1100 11.0940 -40.9450

H 10.1500 12.1740 -41.1040

H 10.1200 10.9240 -39.8650

C 11.3440 10.4410 -41.5620

H 11.3060 9.3650 -41.3740

H 11.3330 10.5840 -42.6450

C 12.6430 11.0030 -40.9880

H 12.6070 10.9490 -39.8980

H 12.7350 12.0570 -41.2630

C 13.8680 10.2390 -41.4840

H 13.7940 9.1990 -41.1560

H 13.8810 10.2390 -42.5770

C 15.1720 10.8380 -40.9620

H 15.2840 11.8530 -41.3510

H 15.1250 10.9060 -39.8720

C 16.3880 10.0060 -41.3580

H 16.4110 9.8880 -42.4440

H 16.2960 9.0090 -40.9190

C 17.6970 10.6390 -40.8910

H 17.8140 11.6160 -41.3640

H 17.6510 10.7970 -39.8120

C 18.9020 9.7680 -41.2200

H 18.8140 8.7980 -40.7350

H 19.8210 10.2370 -40.8760

H 18.9800 9.6080 -42.2930

O 5.7040 9.5760 -36.5950

O 3.7430 9.5740 -34.8100

O 5.2760 10.4360 -39.1520

O -0.3280 11.6350 -37.1000

C -2.7510 11.8820 -32.7040

C -3.6860 12.1970 -31.7120

C -1.5330 11.3510 -32.2670

C -3.3670 11.9890 -30.3840

H -4.6460 12.6110 -31.9820

C -1.3210 11.1790 -30.9120

H -0.7590 11.0850 -32.9690

H -4.0650 12.2370 -29.5960

H -0.3850 10.7810 -30.5410

C -2.6880 12.4130 -43.0820

C -1.5680 12.1110 -43.8680

C -3.8440 12.8290 -43.7480

C -1.6530 12.2180 -45.2360

H -0.6490 11.7950 -43.3970

C -3.8250 12.9070 -45.1270

H -4.7360 13.0830 -43.2000

H -0.8140 11.9860 -45.8800

H -4.6830 13.2270 -45.7010

C -6.4960 13.1590 -37.9170

H -6.5940 12.2580 -37.2950

H -6.3340 14.0220 -37.2550

C 0.6580 11.6220 -38.1130

H 0.3300 10.9830 -38.9450

H 0.7940 12.6410 -38.5040

C -4.0200 13.1840 -48.8040

C -4.8050 13.6790 -54.3810

C -5.0040 13.7200 -55.5720

C -5.3320 13.7550 -56.9370

C -4.4050 13.3470 -57.9150

C -6.5990 14.2080 -57.3100

H -7.2500 14.5630 -56.5280

O -4.8180 13.6130 -47.9950

O -2.9000 12.5820 -48.4620

C -6.1240 12.4600 -67.7000

C -6.5700 13.4200 -62.1480

C -6.4310 13.5900 -60.9580

C -6.1090 13.7030 -59.5970

C -7.0040 14.1990 -58.6330

C -4.8190 13.3120 -59.2320

H -4.1730 12.9710 -60.0240

O -5.0220 12.2210 -68.1760

O -7.2290 12.4890 -68.4010

H -7.0700 12.2670 -69.3720

O -8.1980 14.6490 -59.0980

C -0.8350 12.4970 -57.9070

C -0.6690 12.1280 -56.5820

H -1.5400 12.0740 -55.9500

C 0.6120 11.8720 -56.0990

C 1.7250 11.9950 -56.9470

C 1.5420 12.3260 -58.3000

C 0.2560 12.5860 -58.7580

H 0.0920 12.8730 -59.7840

C 0.0150 11.6260 -53.7770

H -1.0030 11.3470 -54.0730

H 0.0140 12.6820 -53.4680

C 0.4350 10.7340 -52.6080

H 0.3170 9.6910 -52.9060

H -0.2640 10.9350 -51.7930

C 1.8720 10.9520 -52.1240

H 2.1220 12.0120 -52.1940

H 1.9310 10.6670 -51.0710

C 2.8940 10.1320 -52.9110

H 2.8860 10.4510 -53.9540

H 2.6030 9.0780 -52.8780

C 4.3020 10.2740 -52.3370

H 4.6280 11.3110 -52.4390

H 4.2860 10.0390 -51.2690

C 5.3090 9.3610 -53.0340

H 5.2620 9.5320 -54.1110

H 5.0370 8.3170 -52.8560

C 6.7320 9.6090 -52.5400

H 6.9910 10.6560 -52.7150

H 6.7730 9.4350 -51.4620

C 7.7660 8.7200 -53.2270

H 7.7270 8.8830 -54.3080

H 7.5230 7.6700 -53.0460

C 9.1770 9.0100 -52.7210

H 9.4110 10.0630 -52.8980

H 9.2090 8.8430 -51.6430

C 10.2400 8.1410 -53.3890

H 9.9980 7.0870 -53.2320

H 10.2340 8.3220 -54.4670

C 11.6340 8.4280 -52.8370

H 11.6340 8.2520 -51.7600

H 11.8770 9.4810 -53.0000

C 12.7030 7.5560 -53.4840

H 12.4860 6.5020 -53.3250

H 13.6810 7.7720 -53.0610

H 12.7480 7.7360 -54.5570

C 3.9520 12.7240 -56.5420

H 3.5470 13.7060 -56.2760

H 4.2700 12.7710 -57.5880

C 5.1290 12.3270 -55.6550

H 5.1690 11.2370 -55.6370

H 4.9600 12.6660 -54.6320

C 6.4570 12.8610 -56.1800

H 6.5580 12.5750 -57.2300

H 6.4620 13.9520 -56.1330

C 7.6490 12.3060 -55.4040

H 7.5440 11.2230 -55.3200

H 7.6540 12.7160 -54.3910

C 8.9780 12.6160 -56.0890

H 8.9490 12.2180 -57.1070

H 9.1130 13.6970 -56.1610

C 10.1610 11.9980 -55.3500

H 9.9850 10.9280 -55.2280

H 10.2360 12.4380 -54.3520

C 11.4790 12.2000 -56.0930

H 11.3970 11.7550 -57.0880

H 11.6660 13.2680 -56.2230

C 12.6530 11.5620 -55.3570

H 12.4510 10.4980 -55.2150

H 12.7490 12.0160 -54.3670

C 13.9690 11.7200 -56.1140

H 13.8730 11.2530 -57.0970

H 14.1720 12.7820 -56.2710

C 15.1390 11.0840 -55.3690

H 14.9320 10.0230 -55.2140

H 15.2350 11.5480 -54.3840

C 16.4570 11.2340 -56.1250

H 16.3680 10.7590 -57.1040

H 16.6600 12.2950 -56.2900

C 17.6200 10.6070 -55.3660

H 17.7450 11.0840 -54.3960

H 18.5470 10.7170 -55.9240

H 17.4400 9.5470 -55.2040

C 2.5220 12.6170 -60.4710

H 2.3090 13.6820 -60.6180

H 1.7030 12.0240 -60.9030

C 3.8210 12.2050 -61.1560

H 3.6970 12.3420 -62.2320

H 3.9580 11.1410 -60.9630

C 5.0640 12.9490 -60.6680

H 5.0390 13.0080 -59.5790

H 5.0690 13.9720 -61.0490

C 6.3200 12.2030 -61.1150

H 6.2480 11.1690 -60.7680

H 6.3550 12.1800 -62.2070

C 7.6250 12.7960 -60.5860

H 7.7780 13.7940 -61.0020

H 7.5660 12.9000 -59.5000

C 8.8050 11.8960 -60.9510

H 8.6330 10.9010 -60.5320

H 8.8490 11.7900 -62.0380

C 10.1500 12.4170 -60.4490

H 10.1090 12.5530 -59.3650

H 10.3530 13.3930 -60.8940

C 11.2800 11.4510 -60.8000

H 11.0780 10.4800 -60.3410

H 11.3020 11.3040 -61.8830

C 12.6480 11.9480 -60.3380

H 12.8670 12.9050 -60.8170

H 12.6280 12.1200 -59.2590

C 13.7550 10.9500 -60.6730

H 13.7640 10.7700 -61.7510

H 13.5430 9.9980 -60.1810

C 15.1330 11.4430 -60.2380

H 15.3590 12.3810 -60.7490

H 15.1210 11.6510 -59.1650

C 16.2230 10.4230 -60.5440

H 16.0220 9.4840 -60.0330

H 17.1940 10.7890 -60.2200

H 16.2720 10.2260 -61.6140

O 2.9460 11.7240 -56.3890

O 0.9220 11.4550 -54.8450

O 2.6470 12.3390 -59.0890

O -3.1550 13.0240 -57.4880

C -4.5850 13.5940 -52.9960

C -3.3980 13.0430 -52.5080

C -5.5700 14.0300 -52.1050

C -3.2110 12.9030 -51.1510

H -2.6370 12.7300 -53.2060

C -5.3800 13.8830 -50.7500

H -6.4690 14.4840 -52.4920

C -4.2050 13.3140 -50.2670

H -2.3010 12.4780 -50.7610

H -6.1310 14.1990 -50.0430

C -6.5310 13.2030 -63.5350

C -5.2750 12.9420 -64.0990

C -7.6660 13.2230 -64.3480

C -5.1600 12.7150 -65.4480

H -4.4080 12.9230 -63.4560

C -7.5450 12.9900 -65.7020

H -8.6340 13.4160 -63.9070

C -6.2940 12.7370 -66.2600

H -4.2040 12.5090 -65.9040

H -8.4140 12.9920 -66.3430

C -9.1060 15.2290 -58.1890

H -9.2180 14.5960 -57.2990

H -8.7340 16.2150 -57.8680

C -2.1950 12.7750 -58.4910

H -2.5090 11.9070 -59.0940

H -2.1280 13.6420 -59.1650

N -2.2080 11.4920 -29.9820

N -2.7590 12.6060 -45.8520

H -2.8220 12.5640 -47.4470

C -7.7700 13.3360 -38.7040

C -7.8380 12.9810 -40.0390

H -6.9440 12.6390 -40.5300

C -9.0380 13.0750 -40.7260

C -10.1880 13.5780 -40.0880

C -10.1220 13.8920 -38.7200

C -8.9060 13.7840 -38.0530

H -8.8390 14.0280 -37.0060

C -8.0990 12.3460 -42.7980

H -7.4650 11.6040 -42.2890

H -7.5050 13.2570 -42.9550

C -8.5410 11.7590 -44.1400

H -8.9210 10.7480 -43.9810

H -7.6450 11.6840 -44.7610

C -9.6090 12.5820 -44.8650

H -9.4660 13.6400 -44.6500

H -9.4900 12.4380 -45.9420

C -11.0230 12.1700 -44.4620

H -11.1370 12.3140 -43.3890

H -11.1580 11.1070 -44.6840

C -12.0940 12.9720 -45.1970

H -12.0710 14.0050 -44.8450

H -11.8790 12.9800 -46.2690

C -13.4840 12.3850 -44.9680

H -13.6450 12.2630 -43.8950

H -13.5350 11.3910 -45.4200

C -14.5970 13.2580 -45.5420

H -14.5360 14.2540 -45.0980

H -14.4580 13.3690 -46.6200

C -15.9780 12.6660 -45.2660

H -16.0860 12.4990 -44.1910

H -16.0590 11.6940 -45.7570

C -17.1090 13.5730 -45.7440

H -17.0020 14.5570 -45.2830

H -17.0350 13.7080 -46.8260

C -18.4810 13.0000 -45.3980

H -18.5870 12.0150 -45.8590

H -18.5520 12.8630 -44.3160

C -19.6200 13.9030 -45.8660

H -19.5580 14.0300 -46.9480

H -19.5050 14.8890 -45.4120

C -20.9850 13.3310 -45.5010

H -21.1330 12.3650 -45.9770

H -21.7790 13.9990 -45.8250

H -21.0670 13.1980 -44.4240

C -12.3360 14.5410 -40.5100

H -11.9220 15.4760 -40.1250

H -12.9380 14.0610 -39.7290

C -13.2150 14.8430 -41.7190

H -13.4490 13.9100 -42.2330

H -12.6610 15.4780 -42.4100

C -14.5030 15.5190 -41.2530

H -14.9200 14.9390 -40.4250

H -14.2700 16.5160 -40.8700

C -15.5710 15.6310 -42.3380

H -15.7620 14.6410 -42.7570

H -15.2150 16.2700 -43.1480

C -16.8700 16.1940 -41.7640

H -17.1580 15.6000 -40.8920

H -16.6990 17.2180 -41.4220

C -18.0200 16.1810 -42.7660

H -18.1960 15.1540 -43.0970

H -17.7460 16.7690 -43.6460

C -19.3050 16.7410 -42.1600

H -19.5450 16.1830 -41.2510

H -19.1430 17.7830 -41.8750

C -20.4850 16.6580 -43.1250

H -20.6690 15.6130 -43.3810

H -20.2350 17.1860 -44.0490

C -21.7560 17.2590 -42.5310

H -21.9860 16.7560 -41.5890

H -21.5860 18.3160 -42.3090

C -22.9480 17.1280 -43.4760

H -23.1400 16.0690 -43.6700

H -22.7050 17.5980 -44.4320

C -24.2090 17.7730 -42.9050

H -24.4350 17.3280 -41.9330

H -24.0270 18.8380 -42.7430

C -25.4060 17.5990 -43.8310

H -25.2090 18.0550 -44.7990

H -26.2910 18.0660 -43.4050

H -25.6180 16.5430 -43.9880

C -11.2360 14.6890 -36.7400

H -10.6650 15.6250 -36.6550

H -10.7630 13.9210 -36.1140

C -12.6710 14.8830 -36.2540

H -12.6350 15.1430 -35.1950

H -13.1760 13.9240 -36.3520

C -13.4700 15.9370 -37.0200

H -13.3310 15.7800 -38.0900

H -13.1070 16.9360 -36.7750

C -14.9560 15.8260 -36.6820

H -15.2790 14.8010 -36.8810

H -15.1000 16.0110 -35.6150

C -15.8480 16.7740 -37.4820

H -15.6680 17.8050 -37.1720

H -15.6040 16.6970 -38.5440

C -17.3210 16.4220 -37.2770

H -17.4900 15.4020 -37.6360

H -17.5460 16.4360 -36.2080

C -18.2860 17.3570 -38.0010

H -18.0290 17.4000 -39.0610

H -18.1920 18.3660 -37.5940

C -19.7290 16.8780 -37.8470

H -19.8280 15.8890 -38.3040

H -19.9580 16.7740 -36.7830

C -20.7450 17.8250 -38.4800

H -20.6760 18.8030 -37.9980

H -20.5080 17.9590 -39.5370

C -22.1700 17.2930 -38.3440

H -22.3940 17.1270 -37.2860

H -22.2440 16.3270 -38.8510

C -23.2070 18.2480 -38.9300

H -23.1520 19.2050 -38.4050

H -22.9730 18.4340 -39.9800

C -24.6200 17.6900 -38.8170

H -24.6960 16.7320 -39.3280

H -25.3390 18.3730 -39.2630

H -24.8910 17.5410 -37.7740

O -11.2840 13.6720 -40.8880

O -9.2260 12.6620 -42.0090

O -11.2780 14.2680 -38.0880

C -10.4670 15.3540 -58.8200

C -10.7860 14.6640 -59.9740

H -10.0140 14.1050 -60.4700

C -12.0860 14.6810 -60.4540

C -13.0930 15.3940 -59.7700

C -12.7460 16.1230 -58.6200

C -11.4310 16.1030 -58.1720

H -11.1530 16.6420 -57.2810

C -11.5130 13.5230 -62.4470

H -10.8180 12.8450 -61.9260

H -10.9380 14.3760 -62.8320

C -12.1540 12.7560 -63.6060

H -12.5200 11.7940 -63.2380

H -11.3540 12.5540 -64.3220

C -13.3050 13.4880 -64.3030

H -13.1230 14.5620 -64.2990

H -13.3470 13.1590 -65.3440

C -14.6440 13.1880 -63.6340

H -14.6280 13.5910 -62.6230

H -14.7650 12.1020 -63.5680

C -15.8370 13.7590 -64.3950

H -15.8120 14.8490 -64.3440

H -15.7710 13.4770 -65.4490

C -17.1520 13.2370 -63.8180

H -17.1890 13.4690 -62.7520

H -17.1770 12.1480 -63.9170

C -18.3820 13.8260 -64.5020

H -18.3800 14.9120 -64.3760

H -18.3350 13.6220 -65.5740

C -19.6780 13.2500 -63.9350

H -19.7060 13.4210 -62.8560

H -19.6940 12.1700 -64.0970

C -20.9170 13.8750 -64.5730

H -20.9040 14.9550 -64.4030

H -20.8860 13.7150 -65.6540

C -22.2120 13.2900 -64.0150

H -22.2390 12.2170 -64.2130

H -22.2330 13.4260 -62.9310

C -23.4480 13.9470 -64.6270

H -23.4250 13.8190 -65.7110

H -23.4220 15.0210 -64.4240

C -24.7410 13.3570 -64.0770

H -24.8090 12.2980 -64.3150

H -25.6050 13.8610 -64.5050

H -24.7820 13.4640 -62.9950

C -15.3640 16.1770 -60.0340

H -15.0580 17.1670 -60.3920

H -15.5720 16.2240 -58.9610

C -16.6160 15.7090 -60.7650

H -16.8170 14.6820 -60.4570

H -16.4290 15.7100 -61.8380

C -17.8230 16.5820 -60.4300

H -17.8880 16.7090 -59.3460

H -17.6960 17.5710 -60.8750

C -19.1180 15.9520 -60.9330

H -19.2420 14.9760 -60.4570

H -19.0460 15.7870 -62.0100

C -20.3460 16.8080 -60.6340

H -20.3730 17.0480 -59.5680

H -20.2760 17.7500 -61.1840

C -21.6320 16.0860 -61.0230

H -21.7190 15.1720 -60.4290

H -21.5740 15.7900 -62.0730

C -22.8780 16.9400 -60.8120

H -22.9140 17.2850 -59.7760

H -22.8250 17.8240 -61.4530

C -24.1490 16.1590 -61.1330

H -24.2200 15.3020 -60.4580

H -24.0830 15.7690 -62.1510

C -25.4110 17.0060 -61.0030

H -25.4630 17.4270 -59.9970

H -25.3620 17.8390 -61.7080

C -26.6690 16.1860 -61.2760

H -26.7290 15.3700 -60.5510

H -26.5990 15.7370 -62.2700

C -27.9430 17.0240 -61.1930

H -28.0120 17.4850 -60.2050

H -27.8930 17.8290 -61.9300

C -29.1890 16.1820 -61.4420

H -29.1490 15.7230 -62.4280

H -30.0840 16.7960 -61.3850

H -29.2680 15.3900 -60.7000

C -13.3960 17.6560 -56.8750

H -12.7840 18.5010 -57.2220

H -12.8320 17.0970 -56.1180

C -14.6980 18.1550 -56.2500

H -14.4530 18.7650 -55.3790

H -15.2570 17.2840 -55.9040

C -15.5610 18.9460 -57.2320

H -15.5930 18.3990 -58.1740

H -15.1080 19.9220 -57.4200

C -16.9900 19.1230 -56.7250

H -17.3930 18.1420 -56.4630

H -16.9920 19.7310 -55.8180

C -17.8990 19.7580 -57.7770

H -17.5970 20.7930 -57.9500

H -17.7830 19.2170 -58.7190

C -19.3670 19.7130 -57.3600

H -19.6430 18.6750 -57.1530

H -19.5050 20.2790 -56.4360

C -20.2970 20.2610 -58.4400

H -20.1000 19.7400 -59.3790

H -20.0880 21.3210 -58.5990

C -21.7670 20.0800 -58.0680

H -21.9630 19.0180 -57.9030

H -21.9720 20.6030 -57.1300

C -22.7090 20.5940 -59.1530

H -22.5370 21.6620 -59.3070

H -22.4850 20.0840 -60.0920

C -24.1740 20.3640 -58.7930

H -24.4010 20.8740 -57.8540

H -24.3410 19.2950 -58.6370

C -25.1220 20.8620 -59.8800

H -24.9540 21.9290 -60.0450

H -24.8980 20.3430 -60.8150

C -26.5820 20.6300 -59.5110

H -26.7730 19.5690 -59.3650

H -27.2400 20.9900 -60.2980

H -26.8310 21.1530 -58.5890

O -14.3380 15.2370 -60.2970

O -12.4990 14.0000 -61.5570

O -13.7310 16.8200 -57.9650

C -4.0940 9.9930 -56.4270

C -5.1350 10.1760 -57.2550

C -5.0670 10.0780 -58.6950

C -6.2060 10.3290 -59.4910

C -3.8880 9.7100 -59.3390

H -3.0440 9.4860 -58.7130

C -5.8130 9.7880 -63.8340

C -4.8060 9.7270 -62.9470

C -4.9130 9.8610 -61.5110

C -3.7720 9.6150 -60.7130

C -6.1050 10.2070 -60.8670

H -6.9700 10.3910 -61.4790

O -2.5930 9.2860 -61.3310

C -9.5890 11.3860 -58.5580

C -9.2430 11.8340 -57.2970

H -8.2110 11.7910 -56.9920

C -10.2180 12.3480 -56.4550

C -11.5500 12.4750 -56.8880

C -11.9060 11.9890 -58.1590

C -10.9130 11.4270 -58.9590

H -11.1630 11.0350 -59.9300

C -8.7870 12.3120 -54.5560

H -7.8920 12.7460 -55.0300

H -8.7330 11.2180 -54.6550

C -8.7810 12.7000 -53.0800

H -8.9050 13.7810 -53.0060

H -7.7940 12.4400 -52.6910

C -9.8520 12.0110 -52.2330

H -9.9520 10.9690 -52.5450

H -9.5170 12.0110 -51.1930

C -11.2110 12.7030 -52.3100

H -11.5780 12.6760 -53.3360

H -11.0870 13.7530 -52.0350

C -12.2240 12.0630 -51.3630

H -12.3800 11.0180 -51.6450

H -11.8220 12.0720 -50.3470

C -13.5620 12.7960 -51.3790

H -13.9630 12.7790 -52.3960

H -13.4050 13.8430 -51.1090

C -14.5820 12.1780 -50.4260

H -14.6830 11.1130 -50.6480

H -14.2220 12.2640 -49.3980

C -15.9500 12.8440 -50.5490

H -16.2480 12.8480 -51.6010

H -15.8810 13.8850 -50.2240

C -17.0270 12.1270 -49.7400

H -17.0490 11.0730 -50.0280

H -16.7800 12.1690 -48.6770

C -18.4070 12.7390 -49.9730

H -18.4170 13.7670 -49.6030

H -18.6050 12.7740 -51.0470

C -19.5130 11.9400 -49.2910

H -19.3240 11.9100 -48.2160

H -19.4880 10.9150 -49.6640

C -20.8920 12.5350 -49.5480

H -20.9490 13.5520 -49.1670

H -21.6630 11.9440 -49.0570

H -21.1050 12.5580 -50.6150

C -13.7380 12.8140 -55.8830

H -13.9150 12.7330 -54.8020

H -13.9750 11.8560 -56.3480

C -14.6300 13.9230 -56.4410

H -14.6020 13.8900 -57.5300

H -14.2230 14.8830 -56.1230

C -16.0590 13.7660 -55.9230

H -16.3860 12.7360 -56.0820

H -16.0700 13.9450 -54.8450

C -17.0560 14.7070 -56.5940

H -17.0890 14.4970 -57.6650

H -16.7270 15.7410 -56.4750

C -18.4520 14.5420 -55.9950

H -18.7290 13.4850 -56.0240

H -18.4300 14.8420 -54.9440

C -19.5210 15.3500 -56.7250

H -19.5430 15.0570 -57.7780

H -19.2700 16.4130 -56.6850

C -20.9010 15.1280 -56.1090

H -21.1170 14.0560 -56.0940

H -20.8910 15.4730 -55.0720

C -22.0110 15.8480 -56.8690

H -22.0150 15.5120 -57.9080

H -21.8120 16.9230 -56.8700

C -23.3810 15.5880 -56.2480

H -23.5650 14.5110 -56.2190

H -23.3830 15.9480 -55.2160

C -24.5070 16.2690 -57.0200

H -24.5100 15.9050 -58.0500

H -24.3260 17.3470 -57.0500

C -25.8740 16.0070 -56.3910

H -26.0500 14.9290 -56.3500

H -25.8750 16.3810 -55.3650

C -26.9980 16.6730 -57.1740

H -26.8440 17.7490 -57.2230

H -27.9590 16.4860 -56.7010

H -27.0360 16.2840 -58.1890

C -13.6150 11.4900 -59.7590

H -13.6170 10.4010 -59.6280

H -12.9300 11.7580 -60.5690

C -14.9970 11.9830 -60.1680

H -15.2140 11.5140 -61.1310

H -14.9400 13.0600 -60.3240

C -16.1210 11.6500 -59.1910

H -16.0970 12.3470 -58.3560

H -15.9650 10.6480 -58.7930

C -17.4750 11.7310 -59.8990

H -17.4810 12.6090 -60.5500

H -17.5930 10.8570 -60.5440

C -18.6740 11.8240 -58.9580

H -18.6690 10.9810 -58.2640

H -18.6010 12.7380 -58.3670

C -19.9830 11.8340 -59.7460

H -19.9380 12.6260 -60.4990

H -20.0860 10.8880 -60.2830

C -21.2190 12.0520 -58.8760

H -21.1360 13.0100 -58.3580

H -21.2740 11.2690 -58.1160

C -22.4940 12.0360 -59.7170

H -22.4240 12.8090 -60.4870

H -22.5700 11.0740 -60.2310

C -23.7600 12.2630 -58.8950

H -23.8330 11.4930 -58.1220

H -23.7010 13.2310 -58.3920

C -25.0110 12.2180 -59.7710

H -25.0570 11.2550 -60.2830

H -24.9410 12.9920 -60.5400

C -26.2970 12.4200 -58.9740

H -26.3630 11.6560 -58.1960

H -26.2710 13.3950 -58.4810

C -27.5310 12.3330 -59.8630

H -27.4980 13.0890 -60.6450

H -28.4370 12.4820 -59.2780

H -27.5890 11.3580 -60.3420

O -12.3590 13.1220 -56.0000

O -9.9610 12.7870 -55.1840

O -13.2030 12.1060 -58.5470

O -7.3270 10.6790 -58.8180

C -4.1070 9.9830 -54.9840

C -2.9680 9.5010 -54.3260

C -5.1790 10.3970 -54.1870

C -2.9550 9.4520 -52.9500

H -2.1190 9.1500 -54.8940

C -5.0590 10.3170 -52.8130

H -6.0790 10.7860 -54.6320

H -2.1030 9.0750 -52.4030

H -5.8520 10.6290 -52.1480

C -5.6970 9.6130 -65.2670

C -6.8590 9.5730 -66.0470

C -4.4840 9.4740 -65.9480

C -6.7580 9.3850 -67.4120

H -7.8280 9.6820 -65.5820

C -4.4970 9.2970 -67.3190

H -3.5450 9.5040 -65.4200

H -7.6390 9.3390 -68.0380

H -3.5760 9.1840 -67.8760

C -1.4930 9.0640 -60.4740

H -1.3530 9.9300 -59.8120

H -1.7000 8.1810 -59.8440

C -8.5330 10.8920 -59.5160

H -8.3830 11.6500 -60.3020

H -8.8600 9.9560 -59.9920

C -4.0900 8.6770 -29.7250

C -3.5940 9.0100 -35.3560

C -3.5340 9.0750 -36.5610

C -3.4430 9.1150 -37.9620

C -4.5950 9.3580 -38.7350

C -2.2140 8.8820 -38.5840

H -1.3650 8.6820 -37.9480

O -3.1950 8.2090 -29.0360

O -5.2330 9.0980 -29.2380

C -4.4130 10.0100 -48.8710

C -3.3970 9.3610 -43.3310

C -3.2140 9.2690 -42.1390

C -3.2260 9.1950 -40.7340

C -2.0860 8.9130 -39.9610

C -4.4570 9.3970 -40.1110

H -5.2940 9.5780 -40.7650

O -5.4540 10.4540 -49.3030

O -3.4120 9.6350 -49.6460

O -0.9310 8.7060 -40.6540

C -8.1780 9.9320 -38.1390

C -8.2090 10.2240 -36.7890

H -7.3020 10.1170 -36.2170

C -9.4000 10.6490 -36.2140

C -10.5800 10.7440 -36.9760

C -10.5310 10.4710 -38.3550

C -9.3270 10.0440 -38.8990

H -9.2660 9.8110 -39.9480

C -8.4780 10.8380 -34.0090

H -7.5250 11.1710 -34.4420

H -8.3990 9.7640 -33.7810

C -8.7190 11.6310 -32.7230

H -8.5490 12.6910 -32.9240

H -7.9670 11.3030 -32.0030

C -10.1220 11.4630 -32.1320

H -10.4790 10.4490 -32.3170

H -10.0690 11.6070 -31.0500

C -11.1110 12.4700 -32.7170

H -11.1990 12.2960 -33.7890

H -10.7150 13.4780 -32.5660

C -12.4930 12.3860 -32.0720

H -12.9570 11.4330 -32.3330

H -12.3980 12.4240 -30.9840

C -13.3830 13.5330 -32.5450

H -13.3650 13.5610 -33.6370

H -12.9720 14.4810 -32.1890

C -14.8320 13.4060 -32.0830

H -15.2140 12.4220 -32.3650

H -14.8810 13.4790 -30.9940

C -15.7120 14.4850 -32.7120

H -15.6360 14.4110 -33.8000

H -15.3390 15.4710 -32.4260

C -17.1780 14.3610 -32.3060

H -17.5140 13.3350 -32.4780

H -17.2800 14.5640 -31.2380

C -18.0680 15.3180 -33.0970

H -17.7110 16.3420 -32.9610

H -17.9890 15.0790 -34.1610

C -19.5320 15.2350 -32.6730

H -19.6250 15.5450 -31.6290

H -19.8680 14.1970 -32.7370

C -20.4250 16.1080 -33.5460

H -20.1100 17.1480 -33.4950

H -21.4610 16.0480 -33.2190

H -20.3730 15.7830 -34.5830

C -12.9520 11.0830 -36.8550

H -13.1030 10.1320 -37.3770

H -13.0170 11.8930 -37.5900

C -14.0420 11.2610 -35.8000

H -13.7480 12.0500 -35.1080

H -14.1570 10.3430 -35.2220

C -15.3500 11.6260 -36.4980

H -15.2190 12.5880 -37.0000

H -15.5570 10.8870 -37.2770

C -16.5580 11.7200 -35.5690

H -16.3200 12.3590 -34.7180

H -16.8050 10.7290 -35.1830

C -17.7550 12.2980 -36.3230

H -17.5330 13.3340 -36.5910

H -17.8920 11.7440 -37.2560

C -19.0580 12.2620 -35.5320

H -18.9220 12.7700 -34.5770

H -19.3290 11.2260 -35.3210

C -20.1850 12.9370 -36.3130

H -19.9310 13.9890 -36.4660

H -20.2640 12.4750 -37.3000

C -21.5360 12.8440 -35.6110

H -21.4640 13.2910 -34.6170

H -21.8050 11.7930 -35.4820

C -22.6300 13.5520 -36.4070

H -22.3740 14.6090 -36.5080

H -22.6750 13.1290 -37.4130

C -24.0020 13.4260 -35.7500

H -23.9580 13.8400 -34.7390

H -24.2650 12.3700 -35.6630

C -25.0850 14.1510 -36.5450

H -24.8260 15.2090 -36.6230

H -25.1200 13.7470 -37.5590

C -26.4580 14.0090 -35.9000

H -26.7440 12.9610 -35.8430

H -27.2100 14.5390 -36.4800

H -26.4510 14.4180 -34.8920

C -11.6610 10.2610 -40.4730

H -11.7050 9.1650 -40.5100

H -10.7620 10.5990 -40.9960

C -12.8610 10.8870 -41.1870

H -13.0110 10.3290 -42.1140

H -12.5960 11.9110 -41.4470

C -14.1500 10.9050 -40.3720

H -14.0140 11.5680 -39.5180

H -14.3650 9.9080 -39.9860

C -15.3290 11.4000 -41.2050

H -15.0630 12.3510 -41.6700

H -15.5340 10.6860 -42.0070

C -16.5890 11.5990 -40.3680

H -16.8510 10.6610 -39.8730

H -16.3890 12.3370 -39.5870

C -17.7670 12.0690 -41.2160

H -17.4980 13.0010 -41.7190

H -17.9750 11.3230 -41.9870

C -19.0270 12.2960 -40.3850

H -18.8300 13.0590 -39.6270

H -19.2860 11.3720 -39.8610

C -20.2070 12.7320 -41.2480

H -19.9510 13.6600 -41.7650

H -20.3970 11.9700 -42.0080

C -21.4760 12.9470 -40.4280

H -21.7280 12.0200 -39.9060

H -21.2900 13.7110 -39.6680

C -22.6560 13.3730 -41.2960

H -22.8310 12.6160 -42.0640

H -22.4120 14.3090 -41.8040

C -23.9310 13.5610 -40.4790

H -24.1770 12.6220 -39.9770

H -23.7560 14.3130 -39.7050

C -25.1070 13.9910 -41.3480

H -24.8910 14.9340 -41.8430

H -26.0040 14.1170 -40.7460

H -25.3090 13.2430 -42.1110

O -11.6760 11.1170 -36.2460

O -9.5380 11.0470 -34.9140

O -11.6440 10.6760 -39.1150

O -5.7690 9.5360 -38.0750

C -3.7070 8.9340 -33.9560

C -4.9120 9.2870 -33.3410

C -2.6350 8.5000 -33.1700

C -5.0420 9.2190 -31.9740

H -5.7370 9.6110 -33.9570

C -2.7720 8.4230 -31.8030

H -1.7040 8.2230 -33.6390

C -3.9710 8.7870 -31.1930

H -5.9680 9.4920 -31.4910

H -1.9570 8.0880 -31.1800

C -3.6340 9.4960 -44.7120

C -4.8810 9.9540 -45.1480

C -2.6450 9.1910 -45.6490

C -5.1240 10.1200 -46.4930

H -5.6460 10.1720 -44.4170

C -2.8950 9.3550 -46.9940

H -1.6930 8.8180 -45.3070

C -4.1320 9.8270 -47.4240

H -6.0780 10.4750 -46.8520

H -2.1430 9.1130 -47.7280

C 0.1890 8.1710 -39.9880

H 0.4660 8.7840 -39.1180

H -0.0380 7.1590 -39.6140

C -6.9220 9.5890 -38.8880

H -6.7990 10.3690 -39.6520

H -7.0520 8.6220 -39.3960

N -3.9750 9.8580 -52.2090

N -5.6030 9.2450 -68.0440

H -3.6660 9.7570 -50.6180

C -0.1870 8.8280 -61.1830

C -0.0900 8.3110 -62.4610

H -0.9900 8.1070 -63.0150

C 1.1640 8.0840 -63.0140

C 2.3430 8.3780 -62.2940

C 2.2300 8.8490 -60.9730

C 0.9610 9.0750 -60.4500

H 0.8570 9.4490 -59.4440

C 0.2830 6.8720 -64.8580

H -0.4490 7.5920 -65.2520

H -0.2190 6.2360 -64.1150

C 0.8080 6.0030 -65.9980

H 1.2500 6.6450 -66.7600

H -0.0560 5.5020 -66.4380

C 1.8460 4.9720 -65.5370

H 1.6530 4.6950 -64.4990

H 1.7470 4.0670 -66.1410

C 3.2750 5.4960 -65.6690

H 3.3640 6.4380 -65.1270

H 3.4750 5.6940 -66.7260

C 4.3100 4.4980 -65.1470

H 4.3230 4.5270 -64.0550

H 4.0260 3.4850 -65.4440

C 5.7070 4.7990 -65.6880

H 5.9420 5.8530 -65.5250

H 5.7140 4.6290 -66.7680

C 6.7840 3.9340 -65.0380

H 6.8120 4.1390 -63.9650

H 6.5250 2.8800 -65.1660

C 8.1660 4.1900 -65.6380

H 8.4110 5.2500 -65.5450

H 8.1440 3.9540 -66.7050

C 9.2510 3.3570 -64.9600

H 9.2630 3.5840 -63.8900

H 9.0110 2.2970 -65.0710

C 10.6370 3.6210 -65.5440

H 10.6290 3.3960 -66.6130

H 10.8810 4.6810 -65.4350

C 11.7140 2.7820 -64.8590

H 11.4830 1.7230 -64.9960

H 11.7020 2.9890 -63.7870

C 13.1050 3.0680 -65.4130

H 13.1500 2.8340 -66.4740

H 13.8520 2.4690 -64.8970

H 13.3600 4.1190 -65.2860

C 4.7410 8.4660 -62.4350

H 4.8900 7.9210 -61.4990

H 4.7800 9.5410 -62.2290

C 5.8610 8.0870 -63.3950

H 5.7830 8.6690 -64.3130

H 5.7580 7.0330 -63.6550

C 7.2030 8.3310 -62.7070

H 7.3270 9.4000 -62.5230

H 7.1880 7.8300 -61.7350

C 8.4020 7.8080 -63.4910

H 8.5050 8.3560 -64.4300

H 8.2390 6.7570 -63.7380

C 9.6820 7.9390 -62.6680

H 9.8660 8.9930 -62.4480

H 9.5410 7.4230 -61.7150

C 10.9010 7.3460 -63.3680

H 11.0940 7.8910 -64.2950

H 10.6900 6.3070 -63.6350

C 12.1390 7.3940 -62.4770

H 12.3560 8.4320 -62.2120

H 11.9320 6.8520 -61.5510

C 13.3600 6.7750 -63.1510

H 13.5970 7.3340 -64.0600

H 13.1240 5.7500 -63.4460

C 14.5780 6.7640 -62.2300

H 14.8120 7.7870 -61.9270

H 14.3380 6.1960 -61.3280

C 15.8000 6.1430 -62.9010

H 16.0590 6.7230 -63.7910

H 15.5540 5.1300 -63.2310

C 17.0070 6.0880 -61.9670

H 17.2540 7.1000 -61.6370

H 16.7500 5.5030 -61.0810

C 18.2220 5.4670 -62.6440

H 18.0040 4.4490 -62.9590

H 19.0690 5.4410 -61.9620

H 18.5060 6.0430 -63.5220

C 3.3000 9.1210 -58.8200

H 3.0690 8.1180 -58.4350

H 2.5260 9.8120 -58.4790

C 4.6280 9.6250 -58.2680

H 4.5060 9.7110 -57.1880

H 4.8010 10.6240 -58.6660

C 5.8200 8.7320 -58.5900

H 5.9240 8.6570 -59.6740

H 5.6410 7.7290 -58.1940

C 7.1220 9.2740 -58.0050

H 7.2710 10.3030 -58.3370

H 7.0540 9.2850 -56.9150

C 8.3120 8.4210 -58.4340

H 8.1430 7.3900 -58.1170

H 8.3800 8.4290 -59.5260

C 9.6420 8.8980 -57.8560

H 9.8200 9.9350 -58.1480

H 9.5950 8.8660 -56.7650

C 10.7910 8.0200 -58.3440

H 10.8500 8.0790 -59.4340

H 10.5800 6.9810 -58.0790

C 12.1400 8.4140 -57.7500

H 12.3620 9.4530 -58.0010

H 12.0870 8.3440 -56.6610

C 13.2590 7.5120 -58.2640

H 13.0080 6.4710 -58.0460

H 13.3320 7.6140 -59.3500

C 14.6120 7.8370 -57.6380

H 14.5520 7.7020 -56.5550

H 14.8560 8.8850 -57.8250

C 15.7220 6.9500 -58.1950

H 15.4700 5.9030 -58.0130

H 15.7860 7.0910 -59.2770

C 17.0740 7.2600 -57.5660

H 17.3560 8.2930 -57.7520

H 17.8450 6.6140 -57.9790

H 17.0370 7.1050 -56.4890

O 3.4860 8.1240 -62.9920

O 1.3620 7.5550 -64.2490

O 3.3680 9.0750 -60.2390

C 1.3710 8.1290 -40.9260

C 1.2410 8.5260 -42.2450

C 2.3580 8.5490 -43.0770

C 3.6040 8.1700 -42.5650

C 3.7240 7.7280 -41.2340

C 2.5930 7.7100 -40.4240

H 2.6470 7.3640 -39.4050

C 1.0900 9.1100 -45.0130

H 0.4670 9.8170 -44.4490

H 0.5710 8.1400 -45.0460

C 1.2710 9.6520 -46.4290

H 1.7010 10.6540 -46.3700

H 0.2720 9.7380 -46.8590

C 2.1570 8.7800 -47.3260

H 2.1010 7.7410 -46.9960

H 1.7820 8.8200 -48.3510

C 3.6120 9.2450 -47.3100

H 3.9610 9.2720 -46.2780

H 3.6610 10.2610 -47.7110

C 4.5270 8.3410 -48.1330

H 4.5790 7.3590 -47.6570

H 4.1090 8.2010 -49.1330

C 5.9340 8.9270 -48.2420

H 6.2330 9.3080 -47.2630

H 5.9260 9.7760 -48.9290

C 6.9690 7.9030 -48.7010

H 6.9060 7.0240 -48.0570

H 6.7480 7.5820 -49.7210

C 8.3880 8.4640 -48.6290

H 8.5590 8.8620 -47.6260

H 8.4900 9.2950 -49.3330

C 9.4490 7.4080 -48.9250

H 9.3170 6.5670 -48.2390

H 9.3170 7.0260 -49.9400

C 10.8620 7.9640 -48.7660

H 11.0080 8.7870 -49.4710

H 10.9750 8.3720 -47.7590

C 11.9330 6.9020 -48.9980

H 11.8100 6.4710 -49.9940

H 11.8010 6.0950 -48.2730

C 13.3380 7.4770 -48.8650

H 13.5010 8.2600 -49.6030

H 14.0870 6.7030 -49.0180

H 13.4820 7.9050 -47.8750

C 4.9460 7.0280 -44.0990

H 4.1020 6.8550 -44.7830

H 5.0150 6.1790 -43.4060

C 6.2410 7.1730 -44.8860

H 6.2080 8.1230 -45.4170

H 6.2950 6.3670 -45.6200

C 7.4700 7.1380 -43.9850

H 7.3520 7.8900 -43.2030

H 7.5350 6.1580 -43.5050

C 8.7540 7.4040 -44.7670

H 8.7480 8.4320 -45.1350

H 8.7910 6.7420 -45.6360

C 9.9980 7.1770 -43.9140

H 9.9550 7.8230 -43.0340

H 10.0060 6.1400 -43.5670

C 11.2910 7.4540 -44.6770

H 11.3330 8.5110 -44.9500

H 11.2980 6.8720 -45.6020

C 12.5200 7.0930 -43.8480

H 12.5040 7.6600 -42.9140

H 12.4770 6.0310 -43.5930

C 13.8280 7.3750 -44.5820

H 13.8990 8.4430 -44.8000

H 13.8310 6.8410 -45.5350

C 15.0400 6.9430 -43.7600

H 15.0240 7.4590 -42.7980

H 14.9720 5.8700 -43.5610

C 16.3590 7.2380 -44.4680

H 16.4380 8.3120 -44.6530

H 16.3700 6.7370 -45.4390

C 17.5610 6.7770 -43.6470

H 17.5430 7.2740 -42.6760

H 17.4850 5.7020 -43.4710

C 18.8800 7.0810 -44.3460

H 18.9330 6.5680 -45.3030

H 19.7210 6.7580 -43.7360

H 18.9800 8.1490 -44.5280

C 5.2130 6.9970 -39.5030

H 5.0030 5.9270 -39.3780

H 4.5680 7.5670 -38.8270

C 6.6530 7.3480 -39.1420

H 6.7600 7.2030 -38.0670

H 6.7850 8.4110 -39.3450

C 7.7270 6.5510 -39.8760

H 7.6210 6.6960 -40.9520

H 7.6010 5.4870 -39.6680

C 9.1180 6.9960 -39.4230

H 9.2740 8.0350 -39.7200

H 9.1640 6.9580 -38.3320

C 10.2460 6.1390 -39.9910

H 10.1160 5.1060 -39.6630

H 10.2020 6.1480 -41.0820

C 11.6140 6.6430 -39.5340

H 11.7730 7.6520 -39.9200

H 11.6310 6.7050 -38.4440

C 12.7540 5.7410 -40.0020

H 12.7180 5.6450 -41.0890

H 12.6200 4.7430 -39.5790

C 14.1210 6.2820 -39.5900

H 14.2760 7.2590 -40.0560

H 14.1430 6.4290 -38.5080

C 15.2550 5.3460 -39.9970

H 15.1220 4.3820 -39.5010

H 15.2100 5.1720 -41.0740

C 16.6270 5.9120 -39.6380

H 16.6600 6.1340 -38.5690

H 16.7800 6.8510 -40.1750

C 17.7560 4.9470 -39.9880

H 17.6170 4.0150 -39.4340

H 17.7080 4.7070 -41.0520

C 19.1250 5.5310 -39.6630

H 19.3100 6.4210 -40.2620

H 19.9110 4.8110 -39.8750

H 19.1840 5.8060 -38.6130

O 4.7240 8.2260 -43.3670

O 2.3420 8.9410 -44.3800

O 4.9650 7.3610 -40.8530

H -5.3100 8.9270 -28.2490

H -1.5860 11.8940 -41.3690

H -4.4320 12.9400 -40.9700

H -1.2890 11.5080 -34.9890

H -4.0760 12.5200 -34.2460

H -3.1150 9.7840 -56.8350

H -6.1150 10.3900 -56.8570

H -3.8020 9.5360 -63.2940

H -6.8260 9.9510 -63.4900

H 0.2660 8.8130 -42.5990

C -4.9130 16.9130 -57.6930

C -4.0260 16.5640 -58.6400

C -4.2760 16.5180 -60.0630

C -3.2020 16.2810 -60.9490

C -5.5540 16.6650 -60.6010

H -6.3700 16.8310 -59.9210

C -4.1670 15.8250 -65.2260

C -5.0160 16.1980 -64.2550

C -4.7290 16.3290 -62.8440

C -5.8040 16.5730 -61.9580

C -3.4460 16.1970 -62.3080

H -2.6300 16.0200 -62.9890

C -9.4330 16.9310 -62.4020

C -10.5360 17.4900 -61.7730

H -10.4380 17.8530 -60.7630

C -11.7520 17.5650 -62.4370

C -11.8680 17.0560 -63.7400

C -10.7520 16.4850 -64.3690

C -9.5370 16.4360 -63.6890

H -8.6590 16.0050 -64.1390

C -12.7290 19.0210 -60.8270

H -12.5970 18.4410 -59.9050

H -11.8400 19.6490 -60.9660

C -13.9870 19.8930 -60.7580

H -14.4850 19.8250 -61.7270

H -14.6690 19.4940 -60.0080

C -13.6840 21.3580 -60.4450

H -13.0200 21.4150 -59.5790

H -14.6180 21.8580 -60.1760

C -13.0560 22.0960 -61.6250

H -12.1290 21.6040 -61.9240

H -13.7380 22.0490 -62.4770

C -12.7620 23.5570 -61.2970

H -12.0970 23.6080 -60.4310

H -13.6930 24.0600 -61.0260

C -12.1160 24.2910 -62.4700

H -11.1620 23.8180 -62.7130

H -12.7580 24.2030 -63.3490

C -11.8870 25.7680 -62.1600

H -11.2600 25.8570 -61.2690

H -12.8470 26.2390 -61.9330

C -11.2260 26.5110 -63.3180

H -10.2470 26.0700 -63.5190

H -11.8340 26.3910 -64.2180

C -11.0620 27.9980 -63.0150

H -10.4730 28.1170 -62.1010

H -12.0450 28.4370 -62.8310

C -10.3800 28.7500 -64.1540

H -10.9560 28.6120 -65.0720

H -9.3850 28.3300 -64.3170

C -10.2550 30.2430 -63.8600

H -11.2520 30.6610 -63.7000

H -9.6870 30.3820 -62.9370

C -9.5700 30.9950 -64.9950

H -10.1280 30.8770 -65.9210

H -9.5050 32.0560 -64.7670

H -8.5620 30.6150 -65.1500

C -13.1770 18.0140 -65.4530

H -12.4650 17.7230 -66.2350

H -14.1910 17.8860 -65.8400

C -12.9400 19.4680 -65.0410

H -13.6990 19.7550 -64.3110

H -11.9660 19.5390 -64.5470

C -12.9710 20.4150 -66.2360

H -13.9350 20.3300 -66.7440

H -12.1960 20.1220 -66.9470

C -12.7450 21.8660 -65.8160

H -13.5520 22.1790 -65.1490

H -11.8100 21.9390 -65.2540

C -12.6840 22.8080 -67.0150

H -13.6220 22.7460 -67.5720

H -11.8820 22.4850 -67.6830

C -12.4360 24.2560 -66.6010

H -13.2430 24.5880 -65.9430

H -11.5030 24.3130 -66.0340

C -12.3510 25.1900 -67.8050

H -13.2990 25.1690 -68.3480

H -11.5760 24.8280 -68.4860

C -12.0290 26.6250 -67.3990

H -12.8000 26.9890 -66.7140

H -11.0760 26.6410 -66.8650

C -11.9450 27.5600 -68.6020

H -12.9050 27.5670 -69.1240

H -11.1960 27.1810 -69.3020

C -11.5790 28.9860 -68.1970

H -12.3280 29.3660 -67.4980

H -10.6170 28.9750 -67.6790

C -11.4920 29.9230 -69.3980

H -12.4540 29.9360 -69.9160

H -10.7470 29.5420 -70.1010

C -11.1200 31.3430 -68.9870

H -10.1490 31.3580 -68.4960

H -11.0730 31.9940 -69.8570

H -11.8590 31.7460 -68.2970

C -9.9140 15.7930 -66.5310

H -8.9830 15.5100 -66.0340

H -10.2290 14.9600 -67.1670

C -9.6990 17.0360 -67.4000

H -8.9440 16.7960 -68.1510

H -10.6320 17.2590 -67.9230

C -9.2640 18.2650 -66.6060

H -10.0230 18.5030 -65.8580

H -8.3340 18.0560 -66.0710

C -9.0650 19.4750 -67.5150

H -9.9650 19.6220 -68.1190

H -8.2380 19.2820 -68.2020

C -8.7830 20.7490 -66.7230

H -7.8790 20.6140 -66.1250

H -9.6110 20.9330 -66.0340

C -8.6130 21.9590 -67.6370

H -9.5070 22.0710 -68.2560

H -7.7690 21.7880 -68.3100

C -8.3820 23.2510 -66.8570

H -9.2230 23.4190 -66.1810

H -7.4820 23.1480 -66.2450

C -8.2310 24.4520 -67.7850

H -9.1350 24.5550 -68.3890

H -7.3970 24.2750 -68.4690

C -7.9860 25.7530 -67.0250

H -7.0830 25.6530 -66.4170

H -8.8210 25.9380 -66.3440

C -7.8310 26.9400 -67.9720

H -6.9940 26.7510 -68.6500

H -8.7330 27.0340 -68.5810

C -7.5900 28.2500 -67.2280

H -6.6980 28.1540 -66.6040

H -8.4360 28.4470 -66.5660

C -7.4130 29.4210 -68.1870

H -8.2890 29.5290 -68.8220

H -7.2690 30.3500 -67.6390

H -6.5470 29.2630 -68.8270

O -13.0980 17.1210 -64.3550

O -12.8660 18.1490 -61.9320

O -10.9730 15.9650 -65.6100

O -7.0410 16.6920 -62.5060

C 0.4190 16.0430 -60.3590

C 0.3740 15.7350 -59.0100

H -0.5740 15.5590 -58.5340

C 1.5530 15.6260 -58.2860

C 2.8030 15.8020 -58.9120

C 2.8350 16.1610 -60.2690

C 1.6390 16.2780 -60.9730

H 1.6550 16.5140 -62.0250

C 0.4960 15.5340 -56.1300

H -0.1310 14.6340 -56.1220

H -0.0970 16.3840 -56.4880

C 1.0520 15.8380 -54.7390

H 1.8060 15.0820 -54.5150

H 0.2580 15.7660 -53.9950

C 1.6900 17.2290 -54.7220

H 2.1660 17.3990 -55.6900

H 0.9110 17.9850 -54.5960

C 2.7460 17.4000 -53.6340

H 3.5660 16.7040 -53.8240

H 2.3190 17.1550 -52.6600

C 3.2870 18.8300 -53.6150

H 3.4950 19.1480 -54.6400

H 2.5220 19.4990 -53.2150

C 4.5640 18.9690 -52.7880

H 5.3610 18.3860 -53.2570

H 4.3990 18.5590 -51.7900

C 5.0030 20.4270 -52.6710

H 5.0230 20.8790 -53.6660

H 4.2680 20.9760 -52.0780

C 6.3810 20.5710 -52.0280

H 7.1310 20.1140 -52.6780

H 6.3960 20.0320 -51.0780

C 6.7460 22.0330 -51.7840

H 6.6360 22.5940 -52.7160

H 6.0460 22.4620 -51.0620

C 8.1720 22.1950 -51.2640

H 8.3020 21.5850 -50.3670

H 8.8730 21.8280 -52.0170

C 8.5030 23.6490 -50.9350

H 7.8320 24.0020 -50.1490

H 8.3250 24.2670 -51.8190

C 9.9480 23.8160 -50.4830

H 10.1390 23.2240 -49.5910

H 10.1620 24.8580 -50.2560

H 10.6310 23.4890 -51.2640

C 5.0940 16.1910 -58.1950

H 5.4590 16.2660 -59.2220

H 5.7840 15.5630 -57.6230

C 5.0090 17.5750 -57.5480

H 4.5070 17.4750 -56.5840

H 4.4050 18.2370 -58.1740

C 6.4020 18.1630 -57.3450

H 6.9770 17.4940 -56.7000

H 6.9180 18.2150 -58.3060

C 6.3670 19.5520 -56.7130

H 5.8050 19.5110 -55.7780

H 5.8490 20.2460 -57.3800

C 7.7760 20.0700 -56.4310

H 8.2660 19.3990 -55.7220

H 8.3590 20.0520 -57.3550

C 7.7770 21.4860 -55.8640

H 7.1580 21.5170 -54.9650

H 7.3330 22.1700 -56.5920

C 9.1890 21.9540 -55.5210

H 9.6080 21.2950 -54.7570

H 9.8220 21.8730 -56.4070

C 9.2160 23.3930 -55.0120

H 8.5540 23.4830 -54.1490

H 8.8320 24.0590 -55.7890

C 10.6230 23.8310 -54.6180

H 10.9940 23.1760 -53.8240

H 11.2910 23.7150 -55.4740

C 10.6640 25.2780 -54.1370

H 9.9940 25.3960 -53.2830

H 10.3000 25.9340 -54.9320

C 12.0710 25.7110 -53.7330

H 12.4270 25.0670 -52.9250

H 12.7470 25.5820 -54.5810

C 12.1080 27.1630 -53.2750

H 11.7780 27.8230 -54.0750

H 13.1170 27.4500 -52.9880

H 11.4520 27.3100 -52.4190

C 4.1560 16.9190 -62.1370

H 3.4490 17.7490 -62.2560

H 3.9310 16.1500 -62.8900

C 5.5850 17.4330 -62.3000

H 5.7770 17.6280 -63.3560

H 6.2700 16.6520 -61.9650

C 5.8060 18.7030 -61.4800

H 5.4410 18.5340 -60.4660

H 5.2200 19.5160 -61.9150

C 7.2740 19.1180 -61.4170

H 7.8480 18.3390 -60.9100

H 7.6720 19.2130 -62.4300

C 7.4480 20.4420 -60.6770

H 6.9500 21.2360 -61.2390

H 6.9620 20.3760 -59.7010

C 8.9160 20.8120 -60.4780

H 9.4020 20.0460 -59.8690

H 9.4210 20.8300 -61.4470

C 9.0710 22.1720 -59.8020

H 8.5140 22.1730 -58.8620

H 8.6360 22.9430 -60.4420

C 10.5310 22.5160 -59.5190

H 10.9540 21.7650 -58.8460

H 11.0990 22.4780 -60.4510

C 10.6800 23.9000 -58.8920

H 10.2940 24.6520 -59.5840

H 10.0780 23.9500 -57.9820

C 12.1320 24.2250 -58.5520

H 12.7400 24.1570 -59.4570

H 12.5120 23.4830 -57.8450

C 12.2820 25.6190 -57.9480

H 11.9280 26.3630 -58.6650

H 11.6540 25.6950 -57.0580

C 13.7280 25.9240 -57.5740

H 14.0980 25.1950 -56.8550

H 13.8090 26.9140 -57.1320

H 14.3660 25.8870 -58.4540

O 3.8710 15.4880 -58.1250

O 1.6220 15.3320 -56.9660

O 4.0590 16.3630 -60.8410

O -1.9720 16.1460 -60.3760

C -4.6920 16.8890 -56.2640

C -5.7750 17.1460 -55.4130

C -3.4680 16.5970 -55.6520

C -5.5950 17.0890 -54.0480

H -6.7470 17.3810 -55.8220

C -3.3890 16.5820 -54.2730

H -2.5910 16.3820 -56.2390

H -6.4160 17.2660 -53.3670

H -2.4640 16.3600 -53.7580

C -4.5140 15.6190 -66.6180

C -3.5530 15.1050 -67.4960

C -5.7800 15.8700 -67.1570

C -3.8920 14.8660 -68.8150

H -2.5560 14.8850 -67.1440

C -6.0090 15.5940 -68.4920

H -6.5750 16.2710 -66.5490

H -3.1710 14.4600 -69.5120

H -6.9810 15.7670 -68.9360

C -8.1420 16.8460 -61.6330

H -8.1960 15.9880 -60.9490

H -8.0240 17.7640 -61.0390

C -0.8330 16.0770 -61.2010

H -0.8680 15.1630 -61.8190

H -0.8020 16.9430 -61.8770

C -0.7490 14.2750 -31.3640

C -0.9650 15.0090 -36.9750

C -1.0700 15.1320 -38.1740

C -1.3080 15.2620 -39.5530

C -0.2800 15.1030 -40.5010

C -2.6130 15.5290 -39.9700

H -3.3610 15.6300 -39.2000

O -1.6890 14.5940 -30.6480

O 0.3520 13.7260 -30.9070

C -3.0140 16.2540 -50.4520

C -2.3110 15.7670 -44.8350

C -2.1580 15.6500 -43.6400

C -1.9040 15.5130 -42.2610

C -2.9390 15.6380 -41.3110

C -0.5980 15.2430 -41.8420

H 0.1550 15.1440 -42.6090

O -2.1640 15.9080 -51.2460

O -4.1750 16.7690 -50.8210

C -6.5810 16.0670 -41.5240

C -7.6680 16.4760 -40.7660

H -7.5330 16.6760 -39.7150

C -8.9100 16.6170 -41.3620

C -9.0920 16.3230 -42.7270

C -7.9860 15.9130 -43.4850

C -6.7410 15.7880 -42.8660

H -5.8750 15.4750 -43.4240

C -9.9050 17.5590 -39.4080

H -9.7060 16.7270 -38.7170

H -9.0710 18.2710 -39.3400

C -11.2150 18.2530 -39.0380

H -12.0340 17.6770 -39.4700

H -11.3210 18.2360 -37.9520

C -11.3020 19.7020 -39.5180

H -10.5040 20.2840 -39.0490

H -12.2530 20.1160 -39.1750

C -11.2120 19.8570 -41.0350

H -10.2210 19.5580 -41.3770

H -11.9350 19.1910 -41.5100

C -11.4820 21.3000 -41.4560

H -10.8070 21.9650 -40.9110

H -12.5030 21.5720 -41.1760

C -11.2970 21.5250 -42.9550

H -10.2620 21.3000 -43.2260

H -11.9420 20.8380 -43.5070

C -11.6230 22.9610 -43.3590

H -11.0160 23.6490 -42.7660

H -12.6710 23.1690 -43.1320

C -11.3670 23.2190 -44.8420

H -10.3100 23.0480 -45.0590

H -11.9470 22.5110 -45.4380

C -11.7380 24.6430 -45.2520

H -11.1830 25.3530 -44.6330

H -12.8020 24.8070 -45.0650

C -11.4360 24.9140 -46.7230

H -11.9710 24.1890 -47.3410

H -10.3670 24.7770 -46.9030

C -11.8370 26.3260 -47.1460

H -12.9080 26.4600 -46.9810

H -11.3130 27.0520 -46.5200

C -11.5120 26.5930 -48.6100

H -12.0290 25.8830 -49.2510

H -11.8160 27.5980 -48.8950

H -10.4430 26.4970 -48.7870

C -10.7050 16.7900 -44.4750

H -10.3580 16.0370 -45.1890

H -11.8000 16.8050 -44.4820

C -10.1730 18.1690 -44.8640

H -10.3490 18.8600 -44.0380

H -9.0960 18.1060 -45.0270

C -10.8630 18.6790 -46.1260

H -11.9070 18.9060 -45.9000

H -10.8590 17.8890 -46.8820

C -10.1850 19.9210 -46.6970

H -10.1260 20.6900 -45.9250

H -9.1660 19.6690 -46.9950

C -10.9430 20.4700 -47.9030

H -11.9270 20.8200 -47.5830

H -11.1030 19.6640 -48.6250

C -10.1970 21.6110 -48.5880

H -10.0090 22.4060 -47.8630

H -9.2300 21.2470 -48.9410

C -10.9830 22.1770 -49.7670

H -11.9300 22.5880 -49.4060

H -11.2230 21.3690 -50.4620

C -10.2100 23.2640 -50.5080

H -9.9510 24.0620 -49.8090

H -9.2790 22.8430 -50.8930

C -11.0150 23.8500 -51.6650

H -11.9320 24.3000 -51.2780

H -11.3080 23.0470 -52.3450

C -10.2260 24.9010 -52.4410

H -9.9110 25.6930 -51.7580

H -9.3250 24.4400 -52.8530

C -11.0430 25.5120 -53.5760

H -11.9220 26.0100 -53.1610

H -11.3960 24.7160 -54.2360

C -10.2290 26.5130 -54.3870

H -9.3650 26.0270 -54.8340

H -10.8320 26.9430 -55.1840

H -9.8760 27.3210 -53.7500

C -7.0680 15.2540 -45.5970

H -6.2200 15.9280 -45.4320

H -6.7700 14.2380 -45.3110

C -7.4420 15.2850 -47.0730

H -6.6090 14.8350 -47.6140

H -8.3310 14.6750 -47.2320

C -7.6860 16.7070 -47.5730

H -8.6360 17.0710 -47.1790

H -6.8990 17.3620 -47.1910

C -7.6980 16.7920 -49.0960

H -8.4390 16.0950 -49.4950

H -6.7210 16.4930 -49.4810

C -8.0170 18.2040 -49.5810

H -7.3540 18.9160 -49.0820

H -9.0430 18.4600 -49.3070

C -7.8430 18.3400 -51.0900

H -8.4560 17.5850 -51.5900

H -6.7970 18.1460 -51.3380

C -8.2410 19.7240 -51.5960

H -9.2740 19.9290 -51.3040

H -7.6080 20.4780 -51.1220

C -8.1200 19.8460 -53.1130

H -8.7320 19.0720 -53.5850

H -7.0830 19.6750 -53.4110

C -8.5700 21.2150 -53.6170

H -7.9280 21.9860 -53.1860

H -9.5900 21.4070 -53.2770

C -8.5240 21.3130 -55.1400

H -7.5060 21.1130 -55.4860

H -9.1750 20.5470 -55.5700

C -8.9680 22.6860 -55.6380

H -8.2990 23.4480 -55.2340

H -9.9720 22.8960 -55.2630

C -8.9700 22.7720 -57.1590

H -9.6490 22.0350 -57.5830

H -9.2880 23.7590 -57.4870

H -7.9730 22.5840 -57.5540

O -10.3880 16.4260 -43.1470

O -10.0260 17.0610 -40.7220

O -8.1770 15.6600 -44.8130

O -4.1950 15.8410 -41.7830

C 3.3560 14.5990 -40.1770

C 3.4170 14.3460 -38.8180

H 2.5080 14.3600 -38.2420

C 4.6440 14.0790 -38.2230

C 5.8280 14.0740 -38.9840

C 5.7600 14.3880 -40.3500

C 4.5200 14.6340 -40.9310

H 4.4470 14.8300 -41.9890

C 3.7250 13.7850 -36.0380

H 2.9850 13.0660 -36.4080

H 3.2660 14.7830 -35.9870

C 4.2180 13.3500 -34.6600

H 4.9540 12.5600 -34.8090

H 3.3740 12.9310 -34.1100

C 4.8280 14.4830 -33.8350

H 4.0610 15.2370 -33.6400

H 5.1400 14.0750 -32.8700

C 6.0290 15.1460 -34.5060

H 5.7020 15.6630 -35.4090

H 6.7440 14.3800 -34.8110

C 6.7040 16.1450 -33.5680

H 5.9530 16.8410 -33.1850

H 7.1200 15.6110 -32.7100

C 7.8130 16.9400 -34.2520

H 7.3860 17.5140 -35.0780

H 8.5510 16.2510 -34.6710

C 8.5030 17.8940 -33.2790

H 7.7490 18.5260 -32.8040

H 8.9890 17.3140 -32.4910

C 9.5390 18.7840 -33.9620

H 9.0450 19.3920 -34.7240

H 10.2820 18.1600 -34.4650

C 10.2410 19.7020 -32.9630

H 9.4900 20.2860 -32.4240

H 10.7730 19.0940 -32.2280

C 11.2250 20.6540 -33.6370

H 11.9660 20.0760 -34.1950

H 10.6870 21.2810 -34.3520

C 11.9400 21.5470 -32.6260

H 12.4960 20.9220 -31.9240

H 11.1970 22.1060 -32.0510

C 12.8960 22.5230 -33.3000

H 13.6480 21.9860 -33.8730

H 13.4020 23.1390 -32.5600

H 12.3530 23.1800 -33.9780

C 8.2010 14.2430 -38.5250

H 8.5450 14.0780 -39.5500

H 8.8590 13.6980 -37.8410

C 8.2380 15.7330 -38.1890

H 7.7770 15.8830 -37.2110

H 7.6490 16.2860 -38.9250

C 9.6760 16.2410 -38.1730

H 10.2330 15.7050 -37.4010

H 10.1490 16.0170 -39.1330

C 9.7710 17.7400 -37.9030

H 9.2520 17.9740 -36.9720

H 9.2720 18.2900 -38.7060

C 11.2250 18.1930 -37.8000

H 11.7020 17.6690 -36.9680

H 11.7570 17.9110 -38.7120

C 11.3600 19.6980 -37.5850

H 10.7960 19.9880 -36.6960

H 10.9260 20.2280 -38.4370

C 12.8190 20.1120 -37.4160

H 13.2360 19.6030 -36.5430

H 13.3910 19.7880 -38.2880

C 12.9790 21.6200 -37.2400

H 12.3810 21.9480 -36.3870

H 12.5950 22.1310 -38.1270

C 14.4360 22.0150 -37.0170

H 14.8090 21.5200 -36.1170

H 15.0390 21.6620 -37.8570

C 14.6090 23.5240 -36.8700

H 13.9890 23.8800 -36.0440

H 14.2590 24.0180 -37.7800

C 16.0620 23.9130 -36.6100

H 16.3980 23.4470 -35.6810

H 16.6900 23.5300 -37.4180

C 16.2360 25.4240 -36.5110

H 15.9290 25.9040 -37.4380

H 17.2750 25.6790 -36.3210

H 15.6290 25.8250 -35.7020

C 6.9460 14.7420 -42.4190

H 6.3500 15.6440 -42.6090

H 6.5280 13.9090 -43.0010

C 8.4010 15.0020 -42.8040

H 8.4890 15.0000 -43.8920

H 9.0120 14.1880 -42.4100

C 8.8780 16.3370 -42.2370

H 8.6250 16.3760 -41.1760

H 8.3410 17.1470 -42.7370

C 10.3790 16.5580 -42.3980

H 10.9220 15.8130 -41.8110

H 10.6620 16.4230 -43.4450

C 10.7760 17.9590 -41.9390

H 10.3050 18.6960 -42.5950

H 10.3940 18.1270 -40.9300

C 12.2860 18.1820 -41.9410

H 12.7550 17.4960 -41.2310

H 12.6890 17.9560 -42.9310

C 12.6360 19.6200 -41.5660

H 12.1640 19.8680 -40.6130

H 12.2270 20.2970 -42.3210

C 14.1410 19.8480 -41.4510

H 14.5440 19.2070 -40.6620

H 14.6260 19.5600 -42.3860

C 14.4690 21.3050 -41.1370

H 14.1160 21.9380 -41.9550

H 13.9350 21.6070 -40.2340

C 15.9650 21.5330 -40.9350

H 16.5060 21.2100 -41.8270

H 16.3140 20.9190 -40.1000

C 16.2870 22.9980 -40.6510

H 15.9730 23.6080 -41.5020

H 15.7170 23.3290 -39.7810

C 17.7730 23.2160 -40.3940

H 18.1040 22.6220 -39.5430

H 17.9770 24.2630 -40.1820

H 18.3590 22.9200 -41.2620

O 6.9370 13.6520 -38.3040

O 4.8340 13.8000 -36.9110

O 6.9360 14.4150 -41.0440

O 0.9580 14.8120 -40.0070

C -0.8850 14.8410 -35.5810

C 0.1940 14.1660 -35.0020

C -1.9090 15.3330 -34.7640

C 0.2520 13.9950 -33.6370

H 0.9710 13.7720 -35.6370

C -1.8530 15.1500 -33.4020

H -2.7410 15.8510 -35.2170

C -0.7710 14.4830 -32.8260

H 1.0830 13.4800 -33.1810

H -2.6360 15.5160 -32.7560

C -2.4930 15.8960 -46.2280

C -1.5560 15.3500 -47.1120

C -3.6020 16.5740 -46.7400

C -1.7350 15.4760 -48.4720

H -0.7000 14.8250 -46.7170

C -3.7780 16.6940 -48.1010

H -4.3120 17.0100 -46.0530

C -2.8480 16.1440 -48.9800

H -1.0250 15.0550 -49.1680

H -4.6340 17.2140 -48.4990

C -5.2470 15.9170 -40.8400

H -5.2680 15.0070 -40.2210

H -5.0880 16.7770 -40.1730

C 2.0480 14.8210 -40.9000

H 1.9250 14.0250 -41.6520

H 2.0870 15.7840 -41.4320

N -4.4260 16.8230 -53.4840

N -5.0940 15.1040 -69.3130

H -4.2530 16.7890 -51.8300

H 0.3030 13.5390 -29.9190

H -3.0260 16.2800 -58.3520

H -5.9090 17.2240 -57.9790

H -3.1340 15.6060 -64.9900

H -6.0470 16.3990 -64.5030

C -5.2030 -6.0410 -46.4860

C -4.7230 -6.2100 -52.1310

C -4.6900 -6.3030 -53.3360

C -4.5540 -6.4400 -54.7280

C -5.6570 -6.2610 -55.5880

C -3.3000 -6.7610 -55.2490

H -2.4930 -6.8760 -54.5440

O -4.2470 -5.9880 -45.7370

O -6.4490 -6.1470 -46.0700

C -4.4450 -6.4710 -65.7160

C -4.1470 -6.8790 -60.0770

C -4.0980 -6.8540 -58.8680

C -4.1990 -6.7470 -57.4690

C -3.0990 -6.9300 -56.6100

C -5.4520 -6.4150 -56.9480

H -6.2510 -6.2730 -57.6570

O -5.4960 -6.2310 -66.2960

O -3.2930 -6.5970 -66.3320

C 0.3780 -8.0360 -57.0770

C 1.5400 -8.2730 -56.3520

H 1.5430 -8.1050 -55.2870

C 2.6810 -8.7120 -57.0040

C 2.6780 -8.9160 -58.3990

C 1.4910 -8.7040 -59.1120

C 0.3520 -8.2650 -58.4370

H -0.5610 -8.0700 -58.9720

C 4.0040 -8.7980 -55.0100

H 3.8030 -7.7510 -54.7420

H 3.2900 -9.4380 -54.4730

C 5.4370 -9.1650 -54.6290

H 6.1000 -8.7380 -55.3820

H 5.6660 -8.7010 -53.6670

C 5.6830 -10.6700 -54.5240

H 5.0410 -11.0870 -53.7440

H 6.7190 -10.8230 -54.2110

C 5.4450 -11.4250 -55.8310

H 4.3840 -11.4000 -56.0810

H 5.9800 -10.9250 -56.6400

C 5.9080 -12.8770 -55.7250

H 5.4300 -13.3450 -54.8610

H 6.9870 -12.9000 -55.5530

C 5.5820 -13.6910 -56.9750

H 4.4980 -13.7200 -57.1130

H 6.0150 -13.2020 -57.8510

C 6.1170 -15.1180 -56.8770

H 5.7310 -15.5850 -55.9670

H 7.2060 -15.0870 -56.7930

C 5.7290 -15.9750 -58.0800

H 4.6400 -16.0450 -58.1360

H 6.0790 -15.4950 -58.9970

C 6.3190 -17.3810 -57.9890

H 6.0030 -17.8440 -57.0510

H 7.4100 -17.3120 -57.9690

C 5.8920 -18.2690 -59.1550

H 6.1850 -17.7980 -60.0960

H 4.8030 -18.3630 -59.1560

C 6.5150 -19.6600 -59.0730

H 7.6030 -19.5680 -59.0900

H 6.2380 -20.1230 -58.1230

C 6.0650 -20.5560 -60.2210

H 6.3380 -20.1150 -61.1780

H 6.5320 -21.5360 -60.1500

H 4.9860 -20.6890 -60.2000

C 4.0520 -10.0560 -60.0460

H 3.5370 -9.6430 -60.9170

H 5.1310 -10.0430 -60.2300

C 3.5990 -11.4940 -59.7990

H 3.9970 -11.8300 -58.8390

H 2.5080 -11.5290 -59.7410

C 4.1010 -12.4010 -60.9180

H 5.1940 -12.3970 -60.9120

H 3.7790 -11.9980 -61.8810

C 3.6090 -13.8400 -60.7870

H 3.8720 -14.2250 -59.8000

H 2.5200 -13.8640 -60.8720

C 4.2290 -14.7300 -61.8610

H 5.3140 -14.7410 -61.7300

H 4.0230 -14.3050 -62.8460

C 3.7080 -16.1630 -61.8140

H 3.8700 -16.5760 -60.8170

H 2.6310 -16.1660 -62.0010

C 4.4070 -17.0450 -62.8460

H 5.4780 -17.0670 -62.6310

H 4.2830 -16.6070 -63.8390

C 3.8680 -18.4730 -62.8550

H 3.9570 -18.8970 -61.8530

H 2.8060 -18.4590 -63.1130

C 4.6200 -19.3560 -63.8470

H 5.6780 -19.3870 -63.5720

H 4.5560 -18.9160 -64.8450

C 4.0700 -20.7790 -63.8870

H 4.1160 -21.2130 -62.8850

H 3.0190 -20.7510 -64.1850

C 4.8460 -21.6680 -64.8550

H 5.8880 -21.7260 -64.5330

H 4.8330 -21.2180 -65.8500

C 4.2600 -23.0720 -64.9300

H 3.2280 -23.0360 -65.2750

H 4.8300 -23.6900 -65.6200

H 4.2760 -23.5460 -63.9510

C 0.3140 -8.8300 -61.1960

H -0.4800 -9.4380 -60.7410

H 0.0010 -7.7780 -61.2140

C 0.6110 -9.3270 -62.6080

H -0.1940 -9.0100 -63.2730

H 1.5400 -8.8610 -62.9410

C 0.7370 -10.8480 -62.6420

H 1.4200 -11.1610 -61.8510

H -0.2410 -11.2890 -62.4300

C 1.2470 -11.3800 -63.9790

H 2.2770 -11.0520 -64.1340

H 0.6450 -10.9720 -64.7940

C 1.1830 -12.9060 -64.0140

H 0.1360 -13.2190 -63.9910

H 1.6630 -13.3040 -63.1170

C 1.8580 -13.5070 -65.2430

H 2.9250 -13.2720 -65.2230

H 1.4400 -13.0610 -66.1480

C 1.6700 -15.0220 -65.2880

H 1.9900 -15.4510 -64.3360

H 0.6080 -15.2470 -65.4090

C 2.4550 -15.6830 -66.4180

H 3.5230 -15.5110 -66.2650

H 2.1820 -15.2240 -67.3710

C 2.1870 -17.1850 -66.4830

H 1.1310 -17.3520 -66.7090

H 2.3870 -17.6280 -65.5050

C 3.0440 -17.8860 -67.5330

H 2.8720 -17.4260 -68.5100

H 4.1000 -17.7480 -67.2870

C 2.7370 -19.3790 -67.6170

H 1.6940 -19.5170 -67.9100

H 2.8630 -19.8270 -66.6290

C 3.6420 -20.0930 -68.6140

H 4.6870 -19.9780 -68.3320

H 3.4100 -21.1550 -68.6510

H 3.5120 -19.6780 -69.6120

O 3.9060 -9.2200 -58.9150

O 3.8720 -8.9670 -56.4040

O 1.5100 -8.9480 -60.4530

O -1.9070 -7.2550 -57.1850

C -9.2320 -5.5830 -55.0960

C -9.2530 -5.3700 -53.7280

H -8.3260 -5.3740 -53.1800

C -10.4700 -5.1500 -53.0980

C -11.6840 -5.1580 -53.8200

C -11.6480 -5.4100 -55.1980

C -10.4150 -5.6010 -55.8130

H -10.3620 -5.7500 -56.8790

C -9.5340 -5.0660 -50.8930

H -8.7040 -4.4020 -51.1700

H -9.1880 -6.1090 -50.9380

C -9.9740 -4.7160 -49.4750

H -10.3370 -3.6880 -49.4730

H -9.0770 -4.7530 -48.8570

C -11.0520 -5.6440 -48.9110

H -10.8550 -6.6690 -49.2340

H -10.9980 -5.6290 -47.8190

C -12.4590 -5.2320 -49.3420

H -12.4950 -5.1720 -50.4300

H -12.6730 -4.2370 -48.9450

C -13.5250 -6.2070 -48.8470

H -13.3590 -7.1870 -49.3030

H -13.4340 -6.3330 -47.7640

C -14.9350 -5.7250 -49.1820

H -14.9950 -5.5190 -50.2520

H -15.1300 -4.7890 -48.6530

C -16.0010 -6.7510 -48.8060

H -15.7850 -7.6950 -49.3120

H -15.9610 -6.9400 -47.7310

C -17.4070 -6.2930 -49.1870

H -17.4370 -6.0810 -50.2580

H -17.6420 -5.3640 -48.6600

C -18.4590 -7.3470 -48.8540

H -18.1980 -8.2830 -49.3530

H -18.4530 -7.5360 -47.7770

C -19.8630 -6.9290 -49.2840

H -20.1300 -5.9890 -48.7950

H -19.8720 -6.7520 -50.3620

C -20.9060 -7.9910 -48.9410

H -20.9380 -8.1270 -47.8570

H -20.6080 -8.9440 -49.3830

C -22.2930 -7.6120 -49.4450

H -22.6160 -6.6710 -49.0050

H -23.0190 -8.3790 -49.1840

H -22.2890 -7.4980 -50.5270

C -14.0820 -5.1790 -53.3850

H -14.2890 -4.9710 -54.4350

H -14.6890 -4.5140 -52.7610

C -14.4380 -6.6300 -53.0560

H -14.1130 -6.8520 -52.0380

H -13.9030 -7.3030 -53.7290

C -15.9460 -6.8360 -53.1780

H -16.4530 -6.0880 -52.5620

H -16.2540 -6.6680 -54.2120

C -16.3970 -8.2240 -52.7330

H -16.0800 -8.3930 -51.7020

H -15.9190 -8.9850 -53.3540

C -17.9150 -8.3650 -52.8230

H -18.3790 -7.5840 -52.2170

H -18.2340 -8.2120 -53.8560

C -18.4090 -9.7250 -52.3390

H -18.0840 -9.8810 -51.3070

H -17.9590 -10.5160 -52.9450

C -19.9300 -9.8280 -52.4110

H -20.3710 -9.0180 -51.8260

H -20.2530 -9.6990 -53.4460

C -20.4460 -11.1640 -51.8850

H -20.1080 -11.3040 -50.8550

H -20.0220 -11.9770 -52.4800

C -21.9700 -11.2450 -51.9300

H -22.3920 -10.4490 -51.3110

H -22.3080 -11.0780 -52.9550

C -22.4890 -12.5940 -51.4420

H -22.1590 -12.7590 -50.4130

H -22.0580 -13.3900 -52.0550

C -24.0120 -12.6810 -51.5040

H -24.4430 -11.9090 -50.8620

H -24.3430 -12.4850 -52.5250

C -24.5210 -14.0490 -51.0650

H -24.1210 -14.8280 -51.7110

H -25.6070 -14.0880 -51.1130

H -24.2140 -14.2620 -50.0430

C -12.7930 -5.9220 -57.2420

H -12.2070 -6.8470 -57.3260

H -12.3270 -5.1490 -57.8700

C -14.2240 -6.1820 -57.7050

H -14.2170 -6.3020 -58.7890

H -14.8370 -5.3120 -57.4660

C -14.8240 -7.4240 -57.0510

H -14.6880 -7.3550 -55.9710

H -14.2880 -8.3110 -57.3990

C -16.3110 -7.5740 -57.3650

H -16.8550 -6.7230 -56.9490

H -16.4580 -7.5600 -58.4480

C -16.8780 -8.8710 -56.7940

H -16.3900 -9.7210 -57.2760

H -16.6510 -8.9230 -55.7280

C -18.3890 -8.9830 -56.9880

H -18.8810 -8.1500 -56.4790

H -18.6270 -8.9040 -58.0520

C -18.9320 -10.3010 -56.4430

H -18.6460 -10.4010 -55.3940

H -18.4750 -11.1310 -56.9880

C -20.4510 -10.3980 -56.5610

H -20.9090 -9.5710 -56.0120

H -20.7410 -10.2950 -57.6090

C -20.9810 -11.7220 -56.0150

H -20.5410 -12.5470 -56.5800

H -20.6690 -11.8330 -54.9750

C -22.5030 -11.8120 -56.0950

H -22.8180 -11.7060 -57.1360

H -22.9430 -10.9830 -55.5340

C -23.0300 -13.1320 -55.5400

H -22.5960 -13.9600 -56.1060

H -22.7080 -13.2390 -54.5020

C -24.5500 -13.2130 -55.6100

H -25.0020 -12.3940 -55.0520

H -24.9040 -14.1510 -55.1890

H -24.8890 -13.1480 -56.6410

O -12.7570 -4.8410 -53.0410

O -10.6280 -4.9010 -51.7740

O -12.8280 -5.4860 -55.8970

O -6.8420 -5.9390 -55.0130

C -4.8300 -6.1210 -50.7300

C -6.0910 -6.2080 -50.1360

C -3.6950 -5.9710 -49.9260

C -6.2160 -6.1660 -48.7660

H -6.9580 -6.3210 -50.7660

C -3.8260 -5.9250 -48.5550

H -2.7220 -5.9010 -50.3900

C -5.0830 -6.0330 -47.9650

H -7.1850 -6.2440 -48.3000

H -2.9640 -5.8160 -47.9150

C -4.2170 -6.8380 -61.4800

C -5.4210 -6.4730 -62.0970

C -3.1010 -7.1310 -62.2700

C -5.4950 -6.3730 -63.4670

H -6.2820 -6.2580 -61.4810

C -3.1820 -7.0330 -63.6400

H -2.1800 -7.4270 -61.7920

C -4.3710 -6.6370 -64.2510

H -6.4120 -6.0830 -63.9580

H -2.3330 -7.2600 -64.2650

C -0.8280 -7.5410 -56.3220

H -0.5490 -6.6350 -55.7610

H -1.1290 -8.3110 -55.5950

C -7.9570 -5.8040 -55.8680

H -7.8050 -4.9570 -56.5560

H -8.0710 -6.7150 -56.4740

C -4.2540 -6.3940 -31.9720

C -5.3030 -6.6870 -32.7560

C -5.3160 -6.7740 -34.1990

C -6.5660 -6.7940 -34.8600

C -4.1530 -6.8460 -34.9680

H -3.2070 -6.8590 -34.4520

C -6.5290 -6.6160 -39.2270

C -5.4640 -6.8820 -38.4550

C -5.4390 -6.8810 -37.0110

C -4.1910 -6.9120 -36.3500

C -6.6010 -6.8370 -36.2420

H -7.5460 -6.8500 -36.7560

C -0.7790 -7.4990 -37.5970

C 0.4280 -8.0410 -37.1710

H 0.5420 -8.3270 -36.1380

C 1.4700 -8.2000 -38.0710

C 1.3080 -7.8090 -39.4130

C 0.0840 -7.2680 -39.8380

C -0.9540 -7.1300 -38.9180

H -1.8970 -6.7010 -39.2090

C 3.0270 -8.9400 -36.4140

H 2.8420 -8.0310 -35.8260

H 2.4180 -9.7560 -35.9990

C 4.5110 -9.3000 -36.3550

H 5.0360 -8.6410 -37.0450

H 4.8740 -9.1000 -35.3450

C 4.8090 -10.7560 -36.7120

H 4.3390 -11.4140 -35.9760

H 5.8890 -10.9100 -36.6480

C 4.3310 -11.1390 -38.1110

H 3.2410 -11.1210 -38.1370

H 4.6850 -10.3970 -38.8280

C 4.8170 -12.5270 -38.5250

H 4.5590 -13.2500 -37.7470

H 5.9060 -12.5200 -38.6200

C 4.1920 -12.9670 -39.8460

H 3.1070 -13.0170 -39.7220

H 4.4030 -12.2180 -40.6130

C 4.7010 -14.3270 -40.3180

H 4.5520 -15.0650 -39.5270

H 5.7740 -14.2660 -40.5150

C 3.9760 -14.7880 -41.5810

H 2.9080 -14.8720 -41.3650

H 4.0950 -14.0330 -42.3610

C 4.4850 -16.1320 -42.0950

H 4.3960 -16.8790 -41.3030

H 5.5440 -16.0460 -42.3500

C 3.7030 -16.6000 -43.3200

H 3.7870 -15.8500 -44.1100

H 2.6460 -16.6850 -43.0590

C 4.1960 -17.9450 -43.8470

H 5.2470 -17.8580 -44.1340

H 4.1330 -18.6900 -43.0500

C 3.3790 -18.4160 -45.0440

H 3.4370 -17.6920 -45.8540

H 3.7500 -19.3710 -45.4110

H 2.3350 -18.5360 -44.7680

C 2.2710 -8.6410 -41.4330

H 1.6430 -8.0850 -42.1380

H 3.2880 -8.6940 -41.8310

C 1.7060 -10.0440 -41.2150

H 2.3470 -10.5730 -40.5070

H 0.7160 -9.9550 -40.7610

C 1.5970 -10.8360 -42.5130

H 2.5930 -10.9850 -42.9350

H 1.0120 -10.2660 -43.2380

C 0.9350 -12.1920 -42.2780

H 1.5100 -12.7420 -41.5310

H -0.0680 -12.0350 -41.8750

C 0.8380 -13.0320 -43.5480

H 1.8390 -13.1830 -43.9590

H 0.2510 -12.4950 -44.2970

C 0.1970 -14.3890 -43.2730

H 0.7940 -14.9200 -42.5280

H -0.7990 -14.2400 -42.8500

C 0.0870 -15.2500 -44.5280

H 1.0730 -15.3480 -44.9880

H -0.5660 -14.7570 -45.2520

C -0.4590 -16.6410 -44.2150

H 0.2220 -17.1450 -43.5240

H -1.4240 -16.5480 -43.7120

C -0.6230 -17.4960 -45.4690

H 0.3240 -17.5250 -46.0140

H -1.3640 -17.0350 -46.1270

C -1.0550 -18.9210 -45.1350

H -0.2990 -19.3870 -44.4980

H -1.9890 -18.8940 -44.5680

C -1.2500 -19.7740 -46.3860

H -0.3230 -19.7800 -46.9650

H -2.0250 -19.3250 -47.0120

C -1.6400 -21.2060 -46.0410

H -2.5630 -21.2240 -45.4660

H -1.7880 -21.7920 -46.9460

H -0.8600 -21.6800 -45.4490

C -1.1330 -6.4990 -41.8140

H -1.9360 -6.2230 -41.1290

H -0.8650 -5.6100 -42.3930

C -1.5810 -7.6020 -42.7750

H -2.3150 -7.1810 -43.4650

H -0.7130 -7.9110 -43.3610

C -2.1760 -8.8220 -42.0780

H -1.4670 -9.2080 -41.3410

H -3.0880 -8.5390 -41.5480

C -2.4960 -9.9130 -43.0960

H -1.5780 -10.1880 -43.6210

H -3.1920 -9.5130 -43.8380

C -3.0970 -11.1670 -42.4670

H -4.0450 -10.9170 -41.9840

H -2.4240 -11.5460 -41.6940

C -3.3250 -12.2510 -43.5170

H -2.3790 -12.4580 -44.0230

H -4.0240 -11.8790 -44.2690

C -3.8650 -13.5500 -42.9260

H -3.1680 -13.9230 -42.1700

H -4.8170 -13.3580 -42.4270

C -4.0570 -14.6140 -44.0050

H -3.1140 -14.7590 -44.5360

H -4.7920 -14.2590 -44.7310

C -4.5190 -15.9520 -43.4340

H -5.4560 -15.8120 -42.8890

H -3.7770 -16.3170 -42.7190

C -4.7210 -16.9950 -44.5310

H -5.4940 -16.6470 -45.2200

H -3.7950 -17.1000 -45.1020

C -5.1210 -18.3570 -43.9710

H -6.0350 -18.2520 -43.3830

H -4.3370 -18.7160 -43.2990

C -5.3470 -19.3780 -45.0800

H -4.4470 -19.4940 -45.6800

H -5.6090 -20.3480 -44.6630

H -6.1540 -19.0570 -45.7350

O 2.4140 -7.9240 -40.2180

O 2.6890 -8.7060 -37.7660

O 0.0540 -6.8550 -41.1320

O -3.0870 -6.9830 -37.1500

C -10.0280 -6.6440 -33.6590

C -9.7520 -6.5370 -32.3070

H -8.7340 -6.6060 -31.9680

C -10.7840 -6.2970 -31.4150

C -12.1190 -6.1760 -31.8590

C -12.3950 -6.3540 -33.2230

C -11.3370 -6.5750 -34.1010

H -11.5260 -6.6830 -35.1570

C -9.4170 -6.5700 -29.4760

H -8.5820 -5.9230 -29.7770

H -9.1960 -7.6010 -29.7910

C -9.5740 -6.5070 -27.9580

H -9.8060 -5.4810 -27.6750

H -8.6060 -6.7630 -27.5240

C -10.6510 -7.4510 -27.4180

H -10.6350 -8.3820 -27.9900

H -10.4160 -7.7010 -26.3800

C -12.0540 -6.8470 -27.4740

H -12.2510 -6.4840 -28.4820

H -12.0980 -5.9920 -26.7940

C -13.1230 -7.8620 -27.0780

H -13.1380 -8.6710 -27.8140

H -12.8710 -8.3060 -26.1110

C -14.5120 -7.2330 -26.9950

H -14.7110 -6.6820 -27.9160

H -14.5350 -6.5140 -26.1720

C -15.6030 -8.2800 -26.7840

H -15.5750 -8.9970 -27.6090

H -15.4010 -8.8330 -25.8640

C -16.9970 -7.6620 -26.7050

H -17.1860 -7.0830 -27.6120

H -17.0400 -6.9730 -25.8590

C -18.0840 -8.7230 -26.5510

H -18.0410 -9.4070 -27.4020

H -17.8910 -9.3100 -25.6490

C -19.4820 -8.1150 -26.4660

H -19.5360 -7.4520 -25.5990

H -19.6660 -7.5090 -27.3560

C -20.5670 -9.1840 -26.3540

H -20.3820 -9.7950 -25.4680

H -20.5140 -9.8410 -27.2250

C -21.9610 -8.5750 -26.2660

H -22.0470 -7.9440 -25.3840

H -22.7170 -9.3550 -26.2050

H -22.1680 -7.9630 -27.1420

C -14.3710 -5.7640 -31.0080

H -14.6380 -5.2540 -31.9350

H -14.6910 -5.1450 -30.1630

C -15.0620 -7.1250 -30.9300

H -14.6960 -7.6550 -30.0490

H -14.7980 -7.7130 -31.8100

C -16.5740 -6.9450 -30.8410

H -16.8060 -6.3120 -29.9810

H -16.9280 -6.4250 -31.7330

C -17.3190 -8.2690 -30.6960

H -16.9680 -8.7860 -29.8010

H -17.1000 -8.9100 -31.5540

C -18.8250 -8.0440 -30.5940

H -19.0310 -7.4070 -29.7310

H -19.1700 -7.5110 -31.4830

C -19.6100 -9.3440 -30.4520

H -19.2640 -9.8820 -29.5670

H -19.4190 -9.9830 -31.3190

C -21.1080 -9.0800 -30.3330

H -21.2910 -8.4480 -29.4610

H -21.4470 -8.5300 -31.2140

C -21.9220 -10.3640 -30.1990

H -21.5800 -10.9230 -29.3260

H -21.7530 -10.9930 -31.0770

C -23.4140 -10.0740 -30.0610

H -23.5810 -9.4720 -29.1650

H -23.7460 -9.4860 -30.9200

C -24.2470 -11.3490 -29.9740

H -23.9180 -11.9410 -29.1160

H -24.0790 -11.9520 -30.8710

C -25.7380 -11.0490 -29.8400

H -25.9090 -10.4830 -28.9210

H -26.0550 -10.4240 -30.6770

C -26.5760 -12.3210 -29.8150

H -26.4400 -12.8860 -30.7360

H -27.6320 -12.0820 -29.7140

H -26.2850 -12.9550 -28.9800

C -13.9780 -6.5110 -35.0200

H -13.6900 -7.5320 -35.3090

H -13.4130 -5.7980 -35.6350

C -15.4690 -6.2910 -35.2570

H -15.6170 -6.1630 -36.3310

H -15.7610 -5.3610 -34.7680

C -16.3330 -7.4460 -34.7600

H -16.1000 -7.6480 -33.7140

H -16.0960 -8.3460 -35.3340

C -17.8210 -7.1330 -34.8980

H -18.0790 -6.3120 -34.2250

H -18.0270 -6.7990 -35.9180

C -18.6990 -8.3410 -34.5810

H -18.4980 -9.1330 -35.3060

H -18.4420 -8.7260 -33.5930

C -20.1860 -7.9960 -34.6120

H -20.3990 -7.2440 -33.8470

H -20.4380 -7.5560 -35.5800

C -21.0610 -9.2230 -34.3710

H -20.7730 -9.6900 -33.4270

H -20.8850 -9.9520 -35.1660

C -22.5470 -8.8780 -34.3250

H -22.7300 -8.1740 -33.5090

H -22.8340 -8.3820 -35.2560

C -23.4150 -10.1180 -34.1250

H -23.2510 -10.8090 -34.9560

H -23.1080 -10.6270 -33.2090

C -24.9000 -9.7780 -34.0380

H -25.2060 -9.2500 -34.9450

H -25.0670 -9.1040 -33.1940

C -25.7640 -11.0250 -33.8640

H -25.6110 -11.6900 -34.7180

H -25.4450 -11.5600 -32.9680

C -27.2440 -10.6820 -33.7480

H -27.4200 -10.0320 -32.8930

H -27.8380 -11.5840 -33.6200

H -27.5880 -10.1670 -34.6430

O -12.9720 -5.8490 -30.8480

O -10.6200 -6.1370 -30.0770

O -13.7000 -6.3000 -33.6500

O -7.6710 -6.7630 -34.0730

C -4.2950 -6.3190 -30.5240

C -3.1320 -6.0040 -29.8140

C -5.4540 -6.5270 -29.7690

C -3.1860 -5.8970 -28.4370

H -2.2020 -5.8300 -30.3370

C -5.3890 -6.4080 -28.3940

H -6.3870 -6.7880 -30.2410

H -2.3070 -5.6370 -27.8630

H -6.2690 -6.5630 -27.7820

C -6.5120 -6.5360 -40.6710

C -7.7210 -6.5550 -41.3770

C -5.3400 -6.3910 -41.4200

C -7.7010 -6.4510 -42.7510

H -8.6560 -6.6540 -40.8480

C -5.4280 -6.2870 -42.7920

H -4.3790 -6.3290 -40.9380

H -8.6120 -6.4670 -43.3340

H -4.5530 -6.1650 -43.4160

C -1.8420 -7.2600 -36.5520

H -1.5260 -6.4090 -35.9260

H -1.9240 -8.1460 -35.9060

C -8.9390 -6.8050 -34.6880

H -9.0300 -5.9870 -35.4180

H -9.0670 -7.7620 -35.2180

N -4.2860 -6.0990 -27.7320

N -6.5810 -6.3230 -43.4450

H -6.5040 -6.1840 -45.0560

H -3.3660 -6.4320 -67.3240

H -3.2990 -6.1350 -32.4110

H -6.2720 -6.8340 -32.3050

H -4.5120 -7.0990 -38.9180

H -7.4930 -6.4230 -38.7770

C -6.8590 -0.3500 -32.9560

C -5.8470 -0.4460 -33.8340

C -5.9320 -0.4250 -35.2750

C -4.7370 -0.5310 -36.0200

C -7.1340 -0.2900 -35.9770

H -8.0430 -0.2100 -35.4070

C -4.9000 -0.2520 -40.3190

C -5.9950 -0.2300 -39.5420

C -5.9880 -0.3120 -38.1000

C -7.1880 -0.2510 -37.3600

C -4.7940 -0.4510 -37.3970

H -3.8950 -0.4850 -37.9810

O -8.3240 -0.1430 -38.0930

C -1.1430 -1.1610 -35.4340

C -1.0880 -1.7040 -34.1660

H -2.0010 -1.8400 -33.6120

C 0.1450 -2.0740 -33.6380

C 1.3170 -1.9880 -34.4140

C 1.2470 -1.4190 -35.7010

C 0.0190 -0.9660 -36.1640

H -0.0570 -0.5020 -37.1330

C -0.7270 -2.3540 -31.4440

H -1.5500 -3.0650 -31.6140

H -1.1240 -1.3370 -31.5560

C -0.1810 -2.5300 -30.0290

H 0.2100 -3.5430 -29.9200

H -1.0250 -2.4150 -29.3460

C 0.9210 -1.5220 -29.6840

H 0.7170 -0.5740 -30.1880

H 0.9060 -1.3300 -28.6080

C 2.3100 -2.0190 -30.0810

H 2.2940 -2.3270 -31.1260

H 2.5530 -2.8980 -29.4780

C 3.3890 -0.9580 -29.8730

H 3.2280 -0.1310 -30.5700

H 3.3140 -0.5510 -28.8620

C 4.7860 -1.5420 -30.0780

H 4.8130 -2.0780 -31.0290

H 4.9850 -2.2710 -29.2880

C 5.8850 -0.4830 -30.0700

H 5.7290 0.2100 -30.9010

H 5.8280 0.0970 -29.1470

C 7.2700 -1.1160 -30.1850

H 7.3150 -1.7280 -31.0890

H 7.4280 -1.7830 -29.3340

C 8.3920 -0.0810 -30.2220

H 8.2500 0.5790 -31.0810

H 8.3440 0.5350 -29.3210

C 9.7670 -0.7410 -30.3080

H 9.9010 -1.4030 -29.4500

H 9.8170 -1.3600 -31.2080

C 10.9000 0.2810 -30.3350

H 10.8430 0.9030 -29.4400

H 10.7750 0.9320 -31.2040

C 12.2680 -0.3860 -30.3960

H 12.4350 -0.9980 -29.5110

H 13.0580 0.3600 -30.4510

H 12.3430 -1.0300 -31.2710

C 3.6830 -1.9230 -33.9340

H 3.7650 -1.2140 -33.0980

H 3.7840 -1.3670 -34.8680

C 4.7730 -2.9870 -33.8440

H 4.6000 -3.7250 -34.6280

H 4.6910 -3.5030 -32.8850

C 6.1630 -2.3770 -34.0010

H 6.2170 -1.8530 -34.9570

H 6.3250 -1.6380 -33.2140

C 7.2710 -3.4260 -33.9480

H 7.0920 -4.1820 -34.7170

H 7.2500 -3.9330 -32.9800

C 8.6460 -2.7980 -34.1640

H 8.6490 -2.2630 -35.1170

H 8.8340 -2.0640 -33.3780

C 9.7720 -3.8280 -34.1720

H 9.5820 -4.5670 -34.9540

H 9.7880 -4.3590 -33.2170

C 11.1320 -3.1750 -34.4120

H 11.1020 -2.6170 -35.3510

H 11.3340 -2.4580 -33.6120

C 12.2670 -4.1940 -34.4730

H 12.0630 -4.9130 -35.2710

H 12.3060 -4.7520 -33.5350

C 13.6180 -3.5290 -34.7230

H 13.5700 -2.9520 -35.6510

H 13.8320 -2.8290 -33.9120

C 14.7520 -4.5450 -34.8250

H 14.5360 -5.2470 -35.6350

H 14.8050 -5.1240 -33.9000

C 16.1020 -3.8790 -35.0810

H 16.0420 -3.2750 -35.9890

H 16.3360 -3.2070 -34.2520

C 17.2200 -4.9030 -35.2340

H 17.3090 -5.5090 -34.3340

H 18.1720 -4.4080 -35.4100

H 17.0180 -5.5670 -36.0720

C 2.3470 -0.8290 -37.7670

H 2.3970 0.2680 -37.7140

H 1.4240 -1.1150 -38.2770

C 3.5050 -1.3940 -38.5840

H 3.5220 -0.8570 -39.5350

H 3.2840 -2.4400 -38.7940

C 4.8620 -1.2880 -37.8990

H 4.8330 -1.8520 -36.9650

H 5.0740 -0.2450 -37.6560

C 5.9720 -1.8420 -38.7870

H 5.7110 -2.8570 -39.0900

H 6.0470 -1.2330 -39.6910

C 7.3240 -1.8680 -38.0810

H 7.5770 -0.8620 -37.7390

H 7.2580 -2.5120 -37.2010

C 8.4310 -2.3790 -38.9960

H 8.1760 -3.3830 -39.3410

H 8.4970 -1.7330 -39.8750

C 9.7870 -2.4190 -38.2990

H 9.7300 -3.0880 -37.4370

H 10.0330 -1.4210 -37.9290

C 10.8950 -2.8930 -39.2340

H 10.6500 -3.8930 -39.6000

H 10.9440 -2.2270 -40.0990

C 12.2560 -2.9280 -38.5470

H 12.4970 -1.9300 -38.1710

H 12.2080 -3.6030 -37.6890

C 13.3630 -3.3900 -39.4900

H 13.4090 -2.7170 -40.3500

H 13.1230 -4.3880 -39.8630

C 14.7250 -3.4250 -38.8030

H 14.9700 -2.4260 -38.4360

H 14.6720 -4.0930 -37.9410

C 15.8260 -3.9020 -39.7410

H 15.6180 -4.9100 -40.0910

H 16.7880 -3.9080 -39.2320

H 15.9010 -3.2460 -40.6070

O 2.4230 -2.5750 -33.8650

O 0.3070 -2.5730 -32.3850

O 2.3860 -1.3600 -36.4490

O -3.5570 -0.7100 -35.3450

C -6.7270 -0.3680 -31.5140

C -7.8780 -0.3070 -30.7190

C -5.5060 -0.4410 -30.8370

C -7.7560 -0.3310 -29.3430

H -8.8530 -0.2500 -31.1800

C -5.4990 -0.4510 -29.4540

H -4.5740 -0.4910 -31.3760

H -8.6280 -0.2930 -28.7040

H -4.5700 -0.5020 -28.9030

C -4.8450 -0.1320 -41.7550

C -3.5850 -0.0910 -42.3660

C -5.9620 -0.0350 -42.5900

C -3.5030 0.0370 -43.7360

H -2.6880 -0.1550 -41.7680

C -5.7730 0.0810 -43.9520

H -6.9600 -0.0580 -42.1860

H -2.5510 0.0810 -44.2470

H -6.5990 0.1550 -44.6450

C -9.5770 -0.1770 -37.4500

H -9.6390 -1.0630 -36.8000

H -9.7130 0.7220 -36.8330

C -2.4250 -0.8850 -36.1650

H -2.5900 -1.7460 -36.8360

H -2.2800 0.0000 -36.7980

C -5.6550 0.3520 -47.6080

C -5.9600 0.5820 -53.2540

C -6.0980 0.5810 -54.4550

C -6.4030 0.5640 -55.8270

C -5.4410 0.2300 -56.7990

C -7.7130 0.8620 -56.2050

H -8.3890 1.1480 -55.4170

O -6.6490 0.3620 -46.9130

O -4.4320 0.2460 -47.1300

C -5.1140 -0.1740 -66.3300

C -7.1750 0.3760 -61.0870

C -7.3510 0.4630 -59.8910

C -7.1670 0.5000 -58.4970

C -8.1370 0.8040 -57.5260

C -5.8450 0.2240 -58.1180

H -5.1550 0.0050 -58.9150

O -3.9040 -0.2850 -66.4840

O -5.9780 -0.1970 -67.3110

H -5.5430 -0.3670 -68.2020

O -9.4140 1.0340 -57.9340

C -1.8980 -0.7410 -56.7750

C -1.6850 -0.7890 -55.4090

H -2.4880 -0.4890 -54.7580

C -0.4590 -1.2300 -54.9180

C 0.5580 -1.6250 -55.8050

C 0.3440 -1.5480 -57.1930

C -0.8870 -1.0930 -57.6570

H -1.0810 -1.0270 -58.7160

C -1.0240 -0.7160 -52.6680

H -2.0350 -1.1310 -52.7700

H -1.0720 0.3630 -52.8670

C -0.5420 -0.9560 -51.2400

H -0.5610 -2.0270 -51.0370

H -1.2700 -0.4760 -50.5830

C 0.8640 -0.4150 -50.9570

H 1.0780 0.4200 -51.6260

H 0.9040 -0.0350 -49.9330

C 1.9370 -1.4890 -51.1240

H 1.8840 -1.8910 -52.1370

H 1.7280 -2.3040 -50.4270

C 3.3390 -0.9510 -50.8490

H 3.6250 -0.2650 -51.6500

H 3.3360 -0.3810 -49.9170

C 4.3710 -2.0720 -50.7460

H 4.2770 -2.7290 -51.6150

H 4.1620 -2.6750 -49.8590

C 5.8000 -1.5410 -50.6750

H 5.9970 -0.9310 -51.5600

H 5.9080 -0.8940 -49.8010

C 6.8290 -2.6680 -50.6060

H 6.6420 -3.3730 -51.4210

H 6.7100 -3.2140 -49.6680

C 8.2600 -2.1480 -50.7180

H 8.3640 -1.5860 -51.6490

H 8.4600 -1.4570 -49.8960

C 9.2850 -3.2790 -50.6990

H 9.2210 -3.8130 -49.7490

H 9.0480 -3.9930 -51.4930

C 10.7100 -2.7690 -50.8980

H 10.9520 -2.0550 -50.1070

H 10.7710 -2.2350 -51.8490

C 11.7290 -3.9020 -50.8880

H 11.7030 -4.4310 -49.9380

H 12.7350 -3.5140 -51.0360

H 11.5140 -4.6150 -51.6810

C 2.9030 -1.3830 -55.5260

H 2.8050 -0.3600 -55.1350

H 3.0690 -1.3270 -56.6080

C 4.0620 -2.1150 -54.8580

H 3.9370 -3.1780 -55.0680

H 3.9990 -1.9780 -53.7780

C 5.4200 -1.6480 -55.3720

H 5.4340 -1.7370 -56.4610

H 5.5690 -0.5950 -55.1250

C 6.5620 -2.4830 -54.7950

H 6.3460 -3.5410 -54.9590

H 6.6250 -2.3220 -53.7170

C 7.9060 -2.1520 -55.4400

H 7.8200 -2.2680 -56.5230

H 8.1620 -1.1080 -55.2430

C 9.0250 -3.0580 -54.9310

H 8.7450 -4.1010 -55.0960

H 9.1450 -2.9170 -53.8540

C 10.3550 -2.7830 -55.6290

H 10.2320 -2.9230 -56.7050

H 10.6420 -1.7420 -55.4670

C 11.4690 -3.6980 -55.1270

H 11.1820 -4.7400 -55.2880

H 11.5970 -3.5550 -54.0510

C 12.7980 -3.4280 -55.8290

H 12.6770 -3.5850 -56.9040

H 13.0780 -2.3830 -55.6820

C 13.9190 -4.3240 -55.3100

H 13.6480 -5.3710 -55.4640

H 14.0350 -4.1680 -54.2350

C 15.2480 -4.0400 -56.0050

H 15.1370 -4.2030 -57.0790

H 15.5130 -2.9910 -55.8570

C 16.3710 -4.9230 -55.4750

H 16.5180 -4.7540 -54.4100

H 17.3050 -4.7050 -55.9880

H 16.1340 -5.9730 -55.6250

C 1.2930 -1.5730 -59.3830

H 1.2580 -0.4790 -59.4480

H 0.3870 -1.9830 -59.8450

C 2.4870 -2.1240 -60.1570

H 2.4200 -1.7280 -61.1710

H 2.3830 -3.2070 -60.2120

C 3.8600 -1.7940 -59.5750

H 3.8590 -2.0090 -58.5050

H 4.0830 -0.7350 -59.7120

C 4.9240 -2.6520 -60.2570

H 4.6860 -3.7020 -60.0740

H 4.8740 -2.4910 -61.3360

C 6.3430 -2.3750 -59.7660

H 6.6490 -1.3700 -60.0620

H 6.3650 -2.4140 -58.6730

C 7.3310 -3.4000 -60.3190

H 7.0270 -4.3980 -59.9910

H 7.2900 -3.3870 -61.4110

C 8.7650 -3.1450 -59.8640

H 8.8010 -3.1370 -58.7710

H 9.0870 -2.1600 -60.2110

C 9.7290 -4.2070 -60.3890

H 9.4200 -5.1870 -60.0190

H 9.6750 -4.2320 -61.4800

C 11.1700 -3.9390 -59.9630

H 11.4770 -2.9560 -60.3280

H 11.2250 -3.9160 -58.8710

C 12.1340 -4.9970 -60.4930

H 12.0600 -5.0410 -61.5820

H 11.8460 -5.9770 -60.1030

C 13.5810 -4.7060 -60.1010

H 13.8630 -3.7180 -60.4720

H 13.6620 -4.6850 -59.0110

C 14.5410 -5.7490 -60.6570

H 14.3020 -6.7340 -60.2640

H 15.5680 -5.5110 -60.3860

H 14.4740 -5.7900 -61.7430

O 1.7170 -2.1190 -55.2540

O -0.1500 -1.3250 -53.5970

O 1.3560 -1.9490 -58.0120

O -4.1790 -0.0430 -56.3700

C -5.8500 0.5610 -51.8520

C -4.6680 0.1520 -51.2320

C -6.9510 0.9310 -51.0730

C -4.5930 0.1010 -49.8580

H -3.8190 -0.1190 -51.8430

C -6.8770 0.8590 -49.7000

H -7.8550 1.2670 -51.5590

C -5.7010 0.4400 -49.0870

H -3.6880 -0.2170 -49.3640

H -7.7160 1.1260 -49.0780

C -6.7510 0.2490 -62.4230

C -5.3710 0.0770 -62.6120

C -7.5990 0.2770 -63.5310

C -4.8570 -0.0490 -63.8790

H -4.7240 0.0490 -61.7480

C -7.0790 0.1420 -64.8020

H -8.6620 0.4060 -63.3860

C -5.7070 -0.0190 -64.9850

H -3.7990 -0.1790 -64.0430

H -7.7240 0.1620 -65.6660

C -10.3700 1.2720 -56.9170

H -10.2590 0.5240 -56.1230

H -10.2000 2.2690 -56.4830

C -3.2280 -0.3510 -57.3640

H -3.5850 -1.1910 -57.9770

H -3.1020 0.5160 -58.0300

N -6.5940 -0.3990 -28.7140

N -4.5720 0.1100 -44.5130

H -4.4550 0.1910 -46.1150

C -10.6730 -0.2590 -38.4840

C -10.3860 -0.3750 -39.8350

H -9.3600 -0.3510 -40.1580

C -11.4140 -0.5550 -40.7470

C -12.7530 -0.6420 -40.3140

C -13.0420 -0.4790 -38.9490

C -11.9900 -0.2740 -38.0610

H -12.1960 -0.1580 -37.0100

C -9.9750 -0.4520 -42.6540

H -9.2480 -1.1780 -42.2570

H -9.6320 0.5610 -42.3980

C -10.0540 -0.6180 -44.1700

H -10.5250 -1.5790 -44.3820

H -9.0300 -0.6490 -44.5470

C -10.8160 0.4840 -44.9070

H -10.5090 1.4600 -44.5250

H -10.5200 0.4390 -45.9580

C -12.3360 0.3510 -44.8230

H -12.6710 0.5500 -43.8050

H -12.6160 -0.6780 -45.0610

C -13.0290 1.2910 -45.8100

H -12.7460 2.3240 -45.5920

H -12.6760 1.0630 -46.8190

C -14.5510 1.1670 -45.7800

H -14.9240 1.5270 -44.8180

H -14.8310 0.1140 -45.8680

C -15.2190 1.9600 -46.9030

H -14.8750 2.9960 -46.8670

H -14.9130 1.5440 -47.8660

C -16.7430 1.9340 -46.8030

H -17.0480 2.4160 -45.8710

H -17.0880 0.8980 -46.7710

C -17.4150 2.6500 -47.9730

H -17.0070 3.6600 -48.0610

H -17.1840 2.1200 -48.9000

C -18.9310 2.7340 -47.8000

H -19.3390 1.7280 -47.6800

H -19.1570 3.2970 -46.8900

C -19.6070 3.4140 -48.9870

H -19.4140 2.8300 -49.8910

H -19.1680 4.4030 -49.1340

C -21.1110 3.5570 -48.7870

H -21.5780 2.5810 -48.6640

H -21.5680 4.0500 -49.6430

H -21.3220 4.1510 -47.9000

C -15.0160 -0.7530 -41.1300

H -15.2190 0.1970 -40.6260

H -15.3950 -1.5720 -40.5080

C -15.7060 -0.7520 -42.4900

H -15.5390 -1.7080 -42.9870

H -15.2580 0.0240 -43.1100

C -17.1970 -0.4880 -42.3080

H -17.6360 -1.3020 -41.7270

H -17.3200 0.4310 -41.7300

C -17.9590 -0.3350 -43.6210

H -17.9230 -1.2690 -44.1870

H -17.4820 0.4360 -44.2290

C -19.4100 0.0560 -43.3530

H -19.8880 -0.7240 -42.7580

H -19.4230 0.9750 -42.7620

C -20.2200 0.2840 -44.6250

H -20.2600 -0.6400 -45.2070

H -19.7250 1.0390 -45.2400

C -21.6380 0.7450 -44.2990

H -22.1410 -0.0270 -43.7130

H -21.5870 1.6450 -43.6820

C -22.4590 1.0460 -45.5490

H -22.5120 0.1510 -46.1730

H -21.9570 1.8220 -46.1320

C -23.8720 1.5060 -45.2020

H -24.3820 0.7170 -44.6440

H -23.8160 2.3830 -44.5520

C -24.6870 1.8520 -46.4450

H -24.7330 0.9810 -47.1030

H -24.1830 2.6510 -46.9960

C -26.1050 2.2970 -46.0970

H -26.6120 1.4960 -45.5550

H -26.0590 3.1630 -45.4330

C -26.9080 2.6530 -47.3420

H -26.4310 3.4690 -47.8820

H -27.9160 2.9610 -47.0740

H -26.9750 1.7970 -48.0100

C -14.7030 0.0060 -37.2890

H -14.4090 1.0610 -37.2700

H -14.1940 -0.5110 -36.4670

C -16.2110 -0.1420 -37.0770

H -16.4780 0.4970 -36.2320

H -16.4230 -1.1710 -36.7960

C -17.0530 0.2200 -38.2980

H -16.8500 -0.5010 -39.0890

H -16.7690 1.2070 -38.6650

C -18.5470 0.2100 -37.9840

H -18.8450 -0.7930 -37.6720

H -18.7470 0.8890 -37.1500

C -19.3880 0.6300 -39.1860

H -19.1070 1.6430 -39.4830

H -19.1750 -0.0320 -40.0290

C -20.8850 0.5970 -38.8850

H -21.1970 -0.4330 -38.6950

H -21.0800 1.1740 -37.9770

C -21.7120 1.1780 -40.0290

H -21.5010 0.6290 -40.9490

H -21.4130 2.2170 -40.1890

C -23.2100 1.1320 -39.7390

H -23.5390 0.0930 -39.6760

H -23.4000 1.5980 -38.7680

C -24.0210 1.8630 -40.8060

H -23.7010 2.9070 -40.8400

H -23.8160 1.4230 -41.7850

C -25.5220 1.8120 -40.5300

H -25.7170 2.2030 -39.5280

H -25.8620 0.7740 -40.5500

C -26.3170 2.6290 -41.5450

H -25.9770 3.6660 -41.5080

H -26.1180 2.2500 -42.5510

C -27.8140 2.5830 -41.2700

H -28.1840 1.5620 -41.3180

H -28.3570 3.1800 -42.0000

H -28.0290 2.9760 -40.2780

O -13.6250 -0.9030 -41.3290

O -11.2500 -0.6860 -42.0920

O -14.3430 -0.5610 -38.5390

C -11.7910 1.1730 -57.4030

C -12.1380 1.0090 -58.7300

H -11.3590 0.9800 -59.4730

C -13.4740 0.8510 -59.0720

C -14.4810 0.8130 -58.0880

C -14.1210 1.0280 -56.7450

C -12.7810 1.2240 -56.4360

H -12.4810 1.3800 -55.4140

C -13.0330 1.0320 -61.4020

H -12.2330 0.2770 -61.4570

H -12.5650 2.0110 -61.2210

C -13.7920 1.0570 -62.7260

H -14.3290 0.1140 -62.8390

H -13.0470 1.1240 -63.5220

C -14.7760 2.2230 -62.8540

H -14.3290 3.1230 -62.4250

H -14.9480 2.4180 -63.9160

C -16.1230 1.9500 -62.1870

H -15.9720 1.7400 -61.1280

H -16.5620 1.0570 -62.6400

C -17.0890 3.1210 -62.3650

H -16.7280 3.9850 -61.8010

H -17.1190 3.4070 -63.4200

C -18.5020 2.7700 -61.9070

H -18.4740 2.4650 -60.8590

H -18.8650 1.9180 -62.4880

C -19.4800 3.9330 -62.0610

H -19.1470 4.7710 -61.4440

H -19.4820 4.2700 -63.1000

C -20.8980 3.5340 -61.6570

H -20.8910 3.1810 -60.6240

H -21.2300 2.7050 -62.2860

C -21.8910 4.6870 -61.7820

H -21.5680 5.5150 -61.1470

H -21.8970 5.0480 -62.8130

C -23.3060 4.2670 -61.3870

H -23.6280 3.4410 -62.0260

H -23.3010 3.9050 -60.3570

C -24.3020 5.4170 -61.5110

H -24.3220 5.7640 -62.5460

H -23.9660 6.2520 -60.8920

C -25.7090 5.0080 -61.0920

H -26.0760 4.2010 -61.7220

H -26.3930 5.8500 -61.1810

H -25.7180 4.6640 -60.0600

C -16.8220 0.4350 -57.7020

H -16.9540 1.3600 -57.1340

H -16.6300 -0.3830 -57.0000

C -18.1040 0.1500 -58.4760

H -18.0140 -0.7960 -59.0090

H -18.2600 0.9360 -59.2160

C -19.2750 0.1030 -57.4970

H -19.1380 -0.7400 -56.8150

H -19.2610 1.0150 -56.8950

C -20.6420 -0.0090 -58.1660

H -20.7080 -0.9420 -58.7270

H -20.7690 0.8120 -58.8750

C -21.7550 0.0460 -57.1210

H -21.6470 -0.7980 -56.4350

H -21.6440 0.9610 -56.5350

C -23.1540 0.0220 -57.7310

H -23.2960 -0.9070 -58.2870

H -23.2540 0.8470 -58.4410

C -24.2320 0.1460 -56.6570

H -24.1270 -0.6780 -55.9460

H -24.0820 1.0770 -56.1060

C -25.6420 0.1330 -57.2390

H -25.8050 -0.8040 -57.7760

H -25.7430 0.9460 -57.9620

C -26.7050 0.2860 -56.1550

H -26.5840 -0.5090 -55.4160

H -26.5600 1.2410 -55.6420

C -28.1220 0.2330 -56.7200

H -28.2820 -0.7340 -57.2030

H -28.2370 1.0060 -57.4840

C -29.1800 0.4360 -55.6390

H -29.0640 -0.3340 -54.8720

H -29.0260 1.4070 -55.1620

C -30.5920 0.3730 -56.2080

H -30.7340 1.1460 -56.9600

H -31.3290 0.5190 -55.4210

H -30.7730 -0.5940 -56.6730

C -14.7760 1.4110 -54.4590

H -14.5130 2.4760 -54.4500

H -13.9250 0.8280 -54.0880

C -15.9640 1.1510 -53.5280

H -15.7490 1.6600 -52.5860

H -16.0240 0.0840 -53.3170

C -17.3160 1.6120 -54.0610

H -17.5570 1.0380 -54.9570

H -17.2680 2.6650 -54.3380

C -18.4170 1.4070 -53.0210

H -18.3920 0.3750 -52.6650

H -18.2250 2.0540 -52.1620

C -19.8030 1.7100 -53.5830

H -19.8290 2.7410 -53.9400

H -19.9930 1.0560 -54.4390

C -20.9070 1.5140 -52.5470

H -20.8990 0.4800 -52.1940

H -20.7140 2.1580 -51.6870

C -22.2830 1.8480 -53.1170

H -22.4830 1.2060 -53.9800

H -22.2820 2.8830 -53.4670

C -23.3980 1.6720 -52.0910

H -23.4290 0.6310 -51.7610

H -23.1840 2.2880 -51.2150

C -24.7580 2.0660 -52.6630

H -24.7250 3.1130 -52.9730

H -24.9630 1.4630 -53.5510

C -25.8900 1.8770 -51.6570

H -25.6830 2.4690 -50.7630

H -25.9340 0.8280 -51.3560

C -27.2400 2.2930 -52.2360

H -27.2010 3.3490 -52.5170

H -27.4350 1.7180 -53.1440

C -28.3770 2.0750 -51.2460

H -28.4440 1.0240 -50.9700

H -29.3270 2.3750 -51.6820

H -28.2130 2.6570 -50.3430

O -15.7320 0.5760 -58.5910

O -13.9240 0.7210 -60.3550

O -15.1020 1.0290 -55.7840

C -4.2780 -3.2710 -55.6700

C -5.2180 -3.2350 -56.6280

C -4.9790 -3.3680 -58.0460

C -6.0390 -3.1620 -58.9580

C -3.7240 -3.6970 -58.5570

H -2.9370 -3.9010 -57.8540

C -5.0620 -3.2110 -63.2450

C -4.2070 -3.4570 -62.2390

C -4.5060 -3.4570 -60.8270

C -3.4730 -3.7770 -59.9150

C -5.7770 -3.1760 -60.3160

H -6.5690 -2.9630 -61.0130

O -2.2680 -4.1420 -60.4290

C -9.6230 -2.6050 -58.3980

C -9.5260 -2.3170 -57.0490

H -8.5490 -2.2890 -56.6020

C -10.6670 -2.0590 -56.3030

C -11.9380 -2.1090 -56.9080

C -12.0260 -2.3900 -58.2830

C -10.8660 -2.6500 -59.0040

H -10.9190 -2.8690 -60.0590

C -9.4460 -1.7380 -54.2710

H -8.6370 -1.2940 -54.8660

H -9.1920 -2.7840 -54.0510

C -9.5540 -0.9340 -52.9780

H -9.8530 0.0880 -53.2260

H -8.5460 -0.8980 -52.5580

C -10.5150 -1.5050 -51.9370

H -10.4440 -2.5900 -51.9290

H -10.2090 -1.1440 -50.9520

C -11.9680 -1.1110 -52.1820

H -12.2720 -1.4520 -53.1720

H -12.0530 -0.0200 -52.1590

C -12.8900 -1.7090 -51.1200

H -12.8810 -2.7950 -51.2160

H -12.5090 -1.4540 -50.1270

C -14.3130 -1.1800 -51.2650

H -14.6930 -1.4640 -52.2480

H -14.2910 -0.0880 -51.2240

C -15.2740 -1.6900 -50.1930

H -15.3120 -2.7810 -50.2250

H -14.9090 -1.4000 -49.2050

C -16.6740 -1.1190 -50.4090

H -17.0290 -1.4170 -51.3980

H -16.6200 -0.0270 -50.3990

C -17.6900 -1.5740 -49.3650

H -17.7310 -2.6660 -49.3480

H -17.3740 -1.2380 -48.3750

C -19.0790 -1.0180 -49.6740

H -19.0310 0.0730 -49.7120

H -19.3900 -1.3640 -50.6630

C -20.1290 -1.4380 -48.6490

H -19.8450 -1.0590 -47.6650

H -20.1590 -2.5290 -48.5880

C -21.5110 -0.9120 -49.0170

H -21.5060 0.1740 -49.0650

H -22.2510 -1.2220 -48.2820

H -21.8170 -1.2920 -49.9890

C -14.2430 -2.4150 -56.3670

H -14.1080 -3.4810 -56.5760

H -14.6910 -1.9350 -57.2420

C -15.1580 -2.2310 -55.1650

H -15.3440 -1.1660 -55.0420

H -14.6510 -2.5950 -54.2720

C -16.4850 -2.9600 -55.3720

H -16.8550 -2.7440 -56.3780

H -16.3340 -4.0400 -55.3050

C -17.5390 -2.5230 -54.3580

H -17.6560 -1.4390 -54.4220

H -17.1950 -2.7630 -53.3500

C -18.8970 -3.1770 -54.6030

H -19.1620 -3.0710 -55.6580

H -18.8390 -4.2460 -54.3870

C -19.9890 -2.5340 -53.7510

H -20.0170 -1.4620 -53.9620

H -19.7430 -2.6560 -52.6930

C -21.3690 -3.1280 -54.0210

H -21.5690 -3.1020 -55.0960

H -21.3850 -4.1750 -53.7130

C -22.4690 -2.3620 -53.2900

H -22.4520 -1.3180 -53.6110

H -22.2690 -2.3790 -52.2150

C -23.8540 -2.9460 -53.5540

H -24.0250 -2.9900 -54.6330

H -23.8940 -3.9710 -53.1770

C -24.9600 -2.1210 -52.9010

H -24.9230 -1.0990 -53.2860

H -24.7860 -2.0740 -51.8230

C -26.3460 -2.7060 -53.1600

H -26.5230 -2.7540 -54.2380

H -26.3850 -3.7280 -52.7770

C -27.4430 -1.8770 -52.5030

H -27.3010 -1.8450 -51.4250

H -28.4220 -2.3040 -52.7060

H -27.4270 -0.8560 -52.8800

C -13.4010 -2.7010 -60.2290

H -13.0870 -3.7410 -60.4050

H -12.7760 -2.0370 -60.8410

C -14.8640 -2.5130 -60.6220

H -14.9510 -2.6870 -61.6970

H -15.1420 -1.4780 -60.4130

C -15.7950 -3.4560 -59.8650

H -15.7360 -3.2160 -58.8030

H -15.4580 -4.4870 -59.9890

C -17.2460 -3.3350 -60.3310

H -17.4730 -2.2830 -60.5170

H -17.3770 -3.8670 -61.2760

C -18.2250 -3.8800 -59.2930

H -17.9890 -4.9240 -59.0750

H -18.1010 -3.3160 -58.3640

C -19.6780 -3.7660 -59.7490

H -19.8510 -2.7610 -60.1420

H -19.8630 -4.4710 -60.5630

C -20.6630 -4.0290 -58.6110

H -20.4890 -3.2990 -57.8160

H -20.4820 -5.0210 -58.1910

C -22.1150 -3.9270 -59.0720

H -22.2570 -2.9810 -59.6020

H -22.3290 -4.7320 -59.7780

C -23.0990 -3.9960 -57.9060

H -22.9390 -4.9210 -57.3480

H -22.9060 -3.1630 -57.2260

C -24.5510 -3.9310 -58.3760

H -24.7690 -4.8030 -58.9980

H -24.6900 -3.0420 -58.9970

C -25.5340 -3.8830 -57.2090

H -25.3780 -4.7550 -56.5680

H -25.3330 -2.9920 -56.6110

C -26.9820 -3.8530 -57.6810

H -27.1570 -2.9890 -58.3190

H -27.6610 -3.7970 -56.8330

H -27.2180 -4.7490 -58.2510

O -12.9840 -1.8300 -56.0810

O -10.6650 -1.6990 -54.9910

O -13.2650 -2.3800 -58.8610

O -7.2650 -2.9620 -58.4040

C -4.5080 -3.1610 -54.2490

C -3.4110 -3.2830 -53.3860

C -5.7610 -2.9610 -53.6600

C -3.6100 -3.1930 -52.0260

H -2.4220 -3.4500 -53.7870

C -5.8530 -2.8900 -52.2850

H -6.6500 -2.8650 -54.2600

H -2.7910 -3.2770 -51.3240

H -6.7980 -2.7400 -51.7790

C -4.7350 -3.2450 -64.6550

C -5.7650 -3.1520 -65.5970

C -3.4360 -3.3720 -65.1560

C -5.4610 -3.2080 -66.9440

H -6.7900 -3.0460 -65.2760

C -3.2430 -3.4060 -66.5240

H -2.5870 -3.4340 -64.4940

H -6.2370 -3.1460 -67.6960

H -2.2510 -3.5010 -66.9460

C -1.3110 -4.6540 -59.5230

H -1.0590 -3.8930 -58.7700

H -1.7330 -5.5270 -59.0010

C -8.3900 -2.8170 -59.2400

H -8.2590 -1.9460 -59.9020

H -8.5120 -3.7120 -59.8680

C -6.4790 -3.5500 -29.4000

C -7.7210 -3.5870 -34.9160

C -7.7690 -3.5860 -36.1250

C -7.6070 -3.5620 -37.5200

C -8.6750 -3.5290 -38.4340

C -6.2850 -3.5760 -37.9770

H -5.5180 -3.6410 -37.2220

O -5.3320 -3.3020 -29.0510

O -7.4510 -3.8170 -28.5660

C -6.2420 -2.9330 -48.4660

C -6.7720 -3.2290 -42.8280

C -6.8300 -3.3090 -41.6230

C -7.0460 -3.4120 -40.2360

C -5.9780 -3.5150 -39.3230

C -8.3660 -3.4430 -39.7820

H -9.1390 -3.3940 -40.5320

O -7.1800 -2.9780 -49.2350

O -4.9860 -2.8630 -48.8630

O -4.7190 -3.5560 -39.8370

C -12.3530 -3.6790 -38.2310

C -12.5720 -3.4200 -36.8860

H -11.7170 -3.3020 -36.2400

C -13.8670 -3.2910 -36.4090

C -14.9770 -3.5020 -37.2640

C -14.7390 -3.7450 -38.6190

C -13.4300 -3.8250 -39.0810

H -13.2590 -3.9620 -40.1380

C -13.2470 -2.8470 -34.1200

H -12.3210 -2.3880 -34.4870

H -13.0080 -3.8580 -33.7650

C -13.7870 -2.0010 -32.9600

H -13.6580 -0.9430 -33.1960

H -13.1670 -2.2280 -32.0910

C -15.2640 -2.2480 -32.6290

H -15.5110 -3.2940 -32.8120

H -15.4260 -2.0460 -31.5680

C -16.1860 -1.3540 -33.4560

H -16.0610 -1.6070 -34.5070

H -15.8840 -0.3120 -33.3170

C -17.6600 -1.4890 -33.0800

H -18.0010 -2.5020 -33.3010

H -17.7870 -1.3220 -32.0080

C -18.5080 -0.4820 -33.8570

H -18.3040 -0.6010 -34.9230

H -18.2040 0.5300 -33.5800

C -20.0090 -0.6350 -33.6210

H -20.3110 -1.6590 -33.8540

H -20.2360 -0.4560 -32.5680

C -20.8060 0.3370 -34.4890

H -20.5700 0.1500 -35.5400

H -20.4980 1.3590 -34.2600

C -22.3150 0.2120 -34.2900

H -22.6220 -0.8180 -34.4860

H -22.5640 0.4380 -33.2510

C -23.0870 1.1530 -35.2120

H -22.7750 2.1820 -35.0210

H -22.8390 0.9190 -36.2510

C -24.5980 1.0430 -35.0240

H -24.8520 1.2900 -33.9900

H -24.9080 0.0120 -35.2040

C -25.3570 1.9710 -35.9640

H -25.0890 3.0090 -35.7790

H -26.4300 1.8630 -35.8270

H -25.1220 1.7370 -37.0010

C -17.3640 -3.7330 -37.3540

H -17.3230 -4.7770 -37.6920

H -17.4530 -3.0870 -38.2330

C -18.5880 -3.5280 -36.4680

H -18.5750 -2.5070 -36.0850

H -18.5490 -4.2030 -35.6130

C -19.8540 -3.7630 -37.2930

H -19.7100 -3.3230 -38.2830

H -20.0060 -4.8360 -37.4350

C -21.1100 -3.1460 -36.6820

H -20.9270 -2.0870 -36.4890

H -21.3370 -3.6220 -35.7260

C -22.3030 -3.2800 -37.6280

H -22.0010 -2.9500 -38.6260

H -22.5930 -4.3300 -37.7050

C -23.5040 -2.4480 -37.1840

H -23.1990 -1.4030 -37.0960

H -23.8380 -2.7810 -36.1990

C -24.6610 -2.5460 -38.1760

H -24.2980 -2.2940 -39.1760

H -25.0270 -3.5750 -38.2090

C -25.8090 -1.6070 -37.8130

H -25.4390 -0.5800 -37.7980

H -26.1670 -1.8440 -36.8080

C -26.9720 -1.7040 -38.7970

H -26.6070 -1.5030 -39.8070

H -27.3720 -2.7200 -38.7880

C -28.0860 -0.7160 -38.4600

H -27.6900 0.3010 -38.5010

H -28.4310 -0.8960 -37.4390

C -29.2690 -0.8340 -39.4180

H -28.9230 -0.6660 -40.4400

H -29.6740 -1.8470 -39.3700

C -30.3690 0.1680 -39.0880

H -30.7430 0.0050 -38.0790

H -31.2010 0.0670 -39.7820

H -29.9900 1.1850 -39.1530

C -15.8770 -4.9760 -40.3290

H -16.2550 -5.8090 -39.7140

H -14.8980 -5.2660 -40.7260

C -16.8420 -4.6940 -41.4770

H -16.9250 -5.5960 -42.0860

H -16.4280 -3.8970 -42.0960

C -18.2190 -4.2750 -40.9690

H -18.1090 -3.3490 -40.4030

H -18.6010 -5.0370 -40.2850

C -19.2240 -4.0580 -42.0960

H -18.8090 -3.3600 -42.8260

H -19.4060 -5.0040 -42.6110

C -20.5460 -3.5060 -41.5670

H -20.9410 -4.1810 -40.8030

H -20.3640 -2.5400 -41.0870

C -21.5850 -3.3320 -42.6720

H -21.1650 -2.7170 -43.4710

H -21.8220 -4.3090 -43.1000

C -22.8660 -2.6820 -42.1570

H -22.6330 -1.6890 -41.7600

H -23.2690 -3.2780 -41.3340

C -23.9230 -2.5540 -43.2520

H -23.5020 -2.0090 -44.1000

H -24.1950 -3.5510 -43.6060

C -25.1750 -1.8330 -42.7610

H -25.5800 -2.3640 -41.8960

H -24.9060 -0.8260 -42.4330

C -26.2480 -1.7430 -43.8430

H -26.5430 -2.7510 -44.1440

H -25.8350 -1.2460 -44.7240

C -27.4800 -0.9780 -43.3650

H -27.8830 -1.4700 -42.4760

H -27.1850 0.0340 -43.0810

C -28.5620 -0.9080 -44.4350

H -28.1840 -0.4200 -45.3310

H -29.4210 -0.3460 -44.0750

H -28.8950 -1.9070 -44.7070

O -16.1880 -3.4040 -36.6410

O -14.2150 -2.9340 -35.1450

O -15.7780 -3.8120 -39.5260

O -9.9380 -3.6000 -37.9240

C -7.4870 -3.5880 -33.5300

C -8.5060 -3.6050 -32.5770

C -6.1480 -3.5540 -33.1180

C -8.1930 -3.6030 -31.2340

H -9.5380 -3.6280 -32.8960

C -5.8420 -3.5370 -31.7800

H -5.3690 -3.5350 -33.8660

C -6.8600 -3.5740 -30.8270

H -8.9750 -3.6120 -30.4910

H -4.8200 -3.5000 -31.4380

C -6.6490 -3.1480 -44.2280

C -7.7800 -3.2150 -45.0490

C -5.3870 -2.9970 -44.8060

C -7.6450 -3.1260 -46.4170

H -8.7520 -3.3430 -44.5980

C -5.2580 -2.9120 -46.1730

H -4.5190 -2.9590 -44.1680

C -6.3840 -2.9790 -46.9890

H -8.5060 -3.1700 -47.0660

H -4.2870 -2.7950 -46.6270

C -3.6560 -3.7370 -38.9260

H -3.7120 -2.9710 -38.1390

H -3.7440 -4.7190 -38.4380

C -10.9870 -3.7220 -38.8610

H -10.9440 -2.8950 -39.5810

H -10.8710 -4.6680 -39.4150

N -4.8030 -2.9970 -51.4850

N -4.2270 -3.3340 -67.4070

H -4.9310 -2.8810 -49.8770

C -0.0380 -5.0580 -60.2160

C 0.0560 -5.2400 -61.5850

H -0.8170 -5.0770 -62.1930

C 1.2670 -5.6330 -62.1460

C 2.4040 -5.8410 -61.3390

C 2.3110 -5.5900 -59.9580

C 1.0760 -5.2450 -59.4170

H 0.9780 -5.0930 -58.3560

C 0.3390 -5.8620 -64.3230

H -0.2000 -4.9050 -64.2740

H -0.3400 -6.6580 -63.9900

C 0.7520 -6.1140 -65.7710

H 1.3180 -5.2560 -66.1370

H -0.1720 -6.1800 -66.3500

C 1.5860 -7.3830 -65.9670

H 1.2480 -8.1550 -65.2750

H 1.4280 -7.7570 -66.9820

C 3.0780 -7.1270 -65.7630

H 3.2350 -6.6860 -64.7790

H 3.4110 -6.4070 -66.5160

C 3.9140 -8.4000 -65.8940

H 3.7900 -9.0090 -64.9960

H 3.5620 -8.9900 -66.7440

C 5.3930 -8.0720 -66.0900

H 5.6970 -7.3470 -65.3330

H 5.5280 -7.6050 -67.0680

C 6.2970 -9.2990 -65.9890

H 6.2450 -9.6990 -64.9740

H 5.9400 -10.0750 -66.6700

C 7.7470 -8.9570 -66.3240

H 8.0560 -8.0950 -65.7280

H 7.8160 -8.6690 -67.3760

C 8.7020 -10.1160 -66.0510

H 8.6650 -10.3660 -64.9880

H 8.3750 -10.9960 -66.6100

C 10.1390 -9.7750 -66.4380

H 10.1890 -9.5830 -67.5120

H 10.4430 -8.8580 -65.9270

C 11.1170 -10.8910 -66.0780

H 10.8150 -11.8130 -66.5800

H 11.0750 -11.0690 -65.0010

C 12.5450 -10.5410 -66.4770

H 12.6200 -10.4040 -67.5530

H 13.2310 -11.3320 -66.1820

H 12.8600 -9.6170 -65.9950

C 4.6610 -6.6520 -61.3170

H 4.3970 -7.3020 -60.4790

H 5.1640 -5.7590 -60.9270

C 5.6180 -7.3880 -62.2480

H 5.7920 -6.7800 -63.1350

H 5.1720 -8.3320 -62.5650

C 6.9320 -7.6370 -61.5130

H 7.3730 -6.6700 -61.2530

H 6.7240 -8.1630 -60.5780

C 7.9480 -8.4330 -62.3260

H 8.1000 -7.9470 -63.2910

H 7.5640 -9.4380 -62.5150

C 9.2830 -8.5200 -61.5900

H 9.6410 -7.5060 -61.3930

H 9.1320 -9.0080 -60.6240

C 10.3490 -9.2740 -62.3770

H 10.4410 -8.8360 -63.3730

H 10.0440 -10.3160 -62.5000

C 11.7050 -9.2180 -61.6780

H 12.0050 -8.1720 -61.5690

H 11.6120 -9.6430 -60.6750

C 12.7900 -9.9680 -62.4440

H 12.8750 -9.5540 -63.4510

H 12.5060 -11.0190 -62.5430

C 14.1430 -9.8750 -61.7440

H 14.4290 -8.8240 -61.6580

H 14.0520 -10.2780 -60.7320

C 15.2390 -10.6300 -62.4890

H 15.3300 -10.2310 -63.5020

H 14.9600 -11.6830 -62.5740

C 16.5880 -10.5220 -61.7820

H 16.8670 -9.4690 -61.6980

H 16.4970 -10.9200 -60.7690

C 17.6830 -11.2770 -62.5250

H 17.4330 -12.3330 -62.6010

H 18.6340 -11.1860 -62.0050

H 17.8020 -10.8820 -63.5320

C 3.3740 -5.3250 -57.8190

H 2.8480 -6.1320 -57.2930

H 2.8300 -4.3830 -57.6640

C 4.7930 -5.1680 -57.2760

H 4.7210 -5.0770 -56.1900

H 5.2160 -4.2400 -57.6640

C 5.7240 -6.3210 -57.6390

H 5.7940 -6.3870 -58.7230

H 5.3090 -7.2660 -57.2860

C 7.1190 -6.0990 -57.0580

H 7.4620 -5.0980 -57.3330

H 7.0760 -6.1430 -55.9670

C 8.1310 -7.1200 -57.5710

H 7.8340 -8.1230 -57.2550

H 8.1270 -7.1070 -58.6630

C 9.5430 -6.8180 -57.0730

H 9.8090 -5.7970 -57.3580

H 9.5640 -6.8730 -55.9820

C 10.5790 -7.7800 -57.6490

H 10.5320 -7.7460 -58.7410

H 10.3400 -8.8010 -57.3430

C 11.9950 -7.4300 -57.1970

H 12.2180 -6.4010 -57.4870

H 12.0500 -7.4860 -56.1070

C 13.0420 -8.3590 -57.8050

H 12.8340 -9.3890 -57.5040

H 12.9710 -8.3170 -58.8950

C 14.4590 -7.9820 -57.3780

H 14.5390 -8.0540 -56.2910

H 14.6540 -6.9450 -57.6570

C 15.5150 -8.8780 -58.0210

H 15.3320 -9.9160 -57.7340

H 15.4270 -8.8160 -59.1080

C 16.9260 -8.4790 -57.6100

H 17.1300 -7.4500 -57.9010

H 17.6610 -9.1220 -58.0890

H 17.0500 -8.5600 -56.5320

O 3.5010 -6.2630 -62.0280

O 1.4670 -5.8380 -63.4770

O 3.4460 -5.6330 -59.1990

C -2.3090 -3.6250 -39.5980

C -2.1930 -3.3080 -40.9390

C -0.9280 -3.1720 -41.5110

C 0.2180 -3.3390 -40.7210

C 0.0930 -3.6920 -39.3670

C -1.1770 -3.8340 -38.8190

H -1.2940 -4.1020 -37.7810

C -1.7650 -3.0310 -43.7350

H -2.5640 -2.3210 -43.4880

H -2.1710 -4.0510 -43.6780

C -1.2750 -2.7520 -45.1520

H -0.9810 -1.7040 -45.2300

H -2.1290 -2.9130 -45.8110

C -0.1030 -3.6380 -45.5890

H -0.1560 -4.5960 -45.0680

H -0.1920 -3.8410 -46.6580

C 1.2530 -2.9830 -45.3280

H 1.3440 -2.7520 -44.2670

H 1.2970 -2.0420 -45.8840

C 2.4100 -3.8760 -45.7720

H 2.5080 -4.7140 -45.0780

H 2.1880 -4.2910 -46.7590

C 3.7360 -3.1210 -45.8440

H 3.9230 -2.6220 -44.8900

H 3.6700 -2.3480 -46.6130

C 4.9000 -4.0570 -46.1610

H 4.9760 -4.8090 -45.3720

H 4.6960 -4.5820 -47.0970

C 6.2340 -3.3230 -46.2780

H 6.4080 -2.7400 -45.3700

H 6.1930 -2.6230 -47.1160

C 7.3920 -4.2980 -46.4790

H 7.4080 -5.0060 -45.6470

H 7.2260 -4.8730 -47.3940

C 8.7440 -3.5960 -46.5630

H 8.7490 -2.9230 -47.4240

H 8.8910 -2.9870 -45.6680

C 9.8960 -4.5900 -46.6900

H 9.7610 -5.1880 -47.5940

H 9.8730 -5.2730 -45.8380

C 11.2500 -3.8920 -46.7420

H 11.3100 -3.2360 -47.6070

H 12.0550 -4.6210 -46.8070

H 11.4030 -3.2910 -45.8470

C 2.2440 -4.2760 -41.5200

H 1.8360 -4.8410 -42.3700

H 2.2390 -4.9310 -40.6430

C 3.6550 -3.7910 -41.8210

H 3.9800 -3.1610 -40.9900

H 3.6220 -3.1630 -42.7120

C 4.6500 -4.9290 -42.0200

H 4.6290 -5.5890 -41.1500

H 4.3590 -5.5250 -42.8880

C 6.0610 -4.3790 -42.2220

H 6.3210 -3.7440 -41.3720

H 6.0750 -3.7500 -43.1140

C 7.1170 -5.4720 -42.3630

H 7.0910 -6.1190 -41.4830

H 6.8880 -6.0930 -43.2320

C 8.5150 -4.8750 -42.5170

H 8.7300 -4.2370 -41.6560

H 8.5390 -4.2410 -43.4050

C 9.6000 -5.9420 -42.6320

H 9.5740 -6.5840 -41.7490

H 9.3970 -6.5740 -43.5010

C 10.9900 -5.3240 -42.7700

H 11.1920 -4.6890 -41.9040

H 11.0130 -4.6850 -43.6560

C 12.0850 -6.3810 -42.8870

H 12.0760 -7.0120 -41.9940

H 11.8750 -7.0250 -43.7440

C 13.4680 -5.7560 -43.0540

H 13.6690 -5.0870 -42.2140

H 13.4820 -5.1530 -43.9650

C 14.5700 -6.8100 -43.1320

H 14.5710 -7.4010 -42.2130

H 14.3590 -7.4900 -43.9610

C 15.9440 -6.1810 -43.3320

H 15.9710 -5.6110 -44.2580

H 16.7140 -6.9480 -43.3790

H 16.1800 -5.5080 -42.5110

C 1.2350 -4.4900 -37.4210

H 0.7960 -5.4880 -37.5310

H 0.6290 -3.9090 -36.7100

C 2.6590 -4.5900 -36.8780

H 2.6070 -5.1410 -35.9360

H 3.0140 -3.5850 -36.6550

C 3.6420 -5.2670 -37.8320

H 3.6940 -4.6910 -38.7550

H 3.2950 -6.2670 -38.0920

C 5.0340 -5.3500 -37.2090

H 5.3470 -4.3500 -36.9000

H 4.9930 -5.9750 -36.3130

C 6.0790 -5.9150 -38.1680

H 5.7690 -6.9070 -38.5050

H 6.1400 -5.2760 -39.0520

C 7.4560 -5.9960 -37.5110

H 7.7350 -5.0030 -37.1510

H 7.4040 -6.6580 -36.6420

C 8.5400 -6.4980 -38.4620

H 8.5820 -5.8450 -39.3370

H 8.2820 -7.5010 -38.8110

C 9.9120 -6.5280 -37.7920

H 10.1580 -5.5250 -37.4360

H 9.8750 -7.1870 -36.9220

C 11.0120 -7.0030 -38.7380

H 10.7710 -8.0060 -39.1000

H 11.0530 -6.3410 -39.6060

C 12.3800 -7.0260 -38.0600

H 12.3420 -7.6860 -37.1900

H 12.6210 -6.0220 -37.7020

C 13.4850 -7.4980 -39.0020

H 13.2430 -8.4970 -39.3710

H 13.5320 -6.8300 -39.8650

C 14.8450 -7.5280 -38.3150

H 15.1160 -6.5350 -37.9620

H 15.6160 -7.8670 -39.0030

H 14.8270 -8.2010 -37.4610

O 1.4470 -3.1180 -41.2920

O -0.6970 -2.8900 -42.8180

O 1.2570 -3.8510 -38.6840

H -7.1480 -3.7930 -27.6040

H -3.9210 -0.3450 -39.8710

H -6.9720 -0.1360 -39.9890

H -4.8370 -0.5420 -33.4640

H -7.8760 -0.2570 -33.3110

H -3.2380 -3.4040 -55.9360

H -6.2490 -3.0710 -56.3580

H -3.1780 -3.6950 -62.4650

H -6.1000 -2.9890 -63.0360

H -3.0920 -3.1770 -41.5170

C -5.4970 5.9650 -32.6500

C -4.4800 5.7970 -33.5110

C -4.5190 5.9100 -34.9500

C -3.3010 5.8800 -35.6670

C -5.7030 6.0670 -35.6690

H -6.6220 6.0930 -35.1110

C -3.4910 6.0480 -40.0370

C -4.5360 6.1920 -39.2060

C -4.5060 6.1080 -37.7660

C -5.7230 6.1670 -37.0500

C -3.3180 5.9810 -37.0440

H -2.3910 5.9740 -37.5920

O -6.8580 6.3090 -37.7860

C 0.1680 5.6450 -34.4660

C -0.1430 4.9200 -33.3300

H -1.1730 4.6900 -33.1220

C 0.8680 4.4940 -32.4770

C 2.2130 4.7710 -32.7770

C 2.5250 5.4780 -33.9520

C 1.4890 5.9440 -34.7580

H 1.7050 6.5270 -35.6380

C -0.6270 3.6990 -30.7890

H -1.3370 3.2240 -31.4830

H -0.9760 4.7200 -30.5770

C -0.5780 2.8870 -29.4940

H -0.3610 1.8450 -29.7340

H -1.5740 2.9320 -29.0500

C 0.4680 3.3980 -28.4970

H 0.5210 4.4860 -28.5560

H 0.1520 3.1380 -27.4840

C 1.8500 2.8020 -28.7610

H 2.1150 2.9660 -29.8050

H 1.8110 1.7230 -28.5880

C 2.9340 3.4120 -27.8760

H 2.9890 4.4870 -28.0690

H 2.6770 3.2820 -26.8220

C 4.2930 2.7760 -28.1600

H 4.4560 2.7650 -29.2400

H 4.2810 1.7380 -27.8210

C 5.4480 3.5150 -27.4900

H 5.4550 4.5540 -27.8300

H 5.3010 3.5260 -26.4080

C 6.7940 2.8700 -27.8170

H 6.8920 2.7820 -28.9010

H 6.8200 1.8610 -27.3990

C 7.9750 3.6700 -27.2730

H 7.9440 4.6830 -27.6840

H 7.8890 3.7550 -26.1870

C 9.3120 3.0240 -27.6280

H 9.3550 2.0220 -27.1940

H 9.3810 2.9200 -28.7130

C 10.5030 3.8370 -27.1270

H 10.4310 3.9530 -26.0430

H 10.4670 4.8370 -27.5670

C 11.8310 3.1770 -27.4790

H 11.8970 2.1890 -27.0290

H 12.6650 3.7740 -27.1150

H 11.9310 3.0700 -28.5570

C 4.2040 5.0540 -31.4360

H 4.0870 5.1070 -30.3470

H 4.1430 6.0650 -31.8420

C 5.5430 4.4150 -31.7890

H 5.6450 4.4330 -32.8750

H 5.5160 3.3710 -31.4680

C 6.7490 5.1120 -31.1670

H 6.7890 6.1500 -31.5030

H 6.6570 5.1180 -30.0790

C 8.0310 4.3880 -31.5730

H 8.0620 4.3140 -32.6640

H 8.0050 3.3680 -31.1800

C 9.3070 5.0740 -31.0960

H 9.3570 6.0840 -31.5070

H 9.2950 5.1600 -30.0080

C 10.5380 4.2810 -31.5330

H 10.5160 4.1600 -32.6200

H 10.4920 3.2820 -31.0930

C 11.8540 4.9420 -31.1350

H 11.9180 5.9320 -31.5920

H 11.8800 5.0790 -30.0520

C 13.0530 4.1020 -31.5700

H 13.0260 3.9720 -32.6550

H 12.9800 3.1080 -31.1200

C 14.3820 4.7390 -31.1730

H 14.4500 5.7350 -31.6150

H 14.4140 4.8580 -30.0870

C 15.5780 3.9040 -31.6240

H 15.5470 3.7860 -32.7100

H 15.5110 2.9070 -31.1820

C 16.9060 4.5430 -31.2250

H 16.9730 5.5420 -31.6630

H 16.9360 4.6600 -30.1390

C 18.0990 3.7100 -31.6770

H 18.0570 2.7140 -31.2410

H 19.0320 4.1790 -31.3700

H 18.1090 3.6100 -32.7600

C 4.2120 6.0580 -35.5520

H 4.3180 7.1490 -35.5840

H 3.4470 5.7410 -36.2670

C 5.5140 5.3740 -35.9490

H 5.5880 5.4440 -37.0340

H 5.4320 4.3190 -35.6880

C 6.7650 5.9770 -35.3220

H 6.7010 5.9210 -34.2340

H 6.8290 7.0320 -35.5960

C 8.0020 5.2260 -35.8090

H 7.9330 4.1820 -35.4930

H 8.0130 5.2370 -36.9020

C 9.3150 5.8130 -35.3010

H 9.4050 6.8490 -35.6360

H 9.3180 5.8180 -34.2090

C 10.5080 5.0070 -35.8090

H 10.4250 3.9790 -35.4490

H 10.4790 4.9770 -36.9010

C 11.8460 5.5870 -35.3620

H 11.8710 5.6430 -34.2710

H 11.9470 6.6050 -35.7470

C 13.0180 4.7410 -35.8510

H 12.9220 3.7290 -35.4490

H 12.9790 4.6690 -36.9410

C 14.3660 5.3210 -35.4350

H 14.4770 6.3210 -35.8620

H 14.3950 5.4240 -34.3470

C 15.5290 4.4430 -35.8880

H 15.4910 4.3280 -36.9740

H 15.4220 3.4490 -35.4460

C 16.8840 5.0240 -35.4930

H 17.0050 6.0060 -35.9580

H 16.9160 5.1680 -34.4110

C 18.0320 4.1170 -35.9180

H 17.9310 3.1340 -35.4610

H 18.9890 4.5380 -35.6150

H 18.0390 3.9930 -36.9980

O 3.1410 4.2380 -31.9220

O 0.6650 3.7530 -31.3570

O 3.8420 5.6560 -34.2390

O -2.1690 5.7260 -34.9190

C -5.3980 5.8740 -31.2080

C -6.5140 6.1740 -30.4180

C -4.2360 5.4970 -30.5260

C -6.4150 6.0990 -29.0420

H -7.4430 6.4700 -30.8810

C -4.2450 5.4550 -29.1450

H -3.3350 5.2500 -31.0610

H -7.2570 6.3340 -28.4050

H -3.3610 5.1740 -28.5880

C -3.5260 6.1490 -41.4780

C -2.3180 6.0950 -42.1830

C -4.6890 6.3010 -42.2410

C -2.3260 6.1660 -43.5570

H -1.3850 6.0040 -41.6530

C -4.5910 6.3700 -43.6170

H -5.6570 6.3530 -41.7700

H -1.4110 6.1110 -44.1310

H -5.4590 6.4900 -44.2510

C -8.0790 6.4260 -37.0910

H -8.2300 5.5380 -36.4600

H -8.0540 7.3180 -36.4470

C -0.9050 6.0530 -35.4490

H -0.7100 5.5220 -36.3930

H -0.8560 7.1330 -35.6590

C -4.5270 6.6110 -47.3810

C -4.8690 6.8910 -53.0340

C -5.0650 6.9610 -54.2260

C -5.4270 7.0470 -55.5830

C -4.5280 6.7380 -56.6210

C -6.7290 7.4530 -55.8840

H -7.3570 7.7230 -55.0500

O -5.4690 6.8840 -46.6690

O -3.3530 6.2310 -46.9090

C -5.9860 6.3400 -66.3640

C -6.6830 6.9360 -60.7800

C -6.6480 7.0750 -59.5780

C -6.3210 7.1290 -58.2120

C -7.2070 7.4990 -57.1860

C -5.0010 6.7820 -57.9170

H -4.3770 6.5340 -58.7600

O -4.8570 6.3360 -66.8340

O -7.0750 6.2620 -67.0850

H -6.8790 6.2510 -68.0750

O -8.4600 7.8560 -57.5540

C -0.9670 5.8830 -56.9360

C -0.6700 5.5720 -55.6230

H -1.4510 5.6650 -54.8870

C 0.6200 5.1620 -55.2910

C 1.5980 5.0720 -56.2940

C 1.2800 5.3580 -57.6300

C -0.0070 5.7830 -57.9350

H -0.2740 6.0350 -58.9500

C 0.2630 5.0170 -52.9110

H -0.8010 4.8430 -53.1120

H 0.3890 6.0610 -52.5900

C 0.7070 4.0650 -51.7990

H 0.4660 3.0430 -52.0970

H 0.1080 4.3110 -50.9200

C 2.1950 4.1380 -51.4480

H 2.5340 5.1730 -51.5060

H 2.3190 3.8070 -50.4140

C 3.0710 3.2600 -52.3430

H 3.0410 3.6330 -53.3660

H 2.6700 2.2430 -52.3450

C 4.5120 3.2170 -51.8390

H 4.9270 4.2280 -51.8430

H 4.5190 2.8650 -50.8040

C 5.4090 2.3070 -52.6750

H 5.4180 2.6600 -53.7080

H 5.0010 1.2940 -52.6760

C 6.8360 2.2810 -52.1320

H 7.2100 3.3060 -52.0700

H 6.8260 1.8720 -51.1190

C 7.7940 1.4600 -52.9920

H 7.8180 1.8740 -54.0030

H 7.4340 0.4310 -53.0630

C 9.2050 1.4700 -52.4090

H 9.5390 2.5060 -52.3080

H 9.1820 1.0320 -51.4080

C 10.2110 0.7070 -53.2660

H 9.8930 -0.3340 -53.3600

H 10.2340 1.1400 -54.2690

C 11.6140 0.7560 -52.6650

H 11.5880 0.3350 -51.6580

H 11.9290 1.7990 -52.5800

C 12.6280 -0.0070 -53.5070

H 12.3520 -1.0560 -53.5820

H 13.6190 0.0540 -53.0630

H 12.6790 0.4110 -54.5100

C 3.8680 5.6790 -56.0590

H 3.5030 6.6340 -55.6630

H 4.1070 5.8160 -57.1210

C 5.0910 5.1830 -55.2950

H 5.0750 4.0950 -55.3730

H 4.9970 5.4400 -54.2400

C 6.4150 5.6980 -55.8480

H 6.4350 5.5500 -56.9310

H 6.5140 6.7680 -55.6560

C 7.5860 4.9420 -55.2250

H 7.4040 3.8700 -55.3310

H 7.6370 5.1610 -54.1570

C 8.9250 5.2720 -55.8770

H 8.8490 5.1110 -56.9560

H 9.1670 6.3240 -55.7150

C 10.0430 4.3940 -55.3210

H 9.7740 3.3440 -55.4630

H 10.1360 4.5640 -54.2460

C 11.3860 4.6600 -55.9930

H 11.2830 4.5210 -57.0720

H 11.6780 5.6980 -55.8200

C 12.4760 3.7300 -55.4690

H 12.1810 2.6940 -55.6510

H 12.5730 3.8570 -54.3880

C 13.8270 3.9920 -56.1290

H 13.7230 3.8910 -57.2130

H 14.1350 5.0190 -55.9220

C 14.9040 3.0310 -55.6330

H 14.5870 2.0020 -55.8220

H 15.0190 3.1460 -54.5530

C 16.2500 3.2720 -56.3120

H 16.1390 3.1440 -57.3910

H 16.5670 4.3020 -56.1340

C 17.3210 2.3190 -55.7960

H 17.4720 2.4570 -54.7280

H 18.2690 2.4960 -56.3010

H 17.0270 1.2860 -55.9680

C 2.0330 5.4820 -59.9020

H 1.8640 6.5620 -60.0030

H 1.1400 4.9540 -60.2600

C 3.2380 5.0500 -60.7420

H 3.1650 5.5530 -61.7090

H 3.1810 3.9750 -60.9150

C 4.5710 5.3730 -60.0720

H 4.6000 4.8540 -59.1130

H 4.6310 6.4440 -59.8770

C 5.7620 4.9220 -60.9130

H 5.6160 3.8790 -61.1990

H 5.8110 5.5130 -61.8300

C 7.0840 5.0390 -60.1570

H 7.2710 6.0830 -59.8990

H 7.0080 4.4760 -59.2230

C 8.2580 4.4890 -60.9640

H 8.0240 3.4700 -61.2790

H 8.3940 5.0920 -61.8640

C 9.5590 4.4710 -60.1640

H 9.4130 3.8800 -59.2560

H 9.8160 5.4880 -59.8600

C 10.7110 3.8720 -60.9680

H 10.4350 2.8630 -61.2860

H 10.8720 4.4720 -61.8660

C 12.0100 3.8030 -60.1700

H 12.3040 4.8110 -59.8660

H 11.8430 3.2180 -59.2620

C 13.1410 3.1650 -60.9730

H 13.3140 3.7470 -61.8810

H 12.8400 2.1590 -61.2770

C 14.4420 3.0780 -60.1800

H 14.7570 4.0850 -59.8910

H 14.2660 2.5100 -59.2640

C 15.5510 2.4080 -60.9800

H 15.2610 1.3970 -61.2590

H 16.4680 2.3530 -60.3980

H 15.7570 2.9680 -61.8900

O 2.8630 4.6760 -55.9220

O 1.0490 4.7910 -54.0620

O 2.2720 5.1710 -58.5390

O -3.2430 6.4260 -56.2860

C -4.7300 6.8410 -51.6330

C -3.5610 6.3850 -51.0210

C -5.8120 7.2440 -50.8420

C -3.4860 6.3120 -49.6460

H -2.7230 6.0860 -51.6340

C -5.7350 7.1610 -49.4710

H -6.7000 7.6240 -51.3220

C -4.5750 6.6870 -48.8630

H -2.5910 5.9550 -49.1620

H -6.5610 7.4600 -48.8450

C -6.5780 6.7710 -62.1720

C -5.2930 6.5760 -62.6980

C -7.6790 6.7960 -63.0320

C -5.1170 6.4310 -64.0530

H -4.4500 6.5520 -62.0250

C -7.4980 6.6370 -64.3890

H -8.6690 6.9440 -62.6230

C -6.2170 6.4610 -64.9090

H -4.1360 6.2920 -64.4790

H -8.3400 6.6560 -65.0640

C -9.3160 8.3820 -56.5630

H -9.3090 7.7420 -55.6730

H -8.9670 9.3870 -56.2800

C -2.3520 6.2810 -57.3660

H -2.7210 5.5050 -58.0530

H -2.2980 7.2280 -57.9260

N -5.3060 5.7480 -28.4090

N -3.4380 6.2960 -44.2640

H -3.3770 6.2330 -45.8960

C -9.2530 6.5340 -38.0280

C -9.1320 6.5120 -39.4040

H -8.1510 6.4450 -39.8420

C -10.2690 6.5710 -40.1990

C -11.5530 6.6420 -39.6250

C -11.6670 6.6450 -38.2230

C -10.5090 6.6150 -37.4560

H -10.5750 6.6370 -36.3820

C -9.0140 6.7770 -42.2150

H -8.3000 5.9750 -41.9740

H -8.5880 7.7330 -41.8760

C -9.2200 6.8060 -43.7280

H -9.5220 5.8120 -44.0620

H -8.2510 7.0280 -44.1790

C -10.2620 7.8290 -44.1930

H -10.2660 8.6850 -43.5150

H -9.9810 8.1890 -45.1850

C -11.6670 7.2330 -44.2650

H -11.9650 6.9000 -43.2700

H -11.6440 6.3610 -44.9230

C -12.6890 8.2300 -44.8060

H -12.8270 9.0370 -44.0810

H -12.3100 8.6780 -45.7280

C -14.0340 7.5630 -45.0880

H -14.3470 7.0080 -44.2010

H -13.9150 6.8430 -45.9020

C -15.1220 8.5680 -45.4540

H -15.2210 9.2960 -44.6450

H -14.8280 9.1150 -46.3540

C -16.4710 7.8900 -45.6850

H -16.7080 7.2710 -44.8170

H -16.4030 7.2310 -46.5530

C -17.5970 8.8990 -45.8960

H -17.6130 9.5960 -45.0540

H -17.4020 9.4800 -46.8010

C -18.9610 8.2230 -46.0070

H -18.9630 7.5450 -46.8620

H -19.1340 7.6240 -45.1100

C -20.0930 9.2350 -46.1580

H -19.9280 9.8300 -47.0580

H -20.0810 9.9160 -45.3030

C -21.4560 8.5610 -46.2390

H -21.5110 7.8990 -47.0990

H -22.2490 9.3020 -46.3250

H -21.6350 7.9720 -45.3430

C -13.8790 6.9760 -40.0890

H -13.8720 7.8430 -39.4200

H -14.2510 6.1080 -39.5350

C -14.8120 7.2620 -41.2610

H -14.7990 6.4190 -41.9500

H -14.4690 8.1470 -41.7970

C -16.2260 7.4740 -40.7220

H -16.5400 6.5670 -40.1980

H -16.2060 8.2860 -39.9910

C -17.2620 7.8060 -41.7920

H -17.3370 6.9790 -42.5000

H -16.9470 8.6920 -42.3450

C -18.6270 8.0600 -41.1560

H -18.9430 7.1630 -40.6170

H -18.5340 8.8680 -40.4270

C -19.7010 8.4340 -42.1730

H -19.8290 7.6140 -42.8830

H -19.3770 9.3120 -42.7370

C -21.0390 8.7320 -41.5020

H -21.3700 7.8460 -40.9520

H -20.9080 9.5410 -40.7790

C -22.1130 9.1250 -42.5100

H -22.2360 8.3190 -43.2350

H -21.7890 10.0130 -43.0580

C -23.4610 9.4040 -41.8510

H -23.7980 8.5020 -41.3310

H -23.3470 10.1950 -41.1070

C -24.5180 9.8180 -42.8720

H -24.5880 9.0540 -43.6480

H -24.2080 10.7480 -43.3560

C -25.8910 10.0120 -42.2350

H -26.1990 9.0790 -41.7570

H -25.8270 10.7780 -41.4580

C -26.9420 10.4170 -43.2600

H -26.6650 11.3520 -43.7440

H -27.9100 10.5530 -42.7840

H -27.0400 9.6520 -44.0270

C -13.0240 6.8700 -36.2540

H -12.6400 7.8700 -36.0040

H -12.4470 6.1170 -35.7040

C -14.4870 6.7270 -35.8420

H -14.5550 6.9730 -34.7800

H -14.7650 5.6810 -35.9650

C -15.4420 7.6100 -36.6420

H -15.4490 7.2700 -37.6780

H -15.0810 8.6400 -36.6330

C -16.8650 7.5660 -36.0880

H -17.1760 6.5260 -35.9740

H -16.8850 8.0240 -35.0960

C -17.8610 8.2790 -37.0000

H -17.5680 9.3240 -37.1150

H -17.8340 7.8180 -37.9900

C -19.2880 8.2120 -36.4590

H -19.5830 7.1660 -36.3540

H -19.3210 8.6640 -35.4640

C -20.2850 8.9220 -37.3720

H -20.2310 8.4870 -38.3720

H -20.0120 9.9760 -37.4550

C -21.7160 8.8110 -36.8530

H -22.0000 7.7570 -36.8070

H -21.7650 9.2110 -35.8370

C -22.7110 9.5580 -37.7380

H -22.4480 10.6190 -37.7590

H -22.6400 9.1790 -38.7600

C -24.1470 9.4050 -37.2440

H -24.2140 9.7550 -36.2100

H -24.4200 8.3470 -37.2530

C -25.1400 10.1830 -38.1030

H -24.8860 11.2450 -38.0720

H -25.0570 9.8500 -39.1400

C -26.5740 9.9940 -37.6260

H -26.8570 8.9450 -37.6740

H -27.2630 10.5640 -38.2450

H -26.6810 10.3280 -36.5950

O -12.5670 6.7080 -40.5410

O -10.2470 6.5530 -41.5630

O -12.9100 6.6600 -37.6500

C -10.7400 8.4570 -57.0400

C -11.1090 8.1210 -58.3280

H -10.3400 7.8360 -59.0250

C -12.4500 8.1240 -58.6810

C -13.4420 8.4640 -57.7440

C -13.0500 8.8490 -56.4520

C -11.7010 8.8430 -56.1240

H -11.3850 9.1320 -55.1370

C -11.9920 7.6920 -60.9790

H -11.2190 6.9430 -60.7490

H -11.4920 8.6610 -61.1280

C -12.7120 7.2710 -62.2590

H -13.1290 6.2720 -62.1150

H -11.9540 7.2100 -63.0430

C -13.8310 8.2210 -62.6960

H -13.5580 9.2490 -62.4490

H -13.9350 8.1580 -63.7820

C -15.1780 7.8810 -62.0600

H -15.1230 8.0420 -60.9830

H -15.3880 6.8210 -62.2320

C -16.3160 8.7060 -62.6590

H -16.1980 9.7540 -62.3750

H -16.2620 8.6550 -63.7510

C -17.6850 8.2030 -62.2090

H -17.7390 8.2290 -61.1190

H -17.8030 7.1610 -62.5180

C -18.8300 9.0290 -62.7920

H -18.7310 10.0680 -62.4700

H -18.7660 9.0170 -63.8830

C -20.1930 8.4920 -62.3620

H -20.2350 8.4470 -61.2710

H -20.3140 7.4720 -62.7340

C -21.3440 9.3560 -62.8720

H -21.2330 10.3720 -62.4850

H -21.2990 9.4130 -63.9620

C -22.7020 8.7980 -62.4520

H -22.8260 7.7990 -62.8730

H -22.7310 8.6980 -61.3640

C -23.8590 9.6860 -62.9040

H -23.8280 9.7910 -63.9910

H -23.7370 10.6820 -62.4720

C -25.2110 9.1160 -62.4920

H -25.3830 8.1540 -62.9690

H -26.0150 9.7900 -62.7810

H -25.2540 8.9690 -61.4150

C -15.8160 8.2710 -57.3500

H -15.9460 9.1650 -56.7310

H -15.6400 7.4150 -56.6890

C -17.0720 8.0510 -58.1870

H -17.0070 7.0900 -58.6960

H -17.1140 8.8290 -58.9500

C -18.3340 8.1110 -57.3310

H -18.3270 7.2920 -56.6080

H -18.3420 9.0450 -56.7630

C -19.5990 8.0270 -58.1810

H -19.6050 7.0830 -58.7290

H -19.5880 8.8360 -58.9140

C -20.8690 8.1340 -57.3420

H -20.9160 7.2950 -56.6440

H -20.8370 9.0520 -56.7490

C -22.1250 8.1370 -58.2090

H -22.1570 7.2190 -58.8010

H -22.0750 8.9770 -58.9060

C -23.4020 8.2490 -57.3810

H -23.4680 7.3960 -56.7010

H -23.3600 9.1550 -56.7710

C -24.6470 8.2870 -58.2610

H -24.6930 7.3760 -58.8630

H -24.5740 9.1330 -58.9480

C -25.9320 8.4130 -57.4480

H -26.0030 7.5750 -56.7500

H -25.8970 9.3330 -56.8580

C -27.1680 8.4310 -58.3420

H -27.2110 7.5020 -58.9170

H -27.0860 9.2540 -59.0540

C -28.4590 8.5880 -57.5430

H -28.5470 7.7650 -56.8290

H -28.4190 9.5190 -56.9730

C -29.6850 8.6070 -58.4470

H -29.6260 9.4350 -59.1500

H -30.5930 8.7200 -57.8600

H -29.7560 7.6810 -59.0140

C -13.6690 9.4730 -54.2160

H -13.1420 10.4330 -54.1390

H -13.0170 8.6730 -53.8450

C -14.9550 9.4910 -53.3940

H -14.6930 9.6850 -52.3530

H -15.4040 8.5000 -53.4530

C -15.9800 10.5120 -53.8820

H -16.0130 10.4750 -54.9720

H -15.6760 11.5180 -53.5950

C -17.3710 10.2100 -53.3320

H -17.6170 9.1680 -53.5490

H -17.3700 10.3330 -52.2470

C -18.4480 11.0980 -53.9450

H -18.2800 12.1390 -53.6620

H -18.3790 11.0420 -55.0350

C -19.8470 10.6650 -53.5140

H -19.9860 9.6110 -53.7670

H -19.9380 10.7600 -52.4290

C -20.9420 11.4850 -54.1880

H -20.8060 11.4440 -55.2720

H -20.8530 12.5320 -53.8880

C -22.3360 10.9710 -53.8400

H -22.4210 9.9250 -54.1460

H -22.4780 11.0090 -52.7570

C -23.4330 11.7850 -54.5210

H -23.3630 12.8260 -54.1970

H -23.2760 11.7680 -55.6030

C -24.8270 11.2500 -54.2050

H -24.9810 11.2580 -53.1240

H -24.9010 10.2120 -54.5390

C -25.9200 12.0770 -54.8760

H -25.8540 13.1100 -54.5280

H -25.7540 12.0810 -55.9560

C -27.3110 11.5310 -54.5780

H -27.3960 10.4980 -54.9090

H -28.0710 12.1200 -55.0870

H -27.5130 11.5620 -53.5100

O -14.7220 8.4360 -58.2330

O -12.9180 7.7920 -59.9240

O -14.0190 9.2250 -55.5670

C -3.7420 3.1310 -54.9150

C -4.7300 3.4710 -55.7570

C -4.6430 3.4000 -57.1970

C -5.7880 3.6300 -57.9900

C -3.4440 3.1120 -57.8490

H -2.5670 2.9740 -57.2420

C -5.3900 3.3130 -62.3420

C -4.3860 3.1740 -61.4600

C -4.4930 3.2470 -60.0200

C -3.3460 3.0170 -59.2260

C -5.6900 3.5510 -59.3680

H -6.5590 3.7320 -59.9750

O -2.1820 2.7060 -59.8680

C -9.2190 4.5930 -57.1540

C -9.0010 4.9060 -55.8250

H -8.0070 4.8210 -55.4220

C -10.0640 5.3390 -55.0380

C -11.3500 5.4500 -55.5870

C -11.5680 5.1060 -56.9320

C -10.4920 4.6740 -57.6950

H -10.6240 4.4000 -58.7280

C -8.8180 5.2700 -53.0010

H -7.8990 5.7340 -53.3920

H -8.7190 4.1790 -53.0930

C -8.9820 5.6590 -51.5330

H -8.9360 6.7450 -51.4480

H -8.1270 5.2430 -50.9960

C -10.2920 5.1730 -50.9010

H -10.6090 4.2450 -51.3810

H -10.1160 4.9560 -49.8460

C -11.4040 6.2150 -51.0120

H -11.5710 6.4490 -52.0640

H -11.0780 7.1290 -50.5090

C -12.7110 5.7410 -50.3790

H -13.1260 4.9260 -50.9770

H -12.5130 5.3470 -49.3790

C -13.7370 6.8710 -50.2820

H -13.7880 7.3890 -51.2430

H -13.4070 7.5990 -49.5370

C -15.1280 6.3580 -49.9170

H -15.4230 5.5930 -50.6400

H -15.0950 5.8830 -48.9340

C -16.1800 7.4660 -49.9160

H -16.1290 8.0070 -50.8650

H -15.9630 8.1810 -49.1190

C -17.5890 6.9060 -49.7370

H -17.7590 6.1290 -50.4880

H -17.6700 6.4360 -48.7540

C -18.6700 7.9750 -49.8810

H -18.5460 8.7230 -49.0960

H -18.5470 8.4800 -50.8430

C -20.0740 7.3800 -49.8050

H -20.2140 6.9030 -48.8330

H -20.1740 6.6030 -50.5650

C -21.1590 8.4300 -50.0160

H -21.1260 9.1810 -49.2310

H -22.1440 7.9690 -50.0070

H -21.0230 8.9310 -50.9730

C -13.4390 5.0750 -54.5290

H -13.1570 4.3550 -53.7450

H -13.7010 4.5230 -55.4370

C -14.6080 5.9400 -54.0830

H -14.7170 6.7310 -54.8260

H -14.3520 6.4040 -53.1310

C -15.9220 5.1770 -53.9530

H -16.1000 4.6090 -54.8690

H -15.8600 4.4670 -53.1250

C -17.0890 6.1360 -53.7240

H -17.1270 6.8450 -54.5560

H -16.9110 6.7070 -52.8110

C -18.4380 5.4300 -53.6140

H -18.5730 4.7670 -54.4710

H -18.4570 4.8140 -52.7120

C -19.5890 6.4330 -53.5720

H -19.6110 6.9900 -54.5130

H -19.4100 7.1540 -52.7720

C -20.9450 5.7680 -53.3540

H -21.0950 4.9900 -54.1060

H -20.9600 5.2820 -52.3750

C -22.0860 6.7790 -53.4390

H -22.1290 7.1840 -54.4530

H -21.8820 7.6120 -52.7630

C -23.4400 6.1710 -53.0820

H -23.6160 5.2860 -53.6980

H -23.4290 5.8480 -52.0390

C -24.5770 7.1670 -53.2960

H -24.6070 7.4570 -54.3500

H -24.3800 8.0720 -52.7150

C -25.9360 6.5980 -52.8950

H -26.1070 5.6580 -53.4240

H -25.9350 6.3770 -51.8250

C -27.0680 7.5680 -53.2140

H -26.9170 8.5140 -52.6980

H -28.0260 7.1570 -52.9050

H -27.1090 7.7670 -54.2830

C -13.0950 4.6630 -58.6940

H -12.8600 3.5880 -58.6810

H -12.4710 5.1570 -59.4470

C -14.5590 4.8760 -59.0590

H -14.7370 4.3390 -59.9930

H -14.7260 5.9400 -59.2340

C -15.5200 4.3680 -57.9870

H -15.5450 5.0900 -57.1700

H -15.1510 3.4220 -57.5850

C -16.9260 4.1690 -58.5530

H -17.1510 4.9780 -59.2520

H -16.9510 3.2350 -59.1170

C -18.0000 4.1480 -57.4700

H -17.7990 3.3400 -56.7620

H -17.9610 5.0880 -56.9150

C -19.3950 3.9750 -58.0620

H -19.5560 4.7450 -58.8210

H -19.4600 3.0060 -58.5590

C -20.4960 4.0790 -57.0120

H -20.3890 5.0240 -56.4750

H -20.3880 3.2700 -56.2850

C -21.8860 4.0170 -57.6400

H -21.9750 4.8150 -58.3810

H -22.0050 3.0650 -58.1610

C -22.9990 4.1710 -56.6080

H -22.9360 3.3570 -55.8810

H -22.8560 5.1080 -56.0660

C -24.3820 4.1710 -57.2530

H -24.5290 3.2330 -57.7930

H -24.4360 4.9860 -57.9780

C -25.4970 4.3420 -56.2260

H -25.4360 3.5360 -55.4890

H -25.3510 5.2850 -55.6950

C -26.8770 4.3350 -56.8700

H -26.9700 5.1500 -57.5850

H -27.6530 4.4520 -56.1160

H -27.0490 3.3980 -57.3950

O -12.3430 5.9450 -54.7780

O -9.9500 5.7030 -53.7310

O -12.8340 5.2070 -57.4110

O -6.9340 3.9140 -57.3220

C -3.7650 3.1620 -53.4730

C -2.6100 2.7470 -52.7970

C -4.8490 3.5640 -52.6850

C -2.5890 2.7450 -51.4210

H -1.7450 2.4320 -53.3630

C -4.7230 3.5380 -51.3080

H -5.7690 3.8930 -53.1370

H -1.7210 2.4190 -50.8650

H -5.5290 3.8380 -50.6510

C -5.2790 3.2290 -63.7840

C -6.4390 3.2970 -64.5630

C -4.0720 3.0770 -64.4740

C -6.3420 3.2000 -65.9380

H -7.4020 3.4240 -64.0910

C -4.0900 2.9960 -65.8540

H -3.1330 3.0290 -63.9460

H -7.2230 3.2460 -66.5650

H -3.1730 2.8780 -66.4150

C -1.0370 2.4940 -59.0690

H -0.7850 3.4140 -58.5190

H -1.2280 1.6990 -58.3310

C -8.0970 4.1850 -58.0690

H -7.9100 5.0070 -58.7770

H -8.3940 3.2980 -58.6460

C -6.1790 2.8870 -28.4770

C -6.2960 2.8140 -34.1280

C -6.1050 2.8270 -35.3220

C -5.6910 2.8530 -36.6630

C -6.5790 2.9530 -37.7480

C -4.3140 2.7790 -36.8860

H -3.6910 2.6880 -36.0110

O -5.1130 2.7370 -27.8960

O -7.3040 3.1720 -27.8710

C -4.3540 3.3410 -47.4550

C -4.1410 3.0630 -41.7950

C -4.2650 2.9940 -40.5930

C -4.6630 2.9330 -39.2450

C -3.7770 2.8310 -38.1590

C -6.0400 2.9840 -39.0210

H -6.6650 3.0530 -39.8960

O -5.3950 3.5450 -48.0410

O -3.2070 3.1400 -48.0740

O -2.4420 2.7980 -38.4360

C -10.2150 3.1760 -38.1650

C -10.6150 3.2200 -36.8450

H -9.8660 3.1780 -36.0720

C -11.9640 3.3330 -36.5400

C -12.9370 3.4060 -37.5550

C -12.5180 3.3630 -38.8980

C -11.1620 3.2360 -39.1690

H -10.8170 3.1940 -40.1870

C -11.6360 2.7990 -34.2500

H -10.6930 3.3520 -34.1280

H -11.3900 1.7670 -34.5420

C -12.3770 2.8010 -32.9130

H -12.3900 3.8170 -32.5140

H -11.7960 2.1810 -32.2270

C -13.8180 2.2890 -33.0090

H -13.8970 1.5670 -33.8240

H -14.0840 1.7810 -32.0790

C -14.7980 3.4340 -33.2520

H -14.5260 3.9330 -34.1810

H -14.7050 4.1540 -32.4350

C -16.2470 2.9630 -33.3320

H -16.3870 2.3850 -34.2470

H -16.4700 2.3090 -32.4850

C -17.2140 4.1460 -33.3180

H -16.9090 4.8670 -34.0800

H -17.1510 4.6500 -32.3510

C -18.6570 3.7230 -33.5740

H -18.7180 3.2370 -34.5510

H -18.9600 2.9930 -32.8190

C -19.6170 4.9100 -33.5400

H -19.2730 5.6730 -34.2430

H -19.6050 5.3580 -32.5440

C -21.0440 4.5020 -33.8970

H -21.0450 4.0420 -34.8890

H -21.3940 3.7510 -33.1850

C -22.0040 5.6880 -33.8930

H -22.0300 6.1300 -32.8940

H -21.6350 6.4550 -34.5790

C -23.4170 5.2830 -34.3030

H -23.7900 4.5230 -33.6120

H -23.3850 4.8360 -35.3000

C -24.3700 6.4720 -34.3130

H -24.4470 6.9080 -33.3200

H -25.3650 6.1640 -34.6290

H -24.0140 7.2410 -34.9950

C -15.2880 3.7190 -37.9810

H -15.3370 2.9330 -38.7400

H -15.1220 4.6780 -38.4840

C -16.6110 3.7670 -37.2230

H -16.5150 4.4240 -36.3600

H -16.8600 2.7720 -36.8550

C -17.7010 4.2790 -38.1600

H -17.4650 5.3080 -38.4440

H -17.6960 3.6780 -39.0730

C -19.1020 4.2460 -37.5580

H -19.1120 4.8090 -36.6230

H -19.3810 3.2160 -37.3290

C -20.1170 4.8470 -38.5270

H -19.8580 5.8930 -38.7090

H -20.0530 4.3210 -39.4830

C -21.5520 4.7740 -38.0140

H -21.6140 5.2690 -37.0430

H -21.8370 3.7290 -37.8720

C -22.5240 5.4390 -38.9840

H -22.2620 6.4950 -39.0840

H -22.4200 4.9790 -39.9710

C -23.9760 5.3250 -38.5300

H -24.0800 5.7590 -37.5320

H -24.2530 4.2710 -38.4610

C -24.9270 6.0370 -39.4880

H -24.6720 7.0990 -39.5250

H -24.7960 5.6310 -40.4940

C -26.3870 5.8870 -39.0700

H -26.5170 6.2870 -38.0620

H -26.6450 4.8260 -39.0370

C -27.3340 6.6070 -40.0270

H -27.0810 7.6690 -40.0490

H -27.1970 6.2100 -41.0360

C -28.7920 6.4470 -39.6150

H -29.0750 5.3970 -39.6090

H -29.4450 6.9730 -40.3070

H -28.9540 6.8500 -38.6170

C -13.1370 2.9940 -41.2000

H -13.1080 1.8960 -41.1770

H -12.1640 3.3620 -41.5360

C -14.1780 3.4960 -42.2000

H -14.0260 2.9450 -43.1300

H -13.9780 4.5500 -42.4000

C -15.6210 3.3410 -41.7290

H -15.7760 3.9860 -40.8630

H -15.7960 2.3100 -41.4160

C -16.6200 3.7190 -42.8220

H -16.3220 4.6710 -43.2680

H -16.5960 2.9660 -43.6130

C -18.0430 3.8450 -42.2830

H -18.3390 2.9040 -41.8120

H -18.0650 4.6210 -41.5140

C -19.0470 4.1990 -43.3780

H -18.7070 5.0970 -43.9010

H -19.0880 3.3890 -44.1100

C -20.4460 4.4460 -42.8180

H -20.3970 5.2380 -42.0660

H -20.8030 3.5410 -42.3190

C -21.4410 4.8500 -43.9050

H -21.0690 5.7410 -44.4170

H -21.5110 4.0530 -44.6480

C -22.8290 5.1370 -43.3360

H -23.2180 4.2330 -42.8610

H -22.7490 5.9060 -42.5630

C -23.8090 5.6060 -44.4090

H -23.8930 4.8410 -45.1840

H -23.4220 6.5100 -44.8840

C -25.1930 5.8990 -43.8350

H -25.6070 4.9840 -43.4050

H -25.1020 6.6320 -43.0300

C -26.1480 6.4330 -44.8960

H -25.7620 7.3530 -45.3290

H -27.1240 6.6400 -44.4630

H -26.2750 5.7080 -45.6980

O -14.2210 3.4730 -37.0850

O -12.4360 3.3870 -35.2550

O -13.4490 3.4680 -39.8980

O -7.9060 3.0110 -37.4690

C -6.3440 2.7900 -32.7240

C -7.5190 2.9770 -31.9940

C -5.1350 2.5850 -32.0470

C -7.4830 2.9840 -30.6160

H -8.4530 3.1240 -32.5180

C -5.1040 2.5860 -30.6740

H -4.2350 2.4320 -32.6240

C -6.2760 2.7980 -29.9470

H -8.3830 3.1400 -30.0410

H -4.1830 2.4350 -30.1330

C -4.1320 3.1460 -43.2000

C -5.3650 3.2820 -43.8490

C -2.9590 3.0910 -43.9560

C -5.4230 3.3510 -45.2220

H -6.2670 3.3250 -43.2560

C -3.0230 3.1590 -45.3320

H -2.0090 2.9920 -43.4510

C -4.2520 3.2850 -45.9730

H -6.3630 3.4500 -45.7420

H -2.1250 3.1140 -45.9270

C -1.5620 2.8070 -37.3340

H -1.8370 3.6190 -36.6460

H -1.6420 1.8680 -36.7690

C -8.7760 3.0690 -38.5800

H -8.5340 3.9490 -39.1940

H -8.6490 2.1650 -39.1920

N -3.6210 3.1400 -50.6900

N -5.1950 3.0490 -66.5790

H -3.3650 3.1540 -49.0730

C 0.1480 2.0750 -59.8950

C 0.0190 1.5670 -61.1740

H -0.9600 1.5090 -61.6180

C 1.1500 1.1480 -61.8650

C 2.4270 1.2460 -61.2780

C 2.5420 1.7380 -59.9630

C 1.3920 2.1410 -59.2920

H 1.4480 2.5010 -58.2790

C -0.1230 0.1250 -63.5930

H -0.7760 0.9690 -63.8620

H -0.6220 -0.4670 -62.8130

C 0.1050 -0.7410 -64.8290

H 0.6090 -0.1430 -65.5890

H -0.8810 -1.0110 -65.2140

C 0.9270 -2.0050 -64.5520

H 0.6640 -2.4100 -63.5720

H 0.6690 -2.7570 -65.3030

C 2.4320 -1.7540 -64.6130

H 2.7050 -0.9880 -63.8870

H 2.6770 -1.3710 -65.6080

C 3.2480 -3.0210 -64.3580

H 3.1350 -3.3320 -63.3170

H 2.8710 -3.8370 -64.9780

C 4.7240 -2.7960 -64.6720

H 5.0850 -1.9450 -64.0930

H 4.8300 -2.5410 -65.7300

C 5.5900 -4.0140 -64.3640

H 5.4990 -4.2600 -63.3040

H 5.2230 -4.8700 -64.9330

C 7.0570 -3.7590 -64.7100

H 7.4060 -2.8770 -64.1700

H 7.1400 -3.5410 -65.7780

C 7.9570 -4.9440 -64.3700

H 7.8810 -5.1610 -63.3020

H 7.6100 -5.8250 -64.9140

C 9.4190 -4.6800 -64.7280

H 9.5010 -4.4960 -65.8010

H 9.7620 -3.7790 -64.2140

C 10.3210 -5.8520 -64.3470

H 9.9710 -6.7520 -64.8580

H 10.2430 -6.0320 -63.2730

C 11.7790 -5.6030 -64.7140

H 11.8870 -5.4790 -65.7900

H 12.4000 -6.4400 -64.4010

H 12.1480 -4.7000 -64.2320

C 4.6900 1.4060 -62.0910

H 5.0190 1.7080 -61.0930

H 4.5740 2.3030 -62.7160

C 5.7050 0.4440 -62.6990

H 5.5280 0.3570 -63.7720

H 5.5290 -0.5340 -62.2480

C 7.1490 0.8610 -62.4370

H 7.3540 1.8160 -62.9250

H 7.2920 0.9960 -61.3610

C 8.1310 -0.1940 -62.9370

H 8.0520 -0.2770 -64.0240

H 7.8580 -1.1610 -62.5100

C 9.5760 0.1200 -62.5570

H 9.8630 1.0870 -62.9760

H 9.6530 0.1920 -61.4690

C 10.5360 -0.9560 -63.0550

H 10.4780 -1.0160 -64.1440

H 10.2230 -1.9240 -62.6540

C 11.9810 -0.6910 -62.6400

H 12.3010 0.2780 -63.0300

H 12.0400 -0.6470 -61.5490

C 12.9210 -1.7790 -63.1500

H 12.8770 -1.8130 -64.2410

H 12.5810 -2.7470 -62.7750

C 14.3660 -1.5570 -62.7090

H 14.7120 -0.5860 -63.0730

H 14.4090 -1.5350 -61.6170

C 15.2930 -2.6530 -63.2270

H 15.3000 -2.6350 -64.3190

H 14.9050 -3.6260 -62.9170

C 16.7200 -2.4990 -62.7090

H 17.1130 -1.5260 -63.0120

H 16.7110 -2.5260 -61.6170

C 17.6330 -3.6010 -63.2320

H 17.2670 -4.5770 -62.9210

H 18.6440 -3.4760 -62.8500

H 17.6720 -3.5810 -64.3190

C 3.8730 1.9800 -57.9870

H 3.4590 1.1000 -57.4750

H 3.3120 2.8670 -57.6800

C 5.3280 2.1970 -57.5830

H 5.3580 2.1770 -56.4920

H 5.6370 3.1900 -57.9110

C 6.3120 1.1720 -58.1340

H 6.3420 1.2580 -59.2210

H 5.9790 0.1640 -57.8880

C 7.7100 1.4110 -57.5650

H 7.9520 2.4740 -57.6370

H 7.7170 1.1420 -56.5060

C 8.7800 0.6100 -58.2990

H 8.5150 -0.4480 -58.2860

H 8.8130 0.9340 -59.3420

C 10.1630 0.7900 -57.6780

H 10.4040 1.8550 -57.6300

H 10.1500 0.4060 -56.6550

C 11.2400 0.0630 -58.4770

H 11.2820 0.4830 -59.4850

H 10.9690 -0.9920 -58.5660

C 12.6180 0.1720 -57.8330

H 12.8900 1.2260 -57.7340

H 12.5800 -0.2570 -56.8290

C 13.6850 -0.5540 -58.6480

H 13.4080 -1.6070 -58.7400

H 13.7180 -0.1280 -59.6540

C 15.0700 -0.4530 -58.0160

H 15.0320 -0.8560 -57.0010

H 15.3620 0.5970 -57.9460

C 16.1210 -1.2110 -58.8210

H 15.8210 -2.2590 -58.9050

H 16.1680 -0.7950 -59.8300

C 17.5010 -1.1310 -58.1820

H 17.8280 -0.0960 -58.1080

H 18.2300 -1.6790 -58.7740

H 17.4840 -1.5570 -57.1810

O 3.4510 0.7180 -62.0150

O 1.1160 0.6040 -63.1080

O 3.7810 1.7770 -59.3900

C -0.1300 2.9950 -37.7590

C 0.1990 3.4470 -39.0250

C 1.5370 3.6430 -39.3630

C 2.5350 3.4260 -38.4040

C 2.1940 2.9490 -37.1270

C 0.8570 2.7280 -36.8210

H 0.5700 2.3620 -35.8490

C 1.0100 4.3900 -41.5490

H 0.3550 5.1590 -41.1210

H 0.3970 3.5160 -41.8150

C 1.6880 4.9610 -42.7900

H 2.4900 5.6230 -42.4640

H 0.9450 5.5680 -43.3110

C 2.2370 3.9140 -43.7570

H 1.4760 3.1510 -43.9420

H 2.4380 4.4120 -44.7090

C 3.5270 3.2500 -43.2820

H 3.3390 2.6720 -42.3770

H 4.2490 4.0290 -43.0290

C 4.1100 2.3580 -44.3780

H 3.4870 1.4690 -44.5010

H 4.0840 2.9090 -45.3220

C 5.5550 1.9380 -44.1130

H 5.6030 1.2920 -43.2340

H 6.1480 2.8310 -43.9010

C 6.1530 1.2220 -45.3240

H 5.6510 0.2630 -45.4700

H 5.9660 1.8290 -46.2130

C 7.6590 1.0000 -45.1970

H 7.8640 0.2970 -44.3870

H 8.1370 1.9480 -44.9390

C 8.2670 0.4790 -46.4990

H 7.8510 -0.5050 -46.7270

H 7.9880 1.1530 -47.3130

C 9.7900 0.3890 -46.4330

H 10.1930 1.3610 -46.1380

H 10.0800 -0.3340 -45.6680

C 10.4000 -0.0180 -47.7710

H 10.0830 0.6890 -48.5420

H 10.0220 -1.0040 -48.0490

C 11.9220 -0.0560 -47.7180

H 12.3190 0.9250 -47.4630

H 12.3350 -0.3510 -48.6800

H 12.2600 -0.7670 -46.9670

C 4.6690 2.5760 -38.9390

H 4.3070 1.9970 -39.8000

H 4.6370 1.9350 -38.0510

C 6.0860 3.0790 -39.1740

H 6.3090 3.8000 -38.3860

H 6.1210 3.6010 -40.1320

C 7.1290 1.9670 -39.1410

H 7.0240 1.4060 -38.2100

H 6.9630 1.2740 -39.9680

C 8.5400 2.5430 -39.2220

H 8.6660 3.2800 -38.4240

H 8.6650 3.0610 -40.1760

C 9.6240 1.4790 -39.0790

H 9.4810 0.9470 -38.1360

H 9.5320 0.7510 -39.8870

C 11.0210 2.0930 -39.1020

H 11.1030 2.8270 -38.2960

H 11.1670 2.6210 -40.0480

C 12.1180 1.0460 -38.9360

H 11.9690 0.5180 -37.9910

H 12.0440 0.3120 -39.7410

C 13.5120 1.6690 -38.9480

H 13.5880 2.3970 -38.1360

H 13.6600 2.2050 -39.8880

C 14.6100 0.6210 -38.7860

H 14.4570 0.0840 -37.8470

H 14.5370 -0.1040 -39.6000

C 16.0050 1.2410 -38.7880

H 16.0760 1.9720 -37.9790

H 16.1620 1.7740 -39.7290

C 17.1020 0.1940 -38.6140

H 16.9420 -0.3430 -37.6760

H 17.0380 -0.5330 -39.4270

C 18.4890 0.8230 -38.6040

H 18.6730 1.3590 -39.5330

H 19.2560 0.0600 -38.4910

H 18.5830 1.5260 -37.7790

C 2.9750 2.0330 -35.0600

H 2.6350 1.0150 -35.2910

H 2.2020 2.5380 -34.4690

C 4.2670 2.0050 -34.2530

H 4.0880 1.4190 -33.3500

H 4.4890 3.0260 -33.9480

C 5.4510 1.4380 -35.0310

H 5.5870 2.0270 -35.9400

H 5.2380 0.4090 -35.3260

C 6.7390 1.4800 -34.2130

H 6.8800 2.4890 -33.8200

H 6.6500 0.8060 -33.3590

C 7.9640 1.0940 -35.0390

H 7.8330 0.0840 -35.4330

H 8.0550 1.7740 -35.8900

C 9.2440 1.1510 -34.2110

H 9.3470 2.1470 -33.7740

H 9.1690 0.4390 -33.3870

C 10.4910 0.8310 -35.0320

H 10.5830 1.5490 -35.8510

H 10.3890 -0.1640 -35.4710

C 11.7520 0.8760 -34.1730

H 11.8450 1.8660 -33.7200

H 11.6530 0.1530 -33.3610

C 13.0180 0.5620 -34.9630

H 12.9290 -0.4330 -35.4060

H 13.1270 1.2810 -35.7790

C 14.2620 0.6110 -34.0790

H 14.1510 -0.1050 -33.2610

H 14.3480 1.6040 -33.6320

C 15.5370 0.2920 -34.8550

H 15.4530 -0.7070 -35.2890

H 15.6450 1.0030 -35.6770

C 16.7730 0.3550 -33.9680

H 16.8780 1.3450 -33.5280

H 17.6710 0.1360 -34.5430

H 16.7000 -0.3690 -33.1590

O 3.8390 3.7140 -38.7400

O 1.9770 4.0360 -40.5850

O 3.2200 2.7360 -36.2680

H -7.1730 3.3300 -26.8880

H -2.5110 5.8350 -39.6330

H -5.5160 6.3820 -39.6160

H -3.4980 5.5840 -33.1190

H -6.4850 6.2100 -33.0160

H -2.8050 2.7700 -55.3170

H -5.6810 3.8080 -55.3730

H -3.3850 2.9820 -61.8160

H -6.3970 3.4880 -61.9890

H -0.5960 3.6050 -39.7340

1_2_propagated

9280

mixteNew3_XTB_HT-8chains_xtbopt.xyz 0.000000

C -0.0723 10.4281 7.0924

C 0.6515 10.0370 6.0313

C 0.3938 10.3464 4.6444

C 1.3509 9.9848 3.6710

C -0.7641 11.0020 4.2108

H -1.4860 11.2916 4.9554

C 0.4909 10.5776 -0.4676

C -0.3101 11.0688 0.4919

C -0.0810 10.8635 1.9043

C -1.0202 11.2660 2.8763

C 1.0955 10.2691 2.3468

H 1.8319 10.0449 1.6012

O -2.1197 11.9084 2.4016

C 4.6220 8.3010 3.5047

C 4.7097 7.7614 4.7729

H 3.9274 7.9744 5.4802

C 5.8120 6.9806 5.1125

C 6.8255 6.7480 4.1711

C 6.7048 7.2662 2.8685

C 5.6033 8.0495 2.5559

H 5.4971 8.4782 1.5731

C 5.0566 6.6551 7.3531

H 4.0338 6.4416 7.0107

H 5.1157 7.7150 7.6410

C 5.3555 5.7632 8.5533

H 5.2586 4.7261 8.2347

H 4.5822 5.9622 9.2984

C 6.7366 5.9726 9.1770

H 6.9392 7.0415 9.2672

H 6.7209 5.5540 10.1879

C 7.8544 5.2947 8.3871

H 7.9554 5.7686 7.4122

H 7.5799 4.2524 8.2113

C 9.1891 5.3418 9.1274

H 9.4535 6.3797 9.3422

H 9.0912 4.8242 10.0857

C 10.3064 4.6921 8.3139

H 10.4512 5.2596 7.3934

H 10.0022 3.6821 8.0315

C 11.6281 4.6237 9.0755

H 11.9298 5.6320 9.3709

H 11.4912 4.0420 9.9908

C 12.7328 3.9907 8.2332

H 12.8903 4.5940 7.3363

H 12.4120 2.9977 7.9070

C 14.0511 3.8687 8.9920

H 14.3767 4.8607 9.3152

H 13.8979 3.2665 9.8920

C 15.1445 3.2315 8.1377

H 14.8095 2.2482 7.7976

H 15.3096 3.8451 7.2489

C 16.4609 3.0801 8.8950

H 16.2967 2.4704 9.7868

H 16.8009 4.0634 9.2291

C 17.5407 2.4380 8.0316

H 17.2225 1.4559 7.6885

H 18.4647 2.3242 8.5930

H 17.7430 3.0519 7.1568

C 9.1578 6.6278 4.5940

H 9.2639 7.0995 5.5823

H 9.2261 7.4125 3.8347

C 10.2370 5.5739 4.3885

H 10.1103 5.1452 3.3959

H 10.0763 4.7767 5.1166

C 11.6592 6.1080 4.5231

H 11.8112 6.9245 3.8134

H 11.8146 6.5102 5.5274

C 12.6646 4.9904 4.2526

H 12.5140 4.6129 3.2397

H 12.4671 4.1644 4.9402

C 14.1212 5.4182 4.4107

H 14.3391 6.2434 3.7271

H 14.2892 5.7823 5.4274

C 15.0634 4.2511 4.1243

H 14.9056 3.9043 3.0999

H 14.8136 3.4215 4.7907

C 16.5344 4.6093 4.3113

H 16.7951 5.4414 3.6520

H 16.7015 4.9436 5.3386

C 17.4410 3.4180 4.0116

H 17.2750 3.0886 2.9824

H 17.1716 2.5880 4.6690

C 18.9199 3.7445 4.2012

H 19.1887 4.5847 3.5549

H 19.0959 4.0602 5.2322

C 19.8123 2.5497 3.8784

H 19.6045 2.2084 2.8619

H 19.5725 1.7277 4.5579

C 21.2957 2.8832 4.0020

H 21.5394 3.7043 3.3225

H 21.5055 3.2240 5.0195

C 22.1754 1.6822 3.6771

H 21.9575 0.8572 4.3517

H 23.2273 1.9387 3.7781

H 22.0005 1.3455 2.6578

C 7.5850 7.4115 0.6565

H 7.7014 8.5033 0.6509

H 6.6061 7.1538 0.2325

C 8.6760 6.7509 -0.1802

H 8.5820 7.1187 -1.2030

H 8.4899 5.6770 -0.2034

C 10.0730 7.0318 0.3656

H 10.1011 6.7542 1.4206

H 10.2820 8.0994 0.3048

C 11.1458 6.2551 -0.3918

H 10.9115 5.1896 -0.3479

H 11.1386 6.5472 -1.4446

C 12.5365 6.4875 0.1953

H 12.8250 7.5313 0.0522

H 12.5057 6.3037 1.2727

C 13.5870 5.5764 -0.4341

H 13.3012 4.5356 -0.2619

H 13.6105 5.7342 -1.5149

C 14.9800 5.8146 0.1451

H 14.9312 5.7541 1.2342

H 15.3133 6.8228 -0.1146

C 15.9960 4.7957 -0.3650

H 15.6834 3.7962 -0.0522

H 16.0099 4.8090 -1.4579

C 17.4025 5.0695 0.1627

H 17.7463 6.0369 -0.2115

H 17.3712 5.1303 1.2537

C 18.3939 3.9851 -0.2483

H 18.3902 3.8812 -1.3360

H 18.0736 3.0299 0.1761

C 19.8131 4.2917 0.2249

H 20.1532 5.2222 -0.2339

H 19.8036 4.4399 1.3062

C 20.7847 3.1709 -0.1197

H 20.4745 2.2403 0.3511

H 21.7868 3.4098 0.2290

H 20.8249 3.0132 -1.1951

O 7.8940 5.9636 4.5327

O 5.9836 6.3880 6.3220

O 7.6792 6.9372 1.9897

O 2.5013 9.3674 4.0722

C 0.1959 10.1019 8.4770

C -0.6422 10.6095 9.4757

C 1.2578 9.2975 8.9033

C -0.3814 10.3137 10.7997

H -1.4803 11.2380 9.2141

C 1.4228 9.0610 10.2551

H 1.9502 8.8704 8.1956

H -1.0041 10.7044 11.5929

H 2.2411 8.4521 10.6180

C 0.3916 10.7585 -1.8922

C 1.4125 10.2177 -2.6851

C -0.6362 11.4401 -2.5493

C 1.3561 10.3618 -4.0512

H 2.2331 9.6910 -2.2210

C -0.5982 11.5322 -3.9270

H -1.4461 11.8851 -1.9955

H 2.1195 9.9519 -4.7005

H -1.3584 12.0502 -4.4942

C -3.1470 12.2868 3.2921

H -3.4511 11.4237 3.9010

H -2.7908 13.0791 3.9664

C 3.4588 9.1421 3.0568

H 2.9930 8.6086 2.2162

H 3.8269 10.1079 2.6806

C -0.7197 11.9014 -7.5990

C -1.3628 12.6474 -13.1663

C -1.5456 12.7510 -14.3561

C -1.8551 12.8811 -15.7197

C -1.0463 12.2846 -16.7057

C -2.9828 13.6200 -16.0830

H -3.5350 14.1041 -15.2943

O -1.3980 12.4911 -6.7819

O 0.2306 11.0520 -7.2686

C -2.9125 11.9645 -26.4987

C -3.1310 12.9188 -20.9321

C -2.9579 13.0343 -19.7401

C -2.6200 13.0496 -18.3783

C -3.3774 13.7245 -17.4050

C -1.4557 12.3658 -18.0221

H -0.9050 11.8967 -18.8206

O -1.8950 11.4846 -26.9808

O -3.9801 12.2584 -27.1966

H -3.8756 12.0202 -28.1712

O -4.4346 14.4450 -17.8605

C 2.2309 10.6330 -16.7186

C 2.3056 10.2160 -15.3996

H 1.4450 10.3552 -14.7664

C 3.4923 9.6639 -14.9234

C 4.6046 9.5391 -15.7721

C 4.5045 9.9235 -17.1196

C 3.3139 10.4803 -17.5707

H 3.2218 10.8127 -18.5920

C 2.8520 9.5278 -12.6039

H 1.7974 9.4960 -12.9016

H 3.0946 10.5508 -12.2793

C 3.0532 8.5456 -11.4492

H 2.6979 7.5627 -11.7622

H 2.4186 8.8905 -10.6297

C 4.5012 8.4186 -10.9653

H 4.9893 9.3931 -11.0203

H 4.4916 8.1120 -9.9168

C 5.3070 7.3965 -11.7667

H 5.3741 7.7243 -12.8049

H 4.7804 6.4379 -11.7486

C 6.7091 7.2009 -11.1940

H 7.2659 8.1360 -11.2814

H 6.6380 6.9601 -10.1295

C 7.4788 6.0905 -11.9067

H 7.4738 6.2838 -12.9810

H 6.9728 5.1350 -11.7435

C 8.9200 5.9957 -11.4124

H 9.4141 6.9571 -11.5726

H 8.9185 5.8009 -10.3372

C 9.7215 4.9023 -12.1149

H 9.7224 5.0860 -13.1933

H 9.2423 3.9342 -11.9488

C 11.1608 4.8510 -11.6080

H 11.6319 5.8240 -11.7699

H 11.1521 4.6651 -10.5326

C 11.9951 3.7700 -12.2912

H 11.5160 2.7982 -12.1492

H 12.0322 3.9635 -13.3664

C 13.4170 3.7190 -11.7383

H 13.3751 3.5318 -10.6640

H 13.8969 4.6897 -11.8863

C 14.2564 2.6334 -12.4005

H 13.8017 1.6558 -12.2566

H 15.2574 2.6114 -11.9767

H 14.3430 2.8141 -13.4709

C 6.9393 9.7279 -15.3616

H 6.7717 10.7728 -15.0801

H 7.2607 9.7157 -16.4075

C 7.9917 9.0566 -14.4833

H 7.7789 7.9867 -14.4814

H 7.9044 9.4102 -13.4550

C 9.4078 9.2772 -15.0034

H 9.4412 8.9913 -16.0577

H 9.6646 10.3367 -14.9403

C 10.4385 8.4504 -14.2385

H 10.0860 7.4198 -14.1702

H 10.5369 8.8330 -13.2195

C 11.8039 8.4552 -14.9219

H 11.6849 8.0899 -15.9456

H 12.1850 9.4767 -14.9783

C 12.8113 7.5697 -14.1949

H 12.3928 6.5676 -14.0883

H 12.9848 7.9656 -13.1907

C 14.1412 7.4729 -14.9379

H 13.9597 7.0737 -15.9391

H 14.5700 8.4706 -15.0525

C 15.1352 6.5701 -14.2141

H 14.6928 5.5795 -14.0874

H 15.3324 6.9748 -13.2177

C 16.4530 6.4311 -14.9718

H 16.2528 6.0136 -15.9613

H 16.8960 7.4197 -15.1135

C 17.4436 5.5311 -14.2390

H 16.9970 4.5444 -14.0991

H 17.6431 5.9456 -13.2474

C 18.7615 5.3838 -14.9957

H 18.5663 4.9569 -15.9814

H 19.2042 6.3716 -15.1455

C 19.7473 4.4939 -14.2488

H 19.9780 4.9146 -13.2721

H 20.6753 4.3951 -14.8073

H 19.3272 3.5019 -14.1020

C 5.5277 10.0126 -19.2884

H 5.5666 11.1001 -19.4191

H 4.5944 9.6313 -19.7272

C 6.6972 9.3220 -19.9824

H 6.6094 9.4999 -21.0560

H 6.5845 8.2523 -19.8054

C 8.0779 9.7514 -19.4864

H 8.0659 9.7983 -18.3966

H 8.3195 10.7511 -19.8522

C 9.1281 8.7422 -19.9472

H 8.8188 7.7477 -19.6154

H 9.1581 8.7280 -21.0395

C 10.5342 9.0098 -19.4126

H 10.9140 9.9516 -19.8142

H 10.4996 9.1084 -18.3250

C 11.4748 7.8671 -19.7936

H 11.0772 6.9326 -19.3889

H 11.4944 7.7700 -20.8821

C 12.9032 8.0558 -19.2871

H 12.8935 8.1815 -18.2011

H 13.3267 8.9651 -19.7181

C 13.7799 6.8603 -19.6549

H 13.3586 5.9555 -19.2098

H 13.7686 6.7283 -20.7400

C 15.2252 7.0210 -19.1888

H 15.6599 7.9085 -19.6542

H 15.2442 7.1768 -18.1074

C 16.0721 5.7994 -19.5411

H 16.0405 5.6382 -20.6217

H 15.6454 4.9148 -19.0627

C 17.5262 5.9542 -19.1021

H 17.9634 6.8222 -19.5997

H 17.5614 6.1434 -18.0261

C 18.3515 4.7147 -19.4257

H 17.9385 3.8400 -18.9281

H 19.3804 4.8417 -19.0986

H 18.3549 4.5277 -20.4986

O 5.7293 8.9851 -15.2210

O 3.6962 9.1679 -13.6764

O 5.5835 9.6927 -17.9109

O 0.0948 11.6753 -16.2865

C -1.1700 12.4932 -11.7832

C -0.1429 11.6758 -11.3062

C -2.0286 13.1316 -10.8836

C 0.0052 11.4762 -9.9518

H 0.5260 11.2059 -12.0105

C -1.8793 12.9246 -9.5313

H -2.7980 13.7867 -11.2617

C -0.8680 12.0924 -9.0595

H 0.7920 10.8467 -9.5703

H -2.5378 13.3949 -8.8180

C -3.1416 12.7193 -22.3223

C -1.9792 12.1837 -22.8930

C -4.2404 13.0130 -23.1322

C -1.9182 11.9564 -24.2455

H -1.1408 11.9554 -22.2524

C -4.1749 12.7786 -24.4898

H -5.1381 13.4178 -22.6862

C -3.0156 12.2518 -25.0544

H -1.0352 11.5420 -24.7067

H -5.0192 12.9908 -25.1287

C -5.1851 15.2054 -16.9410

H -5.4413 14.6022 -16.0601

H -4.5958 16.0739 -16.6063

C 0.9725 11.2263 -17.2953

H 0.4672 10.4634 -17.9103

H 1.2387 12.0643 -17.9565

N 0.6310 9.5565 11.1917

N 0.3703 11.0039 -4.6588

H 0.3011 11.0014 -6.2541

C -4.3447 12.7650 2.5108

C -4.4914 12.4552 1.1709

H -3.7000 11.9234 0.6728

C -5.6364 12.8341 0.4881

C -6.6399 13.5796 1.1362

C -6.5047 13.8494 2.5085

C -5.3473 13.4535 3.1710

H -5.2269 13.6598 4.2213

C -4.8888 11.9389 -1.5966

H -4.4439 11.0630 -1.1001

H -4.1003 12.6903 -1.7415

C -5.4529 11.4899 -2.9461

H -6.0564 10.5917 -2.8011

H -4.5978 11.2192 -3.5702

C -6.3011 12.5480 -3.6563

H -5.9179 13.5411 -3.4261

H -6.2174 12.3965 -4.7356

C -7.7725 12.4678 -3.2562

H -7.8514 12.6182 -2.1809

H -8.1491 11.4681 -3.4935

C -8.6285 13.5063 -3.9768

H -8.3679 14.5007 -3.6096

H -8.4162 13.4804 -5.0490

C -10.1167 13.2529 -3.7532

H -10.3028 13.1554 -2.6817

H -10.3954 12.3044 -4.2197

C -10.9973 14.3678 -4.3117

H -10.7084 15.3161 -3.8532

H -10.8352 14.4598 -5.3882

C -12.4780 14.1068 -4.0412

H -12.6229 13.9532 -2.9685

H -12.7808 13.1872 -4.5463

C -13.3684 15.2575 -4.5031

H -13.0375 16.1832 -4.0279

H -13.2640 15.3878 -5.5832

C -14.8360 15.0118 -4.1624

H -15.1662 14.0849 -4.6377

H -14.9380 14.8788 -3.0824

C -15.7351 16.1603 -4.6144

H -15.6442 16.2857 -5.6946

H -15.3960 17.0862 -4.1462

C -17.1958 15.9137 -4.2547

H -17.5623 15.0152 -4.7446

H -17.8136 16.7518 -4.5670

H -17.3075 15.7872 -3.1796

C -8.5069 15.0188 0.7334

H -7.8885 15.8271 1.1312

H -9.2043 14.6793 1.5087

C -9.2909 15.5336 -0.4689

H -9.7335 14.6877 -0.9961

H -8.6045 16.0337 -1.1518

C -10.3885 16.4818 0.0100

H -10.9291 16.0016 0.8303

H -9.9320 17.3922 0.4071

C -11.4005 16.8536 -1.0707

H -11.8145 15.9409 -1.5039

H -10.9056 17.4051 -1.8720

C -12.5350 17.6928 -0.4855

H -12.9534 17.1685 0.3783

H -12.1325 18.6444 -0.1288

C -13.6558 17.9607 -1.4849

H -14.0638 17.0072 -1.8306

H -13.2524 18.4826 -2.3567

C -14.7774 18.7933 -0.8677

H -15.1408 18.2923 0.0336

H -14.3794 19.7653 -0.5677

C -15.9435 18.9995 -1.8310

H -16.3636 18.0292 -2.1020

H -15.5773 19.4692 -2.7477

C -17.0420 19.8689 -1.2253

H -17.3830 19.4186 -0.2903

H -16.6327 20.8546 -0.9881

C -18.2309 20.0308 -2.1693

H -18.6621 19.0478 -2.3785

H -17.8849 20.4462 -3.1189

C -19.3095 20.9411 -1.5859

H -19.6333 20.5459 -0.6201

H -18.8866 21.9327 -1.4087

C -20.5133 21.0620 -2.5116

H -20.2152 21.4746 -3.4732

H -21.2670 21.7144 -2.0767

H -20.9633 20.0860 -2.6837

C -7.4067 14.8526 4.5026

H -6.6351 15.6300 4.6001

H -7.1246 13.9869 5.1161

C -8.7587 15.3655 4.9948

H -8.6648 15.5944 6.0574

H -9.4714 14.5507 4.8838

C -9.2917 16.5868 4.2463

H -9.1915 16.4179 3.1738

H -8.7081 17.4712 4.5052

C -10.7636 16.8171 4.5861

H -11.3144 15.8975 4.3727

H -10.8621 17.0144 5.6560

C -11.4116 17.9573 3.8022

H -10.9986 18.9141 4.1270

H -11.1907 17.8418 2.7386

C -12.9263 17.9520 4.0054

H -13.3259 17.0041 3.6318

H -13.1432 18.0016 5.0751

C -13.6484 19.0953 3.2976

H -13.3872 19.0935 2.2377

H -13.3243 20.0491 3.7192

C -15.1632 18.9603 3.4479

H -15.4874 18.0278 2.9765

H -15.4112 18.8961 4.5107

C -15.9322 20.1256 2.8313

H -15.6397 21.0540 3.3275

H -15.6695 20.2170 1.7758

C -17.4417 19.9352 2.9627

H -17.6992 19.8097 4.0187

H -17.7363 19.0201 2.4417

C -18.2294 21.1125 2.3933

H -17.9555 22.0230 2.9322

H -17.9576 21.2551 1.3456

C -19.7332 20.8944 2.5013

H -20.0279 19.9875 1.9764

H -20.2745 21.7315 2.0671

H -20.0325 20.7965 3.5427

O -7.6836 13.9361 0.3402

O -5.9133 12.4948 -0.8004

O -7.5433 14.4728 3.1487

C -6.4797 15.6508 -17.5669

C -6.9482 15.0704 -18.7302

H -6.3256 14.3556 -19.2362

C -8.2085 15.3943 -19.2069

C -9.0244 16.3104 -18.5101

C -8.5197 16.9223 -17.3502

C -7.2454 16.5924 -16.9057

H -6.8514 17.0393 -16.0074

C -7.9162 14.1651 -21.2181

H -7.3972 13.3372 -20.7087

H -7.1593 14.8679 -21.5918

C -8.7157 13.5843 -22.3867

H -9.2945 12.7274 -22.0321

H -7.9832 13.2136 -23.1075

C -9.6657 14.5726 -23.0702

H -9.2406 15.5754 -23.0508

H -9.7814 14.2778 -24.1158

C -11.0386 14.5801 -22.4026

H -10.9311 14.9534 -21.3858

H -11.4072 13.5505 -22.3523

C -12.0666 15.4224 -23.1523

H -11.7905 16.4763 -23.0853

H -12.0663 15.1486 -24.2105

C -13.4672 15.2098 -22.5800

H -13.4509 15.4281 -21.5106

H -13.7430 14.1576 -22.6950

C -14.5271 16.0770 -23.2523

H -14.2745 17.1312 -23.1103

H -14.5273 15.8837 -24.3273

C -15.9218 15.8076 -22.6909

H -15.9108 15.9643 -21.6094

H -16.1866 14.7630 -22.8688

C -16.9822 16.7113 -23.3167

H -16.7203 17.7564 -23.1308

H -16.9878 16.5646 -24.4000

C -18.3779 16.4330 -22.7644

H -18.6518 15.3983 -22.9781

H -18.3682 16.5540 -21.6784

C -19.4281 17.3667 -23.3637

H -19.4340 17.2530 -24.4496

H -19.1549 18.4025 -23.1450

C -20.8230 17.0832 -22.8195

H -21.1335 16.0722 -23.0729

H -21.5467 17.7794 -23.2380

H -20.8394 17.1806 -21.7359

C -11.0528 17.6006 -18.7572

H -10.5260 18.4984 -19.1013

H -11.2456 17.6784 -17.6831

C -12.3782 17.4454 -19.4921

H -12.8114 16.4881 -19.1988

H -12.1948 17.4191 -20.5654

C -13.3513 18.5684 -19.1414

H -13.3865 18.6909 -18.0555

H -12.9988 19.5079 -19.5721

C -14.7562 18.2622 -19.6507

H -15.1029 17.3342 -19.1888

H -14.7231 18.1010 -20.7301

C -15.7537 19.3741 -19.3362

H -15.7257 19.5979 -18.2667

H -15.4673 20.2825 -19.8724

C -17.1712 18.9745 -19.7328

H -17.4676 18.0966 -19.1522

H -17.1819 18.6888 -20.7872

C -18.1865 20.0900 -19.5063

H -18.1430 20.4185 -18.4653

H -17.9300 20.9473 -20.1343

C -19.6032 19.6285 -19.8359

H -19.8710 18.8011 -19.1734

H -19.6279 19.2490 -20.8596

C -20.6355 20.7421 -19.6904

H -20.5900 21.1487 -18.6782

H -20.3946 21.5516 -20.3832

C -22.0486 20.2390 -19.9726

H -22.2963 19.4482 -19.2595

H -22.0831 19.8008 -20.9732

C -23.0947 21.3472 -19.8742

H -23.0565 21.7969 -18.8794

H -22.8593 22.1298 -20.5994

C -24.5012 20.8196 -20.1327

H -24.5672 20.3785 -21.1255

H -25.2302 21.6228 -20.0646

H -24.7618 20.0563 -19.4023

C -8.8000 18.5378 -15.5813

H -8.0090 19.2237 -15.9172

H -8.3813 17.8525 -14.8340

C -9.9523 19.3147 -14.9460

H -9.5740 19.8386 -14.0666

H -10.6977 18.5913 -14.6116

C -10.6081 20.2982 -15.9142

H -10.7645 19.7874 -16.8641

H -9.9417 21.1458 -16.0888

C -11.9581 20.7929 -15.4013

H -12.5771 19.9277 -15.1529

H -11.8207 21.3713 -14.4854

C -12.6947 21.6362 -16.4417

H -12.1616 22.5760 -16.6001

H -12.7057 21.0972 -17.3918

C -14.1339 21.9253 -16.0220

H -14.6424 20.9762 -15.8297

H -14.1385 22.4941 -15.0894

C -14.9109 22.6893 -17.0916

H -14.8385 22.1510 -18.0386

H -14.4625 23.6746 -17.2354

C -16.3834 22.8472 -16.7189

H -16.8196 21.8569 -16.5691

H -16.4631 23.3894 -15.7728

C -17.1800 23.5810 -17.7940

H -16.7657 24.5826 -17.9326

H -17.0787 23.0471 -18.7409

C -18.6589 23.6903 -17.4340

H -18.7630 24.2249 -16.4871

H -19.0685 22.6865 -17.2934

C -19.4650 24.4099 -18.5113

H -19.0549 25.4116 -18.6610

H -19.3658 23.8672 -19.4544

C -20.9395 24.5159 -18.1424

H -21.3706 23.5257 -18.0116

H -21.4956 25.0298 -18.9225

H -21.0620 25.0685 -17.2122

O -10.2714 16.4530 -19.0364

O -8.7664 14.8436 -20.3188

O -9.3178 17.8181 -16.6827

C -1.5201 8.9277 -15.2681

C -2.4897 9.3585 -16.0909

C -2.4446 9.2689 -17.5323

C -3.4939 9.7880 -18.3219

C -1.3817 8.6482 -18.1844

H -0.6130 8.2260 -17.5638

C -3.2315 9.2357 -22.6733

C -2.2668 8.9305 -21.7896

C -2.3416 9.0642 -20.3516

C -1.2892 8.5495 -19.5600

C -3.4222 9.6665 -19.6998

H -4.2206 10.0544 -20.3070

O -0.2174 7.9663 -20.1855

C -6.5423 11.5838 -17.3655

C -6.1036 11.9210 -16.0988

H -5.1098 11.6362 -15.7969

C -6.9345 12.6337 -15.2471

C -8.2006 13.0713 -15.6751

C -8.6578 12.6997 -16.9523

C -7.8206 11.9355 -17.7628

H -8.1532 11.6263 -18.7389

C -5.5527 12.2398 -13.3522

H -4.5811 12.4624 -13.8218

H -5.7527 11.1645 -13.4674

C -5.4589 12.5939 -11.8706

H -5.3299 13.6731 -11.7804

H -4.5591 12.1072 -11.4878

C -6.6610 12.1584 -11.0314

H -6.9987 11.1724 -11.3585

H -6.3363 12.0655 -9.9923

C -7.8234 13.1466 -11.0950

H -8.1855 13.2204 -12.1204

H -7.4605 14.1354 -10.8048

C -8.9579 12.7439 -10.1552

H -9.3507 11.7675 -10.4522

H -8.5658 12.6447 -9.1401

C -10.0904 13.7663 -10.1572

H -10.4833 13.8575 -11.1734

H -9.6961 14.7445 -9.8722

C -11.2266 13.3864 -9.2111

H -11.5706 12.3770 -9.4485

H -10.8577 13.3716 -8.1828

C -12.4036 14.3522 -9.3211

H -12.6915 14.4405 -10.3722

H -12.0964 15.3441 -8.9809

C -13.6181 13.8913 -8.5202

H -13.8826 12.8753 -8.8237

H -13.3692 13.8593 -7.4573

C -14.8191 14.8089 -8.7409

H -14.5918 15.8058 -8.3558

H -15.0024 14.9047 -9.8138

C -16.0805 14.2769 -8.0682

H -15.9048 14.1881 -6.9942

H -16.2925 13.2795 -8.4564

C -17.2845 15.1781 -8.3132

H -17.1055 16.1750 -7.9171

H -18.1717 14.7739 -7.8292

H -17.4852 15.2656 -9.3793

C -10.2523 13.8915 -14.6601

H -10.4445 13.8375 -13.5800

H -10.7036 13.0212 -15.1386

C -10.8634 15.1847 -15.1996

H -10.8425 15.1624 -16.2890

H -10.2460 16.0199 -14.8684

C -12.2906 15.3543 -14.6806

H -12.8465 14.4302 -14.8540

H -12.2612 15.5150 -13.6001

C -13.0425 16.5101 -15.3353

H -13.1219 16.3293 -16.4092

H -12.4837 17.4382 -15.2018

C -14.4396 16.6631 -14.7356

H -14.9532 15.6992 -14.7795

H -14.3501 16.9342 -13.6803

C -15.2922 17.7069 -15.4511

H -15.3801 17.4426 -16.5082

H -14.8025 18.6825 -15.3960

C -16.6869 17.8005 -14.8352

H -17.1446 16.8073 -14.8355

H -16.5986 18.1184 -13.7932

C -17.5997 18.7686 -15.5819

H -17.6800 18.4582 -16.6257

H -17.1577 19.7685 -15.5675

C -18.9934 18.8229 -14.9616

H -19.4212 17.8172 -14.9481

H -18.9134 19.1582 -13.9244

C -19.9308 19.7570 -15.7209

H -20.0166 19.4189 -16.7561

H -19.5056 20.7643 -15.7354

C -21.3220 19.8085 -15.0926

H -21.7423 18.7998 -15.0671

H -21.2378 20.1572 -14.0612

C -22.2609 20.7276 -15.8631

H -21.8625 21.7396 -15.8965

H -23.2396 20.7606 -15.3906

H -22.3866 20.3731 -16.8836

C -10.4340 12.6328 -18.5555

H -10.6877 11.5719 -18.4405

H -9.7047 12.7474 -19.3630

C -11.6643 13.4377 -18.9539

H -11.9827 13.0459 -19.9232

H -11.3599 14.4746 -19.0941

C -12.8360 13.3588 -17.9793

H -12.6526 14.0189 -17.1342

H -12.9161 12.3420 -17.5965

C -14.1338 13.7609 -18.6829

H -13.9361 14.6261 -19.3208

H -14.4498 12.9475 -19.3404

C -15.2800 14.1143 -17.7378

H -15.4706 13.2827 -17.0564

H -14.9985 14.9778 -17.1336

C -16.5504 14.4381 -18.5225

H -16.3228 15.2094 -19.2639

H -16.8685 13.5496 -19.0732

C -17.7036 14.9227 -17.6465

H -17.4022 15.8278 -17.1147

H -17.9388 14.1623 -16.8980

C -18.9469 15.2141 -18.4847

H -18.6993 15.9614 -19.2434

H -19.2424 14.3035 -19.0127

C -20.1271 15.7151 -17.6565

H -20.3769 14.9714 -16.8948

H -19.8467 16.6357 -17.1394

C -21.3537 15.9734 -18.5301

H -21.6203 15.0548 -19.0562

H -21.1059 16.7216 -19.2878

C -22.5592 16.4550 -17.7273

H -22.8007 15.7154 -16.9605

H -22.3092 17.3902 -17.2200

C -23.7789 16.6686 -18.6146

H -23.5713 17.4081 -19.3854

H -24.6266 17.0142 -18.0253

H -24.0600 15.7407 -19.1078

O -8.8393 13.8744 -14.7757

O -6.5845 12.9825 -13.9703

O -9.8923 13.1188 -17.3355

O -4.5045 10.3774 -17.6412

C -1.5367 8.8995 -13.8254

C -0.5406 8.1577 -13.1772

C -2.4850 9.5380 -13.0199

C -0.5408 8.0865 -11.8021

H 0.2050 7.6286 -13.7523

C -2.3883 9.4120 -11.6475

H -3.2703 10.1309 -13.4570

H 0.2004 7.5148 -11.2627

H -3.0885 9.8888 -10.9761

C -3.1574 9.0600 -24.1090

C -4.2964 9.3011 -24.8868

C -2.0086 8.6547 -24.7948

C -4.2399 9.1152 -26.2546

H -5.2145 9.6240 -24.4180

C -2.0605 8.5059 -26.1682

H -1.0886 8.4591 -24.2686

H -5.1070 9.2832 -26.8792

H -1.1899 8.1916 -26.7290

C 0.8006 7.4835 -19.3344

H 1.1361 8.2838 -18.6600

H 0.3945 6.6629 -18.7170

C -5.6279 10.8736 -18.3332

H -5.3059 11.5881 -19.1082

H -6.1617 10.0456 -18.8221

C -1.8506 7.2488 11.4115

C -1.2847 7.5421 5.7849

C -1.2099 7.6094 4.5808

C -1.1105 7.6482 3.1804

C -2.1744 8.1622 2.4137

C 0.0321 7.1469 2.5521

H 0.8112 6.7468 3.1831

O -1.0887 6.5765 12.0914

O -2.8660 7.9151 11.9073

C -1.8351 8.9054 -7.7120

C -1.0028 7.9568 -2.1845

C -0.8474 7.8073 -0.9944

C -0.8778 7.7172 0.4093

C 0.1654 7.1680 1.1754

C -2.0295 8.1887 1.0381

H -2.8014 8.5679 0.3888

O -2.7449 9.5842 -8.1349

O -0.9470 8.3209 -8.4947

O 1.2421 6.7102 0.4768

C -5.5286 9.5393 3.0265

C -5.4928 9.8104 4.3807

H -4.6357 9.4883 4.9489

C -6.5541 10.4904 4.9647

C -7.6794 10.8667 4.2069

C -7.6932 10.6103 2.8239

C -6.6198 9.9249 2.2709

H -6.6131 9.6998 1.2184

C -5.6159 10.4284 7.1701

H -4.6113 10.5387 6.7398

H -5.7874 9.3620 7.3820

C -5.6687 11.2364 8.4682

H -5.2582 12.2314 8.2825

H -5.0136 10.7329 9.1815

C -7.0732 11.3882 9.0599

H -7.6546 10.4870 8.8608

H -6.9896 11.5000 10.1438

C -7.8022 12.6050 8.4922

H -7.9268 12.4721 7.4179

H -7.1843 13.4919 8.6571

C -9.1670 12.8329 9.1391

H -9.8383 12.0168 8.8651

H -9.0670 12.8317 10.2273

C -9.7675 14.1614 8.6851

H -9.7422 14.2007 7.5936

H -9.1490 14.9834 9.0541

C -11.2071 14.3656 9.1486

H -11.8058 13.5008 8.8530

H -11.2392 14.4317 10.2386

C -11.8134 15.6279 8.5376

H -11.7553 15.5546 7.4485

H -11.2231 16.4968 8.8373

C -13.2688 15.8399 8.9452

H -13.8326 14.9219 8.7588

H -13.3224 16.0450 10.0163

C -13.9128 16.9882 8.1704

H -13.3291 17.8999 8.3207

H -13.8900 16.7533 7.1028

C -15.3569 17.2393 8.5966

H -15.3770 17.5468 9.6452

H -15.9235 16.3081 8.5180

C -16.0231 18.3079 7.7386

H -15.4765 19.2461 7.8042

H -17.0453 18.4839 8.0671

H -16.0464 17.9951 6.6968

C -9.9091 11.7426 4.3384

H -10.2751 10.8600 3.8028

H -9.7844 12.5565 3.6156

C -10.9297 12.1518 5.3985

H -10.4622 12.8411 6.1014

H -11.2543 11.2766 5.9631

C -12.1172 12.8194 4.7090

H -11.7670 13.7325 4.2209

H -12.4884 12.1598 3.9197

C -13.2719 13.1760 5.6421

H -12.8937 13.7300 6.5019

H -13.7416 12.2632 6.0140

C -14.3022 14.0260 4.8995

H -13.8466 14.9866 4.6463

H -14.5624 13.5326 3.9588

C -15.5792 14.2801 5.6929

H -15.3306 14.7287 6.6550

H -16.0824 13.3317 5.8892

C -16.5189 15.2088 4.9246

H -16.0286 16.1758 4.7865

H -16.7013 14.7922 3.9311

C -17.8556 15.4199 5.6283

H -17.6835 15.8233 6.6286

H -18.3603 14.4576 5.7424

C -18.7556 16.3732 4.8454

H -18.2623 17.3439 4.7594

H -18.8959 15.9870 3.8334

C -20.1203 16.5577 5.5036

H -19.9831 16.9352 6.5205

H -20.6203 15.5898 5.5757

C -21.0057 17.5250 4.7220

H -20.5092 18.4956 4.6590

H -21.1319 17.1552 3.7022

C -22.3751 17.6944 5.3680

H -22.8955 16.7400 5.4102

H -22.9837 18.3923 4.7976

H -22.2750 18.0756 6.3819

C -8.8387 10.6986 0.7057

H -9.1347 9.6431 0.6526

H -7.8854 10.8275 0.1857

C -9.8609 11.5954 0.0038

H -10.1347 11.1010 -0.9310

H -9.3662 12.5342 -0.2417

C -11.1118 11.8985 0.8220

H -10.8273 12.4993 1.6854

H -11.5517 10.9725 1.1937

C -12.1436 12.6648 -0.0009

H -11.6646 13.5354 -0.4524

H -12.5071 12.0294 -0.8128

C -13.3245 13.1369 0.8419

H -13.7967 12.2775 1.3236

H -12.9604 13.7970 1.6333

C -14.3612 13.8789 0.0037

H -13.8836 14.7309 -0.4861

H -14.7350 13.2126 -0.7777

C -15.5356 14.3783 0.8409

H -15.1686 15.0638 1.6096

H -16.0016 13.5315 1.3518

C -16.5820 15.0879 -0.0128

H -16.1180 15.9393 -0.5167

H -16.9420 14.4018 -0.7835

C -17.7680 15.5780 0.8132

H -18.2279 14.7266 1.3221

H -17.4114 16.2669 1.5840

C -18.8167 16.2779 -0.0457

H -19.1609 15.5933 -0.8243

H -18.3625 17.1397 -0.5404

C -20.0147 16.7431 0.7770

H -20.4715 15.8789 1.2657

H -19.6716 17.4227 1.5616

C -21.0586 17.4460 -0.0828

H -20.6300 18.3209 -0.5644

H -21.9029 17.7668 0.5231

H -21.4270 16.7763 -0.8563

O -8.6604 11.4718 4.9449

O -6.5979 10.8901 6.2707

O -8.7279 11.0782 2.0696

O -3.2763 8.5967 3.0790

C -1.4138 7.4734 7.1839

C -2.5054 8.0860 7.8068

C -0.4719 6.7919 7.9609

C -2.6491 8.0295 9.1730

H -3.2325 8.6009 7.1976

C -0.6246 6.7282 9.3269

H 0.3705 6.3143 7.4857

C -1.7078 7.3502 9.9450

H -3.4876 8.5017 9.6621

H 0.0903 6.2048 9.9430

C -1.2007 8.1635 -3.5628

C -2.3077 8.9036 -3.9891

C -0.3078 7.6523 -4.5065

C -2.5042 9.1412 -5.3309

H -3.0025 9.2814 -3.2532

C -0.5116 7.8896 -5.8484

H 0.5319 7.0644 -4.1723

C -1.6057 8.6409 -6.2685

H -3.3500 9.7123 -5.6824

H 0.1650 7.4914 -6.5876

C 2.2075 5.9211 1.1322

H 2.6176 6.4405 2.0105

H 1.7525 4.9834 1.4917

C -4.3849 8.9266 2.2696

H -4.0842 9.6684 1.5169

H -4.7342 8.0235 1.7477

N -1.4403 8.7061 -11.0528

N -3.1478 8.7216 -26.8913

H -1.1648 8.5127 -9.4642

C 2.0176 6.9628 -20.0499

C 1.9940 6.4565 -21.3356

H 1.0719 6.4741 -21.8904

C 3.1623 5.9542 -21.8948

C 4.3765 5.9572 -21.1733

C 4.3738 6.4218 -19.8452

C 3.1908 6.9270 -19.3160

H 3.1748 7.2999 -18.3043

C 2.0273 5.0061 -23.7544

H 1.4818 5.8815 -24.1360

H 1.3911 4.4922 -23.0197

C 2.3387 4.0564 -24.9083

H 2.9179 4.5902 -25.6618

H 1.3828 3.7751 -25.3537

C 3.1099 2.8068 -24.4650

H 2.8570 2.5664 -23.4308

H 2.8052 1.9582 -25.0821

C 4.6215 2.9885 -24.5926

H 4.9250 3.8762 -24.0370

H 4.8630 3.1506 -25.6470

C 5.3974 1.7707 -24.0878

H 5.4155 1.7797 -22.9955

H 4.8874 0.8553 -24.3991

C 6.8267 1.7489 -24.6276

H 7.2986 2.7176 -24.4496

H 6.7955 1.5980 -25.7100

C 7.6741 0.6490 -23.9929

H 7.7474 0.8260 -22.9171

H 7.1788 -0.3147 -24.1358

C 9.0785 0.5878 -24.5923

H 9.5616 1.5610 -24.4842

H 9.0038 0.3792 -25.6626

C 9.9410 -0.4833 -23.9292

H 10.0039 -0.2812 -22.8560

H 9.4628 -1.4574 -24.0552

C 11.3511 -0.5379 -24.5124

H 11.2926 -0.7390 -25.5846

H 11.8333 0.4353 -24.3884

C 12.2044 -1.6131 -23.8424

H 11.7352 -2.5879 -23.9945

H 12.2394 -1.4249 -22.7675

C 13.6245 -1.6479 -24.3954

H 13.6155 -1.8701 -25.4598

H 14.2124 -2.4108 -23.8900

H 14.1152 -0.6862 -24.2535

C 6.7301 5.4910 -21.3186

H 6.7482 4.9125 -20.3911

H 7.0162 6.5248 -21.0968

C 7.7334 4.8779 -22.2867

H 7.7930 5.4758 -23.1958

H 7.3900 3.8802 -22.5619

C 9.0947 4.7951 -21.5983

H 9.4620 5.8037 -21.3988

H 8.9633 4.2967 -20.6337

C 10.1414 4.0210 -22.3927

H 10.3692 4.5444 -23.3237

H 9.7403 3.0399 -22.6548

C 11.4161 3.8406 -21.5708

H 11.8383 4.8202 -21.3357

H 11.1586 3.3570 -20.6252

C 12.4659 2.9926 -22.2823

H 12.7806 3.4920 -23.2016

H 12.0209 2.0345 -22.5642

C 13.6805 2.7400 -21.3936

H 14.1311 3.6958 -21.1138

H 13.3529 2.2468 -20.4752

C 14.7263 1.8659 -22.0795

H 15.0870 2.3685 -22.9807

H 14.2603 0.9276 -22.3891

C 15.9078 1.5601 -21.1616

H 16.3714 2.4968 -20.8441

H 15.5420 1.0495 -20.2675

C 16.9540 0.6837 -21.8446

H 17.3410 1.2014 -22.7265

H 16.4811 -0.2400 -22.1889

C 18.1146 0.3375 -20.9143

H 18.5883 1.2601 -20.5700

H 17.7284 -0.1854 -20.0364

C 19.1541 -0.5372 -21.6032

H 18.7072 -1.4725 -21.9327

H 19.9714 -0.7682 -20.9236

H 19.5645 -0.0293 -22.4733

C 5.4753 6.4072 -17.6909

H 5.0184 5.4791 -17.3202

H 4.8814 7.2532 -17.3380

C 6.8832 6.5826 -17.1347

H 6.7831 6.6784 -16.0532

H 7.2827 7.5204 -17.5183

C 7.8370 5.4433 -17.4726

H 7.9221 5.3625 -18.5578

H 7.4307 4.5030 -17.0910

C 9.2283 5.6612 -16.8827

H 9.6114 6.6328 -17.1998

H 9.1635 5.6713 -15.7925

C 10.1896 4.5629 -17.3270

H 9.7867 3.5942 -17.0248

H 10.2589 4.5712 -18.4189

C 11.5932 4.7111 -16.7451

H 12.0062 5.6832 -17.0222

H 11.5388 4.6746 -15.6546

C 12.5089 3.5988 -17.2487

H 12.5811 3.6588 -18.3378

H 12.0633 2.6328 -16.9986

C 13.9117 3.6617 -16.6521

H 14.3680 4.6250 -16.8883

H 13.8427 3.5896 -15.5641

C 14.7927 2.5334 -17.1820

H 14.3078 1.5754 -16.9788

H 14.8885 2.6319 -18.2665

C 16.1834 2.5277 -16.5544

H 16.0927 2.3941 -15.4734

H 16.6631 3.4937 -16.7265

C 17.0592 1.4167 -17.1270

H 16.5720 0.4537 -16.9599

H 17.1553 1.5552 -18.2069

C 18.4455 1.3967 -16.4966

H 18.9587 2.3393 -16.6680

H 19.0469 0.5963 -16.9209

H 18.3725 1.2384 -15.4220

O 5.4307 5.4565 -21.8776

O 3.2342 5.4122 -23.1379

O 5.5324 6.3679 -19.1106

C 3.3489 5.6212 0.1909

C 3.3156 6.0571 -1.1218

C 4.4087 5.8339 -1.9559

C 5.5329 5.1697 -1.4525

C 5.5460 4.6922 -0.1284

C 4.4405 4.9238 0.6838

H 4.4120 4.5596 1.6975

C 3.3068 6.7013 -3.8805

H 2.8633 7.5246 -3.3047

H 2.5778 5.8780 -3.9266

C 3.6097 7.2079 -5.2888

H 4.2594 8.0825 -5.2160

H 2.6580 7.5287 -5.7151

C 4.2713 6.1683 -6.2006

H 3.9765 5.1655 -5.8858

H 3.9169 6.3091 -7.2240

C 5.7944 6.2844 -6.1811

H 6.1390 6.2147 -5.1497

H 6.0772 7.2674 -6.5672

C 6.4767 5.2058 -7.0195

H 6.3000 4.2314 -6.5582

H 6.0389 5.1811 -8.0205

C 7.9812 5.4526 -7.1231

H 8.3590 5.7396 -6.1393

H 8.1703 6.2906 -7.7975

C 8.7522 4.2242 -7.5996

H 8.4871 3.3740 -6.9685

H 8.4642 3.9781 -8.6237

C 10.2623 4.4411 -7.5226

H 10.5195 4.7739 -6.5143

H 10.5543 5.2365 -8.2145

C 11.0510 3.1731 -7.8367

H 10.7276 2.3752 -7.1628

H 10.8355 2.8471 -8.8569

C 12.5541 3.3853 -7.6728

H 12.8870 4.1627 -8.3659

H 12.7571 3.7411 -6.6601

C 13.3511 2.1082 -7.9229

H 13.1330 1.7322 -8.9249

H 13.0354 1.3428 -7.2096

C 14.8507 2.3411 -7.7847

H 15.1910 3.0762 -8.5115

H 15.4009 1.4174 -7.9509

H 15.0886 2.7095 -6.7888

C 6.5765 3.7716 -3.0063

H 5.7162 3.8084 -3.6908

H 6.4468 2.9194 -2.3261

C 7.8709 3.6253 -3.7941

H 8.0588 4.5650 -4.3109

H 7.7381 2.8396 -4.5400

C 9.0576 3.2940 -2.8966

H 9.1156 4.0412 -2.1033

H 8.8939 2.3184 -2.4312

C 10.3692 3.2678 -3.6776

H 10.6012 4.2748 -4.0303

H 10.2532 2.6282 -4.5563

C 11.5261 2.7469 -2.8309

H 11.6325 3.3722 -1.9414

H 11.2940 1.7311 -2.4993

C 12.8490 2.7291 -3.5927

H 13.1343 3.7518 -3.8502

H 12.7224 2.1751 -4.5262

C 13.9604 2.0817 -2.7720

H 14.0748 2.6231 -1.8297

H 13.6730 1.0547 -2.5326

C 15.2990 2.0649 -3.5048

H 15.6151 3.0907 -3.7072

H 15.1797 1.5589 -4.4656

C 16.3775 1.3524 -2.6921

H 16.4801 1.8438 -1.7226

H 16.0633 0.3213 -2.5088

C 17.7298 1.3453 -3.3988

H 18.0550 2.3747 -3.5681

H 17.6259 0.8699 -4.3771

C 18.7919 0.6070 -2.5875

H 18.8881 1.0802 -1.6092

H 18.4695 -0.4239 -2.4272

C 20.1463 0.6085 -3.2850

H 20.0804 0.1114 -4.2496

H 20.8892 0.0909 -2.6818

H 20.4904 1.6271 -3.4514

C 6.8239 3.6113 1.5881

H 6.3723 2.6170 1.6978

H 6.3272 4.3048 2.2739

C 8.3056 3.6148 1.9509

H 8.3750 3.4330 3.0234

H 8.6798 4.6215 1.7633

C 9.1673 2.6023 1.2027

H 9.0989 2.7839 0.1292

H 8.7987 1.5932 1.3953

C 10.6230 2.7072 1.6590

H 11.0151 3.6864 1.3770

H 10.6577 2.6434 2.7492

C 11.5232 1.6214 1.0758

H 11.1577 0.6416 1.3888

H 11.4837 1.6566 -0.0149

C 12.9701 1.7890 1.5370

H 13.3583 2.7396 1.1655

H 12.9997 1.8291 2.6277

C 13.8714 0.6551 1.0530

H 13.8155 0.5862 -0.0352

H 13.5101 -0.2911 1.4616

C 15.3260 0.8595 1.4698

H 15.7030 1.7812 1.0179

H 15.3801 0.9814 2.5538

C 16.2136 -0.3069 1.0464

H 15.8609 -1.2214 1.5284

H 16.1308 -0.4497 -0.0330

C 17.6788 -0.0785 1.4105

H 17.7610 0.1139 2.4826

H 18.0452 0.8077 0.8871

C 18.5548 -1.2729 1.0437

H 18.2036 -2.1557 1.5842

H 18.4538 -1.4794 -0.0236

C 20.0213 -1.0258 1.3741

H 20.4075 -0.1937 0.7878

H 20.6199 -1.9046 1.1496

H 20.1410 -0.7875 2.4279

O 6.6364 4.9775 -2.2562

O 4.4851 6.2383 -3.2530

O 6.6682 4.0428 0.2442

H -2.9816 7.7518 12.8939

H 1.3420 9.9735 -0.1896

H -1.1859 11.6425 0.2314

H 1.5345 9.4344 6.1833

H -0.9442 11.0516 6.9477

H -0.6154 8.5044 -15.6814

H -3.3942 9.7871 -15.6875

H -1.3337 8.5180 -22.1418

H -4.1798 9.6231 -22.3246

H 2.4337 6.5668 -1.4692

C -0.7171 15.8678 -16.4299

C 0.0664 15.3376 -17.3841

C -0.1859 15.3717 -18.8070

C 0.8054 14.9063 -19.6989

C -1.3947 15.8179 -19.3398

H -2.1511 16.1578 -18.6555

C -0.2340 14.7493 -23.9799

C -0.9750 15.2938 -23.0015

C -0.6671 15.3339 -21.5894

C -1.6577 15.8064 -20.6974

C 0.5501 14.9012 -21.0584

H 1.3039 14.5507 -21.7439

C -5.1053 16.9995 -21.1276

C -6.0501 17.7887 -20.4879

H -5.8721 18.1042 -19.4729

C -7.2152 18.1524 -21.1479

C -7.4441 17.7034 -22.4580

C -6.4894 16.8995 -23.0979

C -5.3194 16.5611 -22.4215

H -4.5642 15.9457 -22.8799

C -7.8313 19.7706 -19.5143

H -7.8378 19.1621 -18.6012

H -6.8211 20.1782 -19.6461

C -8.8539 20.9084 -19.4295

H -9.3530 20.9717 -20.3982

H -9.6105 20.6666 -18.6839

C -8.2211 22.2590 -19.0956

H -7.5628 22.1482 -18.2304

H -9.0146 22.9571 -18.8171

C -7.4382 22.8495 -20.2661

H -6.6496 22.1612 -20.5744

H -8.1117 22.9740 -21.1171

C -6.8151 24.1980 -19.9172

H -6.1573 24.0811 -19.0521

H -7.6050 24.8984 -19.6367

C -6.0157 24.7803 -21.0808

H -5.1965 24.1035 -21.3329

H -6.6597 24.8561 -21.9595

C -5.4521 26.1597 -20.7495

H -4.8225 26.0882 -19.8588

H -6.2776 26.8363 -20.5134

C -4.6360 26.7471 -21.8980

H -3.7851 26.0950 -22.1077

H -5.2543 26.7842 -22.7982

C -4.1333 28.1514 -21.5735

H -3.5338 28.1175 -20.6592

H -4.9886 28.8027 -21.3807

C -3.2948 28.7424 -22.7028

H -3.8860 28.7548 -23.6214

H -2.4235 28.1064 -22.8743

C -2.8286 30.1616 -22.3871

H -3.7023 30.7961 -22.2186

H -2.2449 30.1519 -21.4635

C -1.9872 30.7519 -23.5125

H -2.5563 30.7797 -24.4388

H -1.6791 31.7656 -23.2690

H -1.0940 30.1517 -23.6754

C -8.4945 18.9633 -24.1536

H -7.8680 18.5274 -24.9414

H -9.5102 19.0787 -24.5401

C -7.9285 20.3169 -23.7207

H -8.6015 20.7606 -22.9848

H -6.9650 20.1537 -23.2280

C -7.7386 21.2632 -24.9015

H -8.6955 21.4107 -25.4085

H -7.0514 20.8097 -25.6186

C -7.1840 22.6163 -24.4607

H -7.8976 23.0973 -23.7873

H -6.2581 22.4630 -23.8999

C -6.9057 23.5365 -25.6458

H -7.8320 23.7011 -26.2015

H -6.1992 23.0470 -26.3204

C -6.3304 24.8817 -25.2111

H -7.0397 25.3813 -24.5464

H -5.4101 24.7132 -24.6455

C -6.0306 25.7887 -26.4014

H -6.9572 25.9953 -26.9424

H -5.3594 25.2676 -27.0894

C -5.3863 27.1043 -25.9750

H -6.0532 27.6263 -25.2829

H -4.4560 26.8918 -25.4430

C -5.0872 28.0124 -27.1642

H -6.0191 28.2487 -27.6838

H -4.4452 27.4811 -27.8715

C -4.4022 29.3091 -26.7391

H -5.0440 29.8414 -26.0328

H -3.4694 29.0685 -26.2236

C -4.0998 30.2185 -27.9263

H -5.0322 30.4610 -28.4418

H -3.4621 29.6862 -28.6366

C -3.4103 31.5079 -27.4953

H -2.4627 31.2909 -27.0064

H -3.2132 32.1433 -28.3557

H -4.0370 32.0603 -26.7977

C -5.8315 16.0650 -25.2718

H -4.9916 15.5672 -24.7812

H -6.3296 15.3368 -25.9193

C -5.3342 17.2375 -26.1229

H -4.6542 16.8408 -26.8791

H -6.1899 17.6777 -26.6404

C -4.6280 18.3208 -25.3119

H -5.3123 18.7165 -24.5587

H -3.7720 17.8947 -24.7822

C -4.1538 19.4656 -26.2034

H -4.9949 19.8254 -26.8030

H -3.3930 19.0970 -26.8951

C -3.5861 20.6280 -25.3933

H -2.7384 20.2790 -24.7995

H -4.3500 20.9880 -24.6998

C -3.1402 21.7795 -26.2898

H -3.9834 22.1042 -26.9050

H -2.3577 21.4283 -26.9672

C -2.6179 22.9715 -25.4913

H -3.3981 23.3191 -24.8110

H -1.7667 22.6543 -24.8830

C -2.1925 24.1188 -26.4018

H -3.0476 24.4368 -27.0022

H -1.4212 23.7642 -27.0903

C -1.6545 25.3166 -25.6233

H -0.7997 25.0017 -25.0190

H -2.4250 25.6793 -24.9377

C -1.2284 26.4497 -26.5530

H -0.4569 26.0826 -27.2357

H -2.0836 26.7585 -27.1585

C -0.6922 27.6573 -25.7904

H 0.1528 27.3486 -25.1699

H -1.4706 28.0345 -25.1236

C -0.2484 28.7699 -26.7324

H -1.0751 29.0868 -27.3637

H 0.1056 29.6323 -26.1711

H 0.5584 28.4257 -27.3767

O -8.6251 18.0599 -23.0691

O -8.1647 18.9703 -20.6317

O -6.8232 16.4632 -24.3459

O -2.8331 16.2160 -21.2407

C 4.2728 13.8296 -19.1209

C 4.1563 13.5203 -17.7765

H 3.1928 13.5609 -17.3009

C 5.2774 13.1312 -17.0569

C 6.5350 13.0230 -17.6832

C 6.6506 13.3851 -19.0348

C 5.5148 13.7856 -19.7342

H 5.5861 14.0272 -20.7827

C 4.2253 13.2537 -14.9001

H 3.4074 12.5228 -14.9039

H 3.8451 14.2229 -15.2441

C 4.8349 13.4003 -13.5060

H 5.3936 12.4873 -13.2950

H 4.0449 13.5025 -12.7613

C 5.7769 14.6059 -13.4700

H 6.2804 14.6758 -14.4365

H 5.1935 15.5194 -13.3311

C 6.8426 14.5121 -12.3821

H 7.4799 13.6485 -12.5842

H 6.3695 14.3579 -11.4108

C 7.6993 15.7781 -12.3432

H 7.9763 16.0547 -13.3639

H 7.1090 16.5996 -11.9316

C 8.9729 15.6060 -11.5172

H 9.6142 14.8618 -11.9967

H 8.7165 15.2304 -10.5250

C 9.7367 16.9213 -11.3798

H 9.8617 17.3712 -12.3680

H 9.1477 17.6163 -10.7770

C 11.1099 16.7335 -10.7379

H 11.7348 16.1254 -11.3963

H 10.9990 16.1916 -9.7960

C 11.8025 18.0679 -10.4732

H 11.8261 18.6530 -11.3966

H 11.2197 18.6362 -9.7434

C 13.2268 17.8884 -9.9542

H 13.2113 17.2516 -9.0666

H 13.8249 17.3807 -10.7142

C 13.8843 19.2216 -9.6046

H 13.3121 19.7082 -8.8119

H 13.8548 19.8771 -10.4789

C 15.3283 19.0435 -9.1535

H 15.3763 18.4102 -8.2708

H 15.7769 20.0044 -8.9117

H 15.9181 18.5793 -9.9409

C 8.8531 12.8616 -16.9659

H 9.2267 12.8655 -17.9925

H 9.3787 12.0827 -16.4048

C 9.0893 14.2180 -16.2983

H 8.5767 14.2223 -15.3347

H 8.6553 15.0109 -16.9131

C 10.5802 14.4653 -16.0899

H 10.9844 13.6720 -15.4562

H 11.0954 14.4110 -17.0512

C 10.8663 15.8152 -15.4374

H 10.3090 15.8912 -14.5018

H 10.5234 16.6200 -16.0929

C 12.3565 15.9895 -15.1511

H 12.6775 15.2130 -14.4532

H 12.9207 15.8511 -16.0766

C 12.6839 17.3584 -14.5633

H 12.0878 17.5181 -13.6625

H 12.4107 18.1372 -15.2800

C 14.1654 17.4824 -14.2167

H 14.4200 16.7332 -13.4635

H 14.7636 17.2706 -15.1053

C 14.5235 18.8685 -13.6866

H 13.8992 19.0962 -12.8208

H 14.3046 19.6167 -14.4528

C 15.9932 18.9638 -13.2894

H 16.2020 18.2291 -12.5060

H 16.6173 18.7094 -14.1486

C 16.3668 20.3549 -12.7872

H 15.7412 20.6117 -11.9300

H 16.1650 21.0890 -13.5716

C 17.8353 20.4451 -12.3802

H 18.0320 19.7244 -11.5826

H 18.4642 20.1761 -13.2315

C 18.2061 21.8423 -11.9009

H 18.0384 22.5726 -12.6903

H 19.2538 21.8842 -11.6120

H 17.6008 22.1241 -11.0413

C 8.1131 13.8452 -20.8945

H 7.6171 14.8177 -20.9996

H 7.7174 13.1603 -21.6582

C 9.6224 14.0176 -21.0532

H 9.8554 14.1787 -22.1067

H 10.1081 13.0946 -20.7314

C 10.1298 15.1899 -20.2151

H 9.7345 15.0946 -19.2029

H 9.7479 16.1226 -20.6367

C 11.6539 15.2536 -20.1494

H 12.0319 14.3556 -19.6553

H 12.0642 15.2692 -21.1618

C 12.1282 16.4904 -19.3904

H 11.8277 17.3863 -19.9394

H 11.6389 16.5239 -18.4143

C 13.6417 16.5083 -19.1893

H 13.9369 15.6418 -18.5928

H 14.1383 16.4236 -20.1591

C 14.1059 17.7855 -18.4937

H 13.5631 17.9012 -17.5525

H 13.8614 18.6456 -19.1212

C 15.6055 17.7788 -18.2091

H 15.8428 16.9404 -17.5482

H 16.1504 17.6245 -19.1428

C 16.0695 19.0814 -17.5621

H 15.8684 19.9125 -18.2420

H 15.4942 19.2556 -16.6500

C 17.5569 19.0572 -17.2207

H 18.1338 18.8641 -18.1280

H 17.7544 18.2370 -16.5256

C 18.0241 20.3697 -16.5965

H 17.8524 21.1859 -17.3017

H 17.4296 20.5754 -15.7041

C 19.5010 20.3268 -16.2214

H 19.6918 19.5215 -15.5141

H 19.8080 21.2647 -15.7651

H 20.1142 20.1566 -17.1034

O 7.5007 12.4591 -16.9034

O 5.2752 12.8095 -15.7416

O 7.8888 13.3074 -19.6066

O 1.9703 14.4823 -19.1308

C -0.5093 15.7722 -15.0020

C -1.5046 16.2597 -14.1448

C 0.6135 15.1963 -14.3972

C -1.3442 16.1423 -12.7812

H -2.3955 16.7189 -14.5480

C 0.6853 15.1429 -13.0188

H 1.4178 14.7933 -14.9894

H -2.1029 16.4940 -12.0957

H 1.5334 14.7056 -12.5093

C -0.6176 14.6498 -25.3740

C 0.1997 13.9408 -26.2617

C -1.7908 15.1944 -25.9063

C -0.1838 13.8063 -27.5833

H 1.1185 13.4913 -25.9153

C -2.0758 14.9987 -27.2447

H -2.4723 15.7591 -25.2906

H 0.4247 13.2551 -28.2879

H -2.9811 15.3981 -27.6838

C -3.8698 16.6072 -20.3630

H -4.1213 15.7747 -19.6916

H -3.5436 17.4641 -19.7558

C 3.0635 14.1644 -19.9594

H 2.8190 13.2925 -20.5907

H 3.2944 15.0098 -20.6226

C 2.6949 11.9475 9.8475

C 2.6607 12.7951 4.2484

C 2.5883 12.9568 3.0516

C 2.3884 13.1588 1.6753

C 3.3529 12.7808 0.7226

C 1.1808 13.7262 1.2653

H 0.4755 13.9858 2.0384

O 1.8532 12.4643 10.5703

O 3.6388 11.1523 10.2938

C 0.9701 14.6803 -9.2039

C 1.5352 13.9605 -3.5964

C 1.6556 13.7935 -2.4036

C 1.8696 13.5810 -1.0273

C 0.8903 13.9275 -0.0732

C 3.0774 13.0104 -0.6154

H 3.7880 12.7516 -1.3855

O 1.7181 14.1592 -10.0049

O -0.0402 15.4549 -9.5626

C -2.5538 15.1892 -0.2713

C -3.5178 15.8269 0.4951

H -3.3415 15.9746 1.5487

C -4.6930 16.2598 -0.0958

C -4.9364 16.0362 -1.4646

C -3.9542 15.3931 -2.2311

C -2.7724 14.9748 -1.6169

H -2.0015 14.4785 -2.1815

C -5.4457 17.3770 1.8742

H -5.4451 16.5113 2.5524

H -4.4699 17.8760 1.9507

C -6.5604 18.3492 2.2574

H -7.4898 17.9844 1.8189

H -6.6687 18.3409 3.3433

C -6.3099 19.7861 1.7991

H -5.3995 20.1610 2.2747

H -7.1399 20.4034 2.1504

C -6.1848 19.9387 0.2843

H -5.2892 19.4240 -0.0644

H -7.0415 19.4648 -0.1988

C -6.1137 21.4111 -0.1147

H -5.3039 21.8940 0.4385

H -7.0445 21.9074 0.1716

C -5.8800 21.6096 -1.6107

H -4.9246 21.1557 -1.8874

H -6.6656 21.0985 -2.1712

C -5.8650 23.0879 -1.9927

H -5.1162 23.6082 -1.3910

H -6.8369 23.5290 -1.7602

C -5.5547 23.3019 -3.4723

H -4.5655 22.8946 -3.6943

H -6.2818 22.7560 -4.0773

C -5.5862 24.7790 -3.8604

H -4.8830 25.3323 -3.2323

H -6.5838 25.1815 -3.6685

C -5.2281 24.9948 -5.3279

H -5.9154 24.4223 -5.9553

H -4.2195 24.6173 -5.5124

C -5.2917 26.4674 -5.7291

H -6.3029 26.8427 -5.5596

H -4.6149 27.0433 -5.0937

C -4.9121 26.6739 -7.1897

H -5.5784 26.1121 -7.8399

H -4.9754 27.7261 -7.4592

H -3.8940 26.3362 -7.3706

C -6.3960 16.8891 -3.2017

H -6.2315 16.0870 -3.9276

H -7.4579 17.1567 -3.2060

C -5.5594 18.1136 -3.5716

H -5.5720 18.8141 -2.7351

H -4.5259 17.8059 -3.7380

C -6.1115 18.7879 -4.8243

H -7.0751 19.2465 -4.5926

H -6.2892 18.0297 -5.5919

C -5.1643 19.8481 -5.3785

H -4.9301 20.5711 -4.5954

H -4.2307 19.3720 -5.6826

C -5.7736 20.5752 -6.5745

H -6.6505 21.1382 -6.2471

H -6.1146 19.8388 -7.3079

C -4.7834 21.5231 -7.2443

H -4.4177 22.2423 -6.5081

H -3.9263 20.9509 -7.6049

C -5.4161 22.2728 -8.4130

H -6.2430 22.8860 -8.0438

H -5.8355 21.5526 -9.1193

C -4.4121 23.1628 -9.1397

H -3.9766 23.8689 -8.4296

H -3.6031 22.5440 -9.5330

C -5.0587 23.9361 -10.2860

H -5.8474 24.5799 -9.8903

H -5.5284 23.2327 -10.9771

C -4.0473 24.7879 -11.0483

H -3.5587 25.4754 -10.3544

H -3.2767 24.1374 -11.4691

C -4.6998 25.5879 -12.1722

H -5.4405 26.2692 -11.7479

H -5.2264 24.9049 -12.8431

C -3.6757 26.3858 -12.9703

H -2.9468 25.7201 -13.4264

H -4.1622 26.9552 -13.7594

H -3.1463 27.0808 -12.3222

C -3.2108 14.5715 -4.3548

H -2.2303 15.0288 -4.1818

H -3.1559 13.5100 -4.0845

C -3.5659 14.7100 -5.8293

H -2.8587 14.0879 -6.3788

H -4.5715 14.3243 -5.9952

C -3.4742 16.1572 -6.3077

H -4.3149 16.7249 -5.9061

H -2.5577 16.6069 -5.9179

C -3.4645 16.2653 -7.8292

H -4.3460 15.7644 -8.2367

H -2.5826 15.7545 -8.2208

C -3.4482 17.7199 -8.2926

H -2.6393 18.2520 -7.7847

H -4.3877 18.2018 -8.0124

C -3.2458 17.8345 -9.7998

H -4.0160 17.2490 -10.3095

H -2.2726 17.4079 -10.0531

C -3.3128 19.2804 -10.2844

H -4.2708 19.7141 -9.9870

H -2.5233 19.8606 -9.8008

C -3.1651 19.3937 -11.7997

H -3.9388 18.7891 -12.2816

H -2.1953 18.9923 -12.1026

C -3.2862 20.8370 -12.2824

H -2.4839 21.4323 -11.8416

H -4.2346 21.2543 -11.9372

C -3.2170 20.9444 -13.8039

H -2.2724 20.5198 -14.1552

H -4.0269 20.3559 -14.2436

C -3.3313 22.3901 -14.2805

H -2.5049 22.9708 -13.8669

H -4.2601 22.8207 -13.9001

C -3.3117 22.4968 -15.8001

H -4.1421 21.9430 -16.2333

H -3.3927 23.5354 -16.1128

H -2.3846 22.0896 -16.2002

O -6.1731 16.4420 -1.8800

O -5.6770 16.9400 0.5533

O -4.1969 15.2109 -3.5623

O -0.2843 14.4221 -0.5392

C 6.7738 11.4459 1.0307

C 6.7732 11.1654 2.3857

H 5.8913 11.3804 2.9639

C 7.9046 10.6134 2.9738

C 9.0563 10.3464 2.2100

C 9.0643 10.6879 0.8490

C 7.9153 11.2223 0.2746

H 7.8907 11.4456 -0.7803

C 6.9401 10.5071 5.1563

H 6.0545 9.9840 4.7775

H 6.7240 11.5833 5.2231

C 7.3177 9.9495 6.5266

H 7.8515 9.0132 6.3643

H 6.3991 9.7286 7.0723

C 8.1720 10.8986 7.3668

H 7.5997 11.8063 7.5747

H 8.3802 10.4152 8.3250

C 9.4944 11.2762 6.7029

H 9.2967 11.8681 5.8084

H 10.0135 10.3704 6.3850

C 10.3808 12.0782 7.6539

H 9.8105 12.9230 8.0489

H 10.6613 11.4498 8.5030

C 11.6443 12.6056 6.9792

H 11.3623 13.2750 6.1627

H 12.2036 11.7711 6.5483

C 12.5348 13.3598 7.9645

H 11.9467 14.1418 8.4506

H 12.8728 12.6716 8.7428

C 13.7492 13.9966 7.2923

H 13.4098 14.7135 6.5405

H 14.3285 13.2254 6.7784

C 14.6431 14.7126 8.3031

H 14.0467 15.4462 8.8524

H 15.0194 13.9873 9.0278

C 15.8211 15.4215 7.6410

H 16.4092 14.6964 7.0728

H 15.4433 16.1664 6.9365

C 16.7219 16.1101 8.6633

H 17.1177 15.3632 9.3548

H 16.1275 16.8169 9.2483

C 17.8783 16.8488 8.0016

H 18.4866 16.1612 7.4190

H 18.5120 17.3202 8.7494

H 17.5025 17.6235 7.3346

C 11.4037 9.9559 2.6659

H 11.7014 9.7312 1.6378

H 11.9172 9.2635 3.3403

C 11.7835 11.3919 3.0238

H 11.3685 11.6297 4.0050

H 11.3390 12.0768 2.2974

C 13.2999 11.5537 3.0440

H 13.7171 10.8921 3.8067

H 13.7095 11.2408 2.0797

C 13.7383 12.9860 3.3358

H 13.2863 13.3197 4.2714

H 13.3807 13.6483 2.5422

C 15.2575 13.0894 3.4421

H 15.5996 12.4570 4.2652

H 15.7110 12.7057 2.5248

C 15.7362 14.5191 3.6790

H 15.2534 14.9183 4.5735

H 15.4373 15.1476 2.8359

C 17.2512 14.5824 3.8507

H 17.5383 13.9779 4.7151

H 17.7339 14.1480 2.9726

C 17.7550 16.0098 4.0485

H 17.2479 16.4543 4.9077

H 17.5004 16.6089 3.1701

C 19.2635 16.0543 4.2739

H 19.5111 15.4731 5.1657

H 19.7697 15.5841 3.4274

C 19.7802 17.4801 4.4428

H 19.2583 17.9574 5.2754

H 19.5548 18.0551 3.5410

C 21.2835 17.5191 4.7051

H 21.5017 16.9743 5.6263

H 21.8070 17.0135 3.8901

C 21.8017 18.9474 4.8260

H 21.6149 19.4991 3.9069

H 22.8713 18.9527 5.0173

H 21.3028 19.4657 5.6422

C 10.3023 10.7892 -1.2173

H 9.9310 11.8072 -1.3926

H 9.7039 10.0840 -1.8106

C 11.7785 10.7118 -1.6018

H 11.8649 10.7057 -2.6900

H 12.1845 9.7729 -1.2213

C 12.5503 11.8919 -1.0163

H 12.3119 11.9725 0.0457

H 12.2155 12.8114 -1.5031

C 14.0619 11.7626 -1.1775

H 14.4175 10.9037 -0.6029

H 14.3073 11.5815 -2.2271

C 14.7713 13.0271 -0.6988

H 14.4840 13.8626 -1.3428

H 14.4373 13.2637 0.3134

C 16.2919 12.8953 -0.7011

H 16.5890 12.1090 -0.0024

H 16.6330 12.5971 -1.6952

C 16.9642 14.2078 -0.3057

H 16.5612 14.5439 0.6519

H 16.7235 14.9721 -1.0497

C 18.4810 14.0803 -0.1909

H 18.7242 13.3519 0.5876

H 18.8875 13.7020 -1.1311

C 19.1363 15.4173 0.1438

H 18.9400 16.1268 -0.6639

H 18.6855 15.8210 1.0524

C 20.6443 15.2906 0.3457

H 21.0971 14.8647 -0.5522

H 20.8411 14.6002 1.1707

C 21.2957 16.6372 0.6505

H 21.1320 17.3158 -0.1906

H 20.8166 17.0779 1.5266

C 22.7916 16.5022 0.9072

H 22.9754 15.8352 1.7486

H 23.2316 17.4705 1.1342

H 23.2943 16.0918 0.0336

O 10.0371 9.6696 2.8811

O 8.0236 10.2785 4.2811

O 10.2155 10.4529 0.1527

O 4.4897 12.2044 1.2094

C 2.6982 12.5924 5.6396

C 3.5914 11.6779 6.2061

C 1.8146 13.2954 6.4662

C 3.6068 11.4778 7.5683

H 4.2571 11.1246 5.5635

C 1.8252 13.0841 7.8252

H 1.1252 13.9982 6.0228

C 2.7232 12.1767 8.3888

H 4.2958 10.7780 8.0147

H 1.1472 13.6114 8.4783

C 1.3895 14.1487 -4.9869

C 2.1760 13.4143 -5.8810

C 0.4676 15.0721 -5.4862

C 2.0325 13.5985 -7.2386

H 2.8872 12.7000 -5.4958

C 0.3257 15.2498 -6.8449

H -0.1232 15.6500 -4.7912

C 1.1045 14.5130 -7.7341

H 2.6269 13.0353 -7.9424

H -0.3866 15.9593 -7.2332

C -1.2913 14.7250 0.4073

H -1.5227 13.8353 1.0128

H -0.9388 15.5150 1.0865

C 5.5533 11.9747 0.3141

H 5.2506 11.2399 -0.4493

H 5.8143 12.9105 -0.2037

N -0.2689 15.6051 -12.2239

N -1.2978 14.3228 -28.0749

H -0.1103 15.5074 -10.5710

H 3.5468 10.9670 11.2790

H 0.9734 14.8260 -17.1026

H -1.6140 16.4047 -16.7090

H 0.7202 14.2941 -23.7496

H -1.9314 15.7312 -23.2441

C -6.3140 -6.5628 -5.5622

C -5.8796 -6.7540 -11.2102

C -5.8676 -6.8342 -12.4165

C -5.7653 -6.9781 -13.8107

C -6.7961 -6.5364 -14.6654

C -4.6188 -7.5723 -14.3393

H -3.8609 -7.8811 -13.6380

O -5.3724 -6.7432 -4.8148

O -7.5512 -6.3843 -5.1449

C -5.6539 -6.8698 -24.7982

C -5.4646 -7.4195 -19.1665

C -5.4125 -7.4245 -17.9574

C -5.4877 -7.3180 -16.5568

C -4.4607 -7.7628 -15.7031

C -6.6307 -6.7133 -16.0280

H -7.3745 -6.3801 -16.7330

O -6.6203 -6.3849 -25.3721

O -4.5614 -7.2493 -25.4187

C -1.3326 -9.6350 -16.1945

C -0.2576 -10.1447 -15.4758

H -0.2171 -9.9978 -14.4084

C 0.7519 -10.8256 -16.1368

C 0.7034 -11.0026 -17.5347

C -0.4017 -10.5116 -18.2417

C -1.4093 -9.8315 -17.5577

H -2.2519 -9.4229 -18.0876

C 2.0169 -11.2446 -14.1474

H 2.0629 -10.1835 -13.8635

H 1.1738 -11.7103 -13.6183

C 3.3260 -11.9382 -13.7752

H 4.0706 -11.6648 -14.5234

H 3.6549 -11.5541 -12.8070

C 3.2176 -13.4608 -13.6930

H 2.4957 -13.7298 -12.9177

H 4.1899 -13.8536 -13.3847

C 2.8131 -14.1208 -15.0104

H 1.7869 -13.8477 -15.2576

H 3.4501 -13.7459 -15.8132

C 2.9281 -15.6419 -14.9270

H 2.3539 -15.9997 -14.0688

H 3.9724 -15.9161 -14.7578

C 2.4243 -16.3399 -16.1881

H 1.3631 -16.1157 -16.3240

H 2.9596 -15.9511 -17.0578

C 2.6151 -17.8532 -16.1124

H 2.1307 -18.2319 -15.2085

H 3.6818 -18.0758 -16.0305

C 2.0411 -18.5794 -17.3270

H 0.9654 -18.3951 -17.3815

H 2.4935 -18.1796 -18.2376

C 2.2902 -20.0848 -17.2581

H 1.8748 -20.4762 -16.3263

H 3.3676 -20.2700 -17.2396

C 1.6710 -20.8327 -18.4361

H 2.0659 -20.4282 -19.3707

H 0.5897 -20.6726 -18.4359

C 1.9558 -22.3311 -18.3761

H 3.0356 -22.4926 -18.3943

H 1.5782 -22.7317 -17.4324

C 1.3123 -23.0817 -19.5361

H 1.6809 -22.7015 -20.4872

H 1.5402 -24.1440 -19.4807

H 0.2317 -22.9622 -19.5146

C 1.7788 -12.4045 -19.2015

H 1.3741 -11.8708 -20.0651

H 2.8319 -12.6383 -19.3878

C 1.0056 -13.7025 -18.9747

H 1.3142 -14.1356 -18.0207

H -0.0640 -13.4854 -18.9147

C 1.2858 -14.6841 -20.1081

H 2.3502 -14.9328 -20.1046

H 1.0667 -14.2033 -21.0643

C 0.4746 -15.9724 -19.9972

H 0.6404 -16.4224 -19.0166

H -0.5904 -15.7429 -20.0800

C 0.8735 -16.9654 -21.0857

H 1.9265 -17.2287 -20.9574

H 0.7724 -16.4897 -22.0638

C 0.0355 -18.2399 -21.0586

H 0.0966 -18.6939 -20.0682

H -1.0128 -17.9912 -21.2431

C 0.5131 -19.2440 -22.1052

H 1.5498 -19.5160 -21.8930

H 0.4947 -18.7744 -23.0913

C -0.3412 -20.5086 -22.1340

H -0.3537 -20.9565 -21.1386

H -1.3709 -20.2458 -22.3893

C 0.1877 -21.5265 -23.1406

H 1.2096 -21.8051 -22.8686

H 0.2282 -21.0688 -24.1319

C -0.6761 -22.7832 -23.2004

H -0.7327 -23.2310 -22.2050

H -1.6919 -22.5088 -23.4955

C -0.1253 -23.8129 -24.1832

H 0.8747 -24.1148 -23.8645

H -0.0329 -23.3573 -25.1714

C -1.0197 -25.0423 -24.2775

H -2.0151 -24.7638 -24.6195

H -0.6071 -25.7649 -24.9779

H -1.1147 -25.5217 -23.3057

C -1.5736 -10.3313 -20.3246

H -2.4871 -10.7461 -19.8768

H -1.6351 -9.2353 -20.3263

C -1.3978 -10.8623 -21.7445

H -2.1071 -10.3581 -22.4028

H -0.3859 -10.6186 -22.0727

C -1.6265 -12.3706 -21.8012

H -1.0352 -12.8447 -21.0165

H -2.6802 -12.5769 -21.5934

C -1.2517 -12.9861 -23.1471

H -0.1736 -12.9026 -23.2996

H -1.7422 -12.4380 -23.9546

C -1.6664 -14.4553 -23.2044

H -2.7574 -14.5183 -23.1836

H -1.2923 -14.9667 -22.3145

C -1.1470 -15.1776 -24.4438

H -0.0546 -15.1957 -24.4228

H -1.4497 -14.6337 -25.3411

C -1.6798 -16.6074 -24.5107

H -1.4687 -17.1128 -23.5658

H -2.7649 -16.5792 -24.6325

C -1.0675 -17.4149 -25.6521

H 0.0112 -17.4965 -25.4991

H -1.2260 -16.8911 -26.5976

C -1.6751 -18.8133 -25.7386

H -2.7408 -18.7284 -25.9646

H -1.5839 -19.3050 -24.7678

C -1.0020 -19.6775 -26.8009

H -1.0619 -19.1757 -27.7706

H 0.0571 -19.7908 -26.5553

C -1.6454 -21.0578 -26.9062

H -2.6918 -20.9468 -27.1988

H -1.6274 -21.5375 -25.9252

C -0.9287 -21.9466 -27.9157

H 0.1143 -22.0803 -27.6345

H -1.3997 -22.9256 -27.9678

H -0.9582 -21.4980 -28.9072

O 1.8286 -11.5743 -18.0580

O 1.8511 -11.3577 -15.5434

O -0.4380 -10.7334 -19.5862

O -3.3753 -8.3458 -16.2856

C -10.1184 -5.0584 -14.1551

C -10.0912 -4.8667 -12.7841

H -9.1908 -5.0929 -12.2384

C -11.2252 -4.3810 -12.1481

C -12.4074 -4.0976 -12.8673

C -12.4290 -4.3306 -14.2489

C -11.2728 -4.7920 -14.8695

H -11.2544 -4.9333 -15.9377

C -10.2976 -4.5483 -9.9442

H -9.3364 -4.0899 -10.2134

H -10.2018 -5.6422 -10.0054

C -10.6465 -4.1273 -8.5202

H -10.7622 -3.0434 -8.5022

H -9.7830 -4.3797 -7.9049

C -11.9103 -4.7895 -7.9675

H -11.9551 -5.8274 -8.3060

H -11.8556 -4.8037 -6.8755

C -13.1836 -4.0573 -8.3891

H -13.2036 -3.9744 -9.4760

H -13.1625 -3.0458 -7.9769

C -14.4466 -4.7670 -7.9060

H -14.5109 -5.7519 -8.3768

H -14.3884 -4.9267 -6.8252

C -15.7067 -3.9674 -8.2306

H -15.7163 -3.7372 -9.2973

H -15.6809 -3.0197 -7.6874

C -16.9813 -4.7249 -7.8673

H -16.9886 -5.6857 -8.3876

H -16.9873 -4.9341 -6.7953

C -18.2431 -3.9490 -8.2382

H -18.2221 -3.7198 -9.3059

H -18.2577 -2.9988 -7.6970

C -19.5105 -4.7363 -7.9183

H -19.4722 -5.6998 -8.4317

H -19.5495 -4.9376 -6.8443

C -20.7795 -3.9990 -8.3388

H -20.8227 -3.0301 -7.8354

H -20.7461 -3.8087 -9.4141

C -22.0399 -4.7964 -8.0091

H -22.1037 -4.9374 -6.9272

H -21.9696 -5.7857 -8.4658

C -23.3013 -4.0998 -8.5042

H -23.3987 -3.1163 -8.0496

H -24.1851 -4.6822 -8.2529

H -23.2699 -3.9737 -9.5844

C -14.7459 -3.5707 -12.4270

H -14.8981 -3.3049 -13.4734

H -15.1836 -2.7928 -11.7919

C -15.4278 -4.9049 -12.1187

H -15.1640 -5.2111 -11.1048

H -15.0619 -5.6732 -12.8028

C -16.9425 -4.7552 -12.2402

H -17.2637 -3.9196 -11.6120

H -17.2021 -4.5052 -13.2709

C -17.7024 -6.0080 -11.8146

H -17.4342 -6.2610 -10.7870

H -17.4124 -6.8495 -12.4479

C -19.2118 -5.7932 -11.9032

H -19.4835 -4.9353 -11.2846

H -19.4856 -5.5553 -12.9331

C -20.0071 -7.0094 -11.4381

H -19.7281 -7.2516 -10.4093

H -19.7513 -7.8738 -12.0568

C -21.5107 -6.7572 -11.5081

H -21.7533 -5.8761 -10.9102

H -21.7940 -6.5417 -12.5403

C -22.3219 -7.9456 -11.0007

H -22.0266 -8.1752 -9.9737

H -22.0965 -8.8256 -11.6086

C -23.8234 -7.6717 -11.0433

H -24.0508 -6.8091 -10.4117

H -24.1125 -7.4159 -12.0650

C -24.6405 -8.8715 -10.5741

H -24.3587 -9.1235 -9.5484

H -24.4043 -9.7363 -11.1998

C -26.1423 -8.6034 -10.6338

H -26.3841 -7.7624 -9.9795

H -26.4179 -8.3211 -11.6510

C -26.9540 -9.8233 -10.2138

H -26.7441 -10.6639 -10.8722

H -28.0196 -9.6096 -10.2599

H -26.7057 -10.1166 -9.1958

C -13.6590 -4.5338 -16.2976

H -13.3024 -5.5677 -16.3966

H -13.0263 -3.8800 -16.9152

C -15.1108 -4.4493 -16.7610

H -15.1305 -4.5515 -17.8467

H -15.5065 -3.4649 -16.5078

C -15.9822 -5.5287 -16.1240

H -15.8352 -5.5091 -15.0434

H -15.6652 -6.5103 -16.4863

C -17.4633 -5.3265 -16.4367

H -17.7965 -4.3792 -16.0070

H -17.6018 -5.2628 -17.5191

C -18.3152 -6.4658 -15.8836

H -18.0362 -7.3983 -16.3792

H -18.1075 -6.5847 -14.8190

C -19.8110 -6.2229 -16.0757

H -20.0978 -5.3065 -15.5533

H -20.0231 -6.0752 -17.1379

C -20.6443 -7.3878 -15.5490

H -20.3904 -7.5668 -14.5022

H -20.3908 -8.2927 -16.1072

C -22.1445 -7.1296 -15.6648

H -22.3997 -6.2274 -15.1026

H -22.4017 -6.9468 -16.7105

C -22.9666 -8.3034 -15.1372

H -22.7285 -9.1992 -15.7153

H -22.6899 -8.4989 -14.0997

C -24.4682 -8.0382 -15.2150

H -24.7490 -7.8468 -16.2536

H -24.7054 -7.1384 -14.6408

C -25.2865 -9.2089 -14.6783

H -25.0548 -10.1062 -15.2575

H -24.9991 -9.4028 -13.6427

C -26.7840 -8.9356 -14.7459

H -27.0352 -8.0427 -14.1749

H -27.3455 -9.7726 -14.3380

H -27.0976 -8.7787 -15.7751

O -13.3790 -3.5530 -12.0812

O -11.3229 -4.1220 -10.8202

O -13.5939 -4.1216 -14.9462

O -7.8754 -5.9580 -14.0829

C -5.9647 -6.6636 -9.8078

C -7.2124 -6.4658 -9.2122

C -4.8267 -6.7918 -9.0043

C -7.3259 -6.4165 -7.8414

H -8.0813 -6.3661 -9.8418

C -4.9451 -6.7372 -7.6325

H -3.8633 -6.9415 -9.4695

C -6.1937 -6.5607 -7.0412

H -8.2872 -6.2755 -7.3744

H -4.0819 -6.8398 -6.9930

C -5.5216 -7.3426 -20.5686

C -6.6081 -6.7002 -21.1773

C -4.5026 -7.8736 -21.3654

C -6.6554 -6.5654 -22.5455

H -7.3968 -6.3014 -20.5562

C -4.5572 -7.7392 -22.7337

H -3.6754 -8.3814 -20.8940

C -5.6219 -7.0702 -23.3360

H -7.4801 -6.0642 -23.0301

H -3.7829 -8.1468 -23.3639

C -2.3925 -8.8861 -15.4295

H -1.9125 -8.0775 -14.8558

H -2.8641 -9.5765 -14.7132

C -8.9281 -5.5564 -14.9333

H -8.5837 -4.7573 -15.6090

H -9.2487 -6.4073 -15.5524

C -5.4887 -7.3416 8.9427

C -6.5761 -7.3727 8.1569

C -6.6072 -7.4328 6.7129

C -7.8273 -7.1537 6.0545

C -5.4914 -7.7600 5.9402

H -4.5746 -7.9989 6.4537

C -7.7452 -6.9241 1.6906

C -6.7713 -7.4403 2.4561

C -6.7484 -7.4666 3.8999

C -5.5421 -7.7949 4.5574

C -7.8697 -7.1669 4.6721

H -8.7916 -6.9536 4.1602

C -2.3565 -9.1355 3.2940

C -1.3078 -9.9479 3.7091

H -1.2642 -10.2678 4.7375

C -0.3297 -10.3298 2.8044

C -0.3955 -9.8920 1.4687

C -1.4609 -9.0767 1.0546

C -2.4401 -8.7164 1.9790

H -3.2581 -8.0769 1.6965

C 1.0124 -11.4340 4.4467

H 1.0417 -10.5157 5.0485

H 0.2309 -12.0934 4.8510

C 2.3730 -12.1279 4.4969

H 3.0368 -11.5978 3.8155

H 2.7712 -12.0322 5.5089

C 2.3271 -13.6079 4.1178

H 1.7169 -14.1504 4.8451

H 3.3422 -14.0081 4.1770

C 1.7751 -13.8492 2.7144

H 0.7188 -13.5796 2.6912

H 2.2917 -13.1985 2.0076

C 1.9278 -15.3056 2.2788

H 1.5089 -15.9610 3.0467

H 2.9891 -15.5489 2.1814

C 1.2196 -15.5696 0.9529

H 0.1523 -15.3695 1.0787

H 1.5988 -14.8783 0.1966

C 1.4013 -17.0032 0.4597

H 1.0849 -17.6986 1.2401

H 2.4595 -17.1888 0.2612

C 0.5908 -17.2655 -0.8082

H -0.4680 -17.1037 -0.5910

H 0.8819 -16.5468 -1.5773

C 0.7762 -18.6829 -1.3432

H 0.5162 -19.4008 -0.5621

H 1.8267 -18.8400 -1.5994

C -0.0914 -18.9393 -2.5731

H 0.1645 -18.2173 -3.3522

H -1.1397 -18.7817 -2.3110

C 0.0782 -20.3538 -3.1211

H 1.1212 -20.5076 -3.4092

H -0.1560 -21.0759 -2.3350

C -0.8241 -20.6055 -4.3230

H -0.5995 -19.9025 -5.1223

H -0.6833 -21.6148 -4.7049

H -1.8679 -20.4852 -4.0464

C 0.3516 -10.8938 -0.5656

H -0.1302 -10.1973 -1.2608

H 1.3293 -11.1743 -0.9667

C -0.5224 -12.1314 -0.3670

H -0.0218 -12.8047 0.3316

H -1.4656 -11.8229 0.0906

C -0.8100 -12.8574 -1.6763

H 0.1252 -13.2261 -2.1027

H -1.2466 -12.1570 -2.3914

C -1.7675 -14.0272 -1.4598

H -1.3360 -14.7062 -0.7223

H -2.7076 -13.6488 -1.0521

C -2.0545 -14.8031 -2.7418

H -1.1150 -15.1750 -3.1573

H -2.5007 -14.1339 -3.4814

C -2.9919 -15.9792 -2.4853

H -2.5345 -16.6448 -1.7497

H -3.9270 -15.6105 -2.0579

C -3.2964 -16.7728 -3.7527

H -2.3591 -17.0890 -4.2163

H -3.8170 -16.1315 -4.4678

C -4.1493 -18.0045 -3.4589

H -3.6039 -18.6624 -2.7770

H -5.0673 -17.6987 -2.9524

C -4.5049 -18.7798 -4.7250

H -3.5896 -19.0186 -5.2726

H -5.1186 -18.1503 -5.3744

C -5.2547 -20.0713 -4.4111

H -4.6275 -20.7087 -3.7828

H -6.1579 -19.8377 -3.8416

C -5.6401 -20.8374 -5.6741

H -4.7388 -21.0488 -6.2553

H -6.2897 -20.2123 -6.2916

C -6.3507 -22.1456 -5.3493

H -7.2535 -21.9585 -4.7725

H -6.6290 -22.6680 -6.2625

H -5.7019 -22.7957 -4.7662

C -2.4652 -8.0180 -0.9070

H -3.1835 -7.5743 -0.2161

H -1.9984 -7.2064 -1.4734

C -3.1547 -8.9733 -1.8831

H -3.7708 -8.3839 -2.5651

H -2.3809 -9.4657 -2.4756

C -4.0162 -10.0331 -1.2028

H -3.4164 -10.5834 -0.4732

H -4.8388 -9.5550 -0.6666

C -4.5784 -11.0054 -2.2360

H -3.7482 -11.4772 -2.7672

H -5.1623 -10.4445 -2.9704

C -5.4535 -12.0959 -1.6242

H -6.3187 -11.6409 -1.1354

H -4.8871 -12.6316 -0.8585

C -5.9245 -13.0822 -2.6896

H -5.0514 -13.4945 -3.2008

H -6.5179 -12.5476 -3.4344

C -6.7507 -14.2300 -2.1165

H -6.1595 -14.7651 -1.3677

H -7.6331 -13.8308 -1.6125

C -7.1820 -15.2047 -3.2107

H -6.2974 -15.5556 -3.7459

H -7.8143 -14.6787 -3.9296

C -7.9412 -16.4081 -2.6584

H -8.8212 -16.0636 -2.1092

H -7.3044 -16.9453 -1.9506

C -8.3774 -17.3598 -3.7702

H -9.0483 -16.8325 -4.4522

H -7.5001 -17.6674 -4.3448

C -9.0818 -18.6008 -3.2294

H -9.9475 -18.2963 -2.6378

H -8.4027 -19.1411 -2.5646

C -9.5363 -19.5254 -4.3528

H -8.6867 -19.8371 -4.9566

H -10.0157 -20.4147 -3.9496

H -10.2466 -19.0169 -5.0011

O 0.6550 -10.2474 0.6595

O 0.7391 -11.1082 3.0991

O -1.3933 -8.6487 -0.2331

O -4.4834 -8.1070 3.7539

C -11.1624 -6.2261 7.2657

C -10.8707 -6.2059 8.6185

H -9.8966 -6.5132 8.9541

C -11.8204 -5.7473 9.5163

C -13.0908 -5.3146 9.0773

C -13.3989 -5.4037 7.7114

C -12.4196 -5.8500 6.8278

H -12.6272 -5.8957 5.7708

C -10.5556 -6.3575 11.4479

H -9.5934 -5.9164 11.1545

H -10.5784 -7.4069 11.1172

C -10.6955 -6.2826 12.9670

H -10.6846 -5.2350 13.2657

H -9.8133 -6.7617 13.3949

C -11.9621 -6.9602 13.4955

H -12.1609 -7.8611 12.9098

H -11.7924 -7.2732 14.5292

C -13.1876 -6.0477 13.4517

H -13.2942 -5.6341 12.4497

H -13.0337 -5.2159 14.1444

C -14.4626 -6.7942 13.8352

H -14.6632 -7.5668 13.0874

H -14.3210 -7.2987 14.7950

C -15.6688 -5.8627 13.9307

H -15.7341 -5.2669 13.0184

H -15.5260 -5.1701 14.7643

C -16.9724 -6.6324 14.1288

H -17.1098 -7.3241 13.2932

H -16.9046 -7.2307 15.0401

C -18.1860 -5.7104 14.2201

H -18.2351 -5.0899 13.3222

H -18.0697 -5.0427 15.0763

C -19.4889 -6.4938 14.3610

H -19.6041 -7.1565 13.4999

H -19.4377 -7.1228 15.2538

C -20.7087 -5.5807 14.4582

H -20.6091 -4.9361 15.3350

H -20.7468 -4.9353 13.5777

C -22.0114 -6.3717 14.5570

H -21.9736 -7.0220 15.4334

H -22.1106 -7.0101 13.6762

C -23.2272 -5.4586 14.6572

H -23.1661 -4.8380 15.5486

H -24.1430 -6.0437 14.7084

H -23.2862 -4.8023 13.7908

C -15.1877 -4.4063 9.9395

H -15.3286 -3.8347 9.0208

H -15.3571 -3.7428 10.7943

C -16.1745 -5.5719 9.9990

H -15.9418 -6.1852 10.8713

H -16.0524 -6.1918 9.1098

C -17.6041 -5.0489 10.0942

H -17.6846 -4.3923 10.9640

H -17.8274 -4.4480 9.2108

C -18.6350 -6.1671 10.2214

H -18.4139 -6.7644 11.1078

H -18.5690 -6.8285 9.3535

C -20.0484 -5.6018 10.3302

H -20.1027 -4.9474 11.2030

H -20.2599 -4.9904 9.4500

C -21.1126 -6.6873 10.4548

H -20.9012 -7.3038 11.3310

H -21.0734 -7.3402 9.5781

C -22.5092 -6.0863 10.5812

H -22.5423 -5.4422 11.4629

H -22.7110 -5.4598 9.7092

C -23.5980 -7.1494 10.6981

H -23.3953 -7.7852 11.5620

H -23.5778 -7.7873 9.8106

C -24.9828 -6.5247 10.8439

H -25.0072 -5.9138 11.7491

H -25.1690 -5.8632 9.9944

C -26.0879 -7.5740 10.9140

H -25.9055 -8.2387 11.7624

H -26.0627 -8.1861 10.0078

C -27.4694 -6.9398 11.0559

H -27.5061 -6.3633 11.9835

H -27.6325 -6.2461 10.2289

C -28.5786 -7.9840 11.0641

H -28.5757 -8.5514 10.1345

H -29.5509 -7.5091 11.1710

H -28.4428 -8.6805 11.8889

C -14.9733 -5.1641 5.9160

H -14.9286 -6.2196 5.6113

H -14.2582 -4.5918 5.3103

C -16.3729 -4.6021 5.6858

H -16.4861 -4.4274 4.6141

H -16.4428 -3.6372 6.1891

C -17.4809 -5.5336 6.1677

H -17.3021 -5.7995 7.2100

H -17.4575 -6.4554 5.5799

C -18.8562 -4.8834 6.0378

H -18.9184 -4.0351 6.7234

H -18.9783 -4.4957 5.0233

C -19.9898 -5.8605 6.3390

H -19.9764 -6.6666 5.6019

H -19.8299 -6.3091 7.3206

C -21.3569 -5.1809 6.3165

H -21.3913 -4.4116 7.0930

H -21.4994 -4.6803 5.3557

C -22.4919 -6.1761 6.5414

H -22.3207 -6.7110 7.4778

H -22.4882 -6.9141 5.7354

C -23.8581 -5.4979 6.5960

H -23.8745 -4.7829 7.4227

H -24.0217 -4.9353 5.6731

C -24.9893 -6.5068 6.7797

H -24.9884 -7.2045 5.9382

H -24.8092 -7.0865 7.6874

C -26.3557 -5.8343 6.8752

H -26.5304 -5.2365 5.9768

H -26.3635 -5.1526 7.7294

C -27.4846 -6.8505 7.0328

H -27.4883 -7.5200 6.1687

H -27.2988 -7.4580 7.9200

C -28.8454 -6.1767 7.1573

H -28.8675 -5.5164 8.0222

H -29.6319 -6.9189 7.2733

H -29.0602 -5.5829 6.2708

O -13.8464 -4.8145 10.0950

O -11.6254 -5.6494 10.8562

O -14.6557 -5.0434 7.2883

O -8.8962 -6.8801 6.8445

C -5.5130 -7.2807 10.3918

C -4.3095 -7.2535 11.1036

C -6.6895 -7.2266 11.1463

C -4.3388 -7.1574 12.4822

H -3.3638 -7.2912 10.5811

C -6.6004 -7.1464 12.5228

H -7.6570 -7.2580 10.6727

H -3.4242 -7.1161 13.0579

H -7.4931 -7.1030 13.1345

C -7.7085 -6.8287 0.2479

C -8.8884 -6.5574 -0.4555

C -6.5339 -6.9471 -0.5016

C -8.8434 -6.4404 -1.8279

H -9.8216 -6.4456 0.0742

C -6.5939 -6.8052 -1.8717

H -5.5851 -7.1160 -0.0210

H -9.7328 -6.2368 -2.4089

H -5.7137 -6.8793 -2.4959

C -3.3368 -8.6730 4.3448

H -2.8335 -7.9274 4.9826

H -3.6219 -9.5256 4.9779

C -10.1389 -6.6189 6.2319

H -10.0376 -5.7912 5.5143

H -10.4839 -7.5124 5.6881

N -5.4566 -7.1104 13.1867

N -7.7233 -6.5642 -2.5225

H -7.6145 -6.4227 -4.1315

H -4.5932 -7.0571 -26.4079

H -4.4992 -7.3037 8.5054

H -7.5534 -7.2986 8.6080

H -5.8947 -7.8644 1.9877

H -8.6391 -6.5203 2.1456

C -6.6261 -0.8454 8.0541

C -5.6627 -1.1594 7.1724

C -5.7389 -1.0979 5.7321

C -4.5998 -1.4660 4.9828

C -6.8764 -0.6785 5.0350

H -7.7430 -0.3992 5.6082

C -4.6891 -1.0929 0.6888

C -5.7503 -0.8301 1.4686

C -5.7640 -0.9330 2.9092

C -6.9183 -0.6075 3.6528

C -4.6352 -1.3545 3.6073

H -3.7677 -1.5865 3.0208

O -7.9978 -0.2291 2.9241

C -1.2492 -2.9177 5.5511

C -1.3225 -3.4775 6.8108

H -2.2429 -3.4072 7.3649

C -0.2090 -4.1301 7.3304

C 0.9521 -4.3056 6.5530

C 1.0169 -3.7167 5.2747

C -0.0727 -2.9855 4.8213

H -0.0384 -2.5021 3.8595

C -1.1245 -4.2338 9.5221

H -2.0893 -4.7329 9.3435

H -1.2758 -3.1511 9.4260

C -0.6356 -4.5522 10.9330

H -0.4893 -5.6296 11.0262

H -1.4310 -4.2556 11.6196

C 0.6691 -3.8313 11.2903

H 0.6901 -2.8544 10.8008

H 0.6976 -3.6570 12.3691

C 1.9061 -4.6297 10.8828

H 1.8206 -4.9101 9.8334

H 1.9389 -5.5499 11.4722

C 3.2008 -3.8498 11.1039

H 3.2360 -2.9977 10.4196

H 3.2207 -3.4516 12.1210

C 4.4254 -4.7376 10.8871

H 4.3289 -5.2511 9.9282

H 4.4497 -5.5045 11.6657

C 5.7392 -3.9613 10.9081

H 5.7485 -3.2387 10.0878

H 5.8167 -3.3976 11.8397

C 6.9407 -4.8953 10.7806

H 6.8442 -5.4876 9.8675

H 6.9394 -5.5933 11.6213

C 8.2715 -4.1470 10.7562

H 8.2867 -3.4593 9.9074

H 8.3660 -3.5500 11.6663

C 9.4569 -5.1054 10.6573

H 9.4334 -5.7931 11.5051

H 9.3636 -5.7057 9.7481

C 10.7954 -4.3724 10.6427

H 10.8826 -3.7674 11.5469

H 10.8251 -3.6973 9.7837

C 11.9724 -5.3363 10.5687

H 11.9925 -5.9835 11.4442

H 12.9134 -4.7922 10.5229

H 11.8976 -5.9671 9.6841

C 3.2686 -4.7960 7.0284

H 3.5112 -4.1377 7.8746

H 3.4963 -4.2645 6.1025

C 4.0832 -6.0842 7.1002

H 3.7454 -6.7505 6.3058

H 3.8832 -6.5816 8.0516

C 5.5767 -5.8095 6.9490

H 5.7514 -5.2980 6.0007

H 5.9041 -5.1397 7.7464

C 6.4124 -7.0867 6.9839

H 6.0645 -7.7694 6.2042

H 6.2738 -7.5895 7.9444

C 7.8955 -6.7901 6.7740

H 8.0231 -6.2562 5.8290

H 8.2471 -6.1312 7.5703

C 8.7532 -8.0521 6.7482

H 8.3985 -8.7155 5.9558

H 8.6450 -8.5866 7.6952

C 10.2275 -7.7274 6.5146

H 10.3283 -7.1636 5.5841

H 10.5887 -7.0885 7.3247

C 11.0965 -8.9800 6.4360

H 10.7329 -9.6205 5.6279

H 11.0045 -9.5458 7.3655

C 12.5649 -8.6414 6.1927

H 12.6525 -8.0551 5.2734

H 12.9338 -8.0219 7.0134

C 13.4336 -9.8902 6.0730

H 13.0623 -10.5112 5.2533

H 13.3504 -10.4795 6.9893

C 14.9013 -9.5503 5.8237

H 14.9834 -8.9353 4.9249

H 15.2832 -8.9629 6.6620

C 15.7527 -10.8024 5.6531

H 15.6983 -11.4259 6.5438

H 16.7935 -10.5381 5.4822

H 15.4037 -11.3892 4.8058

C 2.2258 -3.3661 3.2151

H 2.5278 -2.3112 3.2842

H 1.2623 -3.4235 2.7031

C 3.2229 -4.1710 2.3872

H 3.3646 -3.6383 1.4442

H 2.7665 -5.1344 2.1623

C 4.5669 -4.3915 3.0705

H 4.4073 -4.9474 3.9961

H 5.0138 -3.4294 3.3284

C 5.5199 -5.1736 2.1718

H 5.0319 -6.0963 1.8545

H 5.7346 -4.5850 1.2767

C 6.8286 -5.5217 2.8742

H 7.3067 -4.6066 3.2304

H 6.6146 -6.1461 3.7448

C 7.7886 -6.2609 1.9492

H 7.3090 -7.1736 1.5900

H 8.0031 -5.6346 1.0797

C 9.0979 -6.6234 2.6424

H 8.8870 -7.2739 3.4945

H 9.5674 -5.7148 3.0265

C 10.0675 -7.3265 1.6979

H 9.5986 -8.2373 1.3178

H 10.2700 -6.6770 0.8427

C 11.3829 -7.6851 2.3811

H 11.8475 -6.7755 2.7713

H 11.1793 -8.3435 3.2292

C 12.3543 -8.3762 1.4288

H 12.5555 -7.7193 0.5788

H 11.8907 -9.2861 1.0417

C 13.6706 -8.7351 2.1121

H 14.1393 -7.8253 2.4932

H 13.4638 -9.3856 2.9642

C 14.6327 -9.4395 1.1646

H 14.1979 -10.3669 0.8002

H 15.5667 -9.6751 1.6712

H 14.8582 -8.8057 0.3082

O 1.8920 -5.1403 7.0907

O -0.1680 -4.6717 8.5755

O 2.1396 -3.9113 4.5250

O -3.4938 -1.9227 5.6524

C -6.5035 -0.9149 9.4954

C -7.6102 -0.6015 10.2939

C -5.3331 -1.2780 10.1684

C -7.4986 -0.6735 11.6691

H -8.5451 -0.3140 9.8360

C -5.3302 -1.3099 11.5511

H -4.4373 -1.5339 9.6265

H -8.3390 -0.4447 12.3106

H -4.4387 -1.5823 12.0991

C -4.6062 -0.9674 -0.7454

C -3.3701 -1.2095 -1.3587

C -5.6696 -0.6026 -1.5763

C -3.2592 -1.0835 -2.7268

H -2.5128 -1.4878 -0.7638

C -5.4574 -0.5131 -2.9368

H -6.6464 -0.4005 -1.1703

H -2.3222 -1.2529 -3.2393

H -6.2432 -0.2400 -3.6267

C -9.2255 0.0176 3.5695

H -9.4912 -0.8397 4.2065

H -9.1509 0.9144 4.2000

C -2.4319 -2.3422 4.8272

H -2.7906 -3.1317 4.1440

H -2.0857 -1.5053 4.2070

C -5.2758 -0.2223 -6.5887

C -5.5130 0.1560 -12.2300

C -5.6461 0.2048 -13.4305

C -5.9452 0.2791 -14.8019

C -5.0853 -0.2535 -15.7810

C -7.1506 0.8772 -15.1724

H -7.7431 1.2999 -14.3787

O -6.2414 0.0067 -5.8913

O -4.1109 -0.6150 -6.1152

C -4.8496 -0.5801 -25.3176

C -6.7338 0.3529 -20.0623

C -6.8863 0.4604 -18.8647

C -6.7003 0.4331 -17.4708

C -7.5750 0.9384 -16.4931

C -5.4783 -0.1464 -17.0990

H -4.8566 -0.5069 -17.9007

O -3.6978 -0.9653 -25.4761

O -5.6944 -0.3883 -26.2969

H -5.3094 -0.6409 -27.1913

O -8.7639 1.4632 -16.8947

C -1.8624 -2.0168 -15.7796

C -1.6678 -2.1331 -14.4149

H -2.3806 -1.6655 -13.7577

C -0.5774 -2.8526 -13.9333

C 0.3219 -3.4586 -14.8284

C 0.1330 -3.3135 -16.2147

C -0.9591 -2.5797 -16.6690

H -1.1314 -2.4549 -17.7265

C -1.0110 -2.2556 -11.6747

H -2.0904 -2.4243 -11.7805

H -0.8082 -1.1918 -11.8576

C -0.5991 -2.6216 -10.2515

H -0.8652 -3.6622 -10.0643

H -1.1973 -1.9963 -9.5858

C 0.8936 -2.4243 -9.9638

H 1.2954 -1.6514 -10.6210

H 1.0191 -2.0791 -8.9344

C 1.6897 -3.7145 -10.1492

H 1.5464 -4.0782 -11.1679

H 1.2973 -4.4695 -9.4638

C 3.1777 -3.5190 -9.8695

H 3.6153 -2.9058 -10.6610

H 3.3054 -2.9777 -8.9292

C 3.9227 -4.8495 -9.7855

H 3.6805 -5.4540 -10.6639

H 3.5791 -5.4010 -8.9070

C 5.4357 -4.6640 -9.7100

H 5.7692 -4.1029 -10.5864

H 5.6892 -4.0725 -8.8268

C 6.1764 -5.9991 -9.6600

H 5.8326 -6.6296 -10.4849

H 5.9335 -6.5168 -8.7299

C 7.6890 -5.8221 -9.7677

H 7.9210 -5.2855 -10.6905

H 8.0422 -5.2083 -8.9360

C 8.4250 -7.1594 -9.7678

H 8.2383 -7.6783 -8.8256

H 8.0304 -7.7874 -10.5717

C 9.9295 -6.9894 -9.9625

H 10.3290 -6.3625 -9.1617

H 10.1133 -6.4698 -10.9057

C 10.6592 -8.3272 -9.9716

H 10.5107 -8.8499 -9.0295

H 11.7278 -8.1798 -10.1162

H 10.2863 -8.9593 -10.7746

C 2.6590 -3.7689 -14.5514

H 2.7995 -2.7569 -14.1451

H 2.8347 -3.7367 -15.6328

C 3.6169 -4.7587 -13.8970

H 3.2499 -5.7608 -14.1223

H 3.5860 -4.6269 -12.8149

C 5.0466 -4.6104 -14.4072

H 5.0409 -4.6840 -15.4974

H 5.4345 -3.6241 -14.1450

C 5.9642 -5.6951 -13.8452

H 5.5098 -6.6720 -14.0243

H 6.0614 -5.5690 -12.7651

C 7.3490 -5.6739 -14.4884

H 7.2398 -5.7507 -15.5728

H 7.8390 -4.7203 -14.2766

C 8.2279 -6.8213 -13.9954

H 7.7148 -7.7688 -14.1751

H 8.3760 -6.7279 -12.9167

C 9.5863 -6.8506 -14.6924

H 9.4355 -6.9423 -15.7700

H 10.1058 -5.9065 -14.5157

C 10.4582 -8.0055 -14.2065

H 9.9385 -8.9505 -14.3822

H 10.6146 -7.9120 -13.1289

C 11.8144 -8.0393 -14.9076

H 11.6617 -8.1481 -15.9845

H 12.3281 -7.0896 -14.7458

C 12.6976 -9.1777 -14.4045

H 12.1922 -10.1313 -14.5733

H 12.8452 -9.0687 -13.3276

C 14.0570 -9.1980 -15.0983

H 13.9126 -9.3149 -16.1743

H 14.5570 -8.2409 -14.9355

C 14.9451 -10.3243 -14.5840

H 15.1259 -10.2097 -13.5170

H 15.9048 -10.3203 -15.0959

H 14.4721 -11.2888 -14.7489

C 1.0531 -3.5244 -18.4070

H 1.2718 -2.4511 -18.4558

H 0.0774 -3.7072 -18.8729

C 2.0884 -4.3247 -19.1918

H 2.1158 -3.9089 -20.1997

H 1.7371 -5.3535 -19.2626

C 3.4998 -4.3295 -18.6082

H 3.4480 -4.5543 -17.5415

H 3.9616 -3.3487 -18.7301

C 4.3377 -5.3998 -19.3053

H 3.8634 -6.3690 -19.1372

H 4.3274 -5.2155 -20.3817

C 5.7817 -5.4654 -18.8136

H 6.3119 -4.5539 -19.0954

H 5.7929 -5.5247 -17.7213

C 6.5069 -6.6825 -19.3839

H 5.9802 -7.5881 -19.0700

H 6.4712 -6.6441 -20.4755

C 7.9605 -6.7724 -18.9286

H 7.9961 -6.7892 -17.8356

H 8.5017 -5.8834 -19.2617

C 8.6537 -8.0204 -19.4714

H 8.1263 -8.9079 -19.1152

H 8.5966 -8.0160 -20.5625

C 10.1171 -8.0989 -19.0449

H 10.6433 -7.2080 -19.3960

H 10.1747 -8.1055 -17.9528

C 10.8113 -9.3429 -19.5927

H 10.7304 -9.3524 -20.6820

H 10.3043 -10.2356 -19.2165

C 12.2859 -9.3999 -19.1998

H 12.7890 -8.4983 -19.5568

H 12.3684 -9.4144 -18.1098

C 12.9797 -10.6280 -19.7734

H 12.5192 -11.5369 -19.3944

H 14.0336 -10.6377 -19.5013

H 12.9063 -10.6362 -20.8597

O 1.3348 -4.2150 -14.2875

O -0.3002 -3.0361 -12.6146

O 1.0260 -3.9252 -17.0418

O -3.9210 -0.8170 -15.3590

C -5.4124 0.0893 -10.8287

C -4.3576 -0.5909 -10.2176

C -6.3991 0.6919 -10.0418

C -4.2979 -0.6783 -8.8446

H -3.5934 -1.0415 -10.8345

C -6.3453 0.5843 -8.6701

H -7.2005 1.2348 -10.5206

C -5.2986 -0.1040 -8.0661

H -3.4914 -1.2040 -8.3575

H -7.1006 1.0286 -8.0423

C -6.3491 0.1513 -21.4010

C -5.0459 -0.3319 -21.5958

C -7.1664 0.3909 -22.5065

C -4.5735 -0.5544 -22.8657

H -4.4239 -0.5215 -20.7338

C -6.6902 0.1584 -23.7806

H -8.1710 0.7598 -22.3571

C -5.3923 -0.3124 -23.9691

H -3.5739 -0.9228 -23.0340

H -7.3122 0.3397 -24.6427

C -9.6402 1.9004 -15.8721

H -9.7059 1.1353 -15.0895

H -9.2450 2.8246 -15.4238

C -3.0657 -1.3215 -16.3597

H -3.6064 -2.0471 -16.9841

H -2.7421 -0.4972 -17.0131

N -6.3844 -1.0175 12.2943

N -4.2815 -0.7540 -3.5002

H -4.1472 -0.6783 -5.1010

C -10.3097 0.2064 2.5370

C -10.0557 0.0474 1.1837

H -9.0515 -0.1614 0.8587

C -11.0964 0.1233 0.2716

C -12.4198 0.3415 0.7064

C -12.6649 0.5465 2.0743

C -11.5950 0.4897 2.9628

H -11.7698 0.6345 4.0158

C -9.6704 -0.0805 -1.6371

H -9.1312 -0.9606 -1.2525

H -9.1029 0.8220 -1.3669

C -9.7838 -0.2011 -3.1552

H -10.4638 -1.0241 -3.3802

H -8.7942 -0.4622 -3.5350

C -10.2698 1.0579 -3.8740

H -9.7461 1.9308 -3.4784

H -9.9910 0.9614 -4.9263

C -11.7796 1.2784 -3.7885

H -12.0607 1.5342 -2.7669

H -12.2894 0.3455 -4.0410

C -12.2356 2.3676 -4.7599

H -11.7219 3.3039 -4.5273

H -11.9436 2.0793 -5.7729

C -13.7451 2.5981 -4.7282

H -14.0259 3.0201 -3.7601

H -14.2606 1.6396 -4.8310

C -14.2106 3.5406 -5.8378

H -13.6366 4.4684 -5.7873

H -14.0078 3.0795 -6.8075

C -15.6995 3.8658 -5.7346

H -15.8860 4.3912 -4.7949

H -16.2745 2.9371 -5.7171

C -16.1866 4.7350 -6.8924

H -15.5562 5.6246 -6.9664

H -16.0832 4.1798 -7.8276

C -17.6424 5.1643 -6.7146

H -18.2719 4.2780 -6.6085

H -17.7333 5.7506 -5.7959

C -18.1417 5.9996 -7.8899

H -18.0878 5.4004 -8.8028

H -17.4859 6.8626 -8.0233

C -19.5722 6.4831 -7.6843

H -20.2522 5.6397 -7.5746

H -19.9020 7.0811 -8.5318

H -19.6413 7.0965 -6.7881

C -14.6463 0.7683 -0.1059

H -14.6249 1.7319 0.4125

H -15.2049 0.0499 0.5048

C -15.3159 0.9489 -1.4641

H -15.3736 -0.0123 -1.9756

H -14.7000 1.6096 -2.0737

C -16.7058 1.5474 -1.2748

H -17.3216 0.8483 -0.7048

H -16.6138 2.4613 -0.6830

C -17.4103 1.8918 -2.5836

H -17.5904 0.9833 -3.1634

H -16.7674 2.5408 -3.1813

C -18.7321 2.6034 -2.3065

H -19.3780 1.9461 -1.7220

H -18.5331 3.4916 -1.7020

C -19.4661 3.0312 -3.5731

H -19.7177 2.1502 -4.1686

H -18.8093 3.6605 -4.1780

C -20.7396 3.8024 -3.2370

H -21.4080 3.1588 -2.6613

H -20.4828 4.6570 -2.6069

C -21.4674 4.3034 -4.4805

H -21.7250 3.4543 -5.1175

H -20.7991 4.9511 -5.0532

C -22.7364 5.0721 -4.1235

H -23.4155 4.4141 -3.5760

H -22.4801 5.9027 -3.4608

C -23.4480 5.6155 -5.3593

H -23.6932 4.7886 -6.0300

H -22.7724 6.2846 -5.8997

C -24.7253 6.3708 -5.0015

H -25.4042 5.7005 -4.4702

H -24.4812 7.1927 -4.3249

C -25.4229 6.9211 -6.2392

H -24.7697 7.6128 -6.7683

H -26.3328 7.4496 -5.9644

H -25.6850 6.1138 -6.9196

C -14.1708 1.3772 3.7451

H -13.6411 2.3354 3.7790

H -13.7959 0.7445 4.5582

C -15.6725 1.5784 3.9584

H -15.7856 2.2491 4.8134

H -16.1167 0.6221 4.2247

C -16.4067 2.1432 2.7449

H -16.3748 1.4067 1.9428

H -15.9020 3.0433 2.3918

C -17.8630 2.4739 3.0622

H -18.3849 1.5623 3.3600

H -17.9017 3.1682 3.9066

C -18.5828 3.0946 1.8685

H -18.0751 4.0197 1.5858

H -18.5275 2.4140 1.0153

C -20.0473 3.4038 2.1724

H -20.5890 2.4710 2.3479

H -20.1048 3.9967 3.0893

C -20.7164 4.1771 1.0391

H -20.6369 3.6080 0.1106

H -20.1854 5.1212 0.8937

C -22.1849 4.4740 1.3318

H -22.7451 3.5383 1.3803

H -22.2632 4.9568 2.3100

C -22.8039 5.3884 0.2776

H -22.2514 6.3306 0.2583

H -22.7050 4.9276 -0.7082

C -24.2764 5.6813 0.5564

H -24.3770 6.0918 1.5645

H -24.8469 4.7504 0.5218

C -24.8600 6.6749 -0.4446

H -24.2897 7.6047 -0.3931

H -24.7528 6.2752 -1.4566

C -26.3275 6.9718 -0.1668

H -26.9233 6.0647 -0.2290

H -26.7171 7.6889 -0.8867

H -26.4470 7.3890 0.8313

O -13.3273 0.3041 -0.3104

O -10.9656 -0.0220 -1.0756

O -13.9501 0.7611 2.4861

C -11.0451 2.1395 -16.3562

C -11.4191 2.0799 -17.6847

H -10.6670 1.8828 -18.4298

C -12.7550 2.2399 -18.0258

C -13.7447 2.4208 -17.0402

C -13.3463 2.5268 -15.6950

C -11.9976 2.4034 -15.3862

H -11.6709 2.4707 -14.3628

C -12.2815 2.3488 -20.3539

H -11.6775 1.4303 -20.4219

H -11.6002 3.1904 -20.1596

C -13.0127 2.5682 -21.6757

H -13.7529 1.7765 -21.8013

H -12.2714 2.4731 -22.4723

C -13.7006 3.9317 -21.7841

H -13.0583 4.6976 -21.3430

H -13.8217 4.1769 -22.8427

C -15.0750 3.9673 -21.1181

H -14.9778 3.7123 -20.0627

H -15.7079 3.2067 -21.5832

C -15.7442 5.3322 -21.2766

H -15.1940 6.0810 -20.7007

H -15.7061 5.6331 -22.3271

C -17.2006 5.3103 -20.8205

H -17.2450 4.9915 -19.7772

H -17.7499 4.5740 -21.4132

C -17.8833 6.6699 -20.9550

H -17.3665 7.3991 -20.3265

H -17.8062 7.0137 -21.9889

C -19.3556 6.6033 -20.5537

H -19.4315 6.2428 -19.5260

H -19.8694 5.8828 -21.1940

C -20.0553 7.9562 -20.6593

H -19.5505 8.6776 -20.0129

H -19.9766 8.3241 -21.6849

C -21.5295 7.8685 -20.2673

H -22.0328 7.1488 -20.9176

H -21.6094 7.4998 -19.2427

C -22.2328 9.2192 -20.3720

H -22.1709 9.5768 -21.4017

H -21.7137 9.9447 -19.7415

C -23.6967 9.1400 -19.9558

H -24.2394 8.4491 -20.5967

H -24.1676 10.1185 -20.0307

H -23.7861 8.7921 -18.9289

C -16.1101 2.5881 -16.6543

H -16.0256 3.5100 -16.0724

H -16.1131 1.7375 -15.9649

C -17.4224 2.6184 -17.4295

H -17.5528 1.6853 -17.9766

H -17.3918 3.4301 -18.1574

C -18.5737 2.8286 -16.4485

H -18.6359 1.9667 -15.7794

H -18.3501 3.7036 -15.8332

C -19.9289 3.0453 -17.1159

H -20.2080 2.1613 -17.6905

H -19.8620 3.8839 -17.8124

C -21.0003 3.3404 -16.0676

H -21.0909 2.4841 -15.3944

H -20.6816 4.1961 -15.4685

C -22.3663 3.6492 -16.6747

H -22.7184 2.7865 -17.2440

H -22.2722 4.4855 -17.3722

C -23.3877 4.0028 -15.5964

H -23.4767 3.1664 -14.8979

H -23.0274 4.8657 -15.0321

C -24.7619 4.3245 -16.1753

H -25.1363 3.4586 -16.7257

H -24.6716 5.1496 -16.8860

C -25.7621 4.7028 -15.0867

H -25.8288 3.8904 -14.3598

H -25.4010 5.5907 -14.5600

C -27.1523 4.9869 -15.6491

H -27.5308 4.0903 -16.1459

H -27.0848 5.7769 -16.4013

C -28.1361 5.4127 -14.5627

H -28.2019 4.6253 -13.8075

H -27.7625 6.3146 -14.0718

C -29.5238 5.6860 -15.1293

H -29.4826 6.4820 -15.8695

H -30.2080 5.9865 -14.3385

H -29.9227 4.7940 -15.6081

C -13.8977 3.0167 -13.4021

H -13.3959 3.9919 -13.3780

H -13.2048 2.2474 -13.0417

C -15.1147 3.0243 -12.4722

H -14.7890 3.4558 -11.5233

H -15.4198 1.9970 -12.2769

C -16.3231 3.7930 -12.9952

H -16.6891 3.3036 -13.8990

H -16.0328 4.8104 -13.2568

C -17.4428 3.8323 -11.9558

H -17.6573 2.8173 -11.6151

H -17.1076 4.4046 -11.0878

C -18.7207 4.4556 -12.5100

H -18.5075 5.4699 -12.8517

H -19.0557 3.8760 -13.3751

C -19.8413 4.5045 -11.4745

H -20.0728 3.4915 -11.1368

H -19.5058 5.0736 -10.6055

C -21.1023 5.1557 -12.0363

H -21.4442 4.5902 -12.9082

H -20.8619 6.1676 -12.3710

C -22.2290 5.2268 -11.0104

H -22.5000 4.2163 -10.6957

H -21.8795 5.7636 -10.1259

C -23.4606 5.9327 -11.5733

H -23.1863 6.9483 -11.8679

H -23.7983 5.4067 -12.4696

C -24.6067 5.9953 -10.5676

H -24.2696 6.5101 -9.6654

H -24.8922 4.9805 -10.2820

C -25.8235 6.7205 -11.1372

H -25.5413 7.7430 -11.4027

H -26.1450 6.2196 -12.0532

C -26.9812 6.7562 -10.1479

H -27.2895 5.7451 -9.8873

H -27.8357 7.2740 -10.5772

H -26.6883 7.2711 -9.2368

O -15.0160 2.4867 -17.5437

O -13.2214 2.2365 -19.3096

O -14.3016 2.7401 -14.7318

C -4.7637 -3.9449 -14.7065

C -5.6689 -3.6785 -15.6617

C -5.4654 -3.8420 -17.0820

C -6.4482 -3.3831 -17.9884

C -4.3198 -4.4443 -17.6007

H -3.6020 -4.8350 -16.9027

C -5.5040 -3.5926 -22.2779

C -4.7301 -4.0444 -21.2777

C -5.0226 -3.9964 -19.8651

C -4.0925 -4.5599 -18.9603

C -6.1949 -3.4370 -19.3471

H -6.9155 -3.0365 -20.0390

O -3.0038 -5.1856 -19.4825

C -9.8072 -2.0218 -17.4119

C -9.6479 -1.7841 -16.0590

H -8.6913 -1.9892 -15.6139

C -10.6993 -1.2807 -15.3066

C -11.9467 -1.0268 -15.9093

C -12.0957 -1.2593 -17.2881

C -11.0263 -1.7694 -18.0156

H -11.1273 -1.9545 -19.0736

C -9.4394 -1.2807 -13.2730

H -8.5491 -1.0267 -13.8632

H -9.4342 -2.3602 -13.0690

C -9.3603 -0.4928 -11.9680

H -9.4149 0.5742 -12.2002

H -8.3717 -0.6969 -11.5499

C -10.4284 -0.8419 -10.9333

H -10.6099 -1.9139 -10.9415

H -10.0484 -0.5760 -9.9438

C -11.7508 -0.1193 -11.1691

H -12.1242 -0.3661 -12.1633

H -11.5815 0.9613 -11.1298

C -12.7872 -0.5039 -10.1139

H -13.0292 -1.5611 -10.2259

H -12.3587 -0.3587 -9.1181

C -14.0494 0.3415 -10.2477

H -14.4836 0.1676 -11.2339

H -13.7758 1.3982 -10.1907

C -15.1034 0.0514 -9.1811

H -15.3923 -1.0008 -9.2291

H -14.6824 0.2345 -8.1898

C -16.3334 0.9334 -9.3854

H -16.7465 0.7403 -10.3779

H -16.0287 1.9832 -9.3594

C -17.4282 0.7099 -8.3459

H -17.7204 -0.3432 -8.3449

H -17.0443 0.9491 -7.3518

C -18.6509 1.5762 -8.6434

H -18.3521 2.6271 -8.6654

H -19.0323 1.3262 -9.6367

C -19.7707 1.3949 -7.6223

H -19.4079 1.6834 -6.6334

H -20.0519 0.3395 -7.5773

C -20.9934 2.2313 -7.9792

H -20.7376 3.2874 -8.0112

H -21.7858 2.0897 -7.2472

H -21.3778 1.9468 -8.9560

C -14.2607 -0.8001 -15.3675

H -14.3753 -1.8652 -15.5926

H -14.5847 -0.2166 -16.2343

C -15.1098 -0.4277 -14.1608

H -15.0450 0.6495 -14.0217

H -14.7016 -0.9122 -13.2745

C -16.5691 -0.8273 -14.3755

H -16.8781 -0.5167 -15.3773

H -16.6717 -1.9138 -14.3248

C -17.4948 -0.1738 -13.3527

H -17.3582 0.9087 -13.4004

H -17.2167 -0.5018 -12.3491

C -18.9669 -0.4928 -13.6041

H -19.1990 -0.3127 -14.6568

H -19.1576 -1.5493 -13.4041

C -19.8818 0.3723 -12.7402

H -19.6612 1.4248 -12.9353

H -19.6718 0.1810 -11.6847

C -21.3614 0.1172 -13.0157

H -21.5487 0.2047 -14.0897

H -21.6191 -0.9023 -12.7232

C -22.2555 1.1056 -12.2709

H -21.9975 2.1221 -12.5765

H -22.0661 1.0268 -11.1968

C -23.7377 0.8613 -12.5403

H -23.9130 0.8740 -13.6194

H -24.0138 -0.1323 -12.1784

C -24.6239 1.9096 -11.8726

H -24.3514 2.9010 -12.2426

H -24.4450 1.8991 -10.7944

C -26.1073 1.6645 -12.1370

H -26.2894 1.6747 -13.2152

H -26.3817 0.6735 -11.7690

C -26.9839 2.7146 -11.4653

H -26.8396 2.6968 -10.3872

H -28.0348 2.5283 -11.6723

H -26.7321 3.7097 -11.8272

C -13.5031 -1.2153 -19.2353

H -13.4376 -2.2970 -19.4274

H -12.7410 -0.7046 -19.8389

C -14.8827 -0.6886 -19.6221

H -15.0063 -0.8218 -20.6993

H -14.9144 0.3793 -19.3972

C -16.0072 -1.4023 -18.8769

H -15.8956 -1.1982 -17.8116

H -15.9173 -2.4813 -19.0169

C -17.3905 -0.9425 -19.3377

H -17.3681 0.1362 -19.5076

H -17.6397 -1.4157 -20.2901

C -18.4701 -1.2620 -18.3056

H -18.4819 -2.3354 -18.1035

H -18.2202 -0.7558 -17.3686

C -19.8569 -0.8087 -18.7564

H -19.7927 0.2148 -19.1342

H -20.1988 -1.4397 -19.5803

C -20.8773 -0.8540 -17.6201

H -20.5403 -0.1959 -16.8149

H -20.9308 -1.8672 -17.2153

C -22.2660 -0.4126 -18.0762

H -22.1850 0.5484 -18.5919

H -22.6593 -1.1358 -18.7935

C -23.2406 -0.2698 -16.9091

H -23.2993 -1.2149 -16.3651

H -22.8612 0.4859 -16.2173

C -24.6378 0.1358 -17.3747

H -25.0506 -0.6529 -18.0090

H -24.5670 1.0420 -17.9822

C -25.5845 0.3922 -16.2048

H -25.6349 -0.5017 -15.5771

H -25.1838 1.2037 -15.5942

C -26.9859 0.7629 -16.6729

H -26.9558 1.6533 -17.2977

H -27.6345 0.9615 -15.8226

H -27.4218 -0.0458 -17.2555

O -12.9010 -0.5261 -15.0759

O -10.6157 -0.9505 -13.9895

O -13.2982 -0.9548 -17.8630

O -7.5954 -2.9136 -17.4287

C -4.9637 -3.8059 -13.2835

C -3.9255 -4.1908 -12.4249

C -6.1373 -3.3307 -12.6887

C -4.0999 -4.0776 -11.0633

H -3.0014 -4.5758 -12.8307

C -6.2120 -3.2609 -11.3126

H -6.9794 -3.0231 -13.2851

H -3.3233 -4.3589 -10.3645

H -7.0973 -2.9042 -10.8022

C -5.1921 -3.6802 -23.6890

C -6.1717 -3.3378 -24.6271

C -3.9570 -4.0963 -24.1949

C -5.8873 -3.4424 -25.9755

H -7.1448 -3.0027 -24.3022

C -3.7755 -4.1536 -25.5637

H -3.1460 -4.3626 -23.5358

H -6.6271 -3.1917 -26.7247

H -2.8318 -4.4689 -25.9893

C -2.1920 -5.9182 -18.5864

H -1.7719 -5.2473 -17.8228

H -2.8048 -6.6778 -18.0763

C -8.6556 -2.5003 -18.2598

H -8.3262 -1.6733 -18.9092

H -8.9803 -3.3335 -18.9006

C -6.9996 -4.0992 11.5616

C -8.2103 -3.7662 6.0486

C -8.2553 -3.7361 4.8398

C -8.0906 -3.7294 3.4450

C -9.1210 -3.4370 2.5340

C -6.8071 -4.0416 2.9847

H -6.0767 -4.2932 3.7369

O -5.8267 -4.1281 11.9115

O -8.0079 -4.1469 12.3938

C -6.6047 -3.2697 -7.4937

C -7.1952 -3.5193 -1.8595

C -7.2715 -3.6016 -0.6557

C -7.5070 -3.6726 0.7302

C -6.4927 -4.0331 1.6391

C -8.7990 -3.4047 1.1867

H -9.5389 -3.1673 0.4393

O -7.5269 -3.0854 -8.2611

O -5.3661 -3.4858 -7.8926

O -5.2767 -4.3561 1.1216

C -12.7345 -2.7365 2.7434

C -12.8893 -2.4539 4.0926

H -12.0309 -2.5463 4.7382

C -14.1200 -2.0364 4.5744

C -15.2477 -1.9726 3.7190

C -15.0707 -2.2438 2.3600

C -13.8151 -2.6171 1.8938

H -13.6792 -2.7741 0.8345

C -13.4168 -1.7818 6.8683

H -12.4094 -1.5437 6.5059

H -13.4182 -2.8258 7.2078

C -13.7481 -0.8513 8.0419

H -13.3780 0.1517 7.8212

H -13.1983 -1.2283 8.9060

C -15.2426 -0.7554 8.3727

H -15.7243 -1.7132 8.1748

H -15.3548 -0.5373 9.4369

C -15.9322 0.3396 7.5611

H -15.8678 0.0802 6.5062

H -15.3979 1.2815 7.7147

C -17.3980 0.5431 7.9385

H -17.9635 -0.3603 7.7034

H -17.4842 0.7189 9.0131

C -17.9896 1.7302 7.1784

H -17.8173 1.5832 6.1103

H -17.4603 2.6404 7.4696

C -19.4856 1.9245 7.4156

H -20.0157 1.0016 7.1682

H -19.6663 2.1354 8.4717

C -20.0355 3.0672 6.5639

H -19.8479 2.8464 5.5097

H -19.5001 3.9869 6.8073

C -21.5328 3.2911 6.7646

H -22.0692 2.3629 6.5541

H -21.7241 3.5530 7.8074

C -22.0655 4.3986 5.8584

H -21.5245 5.3248 6.0638

H -21.8771 4.1292 4.8154

C -23.5613 4.6378 6.0482

H -23.7526 4.9214 7.0864

H -24.1008 3.7091 5.8538

C -24.0843 5.7299 5.1238

H -23.5840 6.6751 5.3235

H -25.1534 5.8706 5.2617

H -23.9086 5.4634 4.0829

C -17.6234 -1.6447 3.6312

H -17.8243 -2.6647 3.2777

H -17.5598 -0.9826 2.7620

C -18.7680 -1.1757 4.5229

H -18.5199 -0.1911 4.9209

H -18.8869 -1.8541 5.3678

C -20.0531 -1.0996 3.6975

H -19.8102 -0.6901 2.7138

H -20.4487 -2.1063 3.5401

C -21.1333 -0.2184 4.3205

H -20.7109 0.7667 4.5287

H -21.4652 -0.6433 5.2699

C -22.3239 -0.0591 3.3754

H -21.9527 0.2070 2.3817

H -22.8485 -1.0125 3.2836

C -23.3008 1.0211 3.8344

H -22.7627 1.9660 3.9371

H -23.7038 0.7596 4.8152

C -24.4480 1.2078 2.8438

H -24.0355 1.3840 1.8468

H -25.0417 0.2917 2.7964

C -25.3485 2.3810 3.2233

H -24.7513 3.2944 3.2526

H -25.7527 2.2182 4.2255

C -26.5013 2.5699 2.2407

H -26.0986 2.6962 1.2329

H -27.1252 1.6738 2.2356

C -27.3573 3.7834 2.5948

H -26.7371 4.6819 2.5679

H -27.7358 3.6727 3.6138

C -28.5345 3.9561 1.6379

H -28.1579 4.0548 0.6177

H -29.1626 3.0634 1.6719

C -29.3737 5.1800 1.9852

H -29.7764 5.0928 2.9926

H -30.2057 5.2843 1.2918

H -28.7700 6.0828 1.9344

C -16.4604 -3.1531 0.6347

H -17.0212 -3.8853 1.2382

H -15.5744 -3.6554 0.2312

C -17.3328 -2.6387 -0.5068

H -17.6212 -3.4880 -1.1288

H -16.7452 -1.9498 -1.1149

C -18.5764 -1.9206 0.0106

H -18.2561 -1.0536 0.5899

H -19.1248 -2.5839 0.6841

C -19.5028 -1.4606 -1.1108

H -18.9370 -0.8665 -1.8314

H -19.8978 -2.3312 -1.6392

C -20.6621 -0.6261 -0.5706

H -21.2032 -1.2029 0.1843

H -20.2625 0.2645 -0.0768

C -21.6316 -0.2004 -1.6705

H -21.0800 0.3128 -2.4613

H -22.0874 -1.0898 -2.1123

C -22.7284 0.7201 -1.1430

H -22.2728 1.6265 -0.7319

H -23.2591 0.2212 -0.3279

C -23.7260 1.1051 -2.2335

H -23.1895 1.5507 -3.0743

H -24.2205 0.2033 -2.6015

C -24.7782 2.0884 -1.7290

H -25.2959 1.6525 -0.8710

H -24.2842 3.0010 -1.3868

C -25.8001 2.4399 -2.8070

H -26.3196 1.5319 -3.1222

H -25.2825 2.8411 -3.6816

C -26.8227 3.4616 -2.3149

H -27.3294 3.0628 -1.4324

H -26.3022 4.3737 -2.0167

C -27.8580 3.7955 -3.3813

H -27.3765 4.1963 -4.2708

H -28.5644 4.5353 -3.0110

H -28.4124 2.9046 -3.6672

O -16.4040 -1.6068 4.3462

O -14.3775 -1.6276 5.8444

O -16.0961 -2.0555 1.4545

O -10.3669 -3.2220 3.0459

C -7.9844 -3.8419 7.4339

C -8.9809 -3.6372 8.3889

C -6.6742 -4.1242 7.8432

C -8.6774 -3.7276 9.7310

H -9.9899 -3.4165 8.0720

C -6.3741 -4.1983 9.1806

H -5.9110 -4.2745 7.0937

C -7.3742 -4.0133 10.1353

H -9.4412 -3.5668 10.4756

H -5.3716 -4.4034 9.5207

C -7.0552 -3.4480 -3.2584

C -8.1702 -3.2397 -4.0777

C -5.7918 -3.5840 -3.8371

C -8.0167 -3.1640 -5.4445

H -9.1460 -3.1465 -3.6263

C -5.6451 -3.5107 -5.2030

H -4.9392 -3.7570 -3.2006

C -6.7552 -3.3037 -6.0172

H -8.8638 -2.9982 -6.0921

H -4.6728 -3.6144 -5.6574

C -4.2853 -4.7913 2.0273

H -4.1637 -4.0449 2.8257

H -4.5983 -5.7336 2.5010

C -11.4146 -3.0844 2.1096

H -11.1809 -2.2791 1.4018

H -11.5196 -4.0233 1.5415

N -5.2160 -3.6194 -10.5167

N -4.7152 -3.8431 -26.4432

H -5.3156 -3.5009 -8.9068

C -1.0459 -6.5950 -19.2882

C -0.9950 -6.7734 -20.6600

H -1.8060 -6.4041 -21.2635

C 0.0932 -7.4270 -21.2295

C 1.1504 -7.9040 -20.4283

C 1.1164 -7.6589 -19.0436

C -0.0062 -7.0461 -18.4947

H -0.0676 -6.8914 -17.4313

C -0.8602 -7.4030 -23.4075

H -1.1636 -6.3483 -23.3431

H -1.7050 -8.0256 -23.0847

C -0.5149 -7.7220 -24.8600

H 0.2344 -7.0126 -25.2146

H -1.4285 -7.5642 -25.4378

C 0.0037 -9.1463 -25.0767

H -0.5043 -9.8296 -24.3954

H -0.2353 -9.4585 -26.0967

C 1.5142 -9.2449 -24.8724

H 1.7677 -8.8668 -23.8824

H 2.0054 -8.6101 -25.6155

C 2.0337 -10.6745 -25.0241

H 1.7714 -11.2517 -24.1349

H 1.5559 -11.1545 -25.8819

C 3.5487 -10.6941 -25.2187

H 4.0111 -10.0702 -24.4518

H 3.7891 -10.2563 -26.1900

C 4.1447 -12.0980 -25.1379

H 4.0006 -12.4903 -24.1288

H 3.6189 -12.7604 -25.8295

C 5.6349 -12.0952 -25.4712

H 6.1340 -11.3369 -24.8633

H 5.7698 -11.8153 -26.5190

C 6.2961 -13.4474 -25.2176

H 6.2011 -13.6979 -24.1583

H 5.7753 -14.2197 -25.7887

C 7.7734 -13.4418 -25.6029

H 7.8676 -13.2506 -26.6740

H 8.2804 -12.6275 -25.0791

C 8.4668 -14.7588 -25.2616

H 7.9606 -15.5785 -25.7765

H 8.3836 -14.9383 -24.1873

C 9.9375 -14.7422 -25.6588

H 10.0433 -14.6102 -26.7328

H 10.4219 -15.6745 -25.3771

H 10.4568 -13.9232 -25.1639

C 3.1591 -9.2146 -20.4236

H 2.7511 -9.7985 -19.5946

H 3.8543 -8.4679 -20.0216

C 3.9212 -10.1378 -21.3676

H 4.2320 -9.5733 -22.2459

H 3.2696 -10.9485 -21.6974

C 5.1414 -10.6945 -20.6394

H 5.7935 -9.8595 -20.3662

H 4.8164 -11.1721 -19.7118

C 5.9469 -11.6914 -21.4664

H 6.2082 -11.2394 -22.4245

H 5.3414 -12.5776 -21.6693

C 7.2249 -12.0954 -20.7349

H 7.8072 -11.1945 -20.5238

H 6.9642 -12.5497 -19.7758

C 8.0888 -13.0634 -21.5354

H 8.2806 -12.6437 -22.5250

H 7.5515 -14.0048 -21.6731

C 9.4203 -13.3325 -20.8388

H 9.9537 -12.3859 -20.7151

H 9.2305 -13.7395 -19.8420

C 10.3036 -14.3014 -21.6183

H 10.4831 -13.9032 -22.6193

H 9.7846 -15.2568 -21.7322

C 11.6407 -14.5338 -20.9202

H 12.1616 -13.5787 -20.8194

H 11.4579 -14.9199 -19.9140

C 12.5335 -15.5104 -21.6788

H 12.7154 -15.1281 -22.6860

H 12.0189 -16.4691 -21.7787

C 13.8702 -15.7274 -20.9734

H 14.3847 -14.7687 -20.8745

H 13.6885 -16.1087 -19.9662

C 14.7620 -16.7038 -21.7300

H 14.2749 -17.6722 -21.8210

H 15.7077 -16.8426 -21.2110

H 14.9702 -16.3320 -22.7314

C 2.2094 -7.6785 -16.9034

H 1.5106 -8.3499 -16.3881

H 1.8975 -6.6387 -16.7332

C 3.6257 -7.8616 -16.3614

H 3.5754 -7.7726 -15.2740

H 4.2520 -7.0507 -16.7367

C 4.2656 -9.1929 -16.7436

H 4.3197 -9.2572 -17.8286

H 3.6431 -10.0217 -16.4036

C 5.6735 -9.3078 -16.1626

H 6.2387 -8.4091 -16.4236

H 5.6202 -9.3569 -15.0723

C 6.4229 -10.5272 -16.6930

H 5.9019 -11.4391 -16.3911

H 6.4232 -10.4974 -17.7847

C 7.8659 -10.5669 -16.1939

H 8.3608 -9.6309 -16.4644

H 7.8723 -10.6415 -15.1039

C 8.6523 -11.7335 -16.7864

H 8.6157 -11.6733 -17.8777

H 8.1836 -12.6762 -16.4950

C 10.1103 -11.7268 -16.3326

H 10.5653 -10.7729 -16.6079

H 10.1497 -11.8102 -15.2437

C 10.9151 -12.8634 -16.9567

H 10.4745 -13.8218 -16.6704

H 10.8570 -12.7899 -18.0458

C 12.3804 -12.8303 -16.5275

H 12.4404 -12.9350 -15.4418

H 12.8100 -11.8623 -16.7916

C 13.2016 -13.9362 -17.1861

H 12.7835 -14.9081 -16.9140

H 13.1316 -13.8394 -18.2718

C 14.6662 -13.8801 -16.7725

H 15.1027 -12.9218 -17.0488

H 15.2333 -14.6683 -17.2627

H 14.7669 -14.0036 -15.6961

O 2.1211 -8.5577 -21.1261

O 0.2419 -7.6528 -22.5639

O 2.2099 -7.9742 -18.2879

C -2.9480 -4.9835 1.3539

C -2.7604 -4.6819 0.0175

C -1.4976 -4.8332 -0.5554

C -0.4220 -5.2722 0.2294

C -0.6267 -5.6069 1.5783

C -1.8958 -5.4598 2.1271

H -2.0727 -5.7090 3.1613

C -2.2768 -4.4696 -2.7751

H -2.8905 -3.5980 -2.5158

H -2.9075 -5.3690 -2.7322

C -1.7340 -4.2903 -4.1890

H -1.2058 -3.3375 -4.2522

H -2.6014 -4.2398 -4.8483

C -0.7979 -5.4164 -4.6418

H -1.0713 -6.3439 -4.1348

H -0.9301 -5.5774 -5.7134

C 0.6725 -5.0963 -4.3743

H 0.8131 -4.9084 -3.3102

H 0.9333 -4.1827 -4.9164

C 1.5924 -6.2256 -4.8341

H 1.4934 -7.0738 -4.1528

H 1.2817 -6.5634 -5.8266

C 3.0570 -5.7963 -4.8980

H 3.3531 -5.3683 -3.9372

H 3.1722 -5.0176 -5.6554

C 3.9737 -6.9710 -5.2315

H 3.8731 -7.7319 -4.4539

H 3.6550 -7.4207 -6.1747

C 5.4413 -6.5633 -5.3408

H 5.7442 -6.0499 -4.4247

H 5.5641 -5.8604 -6.1683

C 6.3430 -7.7763 -5.5589

H 6.1941 -8.4812 -4.7374

H 6.0497 -8.2838 -6.4819

C 7.8207 -7.4044 -5.6356

H 7.9820 -6.7380 -6.4866

H 8.1034 -6.8592 -4.7321

C 8.7121 -8.6356 -5.7800

H 8.4436 -9.1727 -6.6924

H 8.5310 -9.3075 -4.9381

C 10.1908 -8.2685 -5.8248

H 10.4017 -7.6313 -6.6802

H 10.8057 -9.1627 -5.9025

H 10.4774 -7.7325 -4.9214

C 1.3337 -6.6398 -0.5881

H 0.8072 -7.0825 -1.4454

H 1.1765 -7.2889 0.2792

C 2.8189 -6.4893 -0.8852

H 3.2796 -5.9639 -0.0457

H 2.9328 -5.8575 -1.7667

C 3.5243 -7.8233 -1.1033

H 3.3505 -8.4735 -0.2431

H 3.1045 -8.3230 -1.9793

C 5.0244 -7.6111 -1.3004

H 5.4231 -7.0661 -0.4418

H 5.1844 -6.9892 -2.1831

C 5.7996 -8.9163 -1.4600

H 5.6238 -9.5528 -0.5896

H 5.4343 -9.4546 -2.3375

C 7.2979 -8.6561 -1.6084

H 7.6534 -8.0979 -0.7386

H 7.4687 -8.0316 -2.4870

C 8.1072 -9.9430 -1.7417

H 7.9326 -10.5747 -0.8682

H 7.7647 -10.4980 -2.6194

C 9.6025 -9.6608 -1.8738

H 9.9447 -9.1026 -0.9990

H 9.7735 -9.0312 -2.7503

C 10.4239 -10.9402 -2.0089

H 10.2684 -11.5653 -1.1253

H 10.0718 -11.5055 -2.8749

C 11.9140 -10.6492 -2.1699

H 12.2632 -10.0573 -1.3206

H 12.0680 -10.0522 -3.0720

C 12.7429 -11.9279 -2.2660

H 12.6063 -12.5168 -1.3559

H 12.3814 -12.5284 -3.1045

C 14.2252 -11.6304 -2.4599

H 14.3842 -11.0683 -3.3775

H 14.7973 -12.5537 -2.5200

H 14.6094 -11.0424 -1.6296

C 0.2978 -6.6760 3.5096

H -0.3597 -7.5438 3.3860

H -0.1584 -5.9814 4.2306

C 1.6596 -7.1102 4.0478

H 1.4807 -7.6483 4.9817

H 2.2369 -6.2178 4.2848

C 2.4607 -7.9817 3.0816

H 2.6454 -7.4196 2.1671

H 1.8924 -8.8705 2.8077

C 3.7952 -8.3932 3.7001

H 4.3304 -7.4973 4.0231

H 3.6099 -9.0051 4.5869

C 4.6825 -9.1700 2.7304

H 4.1522 -10.0584 2.3795

H 4.8905 -8.5492 1.8558

C 6.0028 -9.5766 3.3829

H 6.5033 -8.6804 3.7569

H 5.7984 -10.2216 4.2422

C 6.9427 -10.3012 2.4221

H 7.1354 -9.6626 1.5567

H 6.4604 -11.2122 2.0589

C 8.2699 -10.6572 3.0884

H 8.7405 -9.7436 3.4586

H 8.0807 -11.3028 3.9487

C 9.2315 -11.3593 2.1329

H 8.7657 -12.2740 1.7567

H 9.4253 -10.7118 1.2747

C 10.5564 -11.7078 2.8073

H 10.3660 -12.3540 3.6676

H 11.0224 -10.7920 3.1795

C 11.5236 -12.4081 1.8559

H 11.0578 -13.3186 1.4728

H 11.7246 -11.7563 1.0027

C 12.8391 -12.7617 2.5392

H 13.3317 -11.8635 2.9062

H 13.5117 -13.2593 1.8445

H 12.6652 -13.4250 3.3832

O 0.8254 -5.3325 -0.3412

O -1.2062 -4.5928 -1.8586

O 0.4683 -6.0406 2.2562

H -7.7087 -4.2079 13.3554

H -3.7585 -1.4161 1.1331

H -6.6786 -0.5063 1.0253

H -4.7026 -1.4916 7.5386

H -7.5937 -0.5147 7.7029

H -3.7822 -4.3105 -14.9768

H -6.6344 -3.2848 -15.3869

H -3.7836 -4.5102 -21.5095

H -6.4629 -3.1400 -22.0632

H -3.6042 -4.3382 -0.5564

C -3.8427 4.9790 8.4501

C -2.8910 4.5935 7.5843

C -2.9012 4.7339 6.1472

C -1.7222 4.4340 5.4270

C -4.0161 5.1708 5.4334

H -4.9049 5.4000 5.9939

C -1.8633 4.7064 1.0604

C -2.8477 5.0755 1.8959

C -2.8396 4.9654 3.3344

C -4.0109 5.2932 4.0541

C -1.7139 4.5567 4.0517

H -0.8129 4.3440 3.5015

O -5.0815 5.7045 3.3229

C 1.5973 3.3863 6.6164

C 1.1260 2.7359 7.7423

H 0.0705 2.7469 7.9492

C 2.0103 2.0752 8.5865

C 3.3832 2.0385 8.2875

C 3.8514 2.6718 7.1223

C 2.9520 3.3764 6.3257

H 3.2978 3.9068 5.4539

C 0.3701 1.6220 10.2661

H -0.4296 1.3342 9.5668

H 0.2662 2.6927 10.4939

C 0.2288 0.8014 11.5488

H 0.1995 -0.2588 11.2930

H -0.7304 1.0686 11.9958

C 1.3634 1.0421 12.5508

H 1.6663 2.0892 12.5078

H 0.9947 0.8471 13.5606

C 2.5706 0.1470 12.2748

H 2.8675 0.2609 11.2328

H 2.2833 -0.8963 12.4320

C 3.7652 0.4769 13.1662

H 4.0673 1.5129 12.9890

H 3.4839 0.3941 14.2188

C 4.9409 -0.4515 12.8697

H 5.0982 -0.4838 11.7893

H 4.6891 -1.4636 13.1934

C 6.2346 -0.0093 13.5478

H 6.4818 1.0049 13.2232

H 6.0929 0.0192 14.6302

C 7.3956 -0.9428 13.2082

H 7.4718 -1.0349 12.1228

H 7.1873 -1.9367 13.6112

C 8.7288 -0.4454 13.7612

H 8.9331 0.5533 13.3653

H 8.6635 -0.3591 14.8485

C 9.8808 -1.3774 13.3936

H 9.6907 -2.3686 13.8127

H 9.9252 -1.4784 12.3070

C 11.2268 -0.8690 13.9038

H 11.1823 -0.7557 14.9895

H 11.4233 0.1187 13.4787

C 12.3669 -1.8126 13.5390

H 12.2024 -2.7957 13.9742

H 13.3158 -1.4299 13.9098

H 12.4407 -1.9237 12.4593

C 5.3842 1.8340 9.6279

H 5.2814 1.8964 10.7178

H 5.5588 2.8377 9.2370

C 6.5398 0.9083 9.2624

H 6.6444 0.9185 8.1765

H 6.2720 -0.1059 9.5680

C 7.8735 1.2986 9.8918

H 8.1525 2.3042 9.5710

H 7.7841 1.3095 10.9800

C 8.9540 0.3042 9.4722

H 8.9683 0.2413 8.3801

H 8.6927 -0.6880 9.8501

C 10.3534 0.6697 9.9562

H 10.6358 1.6469 9.5601

H 10.3604 0.7400 11.0454

C 11.3685 -0.3796 9.5047

H 11.3204 -0.4760 8.4161

H 11.0925 -1.3474 9.9300

C 12.8011 -0.0464 9.9094

H 13.0926 0.9088 9.4669

H 12.8568 0.0648 10.9942

C 13.7742 -1.1340 9.4592

H 13.7191 -1.2381 8.3725

H 13.4730 -2.0909 9.8947

C 15.2139 -0.8272 9.8625

H 15.5106 0.1326 9.4351

H 15.2713 -0.7350 10.9500

C 16.1852 -1.9091 9.3964

H 16.1290 -2.0005 8.3089

H 15.8892 -2.8701 9.8238

C 17.6244 -1.6001 9.8017

H 17.9209 -0.6372 9.3783

H 17.6794 -1.5094 10.8893

C 18.5933 -2.6793 9.3347

H 18.3219 -3.6450 9.7560

H 19.6090 -2.4431 9.6464

H 18.5812 -2.7627 8.2503

C 5.6286 2.8702 5.5271

H 5.9838 3.9076 5.5110

H 4.8119 2.7491 4.8093

C 6.7378 1.9099 5.1171

H 6.8272 1.9771 4.0330

H 6.4141 0.8986 5.3627

C 8.0936 2.1983 5.7500

H 8.0171 2.1424 6.8372

H 8.3998 3.2139 5.4914

C 9.1242 1.1892 5.2491

H 8.8156 0.1848 5.5498

H 9.1387 1.2137 4.1563

C 10.5367 1.4495 5.7626

H 10.8640 2.4415 5.4427

H 10.5395 1.4374 6.8545

C 11.5119 0.3974 5.2400

H 11.1932 -0.5889 5.5850

H 11.4780 0.3911 4.1477

C 12.9471 0.6459 5.6924

H 12.9831 0.6784 6.7840

H 13.2810 1.6187 5.3222

C 13.8926 -0.4405 5.1882

H 13.5650 -1.4089 5.5755

H 13.8393 -0.4853 4.0974

C 15.3376 -0.1938 5.6096

H 15.6771 0.7597 5.1971

H 15.3884 -0.1165 6.6989

C 16.2669 -1.3099 5.1410

H 16.2046 -1.3968 4.0535

H 15.9327 -2.2587 5.5685

C 17.7190 -1.0635 5.5413

H 18.0641 -0.1292 5.0906

H 17.7822 -0.9469 6.6253

C 18.6269 -2.2047 5.1003

H 18.3011 -3.1445 5.5430

H 19.6550 -2.0207 5.4073

H 18.6063 -2.3109 4.0186

O 4.1620 1.2929 9.1324

O 1.6403 1.3845 9.6959

O 5.1742 2.5450 6.8349

O -0.6573 4.0116 6.1700

C -3.7690 4.8461 9.8903

C -4.7864 5.3840 10.6873

C -2.7263 4.2008 10.5640

C -4.7090 5.2676 12.0618

H -5.6214 5.8934 10.2309

C -2.7463 4.1415 11.9442

H -1.9061 3.7604 10.0233

H -5.4747 5.6813 12.7041

H -1.9518 3.6556 12.4950

C -1.8724 4.8342 -0.3788

C -0.7087 4.5132 -1.0874

C -2.9680 5.2621 -1.1368

C -0.6985 4.6046 -2.4601

H 0.1774 4.2013 -0.5609

C -2.8551 5.3271 -2.5118

H -3.8983 5.5293 -0.6628

H 0.1797 4.3483 -3.0370

H -3.6712 5.6538 -3.1420

C -6.2433 6.0900 4.0224

H -6.5960 5.2516 4.6406

H -6.0137 6.9424 4.6794

C 0.6487 4.0457 5.6420

H 0.7168 3.4981 4.6898

H 0.9461 5.0882 5.4478

C -2.7329 5.6028 -6.2720

C -2.9945 6.0384 -11.9195

C -3.1677 6.1695 -13.1098

C -3.4985 6.3570 -14.4646

C -2.6940 5.8642 -15.5091

C -4.6711 7.0572 -14.7565

H -5.2207 7.4525 -13.9172

O -3.5871 6.0754 -5.5539

O -1.6789 4.9550 -5.8084

C -4.1934 5.9589 -25.2535

C -4.7402 6.6166 -19.6597

C -4.6754 6.7258 -18.4559

C -4.3464 6.6825 -17.0900

C -5.1241 7.2318 -16.0566

C -3.1425 6.0356 -16.8032

H -2.5917 5.6628 -17.6512

O -3.0953 5.7013 -25.7261

O -5.2701 6.1453 -25.9730

H -5.0808 6.1041 -26.9635

O -6.2603 7.8740 -16.4163

C 0.5736 4.2147 -15.8450

C 0.7892 3.8239 -14.5374

H 0.0500 4.0838 -13.7983

C 1.9493 3.1222 -14.2145

C 2.8812 2.8237 -15.2210

C 2.6394 3.1953 -16.5519

C 1.4857 3.9105 -16.8476

H 1.2853 4.2325 -17.8581

C 1.5657 3.0281 -11.8361

H 0.4905 3.1076 -12.0371

H 1.9291 4.0099 -11.5000

C 1.7766 1.9829 -10.7393

H 1.3063 1.0487 -11.0518

H 1.2496 2.3474 -9.8554

C 3.2408 1.7050 -10.3907

H 3.8097 2.6344 -10.4342

H 3.2838 1.3389 -9.3620

C 3.8913 0.6618 -11.3006

H 3.9494 1.0468 -12.3179

H 3.2662 -0.2349 -11.3167

C 5.2828 0.2796 -10.8007

H 5.9201 1.1674 -10.7907

H 5.2072 -0.0798 -9.7710

C 5.9463 -0.8004 -11.6521

H 6.0378 -0.4437 -12.6798

H 5.3154 -1.6916 -11.6671

C 7.3281 -1.1634 -11.1129

H 7.9287 -0.2535 -11.0366

H 7.2228 -1.5740 -10.1060

C 8.0716 -2.1705 -11.9871

H 8.1917 -1.7583 -12.9919

H 7.4837 -3.0874 -12.0724

C 9.4460 -2.4954 -11.4073

H 10.0102 -1.5662 -11.2918

H 9.3214 -2.9311 -10.4128

C 10.2496 -3.4573 -12.2778

H 9.6998 -4.3952 -12.3864

H 10.3731 -3.0264 -13.2744

C 11.6253 -3.7426 -11.6794

H 11.5016 -4.1612 -10.6787

H 12.1726 -2.8019 -11.5798

C 12.4366 -4.7065 -12.5350

H 11.9258 -5.6622 -12.6248

H 13.4144 -4.8827 -12.0924

H 12.5839 -4.2967 -13.5318

C 5.2297 2.8864 -14.9824

H 5.0947 3.8939 -14.5715

H 5.4951 2.9803 -16.0428

C 6.3042 2.1100 -14.2286

H 6.0374 1.0564 -14.3226

H 6.2709 2.3660 -13.1697

C 7.7120 2.3134 -14.7770

H 7.6985 2.1810 -15.8622

H 8.0553 3.3287 -14.5695

C 8.6760 1.2982 -14.1680

H 8.2514 0.2989 -14.2894

H 8.7750 1.4836 -13.0970

C 10.0558 1.3197 -14.8182

H 9.9459 1.1967 -15.8993

H 10.5340 2.2848 -14.6412

C 10.9401 0.1990 -14.2778

H 10.4360 -0.7582 -14.4346

H 11.0686 0.3269 -13.2006

C 12.3090 0.1576 -14.9489

H 12.1779 0.0623 -16.0296

H 12.8326 1.0974 -14.7613

C 13.1541 -1.0067 -14.4412

H 12.6280 -1.9437 -14.6378

H 13.2766 -0.9216 -13.3587

C 14.5298 -1.0540 -15.1005

H 14.4066 -1.1121 -16.1856

H 15.0665 -0.1291 -14.8790

C 15.3552 -2.2451 -14.6212

H 14.8093 -3.1701 -14.8246

H 15.4924 -2.1758 -13.5399

C 16.7212 -2.3114 -15.2997

H 16.5849 -2.3942 -16.3802

H 17.2673 -1.3852 -15.1073

C 17.5425 -3.4936 -14.8003

H 17.7201 -3.4101 -13.7308

H 18.5064 -3.5328 -15.3049

H 17.0181 -4.4280 -14.9869

C 3.4032 3.1758 -18.8236

H 3.4884 4.2670 -18.9082

H 2.4128 2.8737 -19.1872

C 4.4768 2.4897 -19.6727

H 4.5231 3.0103 -20.6320

H 4.1733 1.4597 -19.8614

C 5.8476 2.4861 -19.0011

H 5.7549 1.9602 -18.0499

H 6.1532 3.5112 -18.7904

C 6.9032 1.7848 -19.8514

H 6.5206 0.8081 -20.1524

H 7.0884 2.3621 -20.7597

C 8.2156 1.5820 -19.0968

H 8.6384 2.5506 -18.8239

H 8.0106 1.0379 -18.1711

C 9.2317 0.7878 -19.9146

H 8.7691 -0.1448 -20.2441

H 9.5044 1.3564 -20.8059

C 10.4925 0.4579 -19.1180

H 10.2129 -0.0969 -18.2185

H 10.9771 1.3834 -18.7996

C 11.4759 -0.3790 -19.9334

H 10.9747 -1.2921 -20.2656

H 11.7722 0.1809 -20.8228

C 12.7229 -0.7580 -19.1396

H 13.2415 0.1502 -18.8214

H 12.4243 -1.3021 -18.2399

C 13.6769 -1.6280 -19.9545

H 13.9807 -1.0882 -20.8542

H 13.1520 -2.5326 -20.2726

C 14.9217 -2.0249 -19.1659

H 15.4605 -1.1223 -18.8628

H 14.6182 -2.5505 -18.2580

C 15.8469 -2.9209 -19.9783

H 15.3315 -3.8334 -20.2715

H 16.7257 -3.1949 -19.3993

H 16.1777 -2.4102 -20.8804

O 4.0201 2.1407 -14.8578

O 2.2796 2.6439 -12.9921

O 3.5624 2.7977 -17.4659

O -1.5162 5.2589 -15.1817

C -2.8724 5.9368 -10.5197

C -1.8410 5.2141 -9.9172

C -3.8330 6.5670 -9.7203

C -1.7865 5.1052 -8.5436

H -1.0941 4.7388 -10.5365

C -3.7788 6.4480 -8.3508

H -4.6086 7.1489 -10.1926

C -2.7603 5.7099 -7.7526

H -0.9987 4.5440 -8.0670

H -4.5141 6.9204 -7.7186

C -4.6746 6.4525 -21.0542

C -3.4688 5.9738 -21.5860

C -5.7390 6.7439 -21.9112

C -3.3295 5.8123 -22.9434

H -2.6549 5.7458 -20.9154

C -5.5981 6.5677 -23.2708

H -6.6686 7.1105 -21.4977

C -4.3918 6.1083 -23.7963

H -2.4066 5.4569 -23.3737

H -6.4122 6.7907 -23.9435

C -6.9728 8.5686 -15.4157

H -7.1149 7.9311 -14.5353

H -6.4014 9.4615 -15.1187

C -0.6815 4.9281 -16.2658

H -1.2190 4.2687 -16.9633

H -0.4096 5.8453 -16.8119

N -3.7118 4.6606 12.6870

N -1.7496 4.9984 -3.1625

H -1.7030 4.9474 -4.7954

C -7.3595 6.4802 3.0898

C -7.2453 6.4513 1.7134

H -6.3058 6.1661 1.2721

C -8.3370 6.7832 0.9220

C -9.5706 7.1403 1.5000

C -9.6824 7.1486 2.9021

C -8.5635 6.8406 3.6659

H -8.6239 6.8612 4.7402

C -7.0661 6.7237 -1.0937

H -6.5569 5.7750 -0.8662

H -6.4312 7.5503 -0.7416

C -7.2581 6.8221 -2.6056

H -7.7811 5.9298 -2.9535

H -6.2635 6.8209 -3.0555

C -8.0351 8.0649 -3.0530

H -7.8420 8.8885 -2.3625

H -7.6774 8.3650 -4.0403

C -9.5397 7.8107 -3.1305

H -9.9077 7.5407 -2.1399

H -9.7180 6.9668 -3.8014

C -10.3031 9.0247 -3.6544

H -10.2518 9.8309 -2.9172

H -9.8298 9.3867 -4.5706

C -11.7655 8.6907 -3.9431

H -12.1992 8.2098 -3.0636

H -11.8151 7.9749 -4.7679

C -12.5915 9.9251 -4.2917

H -12.5206 10.6442 -3.4718

H -12.1781 10.4028 -5.1842

C -14.0604 9.5806 -4.5295

H -14.4349 9.0202 -3.6702

H -14.1454 8.9367 -5.4073

C -14.9226 10.8254 -4.7230

H -14.7782 11.4946 -3.8707

H -14.5977 11.3590 -5.6197

C -16.4058 10.4844 -4.8407

H -16.5633 9.8380 -5.7056

H -16.7135 9.9283 -3.9523

C -17.2732 11.7327 -4.9741

H -16.9742 12.2868 -5.8657

H -17.1052 12.3797 -4.1092

C -18.7550 11.3930 -5.0619

H -18.9604 10.7745 -5.9314

H -19.3553 12.2983 -5.1351

H -19.0662 10.8480 -4.1743

C -11.7560 8.0094 1.0464

H -11.5497 8.8412 1.7281

H -12.3190 7.2426 1.5884

C -12.5963 8.5205 -0.1191

H -12.7776 7.7077 -0.8205

H -12.0576 9.3103 -0.6428

C -13.9238 9.0453 0.4262

H -14.4394 8.2277 0.9375

H -13.7176 9.8198 1.1691

C -14.8538 9.6236 -0.6363

H -15.1170 8.8469 -1.3563

H -14.3421 10.4210 -1.1769

C -16.1240 10.1765 0.0065

H -16.6392 9.3688 0.5330

H -15.8477 10.9302 0.7472

C -17.0814 10.8035 -1.0023

H -17.3945 10.0459 -1.7240

H -16.5627 11.5912 -1.5541

C -18.3151 11.3925 -0.3239

H -18.8425 10.5988 0.2138

H -18.0016 12.1385 0.4107

C -19.2682 12.0379 -1.3235

H -19.5732 11.2930 -2.0600

H -18.7472 12.8351 -1.8591

C -20.5160 12.6108 -0.6573

H -21.0528 11.8034 -0.1499

H -20.2233 13.3429 0.0980

C -21.4477 13.2729 -1.6696

H -21.6913 12.5574 -2.4566

H -20.9307 14.1133 -2.1405

C -22.7394 13.7693 -1.0266

H -23.2552 12.9257 -0.5617

H -22.5011 14.4882 -0.2385

C -23.6673 14.4213 -2.0431

H -23.1813 15.2742 -2.5139

H -24.5783 14.7701 -1.5629

H -23.9385 13.7112 -2.8210

C -10.9530 7.6516 4.8774

H -10.3486 8.5321 5.1412

H -10.5661 6.7776 5.4149

C -12.4099 7.8443 5.2906

H -12.4204 8.0835 6.3563

H -12.9218 6.8927 5.1529

C -13.1342 8.9358 4.5060

H -13.2184 8.6221 3.4651

H -12.5451 9.8543 4.5293

C -14.5295 9.2134 5.0626

H -15.0725 8.2718 5.1619

H -14.4443 9.6488 6.0613

C -15.3328 10.1507 4.1635

H -14.8063 11.1014 4.0633

H -15.4119 9.7107 3.1668

C -16.7374 10.4070 4.7068

H -17.2661 9.4560 4.7970

H -16.6662 10.8396 5.7084

C -17.5424 11.3416 3.8067

H -17.5891 10.9209 2.8003

H -17.0332 12.3052 3.7386

C -18.9609 11.5565 4.3273

H -19.4807 10.5960 4.3584

H -18.9173 11.9418 5.3492

C -19.7554 12.5262 3.4558

H -19.2544 13.4979 3.4498

H -19.7727 12.1563 2.4281

C -21.1885 12.7016 3.9508

H -21.1740 13.0422 4.9900

H -21.6985 11.7356 3.9268

C -21.9740 13.7007 3.1057

H -21.4816 14.6747 3.1518

H -21.9689 13.3730 2.0637

C -23.4134 13.8409 3.5832

H -23.9310 12.8865 3.5204

H -23.9514 14.5638 2.9743

H -23.4415 14.1752 4.6193

O -10.5408 7.4523 0.5874

O -8.3182 6.7809 -0.4422

O -10.8890 7.4418 3.4782

C -8.3404 8.9776 -15.8882

C -8.7756 8.7551 -17.1802

H -8.0924 8.3106 -17.8831

C -10.0792 9.0730 -17.5300

C -10.9670 9.6190 -16.5857

C -10.4981 9.8837 -15.2891

C -9.1873 9.5614 -14.9644

H -8.8143 9.7549 -13.9740

C -9.7308 8.5812 -19.8351

H -9.1520 7.6706 -19.6180

H -9.0203 9.4106 -19.9710

C -10.5271 8.3570 -21.1195

H -11.1637 7.4793 -20.9893

H -9.8028 8.1343 -21.9061

C -11.3959 9.5462 -21.5399

H -10.8931 10.4795 -21.2784

H -11.5104 9.5251 -22.6264

C -12.7858 9.5171 -20.9058

H -12.6963 9.6449 -19.8267

H -13.2347 8.5369 -21.0929

C -13.7017 10.5914 -21.4899

H -13.3452 11.5795 -21.1908

H -13.6597 10.5456 -22.5827

C -15.1504 10.4116 -21.0442

H -15.1982 10.4331 -19.9538

H -15.5055 9.4297 -21.3683

C -16.0730 11.4883 -21.6123

H -15.7370 12.4714 -21.2752

H -16.0123 11.4781 -22.7035

C -17.5237 11.2743 -21.1871

H -17.5762 11.2240 -20.0968

H -17.8766 10.3155 -21.5738

C -18.4434 12.3883 -21.6816

H -18.1012 13.3453 -21.2799

H -18.3852 12.4496 -22.7707

C -19.8940 12.1528 -21.2667

H -20.2449 11.2159 -21.7021

H -19.9466 12.0460 -20.1802

C -20.8141 13.2907 -21.7028

H -20.7585 13.4019 -22.7882

H -20.4658 14.2250 -21.2565

C -22.2617 13.0423 -21.2961

H -22.6507 12.1533 -21.7869

H -22.8879 13.8880 -21.5733

H -22.3387 12.8932 -20.2213

C -13.3218 9.9737 -16.1891

H -13.2425 10.8642 -15.5567

H -13.3490 9.0904 -15.5412

C -14.5937 10.0622 -17.0263

H -14.7518 9.1199 -17.5496

H -14.4540 10.8402 -17.7777

C -15.8087 10.3993 -16.1666

H -15.9919 9.5902 -15.4558

H -15.6014 11.3014 -15.5848

C -17.0579 10.6225 -17.0148

H -17.2812 9.7136 -17.5766

H -16.8595 11.4179 -17.7358

C -18.2698 11.0074 -16.1713

H -18.5101 10.1916 -15.4857

H -18.0273 11.8842 -15.5649

C -19.4901 11.3133 -17.0353

H -19.7326 10.4365 -17.6407

H -19.2467 12.1293 -17.7199

C -20.7077 11.7049 -16.2027

H -20.9697 10.8802 -15.5352

H -20.4583 12.5675 -15.5795

C -21.9092 12.0425 -17.0792

H -22.1637 11.1759 -17.6944

H -21.6420 12.8589 -17.7538

C -23.1313 12.4498 -16.2614

H -23.3948 11.6406 -15.5757

H -22.8854 13.3279 -15.6580

C -24.3287 12.7661 -17.1522

H -24.5845 11.8808 -17.7407

H -24.0580 13.5584 -17.8521

C -25.5495 13.2051 -16.3479

H -25.8260 12.4142 -15.6459

H -25.2961 14.0931 -15.7643

C -26.7369 13.5202 -17.2487

H -26.4874 14.3226 -17.9395

H -27.5949 13.8312 -16.6580

H -27.0192 12.6442 -17.8291

C -10.9588 10.6005 -13.0427

H -10.2244 11.4115 -12.9528

H -10.5096 9.6661 -12.6851

C -12.2068 10.9028 -12.2175

H -11.9082 11.0155 -11.1744

H -12.8725 10.0433 -12.2901

C -12.9677 12.1401 -12.6880

H -13.0071 12.1279 -13.7784

H -12.4399 13.0442 -12.3869

C -14.3914 12.1593 -12.1393

H -14.8712 11.2057 -12.3711

H -14.3633 12.2626 -11.0526

C -15.2335 13.2811 -12.7366

H -14.8299 14.2508 -12.4387

H -15.1781 13.2269 -13.8275

C -16.6952 13.1766 -12.3088

H -17.0736 12.1871 -12.5770

H -16.7630 13.2739 -11.2223

C -17.5704 14.2373 -12.9681

H -17.4463 14.1821 -14.0529

H -17.2423 15.2308 -12.6529

C -19.0458 14.0540 -12.6244

H -19.3698 13.0606 -12.9456

H -19.1764 14.1077 -11.5407

C -19.9244 15.1094 -13.2908

H -19.6162 16.1012 -12.9516

H -19.7743 15.0728 -14.3733

C -21.4046 14.9062 -12.9795

H -21.5538 14.9335 -11.8981

H -21.7160 13.9185 -13.3286

C -22.2763 15.9732 -13.6357

H -21.9738 16.9577 -13.2726

H -22.1126 15.9549 -14.7159

C -23.7561 15.7589 -13.3425

H -24.0770 14.7785 -13.6885

H -24.3589 16.5150 -13.8410

H -23.9467 15.8198 -12.2737

O -12.2183 9.8947 -17.0721

O -10.6099 8.8767 -18.7766

O -11.3551 10.4602 -14.3964

C -2.7643 2.1482 -13.8583

C -3.6461 2.7197 -14.6929

C -3.5762 2.6520 -16.1340

C -4.6362 3.1520 -16.9209

C -2.4754 2.1046 -16.7930

H -1.6547 1.7588 -16.1901

C -4.3172 2.8165 -21.2780

C -3.3735 2.4363 -20.4005

C -3.4624 2.5106 -18.9593

C -2.4004 2.0101 -18.1715

C -4.5575 3.0731 -18.3001

H -5.3605 3.4589 -18.9023

O -1.3390 1.4482 -18.8207

C -7.7530 4.8689 -16.0628

C -7.4701 5.1033 -14.7298

H -6.5230 4.7850 -14.3304

C -8.4052 5.7583 -13.9340

C -9.6302 6.1715 -14.4783

C -9.9202 5.9072 -15.8277

C -8.9722 5.2498 -16.5995

H -9.1627 5.0291 -17.6362

C -7.2112 5.3730 -11.9012

H -6.2094 5.6180 -12.2874

H -7.3667 4.2902 -12.0095

C -7.2826 5.7675 -10.4272

H -6.9870 6.8121 -10.3263

H -6.5474 5.1573 -9.8984

C -8.6701 5.5879 -9.7994

H -9.1923 4.7654 -10.2923

H -8.5502 5.3204 -8.7481

C -9.5112 6.8601 -9.8924

H -9.6185 7.1419 -10.9405

H -8.9835 7.6664 -9.3767

C -10.8931 6.6914 -9.2634

H -11.4844 6.0033 -9.8724

H -10.7926 6.2475 -8.2698

C -11.6304 8.0262 -9.1474

H -11.5593 8.5563 -10.1005

H -11.1421 8.6471 -8.3925

C -13.1027 7.8430 -8.7868

H -13.5656 7.1777 -9.5203

H -13.1815 7.3586 -7.8109

C -13.8704 9.1639 -8.7669

H -13.6947 9.6926 -9.7080

H -13.4950 9.7975 -7.9600

C -15.3708 8.9419 -8.5929

H -15.7148 8.2364 -9.3549

H -15.5593 8.4887 -7.6168

C -16.1755 10.2337 -8.7186

H -15.8830 10.9210 -7.9230

H -15.9381 10.7109 -9.6734

C -17.6791 9.9780 -8.6482

H -17.9266 9.5318 -7.6830

H -17.9550 9.2565 -9.4193

C -18.4919 11.2532 -8.8411

H -18.2873 11.9645 -8.0452

H -19.5568 11.0321 -8.8366

H -18.2428 11.7234 -9.7909

C -11.7505 6.2734 -13.4211

H -11.6433 5.4962 -12.6485

H -12.1319 5.8105 -14.3365

C -12.6886 7.3783 -12.9597

H -12.6111 8.1841 -13.6907

H -12.3334 7.7564 -12.0015

C -14.1435 6.9376 -12.8379

H -14.4468 6.4398 -13.7617

H -14.2481 6.2202 -12.0206

C -15.0576 8.1367 -12.5920

H -14.9299 8.8476 -13.4134

H -14.7536 8.6375 -11.6711

C -16.5334 7.7598 -12.4893

H -16.8169 7.1587 -13.3557

H -16.6952 7.1515 -11.5965

C -17.4216 9.0008 -12.4298

H -17.3133 9.5617 -13.3625

H -17.0818 9.6489 -11.6197

C -18.8948 8.6638 -12.2185

H -19.2196 7.9528 -12.9816

H -19.0228 8.1799 -11.2468

C -19.7713 9.9121 -12.2859

H -19.7185 10.3312 -13.2937

H -19.3812 10.6653 -11.5982

C -21.2295 9.6281 -11.9348

H -21.6045 8.8169 -12.5634

H -21.2946 9.2958 -10.8967

C -22.1055 10.8628 -12.1314

H -22.0665 11.1676 -13.1809

H -21.7054 11.6891 -11.5376

C -23.5596 10.6172 -11.7357

H -23.9425 9.7501 -12.2781

H -23.6109 10.3860 -10.6691

C -24.4366 11.8271 -12.0377

H -24.0718 12.7048 -11.5081

H -25.4640 11.6439 -11.7326

H -24.4293 12.0460 -13.1035

C -11.5062 5.8552 -17.5925

H -11.5259 4.7549 -17.5959

H -10.7841 6.2029 -18.3396

C -12.8810 6.4060 -17.9509

H -13.1772 5.9386 -18.8923

H -12.7975 7.4823 -18.1098

C -13.9346 6.1178 -16.8843

H -13.7931 6.8138 -16.0567

H -13.7945 5.1063 -16.4972

C -15.3479 6.2574 -17.4499

H -15.3791 7.1068 -18.1364

H -15.5873 5.3629 -18.0276

C -16.3989 6.4689 -16.3648

H -16.3908 5.6258 -15.6693

H -16.1445 7.3661 -15.7961

C -17.7955 6.6316 -16.9561

H -17.7734 7.4292 -17.7032

H -18.0820 5.7113 -17.4671

C -18.8439 6.9714 -15.9021

H -18.5221 7.8581 -15.3514

H -18.9265 6.1486 -15.1873

C -20.2099 7.2415 -16.5277

H -20.1113 8.0494 -17.2566

H -20.5450 6.3506 -17.0624

C -21.2584 7.6330 -15.4909

H -21.3860 6.8158 -14.7762

H -20.9035 8.5035 -14.9355

C -22.6033 7.9621 -16.1326

H -22.9623 7.0915 -16.6861

H -22.4667 8.7782 -16.8454

C -23.6498 8.3707 -15.1006

H -23.7775 7.5615 -14.3757

H -23.2905 9.2464 -14.5561

C -24.9934 8.6922 -15.7414

H -24.8948 9.5172 -16.4441

H -25.7222 8.9740 -14.9840

H -25.3765 7.8282 -16.2798

O -10.4829 6.8704 -13.6598

O -8.2117 6.0666 -12.6221

O -11.1281 6.3050 -16.3022

O -5.6864 3.6831 -16.2461

C -2.7812 2.1622 -12.4160

C -1.7540 1.4816 -11.7489

C -3.7439 2.7919 -11.6196

C -1.7356 1.4544 -10.3731

H -0.9845 0.9838 -12.3215

C -3.6289 2.7170 -10.2435

H -4.5625 3.3312 -12.0646

H -0.9670 0.9284 -9.8240

H -4.3446 3.1853 -9.5802

C -4.2270 2.7307 -22.7213

C -5.3390 3.0764 -23.4965

C -3.0870 2.3143 -23.4163

C -5.2655 2.9801 -24.8730

H -6.2472 3.4153 -23.0205

C -3.1216 2.2602 -24.7973

H -2.1850 2.0428 -22.8913

H -6.1113 3.2376 -25.4972

H -2.2560 1.9420 -25.3621

C -0.2749 0.9656 -18.0276

H 0.1822 1.7942 -17.4647

H -0.6452 0.2253 -17.3010

C -6.7545 4.2265 -16.9863

H -6.3819 4.9935 -17.6825

H -7.2477 3.4407 -17.5756

C -5.2219 2.0799 12.5788

C -5.3462 2.1201 6.9276

C -5.1560 2.1064 5.7335

C -4.7456 2.0561 4.3921

C -5.5853 2.3746 3.3107

C -3.4227 1.6694 4.1648

H -2.8386 1.4239 5.0369

O -4.2201 1.6791 13.1550

O -6.2514 2.6080 13.1915

C -3.3198 2.3828 -6.3947

C -3.1832 1.9788 -0.7399

C -3.3212 1.9224 0.4613

C -3.7240 1.9349 1.8091

C -2.8868 1.6149 2.8914

C -5.0523 2.2993 2.0371

H -5.6434 2.5238 1.1646

O -4.2848 2.8304 -6.9752

O -2.2495 1.9315 -7.0192

O -1.5952 1.2786 2.6109

C -9.0710 3.4376 2.9055

C -9.4515 3.5531 4.2270

H -8.7333 3.3278 4.9975

C -10.7383 3.9701 4.5367

C -11.6669 4.2810 3.5252

C -11.2677 4.1624 2.1807

C -9.9774 3.7297 1.9047

H -9.6502 3.6243 0.8854

C -10.5451 3.3407 6.8178

H -9.5000 3.6591 6.9458

H -10.5438 2.2843 6.5101

C -11.2671 3.4939 8.1565

H -11.0456 4.4794 8.5704

H -10.8458 2.7463 8.8319

C -12.7873 3.3301 8.0563

H -13.0300 2.6581 7.2309

H -13.1645 2.8835 8.9793

C -13.4761 4.6740 7.8325

H -13.0951 5.1104 6.9103

H -13.2202 5.3408 8.6598

C -14.9946 4.5516 7.7489

H -15.2633 4.0353 6.8258

H -15.3636 3.9543 8.5867

C -15.6622 5.9256 7.7826

H -15.1980 6.5680 7.0306

H -15.4856 6.3870 8.7568

C -17.1636 5.8512 7.5238

H -17.3341 5.4071 6.5399

H -17.6279 5.1998 8.2686

C -17.8235 7.2272 7.5775

H -17.3117 7.9005 6.8851

H -17.7095 7.6454 8.5800

C -19.3057 7.1652 7.2179

H -19.4118 6.7327 6.2192

H -19.8205 6.5048 7.9195

C -19.9658 8.5407 7.2416

H -19.8902 8.9618 8.2471

H -19.4289 9.2118 6.5661

C -21.4337 8.4792 6.8290

H -21.9729 7.8156 7.5095

H -21.5047 8.0517 5.8254

C -22.0863 9.8561 6.8387

H -22.0616 10.2833 7.8382

H -23.1252 9.7910 6.5205

H -21.5615 10.5322 6.1673

C -13.8816 5.1348 3.1094

H -14.1099 4.3928 2.3390

H -13.4980 6.0369 2.6202

C -15.1586 5.4758 3.8711

H -14.9144 6.0799 4.7434

H -15.6311 4.5599 4.2249

C -16.0998 6.2396 2.9443

H -15.6322 7.1904 2.6749

H -16.2327 5.6674 2.0225

C -17.4712 6.5221 3.5490

H -17.3520 7.0582 4.4922

H -17.9808 5.5811 3.7634

C -18.3188 7.3557 2.5913

H -17.8250 8.3162 2.4242

H -18.3770 6.8434 1.6275

C -19.7325 7.6085 3.1065

H -19.6796 8.0899 4.0849

H -20.2513 6.6556 3.2338

C -20.5235 8.4944 2.1487

H -20.0245 9.4627 2.0637

H -20.5274 8.0376 1.1548

C -21.9631 8.7121 2.6044

H -21.9651 9.1435 3.6089

H -22.4761 7.7497 2.6584

C -22.7228 9.6387 1.6592

H -22.2293 10.6135 1.6373

H -22.6880 9.2284 0.6470

C -24.1784 9.8237 2.0783

H -24.2137 10.2279 3.0924

H -24.6746 8.8507 2.0963

C -24.9324 10.7572 1.1343

H -24.4409 11.7322 1.1274

H -24.8897 10.3543 0.1192

C -26.3884 10.9321 1.5473

H -26.9063 9.9759 1.5384

H -26.9015 11.6050 0.8646

H -26.4541 11.3467 2.5515

C -11.9525 3.9806 -0.1250

H -12.1780 2.9054 -0.1183

H -10.9205 4.1189 -0.4578

C -12.8483 4.7243 -1.1151

H -12.8266 4.1671 -2.0534

H -12.4100 5.7065 -1.2999

C -14.2886 4.8998 -0.6430

H -14.2914 5.5502 0.2327

H -14.6974 3.9326 -0.3449

C -15.1720 5.5146 -1.7280

H -14.6617 6.3786 -2.1606

H -15.3217 4.7883 -2.5301

C -16.5281 5.9578 -1.1839

H -17.0340 5.1037 -0.7261

H -16.3711 6.7064 -0.4035

C -17.4219 6.5504 -2.2712

H -16.8831 7.3533 -2.7817

H -17.6481 5.7828 -3.0150

C -18.7267 7.1055 -1.7044

H -18.4969 7.8535 -0.9409

H -19.2836 6.3001 -1.2179

C -19.6002 7.7445 -2.7830

H -19.0319 8.5330 -3.2826

H -19.8515 6.9964 -3.5375

C -20.8850 8.3358 -2.2066

H -21.4728 7.5392 -1.7440

H -20.6304 9.0540 -1.4225

C -21.7290 9.0344 -3.2702

H -21.9865 8.3212 -4.0562

H -21.1431 9.8316 -3.7327

C -23.0085 9.6306 -2.6887

H -23.6232 8.8297 -2.2713

H -22.7516 10.3107 -1.8732

C -23.8131 10.3865 -3.7395

H -23.2246 11.1988 -4.1598

H -24.7154 10.8068 -3.3012

H -24.1032 9.7225 -4.5518

O -12.9013 4.6357 3.9991

O -11.1865 4.1125 5.8235

O -12.1481 4.4944 1.1846

O -6.8633 2.7334 3.5936

C -5.4000 2.0869 8.3312

C -6.5009 2.5294 9.0666

C -4.2718 1.5982 9.0023

C -6.4658 2.5074 10.4445

H -7.3751 2.8959 8.5470

C -4.2430 1.5715 10.3751

H -3.4309 1.2501 8.4210

C -5.3352 2.0377 11.1079

H -7.3061 2.8584 11.0238

H -3.3824 1.2039 10.9116

C -3.1537 2.0784 -2.1435

C -4.3212 2.5052 -2.7876

C -2.0242 1.7652 -2.9030

C -4.3601 2.6061 -4.1593

H -5.1895 2.7465 -2.1919

C -2.0692 1.8667 -4.2777

H -1.1234 1.4420 -2.4017

C -3.2352 2.2827 -4.9139

H -5.2512 2.9273 -4.6756

H -1.2052 1.6243 -4.8754

C -0.7382 1.0676 3.7108

H -0.8190 1.9109 4.4114

H -1.0335 0.1642 4.2621

C -7.6951 3.0073 2.4856

H -7.2557 3.8167 1.8841

H -7.7797 2.1076 1.8601

N -2.6493 2.0661 -9.6340

N -4.1837 2.5778 -25.5189

H -2.3989 1.9965 -8.0175

C 0.7823 0.2966 -18.8624

C 0.5409 -0.1488 -20.1485

H -0.4246 0.0275 -20.5910

C 1.5453 -0.8073 -20.8482

C 2.8097 -1.0157 -20.2628

C 3.0337 -0.5832 -18.9410

C 2.0072 0.0645 -18.2614

H 2.1437 0.3867 -17.2434

C 0.0724 -1.4828 -22.5882

H -0.3677 -0.5069 -22.8432

H -0.5508 -1.9551 -21.8158

C 0.0956 -2.3595 -23.8373

H 0.7250 -1.8828 -24.5896

H -0.9257 -2.3887 -24.2240

C 0.6031 -3.7832 -23.5809

H 0.2525 -4.1310 -22.6064

H 0.1792 -4.4440 -24.3424

C 2.1254 -3.8857 -23.6417

H 2.5672 -3.2144 -22.9052

H 2.4534 -3.5549 -24.6315

C 2.6264 -5.3106 -23.4074

H 2.4435 -5.6025 -22.3708

H 2.0719 -6.0081 -24.0385

C 4.1148 -5.4279 -23.7214

H 4.6620 -4.6920 -23.1308

H 4.2781 -5.1886 -24.7758

C 4.6757 -6.8175 -23.4335

H 4.5292 -7.0515 -22.3770

H 4.1216 -7.5570 -24.0142

C 6.1624 -6.9031 -23.7791

H 6.7050 -6.1337 -23.2270

H 6.2947 -6.6942 -24.8439

C 6.7639 -8.2688 -23.4587

H 6.6386 -8.4783 -22.3939

H 6.2234 -9.0376 -24.0148

C 8.2478 -8.3443 -23.8162

H 8.3713 -8.1683 -24.8865

H 8.7890 -7.5547 -23.2897

C 8.8542 -9.6985 -23.4546

H 8.3064 -10.4856 -23.9780

H 8.7355 -9.8716 -22.3832

C 10.3307 -9.7876 -23.8213

H 10.4657 -9.6759 -24.8956

H 10.7413 -10.7499 -23.5221

H 10.8978 -9.0015 -23.3269

C 5.0494 -1.3706 -21.0787

H 5.4381 -1.1677 -20.0771

H 5.1444 -0.4619 -21.6901

C 5.8154 -2.5319 -21.7031

H 5.6244 -2.5596 -22.7769

H 5.4178 -3.4494 -21.2662

C 7.3164 -2.4636 -21.4384

H 7.7370 -1.5746 -21.9127

H 7.4855 -2.3813 -20.3609

C 8.0287 -3.7093 -21.9562

H 7.9340 -3.7556 -23.0441

H 7.5393 -4.5934 -21.5429

C 9.5068 -3.7433 -21.5750

H 10.0098 -2.8626 -21.9803

H 9.5971 -3.7072 -20.4862

C 10.1928 -5.0044 -22.0910

H 10.1238 -5.0331 -23.1807

H 9.6643 -5.8798 -21.7036

C 11.6595 -5.0865 -21.6755

H 12.1951 -4.2119 -22.0519

H 11.7258 -5.0736 -20.5841

C 12.3234 -6.3545 -22.2037

H 12.2739 -6.3611 -23.2950

H 11.7685 -7.2232 -21.8423

C 13.7801 -6.4788 -21.7629

H 14.3414 -5.6087 -22.1133

H 13.8257 -6.4836 -20.6708

C 14.4294 -7.7515 -22.2991

H 14.4416 -7.7193 -23.3908

H 13.8268 -8.6130 -22.0026

C 15.8528 -7.9390 -21.7822

H 16.4603 -7.0786 -22.0718

H 15.8366 -7.9794 -20.6907

C 16.4872 -9.2141 -22.3236

H 15.9053 -10.0837 -22.0262

H 17.4993 -9.3317 -21.9421

H 16.5310 -9.1875 -23.4103

C 4.3824 -0.6846 -16.9647

H 3.7757 -1.4527 -16.4648

H 4.0411 0.3033 -16.6434

C 5.8477 -0.8156 -16.5610

H 5.8710 -0.8582 -15.4705

H 6.3781 0.0840 -16.8750

C 6.5690 -2.0318 -17.1293

H 6.6193 -1.9389 -18.2150

H 6.0119 -2.9392 -16.8975

C 7.9837 -2.1307 -16.5601

H 8.4648 -1.1514 -16.6170

H 7.9272 -2.4098 -15.5052

C 8.8406 -3.1461 -17.3084

H 8.3384 -4.1144 -17.3104

H 8.9487 -2.8230 -18.3465

C 10.2271 -3.2997 -16.6880

H 10.7075 -2.3200 -16.6248

H 10.1246 -3.6855 -15.6707

C 11.1079 -4.2438 -17.5001

H 11.2470 -3.8299 -18.5019

H 10.6007 -5.2063 -17.6041

C 12.4731 -4.4657 -16.8578

H 12.9811 -3.5046 -16.7439

H 12.3359 -4.8892 -15.8602

C 13.3445 -5.4063 -17.6859

H 12.8319 -6.3653 -17.7928

H 13.4762 -4.9845 -18.6856

C 14.7147 -5.6373 -17.0557

H 14.5834 -6.0357 -16.0467

H 15.2412 -4.6843 -16.9709

C 15.5631 -6.6055 -17.8743

H 15.0292 -7.5545 -17.9730

H 15.7060 -6.1966 -18.8771

C 16.9235 -6.8559 -17.2374

H 17.4806 -5.9257 -17.1489

H 17.5069 -7.5486 -17.8391

H 16.8074 -7.2813 -16.2427

O 3.6849 -1.7549 -21.0099

O 1.3880 -1.3102 -22.0990

O 4.2476 -0.8400 -18.3704

C 0.6990 0.9261 3.2853

C 1.1250 1.3087 2.0254

C 2.4725 1.1954 1.6872

C 3.3922 0.7395 2.6405

C 2.9488 0.3352 3.9111

C 1.5966 0.4244 4.2170

H 1.2317 0.1202 5.1841

C 2.1347 2.0764 -0.4863

H 1.6746 2.9694 -0.0455

H 1.3367 1.3717 -0.7638

C 2.9277 2.4938 -1.7204

H 3.8606 2.9477 -1.3865

H 2.3456 3.2636 -2.2306

C 3.2211 1.3628 -2.7040

H 2.3047 0.7990 -2.8984

H 3.5328 1.8150 -3.6490

C 4.3223 0.4118 -2.2418

H 4.0049 -0.1206 -1.3450

H 5.2045 0.9991 -1.9791

C 4.6848 -0.5743 -3.3522

H 3.8734 -1.2935 -3.4869

H 4.7878 -0.0182 -4.2879

C 5.9934 -1.3206 -3.0968

H 5.8899 -1.9733 -2.2276

H 6.7764 -0.5920 -2.8731

C 6.4112 -2.1373 -4.3197

H 5.7014 -2.9521 -4.4786

H 6.3705 -1.4903 -5.1992

C 7.8251 -2.7030 -4.1995

H 7.8612 -3.4463 -3.4004

H 8.5088 -1.8950 -3.9286

C 8.2978 -3.3309 -5.5104

H 7.6660 -4.1887 -5.7519

H 8.1829 -2.5986 -6.3137

C 9.7587 -3.7712 -5.4493

H 10.3750 -2.9230 -5.1409

H 9.8730 -4.5529 -4.6957

C 10.2597 -4.2881 -6.7946

H 10.1155 -3.5156 -7.5543

H 9.6645 -5.1559 -7.0862

C 11.7317 -4.6774 -6.7457

H 12.3443 -3.8185 -6.4772

H 12.0665 -5.0454 -7.7129

H 11.8955 -5.4583 -6.0061

C 5.2728 -0.5724 2.0881

H 4.7878 -1.0392 1.2195

H 5.0926 -1.2018 2.9666

C 6.7679 -0.4068 1.8572

H 7.1506 0.2314 2.6553

H 6.9237 0.1072 0.9069

C 7.5259 -1.7300 1.8714

H 7.2930 -2.2654 2.7942

H 7.2052 -2.3535 1.0346

C 9.0318 -1.4944 1.7956

H 9.3237 -0.8184 2.6041

H 9.2742 -1.0051 0.8491

C 9.8406 -2.7820 1.9204

H 9.5775 -3.2805 2.8557

H 9.5838 -3.4569 1.1019

C 11.3417 -2.5070 1.9032

H 11.5901 -1.8238 2.7197

H 11.6068 -2.0129 0.9647

C 12.1670 -3.7814 2.0511

H 11.8990 -4.2747 2.9886

H 11.9264 -4.4664 1.2356

C 13.6672 -3.4971 2.0451

H 13.9084 -2.8185 2.8676

H 13.9361 -2.9958 1.1127

C 14.4932 -4.7727 2.1890

H 14.2193 -5.2737 3.1204

H 14.2557 -5.4490 1.3646

C 15.9937 -4.4917 2.1929

H 16.2307 -3.8090 3.0125

H 16.2707 -3.9954 1.2595

C 16.8190 -5.7662 2.3489

H 16.5382 -6.2656 3.2793

H 16.5898 -6.4466 1.5254

C 18.3138 -5.4748 2.3650

H 18.6177 -4.9820 1.4435

H 18.8837 -6.3959 2.4649

H 18.5667 -4.8249 3.2000

C 3.4948 -0.7671 5.9626

H 2.9291 -1.6755 5.7174

H 2.8587 -0.1061 6.5628

C 4.7445 -1.1048 6.7661

H 4.4339 -1.6470 7.6607

H 5.1959 -0.1674 7.0856

C 5.7664 -1.9183 5.9770

H 6.0358 -1.3632 5.0765

H 5.3218 -2.8658 5.6674

C 7.0283 -2.1871 6.7926

H 7.3981 -1.2439 7.2001

H 6.7850 -2.8350 7.6367

C 8.1319 -2.8333 5.9581

H 7.7716 -3.7797 5.5495

H 8.3785 -2.1801 5.1170

C 9.3896 -3.0858 6.7839

H 9.7193 -2.1471 7.2353

H 9.1512 -3.7734 7.5974

C 10.5299 -3.6729 5.9553

H 10.7861 -2.9834 5.1468

H 10.2013 -4.6108 5.5019

C 11.7662 -3.9332 6.8119

H 12.0848 -2.9983 7.2793

H 11.5019 -4.6258 7.6134

C 12.9263 -4.5193 6.0145

H 12.6104 -5.4601 5.5570

H 13.1994 -3.8328 5.2089

C 14.1470 -4.7721 6.8962

H 13.8727 -5.4552 7.7038

H 14.4595 -3.8326 7.3576

C 15.3147 -5.3654 6.1126

H 15.0027 -6.3114 5.6641

H 15.5849 -4.6864 5.3009

C 16.5308 -5.6028 6.9975

H 16.8611 -4.6705 7.4518

H 17.3546 -6.0147 6.4173

H 16.2916 -6.3023 7.7959

O 4.7279 0.7235 2.3058

O 2.9927 1.4943 0.4701

O 3.8969 -0.1218 4.7645

H -6.0885 2.7168 14.1765

H -0.9595 4.2669 1.4590

H -3.7569 5.4928 1.4910

H -1.9852 4.1536 7.9708

H -4.7469 5.4510 8.0900

H -1.9356 1.5866 -14.2678

H -4.4940 3.2615 -14.3018

H -2.4435 2.0236 -20.7616

H -5.2570 3.2141 -20.9201

H 0.3888 1.6566 1.3206

4640

mixteNew3_XTB_HT-8chains_xtbopt.xyz 0.000000

C -3.0840 12.1170 -34.0930

C -2.2870 11.8880 -35.1490

C -2.6060 12.1090 -36.5400

C -1.5890 11.9640 -37.5090

C -3.8830 12.4730 -36.9820

H -4.6540 12.5990 -36.2410

C -2.5530 12.2810 -41.6550

C -3.4480 12.5880 -40.7020

C -3.1810 12.4620 -39.2870

C -4.1900 12.6510 -38.3200

C -1.9000 12.1620 -38.8370

H -1.1300 12.1030 -39.5800

O -5.4070 13.0150 -38.8030

C 1.9830 11.0790 -37.6540

C 2.1900 10.5930 -36.3780

H 1.3780 10.6300 -35.6730

C 3.4420 10.0930 -36.0280

C 4.4840 10.0870 -36.9670

C 4.2500 10.5440 -38.2770

C 2.9980 11.0470 -38.6000

H 2.7980 11.4250 -39.5890

C 2.7770 9.6350 -33.7820

H 1.8320 9.1860 -34.1200

H 2.5890 10.6840 -33.5100

C 3.2710 8.8540 -32.5690

H 3.4170 7.8180 -32.8720

H 2.4710 8.8800 -31.8260

C 4.5650 9.3860 -31.9500

H 4.5150 10.4740 -31.8760

H 4.6440 8.9900 -30.9330

C 5.8110 8.9730 -32.7310

H 5.8020 9.4430 -33.7130

H 5.7850 7.8930 -32.8910

C 7.0970 9.3380 -31.9930

H 7.1140 10.4120 -31.7940

H 7.1190 8.8260 -31.0270

C 8.3360 8.9520 -32.7980

H 8.3480 9.5240 -33.7270

H 8.2740 7.8950 -33.0650

C 9.6360 9.2020 -32.0370

H 9.6960 10.2570 -31.7570

H 9.6350 8.6180 -31.1130

C 10.8590 8.8290 -32.8710

H 10.8750 9.4390 -33.7770

H 10.7770 7.7840 -33.1820

C 12.1680 9.0260 -32.1120

H 12.2550 10.0710 -31.8040

H 12.1560 8.4180 -31.2030

C 13.3810 8.6460 -32.9580

H 13.2830 7.6070 -33.2830

H 13.4020 9.2680 -33.8560

C 14.6950 8.8140 -32.2000

H 14.6740 8.1960 -31.2990

H 14.7980 9.8540 -31.8810

C 15.8960 8.4260 -33.0550

H 15.8140 7.3920 -33.3830

H 16.8200 8.5370 -32.4930

H 15.9530 9.0570 -33.9390

C 6.7800 10.5150 -36.5450

H 6.7720 11.0130 -35.5640

H 6.6670 11.2830 -37.3160

C 8.0740 9.7360 -36.7360

H 8.0520 9.2750 -37.7220

H 8.1000 8.9340 -35.9960

C 9.3340 10.5860 -36.6110

H 9.2950 11.4050 -37.3330

H 9.3900 11.0280 -35.6130

C 10.5710 9.7270 -36.8660

H 10.5140 9.3100 -37.8730

H 10.5680 8.8880 -36.1660

C 11.8890 10.4820 -36.7160

H 11.9120 11.3250 -37.4120

H 11.9660 10.8900 -35.7050

C 13.0760 9.5600 -36.9860

H 13.0050 9.1710 -38.0050

H 13.0230 8.7050 -36.3070

C 14.4240 10.2510 -36.8060

H 14.4870 11.1110 -37.4780

H 14.5070 10.6300 -35.7840

C 15.5820 9.2970 -37.0890

H 15.4990 8.9230 -38.1130

H 15.5100 8.4370 -36.4190

C 16.9450 9.9590 -36.9060

H 17.0140 10.8290 -37.5650

H 17.0410 10.3220 -35.8800

C 18.0900 8.9980 -37.2120

H 17.9690 8.6030 -38.2230

H 18.0450 8.1530 -36.5200

C 19.4560 9.6670 -37.0950

H 19.5050 10.5120 -37.7870

H 19.5790 10.0620 -36.0830

C 20.5900 8.6970 -37.4030

H 20.5670 7.8540 -36.7160

H 21.5540 9.1910 -37.3070

H 20.5000 8.3140 -38.4170

C 5.0780 10.8560 -40.4920

H 4.9390 11.9450 -40.5140

H 4.1860 10.3730 -40.9110

C 6.2940 10.4530 -41.3200

H 6.1200 10.7740 -42.3480

H 6.3610 9.3650 -41.3270

C 7.5870 11.0570 -40.7800

H 7.6760 10.8090 -39.7210

H 7.5440 12.1430 -40.8570

C 8.8120 10.5380 -41.5270

H 8.8300 9.4480 -41.4670

H 8.7400 10.8050 -42.5840

C 10.1100 11.0940 -40.9450

H 10.1500 12.1740 -41.1040

H 10.1200 10.9240 -39.8650

C 11.3440 10.4410 -41.5620

H 11.3060 9.3650 -41.3740

H 11.3330 10.5840 -42.6450

C 12.6430 11.0030 -40.9880

H 12.6070 10.9490 -39.8980

H 12.7350 12.0570 -41.2630

C 13.8680 10.2390 -41.4840

H 13.7940 9.1990 -41.1560

H 13.8810 10.2390 -42.5770

C 15.1720 10.8380 -40.9620

H 15.2840 11.8530 -41.3510

H 15.1250 10.9060 -39.8720

C 16.3880 10.0060 -41.3580

H 16.4110 9.8880 -42.4440

H 16.2960 9.0090 -40.9190

C 17.6970 10.6390 -40.8910

H 17.8140 11.6160 -41.3640

H 17.6510 10.7970 -39.8120

C 18.9020 9.7680 -41.2200

H 18.8140 8.7980 -40.7350

H 19.8210 10.2370 -40.8760

H 18.9800 9.6080 -42.2930

O 5.7040 9.5760 -36.5950

O 3.7430 9.5740 -34.8100

O 5.2760 10.4360 -39.1520

O -0.3280 11.6350 -37.1000

C -2.7510 11.8820 -32.7040

C -3.6860 12.1970 -31.7120

C -1.5330 11.3510 -32.2670

C -3.3670 11.9890 -30.3840

H -4.6460 12.6110 -31.9820

C -1.3210 11.1790 -30.9120

H -0.7590 11.0850 -32.9690

H -4.0650 12.2370 -29.5960

H -0.3850 10.7810 -30.5410

C -2.6880 12.4130 -43.0820

C -1.5680 12.1110 -43.8680

C -3.8440 12.8290 -43.7480

C -1.6530 12.2180 -45.2360

H -0.6490 11.7950 -43.3970

C -3.8250 12.9070 -45.1270

H -4.7360 13.0830 -43.2000

H -0.8140 11.9860 -45.8800

H -4.6830 13.2270 -45.7010

C -6.4960 13.1590 -37.9170

H -6.5940 12.2580 -37.2950

H -6.3340 14.0220 -37.2550

C 0.6580 11.6220 -38.1130

H 0.3300 10.9830 -38.9450

H 0.7940 12.6410 -38.5040

C -4.0200 13.1840 -48.8040

C -4.8050 13.6790 -54.3810

C -5.0040 13.7200 -55.5720

C -5.3320 13.7550 -56.9370

C -4.4050 13.3470 -57.9150

C -6.5990 14.2080 -57.3100

H -7.2500 14.5630 -56.5280

O -4.8180 13.6130 -47.9950

O -2.9000 12.5820 -48.4620

C -6.1240 12.4600 -67.7000

C -6.5700 13.4200 -62.1480

C -6.4310 13.5900 -60.9580

C -6.1090 13.7030 -59.5970

C -7.0040 14.1990 -58.6330

C -4.8190 13.3120 -59.2320

H -4.1730 12.9710 -60.0240

O -5.0220 12.2210 -68.1760

O -7.2290 12.4890 -68.4010

H -7.0700 12.2670 -69.3720

O -8.1980 14.6490 -59.0980

C -0.8350 12.4970 -57.9070

C -0.6690 12.1280 -56.5820

H -1.5400 12.0740 -55.9500

C 0.6120 11.8720 -56.0990

C 1.7250 11.9950 -56.9470

C 1.5420 12.3260 -58.3000

C 0.2560 12.5860 -58.7580

H 0.0920 12.8730 -59.7840

C 0.0150 11.6260 -53.7770

H -1.0030 11.3470 -54.0730

H 0.0140 12.6820 -53.4680

C 0.4350 10.7340 -52.6080

H 0.3170 9.6910 -52.9060

H -0.2640 10.9350 -51.7930

C 1.8720 10.9520 -52.1240

H 2.1220 12.0120 -52.1940

H 1.9310 10.6670 -51.0710

C 2.8940 10.1320 -52.9110

H 2.8860 10.4510 -53.9540

H 2.6030 9.0780 -52.8780

C 4.3020 10.2740 -52.3370

H 4.6280 11.3110 -52.4390

H 4.2860 10.0390 -51.2690

C 5.3090 9.3610 -53.0340

H 5.2620 9.5320 -54.1110

H 5.0370 8.3170 -52.8560

C 6.7320 9.6090 -52.5400

H 6.9910 10.6560 -52.7150

H 6.7730 9.4350 -51.4620

C 7.7660 8.7200 -53.2270

H 7.7270 8.8830 -54.3080

H 7.5230 7.6700 -53.0460

C 9.1770 9.0100 -52.7210

H 9.4110 10.0630 -52.8980

H 9.2090 8.8430 -51.6430

C 10.2400 8.1410 -53.3890

H 9.9980 7.0870 -53.2320

H 10.2340 8.3220 -54.4670

C 11.6340 8.4280 -52.8370

H 11.6340 8.2520 -51.7600

H 11.8770 9.4810 -53.0000

C 12.7030 7.5560 -53.4840

H 12.4860 6.5020 -53.3250

H 13.6810 7.7720 -53.0610

H 12.7480 7.7360 -54.5570

C 3.9520 12.7240 -56.5420

H 3.5470 13.7060 -56.2760

H 4.2700 12.7710 -57.5880

C 5.1290 12.3270 -55.6550

H 5.1690 11.2370 -55.6370

H 4.9600 12.6660 -54.6320

C 6.4570 12.8610 -56.1800

H 6.5580 12.5750 -57.2300

H 6.4620 13.9520 -56.1330

C 7.6490 12.3060 -55.4040

H 7.5440 11.2230 -55.3200

H 7.6540 12.7160 -54.3910

C 8.9780 12.6160 -56.0890

H 8.9490 12.2180 -57.1070

H 9.1130 13.6970 -56.1610

C 10.1610 11.9980 -55.3500

H 9.9850 10.9280 -55.2280

H 10.2360 12.4380 -54.3520

C 11.4790 12.2000 -56.0930

H 11.3970 11.7550 -57.0880

H 11.6660 13.2680 -56.2230

C 12.6530 11.5620 -55.3570

H 12.4510 10.4980 -55.2150

H 12.7490 12.0160 -54.3670

C 13.9690 11.7200 -56.1140

H 13.8730 11.2530 -57.0970

H 14.1720 12.7820 -56.2710

C 15.1390 11.0840 -55.3690

H 14.9320 10.0230 -55.2140

H 15.2350 11.5480 -54.3840

C 16.4570 11.2340 -56.1250

H 16.3680 10.7590 -57.1040

H 16.6600 12.2950 -56.2900

C 17.6200 10.6070 -55.3660

H 17.7450 11.0840 -54.3960

H 18.5470 10.7170 -55.9240

H 17.4400 9.5470 -55.2040

C 2.5220 12.6170 -60.4710

H 2.3090 13.6820 -60.6180

H 1.7030 12.0240 -60.9030

C 3.8210 12.2050 -61.1560

H 3.6970 12.3420 -62.2320

H 3.9580 11.1410 -60.9630

C 5.0640 12.9490 -60.6680

H 5.0390 13.0080 -59.5790

H 5.0690 13.9720 -61.0490

C 6.3200 12.2030 -61.1150

H 6.2480 11.1690 -60.7680

H 6.3550 12.1800 -62.2070

C 7.6250 12.7960 -60.5860

H 7.7780 13.7940 -61.0020

H 7.5660 12.9000 -59.5000

C 8.8050 11.8960 -60.9510

H 8.6330 10.9010 -60.5320

H 8.8490 11.7900 -62.0380

C 10.1500 12.4170 -60.4490

H 10.1090 12.5530 -59.3650

H 10.3530 13.3930 -60.8940

C 11.2800 11.4510 -60.8000

H 11.0780 10.4800 -60.3410

H 11.3020 11.3040 -61.8830

C 12.6480 11.9480 -60.3380

H 12.8670 12.9050 -60.8170

H 12.6280 12.1200 -59.2590

C 13.7550 10.9500 -60.6730

H 13.7640 10.7700 -61.7510

H 13.5430 9.9980 -60.1810

C 15.1330 11.4430 -60.2380

H 15.3590 12.3810 -60.7490

H 15.1210 11.6510 -59.1650

C 16.2230 10.4230 -60.5440

H 16.0220 9.4840 -60.0330

H 17.1940 10.7890 -60.2200

H 16.2720 10.2260 -61.6140

O 2.9460 11.7240 -56.3890

O 0.9220 11.4550 -54.8450

O 2.6470 12.3390 -59.0890

O -3.1550 13.0240 -57.4880

C -4.5850 13.5940 -52.9960

C -3.3980 13.0430 -52.5080

C -5.5700 14.0300 -52.1050

C -3.2110 12.9030 -51.1510

H -2.6370 12.7300 -53.2060

C -5.3800 13.8830 -50.7500

H -6.4690 14.4840 -52.4920

C -4.2050 13.3140 -50.2670

H -2.3010 12.4780 -50.7610

H -6.1310 14.1990 -50.0430

C -6.5310 13.2030 -63.5350

C -5.2750 12.9420 -64.0990

C -7.6660 13.2230 -64.3480

C -5.1600 12.7150 -65.4480

H -4.4080 12.9230 -63.4560

C -7.5450 12.9900 -65.7020

H -8.6340 13.4160 -63.9070

C -6.2940 12.7370 -66.2600

H -4.2040 12.5090 -65.9040

H -8.4140 12.9920 -66.3430

C -9.1060 15.2290 -58.1890

H -9.2180 14.5960 -57.2990

H -8.7340 16.2150 -57.8680

C -2.1950 12.7750 -58.4910

H -2.5090 11.9070 -59.0940

H -2.1280 13.6420 -59.1650

N -2.2080 11.4920 -29.9820

N -2.7590 12.6060 -45.8520

H -2.8220 12.5640 -47.4470

C -7.7700 13.3360 -38.7040

C -7.8380 12.9810 -40.0390

H -6.9440 12.6390 -40.5300

C -9.0380 13.0750 -40.7260

C -10.1880 13.5780 -40.0880

C -10.1220 13.8920 -38.7200

C -8.9060 13.7840 -38.0530

H -8.8390 14.0280 -37.0060

C -8.0990 12.3460 -42.7980

H -7.4650 11.6040 -42.2890

H -7.5050 13.2570 -42.9550

C -8.5410 11.7590 -44.1400

H -8.9210 10.7480 -43.9810

H -7.6450 11.6840 -44.7610

C -9.6090 12.5820 -44.8650

H -9.4660 13.6400 -44.6500

H -9.4900 12.4380 -45.9420

C -11.0230 12.1700 -44.4620

H -11.1370 12.3140 -43.3890

H -11.1580 11.1070 -44.6840

C -12.0940 12.9720 -45.1970

H -12.0710 14.0050 -44.8450

H -11.8790 12.9800 -46.2690

C -13.4840 12.3850 -44.9680

H -13.6450 12.2630 -43.8950

H -13.5350 11.3910 -45.4200

C -14.5970 13.2580 -45.5420

H -14.5360 14.2540 -45.0980

H -14.4580 13.3690 -46.6200

C -15.9780 12.6660 -45.2660

H -16.0860 12.4990 -44.1910

H -16.0590 11.6940 -45.7570

C -17.1090 13.5730 -45.7440

H -17.0020 14.5570 -45.2830

H -17.0350 13.7080 -46.8260

C -18.4810 13.0000 -45.3980

H -18.5870 12.0150 -45.8590

H -18.5520 12.8630 -44.3160

C -19.6200 13.9030 -45.8660

H -19.5580 14.0300 -46.9480

H -19.5050 14.8890 -45.4120

C -20.9850 13.3310 -45.5010

H -21.1330 12.3650 -45.9770

H -21.7790 13.9990 -45.8250

H -21.0670 13.1980 -44.4240

C -12.3360 14.5410 -40.5100

H -11.9220 15.4760 -40.1250

H -12.9380 14.0610 -39.7290

C -13.2150 14.8430 -41.7190

H -13.4490 13.9100 -42.2330

H -12.6610 15.4780 -42.4100

C -14.5030 15.5190 -41.2530

H -14.9200 14.9390 -40.4250

H -14.2700 16.5160 -40.8700

C -15.5710 15.6310 -42.3380

H -15.7620 14.6410 -42.7570

H -15.2150 16.2700 -43.1480

C -16.8700 16.1940 -41.7640

H -17.1580 15.6000 -40.8920

H -16.6990 17.2180 -41.4220

C -18.0200 16.1810 -42.7660

H -18.1960 15.1540 -43.0970

H -17.7460 16.7690 -43.6460

C -19.3050 16.7410 -42.1600

H -19.5450 16.1830 -41.2510

H -19.1430 17.7830 -41.8750

C -20.4850 16.6580 -43.1250

H -20.6690 15.6130 -43.3810

H -20.2350 17.1860 -44.0490

C -21.7560 17.2590 -42.5310

H -21.9860 16.7560 -41.5890

H -21.5860 18.3160 -42.3090

C -22.9480 17.1280 -43.4760

H -23.1400 16.0690 -43.6700

H -22.7050 17.5980 -44.4320

C -24.2090 17.7730 -42.9050

H -24.4350 17.3280 -41.9330

H -24.0270 18.8380 -42.7430

C -25.4060 17.5990 -43.8310

H -25.2090 18.0550 -44.7990

H -26.2910 18.0660 -43.4050

H -25.6180 16.5430 -43.9880

C -11.2360 14.6890 -36.7400

H -10.6650 15.6250 -36.6550

H -10.7630 13.9210 -36.1140

C -12.6710 14.8830 -36.2540

H -12.6350 15.1430 -35.1950

H -13.1760 13.9240 -36.3520

C -13.4700 15.9370 -37.0200

H -13.3310 15.7800 -38.0900

H -13.1070 16.9360 -36.7750

C -14.9560 15.8260 -36.6820

H -15.2790 14.8010 -36.8810

H -15.1000 16.0110 -35.6150

C -15.8480 16.7740 -37.4820

H -15.6680 17.8050 -37.1720

H -15.6040 16.6970 -38.5440

C -17.3210 16.4220 -37.2770

H -17.4900 15.4020 -37.6360

H -17.5460 16.4360 -36.2080

C -18.2860 17.3570 -38.0010

H -18.0290 17.4000 -39.0610

H -18.1920 18.3660 -37.5940

C -19.7290 16.8780 -37.8470

H -19.8280 15.8890 -38.3040

H -19.9580 16.7740 -36.7830

C -20.7450 17.8250 -38.4800

H -20.6760 18.8030 -37.9980

H -20.5080 17.9590 -39.5370

C -22.1700 17.2930 -38.3440

H -22.3940 17.1270 -37.2860

H -22.2440 16.3270 -38.8510

C -23.2070 18.2480 -38.9300

H -23.1520 19.2050 -38.4050

H -22.9730 18.4340 -39.9800

C -24.6200 17.6900 -38.8170

H -24.6960 16.7320 -39.3280

H -25.3390 18.3730 -39.2630

H -24.8910 17.5410 -37.7740

O -11.2840 13.6720 -40.8880

O -9.2260 12.6620 -42.0090

O -11.2780 14.2680 -38.0880

C -10.4670 15.3540 -58.8200

C -10.7860 14.6640 -59.9740

H -10.0140 14.1050 -60.4700

C -12.0860 14.6810 -60.4540

C -13.0930 15.3940 -59.7700

C -12.7460 16.1230 -58.6200

C -11.4310 16.1030 -58.1720

H -11.1530 16.6420 -57.2810

C -11.5130 13.5230 -62.4470

H -10.8180 12.8450 -61.9260

H -10.9380 14.3760 -62.8320

C -12.1540 12.7560 -63.6060

H -12.5200 11.7940 -63.2380

H -11.3540 12.5540 -64.3220

C -13.3050 13.4880 -64.3030

H -13.1230 14.5620 -64.2990

H -13.3470 13.1590 -65.3440

C -14.6440 13.1880 -63.6340

H -14.6280 13.5910 -62.6230

H -14.7650 12.1020 -63.5680

C -15.8370 13.7590 -64.3950

H -15.8120 14.8490 -64.3440

H -15.7710 13.4770 -65.4490

C -17.1520 13.2370 -63.8180

H -17.1890 13.4690 -62.7520

H -17.1770 12.1480 -63.9170

C -18.3820 13.8260 -64.5020

H -18.3800 14.9120 -64.3760

H -18.3350 13.6220 -65.5740

C -19.6780 13.2500 -63.9350

H -19.7060 13.4210 -62.8560

H -19.6940 12.1700 -64.0970

C -20.9170 13.8750 -64.5730

H -20.9040 14.9550 -64.4030

H -20.8860 13.7150 -65.6540

C -22.2120 13.2900 -64.0150

H -22.2390 12.2170 -64.2130

H -22.2330 13.4260 -62.9310

C -23.4480 13.9470 -64.6270

H -23.4250 13.8190 -65.7110

H -23.4220 15.0210 -64.4240

C -24.7410 13.3570 -64.0770

H -24.8090 12.2980 -64.3150

H -25.6050 13.8610 -64.5050

H -24.7820 13.4640 -62.9950

C -15.3640 16.1770 -60.0340

H -15.0580 17.1670 -60.3920

H -15.5720 16.2240 -58.9610

C -16.6160 15.7090 -60.7650

H -16.8170 14.6820 -60.4570

H -16.4290 15.7100 -61.8380

C -17.8230 16.5820 -60.4300

H -17.8880 16.7090 -59.3460

H -17.6960 17.5710 -60.8750

C -19.1180 15.9520 -60.9330

H -19.2420 14.9760 -60.4570

H -19.0460 15.7870 -62.0100

C -20.3460 16.8080 -60.6340

H -20.3730 17.0480 -59.5680

H -20.2760 17.7500 -61.1840

C -21.6320 16.0860 -61.0230

H -21.7190 15.1720 -60.4290

H -21.5740 15.7900 -62.0730

C -22.8780 16.9400 -60.8120

H -22.9140 17.2850 -59.7760

H -22.8250 17.8240 -61.4530

C -24.1490 16.1590 -61.1330

H -24.2200 15.3020 -60.4580

H -24.0830 15.7690 -62.1510

C -25.4110 17.0060 -61.0030

H -25.4630 17.4270 -59.9970

H -25.3620 17.8390 -61.7080

C -26.6690 16.1860 -61.2760

H -26.7290 15.3700 -60.5510

H -26.5990 15.7370 -62.2700

C -27.9430 17.0240 -61.1930

H -28.0120 17.4850 -60.2050

H -27.8930 17.8290 -61.9300

C -29.1890 16.1820 -61.4420

H -29.1490 15.7230 -62.4280

H -30.0840 16.7960 -61.3850

H -29.2680 15.3900 -60.7000

C -13.3960 17.6560 -56.8750

H -12.7840 18.5010 -57.2220

H -12.8320 17.0970 -56.1180

C -14.6980 18.1550 -56.2500

H -14.4530 18.7650 -55.3790

H -15.2570 17.2840 -55.9040

C -15.5610 18.9460 -57.2320

H -15.5930 18.3990 -58.1740

H -15.1080 19.9220 -57.4200

C -16.9900 19.1230 -56.7250

H -17.3930 18.1420 -56.4630

H -16.9920 19.7310 -55.8180

C -17.8990 19.7580 -57.7770

H -17.5970 20.7930 -57.9500

H -17.7830 19.2170 -58.7190

C -19.3670 19.7130 -57.3600

H -19.6430 18.6750 -57.1530

H -19.5050 20.2790 -56.4360

C -20.2970 20.2610 -58.4400

H -20.1000 19.7400 -59.3790

H -20.0880 21.3210 -58.5990

C -21.7670 20.0800 -58.0680

H -21.9630 19.0180 -57.9030

H -21.9720 20.6030 -57.1300

C -22.7090 20.5940 -59.1530

H -22.5370 21.6620 -59.3070

H -22.4850 20.0840 -60.0920

C -24.1740 20.3640 -58.7930

H -24.4010 20.8740 -57.8540

H -24.3410 19.2950 -58.6370

C -25.1220 20.8620 -59.8800

H -24.9540 21.9290 -60.0450

H -24.8980 20.3430 -60.8150

C -26.5820 20.6300 -59.5110

H -26.7730 19.5690 -59.3650

H -27.2400 20.9900 -60.2980

H -26.8310 21.1530 -58.5890

O -14.3380 15.2370 -60.2970

O -12.4990 14.0000 -61.5570

O -13.7310 16.8200 -57.9650

C -4.0940 9.9930 -56.4270

C -5.1350 10.1760 -57.2550

C -5.0670 10.0780 -58.6950

C -6.2060 10.3290 -59.4910

C -3.8880 9.7100 -59.3390

H -3.0440 9.4860 -58.7130

C -5.8130 9.7880 -63.8340

C -4.8060 9.7270 -62.9470

C -4.9130 9.8610 -61.5110

C -3.7720 9.6150 -60.7130

C -6.1050 10.2070 -60.8670

H -6.9700 10.3910 -61.4790

O -2.5930 9.2860 -61.3310

C -9.5890 11.3860 -58.5580

C -9.2430 11.8340 -57.2970

H -8.2110 11.7910 -56.9920

C -10.2180 12.3480 -56.4550

C -11.5500 12.4750 -56.8880

C -11.9060 11.9890 -58.1590

C -10.9130 11.4270 -58.9590

H -11.1630 11.0350 -59.9300

C -8.7870 12.3120 -54.5560

H -7.8920 12.7460 -55.0300

H -8.7330 11.2180 -54.6550

C -8.7810 12.7000 -53.0800

H -8.9050 13.7810 -53.0060

H -7.7940 12.4400 -52.6910

C -9.8520 12.0110 -52.2330

H -9.9520 10.9690 -52.5450

H -9.5170 12.0110 -51.1930

C -11.2110 12.7030 -52.3100

H -11.5780 12.6760 -53.3360

H -11.0870 13.7530 -52.0350

C -12.2240 12.0630 -51.3630

H -12.3800 11.0180 -51.6450

H -11.8220 12.0720 -50.3470

C -13.5620 12.7960 -51.3790

H -13.9630 12.7790 -52.3960

H -13.4050 13.8430 -51.1090

C -14.5820 12.1780 -50.4260

H -14.6830 11.1130 -50.6480

H -14.2220 12.2640 -49.3980

C -15.9500 12.8440 -50.5490

H -16.2480 12.8480 -51.6010

H -15.8810 13.8850 -50.2240

C -17.0270 12.1270 -49.7400

H -17.0490 11.0730 -50.0280

H -16.7800 12.1690 -48.6770

C -18.4070 12.7390 -49.9730

H -18.4170 13.7670 -49.6030

H -18.6050 12.7740 -51.0470

C -19.5130 11.9400 -49.2910

H -19.3240 11.9100 -48.2160

H -19.4880 10.9150 -49.6640

C -20.8920 12.5350 -49.5480

H -20.9490 13.5520 -49.1670

H -21.6630 11.9440 -49.0570

H -21.1050 12.5580 -50.6150

C -13.7380 12.8140 -55.8830

H -13.9150 12.7330 -54.8020

H -13.9750 11.8560 -56.3480

C -14.6300 13.9230 -56.4410

H -14.6020 13.8900 -57.5300

H -14.2230 14.8830 -56.1230

C -16.0590 13.7660 -55.9230

H -16.3860 12.7360 -56.0820

H -16.0700 13.9450 -54.8450

C -17.0560 14.7070 -56.5940

H -17.0890 14.4970 -57.6650

H -16.7270 15.7410 -56.4750

C -18.4520 14.5420 -55.9950

H -18.7290 13.4850 -56.0240

H -18.4300 14.8420 -54.9440

C -19.5210 15.3500 -56.7250

H -19.5430 15.0570 -57.7780

H -19.2700 16.4130 -56.6850

C -20.9010 15.1280 -56.1090

H -21.1170 14.0560 -56.0940

H -20.8910 15.4730 -55.0720

C -22.0110 15.8480 -56.8690

H -22.0150 15.5120 -57.9080

H -21.8120 16.9230 -56.8700

C -23.3810 15.5880 -56.2480

H -23.5650 14.5110 -56.2190

H -23.3830 15.9480 -55.2160

C -24.5070 16.2690 -57.0200

H -24.5100 15.9050 -58.0500

H -24.3260 17.3470 -57.0500

C -25.8740 16.0070 -56.3910

H -26.0500 14.9290 -56.3500

H -25.8750 16.3810 -55.3650

C -26.9980 16.6730 -57.1740

H -26.8440 17.7490 -57.2230

H -27.9590 16.4860 -56.7010

H -27.0360 16.2840 -58.1890

C -13.6150 11.4900 -59.7590

H -13.6170 10.4010 -59.6280

H -12.9300 11.7580 -60.5690

C -14.9970 11.9830 -60.1680

H -15.2140 11.5140 -61.1310

H -14.9400 13.0600 -60.3240

C -16.1210 11.6500 -59.1910

H -16.0970 12.3470 -58.3560

H -15.9650 10.6480 -58.7930

C -17.4750 11.7310 -59.8990

H -17.4810 12.6090 -60.5500

H -17.5930 10.8570 -60.5440

C -18.6740 11.8240 -58.9580

H -18.6690 10.9810 -58.2640

H -18.6010 12.7380 -58.3670

C -19.9830 11.8340 -59.7460

H -19.9380 12.6260 -60.4990

H -20.0860 10.8880 -60.2830

C -21.2190 12.0520 -58.8760

H -21.1360 13.0100 -58.3580

H -21.2740 11.2690 -58.1160

C -22.4940 12.0360 -59.7170

H -22.4240 12.8090 -60.4870

H -22.5700 11.0740 -60.2310

C -23.7600 12.2630 -58.8950

H -23.8330 11.4930 -58.1220

H -23.7010 13.2310 -58.3920

C -25.0110 12.2180 -59.7710

H -25.0570 11.2550 -60.2830

H -24.9410 12.9920 -60.5400

C -26.2970 12.4200 -58.9740

H -26.3630 11.6560 -58.1960

H -26.2710 13.3950 -58.4810

C -27.5310 12.3330 -59.8630

H -27.4980 13.0890 -60.6450

H -28.4370 12.4820 -59.2780

H -27.5890 11.3580 -60.3420

O -12.3590 13.1220 -56.0000

O -9.9610 12.7870 -55.1840

O -13.2030 12.1060 -58.5470

O -7.3270 10.6790 -58.8180

C -4.1070 9.9830 -54.9840

C -2.9680 9.5010 -54.3260

C -5.1790 10.3970 -54.1870

C -2.9550 9.4520 -52.9500

H -2.1190 9.1500 -54.8940

C -5.0590 10.3170 -52.8130

H -6.0790 10.7860 -54.6320

H -2.1030 9.0750 -52.4030

H -5.8520 10.6290 -52.1480

C -5.6970 9.6130 -65.2670

C -6.8590 9.5730 -66.0470

C -4.4840 9.4740 -65.9480

C -6.7580 9.3850 -67.4120

H -7.8280 9.6820 -65.5820

C -4.4970 9.2970 -67.3190

H -3.5450 9.5040 -65.4200

H -7.6390 9.3390 -68.0380

H -3.5760 9.1840 -67.8760

C -1.4930 9.0640 -60.4740

H -1.3530 9.9300 -59.8120

H -1.7000 8.1810 -59.8440

C -8.5330 10.8920 -59.5160

H -8.3830 11.6500 -60.3020

H -8.8600 9.9560 -59.9920

C -4.0900 8.6770 -29.7250

C -3.5940 9.0100 -35.3560

C -3.5340 9.0750 -36.5610

C -3.4430 9.1150 -37.9620

C -4.5950 9.3580 -38.7350

C -2.2140 8.8820 -38.5840

H -1.3650 8.6820 -37.9480

O -3.1950 8.2090 -29.0360

O -5.2330 9.0980 -29.2380

C -4.4130 10.0100 -48.8710

C -3.3970 9.3610 -43.3310

C -3.2140 9.2690 -42.1390

C -3.2260 9.1950 -40.7340

C -2.0860 8.9130 -39.9610

C -4.4570 9.3970 -40.1110

H -5.2940 9.5780 -40.7650

O -5.4540 10.4540 -49.3030

O -3.4120 9.6350 -49.6460

O -0.9310 8.7060 -40.6540

C -8.1780 9.9320 -38.1390

C -8.2090 10.2240 -36.7890

H -7.3020 10.1170 -36.2170

C -9.4000 10.6490 -36.2140

C -10.5800 10.7440 -36.9760

C -10.5310 10.4710 -38.3550

C -9.3270 10.0440 -38.8990

H -9.2660 9.8110 -39.9480

C -8.4780 10.8380 -34.0090

H -7.5250 11.1710 -34.4420

H -8.3990 9.7640 -33.7810

C -8.7190 11.6310 -32.7230

H -8.5490 12.6910 -32.9240

H -7.9670 11.3030 -32.0030

C -10.1220 11.4630 -32.1320

H -10.4790 10.4490 -32.3170

H -10.0690 11.6070 -31.0500

C -11.1110 12.4700 -32.7170

H -11.1990 12.2960 -33.7890

H -10.7150 13.4780 -32.5660

C -12.4930 12.3860 -32.0720

H -12.9570 11.4330 -32.3330

H -12.3980 12.4240 -30.9840

C -13.3830 13.5330 -32.5450

H -13.3650 13.5610 -33.6370

H -12.9720 14.4810 -32.1890

C -14.8320 13.4060 -32.0830

H -15.2140 12.4220 -32.3650

H -14.8810 13.4790 -30.9940

C -15.7120 14.4850 -32.7120

H -15.6360 14.4110 -33.8000

H -15.3390 15.4710 -32.4260

C -17.1780 14.3610 -32.3060

H -17.5140 13.3350 -32.4780

H -17.2800 14.5640 -31.2380

C -18.0680 15.3180 -33.0970

H -17.7110 16.3420 -32.9610

H -17.9890 15.0790 -34.1610

C -19.5320 15.2350 -32.6730

H -19.6250 15.5450 -31.6290

H -19.8680 14.1970 -32.7370

C -20.4250 16.1080 -33.5460

H -20.1100 17.1480 -33.4950

H -21.4610 16.0480 -33.2190

H -20.3730 15.7830 -34.5830

C -12.9520 11.0830 -36.8550

H -13.1030 10.1320 -37.3770

H -13.0170 11.8930 -37.5900

C -14.0420 11.2610 -35.8000

H -13.7480 12.0500 -35.1080

H -14.1570 10.3430 -35.2220

C -15.3500 11.6260 -36.4980

H -15.2190 12.5880 -37.0000

H -15.5570 10.8870 -37.2770

C -16.5580 11.7200 -35.5690

H -16.3200 12.3590 -34.7180

H -16.8050 10.7290 -35.1830

C -17.7550 12.2980 -36.3230

H -17.5330 13.3340 -36.5910

H -17.8920 11.7440 -37.2560

C -19.0580 12.2620 -35.5320

H -18.9220 12.7700 -34.5770

H -19.3290 11.2260 -35.3210

C -20.1850 12.9370 -36.3130

H -19.9310 13.9890 -36.4660

H -20.2640 12.4750 -37.3000

C -21.5360 12.8440 -35.6110

H -21.4640 13.2910 -34.6170

H -21.8050 11.7930 -35.4820

C -22.6300 13.5520 -36.4070

H -22.3740 14.6090 -36.5080

H -22.6750 13.1290 -37.4130

C -24.0020 13.4260 -35.7500

H -23.9580 13.8400 -34.7390

H -24.2650 12.3700 -35.6630

C -25.0850 14.1510 -36.5450

H -24.8260 15.2090 -36.6230

H -25.1200 13.7470 -37.5590

C -26.4580 14.0090 -35.9000

H -26.7440 12.9610 -35.8430

H -27.2100 14.5390 -36.4800

H -26.4510 14.4180 -34.8920

C -11.6610 10.2610 -40.4730

H -11.7050 9.1650 -40.5100

H -10.7620 10.5990 -40.9960

C -12.8610 10.8870 -41.1870

H -13.0110 10.3290 -42.1140

H -12.5960 11.9110 -41.4470

C -14.1500 10.9050 -40.3720

H -14.0140 11.5680 -39.5180

H -14.3650 9.9080 -39.9860

C -15.3290 11.4000 -41.2050

H -15.0630 12.3510 -41.6700

H -15.5340 10.6860 -42.0070

C -16.5890 11.5990 -40.3680

H -16.8510 10.6610 -39.8730

H -16.3890 12.3370 -39.5870

C -17.7670 12.0690 -41.2160

H -17.4980 13.0010 -41.7190

H -17.9750 11.3230 -41.9870

C -19.0270 12.2960 -40.3850

H -18.8300 13.0590 -39.6270

H -19.2860 11.3720 -39.8610

C -20.2070 12.7320 -41.2480

H -19.9510 13.6600 -41.7650

H -20.3970 11.9700 -42.0080

C -21.4760 12.9470 -40.4280

H -21.7280 12.0200 -39.9060

H -21.2900 13.7110 -39.6680

C -22.6560 13.3730 -41.2960

H -22.8310 12.6160 -42.0640

H -22.4120 14.3090 -41.8040

C -23.9310 13.5610 -40.4790

H -24.1770 12.6220 -39.9770

H -23.7560 14.3130 -39.7050

C -25.1070 13.9910 -41.3480

H -24.8910 14.9340 -41.8430

H -26.0040 14.1170 -40.7460

H -25.3090 13.2430 -42.1110

O -11.6760 11.1170 -36.2460

O -9.5380 11.0470 -34.9140

O -11.6440 10.6760 -39.1150

O -5.7690 9.5360 -38.0750

C -3.7070 8.9340 -33.9560

C -4.9120 9.2870 -33.3410

C -2.6350 8.5000 -33.1700

C -5.0420 9.2190 -31.9740

H -5.7370 9.6110 -33.9570

C -2.7720 8.4230 -31.8030

H -1.7040 8.2230 -33.6390

C -3.9710 8.7870 -31.1930

H -5.9680 9.4920 -31.4910

H -1.9570 8.0880 -31.1800

C -3.6340 9.4960 -44.7120

C -4.8810 9.9540 -45.1480

C -2.6450 9.1910 -45.6490

C -5.1240 10.1200 -46.4930

H -5.6460 10.1720 -44.4170

C -2.8950 9.3550 -46.9940

H -1.6930 8.8180 -45.3070

C -4.1320 9.8270 -47.4240

H -6.0780 10.4750 -46.8520

H -2.1430 9.1130 -47.7280

C 0.1890 8.1710 -39.9880

H 0.4660 8.7840 -39.1180

H -0.0380 7.1590 -39.6140

C -6.9220 9.5890 -38.8880

H -6.7990 10.3690 -39.6520

H -7.0520 8.6220 -39.3960

N -3.9750 9.8580 -52.2090

N -5.6030 9.2450 -68.0440

H -3.6660 9.7570 -50.6180

C -0.1870 8.8280 -61.1830

C -0.0900 8.3110 -62.4610

H -0.9900 8.1070 -63.0150

C 1.1640 8.0840 -63.0140

C 2.3430 8.3780 -62.2940

C 2.2300 8.8490 -60.9730

C 0.9610 9.0750 -60.4500

H 0.8570 9.4490 -59.4440

C 0.2830 6.8720 -64.8580

H -0.4490 7.5920 -65.2520

H -0.2190 6.2360 -64.1150

C 0.8080 6.0030 -65.9980

H 1.2500 6.6450 -66.7600

H -0.0560 5.5020 -66.4380

C 1.8460 4.9720 -65.5370

H 1.6530 4.6950 -64.4990

H 1.7470 4.0670 -66.1410

C 3.2750 5.4960 -65.6690

H 3.3640 6.4380 -65.1270

H 3.4750 5.6940 -66.7260

C 4.3100 4.4980 -65.1470

H 4.3230 4.5270 -64.0550

H 4.0260 3.4850 -65.4440

C 5.7070 4.7990 -65.6880

H 5.9420 5.8530 -65.5250

H 5.7140 4.6290 -66.7680

C 6.7840 3.9340 -65.0380

H 6.8120 4.1390 -63.9650

H 6.5250 2.8800 -65.1660

C 8.1660 4.1900 -65.6380

H 8.4110 5.2500 -65.5450

H 8.1440 3.9540 -66.7050

C 9.2510 3.3570 -64.9600

H 9.2630 3.5840 -63.8900

H 9.0110 2.2970 -65.0710

C 10.6370 3.6210 -65.5440

H 10.6290 3.3960 -66.6130

H 10.8810 4.6810 -65.4350

C 11.7140 2.7820 -64.8590

H 11.4830 1.7230 -64.9960

H 11.7020 2.9890 -63.7870

C 13.1050 3.0680 -65.4130

H 13.1500 2.8340 -66.4740

H 13.8520 2.4690 -64.8970

H 13.3600 4.1190 -65.2860

C 4.7410 8.4660 -62.4350

H 4.8900 7.9210 -61.4990

H 4.7800 9.5410 -62.2290

C 5.8610 8.0870 -63.3950

H 5.7830 8.6690 -64.3130

H 5.7580 7.0330 -63.6550

C 7.2030 8.3310 -62.7070

H 7.3270 9.4000 -62.5230

H 7.1880 7.8300 -61.7350

C 8.4020 7.8080 -63.4910

H 8.5050 8.3560 -64.4300

H 8.2390 6.7570 -63.7380

C 9.6820 7.9390 -62.6680

H 9.8660 8.9930 -62.4480

H 9.5410 7.4230 -61.7150

C 10.9010 7.3460 -63.3680

H 11.0940 7.8910 -64.2950

H 10.6900 6.3070 -63.6350

C 12.1390 7.3940 -62.4770

H 12.3560 8.4320 -62.2120

H 11.9320 6.8520 -61.5510

C 13.3600 6.7750 -63.1510

H 13.5970 7.3340 -64.0600

H 13.1240 5.7500 -63.4460

C 14.5780 6.7640 -62.2300

H 14.8120 7.7870 -61.9270

H 14.3380 6.1960 -61.3280

C 15.8000 6.1430 -62.9010

H 16.0590 6.7230 -63.7910

H 15.5540 5.1300 -63.2310

C 17.0070 6.0880 -61.9670

H 17.2540 7.1000 -61.6370

H 16.7500 5.5030 -61.0810

C 18.2220 5.4670 -62.6440

H 18.0040 4.4490 -62.9590

H 19.0690 5.4410 -61.9620

H 18.5060 6.0430 -63.5220

C 3.3000 9.1210 -58.8200

H 3.0690 8.1180 -58.4350

H 2.5260 9.8120 -58.4790

C 4.6280 9.6250 -58.2680

H 4.5060 9.7110 -57.1880

H 4.8010 10.6240 -58.6660

C 5.8200 8.7320 -58.5900

H 5.9240 8.6570 -59.6740

H 5.6410 7.7290 -58.1940

C 7.1220 9.2740 -58.0050

H 7.2710 10.3030 -58.3370

H 7.0540 9.2850 -56.9150

C 8.3120 8.4210 -58.4340

H 8.1430 7.3900 -58.1170

H 8.3800 8.4290 -59.5260

C 9.6420 8.8980 -57.8560

H 9.8200 9.9350 -58.1480

H 9.5950 8.8660 -56.7650

C 10.7910 8.0200 -58.3440

H 10.8500 8.0790 -59.4340

H 10.5800 6.9810 -58.0790

C 12.1400 8.4140 -57.7500

H 12.3620 9.4530 -58.0010

H 12.0870 8.3440 -56.6610

C 13.2590 7.5120 -58.2640

H 13.0080 6.4710 -58.0460

H 13.3320 7.6140 -59.3500

C 14.6120 7.8370 -57.6380

H 14.5520 7.7020 -56.5550

H 14.8560 8.8850 -57.8250

C 15.7220 6.9500 -58.1950

H 15.4700 5.9030 -58.0130

H 15.7860 7.0910 -59.2770

C 17.0740 7.2600 -57.5660

H 17.3560 8.2930 -57.7520

H 17.8450 6.6140 -57.9790

H 17.0370 7.1050 -56.4890

O 3.4860 8.1240 -62.9920

O 1.3620 7.5550 -64.2490

O 3.3680 9.0750 -60.2390

C 1.3710 8.1290 -40.9260

C 1.2410 8.5260 -42.2450

C 2.3580 8.5490 -43.0770

C 3.6040 8.1700 -42.5650

C 3.7240 7.7280 -41.2340

C 2.5930 7.7100 -40.4240

H 2.6470 7.3640 -39.4050

C 1.0900 9.1100 -45.0130

H 0.4670 9.8170 -44.4490

H 0.5710 8.1400 -45.0460

C 1.2710 9.6520 -46.4290

H 1.7010 10.6540 -46.3700

H 0.2720 9.7380 -46.8590

C 2.1570 8.7800 -47.3260

H 2.1010 7.7410 -46.9960

H 1.7820 8.8200 -48.3510

C 3.6120 9.2450 -47.3100

H 3.9610 9.2720 -46.2780

H 3.6610 10.2610 -47.7110

C 4.5270 8.3410 -48.1330

H 4.5790 7.3590 -47.6570

H 4.1090 8.2010 -49.1330

C 5.9340 8.9270 -48.2420

H 6.2330 9.3080 -47.2630

H 5.9260 9.7760 -48.9290

C 6.9690 7.9030 -48.7010

H 6.9060 7.0240 -48.0570

H 6.7480 7.5820 -49.7210

C 8.3880 8.4640 -48.6290

H 8.5590 8.8620 -47.6260

H 8.4900 9.2950 -49.3330

C 9.4490 7.4080 -48.9250

H 9.3170 6.5670 -48.2390

H 9.3170 7.0260 -49.9400

C 10.8620 7.9640 -48.7660

H 11.0080 8.7870 -49.4710

H 10.9750 8.3720 -47.7590

C 11.9330 6.9020 -48.9980

H 11.8100 6.4710 -49.9940

H 11.8010 6.0950 -48.2730

C 13.3380 7.4770 -48.8650

H 13.5010 8.2600 -49.6030

H 14.0870 6.7030 -49.0180

H 13.4820 7.9050 -47.8750

C 4.9460 7.0280 -44.0990

H 4.1020 6.8550 -44.7830

H 5.0150 6.1790 -43.4060

C 6.2410 7.1730 -44.8860

H 6.2080 8.1230 -45.4170

H 6.2950 6.3670 -45.6200

C 7.4700 7.1380 -43.9850

H 7.3520 7.8900 -43.2030

H 7.5350 6.1580 -43.5050

C 8.7540 7.4040 -44.7670

H 8.7480 8.4320 -45.1350

H 8.7910 6.7420 -45.6360

C 9.9980 7.1770 -43.9140

H 9.9550 7.8230 -43.0340

H 10.0060 6.1400 -43.5670

C 11.2910 7.4540 -44.6770

H 11.3330 8.5110 -44.9500

H 11.2980 6.8720 -45.6020

C 12.5200 7.0930 -43.8480

H 12.5040 7.6600 -42.9140

H 12.4770 6.0310 -43.5930

C 13.8280 7.3750 -44.5820

H 13.8990 8.4430 -44.8000

H 13.8310 6.8410 -45.5350

C 15.0400 6.9430 -43.7600

H 15.0240 7.4590 -42.7980

H 14.9720 5.8700 -43.5610

C 16.3590 7.2380 -44.4680

H 16.4380 8.3120 -44.6530

H 16.3700 6.7370 -45.4390

C 17.5610 6.7770 -43.6470

H 17.5430 7.2740 -42.6760

H 17.4850 5.7020 -43.4710

C 18.8800 7.0810 -44.3460

H 18.9330 6.5680 -45.3030

H 19.7210 6.7580 -43.7360

H 18.9800 8.1490 -44.5280

C 5.2130 6.9970 -39.5030

H 5.0030 5.9270 -39.3780

H 4.5680 7.5670 -38.8270

C 6.6530 7.3480 -39.1420

H 6.7600 7.2030 -38.0670

H 6.7850 8.4110 -39.3450

C 7.7270 6.5510 -39.8760

H 7.6210 6.6960 -40.9520

H 7.6010 5.4870 -39.6680

C 9.1180 6.9960 -39.4230

H 9.2740 8.0350 -39.7200

H 9.1640 6.9580 -38.3320

C 10.2460 6.1390 -39.9910

H 10.1160 5.1060 -39.6630

H 10.2020 6.1480 -41.0820

C 11.6140 6.6430 -39.5340

H 11.7730 7.6520 -39.9200

H 11.6310 6.7050 -38.4440

C 12.7540 5.7410 -40.0020

H 12.7180 5.6450 -41.0890

H 12.6200 4.7430 -39.5790

C 14.1210 6.2820 -39.5900

H 14.2760 7.2590 -40.0560

H 14.1430 6.4290 -38.5080

C 15.2550 5.3460 -39.9970

H 15.1220 4.3820 -39.5010

H 15.2100 5.1720 -41.0740

C 16.6270 5.9120 -39.6380

H 16.6600 6.1340 -38.5690

H 16.7800 6.8510 -40.1750

C 17.7560 4.9470 -39.9880

H 17.6170 4.0150 -39.4340

H 17.7080 4.7070 -41.0520

C 19.1250 5.5310 -39.6630

H 19.3100 6.4210 -40.2620

H 19.9110 4.8110 -39.8750

H 19.1840 5.8060 -38.6130

O 4.7240 8.2260 -43.3670

O 2.3420 8.9410 -44.3800

O 4.9650 7.3610 -40.8530

H -5.3100 8.9270 -28.2490

H -1.5860 11.8940 -41.3690

H -4.4320 12.9400 -40.9700

H -1.2890 11.5080 -34.9890

H -4.0760 12.5200 -34.2460

H -3.1150 9.7840 -56.8350

H -6.1150 10.3900 -56.8570

H -3.8020 9.5360 -63.2940

H -6.8260 9.9510 -63.4900

H 0.2660 8.8130 -42.5990

C -4.9130 16.9130 -57.6930

C -4.0260 16.5640 -58.6400

C -4.2760 16.5180 -60.0630

C -3.2020 16.2810 -60.9490

C -5.5540 16.6650 -60.6010

H -6.3700 16.8310 -59.9210

C -4.1670 15.8250 -65.2260

C -5.0160 16.1980 -64.2550

C -4.7290 16.3290 -62.8440

C -5.8040 16.5730 -61.9580

C -3.4460 16.1970 -62.3080

H -2.6300 16.0200 -62.9890

C -9.4330 16.9310 -62.4020

C -10.5360 17.4900 -61.7730

H -10.4380 17.8530 -60.7630

C -11.7520 17.5650 -62.4370

C -11.8680 17.0560 -63.7400

C -10.7520 16.4850 -64.3690

C -9.5370 16.4360 -63.6890

H -8.6590 16.0050 -64.1390

C -12.7290 19.0210 -60.8270

H -12.5970 18.4410 -59.9050

H -11.8400 19.6490 -60.9660

C -13.9870 19.8930 -60.7580

H -14.4850 19.8250 -61.7270

H -14.6690 19.4940 -60.0080

C -13.6840 21.3580 -60.4450

H -13.0200 21.4150 -59.5790

H -14.6180 21.8580 -60.1760

C -13.0560 22.0960 -61.6250

H -12.1290 21.6040 -61.9240

H -13.7380 22.0490 -62.4770

C -12.7620 23.5570 -61.2970

H -12.0970 23.6080 -60.4310

H -13.6930 24.0600 -61.0260

C -12.1160 24.2910 -62.4700

H -11.1620 23.8180 -62.7130

H -12.7580 24.2030 -63.3490

C -11.8870 25.7680 -62.1600

H -11.2600 25.8570 -61.2690

H -12.8470 26.2390 -61.9330

C -11.2260 26.5110 -63.3180

H -10.2470 26.0700 -63.5190

H -11.8340 26.3910 -64.2180

C -11.0620 27.9980 -63.0150

H -10.4730 28.1170 -62.1010

H -12.0450 28.4370 -62.8310

C -10.3800 28.7500 -64.1540

H -10.9560 28.6120 -65.0720

H -9.3850 28.3300 -64.3170

C -10.2550 30.2430 -63.8600

H -11.2520 30.6610 -63.7000

H -9.6870 30.3820 -62.9370

C -9.5700 30.9950 -64.9950

H -10.1280 30.8770 -65.9210

H -9.5050 32.0560 -64.7670

H -8.5620 30.6150 -65.1500

C -13.1770 18.0140 -65.4530

H -12.4650 17.7230 -66.2350

H -14.1910 17.8860 -65.8400

C -12.9400 19.4680 -65.0410

H -13.6990 19.7550 -64.3110

H -11.9660 19.5390 -64.5470

C -12.9710 20.4150 -66.2360

H -13.9350 20.3300 -66.7440

H -12.1960 20.1220 -66.9470

C -12.7450 21.8660 -65.8160

H -13.5520 22.1790 -65.1490

H -11.8100 21.9390 -65.2540

C -12.6840 22.8080 -67.0150

H -13.6220 22.7460 -67.5720

H -11.8820 22.4850 -67.6830

C -12.4360 24.2560 -66.6010

H -13.2430 24.5880 -65.9430

H -11.5030 24.3130 -66.0340

C -12.3510 25.1900 -67.8050

H -13.2990 25.1690 -68.3480

H -11.5760 24.8280 -68.4860

C -12.0290 26.6250 -67.3990

H -12.8000 26.9890 -66.7140

H -11.0760 26.6410 -66.8650

C -11.9450 27.5600 -68.6020

H -12.9050 27.5670 -69.1240

H -11.1960 27.1810 -69.3020

C -11.5790 28.9860 -68.1970

H -12.3280 29.3660 -67.4980

H -10.6170 28.9750 -67.6790

C -11.4920 29.9230 -69.3980

H -12.4540 29.9360 -69.9160

H -10.7470 29.5420 -70.1010

C -11.1200 31.3430 -68.9870

H -10.1490 31.3580 -68.4960

H -11.0730 31.9940 -69.8570

H -11.8590 31.7460 -68.2970

C -9.9140 15.7930 -66.5310

H -8.9830 15.5100 -66.0340

H -10.2290 14.9600 -67.1670

C -9.6990 17.0360 -67.4000

H -8.9440 16.7960 -68.1510

H -10.6320 17.2590 -67.9230

C -9.2640 18.2650 -66.6060

H -10.0230 18.5030 -65.8580

H -8.3340 18.0560 -66.0710

C -9.0650 19.4750 -67.5150

H -9.9650 19.6220 -68.1190

H -8.2380 19.2820 -68.2020

C -8.7830 20.7490 -66.7230

H -7.8790 20.6140 -66.1250

H -9.6110 20.9330 -66.0340

C -8.6130 21.9590 -67.6370

H -9.5070 22.0710 -68.2560

H -7.7690 21.7880 -68.3100

C -8.3820 23.2510 -66.8570

H -9.2230 23.4190 -66.1810

H -7.4820 23.1480 -66.2450

C -8.2310 24.4520 -67.7850

H -9.1350 24.5550 -68.3890

H -7.3970 24.2750 -68.4690

C -7.9860 25.7530 -67.0250

H -7.0830 25.6530 -66.4170

H -8.8210 25.9380 -66.3440

C -7.8310 26.9400 -67.9720

H -6.9940 26.7510 -68.6500

H -8.7330 27.0340 -68.5810

C -7.5900 28.2500 -67.2280

H -6.6980 28.1540 -66.6040

H -8.4360 28.4470 -66.5660

C -7.4130 29.4210 -68.1870

H -8.2890 29.5290 -68.8220

H -7.2690 30.3500 -67.6390

H -6.5470 29.2630 -68.8270

O -13.0980 17.1210 -64.3550

O -12.8660 18.1490 -61.9320

O -10.9730 15.9650 -65.6100

O -7.0410 16.6920 -62.5060

C 0.4190 16.0430 -60.3590

C 0.3740 15.7350 -59.0100

H -0.5740 15.5590 -58.5340

C 1.5530 15.6260 -58.2860

C 2.8030 15.8020 -58.9120

C 2.8350 16.1610 -60.2690

C 1.6390 16.2780 -60.9730

H 1.6550 16.5140 -62.0250

C 0.4960 15.5340 -56.1300

H -0.1310 14.6340 -56.1220

H -0.0970 16.3840 -56.4880

C 1.0520 15.8380 -54.7390

H 1.8060 15.0820 -54.5150

H 0.2580 15.7660 -53.9950

C 1.6900 17.2290 -54.7220

H 2.1660 17.3990 -55.6900

H 0.9110 17.9850 -54.5960

C 2.7460 17.4000 -53.6340

H 3.5660 16.7040 -53.8240

H 2.3190 17.1550 -52.6600

C 3.2870 18.8300 -53.6150

H 3.4950 19.1480 -54.6400

H 2.5220 19.4990 -53.2150

C 4.5640 18.9690 -52.7880

H 5.3610 18.3860 -53.2570

H 4.3990 18.5590 -51.7900

C 5.0030 20.4270 -52.6710

H 5.0230 20.8790 -53.6660

H 4.2680 20.9760 -52.0780

C 6.3810 20.5710 -52.0280

H 7.1310 20.1140 -52.6780

H 6.3960 20.0320 -51.0780

C 6.7460 22.0330 -51.7840

H 6.6360 22.5940 -52.7160

H 6.0460 22.4620 -51.0620

C 8.1720 22.1950 -51.2640

H 8.3020 21.5850 -50.3670

H 8.8730 21.8280 -52.0170

C 8.5030 23.6490 -50.9350

H 7.8320 24.0020 -50.1490

H 8.3250 24.2670 -51.8190

C 9.9480 23.8160 -50.4830

H 10.1390 23.2240 -49.5910

H 10.1620 24.8580 -50.2560

H 10.6310 23.4890 -51.2640

C 5.0940 16.1910 -58.1950

H 5.4590 16.2660 -59.2220

H 5.7840 15.5630 -57.6230

C 5.0090 17.5750 -57.5480

H 4.5070 17.4750 -56.5840

H 4.4050 18.2370 -58.1740

C 6.4020 18.1630 -57.3450

H 6.9770 17.4940 -56.7000

H 6.9180 18.2150 -58.3060

C 6.3670 19.5520 -56.7130

H 5.8050 19.5110 -55.7780

H 5.8490 20.2460 -57.3800

C 7.7760 20.0700 -56.4310

H 8.2660 19.3990 -55.7220

H 8.3590 20.0520 -57.3550

C 7.7770 21.4860 -55.8640

H 7.1580 21.5170 -54.9650

H 7.3330 22.1700 -56.5920

C 9.1890 21.9540 -55.5210

H 9.6080 21.2950 -54.7570

H 9.8220 21.8730 -56.4070

C 9.2160 23.3930 -55.0120

H 8.5540 23.4830 -54.1490

H 8.8320 24.0590 -55.7890

C 10.6230 23.8310 -54.6180

H 10.9940 23.1760 -53.8240

H 11.2910 23.7150 -55.4740

C 10.6640 25.2780 -54.1370

H 9.9940 25.3960 -53.2830

H 10.3000 25.9340 -54.9320

C 12.0710 25.7110 -53.7330

H 12.4270 25.0670 -52.9250

H 12.7470 25.5820 -54.5810

C 12.1080 27.1630 -53.2750

H 11.7780 27.8230 -54.0750

H 13.1170 27.4500 -52.9880

H 11.4520 27.3100 -52.4190

C 4.1560 16.9190 -62.1370

H 3.4490 17.7490 -62.2560

H 3.9310 16.1500 -62.8900

C 5.5850 17.4330 -62.3000

H 5.7770 17.6280 -63.3560

H 6.2700 16.6520 -61.9650

C 5.8060 18.7030 -61.4800

H 5.4410 18.5340 -60.4660

H 5.2200 19.5160 -61.9150

C 7.2740 19.1180 -61.4170

H 7.8480 18.3390 -60.9100

H 7.6720 19.2130 -62.4300

C 7.4480 20.4420 -60.6770

H 6.9500 21.2360 -61.2390

H 6.9620 20.3760 -59.7010

C 8.9160 20.8120 -60.4780

H 9.4020 20.0460 -59.8690

H 9.4210 20.8300 -61.4470

C 9.0710 22.1720 -59.8020

H 8.5140 22.1730 -58.8620

H 8.6360 22.9430 -60.4420

C 10.5310 22.5160 -59.5190

H 10.9540 21.7650 -58.8460

H 11.0990 22.4780 -60.4510

C 10.6800 23.9000 -58.8920

H 10.2940 24.6520 -59.5840

H 10.0780 23.9500 -57.9820

C 12.1320 24.2250 -58.5520

H 12.7400 24.1570 -59.4570

H 12.5120 23.4830 -57.8450

C 12.2820 25.6190 -57.9480

H 11.9280 26.3630 -58.6650

H 11.6540 25.6950 -57.0580

C 13.7280 25.9240 -57.5740

H 14.0980 25.1950 -56.8550

H 13.8090 26.9140 -57.1320

H 14.3660 25.8870 -58.4540

O 3.8710 15.4880 -58.1250

O 1.6220 15.3320 -56.9660

O 4.0590 16.3630 -60.8410

O -1.9720 16.1460 -60.3760

C -4.6920 16.8890 -56.2640

C -5.7750 17.1460 -55.4130

C -3.4680 16.5970 -55.6520

C -5.5950 17.0890 -54.0480

H -6.7470 17.3810 -55.8220

C -3.3890 16.5820 -54.2730

H -2.5910 16.3820 -56.2390

H -6.4160 17.2660 -53.3670

H -2.4640 16.3600 -53.7580

C -4.5140 15.6190 -66.6180

C -3.5530 15.1050 -67.4960

C -5.7800 15.8700 -67.1570

C -3.8920 14.8660 -68.8150

H -2.5560 14.8850 -67.1440

C -6.0090 15.5940 -68.4920

H -6.5750 16.2710 -66.5490

H -3.1710 14.4600 -69.5120

H -6.9810 15.7670 -68.9360

C -8.1420 16.8460 -61.6330

H -8.1960 15.9880 -60.9490

H -8.0240 17.7640 -61.0390

C -0.8330 16.0770 -61.2010

H -0.8680 15.1630 -61.8190

H -0.8020 16.9430 -61.8770

C -0.7490 14.2750 -31.3640

C -0.9650 15.0090 -36.9750

C -1.0700 15.1320 -38.1740

C -1.3080 15.2620 -39.5530

C -0.2800 15.1030 -40.5010

C -2.6130 15.5290 -39.9700

H -3.3610 15.6300 -39.2000

O -1.6890 14.5940 -30.6480

O 0.3520 13.7260 -30.9070

C -3.0140 16.2540 -50.4520

C -2.3110 15.7670 -44.8350

C -2.1580 15.6500 -43.6400

C -1.9040 15.5130 -42.2610

C -2.9390 15.6380 -41.3110

C -0.5980 15.2430 -41.8420

H 0.1550 15.1440 -42.6090

O -2.1640 15.9080 -51.2460

O -4.1750 16.7690 -50.8210

C -6.5810 16.0670 -41.5240

C -7.6680 16.4760 -40.7660

H -7.5330 16.6760 -39.7150

C -8.9100 16.6170 -41.3620

C -9.0920 16.3230 -42.7270

C -7.9860 15.9130 -43.4850

C -6.7410 15.7880 -42.8660

H -5.8750 15.4750 -43.4240

C -9.9050 17.5590 -39.4080

H -9.7060 16.7270 -38.7170

H -9.0710 18.2710 -39.3400

C -11.2150 18.2530 -39.0380

H -12.0340 17.6770 -39.4700

H -11.3210 18.2360 -37.9520

C -11.3020 19.7020 -39.5180

H -10.5040 20.2840 -39.0490

H -12.2530 20.1160 -39.1750

C -11.2120 19.8570 -41.0350

H -10.2210 19.5580 -41.3770

H -11.9350 19.1910 -41.5100

C -11.4820 21.3000 -41.4560

H -10.8070 21.9650 -40.9110

H -12.5030 21.5720 -41.1760

C -11.2970 21.5250 -42.9550

H -10.2620 21.3000 -43.2260

H -11.9420 20.8380 -43.5070

C -11.6230 22.9610 -43.3590

H -11.0160 23.6490 -42.7660

H -12.6710 23.1690 -43.1320

C -11.3670 23.2190 -44.8420

H -10.3100 23.0480 -45.0590

H -11.9470 22.5110 -45.4380

C -11.7380 24.6430 -45.2520

H -11.1830 25.3530 -44.6330

H -12.8020 24.8070 -45.0650

C -11.4360 24.9140 -46.7230

H -11.9710 24.1890 -47.3410

H -10.3670 24.7770 -46.9030

C -11.8370 26.3260 -47.1460

H -12.9080 26.4600 -46.9810

H -11.3130 27.0520 -46.5200

C -11.5120 26.5930 -48.6100

H -12.0290 25.8830 -49.2510

H -11.8160 27.5980 -48.8950

H -10.4430 26.4970 -48.7870

C -10.7050 16.7900 -44.4750

H -10.3580 16.0370 -45.1890

H -11.8000 16.8050 -44.4820

C -10.1730 18.1690 -44.8640

H -10.3490 18.8600 -44.0380

H -9.0960 18.1060 -45.0270

C -10.8630 18.6790 -46.1260

H -11.9070 18.9060 -45.9000

H -10.8590 17.8890 -46.8820

C -10.1850 19.9210 -46.6970

H -10.1260 20.6900 -45.9250

H -9.1660 19.6690 -46.9950

C -10.9430 20.4700 -47.9030

H -11.9270 20.8200 -47.5830

H -11.1030 19.6640 -48.6250

C -10.1970 21.6110 -48.5880

H -10.0090 22.4060 -47.8630

H -9.2300 21.2470 -48.9410

C -10.9830 22.1770 -49.7670

H -11.9300 22.5880 -49.4060

H -11.2230 21.3690 -50.4620

C -10.2100 23.2640 -50.5080

H -9.9510 24.0620 -49.8090

H -9.2790 22.8430 -50.8930

C -11.0150 23.8500 -51.6650

H -11.9320 24.3000 -51.2780

H -11.3080 23.0470 -52.3450

C -10.2260 24.9010 -52.4410

H -9.9110 25.6930 -51.7580

H -9.3250 24.4400 -52.8530

C -11.0430 25.5120 -53.5760

H -11.9220 26.0100 -53.1610

H -11.3960 24.7160 -54.2360

C -10.2290 26.5130 -54.3870

H -9.3650 26.0270 -54.8340

H -10.8320 26.9430 -55.1840

H -9.8760 27.3210 -53.7500

C -7.0680 15.2540 -45.5970

H -6.2200 15.9280 -45.4320

H -6.7700 14.2380 -45.3110

C -7.4420 15.2850 -47.0730

H -6.6090 14.8350 -47.6140

H -8.3310 14.6750 -47.2320

C -7.6860 16.7070 -47.5730

H -8.6360 17.0710 -47.1790

H -6.8990 17.3620 -47.1910

C -7.6980 16.7920 -49.0960

H -8.4390 16.0950 -49.4950

H -6.7210 16.4930 -49.4810

C -8.0170 18.2040 -49.5810

H -7.3540 18.9160 -49.0820

H -9.0430 18.4600 -49.3070

C -7.8430 18.3400 -51.0900

H -8.4560 17.5850 -51.5900

H -6.7970 18.1460 -51.3380

C -8.2410 19.7240 -51.5960

H -9.2740 19.9290 -51.3040

H -7.6080 20.4780 -51.1220

C -8.1200 19.8460 -53.1130

H -8.7320 19.0720 -53.5850

H -7.0830 19.6750 -53.4110

C -8.5700 21.2150 -53.6170

H -7.9280 21.9860 -53.1860

H -9.5900 21.4070 -53.2770

C -8.5240 21.3130 -55.1400

H -7.5060 21.1130 -55.4860

H -9.1750 20.5470 -55.5700

C -8.9680 22.6860 -55.6380

H -8.2990 23.4480 -55.2340

H -9.9720 22.8960 -55.2630

C -8.9700 22.7720 -57.1590

H -9.6490 22.0350 -57.5830

H -9.2880 23.7590 -57.4870

H -7.9730 22.5840 -57.5540

O -10.3880 16.4260 -43.1470

O -10.0260 17.0610 -40.7220

O -8.1770 15.6600 -44.8130

O -4.1950 15.8410 -41.7830

C 3.3560 14.5990 -40.1770

C 3.4170 14.3460 -38.8180

H 2.5080 14.3600 -38.2420

C 4.6440 14.0790 -38.2230

C 5.8280 14.0740 -38.9840

C 5.7600 14.3880 -40.3500

C 4.5200 14.6340 -40.9310

H 4.4470 14.8300 -41.9890

C 3.7250 13.7850 -36.0380

H 2.9850 13.0660 -36.4080

H 3.2660 14.7830 -35.9870

C 4.2180 13.3500 -34.6600

H 4.9540 12.5600 -34.8090

H 3.3740 12.9310 -34.1100

C 4.8280 14.4830 -33.8350

H 4.0610 15.2370 -33.6400

H 5.1400 14.0750 -32.8700

C 6.0290 15.1460 -34.5060

H 5.7020 15.6630 -35.4090

H 6.7440 14.3800 -34.8110

C 6.7040 16.1450 -33.5680

H 5.9530 16.8410 -33.1850

H 7.1200 15.6110 -32.7100

C 7.8130 16.9400 -34.2520

H 7.3860 17.5140 -35.0780

H 8.5510 16.2510 -34.6710

C 8.5030 17.8940 -33.2790

H 7.7490 18.5260 -32.8040

H 8.9890 17.3140 -32.4910

C 9.5390 18.7840 -33.9620

H 9.0450 19.3920 -34.7240

H 10.2820 18.1600 -34.4650

C 10.2410 19.7020 -32.9630

H 9.4900 20.2860 -32.4240

H 10.7730 19.0940 -32.2280

C 11.2250 20.6540 -33.6370

H 11.9660 20.0760 -34.1950

H 10.6870 21.2810 -34.3520

C 11.9400 21.5470 -32.6260

H 12.4960 20.9220 -31.9240

H 11.1970 22.1060 -32.0510

C 12.8960 22.5230 -33.3000

H 13.6480 21.9860 -33.8730

H 13.4020 23.1390 -32.5600

H 12.3530 23.1800 -33.9780

C 8.2010 14.2430 -38.5250

H 8.5450 14.0780 -39.5500

H 8.8590 13.6980 -37.8410

C 8.2380 15.7330 -38.1890

H 7.7770 15.8830 -37.2110

H 7.6490 16.2860 -38.9250

C 9.6760 16.2410 -38.1730

H 10.2330 15.7050 -37.4010

H 10.1490 16.0170 -39.1330

C 9.7710 17.7400 -37.9030

H 9.2520 17.9740 -36.9720

H 9.2720 18.2900 -38.7060

C 11.2250 18.1930 -37.8000

H 11.7020 17.6690 -36.9680

H 11.7570 17.9110 -38.7120

C 11.3600 19.6980 -37.5850

H 10.7960 19.9880 -36.6960

H 10.9260 20.2280 -38.4370

C 12.8190 20.1120 -37.4160

H 13.2360 19.6030 -36.5430

H 13.3910 19.7880 -38.2880

C 12.9790 21.6200 -37.2400

H 12.3810 21.9480 -36.3870

H 12.5950 22.1310 -38.1270

C 14.4360 22.0150 -37.0170

H 14.8090 21.5200 -36.1170

H 15.0390 21.6620 -37.8570

C 14.6090 23.5240 -36.8700

H 13.9890 23.8800 -36.0440

H 14.2590 24.0180 -37.7800

C 16.0620 23.9130 -36.6100

H 16.3980 23.4470 -35.6810

H 16.6900 23.5300 -37.4180

C 16.2360 25.4240 -36.5110

H 15.9290 25.9040 -37.4380

H 17.2750 25.6790 -36.3210

H 15.6290 25.8250 -35.7020

C 6.9460 14.7420 -42.4190

H 6.3500 15.6440 -42.6090

H 6.5280 13.9090 -43.0010

C 8.4010 15.0020 -42.8040

H 8.4890 15.0000 -43.8920

H 9.0120 14.1880 -42.4100

C 8.8780 16.3370 -42.2370

H 8.6250 16.3760 -41.1760

H 8.3410 17.1470 -42.7370

C 10.3790 16.5580 -42.3980

H 10.9220 15.8130 -41.8110

H 10.6620 16.4230 -43.4450

C 10.7760 17.9590 -41.9390

H 10.3050 18.6960 -42.5950

H 10.3940 18.1270 -40.9300

C 12.2860 18.1820 -41.9410

H 12.7550 17.4960 -41.2310

H 12.6890 17.9560 -42.9310

C 12.6360 19.6200 -41.5660

H 12.1640 19.8680 -40.6130

H 12.2270 20.2970 -42.3210

C 14.1410 19.8480 -41.4510

H 14.5440 19.2070 -40.6620

H 14.6260 19.5600 -42.3860

C 14.4690 21.3050 -41.1370

H 14.1160 21.9380 -41.9550

H 13.9350 21.6070 -40.2340

C 15.9650 21.5330 -40.9350

H 16.5060 21.2100 -41.8270

H 16.3140 20.9190 -40.1000

C 16.2870 22.9980 -40.6510

H 15.9730 23.6080 -41.5020

H 15.7170 23.3290 -39.7810

C 17.7730 23.2160 -40.3940

H 18.1040 22.6220 -39.5430

H 17.9770 24.2630 -40.1820

H 18.3590 22.9200 -41.2620

O 6.9370 13.6520 -38.3040

O 4.8340 13.8000 -36.9110

O 6.9360 14.4150 -41.0440

O 0.9580 14.8120 -40.0070

C -0.8850 14.8410 -35.5810

C 0.1940 14.1660 -35.0020

C -1.9090 15.3330 -34.7640

C 0.2520 13.9950 -33.6370

H 0.9710 13.7720 -35.6370

C -1.8530 15.1500 -33.4020

H -2.7410 15.8510 -35.2170

C -0.7710 14.4830 -32.8260

H 1.0830 13.4800 -33.1810

H -2.6360 15.5160 -32.7560

C -2.4930 15.8960 -46.2280

C -1.5560 15.3500 -47.1120

C -3.6020 16.5740 -46.7400

C -1.7350 15.4760 -48.4720

H -0.7000 14.8250 -46.7170

C -3.7780 16.6940 -48.1010

H -4.3120 17.0100 -46.0530

C -2.8480 16.1440 -48.9800

H -1.0250 15.0550 -49.1680

H -4.6340 17.2140 -48.4990

C -5.2470 15.9170 -40.8400

H -5.2680 15.0070 -40.2210

H -5.0880 16.7770 -40.1730

C 2.0480 14.8210 -40.9000

H 1.9250 14.0250 -41.6520

H 2.0870 15.7840 -41.4320

N -4.4260 16.8230 -53.4840

N -5.0940 15.1040 -69.3130

H -4.2530 16.7890 -51.8300

H 0.3030 13.5390 -29.9190

H -3.0260 16.2800 -58.3520

H -5.9090 17.2240 -57.9790

H -3.1340 15.6060 -64.9900

H -6.0470 16.3990 -64.5030

C -5.2030 -6.0410 -46.4860

C -4.7230 -6.2100 -52.1310

C -4.6900 -6.3030 -53.3360

C -4.5540 -6.4400 -54.7280

C -5.6570 -6.2610 -55.5880

C -3.3000 -6.7610 -55.2490

H -2.4930 -6.8760 -54.5440

O -4.2470 -5.9880 -45.7370

O -6.4490 -6.1470 -46.0700

C -4.4450 -6.4710 -65.7160

C -4.1470 -6.8790 -60.0770

C -4.0980 -6.8540 -58.8680

C -4.1990 -6.7470 -57.4690

C -3.0990 -6.9300 -56.6100

C -5.4520 -6.4150 -56.9480

H -6.2510 -6.2730 -57.6570

O -5.4960 -6.2310 -66.2960

O -3.2930 -6.5970 -66.3320

C 0.3780 -8.0360 -57.0770

C 1.5400 -8.2730 -56.3520

H 1.5430 -8.1050 -55.2870

C 2.6810 -8.7120 -57.0040

C 2.6780 -8.9160 -58.3990

C 1.4910 -8.7040 -59.1120

C 0.3520 -8.2650 -58.4370

H -0.5610 -8.0700 -58.9720

C 4.0040 -8.7980 -55.0100

H 3.8030 -7.7510 -54.7420

H 3.2900 -9.4380 -54.4730

C 5.4370 -9.1650 -54.6290

H 6.1000 -8.7380 -55.3820

H 5.6660 -8.7010 -53.6670

C 5.6830 -10.6700 -54.5240

H 5.0410 -11.0870 -53.7440

H 6.7190 -10.8230 -54.2110

C 5.4450 -11.4250 -55.8310

H 4.3840 -11.4000 -56.0810

H 5.9800 -10.9250 -56.6400

C 5.9080 -12.8770 -55.7250

H 5.4300 -13.3450 -54.8610

H 6.9870 -12.9000 -55.5530

C 5.5820 -13.6910 -56.9750

H 4.4980 -13.7200 -57.1130

H 6.0150 -13.2020 -57.8510

C 6.1170 -15.1180 -56.8770

H 5.7310 -15.5850 -55.9670

H 7.2060 -15.0870 -56.7930

C 5.7290 -15.9750 -58.0800

H 4.6400 -16.0450 -58.1360

H 6.0790 -15.4950 -58.9970

C 6.3190 -17.3810 -57.9890

H 6.0030 -17.8440 -57.0510

H 7.4100 -17.3120 -57.9690

C 5.8920 -18.2690 -59.1550

H 6.1850 -17.7980 -60.0960

H 4.8030 -18.3630 -59.1560

C 6.5150 -19.6600 -59.0730

H 7.6030 -19.5680 -59.0900

H 6.2380 -20.1230 -58.1230

C 6.0650 -20.5560 -60.2210

H 6.3380 -20.1150 -61.1780

H 6.5320 -21.5360 -60.1500

H 4.9860 -20.6890 -60.2000

C 4.0520 -10.0560 -60.0460

H 3.5370 -9.6430 -60.9170

H 5.1310 -10.0430 -60.2300

C 3.5990 -11.4940 -59.7990

H 3.9970 -11.8300 -58.8390

H 2.5080 -11.5290 -59.7410

C 4.1010 -12.4010 -60.9180

H 5.1940 -12.3970 -60.9120

H 3.7790 -11.9980 -61.8810

C 3.6090 -13.8400 -60.7870

H 3.8720 -14.2250 -59.8000

H 2.5200 -13.8640 -60.8720

C 4.2290 -14.7300 -61.8610

H 5.3140 -14.7410 -61.7300

H 4.0230 -14.3050 -62.8460

C 3.7080 -16.1630 -61.8140

H 3.8700 -16.5760 -60.8170

H 2.6310 -16.1660 -62.0010

C 4.4070 -17.0450 -62.8460

H 5.4780 -17.0670 -62.6310

H 4.2830 -16.6070 -63.8390

C 3.8680 -18.4730 -62.8550

H 3.9570 -18.8970 -61.8530

H 2.8060 -18.4590 -63.1130

C 4.6200 -19.3560 -63.8470

H 5.6780 -19.3870 -63.5720

H 4.5560 -18.9160 -64.8450

C 4.0700 -20.7790 -63.8870

H 4.1160 -21.2130 -62.8850

H 3.0190 -20.7510 -64.1850

C 4.8460 -21.6680 -64.8550

H 5.8880 -21.7260 -64.5330

H 4.8330 -21.2180 -65.8500

C 4.2600 -23.0720 -64.9300

H 3.2280 -23.0360 -65.2750

H 4.8300 -23.6900 -65.6200

H 4.2760 -23.5460 -63.9510

C 0.3140 -8.8300 -61.1960

H -0.4800 -9.4380 -60.7410

H 0.0010 -7.7780 -61.2140

C 0.6110 -9.3270 -62.6080

H -0.1940 -9.0100 -63.2730

H 1.5400 -8.8610 -62.9410

C 0.7370 -10.8480 -62.6420

H 1.4200 -11.1610 -61.8510

H -0.2410 -11.2890 -62.4300

C 1.2470 -11.3800 -63.9790

H 2.2770 -11.0520 -64.1340

H 0.6450 -10.9720 -64.7940

C 1.1830 -12.9060 -64.0140

H 0.1360 -13.2190 -63.9910

H 1.6630 -13.3040 -63.1170

C 1.8580 -13.5070 -65.2430

H 2.9250 -13.2720 -65.2230

H 1.4400 -13.0610 -66.1480

C 1.6700 -15.0220 -65.2880

H 1.9900 -15.4510 -64.3360

H 0.6080 -15.2470 -65.4090

C 2.4550 -15.6830 -66.4180

H 3.5230 -15.5110 -66.2650

H 2.1820 -15.2240 -67.3710

C 2.1870 -17.1850 -66.4830

H 1.1310 -17.3520 -66.7090

H 2.3870 -17.6280 -65.5050

C 3.0440 -17.8860 -67.5330

H 2.8720 -17.4260 -68.5100

H 4.1000 -17.7480 -67.2870

C 2.7370 -19.3790 -67.6170

H 1.6940 -19.5170 -67.9100

H 2.8630 -19.8270 -66.6290

C 3.6420 -20.0930 -68.6140

H 4.6870 -19.9780 -68.3320

H 3.4100 -21.1550 -68.6510

H 3.5120 -19.6780 -69.6120

O 3.9060 -9.2200 -58.9150

O 3.8720 -8.9670 -56.4040

O 1.5100 -8.9480 -60.4530

O -1.9070 -7.2550 -57.1850

C -9.2320 -5.5830 -55.0960

C -9.2530 -5.3700 -53.7280

H -8.3260 -5.3740 -53.1800

C -10.4700 -5.1500 -53.0980

C -11.6840 -5.1580 -53.8200

C -11.6480 -5.4100 -55.1980

C -10.4150 -5.6010 -55.8130

H -10.3620 -5.7500 -56.8790

C -9.5340 -5.0660 -50.8930

H -8.7040 -4.4020 -51.1700

H -9.1880 -6.1090 -50.9380

C -9.9740 -4.7160 -49.4750

H -10.3370 -3.6880 -49.4730

H -9.0770 -4.7530 -48.8570

C -11.0520 -5.6440 -48.9110

H -10.8550 -6.6690 -49.2340

H -10.9980 -5.6290 -47.8190

C -12.4590 -5.2320 -49.3420

H -12.4950 -5.1720 -50.4300

H -12.6730 -4.2370 -48.9450

C -13.5250 -6.2070 -48.8470

H -13.3590 -7.1870 -49.3030

H -13.4340 -6.3330 -47.7640

C -14.9350 -5.7250 -49.1820

H -14.9950 -5.5190 -50.2520

H -15.1300 -4.7890 -48.6530

C -16.0010 -6.7510 -48.8060

H -15.7850 -7.6950 -49.3120

H -15.9610 -6.9400 -47.7310

C -17.4070 -6.2930 -49.1870

H -17.4370 -6.0810 -50.2580

H -17.6420 -5.3640 -48.6600

C -18.4590 -7.3470 -48.8540

H -18.1980 -8.2830 -49.3530

H -18.4530 -7.5360 -47.7770

C -19.8630 -6.9290 -49.2840

H -20.1300 -5.9890 -48.7950

H -19.8720 -6.7520 -50.3620

C -20.9060 -7.9910 -48.9410

H -20.9380 -8.1270 -47.8570

H -20.6080 -8.9440 -49.3830

C -22.2930 -7.6120 -49.4450

H -22.6160 -6.6710 -49.0050

H -23.0190 -8.3790 -49.1840

H -22.2890 -7.4980 -50.5270

C -14.0820 -5.1790 -53.3850

H -14.2890 -4.9710 -54.4350

H -14.6890 -4.5140 -52.7610

C -14.4380 -6.6300 -53.0560

H -14.1130 -6.8520 -52.0380

H -13.9030 -7.3030 -53.7290

C -15.9460 -6.8360 -53.1780

H -16.4530 -6.0880 -52.5620

H -16.2540 -6.6680 -54.2120

C -16.3970 -8.2240 -52.7330

H -16.0800 -8.3930 -51.7020

H -15.9190 -8.9850 -53.3540

C -17.9150 -8.3650 -52.8230

H -18.3790 -7.5840 -52.2170

H -18.2340 -8.2120 -53.8560

C -18.4090 -9.7250 -52.3390

H -18.0840 -9.8810 -51.3070

H -17.9590 -10.5160 -52.9450

C -19.9300 -9.8280 -52.4110

H -20.3710 -9.0180 -51.8260

H -20.2530 -9.6990 -53.4460

C -20.4460 -11.1640 -51.8850

H -20.1080 -11.3040 -50.8550

H -20.0220 -11.9770 -52.4800

C -21.9700 -11.2450 -51.9300

H -22.3920 -10.4490 -51.3110

H -22.3080 -11.0780 -52.9550

C -22.4890 -12.5940 -51.4420

H -22.1590 -12.7590 -50.4130

H -22.0580 -13.3900 -52.0550

C -24.0120 -12.6810 -51.5040

H -24.4430 -11.9090 -50.8620

H -24.3430 -12.4850 -52.5250

C -24.5210 -14.0490 -51.0650

H -24.1210 -14.8280 -51.7110

H -25.6070 -14.0880 -51.1130

H -24.2140 -14.2620 -50.0430

C -12.7930 -5.9220 -57.2420

H -12.2070 -6.8470 -57.3260

H -12.3270 -5.1490 -57.8700

C -14.2240 -6.1820 -57.7050

H -14.2170 -6.3020 -58.7890

H -14.8370 -5.3120 -57.4660

C -14.8240 -7.4240 -57.0510

H -14.6880 -7.3550 -55.9710

H -14.2880 -8.3110 -57.3990

C -16.3110 -7.5740 -57.3650

H -16.8550 -6.7230 -56.9490

H -16.4580 -7.5600 -58.4480

C -16.8780 -8.8710 -56.7940

H -16.3900 -9.7210 -57.2760

H -16.6510 -8.9230 -55.7280

C -18.3890 -8.9830 -56.9880

H -18.8810 -8.1500 -56.4790

H -18.6270 -8.9040 -58.0520

C -18.9320 -10.3010 -56.4430

H -18.6460 -10.4010 -55.3940

H -18.4750 -11.1310 -56.9880

C -20.4510 -10.3980 -56.5610

H -20.9090 -9.5710 -56.0120

H -20.7410 -10.2950 -57.6090

C -20.9810 -11.7220 -56.0150

H -20.5410 -12.5470 -56.5800

H -20.6690 -11.8330 -54.9750

C -22.5030 -11.8120 -56.0950

H -22.8180 -11.7060 -57.1360

H -22.9430 -10.9830 -55.5340

C -23.0300 -13.1320 -55.5400

H -22.5960 -13.9600 -56.1060

H -22.7080 -13.2390 -54.5020

C -24.5500 -13.2130 -55.6100

H -25.0020 -12.3940 -55.0520

H -24.9040 -14.1510 -55.1890

H -24.8890 -13.1480 -56.6410

O -12.7570 -4.8410 -53.0410

O -10.6280 -4.9010 -51.7740

O -12.8280 -5.4860 -55.8970

O -6.8420 -5.9390 -55.0130

C -4.8300 -6.1210 -50.7300

C -6.0910 -6.2080 -50.1360

C -3.6950 -5.9710 -49.9260

C -6.2160 -6.1660 -48.7660

H -6.9580 -6.3210 -50.7660

C -3.8260 -5.9250 -48.5550

H -2.7220 -5.9010 -50.3900

C -5.0830 -6.0330 -47.9650

H -7.1850 -6.2440 -48.3000

H -2.9640 -5.8160 -47.9150

C -4.2170 -6.8380 -61.4800

C -5.4210 -6.4730 -62.0970

C -3.1010 -7.1310 -62.2700

C -5.4950 -6.3730 -63.4670

H -6.2820 -6.2580 -61.4810

C -3.1820 -7.0330 -63.6400

H -2.1800 -7.4270 -61.7920

C -4.3710 -6.6370 -64.2510

H -6.4120 -6.0830 -63.9580

H -2.3330 -7.2600 -64.2650

C -0.8280 -7.5410 -56.3220

H -0.5490 -6.6350 -55.7610

H -1.1290 -8.3110 -55.5950

C -7.9570 -5.8040 -55.8680

H -7.8050 -4.9570 -56.5560

H -8.0710 -6.7150 -56.4740

C -4.2540 -6.3940 -31.9720

C -5.3030 -6.6870 -32.7560

C -5.3160 -6.7740 -34.1990

C -6.5660 -6.7940 -34.8600

C -4.1530 -6.8460 -34.9680

H -3.2070 -6.8590 -34.4520

C -6.5290 -6.6160 -39.2270

C -5.4640 -6.8820 -38.4550

C -5.4390 -6.8810 -37.0110

C -4.1910 -6.9120 -36.3500

C -6.6010 -6.8370 -36.2420

H -7.5460 -6.8500 -36.7560

C -0.7790 -7.4990 -37.5970

C 0.4280 -8.0410 -37.1710

H 0.5420 -8.3270 -36.1380

C 1.4700 -8.2000 -38.0710

C 1.3080 -7.8090 -39.4130

C 0.0840 -7.2680 -39.8380

C -0.9540 -7.1300 -38.9180

H -1.8970 -6.7010 -39.2090

C 3.0270 -8.9400 -36.4140

H 2.8420 -8.0310 -35.8260

H 2.4180 -9.7560 -35.9990

C 4.5110 -9.3000 -36.3550

H 5.0360 -8.6410 -37.0450

H 4.8740 -9.1000 -35.3450

C 4.8090 -10.7560 -36.7120

H 4.3390 -11.4140 -35.9760

H 5.8890 -10.9100 -36.6480

C 4.3310 -11.1390 -38.1110

H 3.2410 -11.1210 -38.1370

H 4.6850 -10.3970 -38.8280

C 4.8170 -12.5270 -38.5250

H 4.5590 -13.2500 -37.7470

H 5.9060 -12.5200 -38.6200

C 4.1920 -12.9670 -39.8460

H 3.1070 -13.0170 -39.7220

H 4.4030 -12.2180 -40.6130

C 4.7010 -14.3270 -40.3180

H 4.5520 -15.0650 -39.5270

H 5.7740 -14.2660 -40.5150

C 3.9760 -14.7880 -41.5810

H 2.9080 -14.8720 -41.3650

H 4.0950 -14.0330 -42.3610

C 4.4850 -16.1320 -42.0950

H 4.3960 -16.8790 -41.3030

H 5.5440 -16.0460 -42.3500

C 3.7030 -16.6000 -43.3200

H 3.7870 -15.8500 -44.1100

H 2.6460 -16.6850 -43.0590

C 4.1960 -17.9450 -43.8470

H 5.2470 -17.8580 -44.1340

H 4.1330 -18.6900 -43.0500

C 3.3790 -18.4160 -45.0440

H 3.4370 -17.6920 -45.8540

H 3.7500 -19.3710 -45.4110

H 2.3350 -18.5360 -44.7680

C 2.2710 -8.6410 -41.4330

H 1.6430 -8.0850 -42.1380

H 3.2880 -8.6940 -41.8310

C 1.7060 -10.0440 -41.2150

H 2.3470 -10.5730 -40.5070

H 0.7160 -9.9550 -40.7610

C 1.5970 -10.8360 -42.5130

H 2.5930 -10.9850 -42.9350

H 1.0120 -10.2660 -43.2380

C 0.9350 -12.1920 -42.2780

H 1.5100 -12.7420 -41.5310

H -0.0680 -12.0350 -41.8750

C 0.8380 -13.0320 -43.5480

H 1.8390 -13.1830 -43.9590

H 0.2510 -12.4950 -44.2970

C 0.1970 -14.3890 -43.2730

H 0.7940 -14.9200 -42.5280

H -0.7990 -14.2400 -42.8500

C 0.0870 -15.2500 -44.5280

H 1.0730 -15.3480 -44.9880

H -0.5660 -14.7570 -45.2520

C -0.4590 -16.6410 -44.2150

H 0.2220 -17.1450 -43.5240

H -1.4240 -16.5480 -43.7120

C -0.6230 -17.4960 -45.4690

H 0.3240 -17.5250 -46.0140

H -1.3640 -17.0350 -46.1270

C -1.0550 -18.9210 -45.1350

H -0.2990 -19.3870 -44.4980

H -1.9890 -18.8940 -44.5680

C -1.2500 -19.7740 -46.3860

H -0.3230 -19.7800 -46.9650

H -2.0250 -19.3250 -47.0120

C -1.6400 -21.2060 -46.0410

H -2.5630 -21.2240 -45.4660

H -1.7880 -21.7920 -46.9460

H -0.8600 -21.6800 -45.4490

C -1.1330 -6.4990 -41.8140

H -1.9360 -6.2230 -41.1290

H -0.8650 -5.6100 -42.3930

C -1.5810 -7.6020 -42.7750

H -2.3150 -7.1810 -43.4650

H -0.7130 -7.9110 -43.3610

C -2.1760 -8.8220 -42.0780

H -1.4670 -9.2080 -41.3410

H -3.0880 -8.5390 -41.5480

C -2.4960 -9.9130 -43.0960

H -1.5780 -10.1880 -43.6210

H -3.1920 -9.5130 -43.8380

C -3.0970 -11.1670 -42.4670

H -4.0450 -10.9170 -41.9840

H -2.4240 -11.5460 -41.6940

C -3.3250 -12.2510 -43.5170

H -2.3790 -12.4580 -44.0230

H -4.0240 -11.8790 -44.2690

C -3.8650 -13.5500 -42.9260

H -3.1680 -13.9230 -42.1700

H -4.8170 -13.3580 -42.4270

C -4.0570 -14.6140 -44.0050

H -3.1140 -14.7590 -44.5360

H -4.7920 -14.2590 -44.7310

C -4.5190 -15.9520 -43.4340

H -5.4560 -15.8120 -42.8890

H -3.7770 -16.3170 -42.7190

C -4.7210 -16.9950 -44.5310

H -5.4940 -16.6470 -45.2200

H -3.7950 -17.1000 -45.1020

C -5.1210 -18.3570 -43.9710

H -6.0350 -18.2520 -43.3830

H -4.3370 -18.7160 -43.2990

C -5.3470 -19.3780 -45.0800

H -4.4470 -19.4940 -45.6800

H -5.6090 -20.3480 -44.6630

H -6.1540 -19.0570 -45.7350

O 2.4140 -7.9240 -40.2180

O 2.6890 -8.7060 -37.7660

O 0.0540 -6.8550 -41.1320

O -3.0870 -6.9830 -37.1500

C -10.0280 -6.6440 -33.6590

C -9.7520 -6.5370 -32.3070

H -8.7340 -6.6060 -31.9680

C -10.7840 -6.2970 -31.4150

C -12.1190 -6.1760 -31.8590

C -12.3950 -6.3540 -33.2230

C -11.3370 -6.5750 -34.1010

H -11.5260 -6.6830 -35.1570

C -9.4170 -6.5700 -29.4760

H -8.5820 -5.9230 -29.7770

H -9.1960 -7.6010 -29.7910

C -9.5740 -6.5070 -27.9580

H -9.8060 -5.4810 -27.6750

H -8.6060 -6.7630 -27.5240

C -10.6510 -7.4510 -27.4180

H -10.6350 -8.3820 -27.9900

H -10.4160 -7.7010 -26.3800

C -12.0540 -6.8470 -27.4740

H -12.2510 -6.4840 -28.4820

H -12.0980 -5.9920 -26.7940

C -13.1230 -7.8620 -27.0780

H -13.1380 -8.6710 -27.8140

H -12.8710 -8.3060 -26.1110

C -14.5120 -7.2330 -26.9950

H -14.7110 -6.6820 -27.9160

H -14.5350 -6.5140 -26.1720

C -15.6030 -8.2800 -26.7840

H -15.5750 -8.9970 -27.6090

H -15.4010 -8.8330 -25.8640

C -16.9970 -7.6620 -26.7050

H -17.1860 -7.0830 -27.6120

H -17.0400 -6.9730 -25.8590

C -18.0840 -8.7230 -26.5510

H -18.0410 -9.4070 -27.4020

H -17.8910 -9.3100 -25.6490

C -19.4820 -8.1150 -26.4660

H -19.5360 -7.4520 -25.5990

H -19.6660 -7.5090 -27.3560

C -20.5670 -9.1840 -26.3540

H -20.3820 -9.7950 -25.4680

H -20.5140 -9.8410 -27.2250

C -21.9610 -8.5750 -26.2660

H -22.0470 -7.9440 -25.3840

H -22.7170 -9.3550 -26.2050

H -22.1680 -7.9630 -27.1420

C -14.3710 -5.7640 -31.0080

H -14.6380 -5.2540 -31.9350

H -14.6910 -5.1450 -30.1630

C -15.0620 -7.1250 -30.9300

H -14.6960 -7.6550 -30.0490

H -14.7980 -7.7130 -31.8100

C -16.5740 -6.9450 -30.8410

H -16.8060 -6.3120 -29.9810

H -16.9280 -6.4250 -31.7330

C -17.3190 -8.2690 -30.6960

H -16.9680 -8.7860 -29.8010

H -17.1000 -8.9100 -31.5540

C -18.8250 -8.0440 -30.5940

H -19.0310 -7.4070 -29.7310

H -19.1700 -7.5110 -31.4830

C -19.6100 -9.3440 -30.4520

H -19.2640 -9.8820 -29.5670

H -19.4190 -9.9830 -31.3190

C -21.1080 -9.0800 -30.3330

H -21.2910 -8.4480 -29.4610

H -21.4470 -8.5300 -31.2140

C -21.9220 -10.3640 -30.1990

H -21.5800 -10.9230 -29.3260

H -21.7530 -10.9930 -31.0770

C -23.4140 -10.0740 -30.0610

H -23.5810 -9.4720 -29.1650

H -23.7460 -9.4860 -30.9200

C -24.2470 -11.3490 -29.9740

H -23.9180 -11.9410 -29.1160

H -24.0790 -11.9520 -30.8710

C -25.7380 -11.0490 -29.8400

H -25.9090 -10.4830 -28.9210

H -26.0550 -10.4240 -30.6770

C -26.5760 -12.3210 -29.8150

H -26.4400 -12.8860 -30.7360

H -27.6320 -12.0820 -29.7140

H -26.2850 -12.9550 -28.9800

C -13.9780 -6.5110 -35.0200

H -13.6900 -7.5320 -35.3090

H -13.4130 -5.7980 -35.6350

C -15.4690 -6.2910 -35.2570

H -15.6170 -6.1630 -36.3310

H -15.7610 -5.3610 -34.7680

C -16.3330 -7.4460 -34.7600

H -16.1000 -7.6480 -33.7140

H -16.0960 -8.3460 -35.3340

C -17.8210 -7.1330 -34.8980

H -18.0790 -6.3120 -34.2250

H -18.0270 -6.7990 -35.9180

C -18.6990 -8.3410 -34.5810

H -18.4980 -9.1330 -35.3060

H -18.4420 -8.7260 -33.5930

C -20.1860 -7.9960 -34.6120

H -20.3990 -7.2440 -33.8470

H -20.4380 -7.5560 -35.5800

C -21.0610 -9.2230 -34.3710

H -20.7730 -9.6900 -33.4270

H -20.8850 -9.9520 -35.1660

C -22.5470 -8.8780 -34.3250

H -22.7300 -8.1740 -33.5090

H -22.8340 -8.3820 -35.2560

C -23.4150 -10.1180 -34.1250

H -23.2510 -10.8090 -34.9560

H -23.1080 -10.6270 -33.2090

C -24.9000 -9.7780 -34.0380

H -25.2060 -9.2500 -34.9450

H -25.0670 -9.1040 -33.1940

C -25.7640 -11.0250 -33.8640

H -25.6110 -11.6900 -34.7180

H -25.4450 -11.5600 -32.9680

C -27.2440 -10.6820 -33.7480

H -27.4200 -10.0320 -32.8930

H -27.8380 -11.5840 -33.6200

H -27.5880 -10.1670 -34.6430

O -12.9720 -5.8490 -30.8480

O -10.6200 -6.1370 -30.0770

O -13.7000 -6.3000 -33.6500

O -7.6710 -6.7630 -34.0730

C -4.2950 -6.3190 -30.5240

C -3.1320 -6.0040 -29.8140

C -5.4540 -6.5270 -29.7690

C -3.1860 -5.8970 -28.4370

H -2.2020 -5.8300 -30.3370

C -5.3890 -6.4080 -28.3940

H -6.3870 -6.7880 -30.2410

H -2.3070 -5.6370 -27.8630

H -6.2690 -6.5630 -27.7820

C -6.5120 -6.5360 -40.6710

C -7.7210 -6.5550 -41.3770

C -5.3400 -6.3910 -41.4200

C -7.7010 -6.4510 -42.7510

H -8.6560 -6.6540 -40.8480

C -5.4280 -6.2870 -42.7920

H -4.3790 -6.3290 -40.9380

H -8.6120 -6.4670 -43.3340

H -4.5530 -6.1650 -43.4160

C -1.8420 -7.2600 -36.5520

H -1.5260 -6.4090 -35.9260

H -1.9240 -8.1460 -35.9060

C -8.9390 -6.8050 -34.6880

H -9.0300 -5.9870 -35.4180

H -9.0670 -7.7620 -35.2180

N -4.2860 -6.0990 -27.7320

N -6.5810 -6.3230 -43.4450

H -6.5040 -6.1840 -45.0560

H -3.3660 -6.4320 -67.3240

H -3.2990 -6.1350 -32.4110

H -6.2720 -6.8340 -32.3050

H -4.5120 -7.0990 -38.9180

H -7.4930 -6.4230 -38.7770

C -6.8590 -0.3500 -32.9560

C -5.8470 -0.4460 -33.8340

C -5.9320 -0.4250 -35.2750

C -4.7370 -0.5310 -36.0200

C -7.1340 -0.2900 -35.9770

H -8.0430 -0.2100 -35.4070

C -4.9000 -0.2520 -40.3190

C -5.9950 -0.2300 -39.5420

C -5.9880 -0.3120 -38.1000

C -7.1880 -0.2510 -37.3600

C -4.7940 -0.4510 -37.3970

H -3.8950 -0.4850 -37.9810

O -8.3240 -0.1430 -38.0930

C -1.1430 -1.1610 -35.4340

C -1.0880 -1.7040 -34.1660

H -2.0010 -1.8400 -33.6120

C 0.1450 -2.0740 -33.6380

C 1.3170 -1.9880 -34.4140

C 1.2470 -1.4190 -35.7010

C 0.0190 -0.9660 -36.1640

H -0.0570 -0.5020 -37.1330

C -0.7270 -2.3540 -31.4440

H -1.5500 -3.0650 -31.6140

H -1.1240 -1.3370 -31.5560

C -0.1810 -2.5300 -30.0290

H 0.2100 -3.5430 -29.9200

H -1.0250 -2.4150 -29.3460

C 0.9210 -1.5220 -29.6840

H 0.7170 -0.5740 -30.1880

H 0.9060 -1.3300 -28.6080

C 2.3100 -2.0190 -30.0810

H 2.2940 -2.3270 -31.1260

H 2.5530 -2.8980 -29.4780

C 3.3890 -0.9580 -29.8730

H 3.2280 -0.1310 -30.5700

H 3.3140 -0.5510 -28.8620

C 4.7860 -1.5420 -30.0780

H 4.8130 -2.0780 -31.0290

H 4.9850 -2.2710 -29.2880

C 5.8850 -0.4830 -30.0700

H 5.7290 0.2100 -30.9010

H 5.8280 0.0970 -29.1470

C 7.2700 -1.1160 -30.1850

H 7.3150 -1.7280 -31.0890

H 7.4280 -1.7830 -29.3340

C 8.3920 -0.0810 -30.2220

H 8.2500 0.5790 -31.0810

H 8.3440 0.5350 -29.3210

C 9.7670 -0.7410 -30.3080

H 9.9010 -1.4030 -29.4500

H 9.8170 -1.3600 -31.2080

C 10.9000 0.2810 -30.3350

H 10.8430 0.9030 -29.4400

H 10.7750 0.9320 -31.2040

C 12.2680 -0.3860 -30.3960

H 12.4350 -0.9980 -29.5110

H 13.0580 0.3600 -30.4510

H 12.3430 -1.0300 -31.2710

C 3.6830 -1.9230 -33.9340

H 3.7650 -1.2140 -33.0980

H 3.7840 -1.3670 -34.8680

C 4.7730 -2.9870 -33.8440

H 4.6000 -3.7250 -34.6280

H 4.6910 -3.5030 -32.8850

C 6.1630 -2.3770 -34.0010

H 6.2170 -1.8530 -34.9570

H 6.3250 -1.6380 -33.2140

C 7.2710 -3.4260 -33.9480

H 7.0920 -4.1820 -34.7170

H 7.2500 -3.9330 -32.9800

C 8.6460 -2.7980 -34.1640

H 8.6490 -2.2630 -35.1170

H 8.8340 -2.0640 -33.3780

C 9.7720 -3.8280 -34.1720

H 9.5820 -4.5670 -34.9540

H 9.7880 -4.3590 -33.2170

C 11.1320 -3.1750 -34.4120

H 11.1020 -2.6170 -35.3510

H 11.3340 -2.4580 -33.6120

C 12.2670 -4.1940 -34.4730

H 12.0630 -4.9130 -35.2710

H 12.3060 -4.7520 -33.5350

C 13.6180 -3.5290 -34.7230

H 13.5700 -2.9520 -35.6510

H 13.8320 -2.8290 -33.9120

C 14.7520 -4.5450 -34.8250

H 14.5360 -5.2470 -35.6350

H 14.8050 -5.1240 -33.9000

C 16.1020 -3.8790 -35.0810

H 16.0420 -3.2750 -35.9890

H 16.3360 -3.2070 -34.2520

C 17.2200 -4.9030 -35.2340

H 17.3090 -5.5090 -34.3340

H 18.1720 -4.4080 -35.4100

H 17.0180 -5.5670 -36.0720

C 2.3470 -0.8290 -37.7670

H 2.3970 0.2680 -37.7140

H 1.4240 -1.1150 -38.2770

C 3.5050 -1.3940 -38.5840

H 3.5220 -0.8570 -39.5350

H 3.2840 -2.4400 -38.7940

C 4.8620 -1.2880 -37.8990

H 4.8330 -1.8520 -36.9650

H 5.0740 -0.2450 -37.6560

C 5.9720 -1.8420 -38.7870

H 5.7110 -2.8570 -39.0900

H 6.0470 -1.2330 -39.6910

C 7.3240 -1.8680 -38.0810

H 7.5770 -0.8620 -37.7390

H 7.2580 -2.5120 -37.2010

C 8.4310 -2.3790 -38.9960

H 8.1760 -3.3830 -39.3410

H 8.4970 -1.7330 -39.8750

C 9.7870 -2.4190 -38.2990

H 9.7300 -3.0880 -37.4370

H 10.0330 -1.4210 -37.9290

C 10.8950 -2.8930 -39.2340

H 10.6500 -3.8930 -39.6000

H 10.9440 -2.2270 -40.0990

C 12.2560 -2.9280 -38.5470

H 12.4970 -1.9300 -38.1710

H 12.2080 -3.6030 -37.6890

C 13.3630 -3.3900 -39.4900

H 13.4090 -2.7170 -40.3500

H 13.1230 -4.3880 -39.8630

C 14.7250 -3.4250 -38.8030

H 14.9700 -2.4260 -38.4360

H 14.6720 -4.0930 -37.9410

C 15.8260 -3.9020 -39.7410

H 15.6180 -4.9100 -40.0910

H 16.7880 -3.9080 -39.2320

H 15.9010 -3.2460 -40.6070

O 2.4230 -2.5750 -33.8650

O 0.3070 -2.5730 -32.3850

O 2.3860 -1.3600 -36.4490

O -3.5570 -0.7100 -35.3450

C -6.7270 -0.3680 -31.5140

C -7.8780 -0.3070 -30.7190

C -5.5060 -0.4410 -30.8370

C -7.7560 -0.3310 -29.3430

H -8.8530 -0.2500 -31.1800

C -5.4990 -0.4510 -29.4540

H -4.5740 -0.4910 -31.3760

H -8.6280 -0.2930 -28.7040

H -4.5700 -0.5020 -28.9030

C -4.8450 -0.1320 -41.7550

C -3.5850 -0.0910 -42.3660

C -5.9620 -0.0350 -42.5900

C -3.5030 0.0370 -43.7360

H -2.6880 -0.1550 -41.7680

C -5.7730 0.0810 -43.9520

H -6.9600 -0.0580 -42.1860

H -2.5510 0.0810 -44.2470

H -6.5990 0.1550 -44.6450

C -9.5770 -0.1770 -37.4500

H -9.6390 -1.0630 -36.8000

H -9.7130 0.7220 -36.8330

C -2.4250 -0.8850 -36.1650

H -2.5900 -1.7460 -36.8360

H -2.2800 0.0000 -36.7980

C -5.6550 0.3520 -47.6080

C -5.9600 0.5820 -53.2540

C -6.0980 0.5810 -54.4550

C -6.4030 0.5640 -55.8270

C -5.4410 0.2300 -56.7990

C -7.7130 0.8620 -56.2050

H -8.3890 1.1480 -55.4170

O -6.6490 0.3620 -46.9130

O -4.4320 0.2460 -47.1300

C -5.1140 -0.1740 -66.3300

C -7.1750 0.3760 -61.0870

C -7.3510 0.4630 -59.8910

C -7.1670 0.5000 -58.4970

C -8.1370 0.8040 -57.5260

C -5.8450 0.2240 -58.1180

H -5.1550 0.0050 -58.9150

O -3.9040 -0.2850 -66.4840

O -5.9780 -0.1970 -67.3110

H -5.5430 -0.3670 -68.2020

O -9.4140 1.0340 -57.9340

C -1.8980 -0.7410 -56.7750

C -1.6850 -0.7890 -55.4090

H -2.4880 -0.4890 -54.7580

C -0.4590 -1.2300 -54.9180

C 0.5580 -1.6250 -55.8050

C 0.3440 -1.5480 -57.1930

C -0.8870 -1.0930 -57.6570

H -1.0810 -1.0270 -58.7160

C -1.0240 -0.7160 -52.6680

H -2.0350 -1.1310 -52.7700

H -1.0720 0.3630 -52.8670

C -0.5420 -0.9560 -51.2400

H -0.5610 -2.0270 -51.0370

H -1.2700 -0.4760 -50.5830

C 0.8640 -0.4150 -50.9570

H 1.0780 0.4200 -51.6260

H 0.9040 -0.0350 -49.9330

C 1.9370 -1.4890 -51.1240

H 1.8840 -1.8910 -52.1370

H 1.7280 -2.3040 -50.4270

C 3.3390 -0.9510 -50.8490

H 3.6250 -0.2650 -51.6500

H 3.3360 -0.3810 -49.9170

C 4.3710 -2.0720 -50.7460

H 4.2770 -2.7290 -51.6150

H 4.1620 -2.6750 -49.8590

C 5.8000 -1.5410 -50.6750

H 5.9970 -0.9310 -51.5600

H 5.9080 -0.8940 -49.8010

C 6.8290 -2.6680 -50.6060

H 6.6420 -3.3730 -51.4210

H 6.7100 -3.2140 -49.6680

C 8.2600 -2.1480 -50.7180

H 8.3640 -1.5860 -51.6490

H 8.4600 -1.4570 -49.8960

C 9.2850 -3.2790 -50.6990

H 9.2210 -3.8130 -49.7490

H 9.0480 -3.9930 -51.4930

C 10.7100 -2.7690 -50.8980

H 10.9520 -2.0550 -50.1070

H 10.7710 -2.2350 -51.8490

C 11.7290 -3.9020 -50.8880

H 11.7030 -4.4310 -49.9380

H 12.7350 -3.5140 -51.0360

H 11.5140 -4.6150 -51.6810

C 2.9030 -1.3830 -55.5260

H 2.8050 -0.3600 -55.1350

H 3.0690 -1.3270 -56.6080

C 4.0620 -2.1150 -54.8580

H 3.9370 -3.1780 -55.0680

H 3.9990 -1.9780 -53.7780

C 5.4200 -1.6480 -55.3720

H 5.4340 -1.7370 -56.4610

H 5.5690 -0.5950 -55.1250

C 6.5620 -2.4830 -54.7950

H 6.3460 -3.5410 -54.9590

H 6.6250 -2.3220 -53.7170

C 7.9060 -2.1520 -55.4400

H 7.8200 -2.2680 -56.5230

H 8.1620 -1.1080 -55.2430

C 9.0250 -3.0580 -54.9310

H 8.7450 -4.1010 -55.0960

H 9.1450 -2.9170 -53.8540

C 10.3550 -2.7830 -55.6290

H 10.2320 -2.9230 -56.7050

H 10.6420 -1.7420 -55.4670

C 11.4690 -3.6980 -55.1270

H 11.1820 -4.7400 -55.2880

H 11.5970 -3.5550 -54.0510

C 12.7980 -3.4280 -55.8290

H 12.6770 -3.5850 -56.9040

H 13.0780 -2.3830 -55.6820

C 13.9190 -4.3240 -55.3100

H 13.6480 -5.3710 -55.4640

H 14.0350 -4.1680 -54.2350

C 15.2480 -4.0400 -56.0050

H 15.1370 -4.2030 -57.0790

H 15.5130 -2.9910 -55.8570

C 16.3710 -4.9230 -55.4750

H 16.5180 -4.7540 -54.4100

H 17.3050 -4.7050 -55.9880

H 16.1340 -5.9730 -55.6250

C 1.2930 -1.5730 -59.3830

H 1.2580 -0.4790 -59.4480

H 0.3870 -1.9830 -59.8450

C 2.4870 -2.1240 -60.1570

H 2.4200 -1.7280 -61.1710

H 2.3830 -3.2070 -60.2120

C 3.8600 -1.7940 -59.5750

H 3.8590 -2.0090 -58.5050

H 4.0830 -0.7350 -59.7120

C 4.9240 -2.6520 -60.2570

H 4.6860 -3.7020 -60.0740

H 4.8740 -2.4910 -61.3360

C 6.3430 -2.3750 -59.7660

H 6.6490 -1.3700 -60.0620

H 6.3650 -2.4140 -58.6730

C 7.3310 -3.4000 -60.3190

H 7.0270 -4.3980 -59.9910

H 7.2900 -3.3870 -61.4110

C 8.7650 -3.1450 -59.8640

H 8.8010 -3.1370 -58.7710

H 9.0870 -2.1600 -60.2110

C 9.7290 -4.2070 -60.3890

H 9.4200 -5.1870 -60.0190

H 9.6750 -4.2320 -61.4800

C 11.1700 -3.9390 -59.9630

H 11.4770 -2.9560 -60.3280

H 11.2250 -3.9160 -58.8710

C 12.1340 -4.9970 -60.4930

H 12.0600 -5.0410 -61.5820

H 11.8460 -5.9770 -60.1030

C 13.5810 -4.7060 -60.1010

H 13.8630 -3.7180 -60.4720

H 13.6620 -4.6850 -59.0110

C 14.5410 -5.7490 -60.6570

H 14.3020 -6.7340 -60.2640

H 15.5680 -5.5110 -60.3860

H 14.4740 -5.7900 -61.7430

O 1.7170 -2.1190 -55.2540

O -0.1500 -1.3250 -53.5970

O 1.3560 -1.9490 -58.0120

O -4.1790 -0.0430 -56.3700

C -5.8500 0.5610 -51.8520

C -4.6680 0.1520 -51.2320

C -6.9510 0.9310 -51.0730

C -4.5930 0.1010 -49.8580

H -3.8190 -0.1190 -51.8430

C -6.8770 0.8590 -49.7000

H -7.8550 1.2670 -51.5590

C -5.7010 0.4400 -49.0870

H -3.6880 -0.2170 -49.3640

H -7.7160 1.1260 -49.0780

C -6.7510 0.2490 -62.4230

C -5.3710 0.0770 -62.6120

C -7.5990 0.2770 -63.5310

C -4.8570 -0.0490 -63.8790

H -4.7240 0.0490 -61.7480

C -7.0790 0.1420 -64.8020

H -8.6620 0.4060 -63.3860

C -5.7070 -0.0190 -64.9850

H -3.7990 -0.1790 -64.0430

H -7.7240 0.1620 -65.6660

C -10.3700 1.2720 -56.9170

H -10.2590 0.5240 -56.1230

H -10.2000 2.2690 -56.4830

C -3.2280 -0.3510 -57.3640

H -3.5850 -1.1910 -57.9770

H -3.1020 0.5160 -58.0300

N -6.5940 -0.3990 -28.7140

N -4.5720 0.1100 -44.5130

H -4.4550 0.1910 -46.1150

C -10.6730 -0.2590 -38.4840

C -10.3860 -0.3750 -39.8350

H -9.3600 -0.3510 -40.1580

C -11.4140 -0.5550 -40.7470

C -12.7530 -0.6420 -40.3140

C -13.0420 -0.4790 -38.9490

C -11.9900 -0.2740 -38.0610

H -12.1960 -0.1580 -37.0100

C -9.9750 -0.4520 -42.6540

H -9.2480 -1.1780 -42.2570

H -9.6320 0.5610 -42.3980

C -10.0540 -0.6180 -44.1700

H -10.5250 -1.5790 -44.3820

H -9.0300 -0.6490 -44.5470

C -10.8160 0.4840 -44.9070

H -10.5090 1.4600 -44.5250

H -10.5200 0.4390 -45.9580

C -12.3360 0.3510 -44.8230

H -12.6710 0.5500 -43.8050

H -12.6160 -0.6780 -45.0610

C -13.0290 1.2910 -45.8100

H -12.7460 2.3240 -45.5920

H -12.6760 1.0630 -46.8190

C -14.5510 1.1670 -45.7800

H -14.9240 1.5270 -44.8180

H -14.8310 0.1140 -45.8680

C -15.2190 1.9600 -46.9030

H -14.8750 2.9960 -46.8670

H -14.9130 1.5440 -47.8660

C -16.7430 1.9340 -46.8030

H -17.0480 2.4160 -45.8710

H -17.0880 0.8980 -46.7710

C -17.4150 2.6500 -47.9730

H -17.0070 3.6600 -48.0610

H -17.1840 2.1200 -48.9000

C -18.9310 2.7340 -47.8000

H -19.3390 1.7280 -47.6800

H -19.1570 3.2970 -46.8900

C -19.6070 3.4140 -48.9870

H -19.4140 2.8300 -49.8910

H -19.1680 4.4030 -49.1340

C -21.1110 3.5570 -48.7870

H -21.5780 2.5810 -48.6640

H -21.5680 4.0500 -49.6430

H -21.3220 4.1510 -47.9000

C -15.0160 -0.7530 -41.1300

H -15.2190 0.1970 -40.6260

H -15.3950 -1.5720 -40.5080

C -15.7060 -0.7520 -42.4900

H -15.5390 -1.7080 -42.9870

H -15.2580 0.0240 -43.1100

C -17.1970 -0.4880 -42.3080

H -17.6360 -1.3020 -41.7270

H -17.3200 0.4310 -41.7300

C -17.9590 -0.3350 -43.6210

H -17.9230 -1.2690 -44.1870

H -17.4820 0.4360 -44.2290

C -19.4100 0.0560 -43.3530

H -19.8880 -0.7240 -42.7580

H -19.4230 0.9750 -42.7620

C -20.2200 0.2840 -44.6250

H -20.2600 -0.6400 -45.2070

H -19.7250 1.0390 -45.2400

C -21.6380 0.7450 -44.2990

H -22.1410 -0.0270 -43.7130

H -21.5870 1.6450 -43.6820

C -22.4590 1.0460 -45.5490

H -22.5120 0.1510 -46.1730

H -21.9570 1.8220 -46.1320

C -23.8720 1.5060 -45.2020

H -24.3820 0.7170 -44.6440

H -23.8160 2.3830 -44.5520

C -24.6870 1.8520 -46.4450

H -24.7330 0.9810 -47.1030

H -24.1830 2.6510 -46.9960

C -26.1050 2.2970 -46.0970

H -26.6120 1.4960 -45.5550

H -26.0590 3.1630 -45.4330

C -26.9080 2.6530 -47.3420

H -26.4310 3.4690 -47.8820

H -27.9160 2.9610 -47.0740

H -26.9750 1.7970 -48.0100

C -14.7030 0.0060 -37.2890

H -14.4090 1.0610 -37.2700

H -14.1940 -0.5110 -36.4670

C -16.2110 -0.1420 -37.0770

H -16.4780 0.4970 -36.2320

H -16.4230 -1.1710 -36.7960

C -17.0530 0.2200 -38.2980

H -16.8500 -0.5010 -39.0890

H -16.7690 1.2070 -38.6650

C -18.5470 0.2100 -37.9840

H -18.8450 -0.7930 -37.6720

H -18.7470 0.8890 -37.1500

C -19.3880 0.6300 -39.1860

H -19.1070 1.6430 -39.4830

H -19.1750 -0.0320 -40.0290

C -20.8850 0.5970 -38.8850

H -21.1970 -0.4330 -38.6950

H -21.0800 1.1740 -37.9770

C -21.7120 1.1780 -40.0290

H -21.5010 0.6290 -40.9490

H -21.4130 2.2170 -40.1890

C -23.2100 1.1320 -39.7390

H -23.5390 0.0930 -39.6760

H -23.4000 1.5980 -38.7680

C -24.0210 1.8630 -40.8060

H -23.7010 2.9070 -40.8400

H -23.8160 1.4230 -41.7850

C -25.5220 1.8120 -40.5300

H -25.7170 2.2030 -39.5280

H -25.8620 0.7740 -40.5500

C -26.3170 2.6290 -41.5450

H -25.9770 3.6660 -41.5080

H -26.1180 2.2500 -42.5510

C -27.8140 2.5830 -41.2700

H -28.1840 1.5620 -41.3180

H -28.3570 3.1800 -42.0000

H -28.0290 2.9760 -40.2780

O -13.6250 -0.9030 -41.3290

O -11.2500 -0.6860 -42.0920

O -14.3430 -0.5610 -38.5390

C -11.7910 1.1730 -57.4030

C -12.1380 1.0090 -58.7300

H -11.3590 0.9800 -59.4730

C -13.4740 0.8510 -59.0720

C -14.4810 0.8130 -58.0880

C -14.1210 1.0280 -56.7450

C -12.7810 1.2240 -56.4360

H -12.4810 1.3800 -55.4140

C -13.0330 1.0320 -61.4020

H -12.2330 0.2770 -61.4570

H -12.5650 2.0110 -61.2210

C -13.7920 1.0570 -62.7260

H -14.3290 0.1140 -62.8390

H -13.0470 1.1240 -63.5220

C -14.7760 2.2230 -62.8540

H -14.3290 3.1230 -62.4250

H -14.9480 2.4180 -63.9160

C -16.1230 1.9500 -62.1870

H -15.9720 1.7400 -61.1280

H -16.5620 1.0570 -62.6400

C -17.0890 3.1210 -62.3650

H -16.7280 3.9850 -61.8010

H -17.1190 3.4070 -63.4200

C -18.5020 2.7700 -61.9070

H -18.4740 2.4650 -60.8590

H -18.8650 1.9180 -62.4880

C -19.4800 3.9330 -62.0610

H -19.1470 4.7710 -61.4440

H -19.4820 4.2700 -63.1000

C -20.8980 3.5340 -61.6570

H -20.8910 3.1810 -60.6240

H -21.2300 2.7050 -62.2860

C -21.8910 4.6870 -61.7820

H -21.5680 5.5150 -61.1470

H -21.8970 5.0480 -62.8130

C -23.3060 4.2670 -61.3870

H -23.6280 3.4410 -62.0260

H -23.3010 3.9050 -60.3570

C -24.3020 5.4170 -61.5110

H -24.3220 5.7640 -62.5460

H -23.9660 6.2520 -60.8920

C -25.7090 5.0080 -61.0920

H -26.0760 4.2010 -61.7220

H -26.3930 5.8500 -61.1810

H -25.7180 4.6640 -60.0600

C -16.8220 0.4350 -57.7020

H -16.9540 1.3600 -57.1340

H -16.6300 -0.3830 -57.0000

C -18.1040 0.1500 -58.4760

H -18.0140 -0.7960 -59.0090

H -18.2600 0.9360 -59.2160

C -19.2750 0.1030 -57.4970

H -19.1380 -0.7400 -56.8150

H -19.2610 1.0150 -56.8950

C -20.6420 -0.0090 -58.1660

H -20.7080 -0.9420 -58.7270

H -20.7690 0.8120 -58.8750

C -21.7550 0.0460 -57.1210

H -21.6470 -0.7980 -56.4350

H -21.6440 0.9610 -56.5350

C -23.1540 0.0220 -57.7310

H -23.2960 -0.9070 -58.2870

H -23.2540 0.8470 -58.4410

C -24.2320 0.1460 -56.6570

H -24.1270 -0.6780 -55.9460

H -24.0820 1.0770 -56.1060

C -25.6420 0.1330 -57.2390

H -25.8050 -0.8040 -57.7760

H -25.7430 0.9460 -57.9620

C -26.7050 0.2860 -56.1550

H -26.5840 -0.5090 -55.4160

H -26.5600 1.2410 -55.6420

C -28.1220 0.2330 -56.7200

H -28.2820 -0.7340 -57.2030

H -28.2370 1.0060 -57.4840

C -29.1800 0.4360 -55.6390

H -29.0640 -0.3340 -54.8720

H -29.0260 1.4070 -55.1620

C -30.5920 0.3730 -56.2080

H -30.7340 1.1460 -56.9600

H -31.3290 0.5190 -55.4210

H -30.7730 -0.5940 -56.6730

C -14.7760 1.4110 -54.4590

H -14.5130 2.4760 -54.4500

H -13.9250 0.8280 -54.0880

C -15.9640 1.1510 -53.5280

H -15.7490 1.6600 -52.5860

H -16.0240 0.0840 -53.3170

C -17.3160 1.6120 -54.0610

H -17.5570 1.0380 -54.9570

H -17.2680 2.6650 -54.3380

C -18.4170 1.4070 -53.0210

H -18.3920 0.3750 -52.6650

H -18.2250 2.0540 -52.1620

C -19.8030 1.7100 -53.5830

H -19.8290 2.7410 -53.9400

H -19.9930 1.0560 -54.4390

C -20.9070 1.5140 -52.5470

H -20.8990 0.4800 -52.1940

H -20.7140 2.1580 -51.6870

C -22.2830 1.8480 -53.1170

H -22.4830 1.2060 -53.9800

H -22.2820 2.8830 -53.4670

C -23.3980 1.6720 -52.0910

H -23.4290 0.6310 -51.7610

H -23.1840 2.2880 -51.2150

C -24.7580 2.0660 -52.6630

H -24.7250 3.1130 -52.9730

H -24.9630 1.4630 -53.5510

C -25.8900 1.8770 -51.6570

H -25.6830 2.4690 -50.7630

H -25.9340 0.8280 -51.3560

C -27.2400 2.2930 -52.2360

H -27.2010 3.3490 -52.5170

H -27.4350 1.7180 -53.1440

C -28.3770 2.0750 -51.2460

H -28.4440 1.0240 -50.9700

H -29.3270 2.3750 -51.6820

H -28.2130 2.6570 -50.3430

O -15.7320 0.5760 -58.5910

O -13.9240 0.7210 -60.3550

O -15.1020 1.0290 -55.7840

C -4.2780 -3.2710 -55.6700

C -5.2180 -3.2350 -56.6280

C -4.9790 -3.3680 -58.0460

C -6.0390 -3.1620 -58.9580

C -3.7240 -3.6970 -58.5570

H -2.9370 -3.9010 -57.8540

C -5.0620 -3.2110 -63.2450

C -4.2070 -3.4570 -62.2390

C -4.5060 -3.4570 -60.8270

C -3.4730 -3.7770 -59.9150

C -5.7770 -3.1760 -60.3160

H -6.5690 -2.9630 -61.0130

O -2.2680 -4.1420 -60.4290

C -9.6230 -2.6050 -58.3980

C -9.5260 -2.3170 -57.0490

H -8.5490 -2.2890 -56.6020

C -10.6670 -2.0590 -56.3030

C -11.9380 -2.1090 -56.9080

C -12.0260 -2.3900 -58.2830

C -10.8660 -2.6500 -59.0040

H -10.9190 -2.8690 -60.0590

C -9.4460 -1.7380 -54.2710

H -8.6370 -1.2940 -54.8660

H -9.1920 -2.7840 -54.0510

C -9.5540 -0.9340 -52.9780

H -9.8530 0.0880 -53.2260

H -8.5460 -0.8980 -52.5580

C -10.5150 -1.5050 -51.9370

H -10.4440 -2.5900 -51.9290

H -10.2090 -1.1440 -50.9520

C -11.9680 -1.1110 -52.1820

H -12.2720 -1.4520 -53.1720

H -12.0530 -0.0200 -52.1590

C -12.8900 -1.7090 -51.1200

H -12.8810 -2.7950 -51.2160

H -12.5090 -1.4540 -50.1270

C -14.3130 -1.1800 -51.2650

H -14.6930 -1.4640 -52.2480

H -14.2910 -0.0880 -51.2240

C -15.2740 -1.6900 -50.1930

H -15.3120 -2.7810 -50.2250

H -14.9090 -1.4000 -49.2050

C -16.6740 -1.1190 -50.4090

H -17.0290 -1.4170 -51.3980

H -16.6200 -0.0270 -50.3990

C -17.6900 -1.5740 -49.3650

H -17.7310 -2.6660 -49.3480

H -17.3740 -1.2380 -48.3750

C -19.0790 -1.0180 -49.6740

H -19.0310 0.0730 -49.7120

H -19.3900 -1.3640 -50.6630

C -20.1290 -1.4380 -48.6490

H -19.8450 -1.0590 -47.6650

H -20.1590 -2.5290 -48.5880

C -21.5110 -0.9120 -49.0170

H -21.5060 0.1740 -49.0650

H -22.2510 -1.2220 -48.2820

H -21.8170 -1.2920 -49.9890

C -14.2430 -2.4150 -56.3670

H -14.1080 -3.4810 -56.5760

H -14.6910 -1.9350 -57.2420

C -15.1580 -2.2310 -55.1650

H -15.3440 -1.1660 -55.0420

H -14.6510 -2.5950 -54.2720

C -16.4850 -2.9600 -55.3720

H -16.8550 -2.7440 -56.3780

H -16.3340 -4.0400 -55.3050

C -17.5390 -2.5230 -54.3580

H -17.6560 -1.4390 -54.4220

H -17.1950 -2.7630 -53.3500

C -18.8970 -3.1770 -54.6030

H -19.1620 -3.0710 -55.6580

H -18.8390 -4.2460 -54.3870

C -19.9890 -2.5340 -53.7510

H -20.0170 -1.4620 -53.9620

H -19.7430 -2.6560 -52.6930

C -21.3690 -3.1280 -54.0210

H -21.5690 -3.1020 -55.0960

H -21.3850 -4.1750 -53.7130

C -22.4690 -2.3620 -53.2900

H -22.4520 -1.3180 -53.6110

H -22.2690 -2.3790 -52.2150

C -23.8540 -2.9460 -53.5540

H -24.0250 -2.9900 -54.6330

H -23.8940 -3.9710 -53.1770

C -24.9600 -2.1210 -52.9010

H -24.9230 -1.0990 -53.2860

H -24.7860 -2.0740 -51.8230

C -26.3460 -2.7060 -53.1600

H -26.5230 -2.7540 -54.2380

H -26.3850 -3.7280 -52.7770

C -27.4430 -1.8770 -52.5030

H -27.3010 -1.8450 -51.4250

H -28.4220 -2.3040 -52.7060

H -27.4270 -0.8560 -52.8800

C -13.4010 -2.7010 -60.2290

H -13.0870 -3.7410 -60.4050

H -12.7760 -2.0370 -60.8410

C -14.8640 -2.5130 -60.6220

H -14.9510 -2.6870 -61.6970

H -15.1420 -1.4780 -60.4130

C -15.7950 -3.4560 -59.8650

H -15.7360 -3.2160 -58.8030

H -15.4580 -4.4870 -59.9890

C -17.2460 -3.3350 -60.3310

H -17.4730 -2.2830 -60.5170

H -17.3770 -3.8670 -61.2760

C -18.2250 -3.8800 -59.2930

H -17.9890 -4.9240 -59.0750

H -18.1010 -3.3160 -58.3640

C -19.6780 -3.7660 -59.7490

H -19.8510 -2.7610 -60.1420

H -19.8630 -4.4710 -60.5630

C -20.6630 -4.0290 -58.6110

H -20.4890 -3.2990 -57.8160

H -20.4820 -5.0210 -58.1910

C -22.1150 -3.9270 -59.0720

H -22.2570 -2.9810 -59.6020

H -22.3290 -4.7320 -59.7780

C -23.0990 -3.9960 -57.9060

H -22.9390 -4.9210 -57.3480

H -22.9060 -3.1630 -57.2260

C -24.5510 -3.9310 -58.3760

H -24.7690 -4.8030 -58.9980

H -24.6900 -3.0420 -58.9970

C -25.5340 -3.8830 -57.2090

H -25.3780 -4.7550 -56.5680

H -25.3330 -2.9920 -56.6110

C -26.9820 -3.8530 -57.6810

H -27.1570 -2.9890 -58.3190

H -27.6610 -3.7970 -56.8330

H -27.2180 -4.7490 -58.2510

O -12.9840 -1.8300 -56.0810

O -10.6650 -1.6990 -54.9910

O -13.2650 -2.3800 -58.8610

O -7.2650 -2.9620 -58.4040

C -4.5080 -3.1610 -54.2490

C -3.4110 -3.2830 -53.3860

C -5.7610 -2.9610 -53.6600

C -3.6100 -3.1930 -52.0260

H -2.4220 -3.4500 -53.7870

C -5.8530 -2.8900 -52.2850

H -6.6500 -2.8650 -54.2600

H -2.7910 -3.2770 -51.3240

H -6.7980 -2.7400 -51.7790

C -4.7350 -3.2450 -64.6550

C -5.7650 -3.1520 -65.5970

C -3.4360 -3.3720 -65.1560

C -5.4610 -3.2080 -66.9440

H -6.7900 -3.0460 -65.2760

C -3.2430 -3.4060 -66.5240

H -2.5870 -3.4340 -64.4940

H -6.2370 -3.1460 -67.6960

H -2.2510 -3.5010 -66.9460

C -1.3110 -4.6540 -59.5230

H -1.0590 -3.8930 -58.7700

H -1.7330 -5.5270 -59.0010

C -8.3900 -2.8170 -59.2400

H -8.2590 -1.9460 -59.9020

H -8.5120 -3.7120 -59.8680

C -6.4790 -3.5500 -29.4000

C -7.7210 -3.5870 -34.9160

C -7.7690 -3.5860 -36.1250

C -7.6070 -3.5620 -37.5200

C -8.6750 -3.5290 -38.4340

C -6.2850 -3.5760 -37.9770

H -5.5180 -3.6410 -37.2220

O -5.3320 -3.3020 -29.0510

O -7.4510 -3.8170 -28.5660

C -6.2420 -2.9330 -48.4660

C -6.7720 -3.2290 -42.8280

C -6.8300 -3.3090 -41.6230

C -7.0460 -3.4120 -40.2360

C -5.9780 -3.5150 -39.3230

C -8.3660 -3.4430 -39.7820

H -9.1390 -3.3940 -40.5320

O -7.1800 -2.9780 -49.2350

O -4.9860 -2.8630 -48.8630

O -4.7190 -3.5560 -39.8370

C -12.3530 -3.6790 -38.2310

C -12.5720 -3.4200 -36.8860

H -11.7170 -3.3020 -36.2400

C -13.8670 -3.2910 -36.4090

C -14.9770 -3.5020 -37.2640

C -14.7390 -3.7450 -38.6190

C -13.4300 -3.8250 -39.0810

H -13.2590 -3.9620 -40.1380

C -13.2470 -2.8470 -34.1200

H -12.3210 -2.3880 -34.4870

H -13.0080 -3.8580 -33.7650

C -13.7870 -2.0010 -32.9600

H -13.6580 -0.9430 -33.1960

H -13.1670 -2.2280 -32.0910

C -15.2640 -2.2480 -32.6290

H -15.5110 -3.2940 -32.8120

H -15.4260 -2.0460 -31.5680

C -16.1860 -1.3540 -33.4560

H -16.0610 -1.6070 -34.5070

H -15.8840 -0.3120 -33.3170

C -17.6600 -1.4890 -33.0800

H -18.0010 -2.5020 -33.3010

H -17.7870 -1.3220 -32.0080

C -18.5080 -0.4820 -33.8570

H -18.3040 -0.6010 -34.9230

H -18.2040 0.5300 -33.5800

C -20.0090 -0.6350 -33.6210

H -20.3110 -1.6590 -33.8540

H -20.2360 -0.4560 -32.5680

C -20.8060 0.3370 -34.4890

H -20.5700 0.1500 -35.5400

H -20.4980 1.3590 -34.2600

C -22.3150 0.2120 -34.2900

H -22.6220 -0.8180 -34.4860

H -22.5640 0.4380 -33.2510

C -23.0870 1.1530 -35.2120

H -22.7750 2.1820 -35.0210

H -22.8390 0.9190 -36.2510

C -24.5980 1.0430 -35.0240

H -24.8520 1.2900 -33.9900

H -24.9080 0.0120 -35.2040

C -25.3570 1.9710 -35.9640

H -25.0890 3.0090 -35.7790

H -26.4300 1.8630 -35.8270

H -25.1220 1.7370 -37.0010

C -17.3640 -3.7330 -37.3540

H -17.3230 -4.7770 -37.6920

H -17.4530 -3.0870 -38.2330

C -18.5880 -3.5280 -36.4680

H -18.5750 -2.5070 -36.0850

H -18.5490 -4.2030 -35.6130

C -19.8540 -3.7630 -37.2930

H -19.7100 -3.3230 -38.2830

H -20.0060 -4.8360 -37.4350

C -21.1100 -3.1460 -36.6820

H -20.9270 -2.0870 -36.4890

H -21.3370 -3.6220 -35.7260

C -22.3030 -3.2800 -37.6280

H -22.0010 -2.9500 -38.6260

H -22.5930 -4.3300 -37.7050

C -23.5040 -2.4480 -37.1840

H -23.1990 -1.4030 -37.0960

H -23.8380 -2.7810 -36.1990

C -24.6610 -2.5460 -38.1760

H -24.2980 -2.2940 -39.1760

H -25.0270 -3.5750 -38.2090

C -25.8090 -1.6070 -37.8130

H -25.4390 -0.5800 -37.7980

H -26.1670 -1.8440 -36.8080

C -26.9720 -1.7040 -38.7970

H -26.6070 -1.5030 -39.8070

H -27.3720 -2.7200 -38.7880

C -28.0860 -0.7160 -38.4600

H -27.6900 0.3010 -38.5010

H -28.4310 -0.8960 -37.4390

C -29.2690 -0.8340 -39.4180

H -28.9230 -0.6660 -40.4400

H -29.6740 -1.8470 -39.3700

C -30.3690 0.1680 -39.0880

H -30.7430 0.0050 -38.0790

H -31.2010 0.0670 -39.7820

H -29.9900 1.1850 -39.1530

C -15.8770 -4.9760 -40.3290

H -16.2550 -5.8090 -39.7140

H -14.8980 -5.2660 -40.7260

C -16.8420 -4.6940 -41.4770

H -16.9250 -5.5960 -42.0860

H -16.4280 -3.8970 -42.0960

C -18.2190 -4.2750 -40.9690

H -18.1090 -3.3490 -40.4030

H -18.6010 -5.0370 -40.2850

C -19.2240 -4.0580 -42.0960

H -18.8090 -3.3600 -42.8260

H -19.4060 -5.0040 -42.6110

C -20.5460 -3.5060 -41.5670

H -20.9410 -4.1810 -40.8030

H -20.3640 -2.5400 -41.0870

C -21.5850 -3.3320 -42.6720

H -21.1650 -2.7170 -43.4710

H -21.8220 -4.3090 -43.1000

C -22.8660 -2.6820 -42.1570

H -22.6330 -1.6890 -41.7600

H -23.2690 -3.2780 -41.3340

C -23.9230 -2.5540 -43.2520

H -23.5020 -2.0090 -44.1000

H -24.1950 -3.5510 -43.6060

C -25.1750 -1.8330 -42.7610

H -25.5800 -2.3640 -41.8960

H -24.9060 -0.8260 -42.4330

C -26.2480 -1.7430 -43.8430

H -26.5430 -2.7510 -44.1440

H -25.8350 -1.2460 -44.7240

C -27.4800 -0.9780 -43.3650

H -27.8830 -1.4700 -42.4760

H -27.1850 0.0340 -43.0810

C -28.5620 -0.9080 -44.4350

H -28.1840 -0.4200 -45.3310

H -29.4210 -0.3460 -44.0750

H -28.8950 -1.9070 -44.7070

O -16.1880 -3.4040 -36.6410

O -14.2150 -2.9340 -35.1450

O -15.7780 -3.8120 -39.5260

O -9.9380 -3.6000 -37.9240

C -7.4870 -3.5880 -33.5300

C -8.5060 -3.6050 -32.5770

C -6.1480 -3.5540 -33.1180

C -8.1930 -3.6030 -31.2340

H -9.5380 -3.6280 -32.8960

C -5.8420 -3.5370 -31.7800

H -5.3690 -3.5350 -33.8660

C -6.8600 -3.5740 -30.8270

H -8.9750 -3.6120 -30.4910

H -4.8200 -3.5000 -31.4380

C -6.6490 -3.1480 -44.2280

C -7.7800 -3.2150 -45.0490

C -5.3870 -2.9970 -44.8060

C -7.6450 -3.1260 -46.4170

H -8.7520 -3.3430 -44.5980

C -5.2580 -2.9120 -46.1730

H -4.5190 -2.9590 -44.1680

C -6.3840 -2.9790 -46.9890

H -8.5060 -3.1700 -47.0660

H -4.2870 -2.7950 -46.6270

C -3.6560 -3.7370 -38.9260

H -3.7120 -2.9710 -38.1390

H -3.7440 -4.7190 -38.4380

C -10.9870 -3.7220 -38.8610

H -10.9440 -2.8950 -39.5810

H -10.8710 -4.6680 -39.4150

N -4.8030 -2.9970 -51.4850

N -4.2270 -3.3340 -67.4070

H -4.9310 -2.8810 -49.8770

C -0.0380 -5.0580 -60.2160

C 0.0560 -5.2400 -61.5850

H -0.8170 -5.0770 -62.1930

C 1.2670 -5.6330 -62.1460

C 2.4040 -5.8410 -61.3390

C 2.3110 -5.5900 -59.9580

C 1.0760 -5.2450 -59.4170

H 0.9780 -5.0930 -58.3560

C 0.3390 -5.8620 -64.3230

H -0.2000 -4.9050 -64.2740

H -0.3400 -6.6580 -63.9900

C 0.7520 -6.1140 -65.7710

H 1.3180 -5.2560 -66.1370

H -0.1720 -6.1800 -66.3500

C 1.5860 -7.3830 -65.9670

H 1.2480 -8.1550 -65.2750

H 1.4280 -7.7570 -66.9820

C 3.0780 -7.1270 -65.7630

H 3.2350 -6.6860 -64.7790

H 3.4110 -6.4070 -66.5160

C 3.9140 -8.4000 -65.8940

H 3.7900 -9.0090 -64.9960

H 3.5620 -8.9900 -66.7440

C 5.3930 -8.0720 -66.0900

H 5.6970 -7.3470 -65.3330

H 5.5280 -7.6050 -67.0680

C 6.2970 -9.2990 -65.9890

H 6.2450 -9.6990 -64.9740

H 5.9400 -10.0750 -66.6700

C 7.7470 -8.9570 -66.3240

H 8.0560 -8.0950 -65.7280

H 7.8160 -8.6690 -67.3760

C 8.7020 -10.1160 -66.0510

H 8.6650 -10.3660 -64.9880

H 8.3750 -10.9960 -66.6100

C 10.1390 -9.7750 -66.4380

H 10.1890 -9.5830 -67.5120

H 10.4430 -8.8580 -65.9270

C 11.1170 -10.8910 -66.0780

H 10.8150 -11.8130 -66.5800

H 11.0750 -11.0690 -65.0010

C 12.5450 -10.5410 -66.4770

H 12.6200 -10.4040 -67.5530

H 13.2310 -11.3320 -66.1820

H 12.8600 -9.6170 -65.9950

C 4.6610 -6.6520 -61.3170

H 4.3970 -7.3020 -60.4790

H 5.1640 -5.7590 -60.9270

C 5.6180 -7.3880 -62.2480

H 5.7920 -6.7800 -63.1350

H 5.1720 -8.3320 -62.5650

C 6.9320 -7.6370 -61.5130

H 7.3730 -6.6700 -61.2530

H 6.7240 -8.1630 -60.5780

C 7.9480 -8.4330 -62.3260

H 8.1000 -7.9470 -63.2910

H 7.5640 -9.4380 -62.5150

C 9.2830 -8.5200 -61.5900

H 9.6410 -7.5060 -61.3930

H 9.1320 -9.0080 -60.6240

C 10.3490 -9.2740 -62.3770

H 10.4410 -8.8360 -63.3730

H 10.0440 -10.3160 -62.5000

C 11.7050 -9.2180 -61.6780

H 12.0050 -8.1720 -61.5690

H 11.6120 -9.6430 -60.6750

C 12.7900 -9.9680 -62.4440

H 12.8750 -9.5540 -63.4510

H 12.5060 -11.0190 -62.5430

C 14.1430 -9.8750 -61.7440

H 14.4290 -8.8240 -61.6580

H 14.0520 -10.2780 -60.7320

C 15.2390 -10.6300 -62.4890

H 15.3300 -10.2310 -63.5020

H 14.9600 -11.6830 -62.5740

C 16.5880 -10.5220 -61.7820

H 16.8670 -9.4690 -61.6980

H 16.4970 -10.9200 -60.7690

C 17.6830 -11.2770 -62.5250

H 17.4330 -12.3330 -62.6010

H 18.6340 -11.1860 -62.0050

H 17.8020 -10.8820 -63.5320

C 3.3740 -5.3250 -57.8190

H 2.8480 -6.1320 -57.2930

H 2.8300 -4.3830 -57.6640

C 4.7930 -5.1680 -57.2760

H 4.7210 -5.0770 -56.1900

H 5.2160 -4.2400 -57.6640

C 5.7240 -6.3210 -57.6390

H 5.7940 -6.3870 -58.7230

H 5.3090 -7.2660 -57.2860

C 7.1190 -6.0990 -57.0580

H 7.4620 -5.0980 -57.3330

H 7.0760 -6.1430 -55.9670

C 8.1310 -7.1200 -57.5710

H 7.8340 -8.1230 -57.2550

H 8.1270 -7.1070 -58.6630

C 9.5430 -6.8180 -57.0730

H 9.8090 -5.7970 -57.3580

H 9.5640 -6.8730 -55.9820

C 10.5790 -7.7800 -57.6490

H 10.5320 -7.7460 -58.7410

H 10.3400 -8.8010 -57.3430

C 11.9950 -7.4300 -57.1970

H 12.2180 -6.4010 -57.4870

H 12.0500 -7.4860 -56.1070

C 13.0420 -8.3590 -57.8050

H 12.8340 -9.3890 -57.5040

H 12.9710 -8.3170 -58.8950

C 14.4590 -7.9820 -57.3780

H 14.5390 -8.0540 -56.2910

H 14.6540 -6.9450 -57.6570

C 15.5150 -8.8780 -58.0210

H 15.3320 -9.9160 -57.7340

H 15.4270 -8.8160 -59.1080

C 16.9260 -8.4790 -57.6100

H 17.1300 -7.4500 -57.9010

H 17.6610 -9.1220 -58.0890

H 17.0500 -8.5600 -56.5320

O 3.5010 -6.2630 -62.0280

O 1.4670 -5.8380 -63.4770

O 3.4460 -5.6330 -59.1990

C -2.3090 -3.6250 -39.5980

C -2.1930 -3.3080 -40.9390

C -0.9280 -3.1720 -41.5110

C 0.2180 -3.3390 -40.7210

C 0.0930 -3.6920 -39.3670

C -1.1770 -3.8340 -38.8190

H -1.2940 -4.1020 -37.7810

C -1.7650 -3.0310 -43.7350

H -2.5640 -2.3210 -43.4880

H -2.1710 -4.0510 -43.6780

C -1.2750 -2.7520 -45.1520

H -0.9810 -1.7040 -45.2300

H -2.1290 -2.9130 -45.8110

C -0.1030 -3.6380 -45.5890

H -0.1560 -4.5960 -45.0680

H -0.1920 -3.8410 -46.6580

C 1.2530 -2.9830 -45.3280

H 1.3440 -2.7520 -44.2670

H 1.2970 -2.0420 -45.8840

C 2.4100 -3.8760 -45.7720

H 2.5080 -4.7140 -45.0780

H 2.1880 -4.2910 -46.7590

C 3.7360 -3.1210 -45.8440

H 3.9230 -2.6220 -44.8900

H 3.6700 -2.3480 -46.6130

C 4.9000 -4.0570 -46.1610

H 4.9760 -4.8090 -45.3720

H 4.6960 -4.5820 -47.0970

C 6.2340 -3.3230 -46.2780

H 6.4080 -2.7400 -45.3700

H 6.1930 -2.6230 -47.1160

C 7.3920 -4.2980 -46.4790

H 7.4080 -5.0060 -45.6470

H 7.2260 -4.8730 -47.3940

C 8.7440 -3.5960 -46.5630

H 8.7490 -2.9230 -47.4240

H 8.8910 -2.9870 -45.6680

C 9.8960 -4.5900 -46.6900

H 9.7610 -5.1880 -47.5940

H 9.8730 -5.2730 -45.8380

C 11.2500 -3.8920 -46.7420

H 11.3100 -3.2360 -47.6070

H 12.0550 -4.6210 -46.8070

H 11.4030 -3.2910 -45.8470

C 2.2440 -4.2760 -41.5200

H 1.8360 -4.8410 -42.3700

H 2.2390 -4.9310 -40.6430

C 3.6550 -3.7910 -41.8210

H 3.9800 -3.1610 -40.9900

H 3.6220 -3.1630 -42.7120

C 4.6500 -4.9290 -42.0200

H 4.6290 -5.5890 -41.1500

H 4.3590 -5.5250 -42.8880

C 6.0610 -4.3790 -42.2220

H 6.3210 -3.7440 -41.3720

H 6.0750 -3.7500 -43.1140

C 7.1170 -5.4720 -42.3630

H 7.0910 -6.1190 -41.4830

H 6.8880 -6.0930 -43.2320

C 8.5150 -4.8750 -42.5170

H 8.7300 -4.2370 -41.6560

H 8.5390 -4.2410 -43.4050

C 9.6000 -5.9420 -42.6320

H 9.5740 -6.5840 -41.7490

H 9.3970 -6.5740 -43.5010

C 10.9900 -5.3240 -42.7700

H 11.1920 -4.6890 -41.9040

H 11.0130 -4.6850 -43.6560

C 12.0850 -6.3810 -42.8870

H 12.0760 -7.0120 -41.9940

H 11.8750 -7.0250 -43.7440

C 13.4680 -5.7560 -43.0540

H 13.6690 -5.0870 -42.2140

H 13.4820 -5.1530 -43.9650

C 14.5700 -6.8100 -43.1320

H 14.5710 -7.4010 -42.2130

H 14.3590 -7.4900 -43.9610

C 15.9440 -6.1810 -43.3320

H 15.9710 -5.6110 -44.2580

H 16.7140 -6.9480 -43.3790

H 16.1800 -5.5080 -42.5110

C 1.2350 -4.4900 -37.4210

H 0.7960 -5.4880 -37.5310

H 0.6290 -3.9090 -36.7100

C 2.6590 -4.5900 -36.8780

H 2.6070 -5.1410 -35.9360

H 3.0140 -3.5850 -36.6550

C 3.6420 -5.2670 -37.8320

H 3.6940 -4.6910 -38.7550

H 3.2950 -6.2670 -38.0920

C 5.0340 -5.3500 -37.2090

H 5.3470 -4.3500 -36.9000

H 4.9930 -5.9750 -36.3130

C 6.0790 -5.9150 -38.1680

H 5.7690 -6.9070 -38.5050

H 6.1400 -5.2760 -39.0520

C 7.4560 -5.9960 -37.5110

H 7.7350 -5.0030 -37.1510

H 7.4040 -6.6580 -36.6420

C 8.5400 -6.4980 -38.4620

H 8.5820 -5.8450 -39.3370

H 8.2820 -7.5010 -38.8110

C 9.9120 -6.5280 -37.7920

H 10.1580 -5.5250 -37.4360

H 9.8750 -7.1870 -36.9220

C 11.0120 -7.0030 -38.7380

H 10.7710 -8.0060 -39.1000

H 11.0530 -6.3410 -39.6060

C 12.3800 -7.0260 -38.0600

H 12.3420 -7.6860 -37.1900

H 12.6210 -6.0220 -37.7020

C 13.4850 -7.4980 -39.0020

H 13.2430 -8.4970 -39.3710

H 13.5320 -6.8300 -39.8650

C 14.8450 -7.5280 -38.3150

H 15.1160 -6.5350 -37.9620

H 15.6160 -7.8670 -39.0030

H 14.8270 -8.2010 -37.4610

O 1.4470 -3.1180 -41.2920

O -0.6970 -2.8900 -42.8180

O 1.2570 -3.8510 -38.6840

H -7.1480 -3.7930 -27.6040

H -3.9210 -0.3450 -39.8710

H -6.9720 -0.1360 -39.9890

H -4.8370 -0.5420 -33.4640

H -7.8760 -0.2570 -33.3110

H -3.2380 -3.4040 -55.9360

H -6.2490 -3.0710 -56.3580

H -3.1780 -3.6950 -62.4650

H -6.1000 -2.9890 -63.0360

H -3.0920 -3.1770 -41.5170

C -5.4970 5.9650 -32.6500

C -4.4800 5.7970 -33.5110

C -4.5190 5.9100 -34.9500

C -3.3010 5.8800 -35.6670

C -5.7030 6.0670 -35.6690

H -6.6220 6.0930 -35.1110

C -3.4910 6.0480 -40.0370

C -4.5360 6.1920 -39.2060

C -4.5060 6.1080 -37.7660

C -5.7230 6.1670 -37.0500

C -3.3180 5.9810 -37.0440

H -2.3910 5.9740 -37.5920

O -6.8580 6.3090 -37.7860

C 0.1680 5.6450 -34.4660

C -0.1430 4.9200 -33.3300

H -1.1730 4.6900 -33.1220

C 0.8680 4.4940 -32.4770

C 2.2130 4.7710 -32.7770

C 2.5250 5.4780 -33.9520

C 1.4890 5.9440 -34.7580

H 1.7050 6.5270 -35.6380

C -0.6270 3.6990 -30.7890

H -1.3370 3.2240 -31.4830

H -0.9760 4.7200 -30.5770

C -0.5780 2.8870 -29.4940

H -0.3610 1.8450 -29.7340

H -1.5740 2.9320 -29.0500

C 0.4680 3.3980 -28.4970

H 0.5210 4.4860 -28.5560

H 0.1520 3.1380 -27.4840

C 1.8500 2.8020 -28.7610

H 2.1150 2.9660 -29.8050

H 1.8110 1.7230 -28.5880

C 2.9340 3.4120 -27.8760

H 2.9890 4.4870 -28.0690

H 2.6770 3.2820 -26.8220

C 4.2930 2.7760 -28.1600

H 4.4560 2.7650 -29.2400

H 4.2810 1.7380 -27.8210

C 5.4480 3.5150 -27.4900

H 5.4550 4.5540 -27.8300

H 5.3010 3.5260 -26.4080

C 6.7940 2.8700 -27.8170

H 6.8920 2.7820 -28.9010

H 6.8200 1.8610 -27.3990

C 7.9750 3.6700 -27.2730

H 7.9440 4.6830 -27.6840

H 7.8890 3.7550 -26.1870

C 9.3120 3.0240 -27.6280

H 9.3550 2.0220 -27.1940

H 9.3810 2.9200 -28.7130

C 10.5030 3.8370 -27.1270

H 10.4310 3.9530 -26.0430

H 10.4670 4.8370 -27.5670

C 11.8310 3.1770 -27.4790

H 11.8970 2.1890 -27.0290

H 12.6650 3.7740 -27.1150

H 11.9310 3.0700 -28.5570

C 4.2040 5.0540 -31.4360

H 4.0870 5.1070 -30.3470

H 4.1430 6.0650 -31.8420

C 5.5430 4.4150 -31.7890

H 5.6450 4.4330 -32.8750

H 5.5160 3.3710 -31.4680

C 6.7490 5.1120 -31.1670

H 6.7890 6.1500 -31.5030

H 6.6570 5.1180 -30.0790

C 8.0310 4.3880 -31.5730

H 8.0620 4.3140 -32.6640

H 8.0050 3.3680 -31.1800

C 9.3070 5.0740 -31.0960

H 9.3570 6.0840 -31.5070

H 9.2950 5.1600 -30.0080

C 10.5380 4.2810 -31.5330

H 10.5160 4.1600 -32.6200

H 10.4920 3.2820 -31.0930

C 11.8540 4.9420 -31.1350

H 11.9180 5.9320 -31.5920

H 11.8800 5.0790 -30.0520

C 13.0530 4.1020 -31.5700

H 13.0260 3.9720 -32.6550

H 12.9800 3.1080 -31.1200

C 14.3820 4.7390 -31.1730

H 14.4500 5.7350 -31.6150

H 14.4140 4.8580 -30.0870

C 15.5780 3.9040 -31.6240

H 15.5470 3.7860 -32.7100

H 15.5110 2.9070 -31.1820

C 16.9060 4.5430 -31.2250

H 16.9730 5.5420 -31.6630

H 16.9360 4.6600 -30.1390

C 18.0990 3.7100 -31.6770

H 18.0570 2.7140 -31.2410

H 19.0320 4.1790 -31.3700

H 18.1090 3.6100 -32.7600

C 4.2120 6.0580 -35.5520

H 4.3180 7.1490 -35.5840

H 3.4470 5.7410 -36.2670

C 5.5140 5.3740 -35.9490

H 5.5880 5.4440 -37.0340

H 5.4320 4.3190 -35.6880

C 6.7650 5.9770 -35.3220

H 6.7010 5.9210 -34.2340

H 6.8290 7.0320 -35.5960

C 8.0020 5.2260 -35.8090

H 7.9330 4.1820 -35.4930

H 8.0130 5.2370 -36.9020

C 9.3150 5.8130 -35.3010

H 9.4050 6.8490 -35.6360

H 9.3180 5.8180 -34.2090

C 10.5080 5.0070 -35.8090

H 10.4250 3.9790 -35.4490

H 10.4790 4.9770 -36.9010

C 11.8460 5.5870 -35.3620

H 11.8710 5.6430 -34.2710

H 11.9470 6.6050 -35.7470

C 13.0180 4.7410 -35.8510

H 12.9220 3.7290 -35.4490

H 12.9790 4.6690 -36.9410

C 14.3660 5.3210 -35.4350

H 14.4770 6.3210 -35.8620

H 14.3950 5.4240 -34.3470

C 15.5290 4.4430 -35.8880

H 15.4910 4.3280 -36.9740

H 15.4220 3.4490 -35.4460

C 16.8840 5.0240 -35.4930

H 17.0050 6.0060 -35.9580

H 16.9160 5.1680 -34.4110

C 18.0320 4.1170 -35.9180

H 17.9310 3.1340 -35.4610

H 18.9890 4.5380 -35.6150

H 18.0390 3.9930 -36.9980

O 3.1410 4.2380 -31.9220

O 0.6650 3.7530 -31.3570

O 3.8420 5.6560 -34.2390

O -2.1690 5.7260 -34.9190

C -5.3980 5.8740 -31.2080

C -6.5140 6.1740 -30.4180

C -4.2360 5.4970 -30.5260

C -6.4150 6.0990 -29.0420

H -7.4430 6.4700 -30.8810

C -4.2450 5.4550 -29.1450

H -3.3350 5.2500 -31.0610

H -7.2570 6.3340 -28.4050

H -3.3610 5.1740 -28.5880

C -3.5260 6.1490 -41.4780

C -2.3180 6.0950 -42.1830

C -4.6890 6.3010 -42.2410

C -2.3260 6.1660 -43.5570

H -1.3850 6.0040 -41.6530

C -4.5910 6.3700 -43.6170

H -5.6570 6.3530 -41.7700

H -1.4110 6.1110 -44.1310

H -5.4590 6.4900 -44.2510

C -8.0790 6.4260 -37.0910

H -8.2300 5.5380 -36.4600

H -8.0540 7.3180 -36.4470

C -0.9050 6.0530 -35.4490

H -0.7100 5.5220 -36.3930

H -0.8560 7.1330 -35.6590

C -4.5270 6.6110 -47.3810

C -4.8690 6.8910 -53.0340

C -5.0650 6.9610 -54.2260

C -5.4270 7.0470 -55.5830

C -4.5280 6.7380 -56.6210

C -6.7290 7.4530 -55.8840

H -7.3570 7.7230 -55.0500

O -5.4690 6.8840 -46.6690

O -3.3530 6.2310 -46.9090

C -5.9860 6.3400 -66.3640

C -6.6830 6.9360 -60.7800

C -6.6480 7.0750 -59.5780

C -6.3210 7.1290 -58.2120

C -7.2070 7.4990 -57.1860

C -5.0010 6.7820 -57.9170

H -4.3770 6.5340 -58.7600

O -4.8570 6.3360 -66.8340

O -7.0750 6.2620 -67.0850

H -6.8790 6.2510 -68.0750

O -8.4600 7.8560 -57.5540

C -0.9670 5.8830 -56.9360

C -0.6700 5.5720 -55.6230

H -1.4510 5.6650 -54.8870

C 0.6200 5.1620 -55.2910

C 1.5980 5.0720 -56.2940

C 1.2800 5.3580 -57.6300

C -0.0070 5.7830 -57.9350

H -0.2740 6.0350 -58.9500

C 0.2630 5.0170 -52.9110

H -0.8010 4.8430 -53.1120

H 0.3890 6.0610 -52.5900

C 0.7070 4.0650 -51.7990

H 0.4660 3.0430 -52.0970

H 0.1080 4.3110 -50.9200

C 2.1950 4.1380 -51.4480

H 2.5340 5.1730 -51.5060

H 2.3190 3.8070 -50.4140

C 3.0710 3.2600 -52.3430

H 3.0410 3.6330 -53.3660

H 2.6700 2.2430 -52.3450

C 4.5120 3.2170 -51.8390

H 4.9270 4.2280 -51.8430

H 4.5190 2.8650 -50.8040

C 5.4090 2.3070 -52.6750

H 5.4180 2.6600 -53.7080

H 5.0010 1.2940 -52.6760

C 6.8360 2.2810 -52.1320

H 7.2100 3.3060 -52.0700

H 6.8260 1.8720 -51.1190

C 7.7940 1.4600 -52.9920

H 7.8180 1.8740 -54.0030

H 7.4340 0.4310 -53.0630

C 9.2050 1.4700 -52.4090

H 9.5390 2.5060 -52.3080

H 9.1820 1.0320 -51.4080

C 10.2110 0.7070 -53.2660

H 9.8930 -0.3340 -53.3600

H 10.2340 1.1400 -54.2690

C 11.6140 0.7560 -52.6650

H 11.5880 0.3350 -51.6580

H 11.9290 1.7990 -52.5800

C 12.6280 -0.0070 -53.5070

H 12.3520 -1.0560 -53.5820

H 13.6190 0.0540 -53.0630

H 12.6790 0.4110 -54.5100

C 3.8680 5.6790 -56.0590

H 3.5030 6.6340 -55.6630

H 4.1070 5.8160 -57.1210

C 5.0910 5.1830 -55.2950

H 5.0750 4.0950 -55.3730

H 4.9970 5.4400 -54.2400

C 6.4150 5.6980 -55.8480

H 6.4350 5.5500 -56.9310

H 6.5140 6.7680 -55.6560

C 7.5860 4.9420 -55.2250

H 7.4040 3.8700 -55.3310

H 7.6370 5.1610 -54.1570

C 8.9250 5.2720 -55.8770

H 8.8490 5.1110 -56.9560

H 9.1670 6.3240 -55.7150

C 10.0430 4.3940 -55.3210

H 9.7740 3.3440 -55.4630

H 10.1360 4.5640 -54.2460

C 11.3860 4.6600 -55.9930

H 11.2830 4.5210 -57.0720

H 11.6780 5.6980 -55.8200

C 12.4760 3.7300 -55.4690

H 12.1810 2.6940 -55.6510

H 12.5730 3.8570 -54.3880

C 13.8270 3.9920 -56.1290

H 13.7230 3.8910 -57.2130

H 14.1350 5.0190 -55.9220

C 14.9040 3.0310 -55.6330

H 14.5870 2.0020 -55.8220

H 15.0190 3.1460 -54.5530

C 16.2500 3.2720 -56.3120

H 16.1390 3.1440 -57.3910

H 16.5670 4.3020 -56.1340

C 17.3210 2.3190 -55.7960

H 17.4720 2.4570 -54.7280

H 18.2690 2.4960 -56.3010

H 17.0270 1.2860 -55.9680

C 2.0330 5.4820 -59.9020

H 1.8640 6.5620 -60.0030

H 1.1400 4.9540 -60.2600

C 3.2380 5.0500 -60.7420

H 3.1650 5.5530 -61.7090

H 3.1810 3.9750 -60.9150

C 4.5710 5.3730 -60.0720

H 4.6000 4.8540 -59.1130

H 4.6310 6.4440 -59.8770

C 5.7620 4.9220 -60.9130

H 5.6160 3.8790 -61.1990

H 5.8110 5.5130 -61.8300

C 7.0840 5.0390 -60.1570

H 7.2710 6.0830 -59.8990

H 7.0080 4.4760 -59.2230

C 8.2580 4.4890 -60.9640

H 8.0240 3.4700 -61.2790

H 8.3940 5.0920 -61.8640

C 9.5590 4.4710 -60.1640

H 9.4130 3.8800 -59.2560

H 9.8160 5.4880 -59.8600

C 10.7110 3.8720 -60.9680

H 10.4350 2.8630 -61.2860

H 10.8720 4.4720 -61.8660

C 12.0100 3.8030 -60.1700

H 12.3040 4.8110 -59.8660

H 11.8430 3.2180 -59.2620

C 13.1410 3.1650 -60.9730

H 13.3140 3.7470 -61.8810

H 12.8400 2.1590 -61.2770

C 14.4420 3.0780 -60.1800

H 14.7570 4.0850 -59.8910

H 14.2660 2.5100 -59.2640

C 15.5510 2.4080 -60.9800

H 15.2610 1.3970 -61.2590

H 16.4680 2.3530 -60.3980

H 15.7570 2.9680 -61.8900

O 2.8630 4.6760 -55.9220

O 1.0490 4.7910 -54.0620

O 2.2720 5.1710 -58.5390

O -3.2430 6.4260 -56.2860

C -4.7300 6.8410 -51.6330

C -3.5610 6.3850 -51.0210

C -5.8120 7.2440 -50.8420

C -3.4860 6.3120 -49.6460

H -2.7230 6.0860 -51.6340

C -5.7350 7.1610 -49.4710

H -6.7000 7.6240 -51.3220

C -4.5750 6.6870 -48.8630

H -2.5910 5.9550 -49.1620

H -6.5610 7.4600 -48.8450

C -6.5780 6.7710 -62.1720

C -5.2930 6.5760 -62.6980

C -7.6790 6.7960 -63.0320

C -5.1170 6.4310 -64.0530

H -4.4500 6.5520 -62.0250

C -7.4980 6.6370 -64.3890

H -8.6690 6.9440 -62.6230

C -6.2170 6.4610 -64.9090

H -4.1360 6.2920 -64.4790

H -8.3400 6.6560 -65.0640

C -9.3160 8.3820 -56.5630

H -9.3090 7.7420 -55.6730

H -8.9670 9.3870 -56.2800

C -2.3520 6.2810 -57.3660

H -2.7210 5.5050 -58.0530

H -2.2980 7.2280 -57.9260

N -5.3060 5.7480 -28.4090

N -3.4380 6.2960 -44.2640

H -3.3770 6.2330 -45.8960

C -9.2530 6.5340 -38.0280

C -9.1320 6.5120 -39.4040

H -8.1510 6.4450 -39.8420

C -10.2690 6.5710 -40.1990

C -11.5530 6.6420 -39.6250

C -11.6670 6.6450 -38.2230

C -10.5090 6.6150 -37.4560

H -10.5750 6.6370 -36.3820

C -9.0140 6.7770 -42.2150

H -8.3000 5.9750 -41.9740

H -8.5880 7.7330 -41.8760

C -9.2200 6.8060 -43.7280

H -9.5220 5.8120 -44.0620

H -8.2510 7.0280 -44.1790

C -10.2620 7.8290 -44.1930

H -10.2660 8.6850 -43.5150

H -9.9810 8.1890 -45.1850

C -11.6670 7.2330 -44.2650

H -11.9650 6.9000 -43.2700

H -11.6440 6.3610 -44.9230

C -12.6890 8.2300 -44.8060

H -12.8270 9.0370 -44.0810

H -12.3100 8.6780 -45.7280

C -14.0340 7.5630 -45.0880

H -14.3470 7.0080 -44.2010

H -13.9150 6.8430 -45.9020

C -15.1220 8.5680 -45.4540

H -15.2210 9.2960 -44.6450

H -14.8280 9.1150 -46.3540

C -16.4710 7.8900 -45.6850

H -16.7080 7.2710 -44.8170

H -16.4030 7.2310 -46.5530

C -17.5970 8.8990 -45.8960

H -17.6130 9.5960 -45.0540

H -17.4020 9.4800 -46.8010

C -18.9610 8.2230 -46.0070

H -18.9630 7.5450 -46.8620

H -19.1340 7.6240 -45.1100

C -20.0930 9.2350 -46.1580

H -19.9280 9.8300 -47.0580

H -20.0810 9.9160 -45.3030

C -21.4560 8.5610 -46.2390

H -21.5110 7.8990 -47.0990

H -22.2490 9.3020 -46.3250

H -21.6350 7.9720 -45.3430

C -13.8790 6.9760 -40.0890

H -13.8720 7.8430 -39.4200

H -14.2510 6.1080 -39.5350

C -14.8120 7.2620 -41.2610

H -14.7990 6.4190 -41.9500

H -14.4690 8.1470 -41.7970

C -16.2260 7.4740 -40.7220

H -16.5400 6.5670 -40.1980

H -16.2060 8.2860 -39.9910

C -17.2620 7.8060 -41.7920

H -17.3370 6.9790 -42.5000

H -16.9470 8.6920 -42.3450

C -18.6270 8.0600 -41.1560

H -18.9430 7.1630 -40.6170

H -18.5340 8.8680 -40.4270

C -19.7010 8.4340 -42.1730

H -19.8290 7.6140 -42.8830

H -19.3770 9.3120 -42.7370

C -21.0390 8.7320 -41.5020

H -21.3700 7.8460 -40.9520

H -20.9080 9.5410 -40.7790

C -22.1130 9.1250 -42.5100

H -22.2360 8.3190 -43.2350

H -21.7890 10.0130 -43.0580

C -23.4610 9.4040 -41.8510

H -23.7980 8.5020 -41.3310

H -23.3470 10.1950 -41.1070

C -24.5180 9.8180 -42.8720

H -24.5880 9.0540 -43.6480

H -24.2080 10.7480 -43.3560

C -25.8910 10.0120 -42.2350

H -26.1990 9.0790 -41.7570

H -25.8270 10.7780 -41.4580

C -26.9420 10.4170 -43.2600

H -26.6650 11.3520 -43.7440

H -27.9100 10.5530 -42.7840

H -27.0400 9.6520 -44.0270

C -13.0240 6.8700 -36.2540

H -12.6400 7.8700 -36.0040

H -12.4470 6.1170 -35.7040

C -14.4870 6.7270 -35.8420

H -14.5550 6.9730 -34.7800

H -14.7650 5.6810 -35.9650

C -15.4420 7.6100 -36.6420

H -15.4490 7.2700 -37.6780

H -15.0810 8.6400 -36.6330

C -16.8650 7.5660 -36.0880

H -17.1760 6.5260 -35.9740

H -16.8850 8.0240 -35.0960

C -17.8610 8.2790 -37.0000

H -17.5680 9.3240 -37.1150

H -17.8340 7.8180 -37.9900

C -19.2880 8.2120 -36.4590

H -19.5830 7.1660 -36.3540

H -19.3210 8.6640 -35.4640

C -20.2850 8.9220 -37.3720

H -20.2310 8.4870 -38.3720

H -20.0120 9.9760 -37.4550

C -21.7160 8.8110 -36.8530

H -22.0000 7.7570 -36.8070

H -21.7650 9.2110 -35.8370

C -22.7110 9.5580 -37.7380

H -22.4480 10.6190 -37.7590

H -22.6400 9.1790 -38.7600

C -24.1470 9.4050 -37.2440

H -24.2140 9.7550 -36.2100

H -24.4200 8.3470 -37.2530

C -25.1400 10.1830 -38.1030

H -24.8860 11.2450 -38.0720

H -25.0570 9.8500 -39.1400

C -26.5740 9.9940 -37.6260

H -26.8570 8.9450 -37.6740

H -27.2630 10.5640 -38.2450

H -26.6810 10.3280 -36.5950

O -12.5670 6.7080 -40.5410

O -10.2470 6.5530 -41.5630

O -12.9100 6.6600 -37.6500

C -10.7400 8.4570 -57.0400

C -11.1090 8.1210 -58.3280

H -10.3400 7.8360 -59.0250

C -12.4500 8.1240 -58.6810

C -13.4420 8.4640 -57.7440

C -13.0500 8.8490 -56.4520

C -11.7010 8.8430 -56.1240

H -11.3850 9.1320 -55.1370

C -11.9920 7.6920 -60.9790

H -11.2190 6.9430 -60.7490

H -11.4920 8.6610 -61.1280

C -12.7120 7.2710 -62.2590

H -13.1290 6.2720 -62.1150

H -11.9540 7.2100 -63.0430

C -13.8310 8.2210 -62.6960

H -13.5580 9.2490 -62.4490

H -13.9350 8.1580 -63.7820

C -15.1780 7.8810 -62.0600

H -15.1230 8.0420 -60.9830

H -15.3880 6.8210 -62.2320

C -16.3160 8.7060 -62.6590

H -16.1980 9.7540 -62.3750

H -16.2620 8.6550 -63.7510

C -17.6850 8.2030 -62.2090

H -17.7390 8.2290 -61.1190

H -17.8030 7.1610 -62.5180

C -18.8300 9.0290 -62.7920

H -18.7310 10.0680 -62.4700

H -18.7660 9.0170 -63.8830

C -20.1930 8.4920 -62.3620

H -20.2350 8.4470 -61.2710

H -20.3140 7.4720 -62.7340

C -21.3440 9.3560 -62.8720

H -21.2330 10.3720 -62.4850

H -21.2990 9.4130 -63.9620

C -22.7020 8.7980 -62.4520

H -22.8260 7.7990 -62.8730

H -22.7310 8.6980 -61.3640

C -23.8590 9.6860 -62.9040

H -23.8280 9.7910 -63.9910

H -23.7370 10.6820 -62.4720

C -25.2110 9.1160 -62.4920

H -25.3830 8.1540 -62.9690

H -26.0150 9.7900 -62.7810

H -25.2540 8.9690 -61.4150

C -15.8160 8.2710 -57.3500

H -15.9460 9.1650 -56.7310

H -15.6400 7.4150 -56.6890

C -17.0720 8.0510 -58.1870

H -17.0070 7.0900 -58.6960

H -17.1140 8.8290 -58.9500

C -18.3340 8.1110 -57.3310

H -18.3270 7.2920 -56.6080

H -18.3420 9.0450 -56.7630

C -19.5990 8.0270 -58.1810

H -19.6050 7.0830 -58.7290

H -19.5880 8.8360 -58.9140

C -20.8690 8.1340 -57.3420

H -20.9160 7.2950 -56.6440

H -20.8370 9.0520 -56.7490

C -22.1250 8.1370 -58.2090

H -22.1570 7.2190 -58.8010

H -22.0750 8.9770 -58.9060

C -23.4020 8.2490 -57.3810

H -23.4680 7.3960 -56.7010

H -23.3600 9.1550 -56.7710

C -24.6470 8.2870 -58.2610

H -24.6930 7.3760 -58.8630

H -24.5740 9.1330 -58.9480

C -25.9320 8.4130 -57.4480

H -26.0030 7.5750 -56.7500

H -25.8970 9.3330 -56.8580

C -27.1680 8.4310 -58.3420

H -27.2110 7.5020 -58.9170

H -27.0860 9.2540 -59.0540

C -28.4590 8.5880 -57.5430

H -28.5470 7.7650 -56.8290

H -28.4190 9.5190 -56.9730

C -29.6850 8.6070 -58.4470

H -29.6260 9.4350 -59.1500

H -30.5930 8.7200 -57.8600

H -29.7560 7.6810 -59.0140

C -13.6690 9.4730 -54.2160

H -13.1420 10.4330 -54.1390

H -13.0170 8.6730 -53.8450

C -14.9550 9.4910 -53.3940

H -14.6930 9.6850 -52.3530

H -15.4040 8.5000 -53.4530

C -15.9800 10.5120 -53.8820

H -16.0130 10.4750 -54.9720

H -15.6760 11.5180 -53.5950

C -17.3710 10.2100 -53.3320

H -17.6170 9.1680 -53.5490

H -17.3700 10.3330 -52.2470

C -18.4480 11.0980 -53.9450

H -18.2800 12.1390 -53.6620

H -18.3790 11.0420 -55.0350

C -19.8470 10.6650 -53.5140

H -19.9860 9.6110 -53.7670

H -19.9380 10.7600 -52.4290

C -20.9420 11.4850 -54.1880

H -20.8060 11.4440 -55.2720

H -20.8530 12.5320 -53.8880

C -22.3360 10.9710 -53.8400

H -22.4210 9.9250 -54.1460

H -22.4780 11.0090 -52.7570

C -23.4330 11.7850 -54.5210

H -23.3630 12.8260 -54.1970

H -23.2760 11.7680 -55.6030

C -24.8270 11.2500 -54.2050

H -24.9810 11.2580 -53.1240

H -24.9010 10.2120 -54.5390

C -25.9200 12.0770 -54.8760

H -25.8540 13.1100 -54.5280

H -25.7540 12.0810 -55.9560

C -27.3110 11.5310 -54.5780

H -27.3960 10.4980 -54.9090

H -28.0710 12.1200 -55.0870

H -27.5130 11.5620 -53.5100

O -14.7220 8.4360 -58.2330

O -12.9180 7.7920 -59.9240

O -14.0190 9.2250 -55.5670

C -3.7420 3.1310 -54.9150

C -4.7300 3.4710 -55.7570

C -4.6430 3.4000 -57.1970

C -5.7880 3.6300 -57.9900

C -3.4440 3.1120 -57.8490

H -2.5670 2.9740 -57.2420

C -5.3900 3.3130 -62.3420

C -4.3860 3.1740 -61.4600

C -4.4930 3.2470 -60.0200

C -3.3460 3.0170 -59.2260

C -5.6900 3.5510 -59.3680

H -6.5590 3.7320 -59.9750

O -2.1820 2.7060 -59.8680

C -9.2190 4.5930 -57.1540

C -9.0010 4.9060 -55.8250

H -8.0070 4.8210 -55.4220

C -10.0640 5.3390 -55.0380

C -11.3500 5.4500 -55.5870

C -11.5680 5.1060 -56.9320

C -10.4920 4.6740 -57.6950

H -10.6240 4.4000 -58.7280

C -8.8180 5.2700 -53.0010

H -7.8990 5.7340 -53.3920

H -8.7190 4.1790 -53.0930

C -8.9820 5.6590 -51.5330

H -8.9360 6.7450 -51.4480

H -8.1270 5.2430 -50.9960

C -10.2920 5.1730 -50.9010

H -10.6090 4.2450 -51.3810

H -10.1160 4.9560 -49.8460

C -11.4040 6.2150 -51.0120

H -11.5710 6.4490 -52.0640

H -11.0780 7.1290 -50.5090

C -12.7110 5.7410 -50.3790

H -13.1260 4.9260 -50.9770

H -12.5130 5.3470 -49.3790

C -13.7370 6.8710 -50.2820

H -13.7880 7.3890 -51.2430

H -13.4070 7.5990 -49.5370

C -15.1280 6.3580 -49.9170

H -15.4230 5.5930 -50.6400

H -15.0950 5.8830 -48.9340

C -16.1800 7.4660 -49.9160

H -16.1290 8.0070 -50.8650

H -15.9630 8.1810 -49.1190

C -17.5890 6.9060 -49.7370

H -17.7590 6.1290 -50.4880

H -17.6700 6.4360 -48.7540

C -18.6700 7.9750 -49.8810

H -18.5460 8.7230 -49.0960

H -18.5470 8.4800 -50.8430

C -20.0740 7.3800 -49.8050

H -20.2140 6.9030 -48.8330

H -20.1740 6.6030 -50.5650

C -21.1590 8.4300 -50.0160

H -21.1260 9.1810 -49.2310

H -22.1440 7.9690 -50.0070

H -21.0230 8.9310 -50.9730

C -13.4390 5.0750 -54.5290

H -13.1570 4.3550 -53.7450

H -13.7010 4.5230 -55.4370

C -14.6080 5.9400 -54.0830

H -14.7170 6.7310 -54.8260

H -14.3520 6.4040 -53.1310

C -15.9220 5.1770 -53.9530

H -16.1000 4.6090 -54.8690

H -15.8600 4.4670 -53.1250

C -17.0890 6.1360 -53.7240

H -17.1270 6.8450 -54.5560

H -16.9110 6.7070 -52.8110

C -18.4380 5.4300 -53.6140

H -18.5730 4.7670 -54.4710

H -18.4570 4.8140 -52.7120

C -19.5890 6.4330 -53.5720

H -19.6110 6.9900 -54.5130

H -19.4100 7.1540 -52.7720

C -20.9450 5.7680 -53.3540

H -21.0950 4.9900 -54.1060

H -20.9600 5.2820 -52.3750

C -22.0860 6.7790 -53.4390

H -22.1290 7.1840 -54.4530

H -21.8820 7.6120 -52.7630

C -23.4400 6.1710 -53.0820

H -23.6160 5.2860 -53.6980

H -23.4290 5.8480 -52.0390

C -24.5770 7.1670 -53.2960

H -24.6070 7.4570 -54.3500

H -24.3800 8.0720 -52.7150

C -25.9360 6.5980 -52.8950

H -26.1070 5.6580 -53.4240

H -25.9350 6.3770 -51.8250

C -27.0680 7.5680 -53.2140

H -26.9170 8.5140 -52.6980

H -28.0260 7.1570 -52.9050

H -27.1090 7.7670 -54.2830

C -13.0950 4.6630 -58.6940

H -12.8600 3.5880 -58.6810

H -12.4710 5.1570 -59.4470

C -14.5590 4.8760 -59.0590

H -14.7370 4.3390 -59.9930

H -14.7260 5.9400 -59.2340

C -15.5200 4.3680 -57.9870

H -15.5450 5.0900 -57.1700

H -15.1510 3.4220 -57.5850

C -16.9260 4.1690 -58.5530

H -17.1510 4.9780 -59.2520

H -16.9510 3.2350 -59.1170

C -18.0000 4.1480 -57.4700

H -17.7990 3.3400 -56.7620

H -17.9610 5.0880 -56.9150

C -19.3950 3.9750 -58.0620

H -19.5560 4.7450 -58.8210

H -19.4600 3.0060 -58.5590

C -20.4960 4.0790 -57.0120

H -20.3890 5.0240 -56.4750

H -20.3880 3.2700 -56.2850

C -21.8860 4.0170 -57.6400

H -21.9750 4.8150 -58.3810

H -22.0050 3.0650 -58.1610

C -22.9990 4.1710 -56.6080

H -22.9360 3.3570 -55.8810

H -22.8560 5.1080 -56.0660

C -24.3820 4.1710 -57.2530

H -24.5290 3.2330 -57.7930

H -24.4360 4.9860 -57.9780

C -25.4970 4.3420 -56.2260

H -25.4360 3.5360 -55.4890

H -25.3510 5.2850 -55.6950

C -26.8770 4.3350 -56.8700

H -26.9700 5.1500 -57.5850

H -27.6530 4.4520 -56.1160

H -27.0490 3.3980 -57.3950

O -12.3430 5.9450 -54.7780

O -9.9500 5.7030 -53.7310

O -12.8340 5.2070 -57.4110

O -6.9340 3.9140 -57.3220

C -3.7650 3.1620 -53.4730

C -2.6100 2.7470 -52.7970

C -4.8490 3.5640 -52.6850

C -2.5890 2.7450 -51.4210

H -1.7450 2.4320 -53.3630

C -4.7230 3.5380 -51.3080

H -5.7690 3.8930 -53.1370

H -1.7210 2.4190 -50.8650

H -5.5290 3.8380 -50.6510

C -5.2790 3.2290 -63.7840

C -6.4390 3.2970 -64.5630

C -4.0720 3.0770 -64.4740

C -6.3420 3.2000 -65.9380

H -7.4020 3.4240 -64.0910

C -4.0900 2.9960 -65.8540

H -3.1330 3.0290 -63.9460

H -7.2230 3.2460 -66.5650

H -3.1730 2.8780 -66.4150

C -1.0370 2.4940 -59.0690

H -0.7850 3.4140 -58.5190

H -1.2280 1.6990 -58.3310

C -8.0970 4.1850 -58.0690

H -7.9100 5.0070 -58.7770

H -8.3940 3.2980 -58.6460

C -6.1790 2.8870 -28.4770

C -6.2960 2.8140 -34.1280

C -6.1050 2.8270 -35.3220

C -5.6910 2.8530 -36.6630

C -6.5790 2.9530 -37.7480

C -4.3140 2.7790 -36.8860

H -3.6910 2.6880 -36.0110

O -5.1130 2.7370 -27.8960

O -7.3040 3.1720 -27.8710

C -4.3540 3.3410 -47.4550

C -4.1410 3.0630 -41.7950

C -4.2650 2.9940 -40.5930

C -4.6630 2.9330 -39.2450

C -3.7770 2.8310 -38.1590

C -6.0400 2.9840 -39.0210

H -6.6650 3.0530 -39.8960

O -5.3950 3.5450 -48.0410

O -3.2070 3.1400 -48.0740

O -2.4420 2.7980 -38.4360

C -10.2150 3.1760 -38.1650

C -10.6150 3.2200 -36.8450

H -9.8660 3.1780 -36.0720

C -11.9640 3.3330 -36.5400

C -12.9370 3.4060 -37.5550

C -12.5180 3.3630 -38.8980

C -11.1620 3.2360 -39.1690

H -10.8170 3.1940 -40.1870

C -11.6360 2.7990 -34.2500

H -10.6930 3.3520 -34.1280

H -11.3900 1.7670 -34.5420

C -12.3770 2.8010 -32.9130

H -12.3900 3.8170 -32.5140

H -11.7960 2.1810 -32.2270

C -13.8180 2.2890 -33.0090

H -13.8970 1.5670 -33.8240

H -14.0840 1.7810 -32.0790

C -14.7980 3.4340 -33.2520

H -14.5260 3.9330 -34.1810

H -14.7050 4.1540 -32.4350

C -16.2470 2.9630 -33.3320

H -16.3870 2.3850 -34.2470

H -16.4700 2.3090 -32.4850

C -17.2140 4.1460 -33.3180

H -16.9090 4.8670 -34.0800

H -17.1510 4.6500 -32.3510

C -18.6570 3.7230 -33.5740

H -18.7180 3.2370 -34.5510

H -18.9600 2.9930 -32.8190

C -19.6170 4.9100 -33.5400

H -19.2730 5.6730 -34.2430

H -19.6050 5.3580 -32.5440

C -21.0440 4.5020 -33.8970

H -21.0450 4.0420 -34.8890

H -21.3940 3.7510 -33.1850

C -22.0040 5.6880 -33.8930

H -22.0300 6.1300 -32.8940

H -21.6350 6.4550 -34.5790

C -23.4170 5.2830 -34.3030

H -23.7900 4.5230 -33.6120

H -23.3850 4.8360 -35.3000

C -24.3700 6.4720 -34.3130

H -24.4470 6.9080 -33.3200

H -25.3650 6.1640 -34.6290

H -24.0140 7.2410 -34.9950

C -15.2880 3.7190 -37.9810

H -15.3370 2.9330 -38.7400

H -15.1220 4.6780 -38.4840

C -16.6110 3.7670 -37.2230

H -16.5150 4.4240 -36.3600

H -16.8600 2.7720 -36.8550

C -17.7010 4.2790 -38.1600

H -17.4650 5.3080 -38.4440

H -17.6960 3.6780 -39.0730

C -19.1020 4.2460 -37.5580

H -19.1120 4.8090 -36.6230

H -19.3810 3.2160 -37.3290

C -20.1170 4.8470 -38.5270

H -19.8580 5.8930 -38.7090

H -20.0530 4.3210 -39.4830

C -21.5520 4.7740 -38.0140

H -21.6140 5.2690 -37.0430

H -21.8370 3.7290 -37.8720

C -22.5240 5.4390 -38.9840

H -22.2620 6.4950 -39.0840

H -22.4200 4.9790 -39.9710

C -23.9760 5.3250 -38.5300

H -24.0800 5.7590 -37.5320

H -24.2530 4.2710 -38.4610

C -24.9270 6.0370 -39.4880

H -24.6720 7.0990 -39.5250

H -24.7960 5.6310 -40.4940

C -26.3870 5.8870 -39.0700

H -26.5170 6.2870 -38.0620

H -26.6450 4.8260 -39.0370

C -27.3340 6.6070 -40.0270

H -27.0810 7.6690 -40.0490

H -27.1970 6.2100 -41.0360

C -28.7920 6.4470 -39.6150

H -29.0750 5.3970 -39.6090

H -29.4450 6.9730 -40.3070

H -28.9540 6.8500 -38.6170

C -13.1370 2.9940 -41.2000

H -13.1080 1.8960 -41.1770

H -12.1640 3.3620 -41.5360

C -14.1780 3.4960 -42.2000

H -14.0260 2.9450 -43.1300

H -13.9780 4.5500 -42.4000

C -15.6210 3.3410 -41.7290

H -15.7760 3.9860 -40.8630

H -15.7960 2.3100 -41.4160

C -16.6200 3.7190 -42.8220

H -16.3220 4.6710 -43.2680

H -16.5960 2.9660 -43.6130

C -18.0430 3.8450 -42.2830

H -18.3390 2.9040 -41.8120

H -18.0650 4.6210 -41.5140

C -19.0470 4.1990 -43.3780

H -18.7070 5.0970 -43.9010

H -19.0880 3.3890 -44.1100

C -20.4460 4.4460 -42.8180

H -20.3970 5.2380 -42.0660

H -20.8030 3.5410 -42.3190

C -21.4410 4.8500 -43.9050

H -21.0690 5.7410 -44.4170

H -21.5110 4.0530 -44.6480

C -22.8290 5.1370 -43.3360

H -23.2180 4.2330 -42.8610

H -22.7490 5.9060 -42.5630

C -23.8090 5.6060 -44.4090

H -23.8930 4.8410 -45.1840

H -23.4220 6.5100 -44.8840

C -25.1930 5.8990 -43.8350

H -25.6070 4.9840 -43.4050

H -25.1020 6.6320 -43.0300

C -26.1480 6.4330 -44.8960

H -25.7620 7.3530 -45.3290

H -27.1240 6.6400 -44.4630

H -26.2750 5.7080 -45.6980

O -14.2210 3.4730 -37.0850

O -12.4360 3.3870 -35.2550

O -13.4490 3.4680 -39.8980

O -7.9060 3.0110 -37.4690

C -6.3440 2.7900 -32.7240

C -7.5190 2.9770 -31.9940

C -5.1350 2.5850 -32.0470

C -7.4830 2.9840 -30.6160

H -8.4530 3.1240 -32.5180

C -5.1040 2.5860 -30.6740

H -4.2350 2.4320 -32.6240

C -6.2760 2.7980 -29.9470

H -8.3830 3.1400 -30.0410

H -4.1830 2.4350 -30.1330

C -4.1320 3.1460 -43.2000

C -5.3650 3.2820 -43.8490

C -2.9590 3.0910 -43.9560

C -5.4230 3.3510 -45.2220

H -6.2670 3.3250 -43.2560

C -3.0230 3.1590 -45.3320

H -2.0090 2.9920 -43.4510

C -4.2520 3.2850 -45.9730

H -6.3630 3.4500 -45.7420

H -2.1250 3.1140 -45.9270

C -1.5620 2.8070 -37.3340

H -1.8370 3.6190 -36.6460

H -1.6420 1.8680 -36.7690

C -8.7760 3.0690 -38.5800

H -8.5340 3.9490 -39.1940

H -8.6490 2.1650 -39.1920

N -3.6210 3.1400 -50.6900

N -5.1950 3.0490 -66.5790

H -3.3650 3.1540 -49.0730

C 0.1480 2.0750 -59.8950

C 0.0190 1.5670 -61.1740

H -0.9600 1.5090 -61.6180

C 1.1500 1.1480 -61.8650

C 2.4270 1.2460 -61.2780

C 2.5420 1.7380 -59.9630

C 1.3920 2.1410 -59.2920

H 1.4480 2.5010 -58.2790

C -0.1230 0.1250 -63.5930

H -0.7760 0.9690 -63.8620

H -0.6220 -0.4670 -62.8130

C 0.1050 -0.7410 -64.8290

H 0.6090 -0.1430 -65.5890

H -0.8810 -1.0110 -65.2140

C 0.9270 -2.0050 -64.5520

H 0.6640 -2.4100 -63.5720

H 0.6690 -2.7570 -65.3030

C 2.4320 -1.7540 -64.6130

H 2.7050 -0.9880 -63.8870

H 2.6770 -1.3710 -65.6080

C 3.2480 -3.0210 -64.3580

H 3.1350 -3.3320 -63.3170

H 2.8710 -3.8370 -64.9780

C 4.7240 -2.7960 -64.6720

H 5.0850 -1.9450 -64.0930

H 4.8300 -2.5410 -65.7300

C 5.5900 -4.0140 -64.3640

H 5.4990 -4.2600 -63.3040

H 5.2230 -4.8700 -64.9330

C 7.0570 -3.7590 -64.7100

H 7.4060 -2.8770 -64.1700

H 7.1400 -3.5410 -65.7780

C 7.9570 -4.9440 -64.3700

H 7.8810 -5.1610 -63.3020

H 7.6100 -5.8250 -64.9140

C 9.4190 -4.6800 -64.7280

H 9.5010 -4.4960 -65.8010

H 9.7620 -3.7790 -64.2140

C 10.3210 -5.8520 -64.3470

H 9.9710 -6.7520 -64.8580

H 10.2430 -6.0320 -63.2730

C 11.7790 -5.6030 -64.7140

H 11.8870 -5.4790 -65.7900

H 12.4000 -6.4400 -64.4010

H 12.1480 -4.7000 -64.2320

C 4.6900 1.4060 -62.0910

H 5.0190 1.7080 -61.0930

H 4.5740 2.3030 -62.7160

C 5.7050 0.4440 -62.6990

H 5.5280 0.3570 -63.7720

H 5.5290 -0.5340 -62.2480

C 7.1490 0.8610 -62.4370

H 7.3540 1.8160 -62.9250

H 7.2920 0.9960 -61.3610

C 8.1310 -0.1940 -62.9370

H 8.0520 -0.2770 -64.0240

H 7.8580 -1.1610 -62.5100

C 9.5760 0.1200 -62.5570

H 9.8630 1.0870 -62.9760

H 9.6530 0.1920 -61.4690

C 10.5360 -0.9560 -63.0550

H 10.4780 -1.0160 -64.1440

H 10.2230 -1.9240 -62.6540

C 11.9810 -0.6910 -62.6400

H 12.3010 0.2780 -63.0300

H 12.0400 -0.6470 -61.5490

C 12.9210 -1.7790 -63.1500

H 12.8770 -1.8130 -64.2410

H 12.5810 -2.7470 -62.7750

C 14.3660 -1.5570 -62.7090

H 14.7120 -0.5860 -63.0730

H 14.4090 -1.5350 -61.6170

C 15.2930 -2.6530 -63.2270

H 15.3000 -2.6350 -64.3190

H 14.9050 -3.6260 -62.9170

C 16.7200 -2.4990 -62.7090

H 17.1130 -1.5260 -63.0120

H 16.7110 -2.5260 -61.6170

C 17.6330 -3.6010 -63.2320

H 17.2670 -4.5770 -62.9210

H 18.6440 -3.4760 -62.8500

H 17.6720 -3.5810 -64.3190

C 3.8730 1.9800 -57.9870

H 3.4590 1.1000 -57.4750

H 3.3120 2.8670 -57.6800

C 5.3280 2.1970 -57.5830

H 5.3580 2.1770 -56.4920

H 5.6370 3.1900 -57.9110

C 6.3120 1.1720 -58.1340

H 6.3420 1.2580 -59.2210

H 5.9790 0.1640 -57.8880

C 7.7100 1.4110 -57.5650

H 7.9520 2.4740 -57.6370

H 7.7170 1.1420 -56.5060

C 8.7800 0.6100 -58.2990

H 8.5150 -0.4480 -58.2860

H 8.8130 0.9340 -59.3420

C 10.1630 0.7900 -57.6780

H 10.4040 1.8550 -57.6300

H 10.1500 0.4060 -56.6550

C 11.2400 0.0630 -58.4770

H 11.2820 0.4830 -59.4850

H 10.9690 -0.9920 -58.5660

C 12.6180 0.1720 -57.8330

H 12.8900 1.2260 -57.7340

H 12.5800 -0.2570 -56.8290

C 13.6850 -0.5540 -58.6480

H 13.4080 -1.6070 -58.7400

H 13.7180 -0.1280 -59.6540

C 15.0700 -0.4530 -58.0160

H 15.0320 -0.8560 -57.0010

H 15.3620 0.5970 -57.9460

C 16.1210 -1.2110 -58.8210

H 15.8210 -2.2590 -58.9050

H 16.1680 -0.7950 -59.8300

C 17.5010 -1.1310 -58.1820

H 17.8280 -0.0960 -58.1080

H 18.2300 -1.6790 -58.7740

H 17.4840 -1.5570 -57.1810

O 3.4510 0.7180 -62.0150

O 1.1160 0.6040 -63.1080

O 3.7810 1.7770 -59.3900

C -0.1300 2.9950 -37.7590

C 0.1990 3.4470 -39.0250

C 1.5370 3.6430 -39.3630

C 2.5350 3.4260 -38.4040

C 2.1940 2.9490 -37.1270

C 0.8570 2.7280 -36.8210

H 0.5700 2.3620 -35.8490

C 1.0100 4.3900 -41.5490

H 0.3550 5.1590 -41.1210

H 0.3970 3.5160 -41.8150

C 1.6880 4.9610 -42.7900

H 2.4900 5.6230 -42.4640

H 0.9450 5.5680 -43.3110

C 2.2370 3.9140 -43.7570

H 1.4760 3.1510 -43.9420

H 2.4380 4.4120 -44.7090

C 3.5270 3.2500 -43.2820

H 3.3390 2.6720 -42.3770

H 4.2490 4.0290 -43.0290

C 4.1100 2.3580 -44.3780

H 3.4870 1.4690 -44.5010

H 4.0840 2.9090 -45.3220

C 5.5550 1.9380 -44.1130

H 5.6030 1.2920 -43.2340

H 6.1480 2.8310 -43.9010

C 6.1530 1.2220 -45.3240

H 5.6510 0.2630 -45.4700

H 5.9660 1.8290 -46.2130

C 7.6590 1.0000 -45.1970

H 7.8640 0.2970 -44.3870

H 8.1370 1.9480 -44.9390

C 8.2670 0.4790 -46.4990

H 7.8510 -0.5050 -46.7270

H 7.9880 1.1530 -47.3130

C 9.7900 0.3890 -46.4330

H 10.1930 1.3610 -46.1380

H 10.0800 -0.3340 -45.6680

C 10.4000 -0.0180 -47.7710

H 10.0830 0.6890 -48.5420

H 10.0220 -1.0040 -48.0490

C 11.9220 -0.0560 -47.7180

H 12.3190 0.9250 -47.4630

H 12.3350 -0.3510 -48.6800

H 12.2600 -0.7670 -46.9670

C 4.6690 2.5760 -38.9390

H 4.3070 1.9970 -39.8000

H 4.6370 1.9350 -38.0510

C 6.0860 3.0790 -39.1740

H 6.3090 3.8000 -38.3860

H 6.1210 3.6010 -40.1320

C 7.1290 1.9670 -39.1410

H 7.0240 1.4060 -38.2100

H 6.9630 1.2740 -39.9680

C 8.5400 2.5430 -39.2220

H 8.6660 3.2800 -38.4240

H 8.6650 3.0610 -40.1760

C 9.6240 1.4790 -39.0790

H 9.4810 0.9470 -38.1360

H 9.5320 0.7510 -39.8870

C 11.0210 2.0930 -39.1020

H 11.1030 2.8270 -38.2960

H 11.1670 2.6210 -40.0480

C 12.1180 1.0460 -38.9360

H 11.9690 0.5180 -37.9910

H 12.0440 0.3120 -39.7410

C 13.5120 1.6690 -38.9480

H 13.5880 2.3970 -38.1360

H 13.6600 2.2050 -39.8880

C 14.6100 0.6210 -38.7860

H 14.4570 0.0840 -37.8470

H 14.5370 -0.1040 -39.6000

C 16.0050 1.2410 -38.7880

H 16.0760 1.9720 -37.9790

H 16.1620 1.7740 -39.7290

C 17.1020 0.1940 -38.6140

H 16.9420 -0.3430 -37.6760

H 17.0380 -0.5330 -39.4270

C 18.4890 0.8230 -38.6040

H 18.6730 1.3590 -39.5330

H 19.2560 0.0600 -38.4910

H 18.5830 1.5260 -37.7790

C 2.9750 2.0330 -35.0600

H 2.6350 1.0150 -35.2910

H 2.2020 2.5380 -34.4690

C 4.2670 2.0050 -34.2530

H 4.0880 1.4190 -33.3500

H 4.4890 3.0260 -33.9480

C 5.4510 1.4380 -35.0310

H 5.5870 2.0270 -35.9400

H 5.2380 0.4090 -35.3260

C 6.7390 1.4800 -34.2130

H 6.8800 2.4890 -33.8200

H 6.6500 0.8060 -33.3590

C 7.9640 1.0940 -35.0390

H 7.8330 0.0840 -35.4330

H 8.0550 1.7740 -35.8900

C 9.2440 1.1510 -34.2110

H 9.3470 2.1470 -33.7740

H 9.1690 0.4390 -33.3870

C 10.4910 0.8310 -35.0320

H 10.5830 1.5490 -35.8510

H 10.3890 -0.1640 -35.4710

C 11.7520 0.8760 -34.1730

H 11.8450 1.8660 -33.7200

H 11.6530 0.1530 -33.3610

C 13.0180 0.5620 -34.9630

H 12.9290 -0.4330 -35.4060

H 13.1270 1.2810 -35.7790

C 14.2620 0.6110 -34.0790

H 14.1510 -0.1050 -33.2610

H 14.3480 1.6040 -33.6320

C 15.5370 0.2920 -34.8550

H 15.4530 -0.7070 -35.2890

H 15.6450 1.0030 -35.6770

C 16.7730 0.3550 -33.9680

H 16.8780 1.3450 -33.5280

H 17.6710 0.1360 -34.5430

H 16.7000 -0.3690 -33.1590

O 3.8390 3.7140 -38.7400

O 1.9770 4.0360 -40.5850

O 3.2200 2.7360 -36.2680

H -7.1730 3.3300 -26.8880

H -2.5110 5.8350 -39.6330

H -5.5160 6.3820 -39.6160

H -3.4980 5.5840 -33.1190

H -6.4850 6.2100 -33.0160

H -2.8050 2.7700 -55.3170

H -5.6810 3.8080 -55.3730

H -3.3850 2.9820 -61.8160

H -6.3970 3.4880 -61.9890

H -0.5960 3.6050 -39.7340
